# A catalytic enantioselective stereodivergent aldol reaction

**DOI:** 10.1126/sciadv.adg8776

**Published:** 2023-03-15

**Authors:** Md. Ataur Rahman, Torsten Cellnik, Brij Bhushan Ahuja, Liang Li, Alan R. Healy

**Affiliations:** ^1^ Chemistry Program, New York University Abu Dhabi (NYUAD), Saadiyat Island, Abu Dhabi, United Arab Emirates (UAE).; ^2^ Department of Sciences and Engineering, Sorbonne University Abu Dhabi, Abu Dhabi, United Arab Emirates (UAE).

## Abstract

The aldol reaction is among the most powerful and strategically important carbon–carbon bond–forming transformations in organic chemistry. The importance of the aldol reaction in constructing chiral building blocks for complex small-molecule synthesis has spurred continuous efforts toward the development of direct catalytic variants. The realization of a general catalytic aldol reaction with control over both the relative and absolute configurations of the newly formed stereogenic centers has been a longstanding goal in the field. Here, we report a decarboxylative aldol reaction that provides access to all four possible stereoisomers of the aldol product in one step from identical reactants. The mild reaction can be carried out on a large scale in an open flask, and generates CO_2_ as the only by-product. The method tolerates a broad substrate scope and generates chiral β-hydroxy thioester products with substantial downstream utility.

## Teaser

A small change in catalyst structure enables synthesis of all stereoisomers in a mild, scalable decarboxylative aldol reaction.

## INTRODUCTION

The construction of carbon–carbon bonds with complete control of the stereochemical outcome of the reaction is of paramount importance for organic synthesis. The aldol reaction is one of the most powerful methods of forming carbon–carbon bonds (*1*). The process unites two carbonyl fragments to generate β-hydroxy carbonyl compounds with up to two stereocenters. The development of catalytic variants has been the focus of considerable efforts, which have primarily been motivated by the importance of the asymmetric aldol reaction in the total synthesis of polyketides and stereochemically diverse building blocks for drug discovery. Substantial advances have been made in the development of catalytic aldol reactions that use preformed enolates as aldol donors (*2*–*6*). A more formidable challenge is the development of the direct aldol reaction, an atom-economic process wherein the enolate is catalytically generated in situ (*7*). This reaction necessitates the chemoselective formation of the active enolate in the presence of an inherently more acidic aldehyde acceptor in a catalytic system. There have been several notable successes in this area, including the development of direct aldol reactions using organocatalysts (*8*–*10*), biological catalysts (*11*–*13*), and metal complexes (*14*–*16*). In general, the challenge of chemoselectively activating the pronucleophilic carbonyl in the presence of the acceptor aldehyde has been circumvented by the judicious selection of acidic pronucleophiles such as activated ketones and aldehydes, or through the use of non-enolizable aldehyde acceptors. An alternative direct-type approach involving the chemoselective activation of latent pronucleophilic carbonyls in situ has provided a more general solution to this challenge (*17*–*22*). In particular, malonic acid half thioesters (MAHTs) have proven to be powerful latent pronucleophiles, which undergo decarboxylation under mild conditions to generate the active enolate in a catalytic aldol reaction (*23*–*26*). While remarkable progress toward an enantioselective catalytic aldol has been realized, gaining access to all four possible stereoisomeric products in one step from identical substrates remains an important and unmet synthetic challenge (*27*).

## RESULTS AND DISCUSSION

**Fig. 1.**
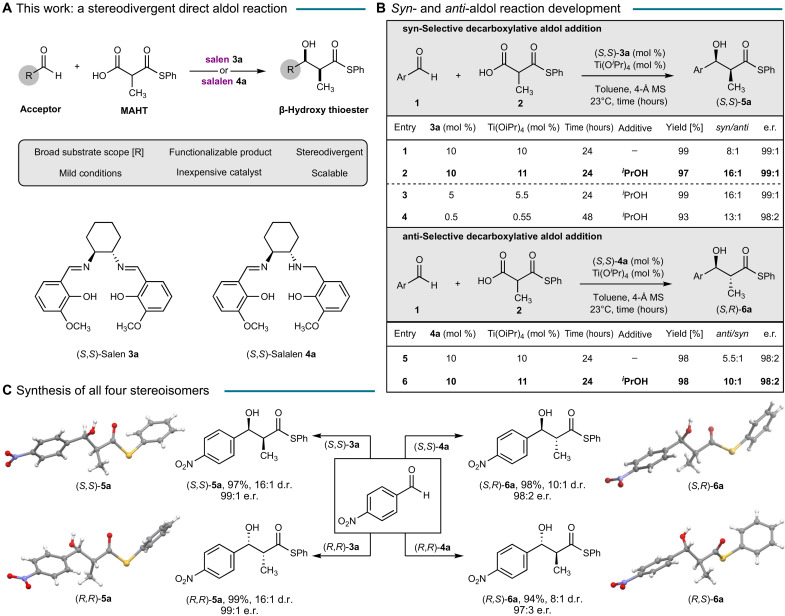
The development of a stereodivergent direct aldol reaction. (**A**) A stereodivergent catalytic aldol reaction using MAHT as a latent pronucleophile. The chiral salen ligand **3a** and salalen ligand **4a** are used in this study. (**B**) *Syn*- and *anti*-aldol reaction development. Ar = 4-NO_2_C_6_H_4_. Details on the complete optimization of the reaction conditions can be found in the Supplementary Materials. The *syn*/*anti* ratio and e.r. values were determined by chiral high-performance liquid chromatography (HPLC); d.r., diastereomeric ratio; e.r., enantiomeric ratio; *^i^*PrOH, 2-propanol; MS, molecular sieves. (**C**) Synthesis of all four stereoisomers of the aldol product from identical substrates. Red, oxygen; blue, nitrogen; gray, carbon; yellow, sulfur; white, hydrogen.

We set our sights on developing a stereodivergent decarboxylative aldol reaction inspired by the mild conditions of the reaction and the downstream utility of the thioester products (Fig. 1A). We hypothesized that coordination of MAHT by a metal-salen complex would catalytically generate the active enolate in situ. An initial investigation of the aldol reaction between aldehyde **1** and MAHT **2** revealed that Ti(O*^i^*Pr)_4_-salen **3a** is a competent catalyst, generating the *syn*-aldol product (*S*,*S*)-**5a** in high yield and enantioselectivity with moderate diastereoselectivity (Fig. 1B, entry 1). The addition of 2-propanol [100 mole percent (mol %)] and a small excess of Ti(O*^i^*Pr)_4_ (1 mol %) resulted in a highly robust and reproducible transformation that provided the *syn*-adduct in high yield and stereoselectivity (entry 2). We found that the catalyst loading could be reduced to 5 mol % without compromising the yield and stereoselectivity (entry 3). Indeed, as little as 0.5 mol % of the catalyst promoted the reaction efficiently, although extended reaction time was required (entry 4).

An attractive feature of salen ligands is their synthetic accessibility in one step by condensation of a salicaldehyde derivative and an inexpensive chiral 1,2-diamine (*28*, *29*). The modularity of the synthesis allows for the systematic tuning of the steric and electronic properties of the catalyst by modification of either component, thereby providing an opportunity to alter the stereochemical outcome of the reaction. The minor diastereomer formed in the reaction was identified as the *anti*-aldol product (*R*,*S*)-**6a**. Attempts to identify conditions that favor the formation of the *anti*-(*R*,*S*)-**6a** by screening a library of salen ligands were unsuccessful (table S2). Instead, our extensive ligand survey revealed that the dihydrosalen (salalen) ligand **4a** generated the *anti*-aldol product (*S*,*R*)-**6a** (entry 5). Using our previously optimized conditions, ligand **4a** provided the *anti*-product (*S*,*R*)-**6a** in high yield (98%) and selectivity [10:1 diastereomeric ratio (d.r.), 98:2 enantiomeric ratio (e.r.)]. Both enantiomers of the salen **3a** ligand are readily synthesized from the commercially available chiral diamines. Partial reduction of (*S*,*S*)- and (*R*,*R*)-salen ligands **3a** affords the corresponding enantiomeric salalen **4a** ligands. Consequently, a completely catalyst-controlled reaction providing access to all four aldol products was achieved solely by choice of the respective ligand (Fig. 1C). The structure of the all four stereoisomers were unambiguously confirmed by x-ray crystallography.

**Fig. 2.**
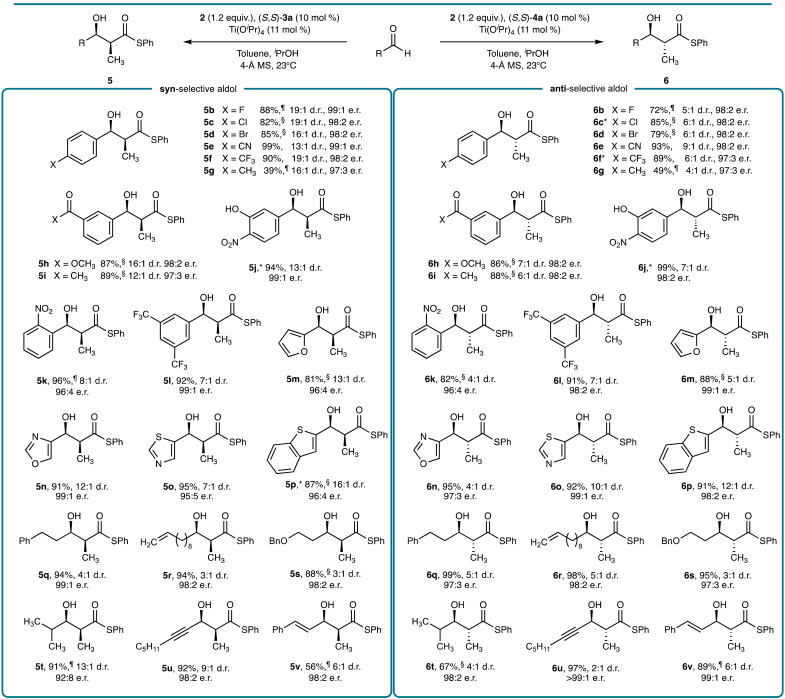
Aldehyde substrate scope for the *syn*- and *anti*-selective aldol reaction. Reactions were performed on a 1.0- to 2.0-mmol scale with respect to the aldehyde in toluene (0.1 M) for 24 hours unless otherwise stated. Isolated yields after chromatographic purification are reported. ^§^Reaction was performed for 48 hours. ^¶^Reaction was performed for 48 hours in toluene (0.4 M). The d.r. and e.r. values were determined by chiral HPLC. ^*^The structure was confirmed by x-ray crystallography.

With the optimized reaction conditions in hand, the scope of both the *syn*- and *anti*-selective decarboxylative aldol reaction was explored (Fig. 2). Aromatic aldehydes with diverse substitution patterns and functional groups were smoothly converted into the corresponding aldol product. The presence of halogens [F (**b**), Cl (**c**), and Br (**d**)] and trifluoromethyl (**f**), methyl (**g**), and nitro (**k**) substituents were all tolerated. The mildness of the reaction conditions as highlighted by the absence of any exogenous base enables the reaction to tolerate reactive functionalities such as a cyano (**e**), methyl ester (**h**), methyl ketone (**i**), and an unprotected hydroxyl group (**j**). Multisubstituted aromatic aldehydes (**l**) and oxygen, nitrogen, and sulfur containing heteroaromatic aldehydes (**m**, **n**, **o** and **p**) provided the aldol product in good yield and selectivity. This method was also compatible with enolizable aliphatic aldehydes, including linear aldehydes containing phenyl (**q**), terminal olefin (**r**), and ether (**s**) functionalities. Branched (**t**) and α,β-unsaturated aldehydes (**u** and **v**) were also smoothly converted into the corresponding aldol product. As no side product formation occurred during the reaction, the conversion of sluggish substrates could be improved by increasing the reaction time and/or concentration (table S4). The Supplementary Materials also includes a further nine examples of aromatic/aliphatic aldehyde substrates (fig. S3). Essentially the same set of aldehydes performed equally well in the *syn*- or *anti*-aldolization reactions, although with higher diastereoselectivities observed for several substrates in the *syn*-aldolization. Notably, high enantioselectivities were obtained for both the *syn*- and *anti*-aldol reaction, irrespective of the steric and electronic properties of the aldehyde substrate.

**Fig. 3.**
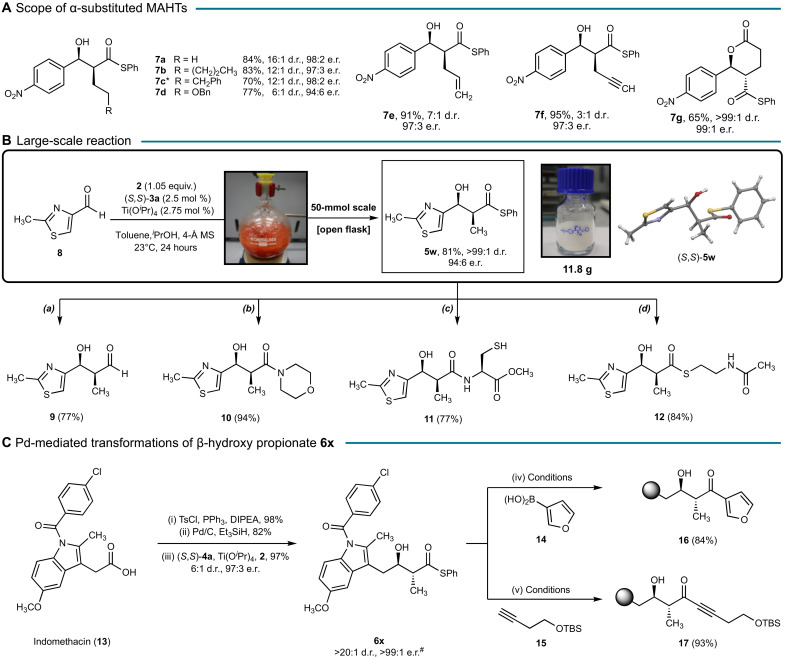
Scope and application of the stereodivergent aldol reaction. (**A**) MAHT substrate scope for the *syn*-selective aldol reaction. See the Supplementary Materials for experimental details. ^*^The structure was confirmed by x-ray crystallography. (**B**) Multigram scale synthesis and functionalization of the thioester product **5w**. (a) Pd(OAc)_2_ (32 mol %), Et_3_SiH (13.5 equiv.), MgSO_4_, and acetone, 23°C, 2 hours. (b) Morpholine (3.0 equiv.), AgTFA (1.1 equiv.), and toluene, 60°C, 20 min. (c) Cysteine methyl ester (1.0 equiv.), DIPEA (1.0 equiv.), dithiothreitol (2.0 equiv.), and DMF, 23°C, 5 hours. (d) *N*-acetylcysteamine (9.4 equiv.), DIPEA (4.6 equiv.), and DMF, 23°C, 24 hours. (**C**) Synthesis and Pd-mediated cross-coupling of **6x**. Reaction conditions: (iv) (a) TBSOTf (1.5 equiv.), 2,6-lutidine (2 equiv.), and DCM, 0°C, 2 hours. (b) **14** (2 equiv.), Pd_2_dba_3_ (5 mol %), CuTC (1.5 equiv.) P(OEt)_3_ (40 mol %), and THF, 23°C, 18 hours. (c) TFA (5 equiv.), DCM, 23°C, 20 hours (v) **17** (1.2 equiv.), Pd(dppf)Cl_2_ (10 mol %), tri(2-furyl)phosphine (25 mol %), CuI (2 equiv.), DIPEA (1.01 equiv.), and DMF, 50°C, 24 hours. ^#^After recrystallization. Gray sphere denotes indomethacin core. DIPEA, *N*,*N*-diisopropylethylamine; DMF, *N*,*N*-dimethylformamide; DCM, dichloromethane; TFA, trifluoroacetic acid.

We next explored the engagement of various α-substituted MAHTs in the direct aldol reaction (Fig. 3A). In addition to longer alkyl chains (**7a** and **7b**), MAHT derivatives containing a phenyl (**7c**) and benzyl ether (**7d**) substituent were tolerated. Allyl (**7e**)– and propargyl (**7f**)–substituted MAHTs were also suitable substrates, providing the *syn*-aldol products with a functional handle for further modification. The aldol reaction with an ethoxy-substituted MAHT provided the aldol product in addition to a small quantity of the lactone **7g**. Exposure of the product mixture to *p*-toluenesulfonic acid catalyzed the quantitative conversion of the aldol product to the lactone **7g**.

The enantioenriched β-hydroxy thiopropionate products are synthetically important building blocks, which can be easily transformed into precursors to natural products and pharmaceutically active compounds (Fig. 3). To demonstrate the synthetic utility of our method, we performed the *syn*-aldol reaction of aldehyde **8** on a 50-mmol scale, furnishing (*S*,*S*)-**5w** in 81% yield after purification (d.r., >99:1). This reaction was performed open to air, with a catalyst loading of 2.5 mol % and only a slight excess of MAHT **2** (1.05 equiv.). Fukuyama reduction of the thioester **5w** provided the aldehyde **9**, which is a valuable intermediate for subsequent asymmetric carbon–carbon bond–forming reactions (*30*). The thioester **5w** was also converted to a diverse array of carboxylic acid derivatives under mild conditions and without recourse to protecting groups. Coupling reagent–free amide bond formation with morpholine furnished amide **10** in high yield. Alternatively, thioester **5w** can be used directly in a native chemical ligation reaction with cysteine to generate **11** (*31*). Transthioesterification of **5w** was achieved under mild conditions to give the *N*-acetylcysteamine thioester **12**, an important chemical probe to interrogate biosynthetic pathways (*32*). An additional notable advantage of using thioesters as carboxylic acid equivalents is their participation in mild Pd-mediated transformations with diverse coupling partners to generate enantiomerically enriched β-hydroxyketones (*33*). The *anti*-β-hydroxy thiopropionate **6x** was synthesized in three steps from the nonsteroidal anti-inflammatory drug indomethacin (**13**) (*34*). Subsequent coupling of **6x** with the boronic acid **14** or the alkyne **15** produced **16** and **17**, respectively, in high yield and without any loss in stereochemical integrity.

In summary, a scalable, stereodivergent decarboxylative aldol reaction has been described using an inexpensive catalyst and a simple reaction setup. The exceptionally mild method can be carried out open to air and generates CO_2_ as the only by-product. The broad substrate scope of the aldol reaction and synthetic utility of the β-hydroxy thioester products bode well for the immediate adoption of this protocol in the synthesis of chiral molecules. Moreover, the modular synthesis of the sal(al)en ligands enables ready tuning of the catalyst system for the development of other stereodivergent enantioselective carbon–carbon bond–forming reactions.

## MATERIALS AND METHODS

### General experimental procedures

All reactions were performed in single-neck, flame-dried, round-bottomed flasks fitted with rubber septa under a positive pressure of argon unless otherwise noted. Air- and moisture-sensitive liquids were transferred via syringe or stainless steel cannula. Organic solutions were concentrated by rotary evaporation at 30° to 32°C. Flash-column chromatography was performed using silica gel (60 Å, 40- to 63-μm particle size) purchased from SiliCycle (Quebec City, Canada). Proton nuclear magnetic resonance spectra (^1^H NMR) were recorded at 500 MHz, proton-decoupled carbon nuclear magnetic resonance spectra (^13^C NMR) were recorded at 125 MHz, and fluorine nuclear magnetic resonance spectra (^19^F NMR) were recorded at 470 MHz. Chiral high-performance liquid chromatography was measured on an Agilent 1260 instrument with an OD CHIRALPAK column (4.6 mm by 250 mm, 5 μm) or AD-H CHIRALPAK column (4.6 mm by 250 mm, 5 μm).

### General method for the stereodivergent aldol reaction

To a suspension of the ligand salen **3** (0.10 mmol, 10 mol %) or salalen **4** (0.10 mmol, 10 mol %) and activated molecular sieves (4 Å, 200 mg) in toluene (10 ml) was added 2-propanol (1.00 mmol, 1.00 equiv.) followed by a solution of titanium (IV) isopropoxide (0.50 M in toluene; 0.11 mmol, 11 mol %). The catalyst mixture was stirred for 1 hour at 23°C. MAHT (1.20 mmol, 1.20 equiv.) was added to the catalyst mixture in one portion. The resulting red solution was stirred for 15 min at 23°C followed by addition of the aldehyde (1.00 mmol, 1 equiv.). The reaction mixture was stirred at 23°C until consumption of the aldehyde was observed. The solution gradually turned to a yellow color over the course of the reaction. The product mixture was filtered through a celite and rinsed with ethyl acetate. The filtrate was concentrated, and the residue was purified by column chromatography.

### Large-scale synthesis of *syn*-aldol product 5w

Activated molecular sieves (4 Å, 10 g) were flame-dried in a 1-liter reaction vessel under high vacuum. After cooling to room temperature, the reaction vessel was back-filled with air and toluene (500 ml, 0.1 M) was added. To the suspension was added (*S*,*S*)-salen **3a** (0.478 g, 1.25 mmol, 0.025 equiv.) followed by 2-propanol (3.8 ml, 50.0 mmol, 1.0 equiv.) and a solution of titanium (IV) isopropoxide (0.5 M in toluene; 0.11 mmol, 11 mol %). The catalyst mixture was stirred for 1 hour at 23°C. MAHT **2** (11.0 g, 52.5 mmol, 1.05 equiv.) was added to the catalyst mixture in one portion. The resulting red solution was stirred for 15 min at 23°C followed by addition of the 2-methylthiazole-4-carboxaldehyde (6.36 g, 50 mmol, 1.0 equiv.). The reaction mixture was stirred for 24 hours. The solution gradually turned a clear, yellow color over the course of the reaction. The product mixture was filtered through a plug of celite, and the plug was washed with ethyl acetate. The filtrate was concentrated, and the residue was purified by column chromatography (eluting with hexane initially, grading to 30% diethyl ether-hexane) to give the *syn*-aldol product **5w** as a white solid (11.8 g, 81% yield).

## Supplementary Materials

### This PDF file includes:

Supplementary Text Figs. S1 to S5 Tables S1 to S4 References
