# Supplementary material for: A catalytic enantioselective stereodivergent aldol reaction
Source: Sci Adv. 2023 Mar 15;9(11):eadg8776. doi: 10.1126/sciadv.adg8776 (PMC10017038; doi:10.1126/sciadv.adg8776)
Supplement: Supplementary file 1 — Supplementary Text Figs. S1 to S5 Tables S1 to S4 References [file sciadv.adg8776_sm.pdf]

Science Advances
AAAS

Supplementary Materials for

A catalytic enantioselective stereodivergent aldol reaction

Md. Ataur Rahman *et al.*

Corresponding author: Alan R. Healy, alan.healy@nyu.edu

Sci. Adv. **9**, eadg8776 (2023)
DOI: 10.1126/sciadv.adg8776

This PDF file includes:

Supplementary Text
Figs. S1 to S5
Tables S1 to S4
References

Supplementary figures:

A.

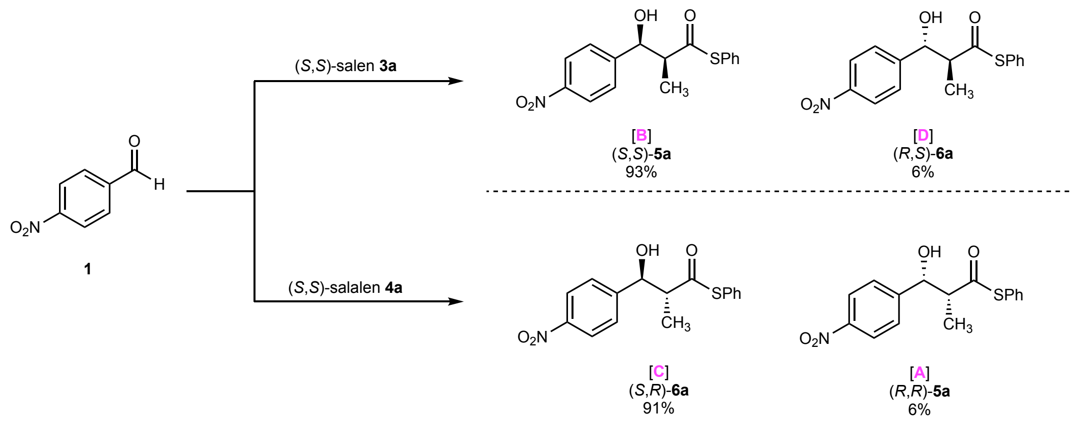

B.

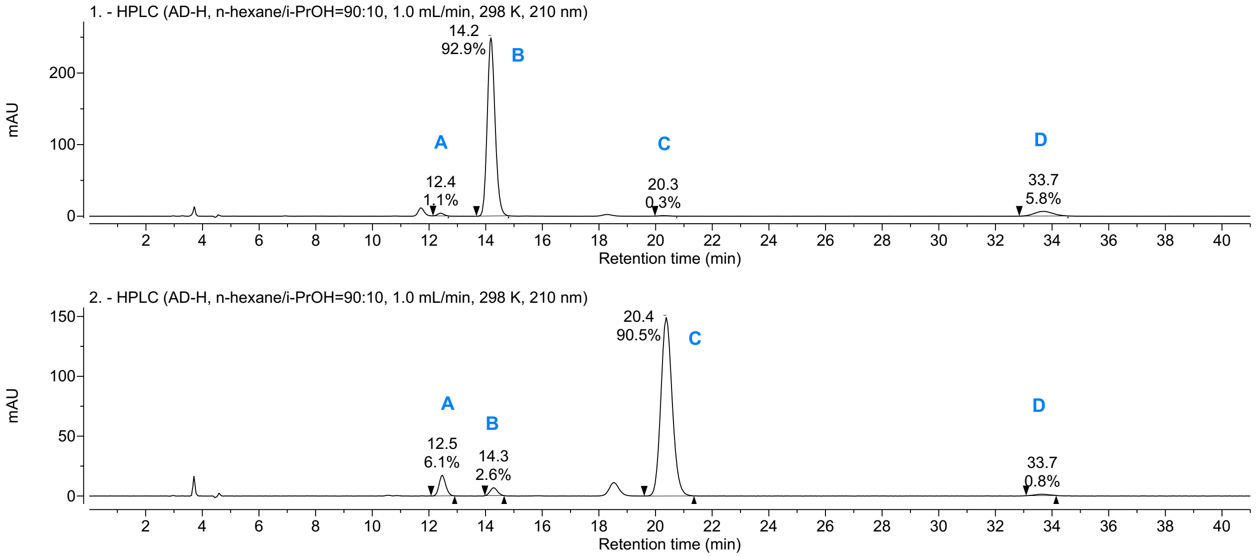

Figure S1. HPLC analysis of the *syn* and *anti*-aldol reaction. A. The aldol reaction of 4-nitrobenzaldehyde **1** and MAHT with (*S,S*)-salen catalyst **3a** provides the *syn*-aldol product (*S*,*S*)-**5a** [B] as the major product. The minor diastereomer is *anti*-aldol (*R*,*S*)-**6a** [D] which is epimeric at the β-hydroxyl stereocenter. The aldol reaction of 4-nitrobenzaldehyde **1** and MAHT with (*S,S*)-salalen catalyst **4a** provides the *anti*-aldol product (*S*,*R*)-**6a** [C] as the major product. The minor diastereomer is the *syn*-aldol (*R*,*S*)-**5a** [A]. **B.** HPLC traces for the salen-aldol reaction (*top*) and salalen-aldol reaction (*bottom*) of 4-nitrobenzaldehyde **1** and MAHT. The four stereoisomers are labeled.

a Symmetrical salen ligands

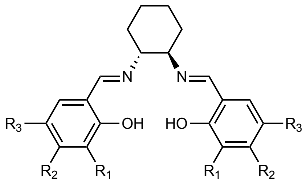

3(a–t)

ligand	R_1	R_2	R_3	ligand	R_1	R_2	R_3
**3a**	OCH_3	H	H	**3k**	H	H	^{t}Bu
**3b**	H	OCH_3	H	**3l**	H	H	Nap
**3c**	H	H	OCH_3	**3m**	OC_2H_5	H	H
**3d**	Cl	H	H	**3n**	$OCH(CH_3)_3$	H	H
**3e**	H	H	H	**3o**	OBn	H	H
**3f**	Cl	H	Cl	**3p**	Ph	H	H
**3g**	I	H	Cl	**3q**	H	H	NO_2
**3h**	Br	H	Br	**3r**	H	$N(C_2H_5)_2$	H
**3i**	I	H	I	**3s***	CH_2(pip)	H	^{t}Bu
**3j**	^{t}Bu	H	^{t}Bu	**3t***	CH_2(mor)	H	^{t}Bu

c Unsymmetrical salen ligands

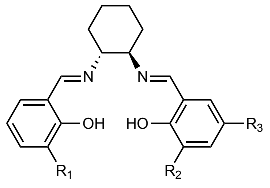

3(u–w)

ligand	R_1	R_2	R_3
**3u**	OCH_3	^{t}Bu	^{t}Bu
**3v**	OCH_3	H	H
**3w**	H	^{t}Bu	^{t}Bu

c Symmetrical salalen ligands

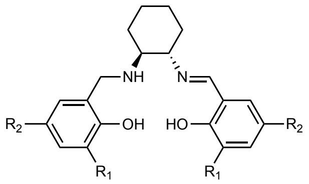

4(a–c)

ligand	R_1	R_2
**4a**	OCH_3	H
**4b**	^{t}Bu	^{t}Bu
**4c**	Ph	H

Figure S2. Structures of the ligands tested in this study. (a) Structures of symmetrical salen ligands. (b) Structures of unsymmetrical salen ligands (c) Structures of salalen ligands. The ligands were synthesized as described in the experimental procedures. *Ligand was purchased and used directly. Nap, naphthyl; pip, piperidine; mor, morpholine.

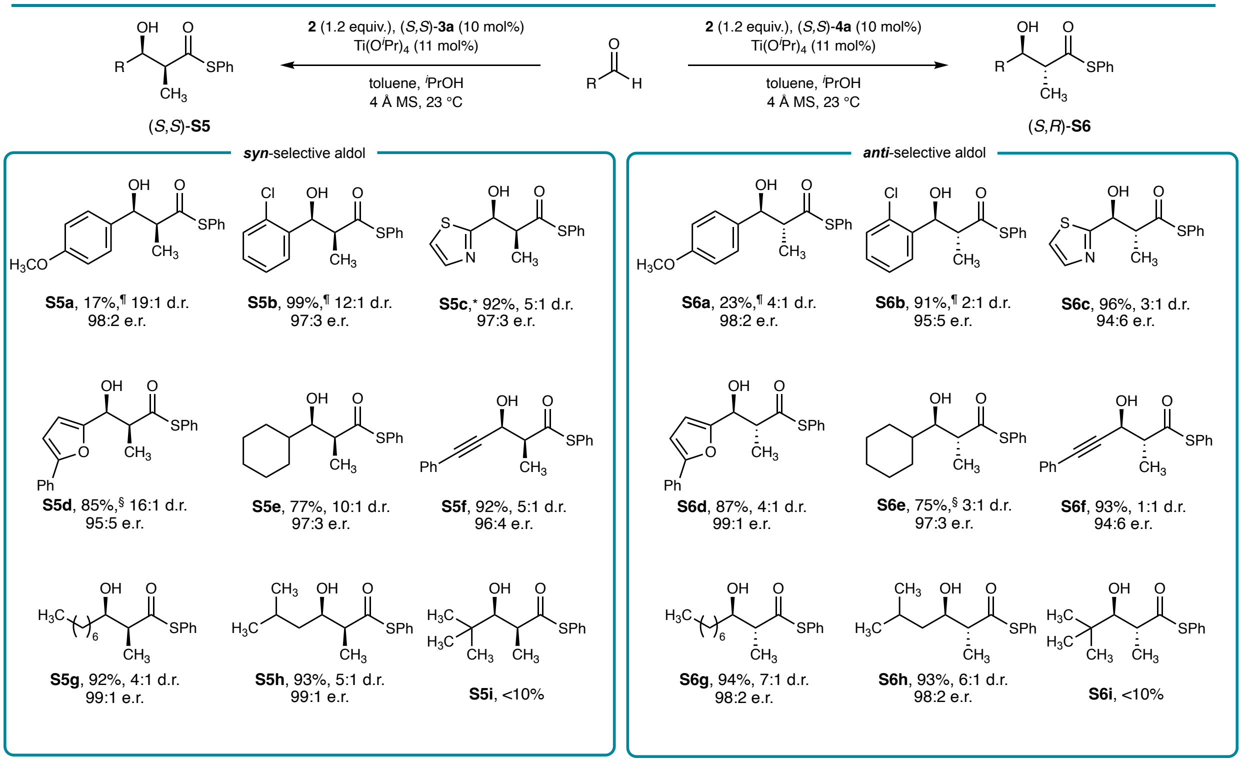

Figure S3. Additional aldehyde substrate scope for the *syn*- and *anti*-selective aldol reaction. Reactions were performed on 1.0- to 2.0-mmol scale with respect to the aldehyde in toluene (0.1 M) for 24 h unless otherwise stated. Isolated yields after chromatographic purification are reported. §Reaction was performed for 48h. ¶Reaction was performed for 48h in toluene (0.4 M). The d.r. and e.r. values were determined by chiral high-performance liquid chromatography; d.r., diastereomeric ratio. *The structure was confirmed by X-ray crystallography.

Electron rich aldehydes (**a**) and highly sterically hindered alkyl aldehydes (**i**) were very sluggish under the reaction conditions.

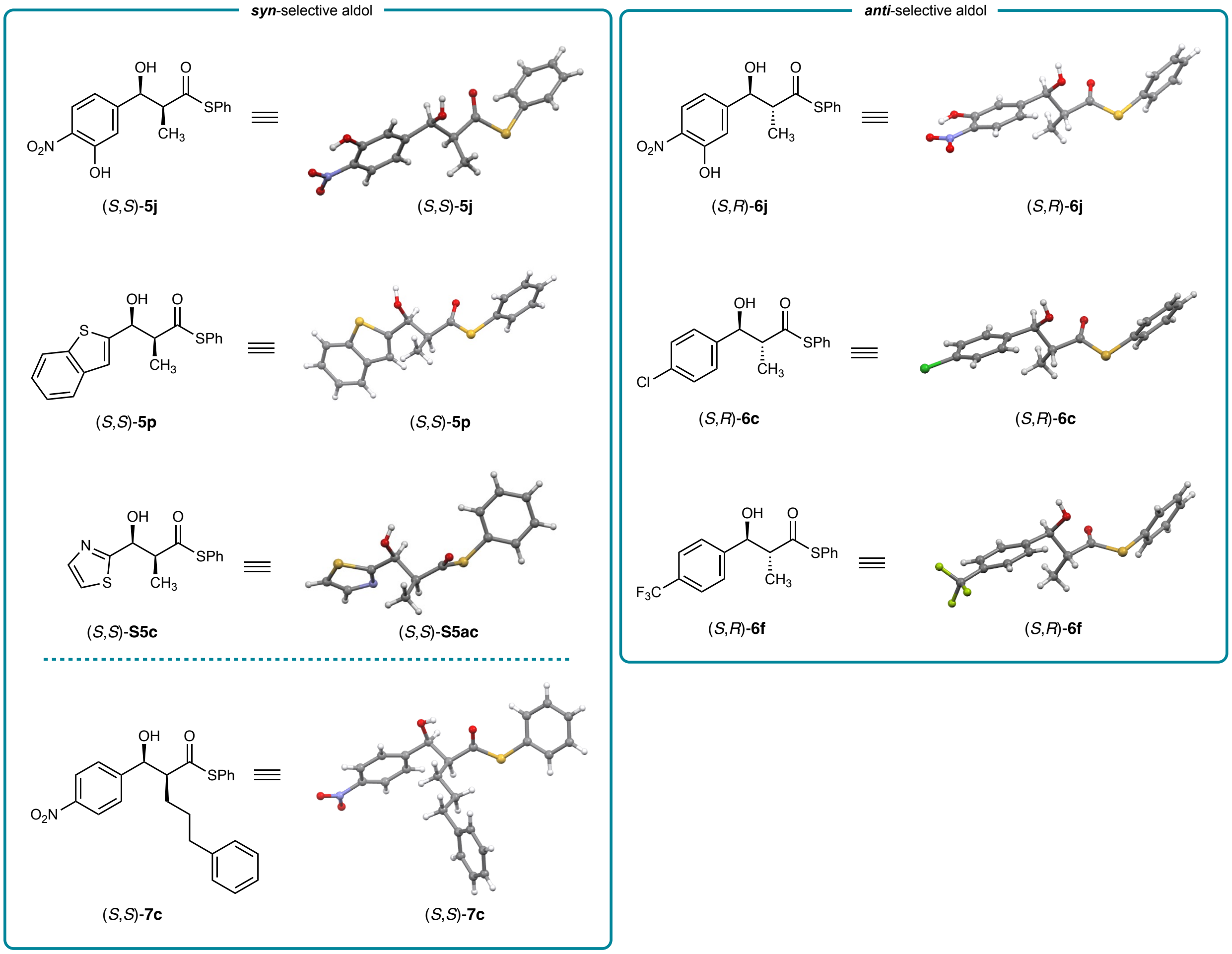

Figure S4. **Additional X-ray crystallographic structures obtained during this study.** Red, oxygen; blue, nitrogen; grey, carbon; yellow, sulfur; green, chlorine; light green, fluorine; white, hydrogen.

Figure S5. Graphical Supporting Information for the *syn*-aldol reaction of 8 (50 mmol).

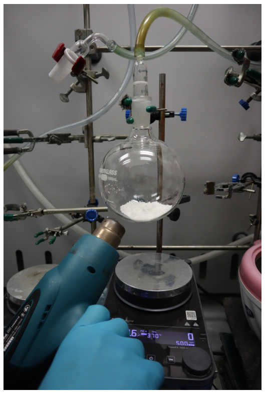
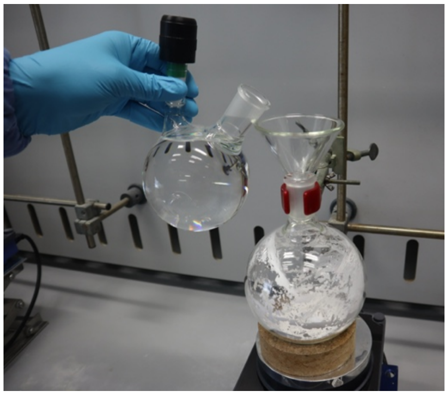
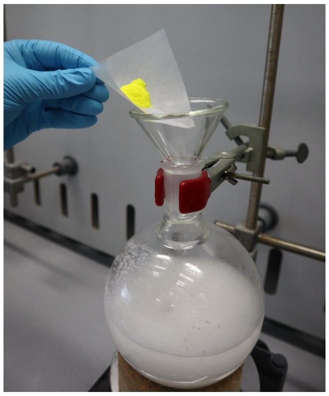

(Left) Activated molecular sieves (10.0 g, 4 Å) were dried by heating for 5 min (heat gun) under vacuum. After cooling down to room temperature, the flask was filled with air. **(Center)** Addition of solvent (toluene, 500 mL). **(Right)** Addition of ligand ((*S,S*)-salen **3a**, 1.25 mmol).

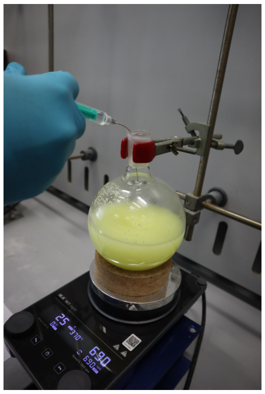
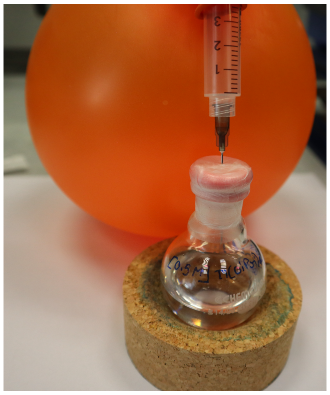
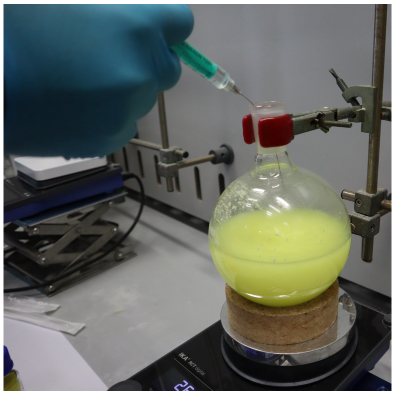

(Left) Addition of ^{i}PrOH (50.0 mmol). **(Center)** A stock solution of Ti(O^{i}Pr)$_4$ in toluene (0.50 M). This can be stored for several weeks under argon. **(Right)** Addition of Ti(O^{i}Pr)$_4$ (1.38 mmol).

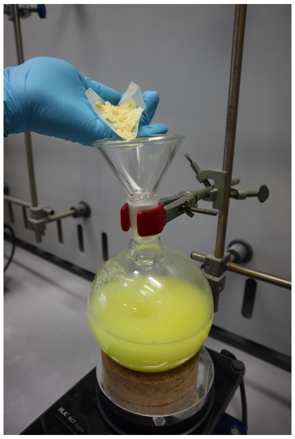
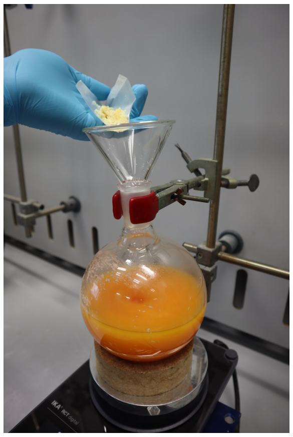
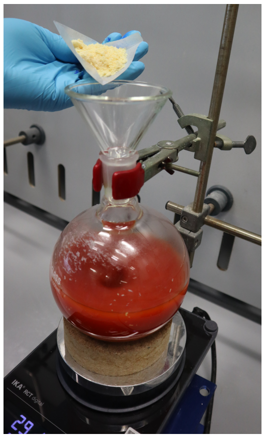

(Left) Start of MAHT **2** addition (52.5 mmol). **(Center)** Slow change of color observed during the addition of MAHT **2** (yellow -> orange -> red). **(Right)** Addition of the aldehyde **8** (50.0 mmol) to the red reaction mixture.

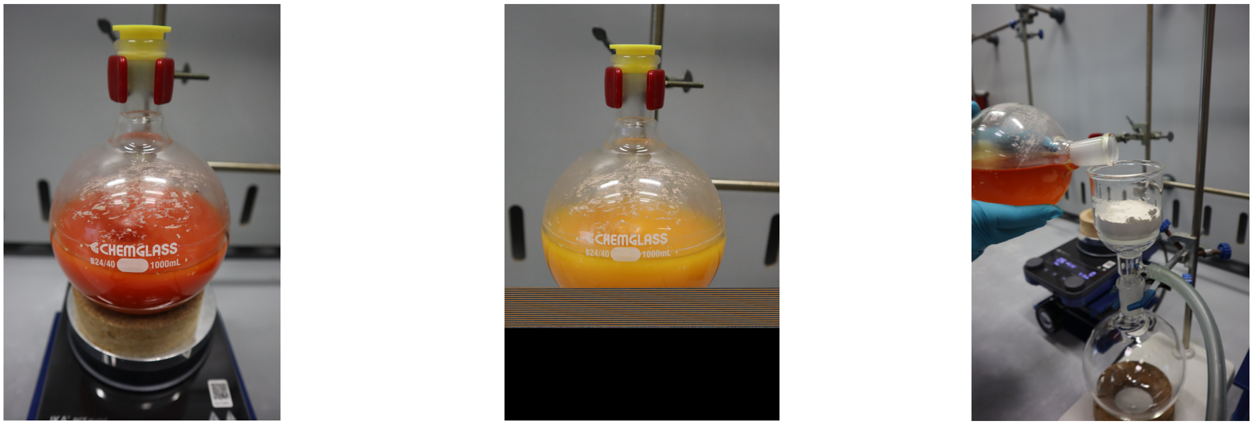

(Left) The reaction at the end of the aldehyde addition. **(Center)** The reaction flask after 24 h. The color changed back to orange/yellow which indicates the consumption of MAHT **2**. **(Right)** Filtration of the reaction mixture through a pad of celite.

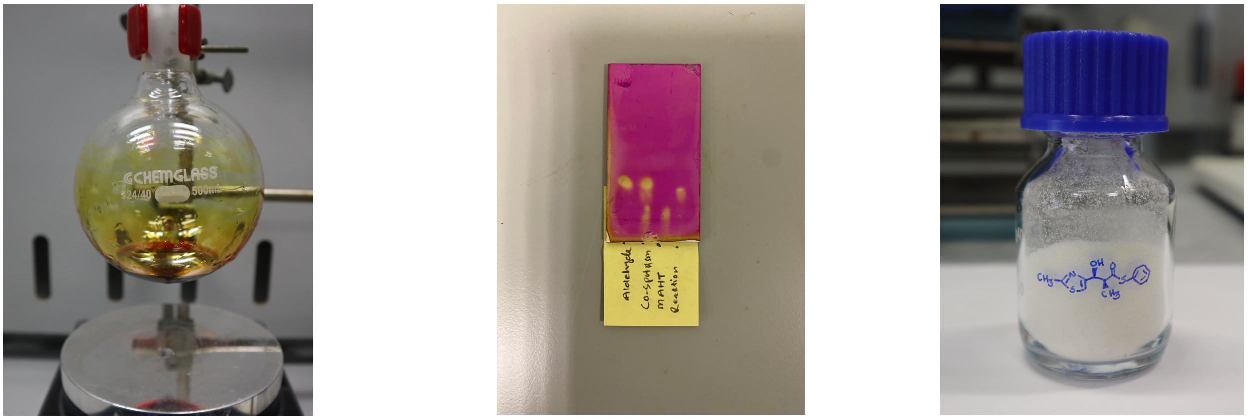

(Left) After removing of the solvent under *vacuo* the crude product was isolated as red oil. **(Center)** The reaction was monitored by TLC: R_f = 0.41 (30% diethyl ether-hexane - $KMnO_4$): *Lane 1* (from left to right): aldehyde; *Lane 2*: Co-spot; *Lane 3*: MAHT; *Lane 4*: reaction mixture after 24 h. **(Right)** Isolated product **5w** (81%, >99:1 d.r.).

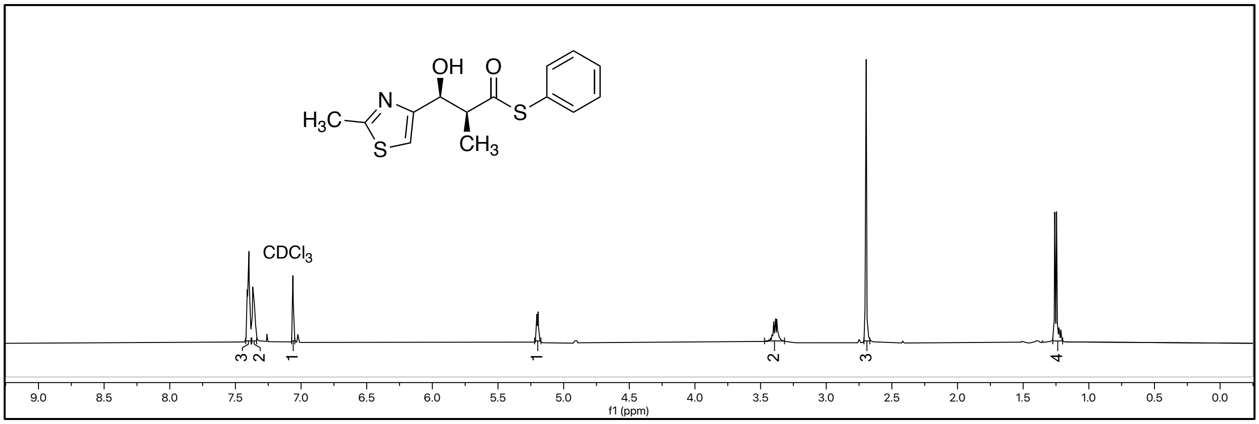

^{1}H NMR of crude product **5w** after filtration and removal of solvent. See NMR catalog for ^{1}H NMR of purified **5w** after column chromatography.

Supplementary tables:

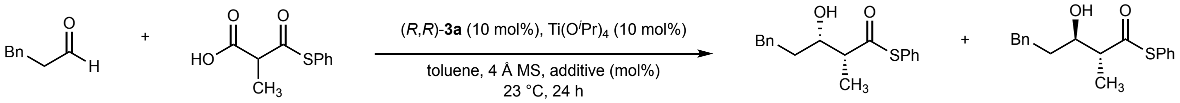

2 (S,R)-5q (R,R)-6q

entry	additive	mol%	yield [%]	**5q/6q**	e.r.
**1**	-	-	99	3.5:1	97:3
**2**	AcOH	10	60	1.9:1	90:10
**3**	CSA	10	56	3.8:1	97:3
**4**	$MgSO_4$	20	97	3.3:1	98:2
**5**	Na_2SO_4	20	99	3.5:1	98:2
**6**	LiCl	20	99	3.5:1	98:2
**7**	$MgBr_2$	100	NR	-	-
**8**	$MgBr_2.OEt_2$	100	NR	-	-
**9**	$MgClO_4$	100	NR	-	-
**10**	$La(O^iPr)_3$	10	85	3:1	96:4
**11**	$Yb(OTf)_3$	10	16	2.4:1	66:34
**12**	$TiCl_4$	10	60	3.8:1	50:50
**13**	$Ti(O^iPr)_4$	1	99	4:1	97:3
**14**	$Ti(O^iPr)_4$	10	99	4:1	97:3
**15**	$Ti(O^iPr)_4$	100	44	1:6.1	72:28
**16**	DIPEA	20	61	1.7:1	95:5
**17**	DMAP	10	44	2.2:1	92:8
**18**	imidazole	10	98	2.6:1	96:4
**19**	5-methoxybenzimidazole	10	96	2.7:1	96:4
**20**	TMSOTf	20	21	2.8:1	91:9
**21**	TMSCl	20	14	1.2:1	83:17
**22**	MeOH	20	65	1.9:1	91:9
**23**	EtOH	20	73	3:1	97:3
**24**	^{n}BuOH	20	89	2.8:1	96:4
**25**	^{i}PrOH	20	99	3.8:1	98:2
**26**	^{i}PrOH	100	97	3.8:1	99:1
**27**	HFIP	20	64	2.3:1	94:6
**28**	^{t}BuOH	20	99	3:1	97:3
**29**	1,2-ethanediol	20	91	3.8:1	98:2
**30**	pentane-2,4-dione	20	80	1.9:1	88:12
**31**	*optimized*		94	4:1	99:1

Table S1. The development of the *syn*-selective aldol reaction – the effect of additive addition. Reaction conditions: hydrocinnamaldehyde (1 equiv.), **2** (1.2 equiv.), **3a** (10%), $Ti(O^iPr)_4$ (10%), additive (mol%), toluene (0.1 M), 23 °C, 24 h. Isolated yields after chromatographic purification are reported. The d.r. and e.r. was determined by chiral HPLC.

Most additives had a minimal effect on the reaction outcome. The addition of strong bases resulted in decarboxylation of the MAHT and lower conversion (entries 16 & 17). The addition of $Ti(O^iPr)_4$ (1 mol%) and ^{i}PrOH (100 mol%) resulted in an increase in stereoselectivity (entries 13 & 26). 2-propanol (^{i}PrOH) is a common additive in salen-$Ti(O^iPr)_4$ catalyzed reactions where it has been demonstrated to accelerate catalyst turnover and promote the formation of the presumed active mononuclear *cis*-β salen-$Ti(O^iPr)_4$ catalyst, in favor of the di-μ-oxotitanium(salen) complex.(*35, 36*) While the observed increase was modest, we did find the additive addition resulted in a highly robust and reproducible transformation. The combination of these two additives provided our optimized conditions (entry 31) which was used for the substrate screening.

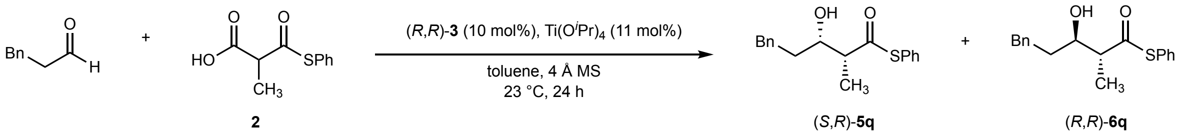

entry	ligand	yield [%]	*syn*/*anti*	e.r.
**1**	3a	>99	4:1	97:3
**2**	3b	>99	2.6:1	95:5
**3**	3c	>99	2.2:1	96:4
**4**	3d	92	2.6:1	96:4
**5**	3e	92	2.1:1	97:3
**6**	3f	94	1.8:1	93:7
**7**	3g	91	1.2:1	91:9
**8**	3h	90	1.4:1	87:13
**9**	3i	95	1.7:1	90:10
**10**	3j	23	1:1.2	ND
**11**	3k	>99	2.2:1	97:3
**12**	3l	>99	1.9:1	98:2
**13**	3m	92	2.8:1	98:2
**14**	3n	>99	1.4:1	90:10
**15**	3o	92	2.3:1	96:4
**16**	3p	98	1:1	91:9
**17**	3q	74	1.5:1	88:12
**18**	3r	96	2.7:1	92:8
**19**	3s	62	1:1.9	95:5[a]
**20**	3t	86	1:2.3	98:2[a]
**21**	3u	>99	1.2:1	96:4
**22**	3v	>99	2.5:1	95:5
**23**	3w	97	1.6:1	93:7

Table S2. The development of the *syn*-selective aldol reaction – ligand survey. Reaction conditions: hydrocinnamaldehyde (1 equiv.), **2** (1.2 equiv.), (*R*,*R*)-**3** (10%), Ti(O^{i}Pr)$_4$ (11%), toluene (0.1 M), 23 °C, 24 h. See Figure S2 for ligand structures. Isolated yields after chromatographic purification are reported. The d.r. and e.r. was determined by chiral HPLC. [a]Enantioselectivity of the major *anti*-aldol product (*R*,*R*)-**6q**. MS = molecular sieves. NR = no reaction.

Hydrocinnamaldehyde was selected as the model substrate for ligand screening due to the modest diastereoselectivity obtained using ligand **3a** (entry 1). The enantioselectivity of the reaction was consistently high across all ligands, however no ligand resulted in a higher diastereoselectivity. Ligands **3s** and **3t** favored the formation of the *anti*-aldol product **6q** (entry 19 & 20), however, attempts to improve on the selectivity of these reactions were unsuccessful.

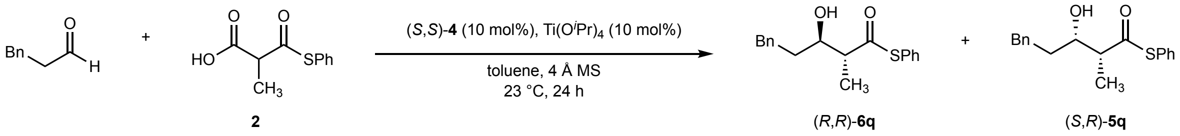

entry	ligand	yield [%]	*anti/syn*	e.r.
**1**	4a	97	4:1	95:5
**2**	4b	40	2.4:1	71:29
**3**	4c	29	1.5:1	33:67
**4**[a]	4a	99	5:1	97:3

Table S3. The development of the *anti*-selective aldol reaction – ligand survey. Reaction conditions: hydrocinnamaldehyde (1 equiv.), **2** (1.2 equiv.), (*S*,*S*)-**4** (10%), Ti(O^{i}Pr)$_4$ (10%), toluene (0.1 M), 23 °C, 24 h. [a]Reaction performed with ^{i}PrOH (100 mol%). Isolated yields after chromatographic purification are reported. The d.r. and e.r. was determined by chiral HPLC.

As part of our ligand survey using hydrocinnamaldehyde as the model substrate we observed that salalen **4a** led to the preferential formation of *anti*-aldol (*R*,*R*)-**6q**. In contrast to what was observed for the salen **3** ligands, changes to the steric or electronic properties of the salalen **4** ligand resulted in a significant drop in yield and selectivity (entries 2 & 3). Application of our previously optimized conditions provided high yield and good selectivity (entry 4).

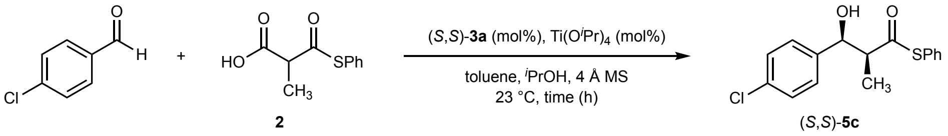

entry	**3a** (mol%)	Ti(O^{i}Pr)$_4$ (mol%)	aldehyde (equiv.)	MAHT (equiv.)	time (h)	yield [%]	*syn/anti*	e.r.
**1**	10	11	1	1.2	24	48	13:1	98:2
**2**	10	11	1	1.2	48	82	19:1	98:2
**3**	10	11	1	1.2	72	76	16:1	97:3
**4**[a]	10	11	1	1.2	24	84	16:1	99:1
**5**	10	11	0.5	1	24	36	19:1	98:2
**6**	10	11	2	1	24	79	10:1	97:3
**7**	10	11	5	1	24	91	7:1	98:2
**8**	10	11	1	0.6	24	37	6:1	96:4
**9**	10	11	1	2.4	24	46	16:1	97:3
**10**	10	11	1	1	24	55	19:1	98:2
**11**	5	5.5	1	1.2	24	37	19:1	97:3
**12**	20	22	1	1.2	24	61	16:1	98:2
**13**	20	22	1	2.4	24	85	19:1	98:2
**14**	50	55	1	1.2	24	37	3:1	92:8

Table S4. Exploration of the effect of reactant stoichiometry and conditions on product formation. Reactions were performed on 1.0 mmol scale with respect to 4-chlorobenzaldehyde in toluene (0.1 M) for 24 h unless otherwise stated. [a]Reaction was performed in toluene (0.4 M).

4-chlorobenzaldehyde was sluggish under our reaction conditions, yielding the *syn*-aldol product **5c** in 48% yield after 24 h under the standard reaction conditions (entry 1). As with our other reactions no side product was observed, and therefore the moderate yield can be attributed to incomplete conversion. Additionally, no decarboxylated MAHT was observed which is in agreement with the decarboxylation step happening after addition to the aldehyde. (*37*) Increasing the reaction time (entry 2) or reaction concentration (entry 4) resulted in increased conversion to the product **5c**. Increasing the aldehyde concentration resulted in higher conversion but a drop in diastereoselectivity (entries 6 and 7). In contrast, increasing the MAHT concentration did not improve the reaction conversion (entry 9). Indeed, reducing the MAHT concentration to 1 equivalent resulted in increased conversion (entry 10). While increasing catalyst concentration did increase conversion (entry 12), a more significant increase was observed when both catalyst and MAHT were increased proportionally (entry 13). This supports an approximate optimal ratio of 10:100 of the catalyst to MAHT in the reaction.

Experimental Procedures

General Information

General Experimental Procedures. All reactions were performed in single-neck, flame-dried, round-bottomed flasks fitted with rubber septa under a positive pressure of argon unless otherwise noted. Reactions that required heating were carried out in temperature-controlled heating blocks. Air- and moisture-sensitive liquids were transferred via syringe or stainless-steel cannula. Organic solutions were concentrated by rotary evaporation at 30–32 °C. Flash-column chromatography was performed employing silica gel (60 Å, 40–63 µm particle size) purchased from SiliCycle (Quebec City, CA). Analytical thin-layered chromatography (TLC) was performed using glass plates pre-coated with silica gel (0.25 mm, 60 Å pore size) impregnated with a fluorescent indicator (254 nm). TLC plates were visualized by exposure to ultraviolet light (UV) or by staining with potassium permanganate ($KMnO_4$) and subsequent heating.
Materials. Commercial solvents and reagents were used as received with the following exceptions. Toluene and dichloromethane were purified and dried *via* PureSolv-system (inert®). Benzaldehyde, cyclohexanecarboxaldehyde, hydrocinnamaldehyde, isobutyraldehyde and octanal were distilled prior usage. The substrates **2**,(*24*) **S11**,(*34*) **S12e**,(*38*) and **S12g**,(*39*) were synthesized according to literature.
Ligand synthesis. The ligands **S3**, **3a**-**f**, **3h**-**m** and **3p**-**3q** were synthesized according a standard method described in the literature.(*40*) Analytical data for **S3** (*41*); **3a**, **3b**, **3c**, **3k**, **3q**,(*42*); **3d** (*43*); **3e** (*44*); **3f** (*45*); **3h**,(*46*); **3i**, **3j**, **3m** (*47*); **3l**,(*48*) and **3p** (*49*); are in accordance with the literature. The ligands **3u** (*50*); **3v**, **3w** (*51*); were synthesized according to the literature. The ligands **3s** and **3t** were purchased from Sigma-Aldrich® and were used as received.
Instrumentation. Proton nuclear magnetic resonance spectra (^{1}H NMR) were recorded at 500 MHz at 20 °C. Chemical shifts are expressed in parts per million (ppm, δ scale) downfield from tetramethylsilane and are referenced using residual undeuterated solvent ($CDCl_3$, δ 7.26). Data are represented as follows: chemical shift, multiplicity (s = singlet, d = doublet, t = triplet, q = quartet, m = multiplet and/or multiple resonances, br = broad, app = apparent), coupling constant in Hertz, integration, and assignment. Proton-decoupled carbon nuclear magnetic resonance spectra (^{13}C NMR) were recorded at 125 MHz at 24 °C. Fluorine nuclear magnetic resonance spectra (^{19}F NMR) were recorded at 470 MHz at 24 °C. Chemical shifts are expressed in parts per million (ppm, δ scale) downfield from tetramethylsilane and are referenced to the carbon resonances of the solvent ($CDCl_3$, δ 77.16). Signals of protons and carbons were assigned, as far as possible, by using [^{13}C] DEPT (Distortionless Enhancement by Polarization Transfer) and the following two-dimensional NMR spectroscopy techniques: [^{1}H, ^{1}H] COSY (Correlation Spectroscopy), [^{1}H, ^{13}C] HSQC (Heteronuclear Single Quantum Coherence) and long range [^{1}H, ^{13}C] HMBC (Heteronuclear Multiple Bond Connectivity). Analytical liquid chromatography/mass spectrometry (LC/MS) was performed on Agilent LC/MS instrument (1260 Infinity II) equipped with a reverse-phase C_{18} column (2.7 µm particle size, 3.0 × 100 mm), electrospray (ESI) mass spectrometry detector, and photodiode array detector.

Samples were eluted with a linear gradient of 30% acetonitrile–water containing 0.1% formic acid→95% acetonitrile-water containing 0.1% formic acid over 3.00 min, followed by 95% acetonitrile-water containing 0.1% formic acid for 1.00 min, at a flow rate of 800 μL/min. High-resolution mass spectrometry (HRMS) was obtained on an UPLC/HRMS instrument (Agilent 1290 Infinity II) equipped with a Q-TOF (UHD Accurate-Mass) and photodiode array detector. Unless otherwise noted, samples were eluted over a reverse-phase C_{18} column (1.8 μm particle size, 2.1 × 50 mm) with a linear gradient of 5% acetonitrile–water containing 0.1% formic acid→100% acetonitrile–water containing 0.1% formic acid for 10 min, at a flow rate of 300 μL/min. Chiral HPLC (high pressure liquid chromatography) was measured on an Agilent 1260 instrument with an OD CHIRALPAK column (4.6 mm × 250 mm, 5 μm) or AD-H CHIRALPAK column (4.6 mm × 250mm, 5 μm).

General methods

General method for racemic aldol reaction:

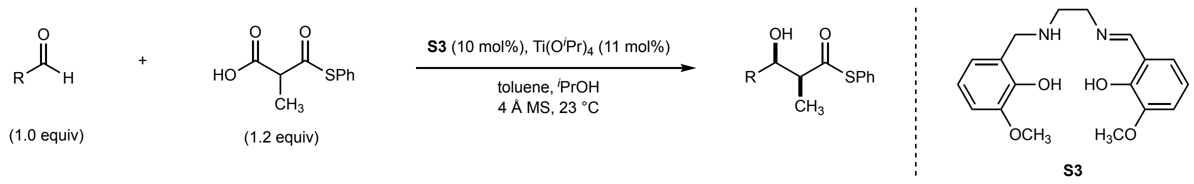

To a suspension of the achiral salen **S3** (0.10 mmol, 10 mol%) and activated molecular sieves (4 Å, 200 mg) in toluene (0.10 M) was added 2-propanol (1.00 mmol, 1.00 equiv) followed by a solution of titanium (IV) isopropoxide (0.50 M in toluene; 0.11 mmol, 11 mol%). The catalyst mixture was stirred for 1 h at 23 °C. Malonic acid half thioester (1.20 mmol, 1.20 equiv) was added to the catalyst mixture in one portion. The resulting red solution was stirred for 15 min at 23 °C followed by addition of the aldehyde (1.00 mmol, 1 equiv). The reaction mixture was stirred at 23 °C until consumption of the aldehyde was observed. The solution gradually turned to a yellow color over the course of the reaction. The product mixture was filtered through celite and rinsed with ethyl acetate. The filtrate was concentrated and the residue was purified by column chromatography.

General method A for the *syn*-selective aldol reaction:

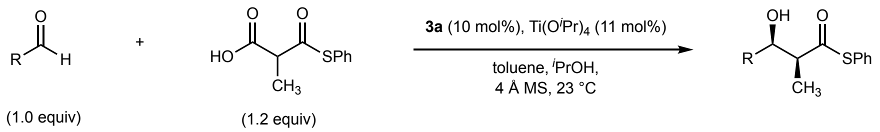

To a suspension of (*S,S*)-salen **3a** (0.10 mmol, 10 mol%) and activated molecular sieves (4 Å, 200 mg) in toluene (0.10 M) was added 2-propanol (1.00 mmol, 1.00 equiv) followed by a solution of titanium (IV) isopropoxide (0.50 M in toluene; 0.11 mmol, 11 mol%). The catalyst mixture was stirred for 1 h at 23 °C. Malonic acid half thioester (1.20 mmol, 1.20 equiv) was added to the catalyst mixture in one portion. The resulting red solution was stirred for 15 min at 23 °C followed by addition of the aldehyde (1.00 mmol, 1 equiv). The reaction mixture was stirred at 23 °C until consumption of the aldehyde was observed. The solution gradually turned to a yellow color over the course of the reaction. The product mixture was filtered through a celite and rinsed with ethyl acetate. The filtrate was concentrated and the residue was purified by column chromatography. **For deviations from the general procedure (for example catalyst loading, reaction concentration, time or scale) see the corresponding entries.*

General method B for the *anti*-selective aldol reaction:

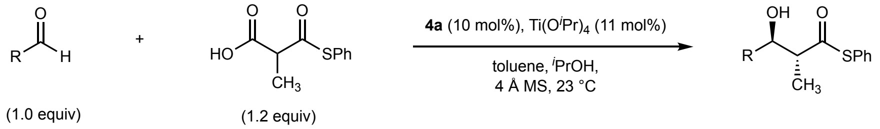

To a suspension of (*S,S*)-salalen **4a** (0.10 mmol, 10 mol%) and activated molecular sieves (4 Å, 200 mg) in toluene (0.10 M) was added 2-propanol (1.00 mmol, 1.00 equiv) followed by a solution of titanium (IV) isopropoxide (0.50 M in toluene; 0.11 mmol, 11 mol%). The catalyst mixture was stirred for 1 h at 23 °C. Malonic acid half thioester (1.20 mmol, 1.20 equiv)

was added to the catalyst mixture in one portion. The resulting red solution was stirred for 15 min at 23 °C followed by addition of the aldehyde (1.00 mmol, 1 equiv). The reaction mixture was stirred at 23 °C until consumption of the aldehyde was observed. The solution gradually turned to a yellow color over the course of the reaction. The product mixture was filtered through celite and rinsed with ethyl acetate. The filtrate was concentrated and the residue was purified by column chromatography. **For deviations from the general procedure (for example catalyst loading, reaction concentration, time or scale) see the corresponding entries.*

General method C for the synthesis of symmetrical salen ligands:

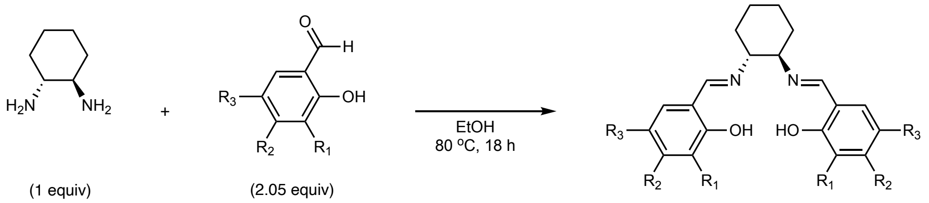

3(**a**–**r**)

To a solution of (1*R*,2*R*)-cyclohexane-1,2-diamine (1 equiv) in ethanol (0.10 M) was added the aldehyde (2.05 equiv) in one portion. The resulting solution was stirred for 12 h at 80 °C. The mixture was concentrated to half the volume and cooled to 0 °C. After 2 h the resulting heterogenous solution was filtered through a sinter funnel and the crystalline precipitate was washed with cold ethanol. After drying under high vacuum for 16 h the product was obtained as a crystalline solid. If the product was not sufficiently pure by ^{1}H-NMR analysis it was further purified by column chromatography. **The (S,S)-salen ligands were prepared accordingly using (1S,2S)-cyclohexane-1,2-diamine.*

General method D for the synthesis of salalen ligands:

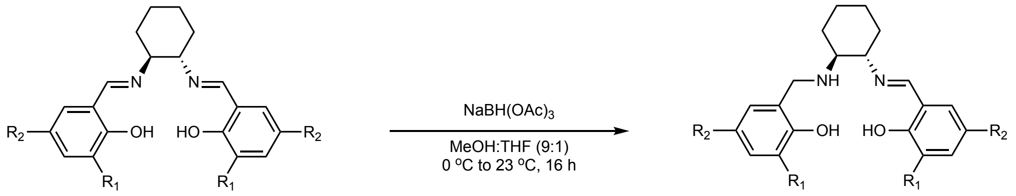

3 **4**(**a**-**c**)

To a stirred solution of (*S*,*S*)-salen **3** (1 equiv) in a mixture of methanol:tetrahydrofuran (9:1, 0.10 M) at 0 °C was added sodium triacetoxyborohydride (1.10 equiv). The reaction was slowly allowed to warm to 23 °C and stirred for 16 h at this temperature. The reaction mixture was diluted with dichloromethane and neutralized with saturated aqueous ammonium chloride solution. The organic layer was separated and the aqueous layer was washed twice with dichloromethane. The combined organic layer was washed twice with saturated sodium bicarbonate solution, the organic layer was dried over anhydrous sodium sulfate, concentrated and the residue was purified by flash column chromatography to obtain salalen **4**. **For deviations from the general procedure (for example catalyst loading, reaction concentration, time or scale) see the corresponding entries.*

General method E for the synthesis of malonic acid half thioesters (MAHTs):

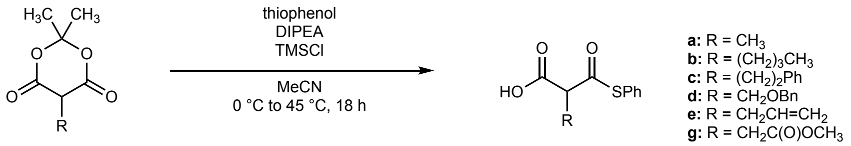

S12(**a-e,g**) **2**(**a-e,g**)

To a suspension of the meldrum's acid derivatives (1 equiv) in acetonitrile (0.40 M) at 0 °C was added *N*,*N*-diisopropylethylamine (1.10 equiv) and trimethylsilyl chloride (1.10 equiv) sequentially. The mixture was stirred for 5 min before the addition of thiophenol (1.05 equiv). The reaction mixture was heated to 45 °C and stirred for 18 h at this temperature. The reaction mixture was cooled to 23 °C and diluted with ice-cold water and stirred for 10 mins. The product mixture was diluted with ethyl acetate and the organic layer was separated. The aqueous layer was washed twice with ethyl acetate. The combined organic extracts were dried over anhydrous sodium sulfate, concentrated and the residue was purified by column chromatography.

Synthetic procedures for the *syn*- and *anti*-aldol reactions:

S-phenyl (2S,3S)-3-hydroxy-2-methyl-3-(4-nitrophenyl)propanethioate (*S*,*S*)-**5a**

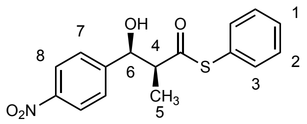

Following general method **A**, (*S*,*S*)-**5a** was prepared from 4-nitrobenzaldehyde (151 mg, 1.00 mmol, 1 equiv) and MAHT **2** (252 mg, 1.20 mmol, 1.20 equiv). The reaction was stirred for 24 h. The residue was purified by column chromatography (eluting with 5% ethyl acetate–hexane initially, grading to 40% ethyl acetate–hexane). The product was obtained as a white solid (360 mg, 97%, 95:5 d.r. (syn:anti)).

Major diastereomer:

^{1}H NMR (500 MHz, $CDCl_3$) δ 8.18 (d, *J* = 8.8 Hz, 2H, H_8), 7.51 (d, *J* = 8.8 Hz, 2H, H_7), 7.42 – 7.40 (m, 3H, $H_{1,2}$), 7.37 – 7.33 (m, 2H, H_3), 5.22 (d, *J* = 4.1 Hz, 1H, H_6), 3.34 (bs, 1H, OH), 3.07 – 3.02 (m, 1H, H_4), 1.23 (d, *J* = 7.1 Hz, 3H, H_5).

^{13}C NMR (126 MHz, $CDCl_3$) δ 201.7 (C), 148.6 (C), 147.3 (C), 134.4 (2 × CH), 129.8 (CH), 129.33 (2 × CH), 127.0 (2 × CH), 126.6 (C), 123.5 (2 × CH), 72.8 (CH), 54.3 (CH), 11.5 (CH_3).

Minor diastereomer (detectable non-overlapping resonances):

^{1}H NMR (500 MHz, $CDCl_3$) δ 4.94 (d, *J* = 7.6 Hz, 1H, H_6), 1.15 (d, *J* = 7.1 Hz, 3H, H_5).

^{13}C NMR (126 MHz, $CDCl_3$) δ 201.4 (C), 148.9 (C), 147.5 (C), 134.3 (2 × CH), 129.28 (CH), 127.5 (2 × CH), 123.6 (2 × CH), 75.4 (CH), 54.7 (CH), 15.3 (CH_3).

HPLC (AD-H, *n*-hexane/^{i}PrOH=90:10, 1.0 mL/min, 298 K, 254 nm): t_R(syn, minor) = 12.4 min, t_R(syn, major) = 14.2 min, t_R(anti, minor) = 20.3 min, t_R(anti, major) = 33.7 min, d.r. (syn:anti) = 95:5, e.r.(syn) = 99:1 (98% *ee*), e.r.(anti) = 95:5 (90% *ee*).

HRMS-CI (m/z): $[M + Na]^+$ calcd for $C_{16}H_{15}NO_4SNa$, 340.0619; found, 340.0614.

R_f = 0.40 (20% ethyl acetate-hexane; UV).

The absolute stereochemistry for (*S*,*S*)-**5a** was confirmed by x-ray crystallography (see **Fig. 1c**). The crystal was grown by a slow evaporation of dichloromethane.

S-phenyl (2R,3R)-3-hydroxy-2-methyl-3-(4-nitrophenyl)propanethioate (*R*,*R*)-**5a**

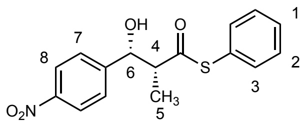

Following general method **A**, (*R*,*R*)-**5a** was prepared from 4-nitrobenzaldehyde (151 mg, 1.00 mmol, 1 equiv) and MAHT **2** (252 mg, 1.20 mmol, 1.20 equiv). (*R,R*)-Salen **3a** (38 mg, 0.10 mmol, 10 mol%) was used as ligand. The reaction was stirred for 24 h. The residue was purified by column chromatography (eluting with 5% ethyl acetate–hexane initially, grading to 40% ethyl acetate–hexane). The product was obtained as a white solid (315 mg, 99%, 94:6 d.r. (syn:anti)).

Major diastereomer:

^{1}H NMR (500 MHz, $CDCl_3$) δ 8.23 (d, *J* = 2.1 Hz, 2H, H_8), 7.55 (d, 2H, H_7), 7.49 – 7.40 (m, 3H, $H_{1,2}$), 7.40 – 7.32 (m, 2H, H_3), 5.27 (d, *J* = 3.6 Hz, 1H, H_6), 3.06 (d, *J* = 2.6 Hz, OH), 3.09 – 3.02 (m, 1H, H_4), 1.23 (d, *J* = 1.6 Hz, 3H, H_5).

^{13}C NMR (126 MHz, $CDCl_3$) δ 202.2 (C), 148.4 (C), 147.5 (C), 134.6 (2 × CH), 130.0 (CH), 129.52 (2 × CH), 127.1 (2 × CH), 126.6 (C), 123.7 (2 × CH), 72.8 (CH), 54.3 (CH), 11.5 (CH_3).

Minor diastereomer (detectable non-overlapping resonances):

^{1}H NMR (500 MHz, $CDCl_3$) δ 4.96 (d, *J* = 7.2 Hz, 1H, H_6), 3.16 – 3.09 (m, 1H, H_4).

^{13}C NMR (126 MHz, $CDCl_3$) δ 201.7 (C), 148.9 (C), 147.8 (C), 134.5 (2 × CH), 129.9 (C), 129.46 (2 × CH), 127.5 (2 × CH), 126.8 (C), 123.8 (2 × CH), 75.7 (CH), 54.8 (CH), 15.6 (CH_3).

HPLC (AD-H, *n*-hexane/^{i}PrOH=90:10, 1.0 mL/min, 298 K, 254 nm): t_R(syn, major) = 12.4 min, t_R(syn, minor) = 14.2 min, t_R(anti, major) = 20.4 min, t_R(anti, minor) = 34.8 min, d.r. (syn:anti) = 94:6, e.r.(syn) = 99:1 (99% *ee*), e.r.(anti) = 96:4 (92% *ee*).

HRMS-CI (m/z): $[M + Na]^+$ calcd for $C_{16}H_{15}NO_4SNa$, 340.0619; found, 340.0615.
R_f = 0.40 (20% ethyl acetate-hexane; UV).
The absolute stereochemistry for (*R*,*R*)-**5a** was confirmed by x-ray crystallography (see **Fig. 1c**). The crystal was grown by a slow evaporation of dichloromethane.

S-phenyl (2R,3S)-3-hydroxy-2-methyl-3-(4-nitrophenyl)propanethioate (*S*,*R*)-**6a**

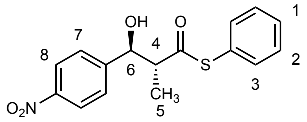

Following general method **B**, (*S*,*R*)-**6a** was prepared from 4-nitrobenzaldehyde (151 mg, 1.00 mmol, 1 equiv) and MAHT **2** (252 mg, 1.20 mmol, 1.20 equiv). The reaction was stirred for 24 h. The residue was purified by column chromatography (eluting with 5% ethyl acetate–hexane initially, grading to 40% ethyl acetate–hexane). The product was obtained as a white solid (312 mg, 98%, 9:91 d.r. (syn:anti)).
Major diastereomer:
^{1}H NMR (500 MHz, $CDCl_3$) δ 8.27 – 8.20 (m, 2H, H_8), 7.60 – 7.49 (m, 2H, H_7), 7.48 – 7.40 (m, 3H, $H_{1,2/3}$), 7.40 – 7.32 (m, 2H, $H_{2/3}$), 4.96 (dd, *J* = 7.2, 5.6 Hz, 1H, H_6), 3.18 – 3.03 (m, 2H, $H_{4,OH}$), 1.23 (d, *J* = 7.2 Hz, 3H, H_5).
^{13}C NMR (126 MHz, $CDCl_3$) δ 201.7 (C), 148.9 (C), 147.8 (C), 134.5 (2 × CH), 130.0 (CH), 129.5 (2 × CH), 127.5 (2 × CH), 126.8 (C), 123.9 (2 × CH), 75.7 (CH), 54.8 (CH), 15.6 (CH_3).
Minor diastereomer (detectable non-overlapping resonances):
^{1}H NMR (500 MHz, $CDCl_3$) δ 5.29 (dd, *J* = 3.1, 3.1 Hz, 1H, H_6).
^{13}C NMR (126 MHz, $CDCl_3$) δ 134.6 (2 × CH), 130.1 (CH), 129.6 (2 × CH), 127.1 (2 × CH), 123.7 (2 × CH), 72.8 (CH), 54.2 (CH), 11.4 (CH_3).
HPLC (AD-H, *n*-hexane/^{i}PrOH=90:10, 1.0 mL/min, 298 K, 254 nm): t_R(syn, major) = 12.5 min, t_R(syn, minor) = 14.3 min, t_R(anti, major) = 20.4 min, t_R(anti, minor) = 33.7 min, d.r. (syn:anti) = 9:91, e.r.(syn) = 68:32 (36% *ee*), e.r.(anti) = 98:2 (97% *ee*).
HRMS-CI (m/z): $[M + Na]^+$ calcd for $C_{16}H_{15}NO_4SNa$, 340.0619; found, 340.0599.
R_f = 0.40 (20% ethyl acetate-hexane; UV).
The absolute stereochemistry for (*S*,*R*)-**6a** was confirmed by x-ray crystallography (see **Fig. 1c**). The crystal was grown by a slow evaporation of dichloromethane.

S-phenyl (2S,3R)-3-hydroxy-2-methyl-3-(4-nitrophenyl)propanethioate (*R*,*S*)-**6a**

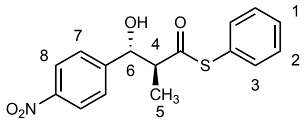

Following general method **B**, (*R*,*S*)-**6a** was prepared from 4-nitrobenzaldehyde (151 mg, 1.00 mmol, 1 equiv) and MAHT **2** (252 mg, 1.20 mmol, 1.20 equiv). (*R,R*)-Salalen **3b** (38 mg, 0.10 mmol, 10 mol%) was used as ligand. The reaction was stirred for 24 h. The residue was purified by column chromatography (eluting with 5% ethyl acetate–hexane initially, grading to 40% ethyl acetate–hexane). The product was obtained as a white solid (300 mg, 94%, 11:89 d.r. (syn:anti)).
Major diastereomer:
^{1}H NMR (500 MHz, $CDCl_3$) δ 8.33 – 8.19 (m, 2H, H_8), 7.59 – 7.48 (m, 2H, H_7), 7.48 – 7.37 (m, 3H, $H_{1,2/3}$), 7.40 – 7.31 (m, 2H, $H_{2/3}$), 4.96 (dd, *J* = 7.2, 5.6 Hz, 1H, H_6), 3.18 – 3.10 (m, 1H, H_4), 3.06 (d, *J* = 5.6 Hz, 1H, OH), 1.23 (d, *J* = 7.2 Hz, 3H, H_5).
^{13}C NMR (126 MHz, $CDCl_3$) δ 201.8 (C), 148.9 (C), 147.9 (C), 134.55 (2 × CH), 130.0 (CH), 129.6 (2 × CH), 127.51 (2 × CH), 126.8 (C), 123.9 (2 × CH), 75.8 (CH), 54.8 (CH), 15.7 (CH_3).
Minor diastereomer (detectable non-overlapping resonances):
^{1}H NMR (500 MHz, $CDCl_3$) δ 5.31 – 5.27 (m, 1H, H_6), 3.08 (d, *J* = 2.6 Hz, 1H, OH).
^{13}C NMR (126 MHz, $CDCl_3$) δ 134.62 (2 × CH), 129.6 (2 × CH), 127.1 (2 × CH), 123.7 (2 × CH), 72.8 (CH), 54.2 (CH), 11.4 (CH_3).

HPLC (AD-H, *n*-hexane/^{i}PrOH=90:10, 1.0 mL/min, 298 K, 254 nm): t_R(syn, minor) = 12.4 min, t_R(syn, major) = 14.2 min, t_R(anti, minor) = 20.3 min, t_R(anti, major) = 34.8 min, d.r. (syn:anti) = 11:89, e.r.(syn) = 66:34 (32% *ee*), e.r.(anti) = 97:3 (94% *ee*).
HRMS-CI (m/z): $[M + Na]^+$ calcd for $C_{16}H_{15}NO_4SNa$, 340.0619; found, 340.0598.
R_f = 0.40 (20% ethyl acetate-hexane; UV).
The absolute stereochemistry for (*S*,*R*)-**6a** was confirmed by x-ray crystallography (see **Fig. 1c**). The crystal was grown by a slow evaporation of dichloromethane.

S-phenyl (2S,3S)-3-(4-fluorophenyl)-3-hydroxy-2-methylpropanethioate **5b**

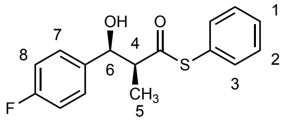

Following general method **A**, **5b** was prepared from 4-fluorobenzaldehyde (211 µL, 2.00 mmol, 1 equiv) and MAHT **2** (505 mg, 2.40 mmol, 1.20 equiv) in toluene (5.00 mL, 0.40 M). The reaction was stirred for 48 h. The residue was purified by column chromatography (eluting with 5% ethyl acetate–hexane initially, grading to 40% ethyl acetate–hexane). The product was obtained as a colorless liquid (508 mg, 88%, 95:5 d.r. (syn:anti)).
Major diastereomer:
^{1}H NMR (500 MHz, $CDCl_3$) δ 7.45 – 7.38 (m, 3H, $H_{1,2}$), 7.36 – 7.30 (m, 4H, $H_{3,7/8}$), 7.10 – 7.03 (m, 2H, $H_{7/8}$), 5.09 (d, *J* = 4.5 Hz, 1H, H_6), 3.02 (qd, *J* = 7.1, 4.5 Hz, 1H, H_4), 2.79 (bs, 1H, OH), 1.27 (d, *J* = 7.1 Hz, 3H, H_5).
^{13}C NMR (126 MHz, $CDCl_3$) δ 202.1 (C), 162.4 (d, *J* = 245.7 Hz, CF), 136.9 (d, *J* = 3.1 Hz, C), 134.6 (2 × CH), 129.8 (CH), 129.40 (2 × CH), 127.9 (d, *J* = 8.1 Hz, 2 × CH), 127.1 (C), 115.3 (d, *J* = 21.4 Hz, 2 × CH), 73.4 (CH), 55.0 (CH), 11.9 (CH_3).
^{19}F NMR (470 MHz, $CDCl_3$) δ -114.6 – -114.8 (m).
Minor diastereomer (detectable non-overlapping resonances):
^{1}H NMR (500 MHz, $CDCl_3$) δ 4.83 (d, *J* = 8.2 Hz, 1H, H_6), 1.11 (d, J = 7.1 Hz, 3H, H_5).
^{13}C NMR (126 MHz, $CDCl_3$) δ 201.7 (C), 129.6 (CH), 129.37 (2 × CH), 128.3 (d, *J* = 8.1 Hz, 2 × CH), 115.5 (d, *J* = 21.7 Hz, 2 × CH), 75.9 (CH), 55.3 (CH), 15.4 (CH_3).
^{19}F NMR (470 MHz, $CDCl_3$) δ -113.9 – -114.0 (m).
HPLC (AD-H, n-hexane/^{i}PrOH=90:10, 1.0 mL/min, 298 K, 254 nm): t_R(syn, minor) = 10.4 min, t_R(syn, major) = 11.1 min, t_R(anti, major) = 12.5 min, t_R(anti, minor) = 20.2 min, d.r. (syn:anti) = 95:5, e.r.(syn) = 99:1 (98% *ee*), e.r.(anti) = 93:7 (86% *ee*).
HRMS-CI (m/z): $[M + Na]^+$ calcd for $C_{16}H_{15}FO_2SNa$, 313.0674; found, 313.0656.
R_f = 0.38 (20% ethyl acetate-hexane; UV).

S-phenyl (2R,3S)-3-(4-fluorophenyl)-3-hydroxy-2-methylpropanethioate **6b**

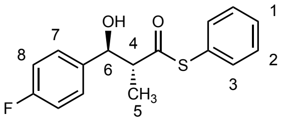

Following general method **B**, **6b** was prepared from 4-fluorobenzaldehyde (211 µL, 2.00 mmol, 1 equiv) and MAHT **2** (505 mg, 2.40 mmol, 1.20 equiv) in toluene (5.00 mL, 0.40 M). The reaction was stirred for 48 h. The residue was purified by column chromatography (eluting with 5% ethyl acetate–hexane initially, grading to 40% ethyl acetate–hexane). The product was obtained as a white solid (420 mg, 72%, 17:83 d.r. (syn:anti)).
Major diastereomer:
^{1}H NMR (500 MHz, $CDCl_3$) δ 7.45 – 7.39 (m, 5H, H_{1-3}), 7.37 – 7.30 (m, 2H, $H_{7/8}$), 7.10 – 7.04 (m, 2H, $H_{7/8}$), 4.84 (d, *J* = 8.1 Hz, 1H, H_6), 3.08 (dq, *J* = 8.1, 7.1 Hz, 1H, H_4), 1.12 (d, *J* = 7.1 Hz, 3H, H_5).
^{13}C NMR (126 MHz, $CDCl_3$) δ 201.8 (C), 162.7 (d, *J* = 246.6 Hz, CF), 137.4 (d, *J* = 3.2 Hz, C), 134.6 (2 × CH), 129.7 (CH), 129.40 (2 × CH), 128.4 (d, *J* = 8.2 Hz, 2 × CH), 127.3 (C), 115.6 (d, *J* = 21.5 Hz, 2 × CH), 76.1 (CH), 55.4 (CH), 15.6 (CH_3).
^{19}F NMR (470 MHz, $CDCl_3$) δ -113.9 – -114.1 (m).
Minor diastereomer (detectable non-overlapping resonances):

^{1}H NMR (500 MHz, $CDCl_3$) δ 5.12 (d, *J* = 4.3 Hz, 1H, H_6), 3.02 (qd, *J* = 7.0, 4.3 Hz, 1H, H_4), 1.26 (d, *J* = 7.0 Hz, 1H, H_5).

^{13}C NMR (126 MHz, $CDCl_3$) δ 202.2 (C), 162.4 (d, *J* = 245.7 Hz, CF), 137.0 (d, *J* = 3.2 Hz, C), 129.8 (CH), 129.44 (2 × CH), 127.9 (d, *J* = 8.1 Hz, 2 × CH), 127.1 (C), 115.4 (d, *J* = 21.4 Hz, 2 × CH), 73.4 (CH), 55.0 (CH), 11.9 (CH_3).

^{19}F NMR (470 MHz, $CDCl_3$) δ -114.6 – -114.9 (m).

HPLC (AD-H, *n*-hexane/^{i}PrOH=90:10, 1.0 mL/min, 298 K, 254 nm): t_R(syn, major) = 10.5 min, t_R(syn, minor) = 11.5 min, t_R(anti, minor) = 12.5 min, t_R(anti, major) = 20.2 min, d.r. (syn:anti) = 17:83, e.r.(syn) = 75:25 (50% *ee*), e.r.(anti) = 98:2 (96% *ee*).

HRMS-CI (m/z): $[M + Na]^+$ calcd for $C_{16}H_{15}FO_2SNa$, 313.0674; found, 313.0654.

R_f = 0.38 (20% ethyl acetate-hexane; UV).

S-phenyl (2S,3S)-3-(4-chlorophenyl)-3-hydroxy-2-methylpropanethioate **5c**

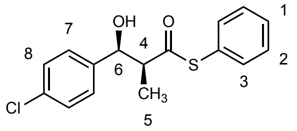

Following general method **A**, **5c** was prepared from 4-chlorobenzaldehyde (140 mg, 1.00 mmol, 1 equiv) and MAHT **2** (252 mg, 1.20 mmol, 1.20 equiv). The reaction was stirred for 48 h. The residue was purified by column chromatography (eluting with 5% ethyl acetate–hexane initially, grading to 40% ethyl acetate–hexane). The product was obtained as a white solid (250 mg, 82%, 95:5 d.r. (syn:anti)).

Major diastereomer:

^{1}H NMR (500 MHz, $CDCl_3$) δ 7.46 – 7.32 (m, 7H, $H_{1\text{-}3,8}$), 7.31 (d, *J* = 8.6 Hz, 2H, H_7), 5.16– 5.10 (m, 1H, H_6), 3.04 – 2.99 (m, 1H, H_4), 2.84 (s, 1H, OH), 1.24 (d, *J* = 7.3 Hz, 3H, H_5).

^{13}C NMR (126 MHz, $CDCl_3$) δ 201.8 (C), 139.7 (C), 134.5 (2 × CH), 133.4 (C), 129.7 (CH), 129.31 (2 × CH), 128.5 (2 × CH), 127.6 (2 × CH), 126.9 (C), 73.3 (CH), 54.8 (CH), 11.8 (CH_3).

Minor diastereomer (detectable non-overlapping resonances):

^{1}H NMR (500 MHz, $CDCl_3$) δ 4.83 (d, *J* = 7.9 Hz, 1H, H_6), 1.14 (d, *J* = 7.3 Hz, 1H, H_5).

^{13}C NMR (126 MHz, $CDCl_3$) δ 140.1 (C), 129.27 (2 × CH), 128.7 (2 × CH), 128.0 (2 × CH), 75.8 (CH), 55.1 (CH), 15.4 (CH_3).

HPLC (AD-H, *n*-hexane/^{i}PrOH=90:10, 1.0 mL/min, 298 K, 254 nm): t_R(syn, minor) = 7.8 min, t_R(syn, major) = 8.9 min, t_R(anti, minor) = 13.0 min, t_R(anti, major) = 13.8 min, d.r. (syn:anti) = 95:5, e.r.(syn) = 98:2 (97% *ee*), e.r.(anti) = 92:8 (84% *ee*).

HRMS-CI (m/z): $[M + Na]^+$ calcd for $C_{16}H_{15}ClO_2SNa$, 329.0379; found, 329.0376.

R_f = 0.50 (20% ethyl acetate-hexane; UV).

S-phenyl (2R,3S)-3-(4-chlorophenyl)-3-hydroxy-2-methylpropanethioate **6c**

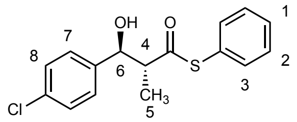

Following general method **B**, **6c** was prepared from 4-chlorobenzaldehyde (140 mg, 1.00 mmol, 1 equiv) and MAHT **2** (252 mg, 1.20 mmol, 1.20 equiv). The reaction was stirred for 48 h. The residue was purified by column chromatography (eluting with 5% ethyl acetate–hexane initially, grading to 40% ethyl acetate–hexane). The product was obtained as a white solid (262 mg, 85%, 15:85 d.r. (syn:anti)).

Major diastereomer:

^{1}H NMR (500 MHz, $CDCl_3$) δ 7.45 – 7.38 (m, 5H, $H_{1\text{-}3}$), 7.37 – 7.33 (m, 2H, H_8), 7.31 – 7.27 (m, 2H, H_7), 4.82 (dd, *J* = 8.0, 4.8 Hz, 1H, H_6), 3.13 – 3.04 (m, 1H, H_4), 2.80 (d, *J* = 4.8 Hz, 1H, OH), 1.13 (d, *J* = 7.1 Hz, 3H, H_5).

^{13}C NMR (126 MHz, $CDCl_3$) δ 201.8 (C), 140.1 (C), 134.59 (2 × CH), 134.0 (C), 129.7 (CH), 129.40 (2 × CH), 128.9 (2 × CH), 128.1 (2 × CH), 127.3 (C), 76.0 (CH), 55.2 (CH), 15.6 (CH_3).

Minor diastereomer (detectable non-overlapping resonances):

^{1}H NMR (500 MHz, $CDCl_3$) δ 5.12 (dd, *J* = 4.1, 2.6 Hz, 1H, H_6), 3.01 (qd, *J* = 7.1, 4.1 Hz, 1H, H_4), 2.87 (d, *J* = 2.6 Hz, 1H, OH), 1.24 (d, *J* = 7.1 Hz, 3H, H_5).

^{13}C NMR (126 MHz, $CDCl_3$) δ 202.2 (C), 139.7 (C), 134.61 (2 × CH), 133.5 (C), 129.8 (CH), 129.44 (2 × CH), 128.6 (2 × CH), 127.6 (2 × CH), 127.0 (C), 73.2 (CH), 54.8 (CH), 11.7 (CH_3).
HPLC (AD-H, *n*-hexane/^{i}PrOH=90:10, 1.0 mL/min, 298 K, 254 nm): t_R(syn, minor) = 7.8 min, t_R(syn, major) = 8.9 min, t_R(anti, minor) = 13.0 min, t_R(anti, major) = 13.8 min, d.r. (syn:anti) = 15:85, e.r.(syn) = 73:27 (46% *ee*), e.r.(anti) = 98:2 (96% *ee*).
HRMS-CI (m/z): $[M + Na]^+$ calcd for $C_{16}H_{15}ClO_2SNa$, 329.0379; found, 329.0358.
R_f = 0.50 (20% ethyl acetate-hexane; UV).
The absolute stereochemistry for **6c** was confirmed by x-ray crystallography (see **fig. S4**). The crystal was grown by a slow evaporation of dichloromethane.

S-phenyl (2S,3S)-3-(4-bromophenyl)-3-hydroxy-2-methylpropanethioate **5d**

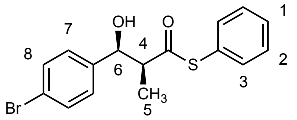

Following general method **A**, **5d** was prepared from 4-bromobenzaldehyde (185 mg, 1.00 mmol, 1 equiv) and MAHT **2** (252 mg, 1.20 mmol, 1.20 equiv). The reaction was stirred for 48 h. The residue was purified by column chromatography (eluting with 5% ethyl acetate–hexane initially, grading to 40% ethyl acetate–hexane). The product was obtained as a white solid (298 mg, 85%, 94:6 d.r. (syn:anti)).
Major diastereomer:
^1H NMR (500 MHz, $CDCl_3$) δ 7.52 (d, *J* = 8.2 Hz, 2H, H_8), 7.47 – 7.42 (m, 3H, $H_{1,2}$), 7.40 – 7.37 (m, 2H, H_3), 7.25 (d, *J* = 8.2 Hz, 2H, H_7), 5.09 (d, *J* = 4.4 Hz, 1H, H_6), 3.06 – 3.00 (m, 2H, $H_{4, OH}$), 1.28 (d, *J* = 7.1 Hz, 3H, H_5).
^{13}C NMR (126 MHz, $CDCl_3$) δ 201.9 (C), 140.2 (C), 134.47 (2 × CH), 131.4 (2 × CH), 129.7 (CH), 129.31 (2 × CH), 127.9 (2 × CH), 126.9 (C), 121.5 (C), 73.2 (CH), 54.7 (CH), 11.7 (CH_3).
Minor diastereomer (detectable non-overlapping resonances):
^1H NMR (500 MHz, $CDCl_3$) δ 4.80 (d, J = 8.1 Hz, 1H, H_6), 1.13 (d, J = 7.2 Hz, 3H, H_5).
^{13}C NMR (126 MHz, $CDCl_3$) δ 201.6 (C), 140.6 (C), 134.45 (2 × CH), 131.6 (2 × CH), 129.28 (2 × CH), 128.4 (2 × CH), 122.0 (C), 75.9 (CH), 55.1 (CH), 15.4 (CH_3),
HPLC (AD-H, *n*-hexane/^{i}PrOH=90:10, 1.0 mL/min, 298 K, 254 nm): t_R(syn, minor) = 8.2 min, t_R(syn, major) = 9.6 min, t_R(anti, minor) = 13.9 min, t_R(anti, major) = 15.5 min, d.r. (syn:anti) = 94:6, e.r.(syn) = 98:2 (96% *ee*), e.r.(anti) = 90:10 (80% *ee*).
HRMS-CI (m/z): $[M + Na]^+$ calcd for $C_{16}H_{15}BrO_2SNa$, 372.9874; found, 372.9846.
R_f = 0.38 (20% ethyl acetate-hexane; UV).

S-phenyl (2R,3S)-3-(4-bromophenyl)-3-hydroxy-2-methylpropanethioate **6d**

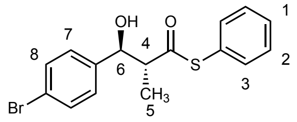

Following general method **B**, **6d** was prepared from 4-bromobenzaldehyde (185 mg, 1.00 mmol, 1 equiv) and MAHT **2** (252 mg, 1.20 mmol, 1.20 equiv). The reaction was stirred for 48 h. The residue was purified by column chromatography (eluting with 5% ethyl acetate–hexane initially, grading to 40% ethyl acetate–hexane). The product was obtained as a white solid (277 mg, 79%, 13:87 d.r. (syn:anti)).
Major diastereomer:
^1H NMR (500 MHz, $CDCl_3$) δ 7.54 – 7.48 (m, 2H, H_8), 7.46 – 7.34 (m, 5H, H_{1-3}), 7.27 – 7.21 (m, 2H, H_7), 4.81 (dd, *J* = 7.9, 4.9 Hz, 1H, H_6), 3.12 – 3.03 (m, 1H, H_4), 2.78 (d, *J* = 4.9 Hz, 1H, OH), 1.14 (d, *J* = 7.1 Hz, 3H, H_5).
^{13}C NMR (126 MHz, $CDCl_3$) δ 201.8 (C), 140.7 (C), 134.61 (2 × CH), 131.8 (2 × CH), 129.8 (CH), 129.4 (2 × CH), 128.4 (2 × CH), 127.3 (C), 122.2 (C), 76.1 (CH), 55.2 (CH), 15.6 (CH_3).
Minor diastereomer (detectable non-overlapping resonances):

^{1}H NMR (500 MHz, $CDCl_3$) δ 5.14 – 5.10 (m, 1H, H_6), 3.01 (qd, J = 7.1, 3.9 Hz, 1H, H_4), 2.85 (d, J = 2.7 Hz, 1H, OH), 1.24 (d, J = 7.1 Hz, 3H, H_5).

^{13}C NMR (126 MHz, $CDCl_3$) δ 140.2 (C), 134.64 (2 × CH), 131.6 (2 × CH), 129.9 (CH), 129.5 (2 × CH), 128.0 (2 × CH), 121.7 (C), 73.2 (CH), 54.7 (CH), 11.6 (CH_3).

HPLC (AD-H, *n*-hexane/^{i}PrOH=90:10, 1.0 mL/min, 298 K, 254 nm): t_R(syn, major) = 8.1 min, t_R(syn, minor) = 9.5 min, t_R(anti, major) = 13.9 min, t_R(anti, minor) = 15.5 min, d.r. (syn:anti) = 13/87, e.r.(syn) = 76:24 (52% *ee*), e.r.(anti) = 98:2 (97% *ee*).

HRMS-CI (m/z): $[M + Na]^+$ calcd for $C_{16}H_{15}BrO_2SNa$, 372.9874; found, 372.9869.

R_f = 0.38 (20% ethyl acetate-hexane; UV).

S-phenyl (2S,3S)-3-(4-cyanophenyl)-3-hydroxy-2-methylpropanethioate **5e**

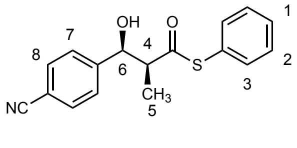

Following general method **A**, **5e** was prepared from 4-formylbenzonitrile (131 mg, 1.00 mmol, 1 equiv) and MAHT **2** (252 mg, 1.20 mmol, 1.20 equiv). The reaction was stirred for 24 h. The residue was purified by column chromatography (eluting with 5% ethyl acetate–hexane initially, grading to 40% ethyl acetate–hexane). The product was obtained as a white solid (293 mg, 99%, 92:8 d.r. (syn:anti)).

Major diastereomer:

^{1}H NMR (500 MHz, $CDCl_3$) δ 7.64 (d, J = 8.0 Hz, 2H, H_8), 7.48 (d, J = 8.0 Hz, 2H, H_7), 7.45-7.34 (m, 5H, H_{1-3}), 5.18 (d, J = 4.2 Hz, 1H, H_6), 3.33 (bs, 1H, OH), 3.08 – 3.00 (m, 1H, H_4), 1.25 (d, J = 7.1 Hz, 3H, H_5),

^{13}C NMR (126 MHz, $CDCl_3$) δ 201.6 (C), 146.7 (C), 134.42 (2 × CH), 132.1 (2 × CH), 129.8 (CH), 129.33 (2 × CH), 126.9 (2 × CH), 126.7 (C), 118.8 (C), 111.2 (CN), 73.0 (CH), 54.5 (CH), 11.6 (CH_3).

Minor diastereomer (detectable non-overlapping resonances):

^{1}H NMR (500 MHz, $CDCl_3$) δ 4.90 (d, J = 7.7 Hz, 1H), 3.13 – 3.09 (m, 1H, H_4), 1.15 (d, J = 7.1 Hz, 3H, H_5).

^{13}C NMR (126 MHz, $CDCl_3$) δ 201.3 (C), 147.0 (C), 134.37 (2 × CH), 132.3 (2 × CH), 129.29 (2 × CH), 127.4 (2 × CH), 75.6 (CH), 54.8 (CH), 15.3 (CH_3).

HPLC (AD-H, *n*-hexane/^{i}PrOH=90:10, 1.0 mL/min, 298 K, 254 nm): t_R(syn, minor) = 12.7 min, t_R(syn, major) = 14.9 min, t_R(anti, minor) = 20.8 min, t_R(anti, major) = 24.9 min, d.r. (syn:anti) = 92:8, e.r.(syn) = 99:1 (98% *ee*), e.r.(anti) = 96:4 (92% *ee*).

HRMS-CI (m/z): $[M + Na]^+$ calcd for $C_{17}H_{15}NO_2SNa$, 320.0721.1473; found, 320.0714.

R_f = 0.30 (20% ethyl acetate-hexane; UV).

S-phenyl (2R,3S)-3-(4-cyanophenyl)-3-hydroxy-2-methylpropanethioate **6e**

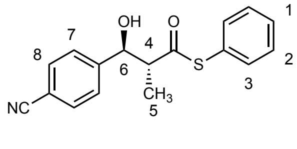

Following general method **B**, **6e** was prepared from 4-formylbenzonitrile (131 mg, 1.00 mmol, 1 equiv) and MAHT **2** (252 mg, 1.20 mmol, 1.20 equiv). The reaction was stirred for 24 h. The residue was purified by column chromatography (eluting with 5% ethyl acetate–hexane initially, grading to 40% ethyl acetate–hexane). The product was obtained as a colorless oil (276 mg, 93%, 11:89 d.r. (syn:anti)).

Major diastereomer:

^{1}H NMR (500 MHz, $CDCl_3$) δ 7.68 – 7.63 (m, 2H, H_8), 7.50 – 7.39 (m, 5H, $H_{1,2/3,7}$), 7.38 – 7.33 (m, 2H, $H_{2/3}$), 4.89 (dd, J = 7.4, 5.3 Hz, 1H, H_6), 3.16 – 3.05 (m, 2H, $H_{4,OH}$), 1.18 (d, J = 7.1 Hz, 3H, H_5).

^{13}C NMR (126 MHz, $CDCl_3$) δ 201.6 (C), 147.0 (C), 134.5 (2 × CH), 132.4 (2 × CH), 129.85 (CH), 129.4 (2 × CH), 127.4 (2 × CH), 126.9 (C), 118.7 (C), 112.0 (CN), 75.8 (CH), 54.8 (CH), 15.5 (CH_3).

Minor diastereomer (detectable non-overlapping resonances):

^{1}H NMR (500 MHz, $CDCl_3$) δ 5.22 – 5.18 (m, 1H, H_6), 3.02 (qd, J = 7.1, 3.8 Hz, 1H, H_4), 1.22 (d, J = 7.1 Hz, 3H, H_5).

^{13}C NMR (126 MHz, $CDCl_3$) δ 202.1 (C), 146.5 (C), 134.6 (2 × CH), 132.3 (2 × CH), 129.94 (CH), 129.5 (2 × CH), 127.0 (2 × CH), 118.8 (C), 111.5 (CN), 73.0 (CH), 54.4 (CH), 11.5 (CH_3).
HPLC (AD-H, *n*-hexane/^{i}PrOH=90:10, 1.0 mL/min, 298 K, 254 nm): t_R(syn, major) = 12.6 min, t_R(syn, minor) = 14.8 min, t_R(anti, major) = 20.6 min, t_R(anti, minor) = 24.8 min, d.r. (syn:anti) = 11:89, e.r.(syn) = 64:36 (28% *ee*), e.r.(anti) = 98:2 (96% *ee*).
HRMS-CI (m/z): $[M + Na]^+$ calcd for $C_{17}H_{15}NO_2SNa$, 320.0721; found, 320.0702.
R_f = 0.30 (20% ethyl acetate-hexane; UV).

S-phenyl (2S,3S)-3-hydroxy-2-methyl-3-(4-(trifluoromethyl)phenyl)propanethioate **5f**

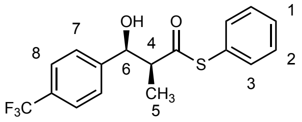

Following general method **A**, **5f** was prepared from 4-(trifluoromethyl)benzaldehyde (137 µL, 1.00 mmol, 1 equiv) and MAHT **2** (252 mg, 1.20 mmol, 1.20 equiv). The reaction was stirred for 24 h. The residue was purified by column chromatography (eluting with 5% ethyl acetate–hexane initially, grading to 40% ethyl acetate–hexane). The product was obtained as a colorless liquid (305 mg, 90%, 95:5 d.r. (syn:anti).

Major diastereomer:

^{1}H NMR (500 MHz, $CDCl_3$) δ 7.64 (d, *J* = 8.0 Hz, 2H, H_8), 7.47 (d, *J* = 8.0 Hz, 2H, H_7), 7.46 – 7.41 (m, 3H, $H_{1,3}$), 7.39 – 7.35 (m, 2H, H_2), 5.17 (d, *J* = 4.3 Hz, 1H, H_6), 3.27 (s, 1H, OH), 3.06 (qd, *J* = 7.1, 4.3 Hz, 1H, H_4), 1.27 (d, *J* = 7.1 Hz, 3H, H_5).
^{13}C NMR (126 MHz, $CDCl_3$) δ 201.8 (C), 145.2 (C), 134.5 (2 × CH), 130.3 (q, *J* = 32.3 Hz, C) 129.8 (CH), 129.3 (2 × CH), 126.8 (C),126.5 (2 × CH), 125.2 (q, *J* = 3.8 Hz, 2 × CH), 124.2 (q, *J* = 262.2 Hz, CF_3), 73.2 (CH), 54.6 (CH), 11.5 (CH_3).
^{19}F NMR (470 MHz, $CDCl_3$) δ -62.4.

Minor diastereomer (detectable non-overlapping resonances):

^{1}H NMR (500 MHz, $CDCl_3$) δ 4.88 (d, *J* = 8.1 Hz, 1H, H_6), 1.12 (d, J = 7.1 Hz, 3H, H_5).
^{13}C NMR (126 MHz, $CDCl_3$) δ 201.6 (C), 145.5 (C), 134.4 (2 × CH), 129.7 (2 × CH), 127.1 (C), 75.8 (CH), 55.0 (CH), 15.2 (CH_3).
HPLC (AD-H, n-hexane/^{i}PrOH=90:10, 1.0 mL/min, 298 K, 254 nm): t_R(syn, minor) = 6.1 min, t_R(syn, major) = 7.2 min, t_R(anti, minor) = 9.5 min, t_R(anti, major) = 12.8 min, d.r. (syn:anti) = 95:5, e.r.(syn) = 98:2 (96% *ee*), e.r.(anti) = 88:12 (76% *ee*).
HRMS-CI (m/z): $[M + Na]^+$ calcd for $C_{17}H_{15}F_3O_2SNa$, 363.0643; found, 363.0638.
R_f = 0.39 (20% ethyl acetate-hexane; UV).

S-phenyl (2R,3S)-3-hydroxy-2-methyl-3-(4-(trifluoromethyl)phenyl)propanethioate **6f**

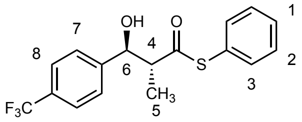

Following general method **B**, **6f** was prepared from 4-(trifluoromethyl)benzaldehyde (137 µL, 1.00 mmol, 1 equiv) and MAHT **2** (252 mg, 1.20 mmol, 1.20 equiv). The reaction was stirred for 24 h. The residue was purified by column chromatography (eluting with 5% ethyl acetate–hexane initially, grading to 40% ethyl acetate–hexane). The product was obtained as a white solid (302 mg, 89%, 14:86 d.r. (syn:anti)).

Major diastereomer:

^{1}H NMR (500 MHz, $CDCl_3$) δ 7.67 – 7.60 (m, 2H, H_8), 7.52 – 7.47 (m, 2H, H_7), 7.46 – 7.41 (m, 3H, $H_{1,3}$), 7.40 – 7.34 (m, 2H, H_2), 4.91 (dd, *J* = 7.7, 5.1 Hz, 1H, H_6), 3.16 – 3.09 (m, 1H, H_4), 2.88 (d, *J* = 5.1 Hz, 1H, OH), 1.18 (d, *J* = 7.2 Hz, 3H, H_5).
^{13}C NMR (126 MHz, $CDCl_3$) δ 201.8 (C), 145.6 (C), 134.58 (2 × CH), 130.5 (q, *J* = 33.0 Hz, C), 129.8 (2 × CH), 129.4 (CH), 127.08 (2 × CH), 126.6 (C), 125.6 (q, *J* = 3.8 Hz, 2 × CH), 124.2 (q, *J* = 271.2 Hz, CF_3), 76.1 (CH), 55.0 (CH), 15.6 (CH_3).
^{19}F NMR (470 MHz, $CDCl_3$) δ -62.6.

Minor diastereomer (detectable non-overlapping resonances):

^{1}H NMR (500 MHz, $CDCl_3$) δ 5.26 – 5.20 (m, 1H, H_6), 3.05 (qd, J = 7.1, 3.6 Hz, 1H, H_4), 2.96 (d, J = 2.6 Hz, 1H, OH), 1.24 (d, J = 7.1 Hz, 3H, H_5).

^{13}C NMR (126 MHz, $CDCl_3$) δ 202.4 (C), 145.1 (C), 134.63 (2 × CH), 129.9 (CH), 129.5 (2 × CH), 127.13 (2 × CH), 126.9 (C), 125.4 (q, J = 3.9 Hz, 2 × CH), 73.1 (CH), 54.5 (CH), 11.5 (CH_3).

HPLC (AD-H, *n*-hexane/^{i}PrOH=90:10, 1.0 mL/min, 298 K, 254 nm): t_R(syn, major) = 6.1 min, t_R(syn, minor) = 7.3 min, t_R(anti, major) = 9.5 min, t_R(anti, minor) = 12.8 min, d.r. (syn:anti) = 14:86, e.r.(syn) = 63:37 (25% *ee*), e.r.(anti) = 97:3 (94% *ee*).

HRMS-CI (m/z): $[M + Na]^+$ calcd for $C_{17}H_{15}F_3O_2SNa$, 363.0643; found, 363.0627.

R_f = 0.39 (20% ethyl acetate-hexane; UV).

The absolute stereochemistry for **6f** was confirmed by x-ray crystallography (see **Fig. S4**). The crystal was grown by a slow evaporation of dichloromethane.

S-phenyl (2S,3S)-3-hydroxy-2-methyl-3-(p-tolyl)propanethioate **5g**

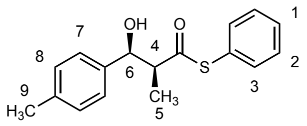

Following general method **A**, **5g** was prepared from *p*-tolualdehyde (238 μL, 2.00 mmol, 1 equiv) and MAHT **2** (505 mg, 2.40 mmol, 1.20 equiv) in toluene (5.00 mL, 0.40 M). The reaction was stirred for 48 h. The residue was purified by column chromatography (eluting with 5% ethyl acetate–hexane initially, grading to 40% ethyl acetate–hexane). The product was obtained as a white solid (221 mg, 39%, 94:6 d.r. (syn:anti)).

Major diastereomer:

^{1}H NMR (500 MHz, $CDCl_3$) δ 7.43 – 7.37 (m, 3H, $H_{1,2}$), 7.36 – 7.30 (m, 2H, H_3), 7.27 – 7.22 (m, 2H H_8), 7.17 (d, J = 7.9 Hz, 2H, H_7), 5.10 (d, J = 4.2 Hz, 1H, H_6), 3.07 – 3.00 (m, 1H, H_4), 2.73 (bs, 1H, OH), 2.35 (s, 3H, H_9), 1.26 (d, J = 7.1 Hz, 3H, H_5).

^{13}C NMR (126 MHz, $CDCl_3$) δ 202.2 (C), 138.2 (C), 137.4 (C), 134.6 (2 × CH), 129.7 (CH), 129.4 (2 × CH), 129.1 (2 × CH), 127.9 (C), 126.1 (2 × CH), 73.8 (CH), 55.1 (CH), 21.3 (CH_3), 11.8 (CH_3).

Minor diastereomer (detectable non-overlapping resonances):

^{1}H NMR (500 MHz, $CDCl_3$) δ 4.80 (d, J = 8.4 Hz, 1H, H_6), 3.13 – 3.07 (m, 1H, H_4), 1.08 (d, J = 7.1 Hz, 3H, H_5).

^{13}C NMR (126 MHz, $CDCl_3$) δ 201.8 (C), 138.6 (C), 138.1 (C), 130.1 (CH), 129.6 (2 × CH),129.3 (2 × CH), 127.6 (CH), 126.7 (2 × CH), 76.6 (CH), 55.4 (CH), 15.6 (CH_3).

HPLC (AD-H, *n*-hexane/EtOH=85:15, 1.0 mL/min, 298 K, 254 nm): t_R(syn, minor) = 9.3 min, t_R(syn, major) = 9.7 min, t_R(anti, major) = 11.2 min, t_R(anti, minor) = 20.6 min, d.r. (syn:anti) = 94:6, e.r.(syn) = 97:3 (94% *ee*), e.r.(anti) = 86:14 (72% *ee*).

HRMS-CI (m/z): $[M + Na]^+$ calcd for $C_{17}H_{18}O_2SNa$, 309.925; found, 309.919.

R_f = 0.35 (20% ethyl acetate-hexane; UV).

S-phenyl (2R,3S)-3-hydroxy-2-methyl-3-(p-tolyl)propanethioate **6g**

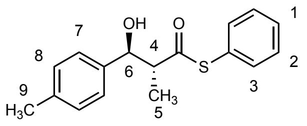

Following general method **B**, **6g** was prepared from *p*-tolualdehyde (238 μL, 2.00 mmol, 1 equiv) and MAHT **2** (505 mg, 2.40 mmol, 1.20 equiv) in toluene (5.00 mL, 0.40 M). The reaction was stirred for 48 h. The residue was purified by column chromatography (eluting with 5% ethyl acetate–hexane initially, grading to 40% ethyl acetate–hexane). The product was obtained as a white solid (280 mg, 49%, 24:76 d.r. (syn:anti)).

Major diastereomer:

^{1}H NMR (500 MHz, $CDCl_3$) δ 7.42 (s, 5H, $H_{1\text{-}3}$), 7.28 – 7.22 (m, 2H, H_8), 7.18 (dd, J = 8.0, 4.3 Hz, 2H, H_7), 4.82 (d, J = 8.4 Hz, 1H, H_6), 3.11 (dq, J = 8.4, 7.1 Hz, 1H, H_4), 2.56 (bs, 1H, OH), 2.36 (s, 3H, H_9), 1.10 (d, J = 7.1 Hz, 3H, H_5).

^{13}C NMR (126 MHz, $CDCl_3$) δ 201.8 (C), 138.7 (C), 138.1 (C), 134.6 (2 × CH), 129.6 (CH), 129.40 (2 × CH), 129.35 (2 × CH), 127.6 (C), 126.7 (2 × CH), 76.7 (CH), 55.5 (CH), 21.3 (CH_3), 15.6 (CH_3).

Minor diastereomer (detectable non-overlapping resonances):

^{1}H NMR (500 MHz, $CDCl_3$) δ 5.12 (d, *J* = 4.2 Hz, 1H, H_6), 3.04 (qd, *J* = 7.1, 4.2 Hz, 1H, H_4), 1.26 (d, *J* = 7.1 Hz, 3H, H_5).

^{13}C NMR (126 MHz, $CDCl_3$) δ 202.3 (C), 138.2 (C), 137.5 (C), 134.7 (2 × CH), 129.7 (CH), 129.39 (2 × CH), 129.2 (2 × CH), 127.3 (C), 126.2 (2 × CH), 73.8 (CH), 55.1 (CH), 21.3 (CH_3), 11.8 (CH_3).

HPLC (AD-H, *n*-hexane/EtOH=85:15, 1.0 mL/min, 298 K, 254 nm): t_R(syn, major) = 9.3 min, t_R(syn, minor) = 9.9 min, t_R(anti, minor) = 11.2 min, t_R(anti, major) = 20.4 min, d.r. (syn:anti) = 24:76, e.r.(syn) = 76:24 (51% *ee*), e.r.(anti) = 97:3 (93% *ee*).

HRMS-CI (m/z): $[M + Na]^+$ calcd for $C_{17}H_{18}O_2SNa$, 309.0925; found, 309.0906.

R_f = 0.35 (20% ethyl acetate-hexane; UV).

Methyl 3-((1S,2S)-1-hydroxy-2-methyl-3-oxo-3-(phenylthio)propyl)benzoate **5h**

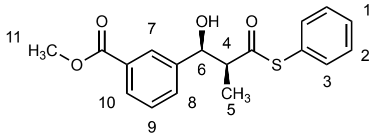

Following general method **A**, **5h** was prepared from methyl 3-formylbenzoate (164 mg, 1.00 mmol, 1 equiv) and MAHT **2** (252 mg, 1.20 mmol, 1.20 equiv). The reaction was stirred for 48 h. The residue was purified by column chromatography (eluting with 5% ethyl acetate–hexane initially, grading to 40% ethyl acetate–hexane). The product was obtained as a colorless liquid (286 mg, 87%, 94:6 d.r. (syn:anti)).

Major diastereomer:

^{1}H NMR (500 MHz, $CDCl_3$) δ 8.05 – 8.00 (m, 1H, H_7), 7.98 – 7.95 (m, 1H, H_8), 7.60 – 7.58 (m, 1H, H_{10}), 7.47 – 7.40 (m, 4H, $H_{1,3,9}$), 7.38 – 7.34 (m, 2H, H_2), 5.23 (d, *J* = 3.7 Hz, 1H, H_6), 3.93 (s, 3H, H_{11}), 3.08 (qd, *J* = 7.1, 3.7 Hz, 1H, H_4), 1.24 (d, *J* = 7.1 Hz, 3H, H_5).

^{13}C NMR (126 MHz, $CDCl_3$) δ 201.9 (C), 167.0 (C), 141.7 (C), 134.54 (2 × CH), 130.8 (CH), 130.2 (C), 129.7 (CH), 129.3 (2 × CH), 128.9 (CH), 128.5 (CH), 127.2 (C), 126.9 (CH), 73.3 (CH), 54.7 (CH), 52.2 (CH_3), 11.6 (CH_3).

Minor diastereomer (detectable non-overlapping resonances):

^{1}H NMR (500 MHz, $CDCl_3$) δ 4.91 (d, *J* = 7.9 Hz, 1H, H_6), 1.15 (d, *J* = 7.1 Hz, 3H, H_5).

^{13}C NMR (126 MHz, $CDCl_3$) δ 201.5 (C), 166.9 (C), 142.1 (C), 134.51 (2 × CH), 131.2 (CH), 130.4 (C), 129.6 (CH), 129.2 (2 × CH), 128.7 (CH), 127.8 (CH), 76.1 (CH), 55.1 (CH), 15.4 (CH_3).

HPLC (AD-H, *n*-hexane/^{i}PrOH=90:10, 1.0 mL/min, 298 K, 254 nm): t_R(syn, minor) = 13.5 min, t_R(syn, major) = 15.6 min, t_R(anti, major) = 22.9 min, t_R(anti, minor) = 29.8 min, d.r. (syn:anti) = 96:6, e.r.(syn) = 98:2 (96% *ee*), e.r.(anti) = 88:12 (76% *ee*).

HRMS-CI (m/z): [M + H]+ calcd for $C_{18}H_{19}O_4S$, 331.1004; found, 331.1003.

R_f = 0.30 (40% ethyl acetate-hexane; UV).

methyl 3-((1S,2R)-1-hydroxy-2-methyl-3-oxo-3-(phenylthio)propyl)benzoate **6h**

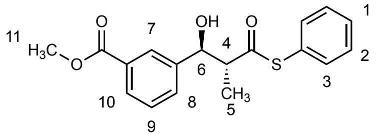

Following general method **B**, **6h** was prepared from methyl 3-formylbenzoate (164 mg, 1.00 mmol, 1 equiv) and MAHT **2** (252 mg, 1.20 mmol, 1.20 equiv). The reaction was stirred for 48 h. The residue was purified by column chromatography (eluting with 5% ethyl acetate–hexane initially, grading to 50% ethyl acetate–hexane). The product was obtained as a colorless oil (283 mg, 86%, 13:87 d.r. (syn:anti)).

Major diastereomer:

^{1}H NMR (500 MHz, $CDCl_3$) δ 8.05 – 8.03 (m, 1H, H_7), 8.02 – 7.99 (m, 1H, H_{10}), 7.62 – 7.55 (m, 1H, H_8), 7.49 – 7.35 (m, 6H, $H_{1\text{-}3,9}$), 4.91 (d, *J* = 7.9 Hz, 1H, H_6), 3.93 (s, 3H, H_{11}), 3.18 – 3.11 (m, 1H, H_4), 1.15 (d, *J* = 7.1 Hz, 3H, H_5).

^{13}C NMR (126 MHz, $CDCl_3$) δ 201.8 (C), 167.0 (C), 142.1 (C), 134.6 (2 × CH), 131.3 (CH), 130.6 (C), 129.8 (CH), 129.54 (CH), 129.47 (2 × CH), 128.9 (CH), 127.9 (CH), 127.3 (C), 76.3 (CH), 55.2 (CH), 52.4 (CH_3), 15.6 (CH_3).

Minor diastereomer (detectable non-overlapping resonances):

^{1}H NMR (500 MHz, $CDCl_3$) δ 7.98 – 7.96 (m, 1H, H_{10}), 5.23 (d, J = 3.7 Hz, 1H, H_6), 3.08 (qd, J = 7.1, 3.7 Hz, 1H, H_4), 1.24 (d, J = 7.1 Hz, 3H, H_5).

^{13}C NMR (126 MHz, $CDCl_3$) δ 141.6 (C), 134.7 (2 × CH), 130.8 (CH), 130.5 (C), 129.9 (CH), 129.45 (2 × CH), 129.0 (CH), 128.6 (CH), 127.0 (C), 73.3 (CH), 54.6 (CH), 52.3 (CH_3), 11.5 (CH_3).

HPLC (AD-H, *n*-hexane/^{i}PrOH=90:10, 1.0 mL/min, 298 K, 254 nm): t_R(syn, minor) = 13.5 min, t_R(syn, major) = 15.5 min, t_R(anti, major) = 21.9 min, t_R(anti, minor) = 29.5 min, d.r. (syn:anti) = 13:87, e.r.(syn) = 75:25 (50% *ee*), e.r.(anti) = 98:2 (96% *ee*).

HRMS-CI (m/z): $[M + H]^+$ calcd for $C_{18}H_{19}O_4S$, 331.1004; found, 331.0985.

R_f = 0.30 (40% ethyl acetate-hexane; UV).

S-phenyl (2S,3S)-3-(3-acetylphenyl)-3-hydroxy-2-methylpropanethioate **5i**

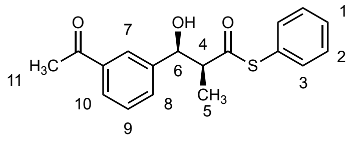

Following general method **A**, **5i** was prepared from 3-acetylbenzaldehyde (156 mg, 1.00 mmol, 1 equiv) and MAHT **2** (252 mg, 1.20 mmol, 1.20 equiv). The reaction was stirred for 48 h. The residue was purified by column chromatography (eluting with 5% ethyl acetate–hexane initially, grading to 40% ethyl acetate–hexane). The product was obtained as a colorless liquid (273 mg, 89%, 92:8 d.r. (syn:anti)).

Major diastereomer:

^{1}H NMR (500 MHz, $CDCl_3$) δ 7.99 – 7.83 (m, 1H, H_7), 7.82 – 7.79 (m, 1H, H_8), 7.62 – 7.58 (m, 1H, H_{10}), 7.50 – 7.45 (m, 1H, H_9), 7.44 – 7.40 (m, 3H, $H_{1,3}$), 7.38 – 7.34 (m, 2H, H_2), 5.23 (d, J = 3.8 Hz, 1H, H_6), 3.08 (qd, J = 7.0, 3.8 Hz, 1H, H_4), 2.62 (s, 3H, H_{11}), 1.24 (d, J = 7.0 Hz, 3H, H_5).

^{13}C NMR (126 MHz, $CDCl_3$) δ 201.4 (C), 198.3 (C), 142.0 (C), 136.9 (C), 134.3 (2 × CH), 131.0 (CH), 129.5 (CH), 129.1 (2 × CH), 128.5 (CH), 127.5 (CH), 126.9 (C), 125.9 (CH), 73.5 (CH), 54.9 (CH), 26.6 (CH_3), 11.9 (CH_3).

Minor diastereomer (detectable non-overlapping resonances):

^{1}H NMR (500 MHz, $CDCl_3$) δ 4.92 (d, J = 7.9 Hz, 1H, H_6), 1.16 (d, J = 7.2 Hz, 3H, H_5).

^{13}C NMR (126 MHz, $CDCl_3$) δ 198.2 (C), 142.3 (C), 137.1 (C), 131.4 (CH), 129.4 (CH), 128.7 (CH), 128.0 (CH), 127.2 (CH), 126.4 (C), 75.9 (CH), 55.0 (CH), 15.1 (CH_3).

HPLC (AD-H, *n*-hexane/^{i}PrOH=88:12, 1.0 mL/min, 298 K, 254 nm): t_R(syn, major) = 12.4 min, t_R(syn, minor) = 14.7 min, t_R(anti, minor) = 16.5 min, t_R(anti, major) = 18.3 min, d.r. (syn:anti) = 92:8, e.r.(syn) = 97:3 (94% *ee*), e.r.(anti) = 85:15 (70% *ee*).

HRMS-CI (m/z): $[M + Na]^+$ calcd for $C_{18}H_{18}$O3SNa, 337.0874; found, 337.0863.

R_f = 0.47 (20% ethyl acetate-hexane; UV).

S-phenyl (2R,3S)-3-(3-acetylphenyl)-3-hydroxy-2-methylpropanethioate **6i**

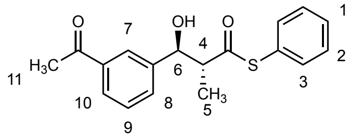

Following general method **B**, **6i** was prepared from 3-acetylbenzaldehyde (156 mg, 1.00 mmol, 1 equiv) and MAHT **2** (252 mg, 1.20 mmol, 1.20 equiv). The reaction was stirred for 48 h. The residue was purified by column chromatography (eluting with 5% ethyl acetate–hexane initially, grading to 40% ethyl acetate–hexane). The product was obtained as a white solid (275 mg, 88%, 14:86 d.r. (syn:anti)).

Major diastereomer:

^{1}H NMR (500 MHz, $CDCl_3$) δ 7.97 – 7.88 (m, 2H, $H_{7,10}$), 7.61 – 7.56 (m, 1H, H_8), 7.52 – 7.46 (m, 1H, H_9), 7.45 – 7.33 (m, 5H, $H_{1\text{-}3}$), 4.93 (d, J = 7.9 Hz, 1H, H_6), 3.21 – 3.11 (m, 1H, H_4), 2.62 (s, 3H, H_{11}), 1.16 (d, J = 7.2 Hz, 3H, H_5).

^{13}C NMR (126 MHz, $CDCl_3$) δ 201.9 (C), 198.1 (C), 142.3 (C), 137.5 (C), 134.61 (2 × CH), 131.4 (CH), 129.8 (CH), 129.4 (2 × CH), 129.0 (CH), 128.3 (CH), 127.2 (C), 126.5 (CH), 76.3 (CH), 55.1 (CH), 26.9 (CH_3), 15.6 (CH_3).

Minor diastereomer (detectable non-overlapping resonances):

^{1}H NMR (500 MHz, $CDCl_3$) δ 5.24 (d, *J* = 3.7 Hz, 1H, H_6), 3.08 (qd, *J* = 7.1, 3.8 Hz, 1H, H_4), 1.24 (d, *J* = 7.1 Hz, 3H, H_5).
^{13}C NMR (126 MHz, $CDCl_3$) δ 198.2 (C), 141.8 (C), 134.64 (2 × CH), 131.0 (CH), 129.9 (CH), 129.5 (2 × CH), 128.8 (CH), 127.8 (CH), 127.0 (C), 126.0 (CH), 73.3 (CH), 54.6 (CH), 11.5 (CH_3).
HPLC (OD-H, *n*-hexane/^{i}PrOH=88:12, 1.0 mL/min, 298 K, 254 nm): t_R(syn, minor) = 12.4 min, t_R(syn, major) = 14.7 min, t_R(anti, major) = 16.5 min, t_R(anti, minor) = 18.3 min, d.r. (syn:anti) = 14:86, e.r.(syn) = 77:23 (54% *ee*), e.r.(anti) = 98:2 (96% *ee*).
HRMS-CI (m/z): $[M + H]^+$ calcd for $C_{18}H_{19}O_3S$, 315.1055; found, 315.1034.
R_f = 0.47 (40% ethyl acetate-hexane; UV).

S-phenyl (2S,3S)-3-hydroxy-3-(3-hydroxy-4-nitrophenyl)-2-methylpropanethioate **5j**

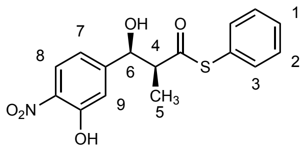

Following general method **A**, **5j** was prepared from 3-hydroxy-4-nitrobenzaldehyde (167 mg, 1.00 mmol, 1 equiv) and MAHT **2** (252 mg, 1.20 mmol, 1.20 equiv). The reaction was stirred for 24 h. The residue was purified by column chromatography (eluting with 5% ethyl acetate–hexane initially, grading to 40% ethyl acetate–hexane). The product was obtained as a yellow solid (313 mg, 94%, 93:7 d.r. (syn:anti)).
^{1}H and ^{13}C NMR data for **5j** prepared in this way were in agreement with the literature.(*24*)
Major diastereomer:
^{1}H NMR (500 MHz, $CDCl_3$) δ 10.61 (s, 1H, OH), 8.07 (d, *J* = 8.8 Hz, 1H, H_8), 7.47 – 7.32 (m, 5H, $H_{1\text{-}3}$), 7.18 (d, *J* = 1.9 Hz, 1H, H_9), 6.96 (dd, *J* = 8.8, 1.9 Hz, 1H, H_7), 5.16 (d, *J* = 3.7 Hz, 1H, H_6), 3.25 (bs, 1H, OH), 3.03 (qd, *J* = 7.1, 3.7 Hz, 1H, H_4), 1.22 (d, *J* = 7.1 Hz, 3H, H_5),
^{13}C NMR (126 MHz, $CDCl_3$) δ 201.9 (C), 155.10 (C), 152.1 (C), 134.5 (2 × CH), 132.7 (C), 129.9 (CH), 129.41 (2 × CH), 126.6 (C), 125.2 (CH), 118.0 (CH), 117.3 (CH), 72.5 (CH), 53.9 (CH), 11.3 (CH_3).
Minor diastereomer (detectable non-overlapping resonances):
^{1}H NMR (500 MHz, $CDCl_3$) δ 4.84 (dd, *J* = 7.5, 3.8 Hz, 1H, H_6), 3.13 – 3.08 (m, 1H, H_4), 1.19 (d, J = 7.2 Hz, 3H, H_5).
^{13}C NMR (126 MHz, $CDCl_3$) δ 201.4 (C), 155.06 (C), 152.4 (C), 134.4 (2 × CH), 133.0 (C), 129.8 (CH), 129.35 (2 × CH), 126.8 (C), 125.3 (CH), 118.4 (CH), 117.8 (CH), 75.4 (CH), 54.4 (CH), 15.40 (CH_3).
HPLC (AD-H, *n*-hexane/^{i}PrOH=90:10, 1.0 mL/min, 298 K, 254 nm): t_R(syn, minor) = 13.6 min, t_R(syn, major) = 15.1 min, t_R(anti, minor) = 26.4 min, t_R(anti, major) = 28.9 min, d.r. (syn:anti) = 93:7, e.r.(syn) = 99:1 (98% *ee*), e.r.(anti) = 96:4 (92% *ee*).
HRMS-CI (m/z): $[M + H]^+$ calcd for $C_{16}H_{16}NO_5S$, 334.0749; found, 334.0741.
R_f = 0.31 (20% ethyl acetate-hexane; UV).
The absolute stereochemistry for **5j** was confirmed by x-ray crystallography (see **Fig. S4**). The crystal was grown by a slow evaporation of dichloromethane.

S-phenyl (2R,3S)-3-hydroxy-3-(3-hydroxy-4-nitrophenyl)-2-methylpropanethioate **6j**

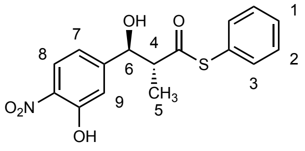

Following general method **B**, **6j** was prepared from 3-hydroxy-4-nitrobenzaldehyde (167 mg, 1.00 mmol, 1 equiv) and MAHT **2** (252 mg, 1.20 mmol, 1.20 equiv). The reaction was stirred for 24 h. The residue was purified by column chromatography (eluting with 5% ethyl acetate–hexane initially, grading to 40% ethyl acetate–hexane). The product was obtained as a white solid (331 mg, 99%, 12:88 d.r. (syn:anti)).
Major diastereomer:
^{1}H NMR (500 MHz, $CDCl_3$) δ 10.63 (s, 1H, OH), 8.14 – 8.08 (m, 1H, H_8), 7.46 – 7.34 (m, 5H, $H_{1\text{-}3}$), 7.17 (d, *J* = 1.9 Hz, 1H, H_9), 7.03 – 6.96 (m, 1H, H_7), 4.86 (dd, *J* = 7.0, 5.8 Hz, 1H, H_6), 3.17 – 3.01 (m, 2H, $H_{4,OH}$), 1.25 (d, *J* = 7.1 Hz, 3H, H_5).

^{13}C NMR (126 MHz, $CDCl_3$) δ 201.7 (C), 155.27 (C), 152.4 (C), 134.6 (2 × CH), 133.2 (C), 130.0 (CH), 129.5 (2 × CH), 126.8 (C), 125.5 (CH), 118.4 (CH), 117.9 (CH), 75.6 (CH), 54.4 (CH), 15.7 (CH_3).

Minor diastereomer (detectable non-overlapping resonances):

^{1}H NMR (500 MHz, $CDCl_3$) δ 7.21 (dd, J = 1.8, 0.8 Hz, 1H, H_9), 5.23 – 5.19 (m, 1H, H_6), 1.22 (d, J = 7.2 Hz, 3H, H_5).

^{13}C NMR (126 MHz, $CDCl_3$) δ 155.30 (C), 134.7 (2 × CH), 130.1 (CH), 129.6 (2 × CH), 125.3 (CH), 118.0 (CH), 117.5 (CH), 72.5 (CH), 53.8 (CH), 11.3 (CH_3).

HPLC (AD-H, *n*-hexane/^{i}PrOH=90:10, 1.0 mL/min, 298 K, 254 nm): t_R(syn, major) = 13.8 min, t_R(syn, minor) = 15.8 min, t_R(anti, major) = 26.7 min, t_R(anti, minor) = 29.2 min, d.r. (syn:anti) = 12:88, e.r.(syn) = 73:27 (46% *ee*), e.r.(anti) = 98:2 (96% *ee*).

HRMS-CI (m/z): $[M + Na]^+$ calcd for $C_{16}H_{15}NO_5SNa$, 356.0569; found, 356.0546.

R_f = 0.31 (20% ethyl acetate-hexane; UV).

The absolute stereochemistry for **6j** was confirmed by x-ray crystallography (see **Fig. S4**). The crystal was grown by a slow evaporation of dichloromethane.

S-phenyl (2S,3S)-3-hydroxy-2-methyl-3-(2-nitrophenyl)propanethioate **5k**

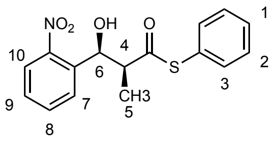

Following general method **A**, **5k** was prepared from 2-nitrobenzaldehyde (151 mg, 1.00 mmol, 1 equiv) and MAHT **2** (252 mg, 1.20 mmol, 1.20 equiv) in toluene (5 mL, 0.4 M). The reaction was stirred for 48 h. The residue was purified by column chromatography (eluting with 5% ethyl acetate–hexane initially, grading to 50% ethyl acetate–hexane). The product was obtained as a colorless oil (253 mg, 96%, 89:11 d.r. (syn:anti)).

Major diastereomer:

^{1}H NMR (500 MHz, $CDCl_3$) δ 8.04 (dd, J = 8.2, 1.3 Hz, 1H, H_{10}), 7.89 (dd, J = 7.8, 1.4 Hz, 1H, H_9), 7.68 (td, J = 7.6, 1.4 Hz, 1H, H_8), 7.50 – 7.45 (m, 1H, H_7), 7.43 (s, 5H, $H_{1\text{-}3}$), 5.81 (d, J = 2.6 Hz, 1H, H_6), 3.38 – 3.30 (m, 1H, H_4), 1.24 (d, J = 7.3 Hz, 3H, H_5).

^{13}C NMR (126 MHz, $CDCl_3$) δ 203.1 (C), 147.3 (C), 136.4 (C), 134.7 (2 × CH),133.5 (CH), 129.8 (CH), 129.6 (CH), 129.4 (2 × CH), 128.6 (CH), 127.0 (C), 125.0 (CH), 69.1 (CH), 51.8 (CH), 11.2 (CH_3).

Minor diastereomer (detectable non-overlapping resonances):

^{1}H NMR (500 MHz, $CDCl_3$) δ 8.01 – 7.97 (m, 1H), 5.49 (d, J = 5.4 Hz, 1H, H_6), 1.37 (d, J = 7.2 Hz, 3H, H_5),

^{13}C NMR (126 MHz, $CDCl_3$) δ 202.3 (C), 148.1 (C), 137.4 (C), 134.4 (2 × CH), 129.8 (CH) 128.8 (2 × CH), 128.7 (2 × CH), 126.9 (C), 124.9 (2 × C), 71.9 (CH), 53.3 (CH), 16.0 (CH_3).

HPLC (AD-H, *n*-hexane/^{i}PrOH=90:10, 1.0 mL/min, 298 K, 254 nm): t_R(syn, major) = 12.8 min, t_R(syn, minor) = 14.4 min, t_R(anti, minor) = 17.7 min, t_R(anti, major) = 19.9 min, d.r. (syn:anti) = 89:11, e.r.(syn) = 96:4 (93% *ee*), e.r.(anti) = 88:12 (76% *ee*).

HRMS-CI (m/z): $[M + Na]^+$ calcd for $C_{16}H_{15}NO_4SNa$, 340.0619; found, 340.0602.

R_f = 0.40 (20% ethyl acetate-hexane; UV).

S-phenyl (2R,3S)-3-hydroxy-2-methyl-3-(2-nitrophenyl)propanethioate **6k**

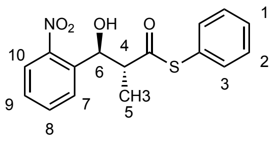

Following general method **A**, **6k** was prepared from 2-nitrobenzaldehyde (151 mg, 1.00 mmol, 1 equiv) and MAHT **2** (252 mg, 1.20 mmol, 1.20 equiv). The reaction was stirred for 48 h. The residue was purified by column chromatography (eluting with 5% ethyl acetate–hexane initially, grading to 40% ethyl acetate–hexane). The product was obtained as a yellow oil (260 mg, 82%, 22:78 d.r. (syn:anti)).

Major diastereomer:

^{1}H NMR (500 MHz, $CDCl_3$) δ 8.01 (dt, *J* = 7.9, 0.9 Hz, 1H, H_{10}), 7.70 – 7.65 (m, 2H, $H_{8,9}$), 7.53 – 7.45 (m, 1H, H_7), 7.43 – 7.37 (m, 2H, H_2), 7.29 – 7.26 (m, 3H, $H_{1,3}$), 5.50 (d, *J* = 5.4 Hz, 1H, H_6), 3.65 (bs, 1H, OH), 3.34 (qd, *J* = 7.2, 5.4 Hz, 1H, H_4), 1.40 (d, *J* = 7.2 Hz, 3H, H_5).

^{13}C NMR (126 MHz, $CDCl_3$) δ 202.5 (C), 148.2 (C), 136.4 (C), 134.5 (2 × CH), 133.6 (CH), 129.93 (CH), 129.45 (2 × CH), 128.9 (CH), 128.8 (CH), 126.9 (C), 125.0 (CH), 72.0 (CH), 53.3 (CH), 16.1 (CH_3).

Minor diastereomer (detectable non-overlapping resonances):

^{1}H NMR (500 MHz, $CDCl_3$) δ 8.05 (dd, *J* = 8.2, 1.3 Hz, 1H, H_{10}), 7.89 (dd, *J* = 7.9, 1.3 Hz, 1H, H_8), 7.44 (s, 5H, H_{1-3}), 5.82 (d, *J* = 2.6 Hz, 1H, H_6), 1.24 (d, *J* = 7.2 Hz, 3H, H_5).

^{13}C NMR (126 MHz, $CDCl_3$) δ 203.3 (C), 137.6 (C), 134.7 (2 × CH), 129.87 (CH), 129.7 (CH),129.46 (2 × CH), 128.7 (CH), 127.0 (C), 125.1 (CH), 69.1 (CH), 51.8 (CH), 11.2 (CH_3).

HPLC (AD-H, *n*-hexane/^{i}PrOH=90:10, 1.0 mL/min, 298 K, 254 nm): t_R(syn, minor) = 12.8 min, t_R(syn, major) = 14.4 min, t_R(anti, major) = 17.7 min, t_R(anti, minor) = 19.9 min, d.r. (syn:anti) = 22:78, e.r.(syn) = 70:30 (40% *ee*), e.r.(anti) = 96:4 (92% *ee*).

HRMS-CI (m/z): $[M + Na]^+$ calcd for $C_{16}H_{15}NO_4SNa$, 340.0619; found, 340.0597.

R_f = 0.31 (20% ethyl acetate-hexane; UV).

S-phenyl (2S,3S)-3-(3,5-bis(trifluoromethyl)phenyl)-3-hydroxy-2-methylpropanethioate **5l**

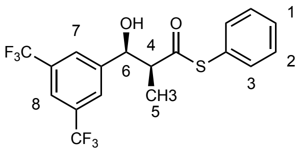

Following general method **A**, **5l** was prepared from 3,5-bis(trifluoromethyl)benzaldehyde (165 μL, 1.00 mmol, 1 equiv) and MAHT **2** (252 mg, 1.20 mmol, 1.20 equiv). The reaction was stirred for 24 h. The residue was purified by column chromatography (eluting with 5% ethyl acetate–hexane initially, grading to 40% ethyl acetate–hexane). The product was obtained as a white solid (376 mg, 92%, 87:13 d.r. (syn:anti)).

Major diastereomer:

^{1}H NMR (500 MHz, $CDCl_3$) δ 7.86-7.84 (m, 3H, $H_{7,8}$), 7.49 – 7.35 (m, 5H, H_{1-3}), 5.25 (d, *J* = 3.6 Hz, 1H, H_6), 3.35 (s, 1H, OH), 3.06 (m, 1H, H_4), 1.25 (d, *J* = 7.1 Hz, 3H, H_5).

^{13}C NMR (126 MHz, $CDCl_3$) δ 202.3 (C), 144.0 (C), 134.6 (2 × CH), 131.8 (q, *J* = 33.4 Hz, 2 × C), 130.0 (CH), 129.50 (2 × CH), 126.9 –126.5 (m, 2 × CH), 126.6 (C), 123.4 (d, *J* = 272.6 Hz, 2 × CF_3), 121.8 – 121.6 (m, CH), 72.6 (CH), 54.3 (CH), 11.4 (CH_3).

^{19}F NMR (470 MHz, $CDCl_3$) δ -62.7.

Minor diastereomer (detectable non-overlapping resonances):

^{1}H NMR (500 MHz, $CDCl_3$) δ 4.96 (d, *J* = 7.4 Hz, 1H, H_6), 3.14 (dd, J = 7.2,7.2 Hz, 1H, H_4), 1.21 (d, *J* = 7.1 Hz, 3H, H_5).

^{13}C NMR (126 MHz, $CDCl_3$) δ 201.9 (C), 144.5 (C), 134.5 (CH), 129.9 (C), 129.45 (CH), 75.4 (CH), 54.7 (CH), 15.3 (CH_3).

HPLC (OD-H, *n*-hexane/^{i}PrOH=90:10, 1.0 mL/min, 298 K, 254 nm): t_R(syn, major) = 4.9 min, t_R(syn, minor) = 5.9 min, t_R(anti, minor) = 6.7 min, t_R(anti, major) = 9.8 min, d.r. (syn:anti) = 87:13, e.r.(syn) = 99:1 (98% *ee*), e.r.(anti) = 93:7 (86% *ee*).

^{19}F NMR (470 MHz, $CDCl_3$) δ -62.8.

HRMS-CI (m/z): $[M + Na]^+$ calcd for $C_{18}H_{14}F_6O_2SNa$, 431.0516; found, 431.0504.

R_f = 0.55 (20% ethyl acetate-hexane; UV).

S-phenyl (2R,3S)-3-(3,5-bis(trifluoromethyl)phenyl)-3-hydroxy-2-methylpropanethioate **6l**

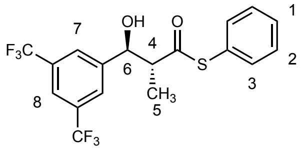

Following general method **B**, **6l** was prepared from 3,5-bis(trifluoromethyl)benzaldehyde (165 μL, 1.00 mmol, 1 equiv) and MAHT **2** (252 mg, 1.20 mmol, 1.20 equiv). The reaction was stirred for 24 h. The residue was purified by column chromatography (eluting with 5% ethyl acetate–hexane initially, grading to 40% ethyl acetate–hexane). The product was obtained as a yellow oil (371 mg, 91%, 13:87 d.r. (syn:anti)).

Major diastereomer:

^{1}H NMR (500 MHz, $CDCl_3$) δ 7.88 – 7.82 (m, 3H, $H_{7,8}$), 7.47 – 7.32 (m, 5H, H_{1-3}), 4.99 (dd, *J* = 7.1, 5.5 Hz, 1H, H_6), 3.20 – 3.10 (m, 2H, $H_{4,OH}$), 1.29 – 1.20 (m, 3H, H_5).

^{13}C NMR (126 MHz, $CDCl_3$) δ 201.8 (C), 144.4 (C), 134.6 (2 × CH), 132.0 (q, *J* = 34.0 Hz, 2 × C), 130.0 (CH), 129.5 (2 × CH), 126.91 – 126.88 (m, 2 × CH), 126.70 (C), 123.37 (d, *J* = 272.8 Hz, 2 × CF_3), 122.28 – 122.15 (m, CH), 75.6 (CH), 54.6 (CH), 15.6 (CH_3).

^{19}F NMR (470 MHz, $CDCl_3$) δ -62.8.

Minor diastereomer (detectable non-overlapping resonances):

^{1}H NMR (500 MHz, $CDCl_3$) δ 5.30 (t, *J* = 2.9 Hz, 1H), 3.06 (qd, *J* = 7.1, 3.5 Hz, 1H).

^{13}C NMR (126 MHz, $CDCl_3$) δ 143.8 (C), 134.7 (2 × CH), 130.1 (CH), 129.6 (2 × CH), 72.5 (CH), 54.1 (CH), 11.4 (CH_3).

HPLC (OD-H, *n*-hexane/^{i}PrOH=90:10, 1.0 mL/min, 298 K, 254 nm): t_R(syn, minor) = 4.9 min, t_R(syn, major) = 5.9 min, t_R(anti, major) = 6.7 min, t_R(anti, minor) = 9.9 min, d.r. (syn:anti) = 13:87, e.r.(syn) = 79:21 (58% *ee*), e.r.(anti) = 98:2 (96% *ee*).

HRMS-CI (m/z): $[M + Na]^+$ calcd for $C_{18}H_{14}F_6O_2SNa$, 431.0516; found, 431.0495.

R_f = 0.55 (20% ethyl acetate-hexane; UV).

S-phenyl (2S,3S)-3-(furan-2-yl)-3-hydroxy-2-methylpropanethioate **5m**

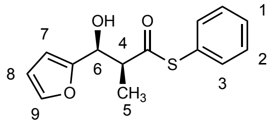

Following general method **A**, **5m** was prepared from furfural (83 μL, 1.00 mmol, 1 equiv) and MAHT **2** (252 mg, 1.20 mmol, 1.20 equiv). The reaction was stirred for 24 h. The residue was purified by column chromatography (eluting with 5% ethyl acetate–hexane initially, grading to 40% ethyl acetate–hexane). The product was obtained as a colorless liquid (213 mg, 81%, 93:7 d.r. (syn:anti)).

^{1}H and ^{13}C NMR data for **5m** prepared in this way were in agreement with the reported data for racemic **5m**.(*52*)

Major diastereomer:

^{1}H NMR (500 MHz, $CDCl_3$) δ 7.43 – 7.39 (m, 4H, $H_{1,3,9}$), 7.38 - 7.35 (m, 2H, H_2), 6.36 (dd, *J* = 3.3, 1.8 Hz, 1H, H_8), 6.33 (d, *J* = 3.3 Hz, 1H, H_7), 5.08 (dd, *J* = 5.0 Hz, 1H, H_6), 3.27 (qd, *J* = 7.1, 5.0 Hz, 1H, H_4), 1.36 (d, *J* = 7.1 Hz, 3H, H_5).

^{13}C NMR (126 MHz, $CDCl_3$) δ 200.6 (C), 153.8 (C), 141.9 (CH), 134.3 (2 × CH), 129.4 (CH), 129.1 (2 × CH), 127.0 (C), 110.2 (CH), 107.1 (CH), 68.7 (CH), 52.6 (CH), 12.9 (CH_3).

Minor diastereomer (detectable non-overlapping resonances):

^{1}H NMR (500 MHz, $CDCl_3$) δ 4.87 (d, *J* = 7.8 Hz, 1H, H_6), 3.38 - 3.32 (m, 1H, H_4), 1.20 (d, J = 7.1 Hz, 3H, H_5).

^{13}C NMR (126 MHz, $CDCl_3$) δ 201.1 (C), 153.9 (C), 142.3 (CH), 127.2 (C), 107.7 (CH), 69.7 (CH), 52.7 (CH), 14.9 (CH_3).

HPLC (AD-H, *n*-hexane/^{i}PrOH=92:8, 1.0 mL/min, 298 K, 254 nm): t_R(syn, minor) = 10.9 min, t_R(syn, major) = 13.4 min, t_R(anti, major) = 17.9 min, t_R(anti, minor) = 19.7 min, d.r. (syn:anti) = 93:7, e.r.(syn) = 96:4 (91% *ee*), e.r.(anti) = 79:21 (58% *ee*).

HRMS-CI (m/z): $[M + Na]^+$ calcd for $C_{14}H_{14}O_3SNa$, 285.0561; found, 285.0545.

R_f = 0.43 (20% ethyl acetate-hexane; UV).

S-phenyl (2R,3S)-3-(furan-2-yl)-3-hydroxy-2-methylpropanethioate **6m**

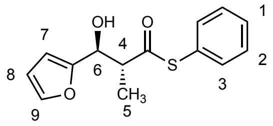

Following general method **B**, **6m** was prepared from furfural (83 μL, 1.00 mmol, 1 equiv) and MAHT **2** (252 mg, 1.20 mmol, 1.20 equiv). The reaction was stirred for 48 h. The residue was purified by column chromatography (eluting with 5% ethyl acetate–hexane initially, grading to 50% ethyl acetate–hexane). The product was obtained as a colorless oil (230 mg, 88%, 16:84 d.r. (syn:anti)).

Major diastereomer:

^{1}H NMR (500 MHz, $CDCl_3$) δ 7.47 – 7.33 (m, 6H, $H_{1\text{-}3,9}$), 6.36 (dd, J = 3.3, 1.8 Hz, 1H, H_8), 6.35 – 6.32 (m, 1H, H_7), 4.87 (d, J = 7.8 Hz, 1H, H_6), 3.41 – 3.31 (m, 1H, H_4), 1.21 (d, J = 7.1 Hz, 3H, H_5).

^{13}C NMR (126 MHz, $CDCl_3$) δ 201.5 (C), 154.1 (C), 142.6 (CH), 134.6 (2 × CH), 129.71 (CH), 129.4 (2 × CH), 127.4 (C), 110.4 (CH), 107.9 (CH), 70.3 (CH), 52.8 (CH), 15.4 (CH_3).

Minor diastereomer (detectable non-overlapping resonances):

^{1}H NMR (500 MHz, $CDCl_3$) δ 5.09 (d, J = 5.0 Hz, 1H, H_6), 3.27 (qd, J = 7.1, 5.0 Hz, 1H, H_4), 1.36 (d, J = 7.1 Hz, 3H, H_5).

^{13}C NMR (126 MHz, $CDCl_3$) δ 201.3 (C), 153.8 (C), 142.2 (CH), 134.7 (2 × CH), 129.74 (CH), 127.2 (C), 110.5 (CH), 107.3 (CH), 69.1 (CH), 52.5 (CH), 12.8 (CH_3).

HPLC (AD-H, *n*-hexane/^{i}PrOH=92:8, 1.0 mL/min, 298 K, 254 nm): t_R(syn, major) = 10.9 min, t_R(syn, minor) = 13.4 min, t_R(anti, minor) = 17.9 min, t_R(anti, major) = 19.7 min, d.r. (syn:anti) = 16:84, e.r.(syn) = 83:17 (67% *ee*), e.r.(anti) = 99:1 (98% *ee*).

HRMS-CI (m/z): $[M + Na]^+$ calcd for $C_{14}H_{14}O_3SNa$, 285.0561; found, 285.0546

R_f = 0.43 (20% ethyl acetate-hexane; UV).

S-phenyl (2S,3S)-3-hydroxy-2-methyl-3-(oxazol-4-yl)propanethioate **5n**

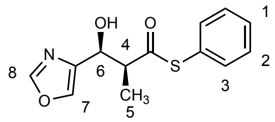

Following general method **A**, **5n** was prepared from 4-oxazolecarboxaldehyde (99 mg, 1.00 mmol, 1 equiv) and MAHT **2** (252 mg, 1.20 mmol, 1.20 equiv). The reaction was stirred for 24 h. The residue was purified by column chromatography (eluting with 5% ethyl acetate–hexane initially, grading to 40% ethyl acetate–hexane). The product was obtained as a colorless liquid (240 mg, 91%, 92:8 d.r. (syn:anti)).

Major diastereomer:

^{1}H NMR (500 MHz, $CDCl_3$) δ 7.88 (s, 1H, H_8), 7.65 (s, 1H, H_7), 7.45 – 7.36 (m, 5H, $H_{1\text{-}3}$), 5.12 (d, J = 3.9, Hz, 1H, H_6), 3.89 (bs, 1H, OH), 3.34 (qd, J = 7.2, 3.9 Hz, 1H, H_4), 1.28 (d, J = 7.2 Hz, 3H, H_5).

^{13}C NMR (126 MHz, $CDCl_3$) δ 201.4 (C), 151.3 (CH), 140.6 (C), 135.9 (CH), 134.48 (2 × CH), 129.5 (CH), 129.2 (2 × CH), 127.0 (C), 68.0 (CH), 52.3 (CH), 12.0 (CH_3).

Minor diastereomer (detectable non-overlapping resonances):

^{1}H NMR (500 MHz, $CDCl_3$) δ 4.86 (d, J = 6.5 Hz, 1H, H_6), 3.42 – 3.38 (m, 1H, H_4).

^{13}C NMR (126 MHz, $CDCl_3$) δ 201.2 (C), 151.5 (CH), 140.8 (C), 136.0 (CH), 134.40 (2 × CH), 129.4 (CH), 129.1 (2 × CH), 127.2 (C), 68.9 (CH), 52.9 (CH), 15.0 (CH_3).

HPLC (AD-H, *n*-hexane/EtOH=92:8, 1.0 mL/min, 298 K, 254 nm): t_R(syn, minor) = 25.0 min, t_R(syn, major) = 27.5 min, t_R(anti, minor) = 38.6 min, t_R(anti, major) = 69.6 min, d.r. (syn:anti) = 92:8, e.r.(syn) = 99:1 (98% *ee*), e.r.(anti) = 88:12 (76% *ee*).

HRMS-CI (m/z): $[M + Na]^+$ calcd for $C_{13}H_{13}NO_3SNa$, 286.0514; found, 286.0506.

R_f = 0.25 (20% ethyl acetate-hexane; UV).

S-phenyl (2R,3S)-3-hydroxy-2-methyl-3-(oxazol-4-yl)propanethioate **6n**

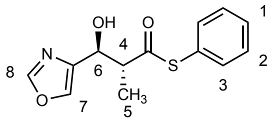

Following general method **B**, **6n** was prepared from 4-oxazolecarboxaldehyde (99 mg, 1.00 mmol, 1 equiv) and MAHT **2** (252 mg, 1.20 mmol, 1.20 equiv). The reaction was stirred for 24 h. The residue was purified by column chromatography (eluting with 5% ethyl acetate–hexane initially, grading to 70% ethyl acetate–hexane). The product was obtained as a colorless oil (250 mg, 95%, 19:81 d.r. (syn:anti)).

Major diastereomer:

^{1}H NMR (500 MHz, $CDCl_3$) δ 7.88 (s, 1H, H_8), 7.63 (s, 1H, H_7), 7.44 – 7.35 (m, 5H, H_{1-3}), 4.88 – 4.64 (m, 1H, H_6), 3.49 (bs, 1H, OH), 3.43 – 3.36 (m, 1H, H_4), 1.33 – 1.25 (m, 3H, H_5).

^{13}C NMR (126 MHz, $CDCl_3$) δ 201.7 (C), 151.5 (CH), 141.1 (C), 136.0 (CH), 134.5 (2 × CH), 129.6 (CH), 129.3 (2 × CH), 127.3 (C), 69.4 (CH), 52.7 (CH), 15.2 (CH_3).

Minor diastereomer (detectable non-overlapping resonances):

^{1}H NMR (500 MHz, $CDCl_3$) δ 7.87 (s, 1H, H_8), 7.65 (s, 1H, H_7), 5.17 – 5.12 (m, 1H, H_6), 3.35 – 3.31 (m, 1H, H_4).

^{13}C NMR (126 MHz, $CDCl_3$) δ 202.0 (C), 151.3 (CH), 140.6 (C), 136.1 (CH), 134.6 (2 × CH), 129.7 (CH), 129.4 (2 × CH), 127.1 (C), 68.3 (CH), 52.2 (CH), 12.0 (CH_3).

HPLC (AD-H, *n*-hexane/EtOH=92:8, 1.0 mL/min, 298 K, 254 nm): t_R(syn, major) = 25.0 min, t_R(syn, minor) = 27.9 min, t_R(anti, major) = 38.6 min, t_R(anti, minor) = 69.6 min, d.r. (syn:anti) = 19:81, e.r.(syn) = 80:20 (60% *ee*), e.r.(anti) = 97:3 (94% *ee*).

HRMS-CI (m/z): $[M + Na]^+$ calcd for $C_{13}H_{13}NO_3SNa$, 286.0514; found, 286.0494.

R_f = 0.25 (20% ethyl acetate-hexane; UV).

S-phenyl (2S,3S)-3-hydroxy-2-methyl-3-(thiazol-5-yl)propanethioate **5o**

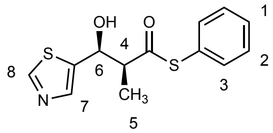

Following general method **A**, **5o** was prepared from 5-thiazolecarboxaldehyde (89 μL, 1.00 mmol, 1 equiv) and MAHT **2** (252 mg, 1.20 mmol, 1.20 equiv). The reaction was stirred for 48 h. The residue was purified by column chromatography (eluting with 5% ethyl acetate–hexane initially, grading to 40% ethyl acetate–hexane). The product was obtained as a colorless liquid (265 mg, 95%, 88:12 d.r. (syn:anti)).

Major diastereomer:

^{1}H NMR (500 MHz, $CDCl_3$) δ 8.72 (s, 1H, H_8), 7.70 (s, 1H, H_7), 7.44 – 7.37 (m, 3H, $H_{1,3}$), 7.34 – 7.32 (m, 2H, H_2), 5.37 (d, *J* = 5.2 Hz, 1H, H_6), 3.15 – 3.05 (m, 1H, H_4), 1.38 (d, *J* = 7.1 Hz, 3H, H_5).

^{13}C NMR (126 MHz, $CDCl_3$) δ 200.6 (C), 153.1 (CH), 141.02 (C), 139.7 (CH), 134.4 (2 × CH), 129.7 (CH), 129.3 (2 × CH), 126.7 (C), 68.7 (CH), 55.6 (CH), 13.1 (CH_3).

Minor diastereomer (detectable non-overlapping resonances):

^{1}H NMR (500 MHz, $CDCl_3$) δ 5.20 (d, *J* = 7.9 Hz, 1H, H_6), 1.20 (d, *J* = 7.1 Hz, 3H, H_5).

^{13}C NMR (126 MHz, $CDCl_3$) 153.7 (CH), 141.07 (C), 140.3 (CH), 69.9 (CH), 55.4 (CH), 15.1 (CH_3).

HPLC (AD-H, *n*-hexane/EtOH=90:10, 1.0 mL/min, 298 K, 254 nm): t_R(syn, major) = 20.0 min, t_R(syn, minor) = 27.2 min, t_R(anti, minor) = 30.1 min, t_R(anti, major) = 33.7 min, d.r. (syn:anti) = 88:12, e.r.(syn) = 95:5 (89% *ee*), e.r.(anti) = 66:34 (33% *ee*).

HRMS-CI (m/z): $[M + H]^+$ calcd for $C_{13}H_{14}NO_2S_2$, 280.0466; found, 280.0460.

R_f = 0.19 (20% ethyl acetate-hexane; UV).

S-phenyl (2R,3S)-3-hydroxy-2-methyl-3-(thiazol-5-yl)propanethioate **6o**

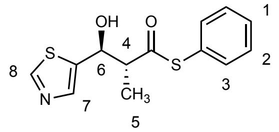

Following general method **B**, **6o** was prepared from 5-thiazolecarboxaldehyde (89 µL, 1.00 mmol, 1 equiv) and MAHT **2** (252 mg, 1.20 mmol, 1.20 equiv). The reaction was stirred for 24 h. The residue was purified by column chromatography (eluting with 5% ethyl acetate–hexane initially, grading to 40% ethyl acetate–hexane). The product was obtained as a colorless oil (257 mg, 92%, 9:91 d.r. (syn:anti)).

Major diastereomer:

^{1}H NMR (500 MHz, $CDCl_3$) δ 8.82 (s, 1H, H_8), 7.81 (s, 1H, H_7), 7.45 – 7.39 (m, 5H, H_{1-3}), 5.21 (d, *J* = 7.4 Hz, 1H, H_6), 3.21 – 3.09 (m, 1H, H_4), 1.25 (d, *J* = 7.2 Hz, 3H, H_5).

^{13}C NMR (126 MHz, $CDCl_3$) δ 201.5 (C), 153.8 (CH), 140.8 (C), 140.4 (CH), 134.6 (2 × CH), 129.9 (CH), 129.49 (2 × CH), 126.9 (C), 70.5 (CH), 55.2 (CH), 15.6 (CH_3).

Minor diastereomer (detectable non-overlapping resonances):

^{1}H NMR (500 MHz, $CDCl_3$) δ 8.81 (s, 1H, H_8), 7.77 (s, 1H, H_7), 5.49 – 5.41 (m, 1H, H_6), 1.38 (d, *J* = 7.1 Hz, 3H, H_5).

^{13}C NMR (126 MHz, $CDCl_3$) δ 153.3 (CH), 134.7 (2 × CH), 130.0 (CH), 129.52 (2 × CH), 69.0 (CH), 55.0 (CH), 12.7 (CH_3).

HPLC (AD-H, *n*-hexane/EtOH=90:10, 1.0 mL/min, 298 K, 254 nm): t_R(syn, minor) = 20.1 min, t_R(syn, major) = 27.2 min, t_R(anti, major) = 29.8 min, t_R(anti, minor) = 33.7 min, d.r. (syn:anti) = 9:91, e.r.(syn) = 79:21 (58% *ee*), e.r.(anti) = 99:1 (98% *ee*).

HRMS-CI (m/z): $[M + H]^+$ calcd for $C_{13}H_{14}NO_2S_2$, 280.0466; found, 280.0448.

R_f = 0.20 (20% ethyl acetate-hexane; UV).

S-phenyl (2S,3S)-3-(benzo[b]thiophen-2-yl)-3-hydroxy-2-methylpropanethioate **5p**

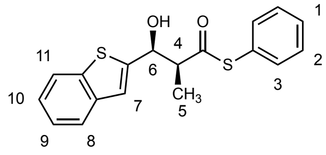

Following general method **A**, **5p** was prepared from benzo[b]thiophene-2-carboxaldehyde (162 mg, 1.00 mmol, 1 equiv) and MAHT **2** (252 mg, 1.20 mmol, 1.20 equiv). The reaction was stirred for 24 h. The residue was purified by column chromatography (eluting with 5% ethyl acetate–hexane initially, grading to 40% ethyl acetate–hexane). The product was obtained as a colorless liquid (285 mg, 87%, 94:6 d.r. (syn:anti)).

Major diastereomer:

^{1}H NMR (500 MHz, $CDCl_3$) δ 7.85 – 7.82 (m, 1H, H_{11}), 7.75 – 7.71 (m, 1H, H_8), 7.45 – 7.39 (m, 3H, H_{1-3}), 7.38 – 7.30 (m, 4H, $H_{2,9,10}$), 7.23 (s, 1H, H_7), 5.46 (d, *J* = 4.3 Hz, 1H, H_6), 3.23 (qd, *J* = 7.1, 4.3 Hz, 1H, H_4), 1.40 (d, *J* = 7.1 Hz, 3H, H_5).

^{13}C NMR (126 MHz, $CDCl_3$) δ 201.5 (C), 145.8 (C), 139.5 (C), 139.3 (C), 134.6 (2 × CH), 129.7 (CH), 129.3 (2 × CH), 126.9 (C), 124.4 (CH), 124.2 (CH), 123.5 (CH), 122.4 (CH), 120.8 (CH), 71.2 (CH), 55.0 (CH), 12.4 (CH_3).

Minor diastereomer (detectable non-overlapping resonances):

^{1}H NMR (500 MHz, $CDCl_3$) δ 5.19 (d, *J* = 7.8 Hz, 1H, H_6), 1.27 (d, *J* = 7.2 Hz, 3H, H_5).

^{13}C NMR (126 MHz, $CDCl_3$) δ 201.4 (C), 146.0 (C), 139.6 (C), 139.2 (C), 134.5 (2 × CH), 124.54 (CH), 124.47 (CH), 123.7 (CH), 122.6 (CH), 121.8 (CH), 73.1 (CH), 15.6 (CH_3).

HPLC (AD-H, *n*-hexane/^{i}PrOH=90:10, 1.0 mL/min, 298 K, 254 nm): t_R(syn, minor) = 12.9 min, t_R(syn, major) = 16.0 min, t_R(anti, major) = 19.4 min, t_R(anti, minor) = 27.9 min, d.r. (syn:anti) = 94:6, e.r.(syn) = 96:4 (92% *ee*), e.r.(anti) = 86:14 (72% *ee*).

HRMS-CI (m/z): $[M + Na]^+$ calcd for $C_{18}H_{16}O_2S_2Na$, 351.0489; found, 351.0482.

R_f = 0.48 (20% ethyl acetate-hexane; UV).

The absolute stereochemistry for **5p** was confirmed by x-ray crystallography (see fig. S4). The crystal was grown by a slow evaporation of dichloromethane.

S-phenyl (2R,3S)-3-(benzo[b]thiophen-2-yl)-3-hydroxy-2-methylpropanethioate **6p**

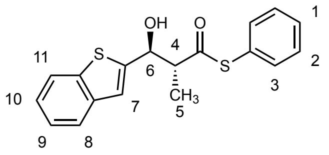

Following general method **B**, **6p** was prepared from benzo[b]thiophene-2-carboxaldehyde (166 mg, 1.00 mmol, 1 equiv) and MAHT **2** (252 mg, 1.20 mmol, 1.20 equiv). The reaction was stirred for 24 h. The residue was purified by column chromatography (eluting with 5% ethyl acetate–hexane initially, grading to 50% ethyl acetate–hexane). The product was obtained as a colorless oil (298 mg, 91%, 8:92 d.r. (syn:anti)).

Major diastereomer:

^{1}H NMR (500 MHz, $CDCl_3$) δ 7.86 – 7.81 (m, 1H, H_8), 7.76 – 7.71 (m, 1H, H_{11}), 7.45 – 7.39 (m, 5H, $H_{1\text{-}3}$), 7.39 – 7.30 (m, 2H, $H_{9,10}$), 7.25 – 7.21 (m, 1H, H_7), 5.19 (dd, *J* = 8.0, 3.7 Hz, 1H, H_6), 3.33 – 3.17 (m, 1H, H_4), 3.07 (d, *J* = 3.7 Hz, 1H, OH), 1.27 (d, *J* = 7.1 Hz, 3H, H_5).

^{13}C NMR (126 MHz, $CDCl_3$) δ 201.5 (C), 146.1 (C), 139.7 (C), 139.3 (C), 134.6 (2 × CH), 129.7 (CH), 129.38 (2 × CH), 127.3 (C), 124.62 (CH), 124.55 (CH), 123.8 (CH), 122.7 (CH), 121.9 (CH), 73.3 (CH), 55.0 (CH), 15.8 (CH_3).

Minor diastereomer (detectable non-overlapping resonances):

^{1}H NMR (500 MHz, $CDCl_3$) δ 5.47 – 5.44 (m, 1H, H_6), 1.40 (d, *J* = 7.1 Hz, 3H, H_5).

^{13}C NMR (126 MHz, $CDCl_3$) δ 201.8 (C), 145.7 (C), 139.6 (C), 139.4 (C), 134.7 (2 × CH), 129.8 (CH), 129.41 (2 × CH), 127.0 (C), 124.47 (CH), 124.3 (CH), 123.6 (CH), 122.5 (CH), 120.9 (CH), 71.2 (CH), 54.9 (CH), 12.4 (CH_3).

HPLC (AD-H, *n*-hexane/^{i}PrOH=90:10, 1.0 mL/min, 298 K, 254 nm): t_R(syn, major) = 12.9 min, t_R(syn, minor) = 16.0 min, t_R(anti, minor) = 19.4 min, t_R(anti, major) = 27.9 min, d.r. (syn:anti) = 8:92, e.r.(syn) = 75:25 (50% *ee*), e.r.(anti) = 98:2 (96% *ee*).

HRMS-CI (m/z): $[M + Na]^+$ calcd for $C_{18}H_{16}O_2S_2Na$, 351.0489; found, 351.0482.

R_f = 0.48 (20% ethyl acetate-hexane; UV).

S-phenyl (2S,3R)-3-hydroxy-2-methyl-5-phenylpentanethioate **5q**

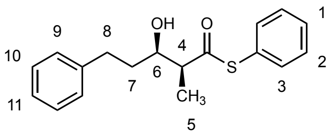

Following general method **A**, **5q** was prepared from hydrocinnamaldehyde (132 µL, 1.00 mmol, 1 equiv) and MAHT **2** (252 mg, 1.20 mmol, 1.20 equiv). The reaction was stirred for 24 h. The residue was purified by column chromatography (eluting with 5% ethyl acetate–hexane initially, grading to 40% ethyl acetate–hexane). The product was obtained as a colorless oil (281 mg, 94%, 80:20 d.r. (syn:anti)).

^{1}H and ^{13}C NMR data for **5q** prepared in this way were in agreement with the literature.(*24*) The chiral HPLC data for **5q** prepared in this way were in agreement with that obtained for a control sample **5q** prepared according to the literature.(*24*)

Major diastereomer:

^{1}H NMR (500 MHz, $CDCl_3$) δ 7.45 – 7.38 (m, 5H, $H_{1\text{-}3}$), 7.30 (t, *J* = 7.5 Hz, 2H, H_{10}), 7.24 – 7.18 (m, 3H, $H_{9,11}$), 4.01 (dt, *J* = 9.3, 3.6 Hz, 1H, H_6), 2.91 – 2.80 (m, 2H, $H_{4,8}$), 2.77 – 2.64 (m, 1H, H_8), 2.44 (bs, 1H, OH), 1.92 – 1.79 (m, 1H, H_7), 1.76 – 1.69 (m, 1H, H_7), 1.33 (m, 3H, H_5).

^{13}C NMR (126 MHz, $CDCl_3$) δ 202.5 (C), 141.8 (C), 134.61 (2 × CH), 129.7 (CH), 129.4 (2 × CH), 128.59 (2 × CH), 128.57 (2 × CH), 127.2 (C), 126.07 (CH), 71.3 (CH), 53.0 (CH), 36.0 (CH_2), 32.4 (CH_2), 11.9 (CH_3).

Minor diastereomer (detectable non-overlapping resonances):

^{1}H NMR (500 MHz, $CDCl_3$) δ 3.78 – 3.73 (m, 1H, H_6).

^{13}C NMR (126 MHz, $CDCl_3$) δ 141.6 (C), 134.57 (2 × CH), 127.3 (C), 126.05 (CH), 73.3 (CH), 53.6 (CH), 36.9 (CH_2), 32.1 (CH_2), 15.5 (CH_3).

HPLC (AD-H, *n*-hexane/^{i}PrOH=90:10, 1.0 mL/min, 298 K, 254 nm): t_R(syn, minor) = 7.7 min, t_R(syn, major) = 8.3 min, t_R(anti, minor) = 9.3 min, t_R(anti, major) = 12.0 min, d.r. (syn:anti) = 80:20, e.r.(syn) = 99:1 (98% *ee*), e.r.(anti) = 97:3 (95% *ee*).

HRMS-CI (m/z): $[M + Na]^+$ calcd for $C_{18}H_{20}O_2SNa$, 323.1082; found, 323.1082.

R_f = 0.50 (20% ethyl acetate-hexane; UV).

S-phenyl (2S,3R)-3-hydroxy-2-methyl-5-phenylpentanethioate **6q**

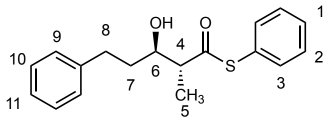

Following general method **B**, **6q** was prepared from hydrocinnamaldehyde (132 µL, 1.00 mmol, 1 equiv) and MAHT **2** (252 mg, 1.20 mmol, 1.20 equiv). The reaction was stirred for 24 h. The residue was purified by column chromatography (eluting with 5% ethyl acetate–hexane initially, grading to 40% ethyl acetate–hexane). The product was obtained as a colorless oil (298 mg, 99%, 17:83 d.r. (syn:anti)).

^{1}H and ^{13}C NMR data for **6q** prepared in this way were in agreement with the literature.(*53*)

Major diastereomer:

^{1}H NMR (500 MHz, CDCl$_3$) δ 7.39 (q, *J* = 2.1 Hz, 5H, $H_{1\text{-}3}$), 7.30 – 7.24 (m, 2H, H_{10}), 7.25 – 7.12 (m, 3H, $H_{9,11}$), 3.72 (qt, *J* = 6.0, 3.3 Hz, 1H, H_6), 2.90 – 2.76 (m, 2H, $H_{4,8}$), 2.75 – 2.60 (m, 1H, H_8), 2.44 (bs, 1H, OH), 1.91 – 1.73 (m, 2H, H_7), 1.34 – 1.27 (m, 3H, H_5).

^{13}C NMR (126 MHz, CDCl$_3$) δ 202.50 (C), 141.9 (C), 134.59 (2 × CH), 129.7 (CH), 129.4 (2 × CH), 128.63 (2 × CH), 128.59 (2 × CH), 127.34 (C), 126.07 (CH), 73.4 (CH), 53.6 (CH), 37.0 (CH$_2$), 32.1 (CH$_2$), 15.5 (CH$_3$).

Minor diastereomer (detectable non-overlapping resonances):

^{1}H NMR (500 MHz, CDCl$_3$) δ 4.01 – 3.95 (m, 1H, H_6), 1.73 – 1.66 (m, 1H, H_7).

^{13}C NMR (126 MHz, CDCl$_3$) δ 202.52 (C), 141.8 (C), 134.64 (2 × CH), 128.61 (2 × CH), 127.25 (C), 126.09 (CH), 71.3 (CH), 53.0 (CH), 36.0 (CH$_2$), 32.4 (CH$_2$), 11.8 (CH$_3$).

HPLC (AD-H, *n*-hexane/^{i}PrOH=90:10, 1.0 mL/min, 298 K, 254 nm): t_R(syn, major) = 7.7 min, t_R(syn, minor) = 8.4 min, t_R(anti, major) = 9.3 min, t_R(anti, minor) = 12.0 min, d.r. (syn:anti) = 17:83, e.r.(syn) = 80:20 (61% *ee*), e.r.(anti) = 97:3 (94% *ee*).

HRMS-CI (m/z): $[M + Na]^+$ calcd for $C_{18}H_{20}O_2SNa$, 323.1082; found, 323.1067.

R_f = 0.50 (20% ethyl acetate-hexane; UV).

S-phenyl (2S,3R)-3-hydroxy-2-methyltridec-12-enethioate **5r**

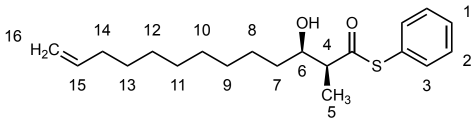

Following general method **A**, **5r** was prepared from 10-undecenal (215 µL, 1.00 mmol, 1 equiv) and MAHT **2** (252 mg, 1.20 mmol, 1.20 equiv). The reaction was stirred for 24 h. The residue was purified by column chromatography (eluting with 5% ethyl acetate–hexane initially, grading to 40% ethyl acetate–hexane). The product was obtained as a colorless liquid (302 mg, 94%, 76:24 d.r. (syn:anti)).

Major diastereomer:

^{1}H NMR (500 MHz, CDCl$_3$) δ 7.32 (s, 5H, $H_{1\text{-}3}$), 5.72 (ddt, *J* = 16.9, 10.1, 6.7 Hz, 1H, H_{15}), 4.93 – 4.81 (m, 2H, H_{16}), 3.88 - 3.85 (m, 1H, H_6), 2.71 (qd, *J* = 7.1, 3.7 Hz, 1H, H_4), 2.29 (bs, 1H, OH), 1.97 – 1.92 (m, 2H, H_{14}), 1.50 – 1.33 (m, 2H, H_7), 1.28 – 1.17 (m, 15H, $H_{5,8\text{-}13}$).

^{13}C NMR (126 MHz, CDCl$_3$) δ 202.37 (C), 139.2 (CH), 134.59 (2 × CH), 129.63 (CH), 129.34 (2 × CH), 127.3 (C), 114.2 (CH$_2$), 72.0 (CH), 52.9 (CH), 34.2 (CH$_2$), 33.9 (CH$_2$), 29.6 (2 × CH$_2$), 29.5 (CH$_2$), 29.2 (CH$_2$), 29.0 (CH$_2$), 26.0 (CH$_2$), 11.6 (CH$_3$).

Minor diastereomer (detectable non-overlapping resonances):

^{1}H NMR (500 MHz, CDCl$_3$) δ 3.64 (m, 1H, H_6), 2.78 (m, 1H, H_4).

^{13}C NMR (126 MHz, CDCl$_3$) δ 202.31 (C), 134.55 (2 × CH), 129.60 (CH), 129.60 (CH), 129.32 (2 × CH), 127.4 (C), 74.01 (CH), 53.5 (CH), 35.0 (CH$_2$), 25.6 (CH$_2$), 15.3 (CH$_3$).

HPLC (AD-H, n-hexane/^{i}PrOH=93:7, 1.0 mL/min, 298 K, 254 nm): t_R(syn, minor) = 5.6 min, t_R(syn, major) = 5.9 min, t_R(anti, minor) = 6.7 min, t_R(anti, major) = 7.5 min, d.r. (syn:anti) = 75:25, e.r.(syn) = 98:2 (96% *ee*), e.r.(anti) = 95:5 (90% *ee*).

HRMS-CI (m/z): $[M + Na]^+$ calcd for $C_{20}H_{30}O_2SNa$, 357.1864; found, 357.1852.
R_f = 0.50 (20% ethyl acetate-hexane; UV).

S-phenyl (2R,3R)-3-hydroxy-2-methyltridec-12-enethioate **6r**

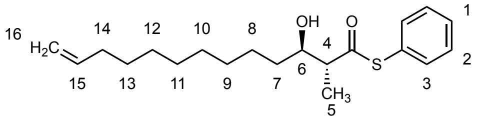

Following general method **B**, **6r** was prepared from 10-undecenal (215 µL, 1.00 mmol, 1 equiv) and MAHT **2** (252 mg, 1.20 mmol, 1.20 equiv). The reaction was stirred for 24 h. The residue was purified by column chromatography (eluting with 5% ethyl acetate–hexane initially, grading to 40% ethyl acetate–hexane). The product was obtained as a colorless oil (329 mg, 98%, 16:84 d.r. (syn:anti)).

Major diastereomer:

^{1}H NMR (500 MHz, $CDCl_3$) δ 7.42 (s, 5H, H_{1-3}), 5.81 (ddt, J = 16.9, 10.2, 6.7 Hz, 1H, H_{15}), 5.05 – 4.86 (m, 2H, H_{16}), 3.77 – 3.69 (m, 1H, H_6), 2.94 – 2.76 (m, 1H, H_4), 2.27 (d, J = 7.4 Hz, 1H, OH), 2.09 – 2.00 (m, 2H, H_{14}), 1.63 – 1.43 (m, 2H, H_7), 1.44 – 1.24 (m, 15H, $H_{5,8-13}$).

^{13}C NMR (126 MHz, $CDCl_3$) δ 202.4 (C), 139.4 (CH), 134.6 (2 × CH), 129.68 (CH), 129.38 (2 × CH), 127.5 (C), 114.3 (CH_2), 74.1 (CH), 53.6 (CH), 35.1 (CH_2), 34.0 (CH_2), 29.7 (2 × CH_2), 29.6 (CH_2), 29.3 (CH_2), 29.1 (CH_2), 25.7 (CH_2), 15.5 (CH_3).

Minor diastereomer (detectable non-overlapping resonances):

^{1}H NMR (500 MHz, $CDCl_3$) δ 3.99 – 3.94 (m, 1H, H_6), 2.31 (d, J = 3.8 Hz, 1H, OH).

^{13}C NMR (126 MHz, $CDCl_3$) δ 202.5 (C), 134.7 (2 × CH), 129.70 (CH), 129.40 (2 × CH), 127.4 (C), 72.0 (CH), 52.9 (CH), 34.2 (CH_2), 26.1 (CH_2), 11.6 (CH_3).

HPLC (AD-H, *n*-hexane/^{i}PrOH=93:7, 1.0 mL/min, 298 K, 254 nm): t_R(syn, major) = 5.6 min, t_R(syn, minor) = 5.9 min, t_R(anti, major) = 6.9 min, t_R(anti, minor) = 7.5 min, d.r. (syn:anti) = 16:84, e.r.(syn) = 80:20 (60% *ee*), e.r.(anti) = 98:2 (96% *ee*).

HRMS-CI (m/z): $[M + Na]^+$ calcd for $C_{20}H_{30}O_2SNa$, 357.1864; found, 357.1844.
R_f = 0.50 (20% ethyl acetate-hexane; UV).

S-phenyl (2S,3R)-5-(benzyloxy)-3-hydroxy-2-methylpentanethioate **5s**

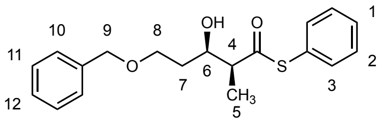

Following general method **A**, **5s** was prepared from 3-(benzyloxy)propanal (155 µL, 1.00 mmol, 1 equiv) and MAHT **2** (252 mg, 1.20 mmol, 1.20 equiv). The reaction was stirred for 24 h. The residue was purified by column chromatography (eluting with 5% ethyl acetate–hexane initially, grading to 40% ethyl acetate–hexane). The product was obtained as a colorless liquid (289 mg, 88%, 76:24 d.r. (syn:anti)).

Major diastereomer:

^{1}H NMR (500 MHz, $CDCl_3$) δ 7.41 (s, 5H, H_{10-12}), 7.38 – 7.28 (m, 5H, H_{1-3}), 4.53 (s, 2H, H_9), 4.13 (ddd, J = 8.8, 5.3, 3.1 Hz, 1H, H_6), 3.76 – 3.72 (m, 1H, H_8), 3.71 – 3.63 (m, 1H, H_8), 2.92 – 2.81 (m, 1H, H_4), 1.91 – 1.75 (m, 2H, H_7), 1.33 (d, J = 7.0 Hz, 3H, H_5).

^{13}C NMR (126 MHz, $CDCl_3$) δ 201.4 (C), 137.9 (C), 134.5 (2 × CH), 129.6 (CH), 129.3 (2 × CH), 128.6 (2 × CH), 127.9 (CH), 127.8 (2 × CH), 127.5 (C), 73.4 (CH_2), 71.6 (CH), 68.7 (CH_2), 53.6 (CH), 33.9 (CH_2), 12.8 (CH_3).

Minor diastereomer (detectable non-overlapping resonances):

^{1}H NMR (500 MHz, $CDCl_3$) δ 4.04 (ddd, J = 9.3, 6.5, 2.7 Hz, 1H, H_6), 1.29 (d, J = 7.1 Hz, 3H, H_5).

^{13}C NMR (126 MHz, $CDCl_3$) δ 129.5 (CH), 129.2 (2 × CH), 127.6 (C), 73.5 (CH_2), 72.8 (CH), 68.6 (CH_2), 53.8 (CH), 33.8 (CH_2), 14.5 (CH_3).

HPLC (AD-H, n-hexane/EtOH=90:10, 1.0 mL/min, 298 K, 254 nm): t_R(syn, minor) = 10.9 min, t_R(syn, major) = 11.6 min, t_R(anti, major) = 13.7 min, t_R(anti, minor) = 15.5 min, d.r. (syn:anti) = 76:24, e.r.(syn) = 98:2 (96% *ee*), e.r.(anti) = 97:3 (94% *ee*).

HRMS-CI (m/z): $[M + Na]^+$ calcd for $C_{19}H_{22}O_3SNa$, 353.1187; found, 353.1180.
R_f = 0.52 (20% ethyl acetate-hexane; UV).

S-phenyl (2R,3R)-5-(benzyloxy)-3-hydroxy-2-methylpentanethioate **6s**

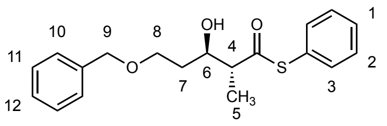

Following general method **B**, **6s** was prepared from 3-(benzyloxy)propanal (155 µL, 1.00 mmol, 1 equiv) and MAHT **2** (252 mg, 1.20 mmol, 1.20 equiv). The reaction was stirred for 24 h. The residue was purified by column chromatography (eluting with 5% ethyl acetate–hexane initially, grading to 40% ethyl acetate–hexane). The product was obtained as a colorless oil (315 mg, 95%, 23:77 d.r. (syn:anti)).

Major diastereomer:

^1H NMR (500 MHz, $CDCl_3$) δ 7.46 – 7.27 (m, 10H, $H_{1-3,10-12}$), 4.53 (s, 2H, H_9), 4.04 (ddd, *J* = 9.3, 6.5, 2.7 Hz, 1H, H_6), 3.78 – 3.72 (m, 1H, H_8), 3.72 – 3.61 (m, 2H, H_8), 3.16 (bs, 1H, OH), 2.98 – 2.83 (m, 1H, H_4), 1.96 – 1.71 (m, 2H, H_7), 1.29 (d, *J* = 7.2 Hz, 3H, H_5).

^{13}C NMR (126 MHz, $CDCl_3$) δ 201.4 (C), 138.0 (C), 134.59 (2 × CH), 129.5 (CH), 129.30 (2 × CH), 128.6 (2 × CH), 127.93 (CH), 127.87 (2 × CH), 127.7 (C), 73.6 (CH_2), 72.9 (CH), 68.7 (CH_2), 53.9 (CH), 34.0 (CH_2), 14.6 (CH_3).

Minor diastereomer (detectable non-overlapping resonances):

^1H NMR (500 MHz, $CDCl_3$) δ 4.13 (ddd, *J* = 8.5, 5.1, 3.0 Hz, 1H, H_6), 1.33 (d, *J* = 7.0 Hz, 3H, H_5).

^{13}C NMR (126 MHz, $CDCl_3$) δ 134.61 (2 × CH), 129.6 (CH), 129.34 (2 × CH), 127.6 (C), 73.5 (CH_2), 71.7 (CH), 68.8 (CH_2), 53.6 (CH), 33.9 (CH_2), 12.9 (CH_3).

HPLC (AD-H, *n*-hexane/EtOH=90:10, 1.0 mL/min, 298 K, 254 nm): t_R(syn, major) = 10.9 min, t_R(syn, minor) = 11.7 min, t_R(anti, minor) = 13.7 min, t_R(anti, major) = 15.6 min, d.r. (syn:anti) = 23:77, e.r.(syn) = 79:21 (58% *ee*), e.r.(anti) = 97:3 (93% *ee*).

HRMS-CI (m/z): $[M + Na]^+$ calcd for $C_{19}H_{22}O_3SNa$, 353.1187; found, 353.1166.
R_f = 0.52 (20% ethyl acetate-hexane; UV).

S-phenyl (2S,3R)-3-hydroxy-2,4-dimethylpentanethioate **5t**

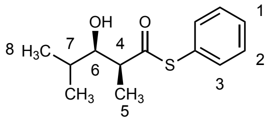

Following general method **A**, **5t** was prepared from isobutyraldehyde (185 µL, 2.00 mmol, 1.0 equiv) and MAHT **2** (505 mg, 2.40 mmol, 1.20 equiv). The reaction was stirred for 48 h. The residue was purified by column chromatography (eluting with 5% ethyl acetate–hexane initially, grading to 40% ethyl acetate–hexane). The product was obtained as a colorless liquid (434 mg, 91%, 93:7 d.r. (syn:anti)).

^1H and ^{13}C NMR data for **5t** prepared in this way were in agreement with the reported data for racemic **5t**.(*54*)

Major diastereomer:

^1H NMR (500 MHz, $CDCl_3$) δ 7.42 (s, 5H, H_{1-3}), 3.65 (dd, *J* = 7.6, 3.6 Hz, 1H, H_6), 2.97 (qd, *J* = 7.0, 3.6 Hz, 1H, H_4), 1.81 – 1.70 (m, 1H, H_7), 1.30 (d, *J* = 7.0 Hz, 3H, H_5), 1.04 (d, *J* = 6.5 Hz, 3H, H_8), 0.92 (d, *J* = 7.0 Hz, 3H, H_9).

^{13}C NMR (126 MHz, $CDCl_3$) δ 202.5 (C), 134.6 (2 × CH), 129.6 (CH), 129.3 (2 × CH), 127.3 (C), 77.0 (CH), 50.5 (CH), 30.8 (CH), 19.3 (CH_3), 18.4 (CH_3), 11.5 (CH_3).

Minor diastereomer (detectable non-overlapping resonances):

^1H NMR (500 MHz, $CDCl_3$) δ 3.46 – 3.44 (m, 1H, H_6), 1.33 (d, *J* = 7.2 Hz, 3H, H_5), 1.02 (d, *J* = 6.9 Hz, 3H, H_8), 0.96 (d, *J* = 6.7 Hz, 3H, H_9).

^{13}C NMR (126 MHz, $CDCl_3$) δ 202.7 (C), 134.5 (2 × CH), 78.9 (CH), 50.9 (CH), 31.3 (CH), 19.9 (CH_3), 16.6 (CH_3), 15.9 (CH_3).

HPLC (AD-H, n-hexane/^{i}PrOH=92:8, 1.0 mL/min, 298 K, 254 nm): t_R(syn, minor) = 6.0 min, t_R(syn, major) = 6.3 min, t_R(anti, major) = 7.1 min, t_R(anti, minor) = 8.2 min, d.r. (syn:anti) = 93:7, e.r.(syn) = 92:8 (84% *ee*), e.r.(anti) = 94:6 (88% *ee*).

HRMS-CI (m/z): [M + H]+ calcd for $C_{13}H_{19}O_2S$, 239.1106; found, 239.1103.
R_f = 0.46 (20% ethyl acetate-hexane; UV).

S-phenyl (2R,3R)-3-hydroxy-2,4-dimethylpentanethioate **6t**

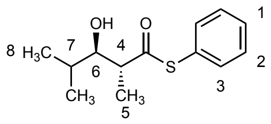

Following general method **B**, **6t** was prepared from isobutyraldehyde (92 µL, 1.00 mmol, 1 equiv) and MAHT **2** (252 mg, 1.20 mmol, 1.20 equiv). The reaction was stirred for 24 h. The residue was purified by column chromatography (eluting with 5% ethyl acetate–hexane initially, grading to 40% ethyl acetate–hexane). The product was obtained as a colorless oil (160 mg, 67%, 19:81 d.r. (syn:anti)).

^{1}H and ^{13}C NMR data for **6t** prepared in this way were in agreement with the reported data for racemic **6t**.(*54*)

Major diastereomer:

^{1}H NMR (500 MHz, $CDCl_3$) δ 7.42 (s, 5H, $H_{1\text{-}3}$), 3.45 (dd, *J* = 5.8, 5.8 Hz, 1H, H_6), 3.04 – 2.94 (m, 1H, H_4), 2.21 (bs, 1H, OH), 1.86 – 1.70 (m, 1H, H_7), 1.33 (d, *J* = 7.1 Hz, 3H, H_5), 1.02 (d, *J* = 6.8 Hz, 3H, H_8), 0.97 (d, *J* = 6.7 Hz, 3H, H_8).

^{13}C NMR (126 MHz, $CDCl_3$) δ 202.8 (C), 134.6 (2 × CH), 129.7 (CH), 129.38 (2 × CH), 127.5 (C), 79.1 (CH), 51.0 (CH), 31.5 (CH), 20.0 (CH_3), 16.8 (CH_3), 16.1 (CH_3).

Minor diastereomer (detectable non-overlapping resonances):

^{1}H NMR (500 MHz, $CDCl_3$) δ 3.65 (dd, *J* = 7.6, 3.7 Hz, 1H, H_6), 1.30 (d, *J* = 7.1 Hz, 1H, H_5), 1.04 (d, *J* = 6.6 Hz, 1H, H_8), 0.92 (d, *J* = 6.8 Hz, 1H, H_8).

^{13}C NMR (126 MHz, $CDCl_3$) δ 202.7 (C), 134.7 (2 × CH), 129.39 (2 × CH), 127.4 (C), 77.1 (CH), 50.5 (CH), 30.9 (CH), 19.3 (CH_3), 18.7 (CH_3), 11.4 (CH_3).

HPLC (AD-H, *n*-hexane/^{i}PrOH=92:8, 1.0 mL/min, 298 K, 254 nm): t_R(syn, major) = 6.0 min, t_R(syn, minor) = 6.3 min, t_R(anti, minor) = 7.1 min, t_R(anti, major) = 8.2 min, d.r. (syn:anti) = 19:81, e.r.(syn) = 57:43 (14% *ee*), e.r.(anti) = 98:2 (96% *ee*).

HRMS-CI (m/z): $[M + Na]^+$ calcd for $C_{13}H_{18}O_2S$, 261.0925; found, 261.0911

R_f = 0.46 (20% ethyl acetate-hexane; UV).

S-phenyl (2S,3S)-3-hydroxy-2-methyldec-4-ynethioate **5u**

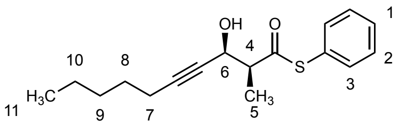

Following general method **A**, **5u** was prepared from 2-octynal (143 µL, 1.00 mmol, 1 equiv) and MAHT **2** (252 mg, 1.20 mmol, 1.20 equiv). The reaction was stirred for 48 h. The residue was purified by column chromatography (eluting with 5% ethyl acetate–hexane initially, grading to 40% ethyl acetate–hexane). The product was obtained as a colorless oil (267 mg, 92%, 90:10 d.r. (syn:anti).

^{1}H and ^{13}C NMR data for **5u** prepared in this way were in agreement with the literature.(*24*)

Major diastereomer:

^{1}H NMR (500 MHz, $CDCl_3$) δ 7.41 (s, 5H, $H_{1\text{-}3}$), 4.69 – 4.68 (m, 1H, H_6), 3.03 – 2.97 (m, 1H, H_4), 2.45 (bs, 1H, OH), 2.25 - 2.21 (m, 2H, H_7), 1.55 – 1.50 (m, 2H, H_8), 1.44 (d, *J* = 7.1 Hz, 3H, H_5), 1.40 – 1.27 (m, 4H, $H_{9,10}$), 0.92 – 0.88 (m, 3H, H_{11}).

^{13}C NMR (126 MHz, $CDCl_3$) δ 200.6 (C), 134.6 (2 × CH), 129.6 (CH), 129.3 (2 × CH), 127.2 (C), 87.2 (C), 78.3 (C), 64.0 (CH), 53.9 (CH), 31.1 (CH_2), 28.3 (CH_2), 22.2 (CH_2), 18.7 (CH_2), 14.0 (CH_3), 12.7 (CH_3).

Minor diastereomer (detectable non-overlapping resonances):

^{1}H NMR (500 MHz, $CDCl_3$) δ 4.57 - 4.55 (m, 1H, H_6).

^{13}C NMR (126 MHz, $CDCl_3$) δ 134.5 (2 × CH), 129.5 (CH), 129.2 (2 × CH), 127.3 (C), 87.5 (C), 78.7 (C), 64.8 (CH), 54.5 (CH), 15.0 (CH_3).

HPLC (AD-H, *n*-hexane/EtOH=90:10, 1.0 mL/min, 298 K, 254 nm): t_R(anti, major) = 7.0 min, t_R(anti, minor) = 7.6 min, t_R(syn, minor) = 8.5 min, t_R(syn, major) = 9.5 min, d.r. (syn:anti) = 90:10, e.r.(syn) = 98:2 (96% *ee*), e.r.(anti) = 85:15 (70% *ee*).
HRMS-CI (m/z): $[M + Na]^+$ calcd for $C_{17}H_{22}O_2SNa$, 313.1238; found, 313.1230.
R_f = 0.63 (20% ethyl acetate-hexane; UV).

S-phenyl (2R,3S)-3-hydroxy-2-methyldec-4-ynethioate **6u**

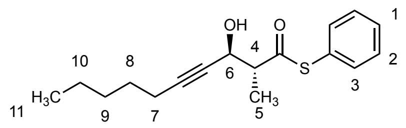

Following general method **B**, **6u** was prepared from 2-octynal (143 μL, 1.00 mmol, 1 equiv) and MAHT **2** (252 mg, 1.20 mmol, 1.20 equiv). The reaction was stirred for 24 h. The residue was purified by column chromatography (eluting with 5% ethyl acetate–hexane initially, grading to 40% ethyl acetate–hexane). The product was obtained as a yellow oil (282 mg, 97%, 33:67 d.r. (syn:anti)).

Major diastereomer:

^{1}H NMR (500 MHz, $CDCl_3$) δ 7.45 – 7.40 (m, 5H, $H_{1\text{-}3}$), 4.58 – 4.53 (m, 1H, H_6), 3.05 – 2.97 (m, 1H, H_4), 2.45 (bs, 1H, OH), 2.25 – 2.20 (m, 2H, H_7), 1.57 – 1.48 (m, 2H, H_8), 1.42 – 1.28 (m, 7H, $H_{5,9,10}$), 0.94 – 0.84 (m, 3H, H_{11}).

^{13}C NMR (126 MHz, $CDCl_3$) δ 200.75 (C), 134.6 (2 × CH), 129.66 (2 × CH), 129.35 (CH), 127.4 (C), 87.7 (C), 78.8 (C), 64.9 (CH), 54.6 (CH), 31.2 (CH_2), 28.4 (CH_2), 22.3 (CH_2), 18.8 (CH_2), 15.2 (CH_3), 14.1 (CH_3).

Minor diastereomer (detectable non-overlapping resonances):

^{1}H NMR (500 MHz, $CDCl_3$) δ 4.71 – 4.67 (m, 1H, H_6), 1.44 (d, *J* = 7.2 Hz, 3H, H_5).

^{13}C NMR (126 MHz, $CDCl_3$) δ 200.80 (C), 134.7 (2 × CH), 129.69 (2 × CH), 129.38 (CH), 127.3 (C), 87.4 (C), 78.3 (C), 64.1 (CH), 53.9 (CH), 12.8 (CH_3).

HPLC (AD-H, n-hexane/EtOH=90:10, 1.0 mL/min, 298 K, 254 nm): t_R(anti, minor) = 7.0 min, t_R(anti, major) = 7.6 min, t_R(syn, major) = 8.5 min, t_R(syn, minor) = 9.5 min, d.r. (syn:anti) = 33:67, e.r.(syn) = 93:7 (86% *ee*), e.r.(anti) = 99:1 (98% *ee*).
HRMS-CI (m/z): $[M + Na]^+$ calcd for $C_{17}H_{22}O_2SNa$, 313.1238; found, 313.1215.
R_f = 0.63 (20% ethyl acetate-hexane; UV).

S-phenyl (2S,3R,E)-3-hydroxy-2-methyl-5-phenylpent-4-enethioate **5v**

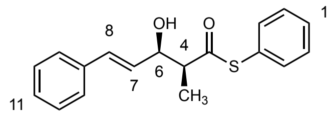

Following general method **A**, **5v** was prepared from cinnamaldehyde (265 μL, 2.00 mmol, 1 equiv) and MAHT **2** (504 mg, 2.40 mmol, 1.20 equiv) in toluene (5.00 mL, 0.40 M). The reaction was stirred for 48 h. The residue was purified by column chromatography (eluting with 5% ethyl acetate–hexane initially, grading to 40% ethyl acetate–hexane). The product was obtained as a white solid (337 mg, 56%, 86:14 d.r. (syn:anti)).

Major diastereomer:

^{1}H NMR (500 MHz, $CDCl_3$) δ 7.45 – 7.20 (m, 10H, $H_{1\text{-}3,\,9\text{-}11}$), 6.69 – 6.62 (m, 1H, H_8), 6.21 (dd, *J* = 15.9, 6.2 Hz, 1H, H_7), 4.64 (t, *J* = 5.3 Hz, 1H, H_6), 3.06 – 2.94 (m, 1H, H_4), 2.58 (bs, 1H, OH), 1.35 (d, *J* = 7.1 Hz, 3H, H_5).

^{13}C NMR (126 MHz, $CDCl_3$) δ 201.7 (C), 136.6 (C), 134.6 (2 × CH), 132.0 (CH), 129.7 (CH), 129.4 (2 × CH), 128.7 (2 × CH), 128.5 (CH), 128.0 (CH), 127.2 (C), 126.7 (2 × CH), 73.3 (CH), 53.5 (CH), 12.4 (CH_3).

Minor diastereomer (detectable non-overlapping resonances):

^{1}H NMR (500 MHz, $CDCl_3$) δ 4.47 (t, *J* = 7.1 Hz, 1H, H_6), 1.31 (d, *J* = 7.1 Hz, 3H, H_5).

^{13}C NMR (126 MHz, $CDCl_3$) δ 201.5 (C), 136.4 (C), 134.6 (2 × CH), 132.5 (CH), 129.6 (CH), 129.3 (2 × CH), 129.1 (CH), 128.7 (2 × CH), 128.1 (CH), 127.4 (C), 126.7 (2 × CH), 75.1 (CH), 53.9 (CH), 15.2 (CH_3).

HPLC (AD-H, *n*-hexane/^{i}PrOH=96:4, 1.0 mL/min, 298 K, 254 nm): t_R(syn, major) = 37.9 min, t_R(syn, minor) = 41.5 min, t_R(anti, major) = 44.7 min, t_R(anti, minor) = 57.7 min, d.r. (syn:anti) = 86:14, e.r.(syn) = 98:2 (96% *ee*), e.r.(anti) = 90:10 (80% *ee*).

HRMS-CI (m/z): $[M + Na]^+$ calcd for $C_{18}H_{18}O_2SNa$, 321.0925; found, 321.0918.
R_f = 0.39 (20% ethyl acetate-hexane; UV).

S-phenyl (2R,3R,E)-3-hydroxy-2-methyl-5-phenylpent-4-enethioate **6v**

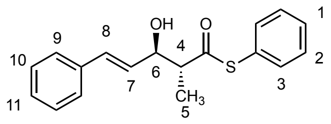

Following general method **B**, **6v** was prepared from cinnamaldehyde (265 μL, 2.00 mmol, 1 equiv) and MAHT **2** (504 mg, 2.40 mmol, 1.20 equiv) in toluene (5.00 mL, 0.40 M). The reaction was stirred for 48 h. The residue was purified by column chromatography (eluting with 5% ethyl acetate–hexane initially, grading to 40% ethyl acetate–hexane). The product was obtained as a white solid (529 mg, 89%, 15:85 d.r. (syn:anti)).

Major diastereomer:

^{1}H NMR (500 MHz, $CDCl_3$) δ 7.44 – 7.24 (m, 10H, $H_{1-3,9,10}$), 6.74 – 6.61 (m, 1H, H_8), 6.29 – 6.17 (m, 1H, H_7), 4.54 – 4.42 (m, 1H, H_6), 3.06 – 2.93 (m, 1H, H_4), 2.42 (bs, 1H, OH), 1.33 (d, *J* = 7.1 Hz, 3H, H_5).

^{13}C NMR (126 MHz, $CDCl_3$) δ 201.5 (C), 136.5 (C), 134.6 (2 × CH), 132.5 (CH), 129.66 (CH), 129.37 (2 × CH), 129.2 (CH), 128.8 (2 × CH), 128.1 (CH), 127.4 (C), 126.8 (2 × CH), 75.1 (CH), 53.9 (CH), 15.2 (CH_3).

Minor diastereomer (detectable non-overlapping resonances):

^{1}H NMR (500 MHz, $CDCl_3$) δ 4.67 (ddd, *J* = 6.0, 4.2, 1.4 Hz, 1H, H_6), 1.37 (d, *J* = 7.1 Hz, 1H, H_5).

^{13}C NMR (126 MHz, $CDCl_3$) δ 201.7 (C), 136.6 (C), 134.7 (2 × CH), 132.0 (CH), 129.73 (CH), 129.40 (2 × CH), 128.7 (2 × CH), 128.5 (CH), 128.0 (CH), 127.3 (C), 126.7 (2 × CH), 73.3 (CH), 53.5 (CH), 12.4 (CH_3).

HPLC (AD-H, *n*-hexane/EtOH=96:4, 1.0 mL/min, 298 K, 254 nm): t_R(syn, minor) = 38.2 min, t_R(syn, major) = 41.3 min, t_R(anti, minor) = 44.7 min, t_R(anti, major) = 57.2 min, d.r. (syn:anti) = 15:85, e.r.(syn) = 90:10 (80% *ee*), e.r.(anti) = 99:1 (98% *ee*).

HRMS-CI (m/z): $[M + Na]^+$ calcd for $C_{18}H_{18}O_2SNa$, 321.0925; found, 321.0909.
R_f = 0.39 (20% ethyl acetate-hexane; UV).

S-phenyl (2S,3S)-3-hydroxy-3-(4-methoxyphenyl)-2-methylpropanethioate **S5a**

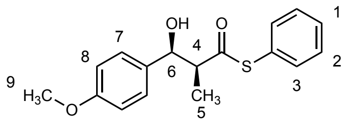

Following general method **A**, **S5a** was prepared from *p*-anisaldehyde (272 mg, 2.00 mmol, 1 equiv) and MAHT **2** (505 mg, 2.40 mmol, 1.20 equiv) in toluene (5.00 mL, 0.40 M). The reaction was stirred for 48 h. The residue was purified by column chromatography (eluting with 5% ethyl acetate–hexane initially, grading to 40% ethyl acetate–hexane). The product was obtained as a colorless oil (104 mg, 17%, 95:5 d.r. (syn:anti)).

Major diastereomer:

^{1}H NMR (500 MHz, $CDCl_3$) δ 7.45 – 7.37 (m, 3H, $H_{1,2}$), 7.37 – 7.31 (m, 2H, H_3), 7.31 – 7.27 (m, 2H, H_7), 6.93 – 6.88 (m, 2H, H_8), 5.07 (d, *J* = 4.5 Hz, 1H, H_6), 3.82 (s, 3H, H_9), 3.06 – 3.00 (m, 1H, H_4), 2.68 (bs, 1H, OH), 1.28 (d, *J* = 7.1 Hz, 3H, H_5).

^{13}C NMR (126 MHz, $CDCl_3$) δ 202.1 (C), 159.3 (C), 134.6 (2 × CH), 133.4 (C), 129.7 (CH), 129.4 (2 × CH), 127.5 (2 × CH), 127.3 (C), 113.9 (2 × CH), 73.8 (CH), 55.4 (CH_3), 55.2 (CH), 12.1 (CH_3).

Minor diastereomer (detectable non-overlapping resonances):

^{1}H NMR (500 MHz, $CDCl_3$) δ 4.81 (d, *J* = 8.5 Hz, 1H, H_6), 3.76 (s, 3H, H_9), 3.13 – 3.06 (m, 1H, H_4), 1.08 (d, *J* = 7.1 Hz, 3H, H_5).

^{13}C NMR (126 MHz, $CDCl_3$) δ 129.6 (CH), 128.0 (2 × CH), 114.1 (2 × CH), 76.4 (CH), 55.6 (CH_3), 15.6(CH_3).

HPLC (AD-H, *n*-hexane/^{i}PrOH=90:10, 1.0 mL/min, 298 K, 254 nm): t_R(syn, minor) = 11.4 min, t_R(syn, major) = 12.9 min, t_R(anti, major) = 19.3 min, t_R(anti, minor) = 20.3 min, d.r. (syn:anti) = 95:5, e.r.(syn) = 98:2 (96% *ee*), e.r.(anti) = 92:8 (84% *ee*).

HRMS-CI (m/z): $[M + Na]^+$ calcd for $C_{17}H_{18}O_3SNa$, 325.0874; found, 325.0870.
R_f = 0.30 (20% ethyl acetate-hexane; UV).

S-phenyl (2R,3S)-3-hydroxy-3-(4-methoxyphenyl)-2-methylpropanethioate **S6a**

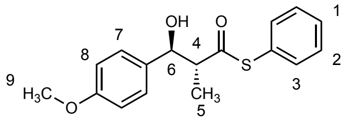

Following general method **B**, **S6a** was prepared from *p*-anisaldehyde (272 mg, 2.00 mmol, 1 equiv) and MAHT **2** (505 mg, 2.40 mmol, 1.20 equiv) in toluene (5.00 mL, 0.40 M). The reaction was stirred for 48 h. The residue was purified by column chromatography (eluting with 5% ethyl acetate–hexane initially, grading to 40% ethyl acetate–hexane). The product was obtained as a white solid (136 mg, 23%, 21:79 d.r. (syn:anti)).

Major diastereomer:

^{1}H NMR (500 MHz, $CDCl_3$) δ 7.42 (s, 5H, $H_{1\text{-}3}$), 7.31 – 7.27 (m, 2H, H_7), 6.93 – 6.89 (m, 2H, H_8), 4.81 (d, *J* = 8.5 Hz, 1H, H_6), 3.82 (s, 3H, H_9), 3.10 (dq, *J* = 8.5, 7.1 Hz, 1H, H_4), 1.09 (d, *J* = 7.1 Hz, 3H, H_5).

^{13}C NMR (126 MHz, $CDCl_3$) δ 201.8 (C), 159.7(C), 134.6 (2 × CH), 133.8 (C), 129.6 (CH), 129.35 (2 × CH), 128.0 (2 × CH), 127.6 (C), 114.1 (2 × CH), 76.4 (CH), 55.6 (CH), 55.5 (CH_3), 15.6 (CH_3).

Minor diastereomer (detectable non-overlapping resonances):

^{1}H NMR (500 MHz, $CDCl_3$) δ 5.08 (d, *J* = 4.6 Hz, 1H, H_6), 3.03 (qd, *J* = 7.0, 4.6 Hz, 1H, H_4), 1.28 (d, *J* = 7.0 Hz, 3H, H_5).

^{13}C NMR (126 MHz, $CDCl_3$) δ 202.1 (C), 159.3 (C), 133.4 (C), 127.3 (C), 129.7 (CH), 129.37 (2 × CH), 127.5 (2 × CH), 113.9 (2 × CH), 73.8 (CH), 55.4 (CH_3), 55.2 (CH), 12.1 (CH_3).

HPLC (AD-H, *n*-hexane/^{i}PrOH=90:10, 1.0 mL/min, 298 K, 254 nm): t_R(syn, major) = 11.3 min, t_R(syn, minor) = 12.9 min, t_R(anti, minor) = 19.3 min, t_R(anti, major) = 20.3 min, d.r. (syn:anti) = 21:79, e.r.(syn) = 79:21 (57% *ee*), e.r.(anti) = 98:2 (96% *ee*).

HRMS-CI (m/z): $[M + Na]^+$ calcd for $C_{17}H_{18}O_3SNa$, 325.0874; found, 325.0857.

R_f = 0.30 (20% ethyl acetate-hexane; UV).

S-phenyl (2S,3S)-3-(2-chlorophenyl)-3-hydroxy-2-methylpropanethioate **S5b**

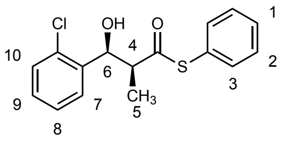

Following general method **A**, **S5b** was prepared from 2-chlorobenzaldehyde (225 μL, 2.00 mmol, 1 equiv) and MAHT **2** (505 mg, 2.40 mmol, 1.20 equiv) in toluene (5.00 mL, 0.40 M). The reaction was stirred for 48 h. The residue was purified by column chromatography (eluting with 5% ethyl acetate–hexane initially, grading to 40% ethyl acetate–hexane). The product was obtained as colorless oil (607 mg, 99%, 92:8 d.r. (syn:anti)).

Major diastereomer:

^{1}H NMR (500 MHz, $CDCl_3$) δ 7.61 (dd, *J* = 7.7, 1.8 Hz, 1H, H_{10}), 7.43 (s, 5H, $H_{1,2,3}$), 7.35 (dd, *J* = 7.9, 1.4 Hz, 1H, H_9), 7.31 (td, *J* = 7.5, 1.4 Hz, 1H, H_7), 7.27 – 7.20 (m, 1H, H_8), 5.57 (t, *J* = 2.5 Hz, 1H, H_6), 3.28 (qd, *J* = 7.2, 2.4 Hz, 1H, H_4), 3.06 (d, *J* = 2.6 Hz, 1H, OH), 1.17 (d, *J* = 7.2 Hz, 3H, H_5).

^{13}C NMR (126 MHz, $CDCl_3$) δ 203.0 (C), 138.0 (C), 134.7 (2 × CH), 131.4 (C), 129.8 (CH), 129.6 (CH), 129.4 (2 × CH), 128.8 (CH), 128.5 (CH), 127.1 (C), 126.90 (CH), 70.2 (CH), 50.9 (CH), 10.5 (CH_3).

Minor diastereomer (detectable non-overlapping resonances):

^{1}H NMR (500 MHz, $CDCl_3$) δ 5.31 (s, 1H, H_6), 3.19 (d, *J* = 6.6 Hz, 1H, OH), 1.29 (d, *J* = 7.2 Hz, 3H, H_5).

^{13}C NMR (126 MHz, $CDCl_3$) δ 202.2 (C), 139.3 (C), 134.5 (2 × CH), 132.5 (C), 129.7 (CH), 129.7 (CH), 129.3 (2 × CH), 129.1 (CH), 128.0 (CH), 127.3 (CH), 72.9 (CH), 53.7 (CH), 15.5 (CH_3).

HPLC (AD-H, *n*-hexane/EtOH=90:10, 1.0 mL/min, 298 K, 254 nm): t_R(syn, major) = 7.1 min, t_R(syn, minor) = 8.4 min, t_R(anti, major) = 9.7 min, t_R(anti, minor) = 11.7 min, d.r. (syn:anti) = 92:8, e.r.(syn) = 97:3 (94% *ee*), e.r.(anti) = 90:10 (80% *ee*).

HRMS-CI (m/z): $[M + Na]^+$ calcd for $C_{16}H_{15}ClO_2SNa$, 329.0379; found, 329.0374

R_f = 0.45 (20% ethyl acetate-hexane; UV).

S-phenyl (2R,3S)-3-(2-chlorophenyl)-3-hydroxy-2-methylpropanethioate **S6b**

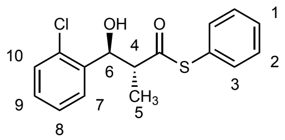

Following general method **B**, **S6b** was prepared from 2-chlorobenzaldehyde (225 µL, 2.00 mmol, 1 equiv) and MAHT **2** (505 mg, 2.40 mmol, 1.20 equiv) in toluene (5.00 mL, 0.40 M). The reaction was stirred for 48 h. The residue was purified by column chromatography (eluting with 5% ethyl acetate–hexane initially, grading to 40% ethyl acetate–hexane). The product was obtained as colorless oil (559 mg, 91%, 30:70 d.r. (syn:anti).

Major diastereomer:

^{1}H NMR (500 MHz, $CDCl_3$) δ 7.50 – 7.22 (m, 9H, $H_{1\text{-}3,7\text{-}10}$), 5.31 (d, *J* = 6.5 Hz, 1H, H_6), 3.34 – 3.24 (m, 1H, H_4), 1.31 (d, *J* = 7.2 Hz, 3H, H_5).

^{13}C NMR (126 MHz, $CDCl_3$) δ 202.3 (C), 139.4 (C), 134.6 (2 × CH), 132.5 (C), 129.77 (2 × CH), 129.7 (CH), 129.4 (CH), 129.2 (CH), 128.0 (CH), 127.3 (CH), 127.21 (C), 73.0 (CH), 53.6 (CH), 15.5 (CH_3).

Minor diastereomer (detectable non-overlapping resonances):

^{1}H NMR (500 MHz, $CDCl_3$) δ 7.62 (dd, *J* = 7.7, 1.7 Hz, 1H, H_{10}), 5.58 (d, *J* = 2.3 Hz, 1H, H_6), 1.18 (d, *J* = 7.2 Hz, 2H, H_5).

^{13}C NMR (126 MHz, $CDCl_3$) δ 203.1 (C), 138.0 (C), 134.7 (2 × CH), 131.5 (C), 129.82 (2 × CH), 129.6 (CH), 129.5 (CH), 128.9 (CH), 128.6 (CH), 127.15 (C), 126.9 (CH), 70.3 (CH), 50.9 (CH), 10.5 (CH_3).

HPLC (AD-H, *n*-hexane/EtOH=90:10, 1.0 mL/min, 298 K, 254 nm): t_R(syn, minor) = 7.1 min, t_R(syn, major) = 8.4 min, t_R(anti, minor) = 9.7 min, t_R(anti, major) = 11.7 min, d.r. (syn:anti) = 30:70, e.r.(syn) = 63:37 (25% *ee*), e.r.(anti) = 95:5 (89% *ee*).

HRMS-CI (m/z): $[M + Na]^+$ calcd for $C_{16}H_{15}ClO_2SNa$, 329.0379; found, 329.0358.

R_f = 0.45 (20% ethyl acetate-hexane; UV).

S-phenyl (2S,3S)-3-hydroxy-2-methyl-3-(thiazol-2-yl)propanethioate **S5c**

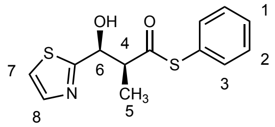

Following general method **A**, **S5c** was prepared from 2-thiazolecarboxaldehyde (89 µL, 1.00 mmol, 1 equiv) and MAHT **2** (252 mg, 1.20 mmol, 1.20 equiv). The reaction was stirred for 24 h. The residue was purified by column chromatography (eluting with 5% ethyl acetate–hexane initially, grading to 50% ethyl acetate–hexane). The product was obtained as a yellow oil (257 mg, 92%, 84:16 d.r. (syn:anti)).

Major diastereomer:

^{1}H NMR (500 MHz, $CDCl_3$) δ 7.79 (d, *J* = 3.1 Hz, 1H, H_8), 7.45 – 7.38 (m, 5H, $H_{1\text{-}3}$), 7.33 – 7.30 (m, 1H, H_7), 5.48 (d, *J* = 3.0 Hz, 1H, H_6), 3.56 (qd, *J* = 7.2, 3.0 Hz, 1H, H_4), 1.27 (d, *J* = 7.2 Hz, 3H, H_5).

^{13}C NMR (126 MHz, $CDCl_3$) δ 201.2 (C), 172.9 (C), 142.5 (CH), 134.5 (2 × CH), 129.51 (CH), 129.19 (2 × CH), 127.0 (C), 119.3 (CH), 71.8 (CH), 53.3 (CH), 11.3 (CH_3).

Minor diastereomer (detectable non-overlapping resonances):

^{1}H NMR (500 MHz, $CDCl_3$) δ 5.11 (d, J = 5.4 Hz, 1H, H_6), 1.43 (d, *J* = 7.2 Hz, 3H, H_5).

^{13}C NMR (126 MHz, $CDCl_3$) δ 173.5 (C), 142.4 (CH), 134.4 (2 × CH), 129.47 (CH), 129.15 (2 × CH), 127.1 (C), 119.6 (CH), 73.7 (CH), 53.8 (CH), 15.0 (CH_3),

HPLC (AD-H, *n*-hexane/^{i}PrOH=90:10, 1.0 mL/min, 298 K, 254 nm): t_R(syn, minor) = 17.2 min, t_R(syn, major) = 18.1 min, t_R(anti, minor) = 21.3 min, t_R(anti, major) = 33.5 min, d.r. (syn:anti) = 84:16, e.r.(syn) = 97:3 (94% *ee*), e.r.(anti) = 96:4 (92% *ee*).

HRMS-CI (m/z): $[M + H]^+$ calcd for $C_{13}H_{14}NO_2S_2$, 280.0466; found, 280.0459.

R_f = 0.19 (20% ethyl acetate-hexane; UV).

The absolute stereochemistry for **S5c** was confirmed by X-ray crystallography (see **fig. S4**). The crystal was grown by a slow evaporation of dichloromethane.

S-phenyl (2R,3S)-3-hydroxy-2-methyl-3-(thiazol-2-yl)propanethioate **S6c**

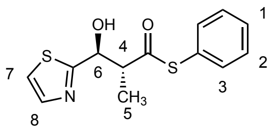

Following general method **B**, **S6c** was prepared from 2-thiazolecarboxaldehyde (89 µL, 1.00 mmol, 1 equiv) and MAHT **2** (252 mg, 1.20 mmol, 1.20 equiv). The reaction was stirred for 24 h. The residue was purified by column chromatography (eluting with 5% ethyl acetate–hexane initially, grading to 50% ethyl acetate–hexane). The product was obtained as a white solid (267 mg, 96%, 26:74 d.r. (syn:anti)).

Major diastereomer:

^{1}H NMR (500 MHz, $CDCl_3$) δ 7.81 – 7.77 (m, 1H, H_8), 7.47 – 7.30 (m, 6H, $H_{1\text{-}3,7}$), 5.11 (dd, *J* = 8.0, 5.5 Hz, 1H, H_6), 3.95 (d, *J* = 8.0 Hz, 1H, OH), 3.59 – 3.48 (m, 1H, H_4), 1.43 (d, *J* = 7.2 Hz, 3H, H_5).

^{13}C NMR (126 MHz, $CDCl_3$) δ 202.1 (C), 173.0 (C), 142.61 (CH), 134.5 (2 × CH), 129.7 (CH), 129.27 (2 × CH), 126.9 (C), 119.6 (CH), 74.3 (CH), 53.1 (CH), 15.3 (CH_3).

Minor diastereomer (detectable non-overlapping resonances):

^{1}H NMR (500 MHz, $CDCl_3$) δ 5.49 – 5.45 (m, 1H, H_6), 1.27 (d, *J* = 7.2 Hz, 3H, H_5).

^{13}C NMR (126 MHz, $CDCl_3$) δ 202.3 (C), 171.8 (C), 142.59 (CH), 134.6 (2 × CH), 129.8 (CH), 129.34 (2 × CH), 126.8 (C), 119.3 (CH), 72.0 (CH), 52.9 (CH), 11.4 (CH_3).

HPLC (AD-H, *n*-hexane/^{i}PrOH=90:10, 1.0 mL/min, 298 K, 254 nm): t_R(syn, major) = 17.1 min, t_R(syn, minor) = 18.0 min, t_R(anti, major) = 21.1 min, t_R(anti, minor) = 33.3 min, d.r. (syn:anti) = 26:74, e.r.(syn) = 78:22 (56% *ee*), e.r.(anti) = 94:6 (88% *ee*).

HRMS-CI (m/z): $[M + H]^+$ calcd for $C_{13}H_{14}NO_2S_2$, 280.0466; found, 280.0446.

R_f = 0.19 (20% ethyl acetate-hexane; UV).

S-phenyl (2S,3S)-3-hydroxy-2-methyl-3-(5-phenylfuran-2-yl)propanethioate **S5d**

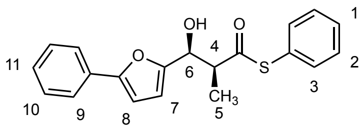

Following general method **A**, **S5d** was prepared from 5-phenyl-2-furaldehyde (149 µL, 1.00 mmol, 1 equiv) and MAHT **2** (252 mg, 1.20 mmol, 1.20 equiv). The reaction was stirred for 24 h. The residue was purified by column chromatography (eluting with 5% ethyl acetate–hexane initially, grading to 50% ethyl acetate–hexane). The product was obtained as an orange oil (287 mg, 85%, 94:6 d.r. (syn:anti)).

Major diastereomer:

^{1}H NMR (500 MHz, $CDCl_3$) δ 7.56 – 7.51 (m, 2H, H_9), 7.31 – 7.18 (m, 7H, $H_{2,3,10,11}$), 7.16 – 7.09 (m, 1H, H_1), 6.47 (d, *J* = 3.4 Hz, 1H, H_8), 6.27 (dd, *J* = 3.4, 0.8 Hz, 1H, H_7), 4.95 (d, *J* = 5.6 Hz, 1H, H_6), 3.20 (m, 1H, H_4), 2.95 (bs, 1H, OH), 1.27 (d, *J* = 7.1 Hz, 3H, H_5).

^{13}C NMR (126 MHz, $CDCl_3$) δ 200.9 (C), 153.5 (C), 153.4 (C), 134.6 (2 × CH), 130.7 (C), 129.6 (CH), 129.2 (2 × CH), 128.7 (2 × CH), 127.4 (CH), 127.1 (C), 123.8 (2 × CH), 109.4 (CH), 105.7 (CH), 69.1 (CH), 52.6 (CH), 13.00 (CH_3).

Minor diastereomer (detectable non-overlapping resonances):

^{1}H NMR (500 MHz, $CDCl_3$) δ 4.77 (d, *J* = 7.7 Hz, 1H, H_6), 3.31 – 3.25 (m, 1H, H_4), 1.12 (d, *J* = 7.1 Hz, 3H, H_5).

^{13}C NMR (126 MHz, $CDCl_3$) δ 201.4 (C), 153.8 (C), 153.6 (C), 134.5 (2 × CH), 123.9 (2 × CH), 109.9 (CH), 105.6 (CH), 70.2 (CH), 52.7 (CH), 15.3 (CH_3).

HPLC (AD-H, *n*-hexane/^{i}PrOH=92:8, 1.0 mL/min, 298 K, 254 nm): t_R(syn, major) = 13.8 min, t_R(syn, minor) = 14.6 min, t_R(anti, major) = 20.4 min, t_R(anti, minor) = 23.4 min, d.r. (syn:anti) = 94:6, e.r.(syn) = 95:5 (90% *ee*), e.r.(anti) = 90:10 (80% *ee*).

HRMS-CI (m/z): $[M + Na]^+$ calcd for $C_{20}H_{18}O_3SNa$, 361.0874; found, 361.0876.

R_f = 0.32 (20% ethyl acetate-hexane; UV).

S-phenyl (2R,3S)-3-hydroxy-2-methyl-3-(5-phenylfuran-2-yl)propanethioate **S6d**

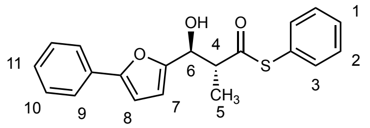

Following general method **B**, **S6d** was prepared from 5-phenyl-2-furaldehyde (149 µL, 1.00 mmol, 1 equiv) and MAHT **2** (252 mg, 1.20 mmol, 1.20 equiv). The reaction was stirred for 24 h. The residue was purified by column chromatography (eluting with 5% ethyl acetate–hexane initially, grading to 50% ethyl acetate–hexane). The product was obtained as an orange oil (294 mg, 87%, 21:79 d.r. (syn:anti)).

Major diastereomer:

^{1}H NMR (500 MHz, $CDCl_3$) δ 7.71 – 7.63 (m, 2H, H_9), 7.46 – 7.34 (m, 7H, $H_{2,3,10,11}$), 7.31 – 7.25 (m, 1H, H_1), 6.64 – 6.60 (m, 1H, H_8), 6.45 – 6.39 (m, 1H, H_7), 4.93 – 4.89 (m, 1H, H_6), 3.46 – 3.38 (m, 1H, H_4), 2.86 (d, *J* = 6.7 Hz, 1H, OH), 1.29 (d, *J* = 7.1 Hz, 3H, H_5).

^{13}C NMR (126 MHz, $CDCl_3$) δ 201.6 (C), 154.0 (C), 153.66 (C), 134.66 (2 × CH), 130.7 (C), 129.71 (CH), 129.4 (2 × CH), 128.8 (2 × CH), 127.7 (CH), 127.3 (C), 124.0 (2 × CH), 110.0 (CH), 105.7 (CH), 70.5 (CH), 52.7 (CH), 15.5 (CH_3).

Minor diastereomer (detectable non-overlapping resonances):

^{1}H NMR (500 MHz, $CDCl_3$) δ 5.16 – 5.10 (m, 1H, H_6), 3.35 (qd, *J* = 7.1, 5.1 Hz, 1H, H_4), 2.76 (d, *J* = 4.8 Hz, 1H, OH), 1.42 (d, *J* = 7.1 Hz, 3H, H_5).

^{13}C NMR (126 MHz, $CDCl_3$) δ 201.3 (C), 153.71 (C), 153.34 (C), 134.70 (2 × CH), 130.8 (C), 129.74 (CH), 127.6 (CH), 127.2 (C), 123.9 (2 × CH), 109.6 (CH), 105.8 (CH), 69.3 (CH), 52.6 (CH), 12.9 (CH_3).

HPLC (AD-H, *n*-hexane/^{i}PrOH=92:8, 1.0 mL/min, 298 K, 254 nm): t_R(syn, minor) = 13.8 min, t_R(syn, major) = 14.6 min, t_R(anti, minor) = 20.4 min, t_R(anti, major) = 23.3 min, d.r. (syn:anti) = 21:79, e.r.(syn) = 85:15 (70% *ee*), e.r.(anti) = 99:1 (98% *ee*).

HRMS-CI (m/z): $[M + Na]^+$ calcd for $C_{20}H_{18}O_3SNa$, 361.0874; found, 361.0851.

R_f = 0.32 (20% ethyl acetate-hexane; UV).

S-phenyl (2S,3R)-3-cyclohexyl-3-hydroxy-2-methylpropanethioate **S5e**

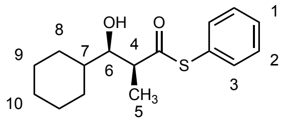

Following general method **A**, **S5e** was prepared from cyclohexanecarboxaldehyde (121 µL, 1.00 mmol, 1.0 equiv) and MAHT **2** (252 mg, 1.20 mmol, 1.20 equiv). The reaction was stirred for 48 h. The residue was purified by column chromatography (eluting with 5% ethyl acetate–hexane initially, grading to 40% ethyl acetate–hexane). The product was obtained as a colorless liquid (213 mg, 77%, 91:9 d.r. (syn:anti)).

^{1}H and ^{13}C NMR data for **S5e** prepared in this way were in agreement with the literature.(*24*)

Major diastereomer:

^{1}H NMR (500 MHz, $CDCl_3$) δ 7.43- 7.41 (m, 5H, $H_{1\text{-}3}$), 3.71 (dd, *J* = 7.8, 3.5 Hz, 1H, H_6), 2.98 (qd, *J* = 7.1, 3.5 Hz, 1H, H_4), 2.11 – 2.05 (m, 1H, H_8), 1.82 – 1.73 (m, 2H, H_9), 1.71 – 1.64 (m, 1H, H_{10}), 1.61 – 1.56 (m, 1H, H_8), 1.47 – 1.39 (m, 1H, H_7), 1.28 (d, *J* = 7.1 Hz, 3H, H_5), 1.27 – 1.12 (m, 3H, $H_{9,10}$), 1.09 – 0.96 (m, 2H, H_8).

^{13}C NMR (126 MHz, $CDCl_3$) δ 202.3 (C), 134.5 (2 × CH), 129.4 (CH), 129.1 (2 × CH), 127.35 (C), 75.9 (CH), 49.9 (CH), 40.3 (CH), 29.2 (CH_2), 28.7 (CH_2), 26.3 (CH_2), 26.1 (CH_2), 25.8 (CH_2), 11.2 (CH_3).

Minor diastereomer (detectable non-overlapping resonances):

^{1}H NMR (500 MHz, $CDCl_3$) δ 3.45 – 3.43 (m, 1H, H_6), 1.34 (d, *J* = 7.1 Hz, 3H, H_5).

^{13}C NMR (126 MHz, $CDCl_3$) δ 202.6 (C), 134.4 (2 × CH), 127.38 (C), 78.2 (CH), 50.23 (CH), 41.2 (CH), 30.1 (CH_2), 27.1 (CH_2), 26.4 (CH_2), 26.0 (CH_2), 15.8 (CH_3).

HPLC (AD-H, n-hexane/^{i}PrOH=90:10, 1.0 mL/min, 298 K, 254 nm): t_R(anti, major) = 12.4 min, t_R(anti, minor) = 14.5 min, t_R(syn, minor) = 16.3 min, t_R(syn, major) = 23.3 min, d.r. (syn:anti) = 91:9, e.r.(syn) = 97:3 (95% *ee*), e.r.(anti) = 92:8 (84% *ee*).

HRMS-CI (m/z): $[M + Na]^+$ calcd for $C_{16}H_{22}O_2SNa$, 301.1238; found, 301.1236.
R_f = 0.68 (20% ethyl acetate-hexane; UV)

S-phenyl (2R,3R)-3-cyclohexyl-3-hydroxy-2-methylpropanethioate **S6e**

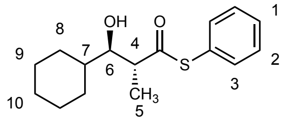

Following general method **B**, **S6e** was prepared from cyclohexanecarboxaldehyde (121 µL, 1.00 mmol, 1 equiv) and MAHT **2** (252 mg, 1.20 mmol, 1.20 equiv). The reaction was stirred for 48 h. The residue was purified by column chromatography (eluting with 5% ethyl acetate–hexane initially, grading to 40% ethyl acetate–hexane). The product was obtained as a colorless oil (210 mg, 75%, 27:73 d.r. (syn:anti)).

Major diastereomer:

^{1}H NMR (500 MHz, $CDCl_3$) δ 7.42 (s, 5H, H_{1-3}), 3.44 (d, *J* = 5.8, 5.8 Hz, 1H, H_6), 3.03 (qd, *J* = 7.1, 5.8 Hz, 1H, H_4), 2.41 (bs, 1H, OH), 2.12 – 1.56 (m, 6H, H_{8-10}), 1.48 – 1.39 (m, 1H, H_7), 1.34 (d, *J* = 7.1 Hz, 3H, H_5), 1.32 – 0.96 (m, 4H, H_{8-10}).

^{13}C NMR (126 MHz, $CDCl_3$) δ 203.0 (C), 134.6 (2 × CH), 129.7 (CH), 129.4 (2 × CH), 127.5 (C), 78.6 (CH), 50.3 (CH), 41.5 (CH), 30.3 (CH_2), 27.4 (CH_2), 26.51 (CH_2), 26.45 (CH_2), 26.15 (CH_2), 16.1 (CH_3).

Minor diastereomer (detectable non-overlapping resonances):

^{1}H NMR (500 MHz, $CDCl_3$) δ 3.71 (dd, *J* = 7.8, 3.5 Hz, 1H, H_6), 3.00 – 2.95 (m, 1H, H_4), 1.28 (d, *J* = 7.0 Hz, 3H, H_5).

^{13}C NMR (126 MHz, $CDCl_3$) δ 202.8 (C), 134.7 (2 × CH), 127.4 (C), 76.0 (CH), 49.9 (CH), 40.4 (CH), 29.3 (CH_2), 29.1 (CH_2), 26.21 (CH_2), 26.0 (CH_2), 11.2 (CH_3).

HPLC (AD-H, *n*-hexane/EtOH=97:3, 1.0 mL/min, 298 K, 254 nm): t_R(anti, minor) = 12.4 min, t_R(anti, major) = 14.5 min, t_R(syn, major) = 16.3 min, t_R(syn, minor) = 23.2 min, d.r. (syn:anti) = 27:73, e.r.(syn) = 55:45 (10% *ee*), e.r.(anti) = 97:3 (94% *ee*).

HRMS-CI (m/z): $[M + Na]^+$ calcd for $C_{16}H_{22}O_2SNa$, 301.1238; found, 301.1223.
R_f = 0.68 (20% ethyl acetate-hexane; UV).

S-phenyl (2S,3S)-3-hydroxy-2-methyl-5-phenylpent-4-ynethioate **S5f**

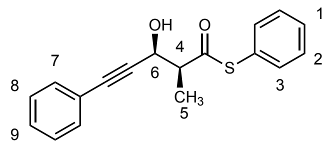

Following general method **A**, **S5f** was prepared from 3-phenylpropiolaldehyde (122 µL, 1.00 mmol, 1 equiv) and MAHT **2** (252 mg, 1.20 mmol, 1.20 equiv). The reaction was stirred for 24 h. The residue was purified by column chromatography (eluting with 5% ethyl acetate–hexane initially, grading to 40% ethyl acetate–hexane). The product was obtained as a colorless liquid (273 mg, 92%, 84:16 d.r. (syn:anti)).

Major diastereomer:

^{1}H NMR (500 MHz, $CDCl_3$) δ 7.48 – 7.44 (m, 2H, $H_{7/8}$), 7.42 (s, 5H, H_{1-3}), 7.36 – 7.29 (m, 3H, $H_{7/8,9}$), 4.93 (d, *J* = 4.6 Hz, 1H, H_6), 3.15 (qd, *J* = 7.1, 4.6 Hz, 1H, H_4), 1.53 (d, *J* = 7.1 Hz, 3H, H_5).

^{13}C NMR (126 MHz, $CDCl_3$) δ 200.6 (C), 134.7 (2 × CH), 131.94 (2 × CH), 129.8 (CH), 129.4 (2 × CH), 128.7 (CH), 128.4 (2 × CH), 127.2 (C), 122.36 (C), 87.1 (C), 86.3 (C), 64.3 (CH), 53.7 (CH), 12.9 (CH_3).

Minor diastereomer (detectable non-overlapping resonances):

^{1}H NMR (500 MHz, $CDCl_3$) δ 4.80 (d, *J* = 7.2 Hz, 1H, H_6), 1.46 (d, *J* = 7.1 Hz, 3H, H_5).

^{13}C NMR (126 MHz, $CDCl_3$) δ 200.7 (C), 134.6 (2 × CH), 131.90 (2 × CH), 129.7 (CH), 129.3 (2 × CH), 128.8 (CH), 128.5 (2 × CH), 127.1 (C), 122.34 (C), 87.6 (C), 86.6 (C), 65.1 (CH), 54.2 (CH), 15.1 (CH_3).

HPLC (AD-H, *n*-hexane/^{i}PrOH=90:10, 1.0 mL/min, 298 K, 254 nm): t_R(syn, minor) = 10.5 min, t_R(syn, major) = 11.9 min, t_R(anti, major) = 12.9 min, t_R(anti, minor) = 16.0 min, d.r. (syn:anti) = 84:16, e.r.(syn) = 96:4 (93% *ee*), e.r.(anti) = 88:12 (76% *ee*).

HRMS-CI (m/z): $[M + Na]^+$ calcd for $C_{18}H_{16}O_2SNa$, 319.0769; found, 319.0762.
R_f = 0.37 (20% ethyl acetate-hexane; UV).

S-phenyl (2R,3S)-3-hydroxy-2-methyl-5-phenylpent-4-ynethioate **S6f**

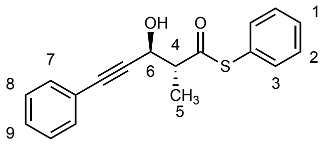

Following general method **B**, **S6f** was prepared from 3-phenylpropionaldehyde (122 µL, 1.00 mmol, 1 equiv) and MAHT **2** (252 mg, 1.20 mmol, 1.20 equiv). The reaction was stirred for 24 h. The residue was purified by column chromatography (eluting with 5% ethyl acetate–hexane initially, grading to 40% ethyl acetate–hexane). The product was obtained as a yellow oil (274 mg, 93%, 51:49 d.r. (syn:anti)).

Major diastereomer (anti-aldol product):

^{1}H NMR (500 MHz, $CDCl_3$) δ 7.48 – 7.41 (m, 7H, H_{arom}), 7.35 – 7.29 (m, 3H, H_{arom}), 4.93 – 4.91 (m, 1H, H_6), 3.24 – 3.08 (m, 1H, H_4), 2.75 (d, *J* = 7.0 Hz, 1H, OH), 1.53 (d, *J* = 7.2 Hz, 3H, H_5).

^{13}C NMR (126 MHz, $CDCl_3$) δ 200.7 (C), δ134.8 (2 × CH), 132.0 (2 × CH), 129.78 (CH), 129.43 (2 × CH), 128.81 (CH), 128.46 (2 × CH), 127.1 (C), 122.38 (C), 87.2 (C), 86.4 (C), 64.3 (CH), 53.8 (CH), 12.9 (CH_3).

Minor diastereomer (detectable non-overlapping resonances, syn-aldol product):

^{1}H NMR (500 MHz, $CDCl_3$) δ 4.92 (dd, *J* = 6.0, 4.5 Hz, 1H, H_6), 2.75 (d, *J* = 6.0 Hz, 1H, OH), 1.53 (d, *J* = 7.1 Hz, 3H, H_5).

^{13}C NMR (126 MHz, $CDCl_3$) 134.6 (2 × CH), 131.9 (2 × CH), 129.75 (CH), 129.41 (2 × CH), 128.84 (CH), 128.48 (2 × CH), 127.3 (C), 122.35 (C), 87.6 (C), 86.7 (C), 65.2 (CH), 54.3 (CH), 15.2 (CH_3).

HPLC (AD-H, *n*-hexane/^{i}PrOH=90:10, 1.0 mL/min, 298 K, 254 nm): t_R(syn, major) = 10.5 min, t_R(syn, minor) = 12.0 min, t_R(anti, minor) = 12.9 min, t_R(anti, major) = 16.0 min, d.r. (syn:anti) = 51:49, e.r.(syn) = 92:08 (84% *ee*), e.r.(anti) = 94:6 (88% *ee*).

HRMS-CI (m/z): $[M + Na]^+$ calcd for $C_{18}H_{16}O_2SNa$, 319.0769; found, 319.0746.

R_f = 0.37 (20% ethyl acetate-hexane; UV).

S-phenyl (2S,3R)-3-hydroxy-2-methyldecanethioate **S5g**

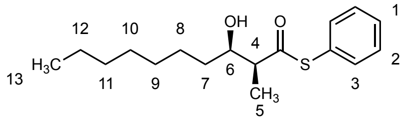

Following general method **A**, **S5g** was prepared from octanal (156 µL, 1.00 mmol, 1 equiv) and MAHT **2** (252 mg, 1.20 mmol, 1.20 equiv). The reaction was stirred for 24 h. The residue was purified by column chromatography (eluting with 5% ethyl acetate–hexane initially, grading to 40% ethyl acetate–hexane). The product was obtained as a colorless liquid (271 mg, 92%, 78:22 d.r. (syn:anti)).

^{1}H and ^{13}C NMR data for **S5g** prepared in this way were in agreement with the literature.(*24*)

Major diastereomer:

^{1}H NMR (500 MHz, $CDCl_3$) δ 7.42 (s, 5H, H_{1-3}), 3.99 – 3.95 (m, 1H, H_6), 2.82 – 2.79 (m, 1H, H_4), 2.30 (bs, 1H, OH), 1.59 – 1.40 (m, 3H, $H_{7,8}$), 1.37-1.29 (m, 12H, $H_{5,8-12}$), 0.92 – 0.85 (m, 3H, H_{13}).

^{13}C NMR (126 MHz, $CDCl_3$) δ 202.2 (C), 134.54 (2 × CH), 129.55 (CH), 129.27 (2 × CH), 127.3 (C), 72.0 (CH), 53.0 (CH), 34.2 (CH_2), 31.8 (CH_2), 29.5 (CH_2), 29.3 (CH_2), 26.0 (CH_2), 22.7 (CH_2), 14.1 (CH_3), 11.7 (CH_3).

Minor diastereomer (detectable non-overlapping resonances):

^{1}H NMR (500 MHz, $CDCl_3$) δ 3.75-3.72 (m, 1H, H_6), 2.90 – 2.85 (m, 1H, H_4).

^{13}C NMR (126 MHz, $CDCl_3$) δ 202.1 (C), 134.50 (2 × CH), 129.53 (CH), 129.25 (2 × CH), 127.4 (C), 73.9 (CH), 53.6 (CH), 34.9 (CH_2), 25.6 (CH_2), 15.2 (CH_3).

HPLC (AD-H, n-hexane/^{i}PrOH=98:2, 1.0 mL/min, 298 K, 254 nm): t_R(anti, major) = 15.1 min, t_R(syn, minor) = 19.1 min, t_R(syn, major) = 20.7 min, t_R(anti, minor) = 27.1 min, d.r. (syn:anti) = 78:12, e.r.(syn) = 99:1 (98% *ee*), e.r.(anti) = 98:2 (94% *ee*).

HRMS-CI (m/z): $[M + Na]^+$ calcd for $C_{17}H_{26}O_2SNa$, 317.1551; found, 317.1535.

R_f = 0.48 (20% ethyl acetate-hexane; UV).

S-phenyl (2R,3R)-3-hydroxy-2-methyldecanethioate **S6g**

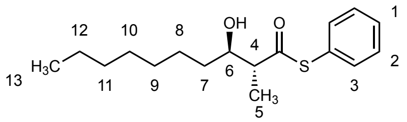

Following general method **B**, **S6g** was prepared from octanal (156 µL, 1.00 mmol, 1 equiv) and MAHT **2** (252 mg, 1.20 mmol, 1.20 equiv). The reaction was stirred for 24 h. The residue was purified by column chromatography (eluting with 5% ethyl acetate–hexane initially, grading to 40% ethyl acetate–hexane). The product was obtained as a colorless oil (276 mg, 94%, 13:87 d.r. (syn:anti)).

Major diastereomer:

^{1}H NMR (500 MHz, CDCl$_3$) δ 7.42 (s, 5H, H_{1-3}), 3.79 – 3.68 (m, 1H, H_6), 2.91 – 2.78 (m, 1H, H_4), 2.33 (bs, 1H, OH), 1.67 – 1.44 (m, 3H, $H_{7,8}$), 1.42 – 1.23 (m, 12H, $H_{5,8-12}$), 0.92 – 0.83 (m, 3H, H_{13}).

^{13}C NMR (126 MHz, CDCl$_3$) δ 202.4 (C), 134.6 (2 × CH), 129.67 (CH), 129.38 (2 × CH), 127.5 (C), 74.1 (CH), 53.6 (CH), 35.1 (CH$_2$), 32.0 (CH$_2$), 29.7 (CH$_2$), 29.4 (CH$_2$), 25.7 (CH$_2$), 22.8 (CH$_2$), 15.5 (CH$_3$), 14.2 (CH$_3$).

Minor diastereomer (detectable non-overlapping resonances):

^{1}H NMR (500 MHz, CDCl$_3$) δ 4.03 – 3.92 (m, 1H, H_6).

^{13}C NMR (126 MHz, CDCl$_3$) δ 202.5 (C), 134.7 (2 × CH), 129.72 (CH), 129.40 (2 × CH), 127.4 (C), 72.0 (CH), 52.9 (CH), 34.2 (CH$_2$), 29.6 (CH$_2$), 26.1 (CH$_2$), 11.6 (CH$_3$).

HPLC (AD-H, *n*-hexane/^{i}PrOH=98:2, 1.0 mL/min, 298 K, 254 nm): t_R(anti, minor) = 15.0 min, t_R(syn, major) = 19.0 min, t_R(syn, minor) = 20.7 min, t_R(anti, major) = 26.8 min, d.r. (syn:anti) = 13:87, e.r.(syn) = 80:20 (60% *ee*), e.r.(anti) = 98:2 (96% *ee*).

HRMS-CI (m/z): $[M + Na]^+$ calcd for $C_{17}H_{26}O_2SNa$, 317.1551; found, 317.1536.

R_f = 0.48 (20% ethyl acetate-hexane; UV).

S-phenyl (2S,3R)-3-hydroxy-2,5-dimethylhexanethioate **S5h**

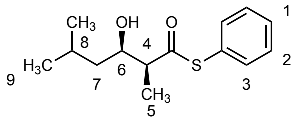

Following general method **A**, **S5h** was prepared from isovaleraldehyde (111 µL, 1.00 mmol, 1 equiv) and MAHT **2** (252 mg, 1.20 mmol, 1.20 equiv). The reaction was stirred for 24 h. The residue was purified by column chromatography (eluting with 5% ethyl acetate–hexane initially, grading to 40% ethyl acetate–hexane). The product was obtained as a colorless liquid (234 mg, 0.93 mmol, 93%, 82:18 d.r. (syn:anti)).

^{1}H and ^{13}C NMR data for **S5h** prepared in this way were in agreement with the literature.(*24*)

Major diastereomer:

^{1}H NMR (500 MHz, CDCl$_3$) δ 7.42 (s, 5H, H_{1-3}), 4.09 – 4.04 (m, 1H, H_6), 2.78 (qd, J = 7.8, 4.8 Hz, 1H, H_4), 2.05 (bs, 1H, OH), 1.83 – 1.75 (m, 1H, H_8), 1.55 – 1.42 (m, 1H, H_7), 1.31 (d, J = 6.3 Hz, 3H, H_5), 1.22 – 1.16 (m, 1H, H_7), 0.94 (m, 6H, H_9).

^{13}C NMR (126 MHz, CDCl$_3$) δ 202.35 (C), 134.6 (2 × CH), 129.5 (CH), 129.3 (2 × CH), 127.3 (C), 69.9 (CH), 53.3 (CH), 43.2 (CH$_2$), 24.6 (CH), 23.5 (CH$_3$), 21.9 (CH$_3$), 11.7 (CH$_3$).

Minor diastereomer (detectable non-overlapping resonances):

^{1}H NMR (500 MHz, CDCl$_3$) δ 3.83 – 3.78 (m, 1H, H_6), 2.86 – 2.81 (m, 1H, H_4), 1.90 – 1.87 (m, 1H, H_8), 1.33 (d, J = 7.2 Hz, 3H, H_5).

^{13}C NMR (126 MHz, CDCl$_3$) δ 202.32 (C), 134.5 (2 × CH), 127.4 (C), 72.1 (CH), 54.0 (CH), 44.0 (CH$_2$), 24.5 (CH), 23.7 (CH$_3$), 21.7 (CH$_3$), 15.2 (CH$_3$).

HRMS-CI (m/z): $[M + H]^+$ calcd for $C_{14}H_{21}O_2S$, 253.1262; found, 253.1245.

R_f = 0.49 (20% ethyl acetate-hexane; UV).

The *ee* was determined by chiral HPLC analysis of the benzoate derivative obtained by the reduction of the thioester with lithium borohydride and formation of the monobenzoate.

(2R,3R)-3-hydroxy-2,5-dimethylhexyl benzoate **S7h**

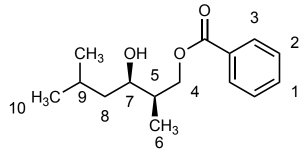

To S-phenyl (2*S*,3*R*)-3-hydroxy-2,5-dimethylhexanethioate **S5h** (79 mg, 0.31 mmol, 1 equiv) in diethylether (1.30 mL, 0.25 M) was added lithium borohydride (7 mg, 0.31 mmol, 1.00 equiv) under argon. The reaction was stirred for 1 h at 23 °C. The reaction was quenched with ammonium chloride and the organic layer was separated. The aqueous layer was extracted twice with ethyl acetate. The combined organic phase was washed with sodium bicarbonate and brine solution and the organic layer was dried over sodium sulfate. The dried solution was filtered and the filtrate was concentrated. The crude mixture was dissolved in dichloromethane (1.70 mL, 0.20 M). Benzoic acid (49 mg, 0.40 mmol, 1.20 equiv), 4-dimethylaminopyridine (4 mg, 0.03 mmol, 0.10 equiv) and 1-ethyl-3-(3-dimethylaminopropyl)carbodiimide (71 mg, 0.37 mmol, 1.10 equiv) were successively added. The reaction was stirred for 20 h at 23 °C. The reaction was quenched with water and the organic layer was separated. The aqueous layer was extracted twice with dichloromethane. The combined organic phase was washed with brine solution and the organic layer was dried over sodium sulfate. The dried solution was filtered and the filtrate was concentrated. The residue was purified by column chromatography (eluting with 5% ethyl acetate–hexane initially, grading to 60% ethyl acetate–hexane). The product **S7h** was obtained as a colorless oil (10 mg, 12%).

^{1}H and ^{13}C NMR data for **S7h** prepared in this way were in agreement with the literature.(*24*)

Major diastereomer:

^{1}H NMR (500 MHz, $CDCl_3$) δ 8.07 – 8.01 (m, 2H, H_3), 7.62 – 7.53 (m, 1H, H_1), 7.48 – 7.42 (m, 2H, H_2), 4.47 (ddd, *J* = 11.0, 7.0, 7.0 Hz, 1H, H_4), 4.19 (dd, *J* = 11.0, 6.0 Hz, 1H, H_4), 3.84 (ddd, *J* = 9.3, 4.0, 3.0 Hz, 1H, H_7), 2.05 – 1.93 (m, 1H, H_5), 1.80 – 1.68 (m, 2H, $H_{9,OH}$), 1.49 (ddd, *J* = 13.9, 9.3, 5.5 Hz, 1H, H_8), 1.24 (ddd, *J* = 13.8, 8.6, 4.0 Hz, 1H, H_8), 1.00 (d, *J* = 7.0 Hz, 3H, H_6), 0.94 (d, *J* = 6.7 Hz, 3H, H_{10}), 0.91 (d, *J* = 6.6 Hz, 3H, H_{10}).

^{13}C NMR (126 MHz, $CDCl_3$) δ 167.1 (C), 133.2 (CH), 130.3 (C), 129.7 (2 × CH), 128.6 (2 × CH), 69.5 (CH), 67.6 (CH_2), 43.7 (CH_2), 38.3 (CH), 24.9 (CH), 23.6 (CH_3), 22.2 (CH_3), 10.4 (CH_3).

Minor diastereomer (detectable non-overlapping resonances):

^{1}H NMR (500 MHz, $CDCl_3$) δ 4.34 (dd, *J* = 11.1, 5.1 Hz, 1H, H_4), 3.63 (ddd, *J* = 9.6, 6.4, 2.9 Hz, 1H, H_7), 1.85 (dddd, *J* = 9.8, 6.8, 6.8, 4.6 Hz, 1H, H_9), 1.44 – 1.41 (m, 1H, H_8), 1.34 – 1.30 (m, 1H, H_8), 1.06 (d, *J* = 6.9 Hz, 1H, H_6).

^{13}C NMR (126 MHz, $CDCl_3$) δ 130.4 (C), 71.4 (CH), 67.1 (CH_2), 39.6 (CH), 24.7 (CH), 24.0 (CH_3), 21.7 (CH_3), 14.0 (CH_3).

HPLC (AD-H, *n*-hexane/^{i}PrOH=93:7, 1.0 mL/min, 298 K, 254 nm): t_R(syn, minor) = 7.3 min, t_R(syn, major) = 8.1 min, t_R(anti, minor) = 9.0 min, t_R(anti, major) = 9.3 min, d.r. (syn:anti) = 82:18, e.r.(syn) = 99:1 (99% *ee*), e.r.(anti) = 96:4 (92% *ee*).

HRMS-CI (m/z): $[M + Na]^+$ calcd for $C_{17}H_{22}O_3Na$, 273.1467; found, 273.1445.

R_f = 0.56 (20% ethyl acetate-hexane; UV).

S-phenyl (2R,3R)-3-hydroxy-2,5-dimethylhexanethioate **S6h**

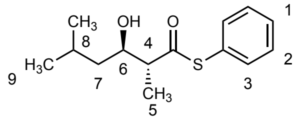

Following general method **B**, **S6h** was prepared from isovaleraldehyde (111 μL, 1.00 mmol, 1 equiv) and MAHT **2** (252 mg, 1.20 mmol, 1.20 equiv). The reaction was stirred for 24 h. The residue was purified by column chromatography (eluting with 5% ethyl acetate–hexane initially, grading to 40% ethyl acetate–hexane). The product was obtained as a white solid (235 mg, 93%, 14:86 d.r. (syn:anti)).

Major diastereomer:

^{1}H NMR (500 MHz, $CDCl_3$) δ 7.42 (s, 5H, $H_{1\text{-}3}$), 3.85 – 3.77 (m, 1H, H_6), 2.87 – 2.75 (m, 1H, H_8), 2.24 (d, *J* = 7.6 Hz, 1H, OH), 1.93 – 1.74 (m, 1H, H_4), 1.54 – 1.42 (m, 1H, H_7), 1.38 – 1.25 (m, 4H, $H_{5,7}$), 0.98 – 0.88 (m, 6H, H_9).

^{13}C NMR (126 MHz, $CDCl_3$) δ 202.5 (C), 134.6 (2 × CH), 129.68 (CH), 129.39 (2 × CH), 127.5 (C), 72.3 (CH), 54.1 (CH), 44.4 (CH_2), 24.7 (CH), 23.8 (CH_3), 21.8 (CH_3), 15.5 (CH_3).

Minor diastereomer (detectable non-overlapping resonances):

^{1}H NMR (500 MHz, $CDCl_3$) δ 4.10 – 4.02 (m, 1H, H_6), 2.29 (d, J = 3.9 Hz, 1H, OH), 1.23 – 1.15 (m, 1H, H_7).

^{13}C NMR (126 MHz, $CDCl_3$) δ 202.6 (C), 134.7 (2 × CH), 129.70 (CH), 129.40 (2 × CH), 127.4 (C), 70.0 (CH), 53.3 (CH), 43.2 (CH_2), 24.8 (CH), 23.6 (CH_3), 22.0 (CH_3), 11.6 (CH_3).

HRMS-CI (m/z): $[M + Na]^+$ calcd for $C_{14}H_{20}O_2SNa$, 275.1082; found, 275.1079.

R_f = 0.49 (20% ethyl acetate-hexane; UV).

The *ee* was determined by chiral HPLC analysis of the benzoate derivative obtained by the reduction of the thioester with lithium borohydride and formation of the monobenzoate.

(2S,3R)-3-hydroxy-2,5-dimethylhexyl benzoate **S8h**

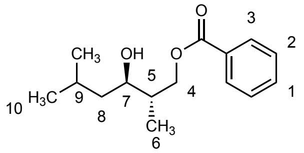

To S-phenyl (2*R*,3*R*)-3-hydroxy-2,5-dimethylhexanethioate **S6h** (89 mg, 0.35 mmol, 1 equiv) in diethylether (1.40 mL, 0.25 M) was added lithium borohydride (8 mg, 0.35 mmol, 1.00 equiv) under argon. The reaction was stirred for 1 h at 23 °C. The reaction was quenched with ammonium chloride and the organic layer was separated. The aqueous layer was extracted twice with ethyl acetate. The combined organic phase was washed with sodium bicarbonate and brine solution and the organic layer was dried over sodium sulfate. The dried solution was filtered and the filtrate was concentrated. The crude mixture was dissolved in dichloromethane (1.80 mL, 0.20 M). Benzoic acid (52 mg, 0.42 mmol, 1.20 equiv), 4-dimethylaminopyridine (4 mg, 0.04 mmol, 0.10 equiv) and 1-ethyl-3-(3-dimethylaminopropyl)carbodiimide (74 mg, 0.39 mmol, 1.10 equiv) were successively added. The reaction was stirred for 20 h at 23 °C. The reaction was quenched with water and the organic layer was separated. The aqueous layer was extracted twice with dichloromethane. The combined organic phase was washed with brine solution and the organic layer was dried over sodium sulfate. The dried solution was filtered and the filtrate was concentrated. The residue was purified by column chromatography (eluting with 5% ethyl acetate–hexane initially, grading to 60% ethyl acetate–hexane). The product **S8h** was obtained as a colorless oil (34 mg, 39%).

Major diastereomer:

^{1}H NMR (500 MHz, $CDCl_3$) δ 8.07 – 8.01 (m, 2H, H_3), 7.60 – 7.53 (m, 1H, H_1), 7.49 – 7.41 (m, 2H, H_2), 4.47 (dd, J = 11.1, 5.7 Hz, 1H, H_4), 4.34 (dd, J = 11.1, 5.1 Hz, 1H, H_4), 3.63 (ddd, J = 9.7, 6.5, 2.9 Hz, 1H, H_7), 2.56 (bs, 1H, OH), 2.02 – 1.92 (m, 1H, H_5), 1.91 – 1.79 (m, 1H, H_9), 1.54 – 1.39 (m, 1H, H_8), 1.32 (ddd, J = 14.0, 9.7, 2.9 Hz, 1H, H_8), 1.05 (d, J = 7.0 Hz, 3H, H_6), 0.95 (d, J = 6.7 Hz, 3H, H_{10}), 0.90 (d, J = 6.6 Hz, 3H, H_{10}).

^{13}C NMR (126 MHz, $CDCl_3$) δ 167.1 (C), 133.1 (CH), 130.33 (C), 129.70 (2 × CH), 128.5 (2 × CH), 71.4 (CH), 67.1 (CH_2), 43.6 (CH_2), 39.6 (CH), 24.7 (CH), 24.0 (CH_3), 21.7 (CH_3), 14.0 (CH_3).

Minor diastereomer (detectable non-overlapping resonances):

^{1}H NMR (500 MHz, $CDCl_3$) δ 8.12 – 8.06 (m, 2H, H_3), 4.19 (dd, J = 11.0, 6.1 Hz, 1H, H_4), 3.84 (ddd, J = 9.3, 4.0, 3.1 Hz, 1H, H_7), 1.81 – 1.71 (m, 1H, H_5), 1.29 – 1.20 (m, 1H, H_8), 1.00 (d, J = 7.0 Hz, 3H, H_6).

^{13}C NMR (126 MHz, $CDCl_3$) δ 169.9 (C), 133.6 (CH), 130.31 (C), 69.5 (CH), 67.6 (CH_2), 38.3 (CH), 24.9 (CH), 23.5 (CH_3), 22.2 (CH_3), 10.4 (CH_3).

HPLC (AD-H, *n*-hexane/^{i}PrOH=93:7, 1.0 mL/min, 298 K, 254 nm): t_R(syn, minor) = 7.3 min, t_R(syn, major) = 8.1 min, t_R(anti, minor) = 9.0 min, t_R(anti, major) = 9.3 min, d.r. (syn:anti) = 15:85, e.r.(syn) = 82:18 (64% *ee*), e.r.(anti) = 98:2 (96% *ee*).

HRMS-CI (m/z): $[M + Na]^+$ calcd for $C_{15}H_{22}O_3Na$, 275.1467; found, 273.1450.

R_f = 0.56 (20% ethyl acetate-hexane; UV).

Synthetic Procedures for the *syn*-aldol reaction using substituted MAHTs:

S-phenyl (S)-2-((S)-hydroxy(4-nitrophenyl)methyl)butanethioate **7a**

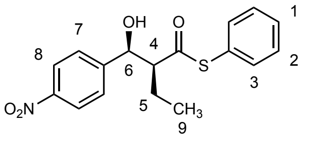

Following general method **A**, **7a** was prepared from 4-nitrobenzaldehyde (77 mg, 0.50 mmol, 1 equiv) and MAHT **S2a** (135 mg, 0.60 mmol, 1.20 equiv). The reaction was stirred for 48 h. The residue was purified by column chromatography (eluting with 5% ethyl acetate–hexane initially, grading to 50% ethyl acetate–hexane). The product was obtained as a white solid (140 mg, 84%, 94:6 d.r. (syn:anti)).

Major diastereomer:

^{1}H NMR (500 MHz, $CDCl_3$) δ 8.26 – 8.20 (m, 2H, H_7), 7.59 – 7.54 (m, 2H, H_8), 7.47 – 7.38 (m, 3H, $H_{1,2}$), 7.34 – 7.27 (m, 2H, H_3), 5.15 (d, J = 4.7 Hz, 1H, H_6), 2.95 – 2.88 (m, 1H, H_4), 1.96 – 1.79 (m, 1H, H_5), 1.70 – 1.58 (m, 1H, H_5), 0.98 (t, J = 7.5 Hz, 3H, H_9).

^{13}C NMR (126 MHz, $CDCl_3$) δ 201.4 (C), 148.6 (C), 147.6 (C), 134.4 (2 × CH), 130.0 (CH), 129.5 (2 × CH), 127.3 (2 × CH), 126.9 (C), 123.7 (2 × CH), 73.3 (CH), 62.0 (CH), 20.5 (CH_2), 12.1 (CH_3).

Minor diastereomer (detectable non-overlapping resonances):

^{1}H NMR (500 MHz, $CDCl_3$) δ 7.54 – 7.51 (m, 2H), 5.00 (d, J = 6.1 Hz, 1H), 3.01 – 2.96 (m, 1H), 1.05 (t, J = 7.4 Hz, 3H).

^{13}C NMR (126 MHz, $CDCl_3$) δ 201.5 (C), 149.3 (C), 129.9 (CH), 129.4 (2 × CH), 127.1 (2 × CH), 123.8 (2 × CH), 74.3 (CH), 61.6 (CH), 23.6 (CH_2), 11.7 (CH_3).

HPLC (AD-H, *n*-hexane/^{i}PrOH=90:10, 1.0 mL/min, 298 K, 210 nm): t_R(syn, minor) = 10.8 min, t_R(syn, major) = 11.7 min, t_R(anti, minor) = 17.4 min, t_R(anti, major) = 36.3 min, d.r. (syn:anti) = 94:6, e.r.(syn) = 98:2 (97% ee), e.r.(anti) = 90:10 (80% ee).

HRMS-CI (m/z): [M + Na]$^+$ calcd for $C_{17}H_{17}NO_4SNa$, 354.0776; found, 354.0772.

R_f = 0.40 (30% ethyl acetate-hexane; UV).

S-phenyl (S)-2-((S)-hydroxy(4-nitrophenyl)methyl)heptanethioate **7b**

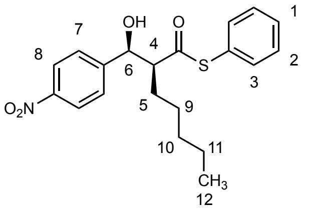

Following general method **A**, **7b** was prepared from 4-nitrobenzaldehyde (77 mg, 0.50 mmol, 1 equiv), MAHT **S2b** (266 mg, 1.00 mmol, 2.00 equiv), (*S,S*)-salen **3a** (38 mg, 0.10 mmol, 20 mol%) and titanium (IV) isopropoxide (0.50 M in toluene; 0.11 mmol, 22 mol%). The reaction was stirred for 48 h. The residue was purified by column chromatography (eluting with 5% ethyl acetate–hexane initially, grading to 50% ethyl acetate–hexane). The product was obtained as a colorless oil (156 mg, 83%, 92:8 d.r. (syn:anti)).

Major diastereomer:

^{1}H NMR (500 MHz, $CDCl_3$) δ 8.84 – 8.79 (m, 2H, H_7), 8.14 (d, 2H, H_8), 8.04 – 7.97 (m, 3H, $H_{1,2}$), 7.91 – 7.87 (m, 2H, H_3), 5.72 (d, J = 4.7 Hz, 1H, H_6), 3.63 (d, J = 2.3 Hz, 1H, OH), 3.59 – 3.52 (m, 1H, H_4), 2.51 – 2.32 (m, 1H, H_5), 2.19 – 2.07 (m, 1H, H_5), 2.07 – 1.94 (m, 1H, H_9), 1.93 – 1.72 (m, 5H, $H_{9,10,11}$), 1.44 (t, 3H, H_{12}).

^{13}C NMR (126 MHz, $CDCl_3$) δ 201.5 (C), 148.6 (C), 147.6 (C), 134.4 (2 × CH), 130.0 (CH), 129.5 (2 × CH), 127.3 (2 × CH), 126.9 (C), 123.7 (2 × CH), 73.4 (CH), 60.6 (CH), 31.8 (CH_2), 27.2 (CH_2), 27.1 (CH_2), 22.5 (CH_2), 14.1 (CH_3).

Minor diastereomer (detectable non-overlapping resonances):

^{1}H NMR (500 MHz, $CDCl_3$) δ 8.11 (d, J = 8.6 Hz, 2H, H_8), 5.57 – 5.55 (dd, J = 6.2, 6.2 Hz, 1H, H_6), 3.83 (d, J = 6.2 Hz, 1H, OH), 1.49 – 1.47 (m, 3H, H_{12}).

^{13}C NMR (126 MHz, $CDCl_3$) δ 201.7 (C), 149.5 (C), 147.7 (C), 130.6 (CH), 127.2 (2 × CH), 123.8 (2 × CH), 74.7 (CH), 60.2 (CH), 31.7 (CH_2), 30.4 (CH_2), 26.9 (CH_2).

HPLC (AD-H, *n*-hexane/^{i}PrOH=90:10, 1.0 mL/min, 298 K, 210 nm): t_R(syn, minor) = 10.9 min, t_R(syn, major) = 12.2 min, t_R(anti, minor) = 13.3 min, t_R(anti, major) = 31.5 min, d.r. (syn:anti) = 92:8, e.r.(syn) = 97:3 (94% ee), e.r.(anti) = 76:24 (52% ee).
HRMS-CI (m/z): $[M + H]^+$ calcd for $C_{20}H_{24}NO_4S$, 374.1426; found, 374.1412.
R_f = 0.50 (20% ethyl acetate-hexane; UV).

S-phenyl (S)-2-((S)-hydroxy(4-nitrophenyl)methyl)-5-phenylpentanethioate **7c**

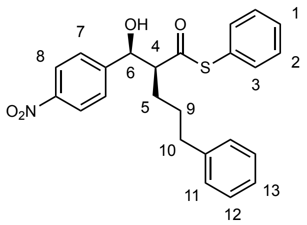

Following general method **A**, **7c** was prepared from 4-nitrobenzaldehyde (77 mg, 0.50 mmol, 1 equiv), MAHT **S2c** (314 mg, 1.00 mmol, 2.00 equiv), (*S,S*)-salen **3a** (38 mg, 0.10 mmol, 20 mol%) and titanium (IV) isopropoxide (0.50 M in toluene; 0.11 mmol, 22 mol%). The reaction was stirred for 48 h. The residue was purified by column chromatography (eluting with 5% ethyl acetate–hexane initially, grading to 50% ethyl acetate–hexane). The product was obtained as a white solid (148 mg, 70%, 92:8 d.r. (syn:anti)).

Major diastereomer:

^{1}H NMR (500 MHz, $CDCl_3$) δ 8.25 – 8.19 (m, 2H, H_7), 7.55 – 7.51 (m, 2H, H_8), 7.45 – 7.38 (m, 3H, $H_{12,13}$), 7.33 – 7.22 (m, 5H, $H_{1\text{-}3}$), 7.12 – 7.07 (m, 2H, H_{11}), 5.15 (d, *J* = 4.6 Hz, 1H, H_6), 3.02 – 2.96 (m, 1H, H_4), 2.64 – 2.52 (m, 2H, H_{10}), 1.96 – 1.85 (m, 1H, H_5), 1.85 – 1.72 (m, 1H, H_5), 1.68 – 1.52 (m, 2H, H_9).

^{13}C NMR (126 MHz, $CDCl_3$) δ 201.3 (C), 148.4 (C), 147.6 (C), 141.6 (C), 134.3 (2 × CH), 130.1 (CH), 129.54 (2 × CH), 128.5 (2 × CH), 128.5 (2 × CH), 127.29 (2 × CH), 126.77 (C), 126.1 (CH), 123.7 (2 × CH), 73.4 (CH), 60.3 (CH), 35.8 (CH_2), 29.2 (CH_2), 26.6 (CH_2).

Minor diastereomer (detectable non-overlapping resonances):

^{1}H NMR (500 MHz, $CDCl_3$) δ 7.58 – 7.55 (m, 2H, H_8), 4.96 (d, *J* = 6.1 Hz, 1H, H_6), 3.08 – 3.03 (m, 1H, H_4), 2.94 – 2.90 (m, 2H, H_{10}).

^{13}C NMR (126 MHz, $CDCl_3$) δ 149.3 (C), 147.7 (C), 141.5 (C), 130.0 (CH), 129.49 (2 × CH), 128.6 (2 × CH), 127.34 (CH), 127.2 (2 × CH), 126.79 (C), 126.2 (CH), 123.8 (2 × CH), 74.6 (CH), 62.0 (CH), 35.7 (CH_2), 29.9 (CH_2), 28.9 (CH_2).

HPLC (AD-H, *n*-hexane/^{i}PrOH=88:12, 1.0 mL/min, 298 K, 210 nm): t_R(syn, minor) = 13.0 min, t_R(syn, major) = 16.0 min, t_R(anti, minor) = 19.9 min, t_R(anti, major) = 38.7 min, d.r. (syn:anti) = 92:8, e.r.(syn) = 98:2 (95% ee), e.r.(anti) = 76:24 (52% ee).
HRMS-CI (m/z): $[M + Na]^+$ calcd for $C_{24}H_{23}NO_4SNa$, 444.1245; found, 444.1223.
R_f = 0.43 (20% ethyl acetate-hexane; UV).
The absolute stereochemistry for **7c** was confirmed by X-ray crystallography (see **fig. S4**). The crystal was grown by a slow evaporation of dichloromethane.

S-phenyl (S)-4-(benzyloxy)-2-((S)-hydroxy(4-nitrophenyl)methyl)butanethioate **7d**

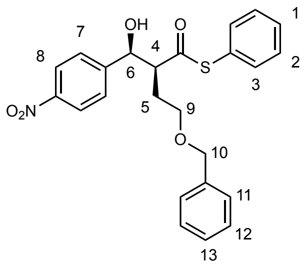

Following general method **A**, **7d** was prepared from 4-nitrobenzaldehyde (77 mg, 0.50 mmol, 1 equiv), MAHT **S2d** (330 mg, 1.00 mmol, 2.00 equiv), (*S,S*)-salen **3a** (38 mg, 0.10 mmol, 20 mol%) and titanium (IV) isopropoxide (0.50 M in toluene; 0.11 mmol, 22 mol%). The reaction was stirred for 48 h. The residue was purified by column chromatography (eluting with 5% ethyl acetate–hexane initially, grading to 70% ethyl acetate–hexane). The product was obtained as a colorless oil (111 mg, 77%, 85:15 d.r. (syn:anti)).

Major diastereomer:

^{1}H NMR (500 MHz, $CDCl_3$) δ 8.26 – 8.17 (m, 2H, H_7), 7.58 – 7.52 (m, 2H, H_8), 7.45 – 7.28 (m, 8H, $H_{1,2,11\text{-}13}$), 7.22 – 7.14 (m, 2H, H_3), 5.15 (d, *J* = 5.8 Hz, 1H, H_6), 4.50 (s, 2H, H_{10}), 3.76 (bs, 1H, OH), 3.65 – 3.46 (m, 2H, H_9), 3.18 (ddd, *J* = 7.9, 5.8, 4.3 Hz, 1H, H_4), 2.27 – 2.12 (m, 1H, H_5), 2.03 – 1.84 (m, 1H, H_5).

^{13}C NMR (126 MHz, $CDCl_3$) δ 200.36 (C), 148.65 (C), 147.56 (C), 137.62 (C), 134.36 (2 × CH), 129.9 (CH), 129.43 (2 × CH), 128.61 (2 × CH), 128.03 (CH), 128.02 (2 × CH), 127.51 (2 × CH), 126.8 (C), 123.66 (2 × CH), 73.5 (CH_2), 73.3 (CH), 67.7 (CH_2), 58.63 (CH), 28.30 (CH_2).

Minor diastereomer (detectable non-overlapping resonances):

^1H NMR (500 MHz, $CDCl_3$) δ 6.79 (d, J = 8.6 Hz, 2H, H_6), 4.29 (d, J = 5.5 Hz, 1H, H_5), 3.84 (s, 2H, H_{10}), 2.82 – 2.74 (m, 2H, H_9), 2.68 – 2.63 (m, 1H, H_4), 1.45 – 1.40 (m, 1H, H_8), 1.25 – 1.17 (m, 1H, H_8).

^{13}C NMR (126 MHz, $CDCl_3$) δ 201.27 (C), 149.28 (C), 147.61 (C), 137.92 (C), 134.30 (2 × CH), 128.10 (2 × CH), 127.19 (2 × CH), 126.83(2 × CH), 126.80 (2 × CH), 123.75 (2 × CH), 74.43 (CH_2), 67.14 (CH_2), 56.88 (CH), 30.49 (CH_2).

HPLC (AD-H, *n*-hexane/^{i}PrOH=88:12, 1.0 mL/min, 298 K, 210 nm): t_R(syn, minor) = 16.2 min, t_R(syn, major) = 18.0 min, t_R(anti, minor) = 25.0 min, t_R(anti, major) = 37.3 min, d.r. (syn:anti) = 85:15, e.r.(syn) = 94:6 (89% ee), e.r.(anti) = 66:34 (32% ee).

HRMS-CI (m/z): $[M + Na]^+$ calcd for $C_{24}H_{23}NO_5SNa$, 460.1195; found, 460.1178.

R_f = 0.25 (30% ethyl acetate-hexane; UV).

S-phenyl (S)-2-((S)-hydroxy(4-nitrophenyl)methyl)pent-4-enethioate **7e**

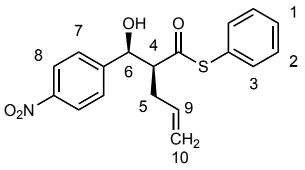

Following general method **A**, **7e** was prepared from 4-nitrobenzaldehyde (77 mg, 0.50 mmol, 1 equiv) and MAHT **S2e** (142 mg, 0.60 mmol, 1.20 equiv). The reaction was stirred for 48 h. The residue was purified by column chromatography (eluting with 5% ethyl acetate–hexane initially, grading to 40% ethyl acetate–hexane). The product was obtained as a white solid (158 mg, 91%, 88:12 d.r. (syn:anti)).

Major diastereomer:

^1H NMR (500 MHz, $CDCl_3$) δ 8.26 – 8.21 (m, 2H, H_7), 7.58 – 7.54 (m, 2H, H_8), 7.46 – 7.37 (m, 3H, $H_{1,2}$), 7.31 – 7.23 (m, 2H, H_3), 5.86 – 5.70 (m, 1H, H_9), 5.18 (d, J = 4.8 Hz, 1H, H_6), 5.12 – 5.06 (m, 2H, H_{10}), 3.10 – 3.04 (m, 1H, H_4), 2.65 – 2.57 (m, 1H, H_5), 2.41 – 2.34 (m, 1H, H_5).

^{13}C NMR (126 MHz, $CDCl_3$) δ 200.5 (C), 148.3 (C), 147.66 (C), 134.4 (2 × CH), 134.3 (CH), 130.0 (CH), 129.5 (2 × CH), 127.4 (C), 126.8 (2 × CH), 123.8 (2 × CH), 118.2 (CH_2), 73.1 (CH), 60.1 (CH), 31.8 (CH_2).

Minor diastereomer (detectable non-overlapping resonances):

^1H NMR (500 MHz, $CDCl_3$) δ 7.54 – 7.51 (m, 2H, H_8), 5.16 – 5.14 (m, 2H, H_{10}), 5.01 (d, J = 5.7 Hz, 1H, H_6), 3.16 – 3.10 (m, 1H, H_4), 2.56 – 2.48 (m, 1H, H_5), 2.45 – 2.41 (m, 1H, H_5).

^{13}C NMR (126 MHz, $CDCl_3$) δ 201.0 (C), 149.2 (C), 147.72 (C), 133.5 (2 × CH), 127.21 (2 × CH), 126.7 (C), 123.9 (2 × CH), 118.9 (CH_2), 73.9 (CH), 59.5 (CH), 34.6 (CH_2).

HPLC (AD-H, *n*-hexane/^{i}PrOH=90:10, 1.0 mL/min, 298 K, 210 nm): t_R(syn, minor) = 11.1 min, t_R(syn, major) = 11.9 min, t_R(anti, minor) = 16.7 min, t_R(anti, major) = 39.8 min, d.r. (syn:anti) = 88:12, e.r.(syn) = 97:3 (93% ee), e.r.(anti) = 82:18 (63% ee).

HRMS-CI (m/z): $[M + Na]^+$ calcd for $C_{18}H_{17}NO_4SNa$, 366.0776; found, 366.0757.

R_f = 0.45 (30% ethyl acetate-hexane; UV).

S-phenyl (S)-2-((S)-hydroxy(4-nitrophenyl)methyl)pent-4-ynethioate **7f**

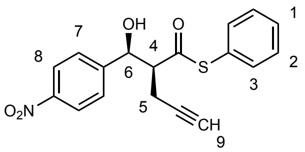

Following general method **A**, **7f** was prepared from 4-nitrobenzaldehyde (77 mg, 0.50 mmol, 1 equiv), MAHT **S2f** (234 mg, 1.00 mmol, 2.00 equiv), (*S,S*)-salen **3a** (38 mg, 0.10 mmol, 20 mol%) and titanium (IV) isopropoxide (0.50 M in toluene; 0.11 mmol, 22 mol%). The reaction was stirred for 48 h. The residue was purified by column chromatography (eluting with 5% ethyl acetate–hexane initially, grading to 50% ethyl acetate–hexane). The product was obtained as a colorless oil (163 mg, 95%, 77:23 d.r. (syn:anti)).

^{1}H NMR (500 MHz, $CDCl_3$) δ 8.26 – 8.20 (m, 2H, H_7), 7.60 – 7.51 (m, 2H, H_8), 7.47 – 7.37 (m, 3H, $H_{1,2}$), 7.32 – 7.24 (m, 2H, H_3), 5.20 (d, J = 5.5 Hz, 1H, H_6), , 3.26 – 3.17 (m, 1H, H_4), 3.09 (bs, 1H, OH), 2.76 (ddd, J = 17.1, 8.9, 2.7 Hz, 1H, H_5), 2.60 – 2.48 (m, 1H, H_5), 2.08 (t, J = 2.7 Hz, 1H, H_9).

^{13}C NMR (126 MHz, $CDCl_3$) δ 199.0 (C), 147.80 (C), 147.76 (C), 134.4 (2 × CH), 130.1 (CH), 129.5 (2 × CH), 127.4 (2 × CH), 126.5 (C), 123.8 (2 × CH), 80.3 (C), 72.7 (CH), 71.6 (CH), 58.8 (CH), 17.4 (CH_2).

Minor diastereomer (detectable non-overlapping resonances):

^{1}H NMR (500 MHz, $CDCl_3$) δ 3.33 (d, J = 6.9 Hz, 1H, OH), 2.67 (ddd, J = 17.1, 6.9, 2.7 Hz, 1H, H_5), 2.18 (t, J = 2.6 Hz, 1H, H_9).

^{13}C NMR (126 MHz, $CDCl_3$) δ 199.6 (C), 148.5 (C), 130.1 (CH), 127.2 (2 × CH), 126.6 (2 × CH), 123.9 (2 × CH), 79.4 (C), 73.2 (CH), 72.2 (CH), 58.0 (CH), 20 (CH_2).

HPLC (AD-H, *n*-hexane/EtOH=85:15, 1.0 mL/min, 298 K, 210 nm): t_R(syn, major) = 16.3 min, t_R(syn, minor) = 18.5 min, t_R(anti, major) = 26.5 min, t_R(anti, minor) = 34.9 min, d.r. (syn:anti) = 77:23, e.r.(syn) = 97:3 (93% ee), e.r.(anti) = 90:10 (80% ee).

HRMS-CI (m/z): $[M + Na]^+$ calcd for $C_{18}H_{15}NO_4SNa$, 364.0619; found, 364.0601.

R_f = 0.35 (30% ethyl acetate-hexane; UV).

S-phenyl (2*S*,3*S*)-2-(4-nitrophenyl)-6-oxotetrahydro-2*H*-pyran-3-carbothioate **7g**

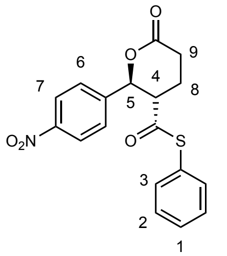

Following general method **A**, **7g** was prepared from 4-nitrobenzaldehyde (77 mg, 0.50 mmol, 1 equiv) and MAHT **S2g** (282 mg, 1.00 mmol, 2.00 equiv), (*S,S*)-Salen **3a** (38.2 mg, 0.10 mmol, 20 mol%) and titanium (IV) isopropoxide (0.50 M in toluene, 0.11 mmol, 22 mol%). The reaction was stirred for 48 h. The crude reaction was filtered through celite and rinsed with dichloromethane. The filtrate was concentrated. The crude aldol product was redissolved in dichloromethane (10.0 mL, 0.05 M) and *p*-toluenesulfonic acid (19 mg, 0.10 mmol, 0.20 equiv), and the reaction was heated at 50 °C for 60 h. The reaction was quenched by addition of cold water and the aqueous layer was extracted with dichloromethane. The combined organic extracts were washed with a brine solution and dried over sodium sulfate. The dried solution was filtered and the filtrate was concentrated. The crude compound was purified by column chromatography (eluting with 10% ethyl acetate–hexane initially, grading to 80% ethyl acetate–hexane). The product was obtained as a white solid (117 mg, 65 %, >99:1 d.r. (syn:anti)).

^{1}H NMR (500 MHz, $CDCl_3$) δ 8.30 – 8.21 (m, 2H, H_6), 7.58 – 7.49 (m, 2H, H_7), 7.46 – 7.34 (m, 3H, $H_{1,2}$), 7.21 – 7.13 (m, 2H, H_3), 5.62 (d, J = 10.0 Hz, 1H, H_5), 3.19 (ddd, J = 10.0, 9.1, 6.4 Hz, 1H, H_4), 2.89 (dt, J = 17.6, 6.9 Hz, 1H, H_9), 2.73 (dt, J = 17.6, 6.9 Hz, 1H, H_9), 2.42 – 2.28 (m, 2H, H_8).

^{13}C NMR (126 MHz, $CDCl_3$) δ 196.9 (C), 169.3 (C), 148.4 (C), 144.0 (C), 134.4 (2 × CH), 130.3 (CH), 129.6 (2 × CH), 128.0 (2 × CH), 125.8 (C), 124.1 (2 × CH), 80.7 (CH), 54.3 (CH), 28.5 (CH_2), 24.0 (CH_2).

HPLC (AD-H, *n*-hexane/EtOH=88:12, 1.0 mL/min, 298 K, 210 nm): t_R(syn, minor) = 12.4 min, t_R(syn, major) = 14.2 min, t_R(anti, minor) = 20.3 min, d.r. (syn:anti) = >99:1, e.r.(syn) = 99:1 (98% ee).

HRMS-CI (m/z): $[M + H]^+$ calcd for $C_{18}H_{15}NO_5S$, 358.0749; found, 358.0751.

R_f = 0.50 (40% ethyl acetate-hexane; UV).

Synthetic procedures for the large-scale synthesis and transformation of aldol product 5w:

S-phenyl (2S,3S)-3-hydroxy-2-methyl-3-(2-methylthiazol-4-yl)propanethioate **5w**

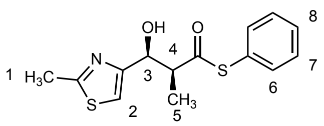

To a 1 L flask, molecular sieves (4 Å, 10 g) were added and flame dried under high vacuum. After cooling down to room temperature, the flask was back filled with air and toluene (500 mL, 0.1 M) was added followed by (*S,S*)-salen **3a** (0.478 g, 1.25 mmol, 0.025 equiv), 2-propanol (3.8 mL, 50.0 mmol, 1.0 equiv) and a solution of titanium (IV) isopropoxide (0.5 M in toluene; 0.11 mmol, 11 mol%). The catalyst mixture was stirred for 1 h at 23 °C. Malonic acid half thioester **2** (11.0 g, 52.5 mmol, 1.05 equiv) was added to the catalyst mixture in one portion. The resulting red solution was stirred for 15 min at 23 °C followed by addition of the 2-methylthiazole-4-carboxaldehyde (6.36 g, 50 mmol, 1.0 equiv). The reaction mixture was stirred for 24 h. The solution gradually turned a clear, yellow color over the course of the reaction. The product mixture was filtered through a plug of celite and the plug was washed with ethyl acetate. The filtrate was concentrated and the residue was purified by column chromatography (eluting with hexane initially, grading to 30% diethyl ether-hexane) to give *S*-phenyl (2*S*,3*S*)-3-hydroxy-2-methyl-3-(2-methylthiazol-4-yl)propanethioate as a white solid (11.8 g, 40.34 mmol, 81% yield).

^{1}H NMR (500 MHz, $CDCl_3$) δ 7.45 – 7.33 (m, 5H, $H_{6\text{-}8}$), 7.06 (d, *J* = 1.1 Hz, 1H, H_2), 5.23 – 5.19 (m, 1H, H_3), 3.39 (qd, *J* = 7.1, 4.2 Hz, 1H, H_4), 3.29 (d, *J* = 4.2 Hz, 1H, OH), 2.70 (s, 3H, H_1), 1.25 (d, *J* = 7.1 Hz, 3H, H_5).

^{13}C NMR (126 MHz, $CDCl_3$) δ 202.2 (C), 166.4 (C), 155.9 (C), 134.6 (2 × CH), 129.6 (CH), 129.3 (2 × CH), 127.3 (C), 114.5 (CH), 71.4 (CH), 53.0 (CH), 19.2 (CH_3), 12.0 (CH_3).

HPLC (AD-H, *n*-hexane/^{i}PrOH=90:10, 1.0 mL/min, 298 K, 254 nm): t_R(syn, minor) = 10.5 min, t_R(syn, major) = 11.9 min, t_R(anti, major) = 15.8 min, t_R(anti, minor) = 17.9 min, d.r. (syn:anti) = 94:6, e.r.(syn) = 96:4 (92% *ee*), e.r.(anti) = 95:5 (90% *ee*).

HRMS-CI (m/z): $[M + H]^+$ calcd for $C_{14}H_{15}NO_2S_2$, 294.0622; found, 294.0612.

R_f = 0.50 (syn), 0.40 (anti) (20% ethyl acetate-hexane; UV).

The absolute stereochemistry for **5w** was confirmed by X-ray crystallography (see **Fig. 3b**). The crystal was grown by a slow evaporation of dichloromethane.

(2S,3S)-3-hydroxy-2-methyl-3-(2-methylthiazol-4-yl)propanal **9**

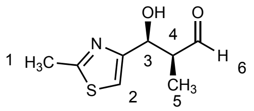

To a solution of the thioester **5w** (293 mg, 1.00 mmol, 1 equiv) in acetone (35.2 mL, 0.03 M) was added palladium(II) acetate (72 mg, 0.32 mmol, 0.32 equiv), magnesium sulfate (1.00 g, 8.31 mmol, 8.30 equiv) and triethylsilane (2.10 mL, 13.5 mmol, 13.5 equiv) sequentially. The reaction was stirred for 2 h at 23 °C. The reaction was quenched by addition of methanol. The mixture was filtered through celite. The filtrate was concentrated and the obtained residue was purified by column chromatography (eluting with 2% ethyl acetate-hexane initially, grading to 60% ethyl acetate-hexane) to give the aldehyde **9** as a brown liquid (142 mg, 77%).

^{1}H NMR (500 MHz, $CDCl_3$) δ 9.79 (s, 1H, H_6), 7.03 (s, 1H, H_2), 5.40 – 5.19 (m, 1H, H_3), 3.34 (s, 1H, OH), 2.95 – 3.01 (m, 1H, H_4), 2.65 (s, 3H, H_1), 1.03 (d, *J* = 7.3 Hz, 3H, H_5).

^{13}C NMR (126 MHz, $CDCl_3$) δ 204.4 (CHO), 166.6 (C), 156.6 (C), 114.2 (CH), 69.7 (CH), 51.6 (CH), 19.2 (CH_3), 7.6 (CH_3).

HRMS-CI (m/z): $[M + Na]^+$ calcd for $C_8H_{11}NO_2SNa$, 208.0408; found, 208.0407.

R_f = 0.34 (40% ethyl acetate-hexane; UV).

(2S,3S)-3-hydroxy-2-methyl-3-(2-methylthiazol-4-yl)-1-morpholinopropan-1-one **10**

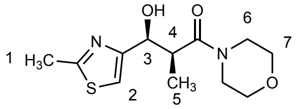

To a solution of thioester **5w** (100 mg, 0.34 mmol, 1 equiv) in toluene (4.30 mL, 0.08 M) was added morpholine (88 µL, 1.02 mmol, 3.00 equiv) and silver trifluoroacetate (83 mg, 0.37 mmol, 1.10 equiv). The reaction mixture was stirred at 60 °C for 20 min. The product mixture was allowed to cool to room temperature. The product mixture was diluted with dichloromethane and ammonium hydroxide solution (10%) and stirred vigorously for 10 min. The resulting heterogenous solution was filtered. The filtrate was extracted with dichloromethane. The combined organic extracts were dried over sodium sulfate, filtered and concentrated. The crude compound was purified by column chromatography (eluting with 5% ethyl acetate-hexane initially, grading to 60% ethyl acetate-hexane) to give amide **10** as a colorless liquid (87 mg, 94%).

^{1}H NMR (500 MHz, $CDCl_3$) δ 7.11 (d, *J* = 1.2 Hz, 1H, H_2), 5.13 (s, 1H, H_3), 4.92 (bs, 1H, OH), 3.66 – 3.41 (m, 8H, $H_{6,7}$), 3.22 (qd, *J* = 7.2, 2.8 Hz, 1H, H_4), 2.63 (s, 3H, H_1), 0.96 (d, *J* = 7.2 Hz, 3H, H_5).

^{13}C NMR (126 MHz, $CDCl_3$) δ 175.9 (C), 165.9 (C), 156.4 (C), 114.4 (CH), 71.6 (CH), 66.9 (CH_2), 66.7 (CH_2), 46.2 (CH_2), 41.9 (CH_2), 38.9 (CH), 19.1 (CH_3), 10.4 (CH_3).

HRMS-CI (m/z): $[M + Na]^+$ calcd for $C_{12}H_{18}N_2O_3SNa$, 293.0936; found, 293.0930.

R_f = 0.17 (80% ethyl acetate-hexane; UV).

methyl ((2S,3S)-3-hydroxy-2-methyl-3-(2-methylthiazol-4-yl)propanoyl)-L-cysteinate **11**

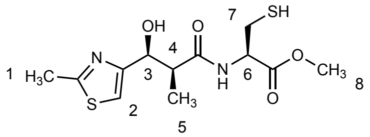

To a solution of thioester **5w** (293 mg, 1.00 mmol, 1 equiv) in dimethylformamide (5.00 mL, 0.20 M) was added *L*-cysteine methyl ester hydrochloride (171 mg, 1.00 mmol, 1.00 equiv), DL-dithiothreitol (308 mg, 2.00 mmol, 2.00 equiv), and *N*,*N*-diisopropylethylamine (170 µL, 1.00 mmol, 1.00 eq) sequentially. The mixture was stirred for 5 h at 23 °C. The product mixture was concentrated and the obtained residue was purified by flash chromatography, (eluting with 20% ethyl acetate-hexane initially, grading to 80% ethyl acetate-hexane) to give ester **11** as a colorless liquid (245 mg, 77%).

^{1}H NMR (500 MHz, $CDCl_3$) δ 7.25 (d, *J* = 7.4 Hz, 1H, NH), 7.04 (s, 1H, H_2), 5.06 (d, *J* = 3.7 Hz, 1H, H_3), 4.72 – 4.75 (m, 1H, H_6), 4.55 (bs, 1H, OH), 3.69 (s, 3H, H_8), 3.07 – 2.86 (m, 3H, $H_{4,7}$), 2.59 (s, 3H, H_1), 1.40 (t, *J* = 8.9 Hz, 1H, SH), 1.04 (d, *J* = 7.0 Hz, 3H, H_5).

^{13}C NMR (126 MHz, $CDCl_3$) δ 175.9 (C), 170.4 (C), 166.4 (C), 156.3 (C), 114.5 (CH), 71.3 (CH), 53.6 (CH), 52.7 (CH_3), 44.9 (CH), 26.6 (CH_2), 19.0 (CH_3), 11.3 (CH_3).

HRMS-CI (m/z): $[M + H]^+$ calcd for $C_{12}H_{19}N_2O_4S_2$, 319.0786; found, 319.0775.

R_f = 0.20 (80% ethyl acetate-hexane; UV).

S-(2-acetamidoethyl) (2S,3S)-3-hydroxy-2-methyl-3-(2-methylthiazol-4-yl)propanethioate **12**

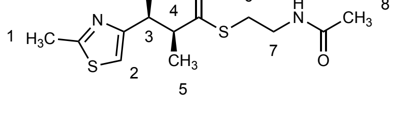

To as solution of thioester **5w** (100 mg, 0.34 mmol, 1 eq) in dimethylformamide (6.80 mL, 0.05 M) was added *N*,*N*-diisopropylethylamine (270 µL, 1.56 mmol, 4.60 equiv) and *N*-acetylcysteamine (HSNAC) (401 mg, 3.20 mmol, 9.40 equiv) sequentially. The reaction was stirred for 24 h at 23 °C. The product mixture was concentrated and the obtained residue was purified by flash chromatography on neutralized silica, (eluting with 10% ethyl acetate-hexane initially, grading to 80% ethyl acetate-hexane) to give thioester **12** as a colorless liquid (87 mg, 84%).

^{1}H NMR (500 MHz, $CDCl_3$) δ 7.04 (s, 1H, H_2), 6.19 (s, 1H, NH), 5.20 – 5.15 (m, 1H, H_3), 3.71 (d, *J* = 4.9 Hz, 1H, OH), 3.47 – 3.32 (m, 2H, H_7), 3.25 (qd, *J* = 7.1, 4.0 Hz, 1H, H_4), 3.07 – 2.89 (m, 2H, H_6), 2.65 (s, 3H, H_1), 1.93 (s, 3H, H_8), 1.11 (d, *J* = 7.1 Hz, 3H, H_5).

^{13}C NMR (126 MHz, $CDCl_3$) δ 202.9 (C), 171.0 (C), 166.3 (C), 156.5 (C), 114.4 (CH), 71.4 (CH), 53.4 (CH), 39.1 (CH_2), 28.5 (CH_2), 23.0 (CH_3), 19.0 (CH_3), 11.0 (CH_3).

HRMS-CI (m/z): $[M + Na]^+$ calcd for $C_{12}H_{18}N_2O_3S_2Na$, 325.0657; found, 325.0649.

R_f = 0.13 (80% ethyl acetate-hexane; UV).

Synthetic procedures for the synthesis and transformation of aldol product 6x:

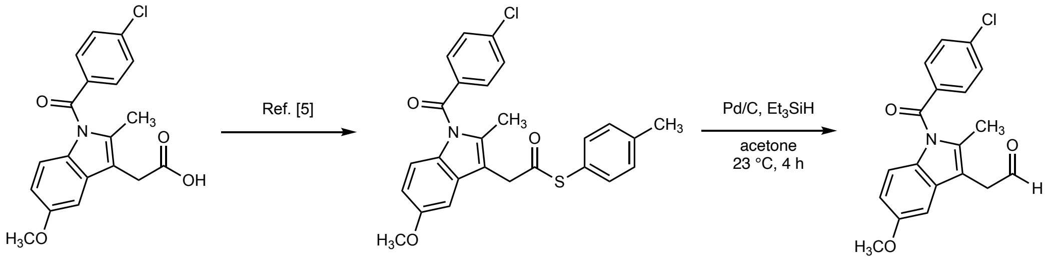

Indomethacin (**13**) **S9** **S10**

2-(1-(4-chlorobenzoyl)-5-methoxy-2-methyl-1H-indol-3-yl)acetaldehyde **S10**

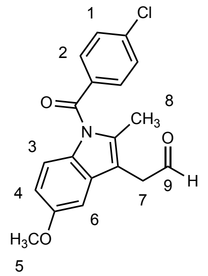

To a solution of thioester **S9** (396 mg, 0.85 mmol, 1 equiv) in acetone (17.1 mL, 0.05 M) was added Pd/C (wt% = 10%, 45 mg, 0.04 mmol, 5 mol%), followed by the dropwise addition of triethylsilane (679 µL, 4.27 mmol, 5.00 equiv). The reaction mixture was stirred for 3 h at 23 °C before the dropwise addition of an additional portion of triethylsilane (679 µL, 4.27 mmol, 5.00 equiv). The reaction mixture was stirred for 1 h at 23 °C. The product mixture was filtered through a short plug of silica and the filtrate was concentrated. The obtained residue was purified by column chromatography (eluting with 5% ethyl acetate–hexane initially, grading to 50% ethyl acetate–hexane) to yield aldehyde **S10** as a yellow solid (238 mg, 82%).

^{1}H and ^{13}C NMR data for **S10** prepared in this way were in agreement with the literature.(*55*)

^{1}H NMR (500 MHz, $CDCl_3$) δ 9.71 (t, *J* = 2.4 Hz, 1H, H_9), 7.72 – 7.64 (m, 2H, H_2), 7.52 – 7.46 (m, 2H, H_1), 6.89 – 6.82 (m, 2H, $H_{3,6}$), 6.69 (dd, *J* = 9.0, 2.6 Hz, 1H, H_4), 3.83 (s, 3H, H_5), 3.72 (d, *J* = 2.4 Hz, 2H, H_7), 2.39 (s, 3H, H_8).

^{13}C NMR (126 MHz, $CDCl_3$) δ 198.2 (C), 168.4 (C), 156.3 (C), 139.6 (C), 136.7 (C), 133.9 (C), 131.4 (2 × CH), 131.1 (C), 130.8 (C), 129.3 (2 × CH), 115.3 (CH), 112.1 (CH), 110.1 (CH), 101.0 (CH), 55.9 (CH_3), 39.6 (CH_2), 13.5 (CH_3).

HRMS-CI (m/z): $[M + H]^+$ calcd for $C_{19}H_{17}ClNO_3$, 342.0897; found, 342.0876.

R_f = 0.27 (20% ethyl acetate-hexane; UV).

S-phenyl (2R,3R)-4-(1-(4-chlorobenzoyl)-5-methoxy-2-methyl-1H-indol-3-yl)-3-hydroxy-2-methylbutanethioate **6x**

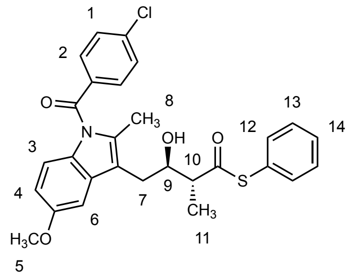

Following general method **B**, **6x** was prepared from **S10** (342 µL, 1.00 mmol, 1 equiv) and MAHT **2** (252 mg, 1.20 mmol, 1.20 equiv). The reaction was stirred for 24 h. The residue was purified by column chromatography (eluting with 5% ethyl acetate–hexane initially, grading to 40% ethyl acetate–hexane). The product was obtained as a white solid (491 mg, 0.97 mmol, 97%, 16:84 d.r. (syn:anti)). Recrystallization from ethanol (300 mL) provided the product in 4:96 d.r. (syn:anti) with 99:1 e.r. (anti).

^{1}H NMR (500 MHz, $CDCl_3$) δ 7.67 – 7.63 (m, 2H, H_2), 7.50 – 7.40 (m, 7H, $H_{1,12\text{-}14}$), 6.96 (d, *J* = 2.5 Hz, 1H, H_6), 6.90 (d, *J* = 9.0 Hz, 1H, H_3), 6.68 (dd, *J* = 9.0, 2.5 Hz, 1H, H_4), 4.11 (ddd, *J* = 8.7, 5.6, 4.5 Hz, 1H, H_9), 3.82 (s, 3H, H_5), 3.06 – 2.94 (m, 2H, $H_{7,10}$), 2.90 – 2.83 (m, 1H, H_7), 2.37 (s, 3H, H_8), 1.47 (d, *J* = 7.1 Hz, 3H, H_{11}).

^{13}C NMR (126 MHz, $CDCl_3$) δ 201.8 (C), 168.5 (C), 156.2 (C), 139.4 (C), 135.9 (C), 134.6 (2 × CH), 134.2 (C), 131.3 (2 × CH), 131.19 (C), 131.17 (C), 129.8 (CH), 129.4 (2 × CH), 129.3 (2 × CH), 127.3 (C), 115.8 (C), 115.2 (CH), 111.5 (CH), 101.8 (CH), 73.8 (CH), 55.9 (CH_3), 52.9 (CH), 30.0 (CH_2), 15.3 (CH_3), 13.9 (CH_3).

HPLC (AD-H, *n*-hexane/ ^{i}PrOH =85:15, 1.0 mL/min, 298 K, 254 nm): t_R(anti, major) = 22.4 min, t_R(syn, minor) = 23.8 min, t_R(anti, minor) = 28.6 min, t_R(syn, major) = 39.4 min, d.r. (syn:anti) = 4:96, e.r.(syn) = 98:2 (96% *ee*), e.r.(anti) = 99:1 (98% *ee*).
HRMS-CI (m/z): $[M + H]^+$ calcd for $C_{28}H_{27}ClNO_4S$, 508.1349; found, 508.1316.
R_f = 0.35 (20% ethyl acetate-hexane; UV).

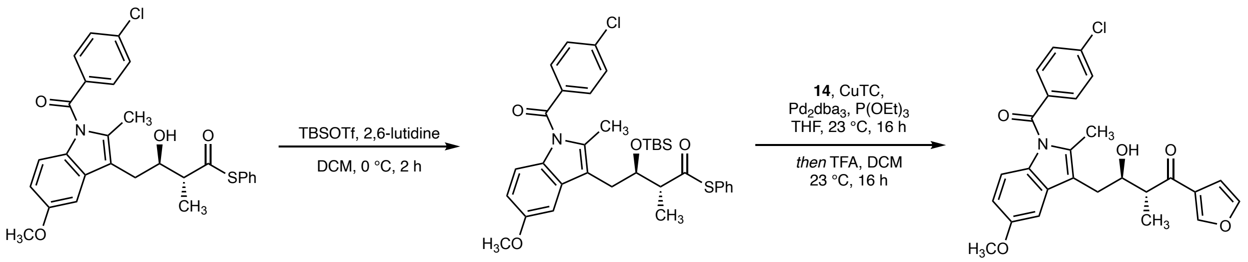

6x S11 16

S-phenyl(2R,3R)-3-((tert-butyldimethylsilyl)oxy)-4-(1-(4-chlorobenzoyl)-5-methoxy-2-methyl-1H-indol-3-yl)-2-methylbutane-thioate **S11**

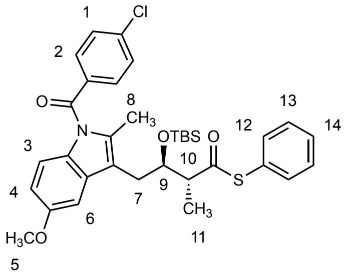

To a solution of thioester **6x** (324 mg, 0.64 mmol, 1 equiv) in dichloromethane (6.40 mL, 0.10 M) at 0 °C was added 2,6-lutidine (150 µL, 1.28 mmol, 2.00 equiv). To the mixture was added *t*-butyldimethylsilyl trifluoromethanesulfonate (220 µL, 0.96 mmol, 1.50 equiv) and the reaction mixture was stirred at 0 °C for 2 h. The reaction was quenched by the addition of an ammonium chloride solution and the organic layer was separated. The aqueous layer was extracted twice with dichloromethane. The combined organic extracts were washed with brine solution and dried over sodium sulfate. The dried solution was filtered and the filtrate was concentrated. The residue was purified by column chromatography (eluting with 5% ethyl acetate–hexane initially, grading to 40% ethyl acetate–hexane) to yield thioester **S11** as a yellow oil (400 mg, 99%).

^{1}H NMR (500 MHz, $CDCl_3$) δ 7.63 – 7.58 (m, 2H, H_2), 7.47 – 7.39 (m, 7H, $H_{1,12-14}$), 6.98 (d, *J* = 2.6 Hz, 1H, H_6), 6.80 (d, *J* = 9.0 Hz, 1H, H_3), 6.63 (dd, *J* = 9.0, 2.6 Hz, 1H, H_4), 4.49 (ddd, *J* = 8.3, 4.7, 3.7 Hz, 1H, H_9), 3.75 (s, 3H, H_5), 3.12 – 3.02 (m, 1H, H_{10}), 2.83 – 2.76 (m, 2H, H_7), 2.36 (s, 3H, H_8), 1.43 (d, *J* = 7.1 Hz, 3H, H_{11}), 0.83 (s, 9H, TBS), -0.02 (s, 3H, TBS), -0.43 (s, 3H, TBS).

^{13}C NMR (126 MHz, $CDCl_3$) δ 199.2 (C), 168.4 (C), 156.0 (C), 139.1 (C), 135.6 (C), 134.7 (2 × CH), 134.3 (C), 131.6 (C), 131.3 (2 × CH), 131.0 (C), 129.6 (CH), 129.4 (2 × CH), 129.2 (2 × CH), 127.6 (C), 116.9 (C), 115.1 (CH), 111.4 (CH), 101.7 (CH), 72.6 (CH), 55.8 (CH_3), 54.0 (CH), 27.6 (CH_2), 25.9 (3 × CH_3), 17.9 (C), 13.7 (CH_3), 9.9 (CH_3), -5.0 (CH_3), -5.1 (CH_3).

HRMS-CI (m/z): $[M + Na]^+$ calcd for $C_{34}H_{40}ClNO_4SSiNa$, 644.2034; found, 644.1993.
R_f = 0.64 (20% ethyl acetate-hexane; UV).

(2R,3R)-4-(1-(4-chlorobenzoyl)-5-methoxy-2-methyl-1H-indol-3-yl)-1-(furan-3-yl)-3-hydroxy-2-methylbutan-1-one **16**

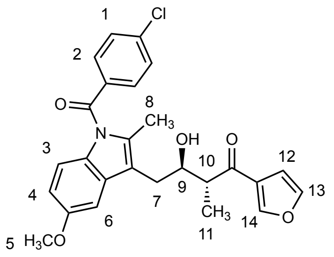

To a solution of thioester **S11** (229 mg, 0.39 mmol, 1 equiv), tris(dibenzylideneacetone)-dipalladium (17 mg, 0.02 mmol, 5 mol%), 3-furanylboronic acid **14** (62 mg, 0.55 mmol, 1.50 equiv) and copper(I) thiophene-2-carboxylate (140 mg, 0.74 mmol, 2.00 equiv) in tetrahydrofurane (11.0 mL, 0.03 M), was added triethyl phosphite (26 µL, 0.15 mmol, 0.40 equiv). The reaction was stirred at 23 °C for 16 h. The reaction was quenched by the addition of an ammonium chloride solution and the aqueous layer was extracted twice with ethyl acetate. The combined organic extracts were washed with a brine solution and dried over sodium sulfate. The dried solution was filtered and the filtrate was concentrated. To a solution of the crude ketone in dichloromethane (3.70 mL, 0.10 M) was added trifluoroacetic acid (141 µL, 1.84 mmol, 5.00 equiv). The reaction mixture was stirred at 23 °C for 16 h. The reaction mixture was quenched by addition of water and the organic layer was separated. The aqueous layer was extracted twice with dichloromethane. The combined organic extracts were washed with a brine solution and dried over sodium sulfate. The dried solution was filtered and the filtrate was concentrated. The residue was purified by column chromatography (eluting with 10% ethyl acetate–hexane initially, grading to 60% ethyl acetate–hexane) to yield ketone **16** as a white solid (144 mg, 84%).

^{1}H NMR (500 MHz, $CDCl_3$) δ 7.98 – 7.95 (m, 1H, H_{14}), 7.66 – 7.61 (m, 2H, H_2), 7.49 – 7.44 (m, 3H, $H_{1,12}$), 6.89 – 6.84 (m, 2H, $H_{3,6}$), 6.76 (dd, *J* = 1.9, 0.8 Hz, 1H, H_{13}), 6.65 (dd, *J* = 9.0, 2.6 Hz, 1H, H_4), 4.14 – 4.07 (m, 1H, H_9), 3.77 (s, 3H, H_5), 3.19 – 3.10 (m, 2H, $H_{10,OH}$), 3.01 – 2.87 (m, 2H, H_7), 2.26 (s, 3H, H_8), 1.40 (d, *J* = 7.3 Hz, 3H, H_{11}).

^{13}C NMR (126 MHz, $CDCl_3$) δ 200.1 (C), 168.4 (C), 156.1 (C), 148.0 (CH), 144.7 (CH), 139.4 (C), 135.7 (C), 134.2 (C), 131.3 (2 × CH), 131.2 (C), 131.1 (C), 129.3 (2 × CH), 127.6 (C), 116.2 (C), 115.1 (CH), 111.6 (CH), 108.8 (CH), 101.7 (CH), 74.2 (CH), 55.8 (CH_3), 47.5 (CH), 30.4 (CH_2), 15.9 (CH_3), 13.7 (CH_3).

HRMS-CI (m/z): $[M + H]^+$ calcd for $C_{26}H_{25}ClNO_5$, 466.1421; found, 466.1388.

R_f = 0.25 (20% ethyl acetate-hexane; UV).

(2R,3R)-8-((tert-butyldimethylsilyl)oxy)-1-(1-(4-chlorobenzoyl)-5-methoxy-2-methyl-1H-indol-3-yl)-2-hydroxy-3-methyloct-5-yn-4-oneas **17**

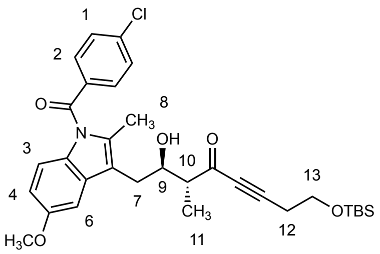

17

Thioester **6x** was azeotroped with toluene before use. To a solution of the dried thioester **6x** (100 mg, 0.19 mmol, 1 equiv), $Pd(dppf)Cl_2•CH_2Cl_2$ (14 mg, 0.02 mmol, 0.10 equiv), tri(2-furyl)phosphine (11 mg, 0.05 mmol, 0.25 equiv), and copper(I) iodide (75 mg, 0.39 mmol, 2.00 equiv) in dimethylformamide (0.45 mL, 0.44 M), was added *N*,*N*-diisopropylethylamine (40 µL, 0.20 mmol, 1.01 eq) and (but-3-yn-1-yloxy)(*tert*-butyl)dimethylsilane **15** (44 mg, 0.24 mmol, 1.20 equiv) sequentially. The reaction mixture was stirred at 50 °C for 24 h. Celite was added to the solution and the resulting suspension was stirred for 5 min. To the heterogenous mixture was added diethyl ether and brine solution. The heterogenous mixture was filtered through celite and rinsed with diethyl ether and ethyl acetate. The combined filtrates were dried over sodium sulfate and concentrated. The obtained residue was purified by column chromatography (eluting with 5% ethyl acetate-hexane initially, grading to 30% ethyl acetate-hexane) to give ketone **17** as a yellow liquid (106 mg, 93%).

^{1}H NMR (500 MHz, $CDCl_3$) δ 7.64 (d, *J* = 8.5 Hz, 2H, H_2), 7.45 (d, *J* = 8.5 Hz, 2H, H_1), 6.98 (d, *J* = 2.6 Hz, 1H, H_6), 6.86 (d, *J* = 9.0 Hz, 1H, H_3), 6.65 (dd, *J* = 9.0, 2.6 Hz, 1H, H_4), 4.16 – 4.11 (m, 1H, H_9), 3.83 (s, 3H, H_5), 3.76 (t, *J* = 6.8 Hz, 2H, H_{13}), 2.95 – 2.72 (m, 3H, $H_{7,10}$), 2.53 (d, *J* = 5.8 Hz, 1H, OH), 2.58 (t, *J* = 6.8 Hz, 2H, H_{12}), 2.34 (s, 3H, H_8), 1.37 (d, *J* = 7.2 Hz, 3H, H_{11}), 0.88 (s, 9H, TBS), 0.06 (s, 6H, TBS).

^{13}C NMR (126 MHz, $CDCl_3$) δ 190.9 (C), 168.4 (C), 156.1 (C), 139.3 (C), 135.8 (C), 134.1 (C), 131.3 (C), 131.2 (2 × CH), 131.1 (C), 129.2 (2 × CH), 116.0 (C), 115.1 (CH), 111.4 (CH), 101.7 (CH), 93.2 (C), 81.2 (C), 72.89 (CH), 60.81 (CH_2), 55.8 (CH_3), 53.2 (CH), 29.4 (CH_2), 25.9 (3 × CH_3), 23.5 (CH_2), 18.34(C), 13.7 (CH_3), 13.3 (CH_3), -5.3 (2 × CH_3).

HRMS-CI (m/z): $[M + Na]^+$ calcd for $C_{32}H_{40}ClNO_5SiNa$, 604.2262; found, 604.2250.

R_f = 0.24 (20% ethyl acetate-hexane; UV).

Synthetic procedures for the synthesis of salen ligands:

6,6'-((1E,1'E)-(((1R,2R)-cyclohexane-1,2-diyl)bis(azaneylylidene))bis(methaneylylidene))bis(4-chloro-2-iodophenol) **3g**

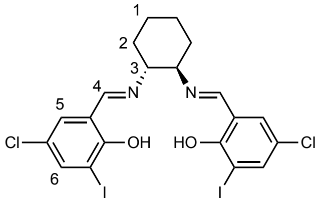

Following general method **C**, **3g** was prepared from (1*R*,2*R*)-cyclohexane-1,2-diamine (136 mg, 1.19 mmol, 1 equiv) and 5-chloro-2-hydroxy-3-iodobenzaldehyde (690 mg, 2.44 mmol, 2.05 equiv). The reaction was stirred for 12 h at 60 °C. The product was obtained as a yellow solid (677 mg, 88%).

^{1}H NMR (500 MHz, $CDCl_3$) δ 14.46 (s, 2H, OH), 8.07 (s, 2H, H_4), 7.70 (d, J = 2.5 Hz, 2H, H_5), 7.14 (d, J = 2.5 Hz, 2H, H_6), 3.41 – 3.30 (m, 2H, H_3), 1.99 – 1.82 (m, 4H, H_2), 1.75 – 1.61 (m, 2H, H_1), 1.52 – 1.38 (m, 2H, H_1).

^{13}C NMR (126 MHz, $CDCl_3$) δ 163.3 (2 × C), 159.8 (2 × C), 141.0 (2 × CH), 131.1 (2 × CH), 123.9 (2 × C), 118.0 (2 × C), 86.5 (2 × CH), 72.0 (2 × CH), 33.0 (2 × CH_2), 24.0 (2 × CH_2).

HRMS-CI (m/z): $[M + H]^+$ calcd for $C_{20}H_{19}Cl_2I_2N_2O_2$, 642.8912; found, 642.8905.

R_f = 0.50 (50% ethyl acetate-hexane; UV).

6,6'-((1E,1'E)-(((1R,2R)-cyclohexane-1,2-diyl)bis(azaneylylidene))bis(methaneylylidene))bis(2-isopropoxyphenol) **3n**

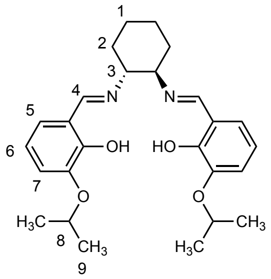

Following general method **C**, **3n** was prepared from (1*R*,2*R*)-cyclohexane-1,2-diamine (20 mg, 0.17 mmol, 1 equiv) and 2-hydroxy-3-isopropoxybenzaldehyde (65 mg, 0.36 mmol, 2.05 equiv). The reaction was stirred for 12 h at 60 °C. The product was obtained as a yellow solid (62 mg, 80%).

^{1}H NMR (500 MHz, $CDCl_3$) δ 13.80 (s, 2H, OH), 8.23 (s, 2H, H_4), 6.87 (dd, J = 7.8, 1.5 Hz, 2H, H_5), 6.77 (dd, J = 7.8, 1.5 Hz, 2H, H_7), 6.69 (t, J = 7.8 Hz, 2H, H_6), 4.60 – 4.46 (m, J = 6.4 Hz, 2H, H_8), 3.34 – 3.18 (m, 2H, H_3), 1.96 – 1.79 (m, 4H, H_2), 1.74 – 1.60 (m, 2H, H_1), 1.50 – 1.41 (m, 2H, H_1), 1.35 (d, J = 6.0 Hz, 12H, H_9).

^{13}C NMR (126 MHz, $CDCl_3$) δ 164.9 (2 × C), 152.7 (2 × C), 146.3 (2 × C), 123.7 (2 × CH), 119.0 (2 × CH), 118.3 (2 × CH), 117.9 (2 × CH), 72.6 (2 × CH), 71.2 (2 × CH), 33.1 (2 × CH_2), 24.2 (2 × CH_2), 22.2 (4 × CH_3).

HRMS-CI (m/z): $[M + H]^+$ calcd for $C_{26}H_{35}N_2O_4$, 439.2596; found, 439.2602.

R_f = 0.62 (40% ethyl acetate-hexane; UV).

6,6'-((1*E*,1'*E*)-(((1*R*,2*R*)-cyclohexane-1,2-diyl)bis(azaneylylidene))bis(methaneylylidene))bis(2-(benzyloxy)phenol) **3o**

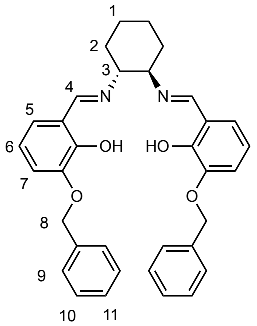

Following general method C, **3o** was prepared from (1*R*,2*R*)-cyclohexane-1,2-diamine (20 mg, 0.17 mmol, 1.0 equiv) and 3-(benzyloxy)-2-hydroxybenzaldehyde (82 mg, 0.36 mmol, 2.05 equiv), the reaction was stirred for 12 h at 60 ºC. The solid residue was filtered and dried over high vacuum to obtained as a yellow solid (51 mg, 0.09 mmol, 54 %).

^{1}H NMR (500 MHz, $CDCl_3$) δ 13.76 (s, 2H), 8.19 (s, 2H, H_4), 7.37 (dt, J = 7.7, 1.7 Hz, 4H, H_{arom}), 7.34 – 7.17 (m, 6H, H_{arom}), 6.84 – 6.78 (m, 2H, H_{arom}), 6.75 (d, J = 7.8 Hz, 2H, H_{arom}), 6.59 (t, J = 7.9 Hz, 2H, H_{arom}), 5.06 (s, 4H, H_8), 3.31 – 3.22 (m, 2H, H_3), 1.91 – 1.76 (m, 4H, H_2), 1.71 – 1.55 (m, 2H, H_1), 1.46 – 1.32 (m, 2H, H_1).

^{13}C NMR (126 MHz, $CDCl_3$) δ 164.96 (2 × C), 152.34 (2 × C), 147.37 (2 × C), 137.32 (2 × CH), 128.65 (4 × CH), 127.94 (2 × CH), 127.55 (4 × CH), 123.99 (2 × CH), 118.89 (2 × C), 117.96 (2 × CH), 116.90 (2 × CH), 72.59 (2 × CH_2), 71.27 (2 × CH), 33.21 (2 × CH_2), 24.23 (2 × CH_2).

HRMS-CI (m/z): $[M + Na]^+$ calcd for $C_{34}H_{34}N_2O_4Na$, 557.2416; found, 557.2413.
R_f = 0.50 (40% ethyl acetate-hexane; UV).

6,6'-((1E,1'E)-(((1R,2R)-cyclohexane-1,2-diyl)bis(azaneylylidene))bis(methaneylylidene))bis(3-(diethylamino)phenol) **3r**

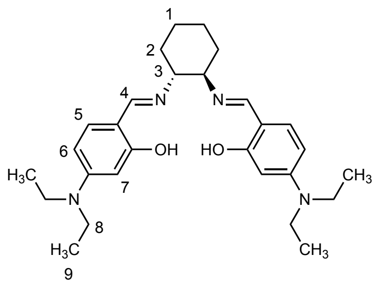

Following general method **C**, **3r** was prepared from (1*R*,2*R*)-cyclohexane-1,2-diamine (110 mg, 0.96 mmol, 1 equiv) and 4-(diethylamino)-2-hydroxybenzaldehyde (382 mg, 1.97 mmol, 2.05 equiv). The reaction was stirred for 12 h at 60 °C. The product was obtained as a yellow solid (351 mg, 78%).

^{1}H NMR (500 MHz, $CDCl_3$) δ 7.92 (s, 2H, H_4), 6.88 (d, *J* = 8.7 Hz, 2H, H_7), 6.06 – 6.02 (m, 4H, $H_{5,6}$), 3.23 (q, *J* = 7.1 Hz, 8H, H_8), 3.14 – 2.98 (m, 2H, H_3), 1.84 (d, *J* = 13.3 Hz, 2H, H_2), 1.73 (d, *J* = 8.8 Hz, 2H, H_2), 1.54 (q, *J* = 11.4 Hz, 2H, H_1), 1.33 (t, *J* = 10.3 Hz, 2H, H_1), 1.06 (t, *J* = 7.1 Hz, 12H, H_9).

^{13}C NMR (126 MHz, $CDCl_3$) δ 166.1 (2 × C), 162.77 (2 × C), 151.5 (2 × C), 133.1 (2 × CH), 108.3 (2 × C), 103.0 (2 × CH), 98.2 (2 × CH), 70.8 (2 × CH), 44.5 (4 × CH_2), 33.3 (2 × CH_2), 24.7 (2 × CH_2), 12.7 (4 × CH_3).
HRMS-CI (m/z): $[M + H^+]$ calcd for $C_{28}H_{41}N_4O_2$, 465.3229; found, 465.3233.
R_f = 0.52 (40% ethyl acetate-hexane; UV).

Synthetic procedures for the synthesis of salalen ligands:

2-((E)-(((1S,2S)-2-((2-hydroxy-3-methoxybenzyl)amino)cyclohexyl)imino)methyl)-6-methoxyphenol **4a**

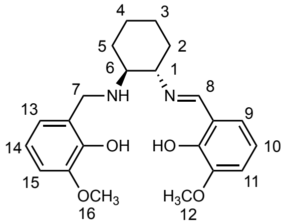

Following general method **D**, **4a** was prepared from **3a** (3.00 g, 7.84 mmol, 1 equiv) and sodium triacetoxyborohydride (1.83 g, 8.63 mmol, 1.10 equiv), the reaction was stirred for 16 h. The residue was purified by column chromatography (eluting with 20% ethyl acetate–hexane initially, grading to 100% ethyl acetate). The product was obtained as a yellow solid (1.72 g, 57%).

Major diastereomer:

^{1}H NMR (500 MHz, $CDCl_3$) δ 13.60 (s, 2H, OH), 8.37 (s, 1H, H_8), 6.89 (dd, *J* = 7.9, 1.6 Hz, 1H, H_{arom}), 6.85 (dd, *J* = 7.9, 1.6 Hz, 1H, H_{arom}), 6.78 (t, *J* = 7.8 Hz, 1H, H_{arom}), 6.72 (dd, *J* = 8.1, 1.5 Hz, 1H, H_{arom}), 6.66 (t, *J* = 7.8 Hz, 1H, H_{arom}), 6.54 (dd, *J* = 7.6, 1.5 Hz, 1H, H_{arom}), 3.98 (d, *J* = 13.9 Hz, 1H, H_7), 3.88 (d, *J* = 7.5 Hz, 1H, H_7), 3.85 (s, 3H, H_{12}), 3.79 (s, 3H, H_{16}), 3.05 (ddd, *J* = 10.8, 8.9, 4.0 Hz, 1H, H_1), 2.75 (ddd, *J* = 10.5, 8.9, 3.9 Hz, 1H, H_6), 2.16 – 2.07 (m, 1H, H_5), 1.81 – 1.67 (m, 3H, $H_{2,3,4}$), 1.65 – 1.52 (m, 1H, H_2), 1.42 – 1.30 (m, 2H, $H_{3,4}$), 1.29 – 1.18 (m, 1H, H_5).

^{13}C NMR (126 MHz, $CDCl_3$) δ 165.7 (C), 151.4 (C), 148.3 (C), 148.1 (C), 147.0 (C), 123.6 (CH), 123.2 (CH), 120.3 (CH), 118.7 (CH), 118.5 (C), 118.2 (CH), 114.2 (CH), 110.8 (CH), 73.1 (CH), 61.2 (CH), 56.1 (CH_3), 55.8 (CH_3), 49.7 (CH_2), 33.7 (CH_2), 29.9 (CH_2), 24.2 (CH_2), 24.1 (CH_2).

HRMS-CI (m/z): $[M + H]^+$ calcd for $C_{22}H_{29}N_2O_4$, 385.2127; found, 385.2120.

R_f = 0.38 (60% ethyl acetate-hexane; UV).

The (R,R)-salalen* ***4a* *ligand was prepared accordingly using (R,R)-salen* ***3a****.*

*2,4-di-tert-butyl-6-((E)-(((1S,2S)-2-((3,5-di-tert-butyl-2-hydroxybenzyl)amino)cyclohexyl)imino)methyl)*phenol **4b**

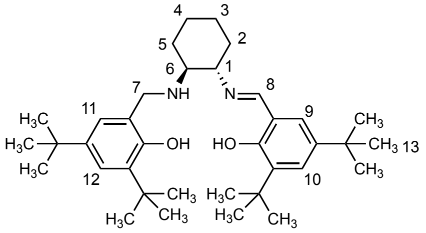

Following general method **D**, **4b** was prepared from **3j** (500 mg, 0.91 mmol, 1 equiv) and sodium triacetoxyborohydride (214 mg, 1.01 mmol, 1.10 equiv), the reaction was stirred for 16 h at 23 °C. The residue was purified by column chromatography (eluting with 20% ethyl acetate–hexane initially, grading to 100% ethyl acetate). The product was obtained as a yellow solid (132 mg, 26%). ^{1}H and ^{13}C NMR data for **4b** prepared in this way were in agreement with the reported data for (*R*,*R*)-**4b**.(*56*)

^{1}H NMR (500 MHz, $CDCl_3$) δ 13.47 (s, 1H), 8.41 (s, 1H, OH), 7.38 (d, *J* = 2.4 Hz, 1H, H_8), 7.17 (d, *J* = 2.5 Hz, 1H, H_{arom}), 7.06 (d, *J* = 2.4 Hz, 1H, H_{arom}), 6.82 (d, *J* = 2.4 Hz, 1H, H_{arom}), 4.04 (d, 1H, H_7), 3.81 (d, *J* = 13.2 Hz, 1H, H_7), 3.04 (ddd, *J* = 10.8, 9.0, 4.1 Hz, 1H, H_1), 2.81 (ddd, *J* = 10.8, 9.1, 4.0 Hz, 1H, H_6), 2.25 (d, *J* = 12.7 Hz, 1H, H_2), 1.81 (d, *J* = 12.2 Hz, 3H, $H_{3,4,5}$), 1.76 – 1.63 (m, 1H, H_5), 1.44 (s, 9H, H_{13}), 1.34 (s, 9H, H_{13}), 1.29 (s, 13H, $H_{2,3,4,13}$), 1.24 (s, 9H).

^{13}C NMR (126 MHz, $CDCl_3$) δ 166.7 (C), 158.1 (C), 154.6 (C), 140.5 (C), 140.3 (C), 136.7 (C), 136.0 (C), 127.3 (CH), 126.2 (CH), 123.2 (CH), 123.0 (CH), 117.9 (CH), 74.0 (CH), 61.2 (CH), 50.9 (CH_2), 35.1 (C), 35.0 (C), 34.2 (C), 34.1 (CH_2), 31.8 (CH_3), 31.8 (CH_3), 31.6 (CH_3),30.5 (CH_2), 29.8 (CH_3), 29.7 (CH_3), 29.6 (CH_3), 24.7 (CH_2), 24.6 (CH_2).

HRMS-CI (m/z): $[M + Na]^+$ calcd for $C_{36}H_{56}N_2O_2Na$, 571.4239; found, 571.4245.

R_f = 0.55 (50% ethyl acetate-hexane; UV).

3-((E)-(((1S,2S)-2-(((2-hydroxy-[1,1'-biphenyl]-3-yl)methyl)amino)cyclohexyl)imino)methyl)-[1,1'-biphenyl]-2-ol **4c**

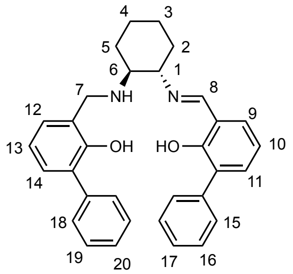

Following general method **D**, **4c** was prepared from **3p** (500 mg, 1.05 mmol, 1 equiv) and sodium triacetoxyborohydride (245 mg, 1.16 mmol, 1.10 equiv), the reaction was stirred for 16 h. The residue was purified by column chromatography (eluting with 20% ethyl acetate–hexane initially, grading to 100% ethyl acetate). The product was obtained as a yellow solid (147 mg, 29%).

^{1}H and ^{13}C NMR data for **4b** prepared in this way were in agreement with the reported data for (*R*,*R*)-**4c**.(*57*)

^{1}H NMR (500 MHz, $CDCl_3$) δ 13.67 (s, 1H, OH), 8.37 (s, 1H, H_8), 7.54 – 7.50 (m, 2H, H_{arom}), 7.48 – 7.44 (m, 2H, H_{arom}), 7.38 – 7.24 (m, 6H, H_{arom}), 7.23 – 7.19 (m, 1H, H_{arom}), 7.18 – 7.15 (m, 2H), 7.13 (dd, *J* = 7.6, 1.7 Hz, 1H, H_{arom}), 6.91 – 6.85 (m, 2H, H_{arom}), 6.73 (t, *J* = 7.5 Hz, 1H, H_{arom}), 4.02 (d, *J* = 13.7 Hz, 1H, H_7), 3.82 (d, *J* = 13.7 Hz, 1H, H_7), 2.98 (ddd, *J* = 10.9, 9.0, 4.2 Hz, 1H, H_1), 2.70 (ddd, *J* = 10.7, 9.1, 4.0 Hz, 1H, H_6), 2.14 (ddd, *J* = 14.7, 5.5, 3.0 Hz, 1H, H_5), 1.79 – 1.65 (m, 3H, $H_{2,4}$), 1.65 – 1.48 (m, 1H, H_2), 1.28 (tt, *J* = 10.2, 2.9 Hz, 2H, H_3), 1.24 – 1.11 (m, 1H, H_5).

δ 13C NMR (126 MHz, CDCl3) δ 166.1 (C), 158.3 (C), 155.1 (C), 138.6 (C), 137.7 (C), 133.6 (CH), 131.13 (CH), 129.9 (C), 129.8 (CH), 129.43 (2 × CH), 129.40 (2 × CH), 129.3 (C), 128.2 (2 × CH), 128.1 (2 × CH), 127.5 (CH), 127.3 (CH), 126.8 (CH), 123.7 (CH), 119.1 (CH), 118.9 (CH), 118.8 (C), 77.4 (CH), 61.4 (CH), 50.3 (CH_2), 34.0 (CH_2), 30.1 (CH_2), 24.44 (CH_2), 24.36 (CH_2).

HRMS-CI (m/z): $[M + Na]^+$ calcd for $C_{32}H_{32}N_2O_2Na$, 499.2361; found, 499.2364.

R_f = 0.45 (50% ethyl acetate-hexane; UV).

Synthetic procedures for synthesis of substituted meldrum's acid:

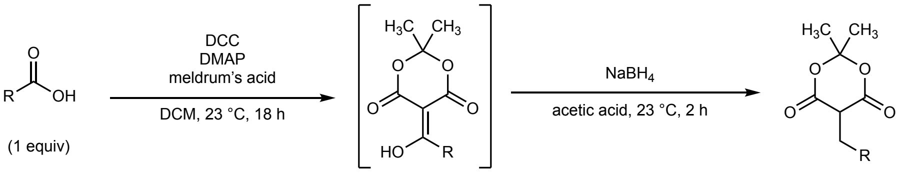

S12 (a-e,g)

S12a: R = CH_3
S12b: R = $(CH_2)_3CH_3$
S12c: R = $(CH_2)_2Ph$
S12d: R = CH_2OBn
S12e: R = $CH_2CH{=}CH_2$
S12g: R = $CH_2C(O)OCH_3$

5-ethyl-2,2-dimethyl-1,3-dioxane-4,6-dione **S12a**

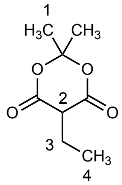

To the stirred solution of meldrum's acid (6.00 g, 41.6 mmol, 1 equiv) in dichloromethane (83.0 mL, 0.50 M) was added acetic acid (2.6 mL, 45.8 mmol, 1.10 equiv) and *N*,*N*-dimethylaminopyridine (5.34 g, 43.7 mmol, 1.05 equiv) at 0 °C. After 5 min a solution of *N,N*-dicyclohexylcarbodiimide (10.30 g, 49.9 mmol, 1.20 equiv) in dichloromethane (83.0 mL, 0.50 M) was added to the reaction mixture. The reaction was stirred at 23 °C for 18 h. Upon completion of the reaction the insoluble urea was removed by filtration through a pad of celite. The filter cake was washed with dichloromethane. The combined filtrates were washed twice with a 5% aqueous potassium hydrogen sulfite solution and once with brine solution. The organic layer was dried over sodium sulfate, concentrated and dried over high vacuum. To a solution of the obtained acylated meldrum's acid intermediate in acetic acid (66.0 mL, 0.70 M) was added sodium borohydride (2.62 g, 69.1 mmol, 1.50 equiv) portion wise over 30 min at 0 °C. The reaction mixture was stirred for 1 h at 23 °C. The product mixture was quenched by the addition of an 10% aqueous hydrogen chloride solution at 0 °C. The mixture was diluted with dichloromethane and the organic layer was separated. The aqueous layer was washed twice with dichloromethane and the combined organic extracts were dried over sodium sulfate. The filtrate was concentrated and the residue was purified by column chromatography (eluting with 5% ethyl acetate–hexane initially, grading to 70% ethyl acetate–hexane). The product was obtained as white solid (6.91 g, 87%).

^{1}H and ^{13}C NMR data for **S12a** prepared in this way were in agreement with the literature.(*58*)

^{1}H NMR (500 MHz, $CDCl_3$) δ 3.50 (t, *J* = 4.9 Hz, 1H, H_2), 2.15 (qd, *J* = 7.4, 4.9 Hz, 2H, H_3), 1.77 (s, 3H, H_1), 1.74 (s, 3H, H_1), 1.03 (t, *J* = 7.4 Hz, 3H, H_4).

^{13}C NMR (126 MHz, $CDCl_3$) δ 165.6 (2 × C), 104.9 (C), 47.2 (CH), 28.5 (CH_3), 27.0 (CH_3), 20.1 (CH_2), 10.8 (CH_3).

HRMS-CI (m/z): $[M + H]^+$ calcd for $C_8H_{13}O_4$, 173.0813; found, 173.0803.

R_f = 0.40 (30% ethyl acetate-hexane; UV).

2,2-dimethyl-5-pentyl-1,3-dioxane-4,6-dione **S12b**

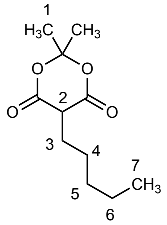

To the stirred solution of meldrum's acid (5.00 g, 34.7 mmol, 1 equiv) in dichloromethane (69.4 mL, 0.50 M) was added valeric acid (4.50 mL, 41.6 mmol, 1.20 equiv) and dimethylaminopyridine (4.45 g, 36.4 mmol, 1.05 equiv) at 0 °C. After 5 min a solution of *N,N*-dicyclohexylcarbodiimide (8.59 g, 41.6 mmol, 1.20 equiv) in dichloromethane (69.4 mL, 0.50 M) was added to the reaction mixture and stirred at 23 °C for 18 h. Upon completion of the reaction the insoluble urea was removed by filtration through a pad of celite. The filter cake was washed with dichloromethane. The combined filtrates were washed twice with a 5% aqueous potassium hydrogen sulfite solution and once with brine solution. The organic layer was dried over sodium sulfate, concentrated and dried over high vacuum. To a solution of the obtained acylated meldrum's acid intermediate in acetic acid (53.6 mL, 0.70 M) was added sodium borohydride (2.13 g, 56.3 mmol, 1.50 equiv) portion wise over 30 min at 0 °C. The reaction mixture was stirred for 1 h at 23 °C. The product mixture was quenched by the addition of an 10% aqueous hydrogen chloride solution at 0 °C. The mixture was diluted with dichloromethane and the organic layer was separated. The aqueous layer was washed twice with dichloromethane and the combined organic extracts were dried over sodium sulfate. The filtrate

was concentrated and the residue was purified by column chromatography (eluting with 5% ethyl acetate–hexane initially, grading to 50% ethyl acetate–hexane). The product was obtained as white solid (6.62g, 82%)

^{1}H and ^{13}C NMR data for **S12b** prepared in this way were in agreement with the literature.(*59*)

^{1}H NMR (500 MHz, $CDCl_3$) δ 3.50 (t, *J* = 5.0 Hz, 1H, H_2), 2.11 – 2.00 (m, 2H, H_3), 1.76 (s, 3H, H_1), 1.73 (s, 3H, H_1), 1.46 – 1.38 (m, 2H, H_4), 1.35 – 1.25 (m, 4H, $H_{5,6}$), 0.92 – 0.81 (m, 3H, H_7).

^{13}C NMR (126 MHz, $CDCl_3$) δ 165.8 (2 × C), 104.8 (C), 46.2 (CH), 31.7 (CH_2), 28.5 (CH_3), 27.0 (CH_3), 26.6 (CH_2), 26.2 (CH_2), 22.4 (CH_2), 14.0 (CH_3).

HRMS-CI (m/z): $[M + Na]^+$ calcd for $C_{11}H_{18}O_4Na$, 237.1102; found, 237.1103.

R_f = 0.53 (30% ethyl acetate-hexane; UV).

2,2-dimethyl-5-(3-phenylpropyl)-1,3-dioxane-4,6-dione **S12c**

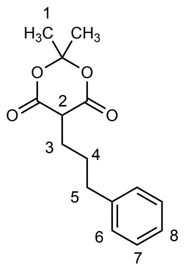

To the stirred solution of meldrum's acid (4.80 g, 33.3 mmol, 1 equiv) in dichloromethane (66.7 mL, 0.50 M) was added hydrocinnamic acid (5.00 g, 33.3 mmol, 1.00 equiv) and dimethylaminopyridine (4.27 g, 35.0 mmol, 1.05 equiv) at 0 °C. After 5 min a solution of *N,N*-dicyclohexylcarbodiimide (8.24 g, 33.3 mmol, 1.20 equiv) in dichloromethane (66.7 mL, 0.50 M) was added to the reaction mixture and stirred at 23 °C for 18 h. Upon completion of the reaction the insoluble urea was removed by filtration through a pad of celite. The filter cake was washed with dichloromethane. The combined filtrates were washed twice with a 5% aqueous potassium hydrogen sulfite solution and once with brine solution. The organic layer was dried over sodium sulfate, concentrated and dried over high vacuum. To a solution of the obtained acylated meldrum's acid intermediate in acetic acid (46.6 mL, 0.70 M) was added sodium borohydride (1.85 g, 48.97 mmol, 1.50 equiv) portion wise over 30 min at 0 °C. The reaction mixture was stirred for 1 h at 23 °C. The product mixture was quenched by the addition of an 10% aqueous hydrogen chloride solution at 0 °C. The mixture was diluted with dichloromethane and the organic layer was separated. The aqueous layer was washed twice with dichloromethane and the combined organic extracts were dried over sodium sulfate. The filtrate was concentrated and the residue was purified by column chromatography (eluting with 5% ethyl acetate–hexane initially, grading to 70% ethyl acetate–hexane). The product was obtained as white solid (7.47 g, 92%)

^{1}H and ^{13}C NMR data for **S12c** prepared in this way were in agreement with the literature.(*60*)

^{1}H NMR (500 MHz, $CDCl_3$) δ 7.28 (dd, *J* = 8.2, 7.1 Hz, 2H, H_7), 7.22 – 7.15 (m, 3H, $H_{6,8}$), 3.50 (t, *J* = 5.1 Hz, 1H, H_2), 2.68 (t, *J* = 7.7 Hz, 2H, H_5), 2.19 – 2.10 (m, 2H, H_3), 1.84 – 1.78 (m, 2H, H_4), 1.72 (s, 6H, H_1).

^{13}C NMR (126 MHz, $CDCl_3$) δ 165.5 (2 × C), 141.5 (C), 128.4 (2 × CH), 128.4 (2 × CH), 126.0 (CH), 104.9 (C), 46.1 (CH), 35.8 (CH_2), 28.4 (CH_2), 28.1 (CH_2), 26.7 (CH_3), 26.2 (CH_3).

HRMS-CI (m/z): $[M + H]^+$ calcd for $C_{15}H_{19}O_4$, 263.1283; found, 263.1273.

R_f = 0.40 (30% ethyl acetate-hexane; UV).

5-(2-(benzyloxy)ethyl)-2,2-dimethyl-1,3-dioxane-4,6-dione **S12d**

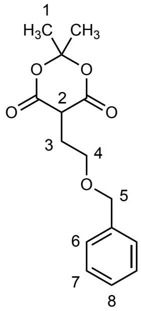

To the stirred solution of meldrum's acid (4.00 g, 27.8 mmol, 1 equiv) in dichloromethane (55.0 mL, 0.50 M) was added benzyloxy acetic acid (4.61 g, 27.8 mmol, 1.00 equiv) and *N*,*N*-dimethylaminopyridine (3.56 g, 29.1 mmol, 1.05 equiv) at 0 °C. After 5 min a solution of *N,N*-dicyclohexylcarbodiimide (6.87 g, 33.3 mmol, 1.20 equiv) in dichloromethane (55.0 mL, 0.50 M) was added to the reaction mixture and stirred at 23 °C for 18 h. Upon completion of the reaction the insoluble urea was removed by filtration through a pad of celite. The filter cake was washed with dichloromethane. The combined filtrates were washed twice with a 5% aqueous potassium hydrogen sulfite solution and once with brine solution. The organic layer was dried over sodium sulfate, concentrated and dried over high vacuum. To a solution of the obtained acylated meldrum's acid intermediate in acetic acid (44.0 mL, 0.70 M) was added sodium borohydride (1.77 g, 46.7 mmol,

1.50 equiv) portion wise over 30 min at 0 °C. The reaction mixture was stirred for 1 h at 23 °C. The product mixture was quenched by the addition of an 10% aqueous hydrogen chloride solution at 0 °C. The mixture was diluted with dichloromethane and the organic layer was separated. The aqueous layer was washed twice with dichloromethane and the combined organic extracts were dried over sodium sulfate. The filtrate was concentrated and the residue was purified by column chromatography (eluting with 5% ethyl acetate–hexane initially, grading to 70% ethyl acetate–hexane). The product was obtained as white solid (6.35 g, 73%)

^{1}H NMR (500 MHz, $CDCl_3$) δ 7.37 – 7.26 (m, 5H, H_{6-8}), 4.48 (s, 2H, H_5), 3.78 – 3.73 (m, 3H, $H_{2,4}$), 2.49 – 2.40 (m, 2H, H_3), 1.76 (s, 3H, H_1), 1.69 (s, 3H, H_1).

^{13}C NMR (126 MHz, $CDCl_3$) δ 165.8 (2 × C), 138.1 (C), 128.5 (2 × CH), 127.8 (CH), 127.7 (2 × CH), 105.0 (C), 73.1 (CH_2), 66.1 (CH_2), 42.4 (CH), 28.4 (CH_2), 26.8 (CH_3), 26.4 (CH_3).

HRMS-CI (m/z): $[M + Na]^+$ calcd for $C_{15}H_{18}O_5Na$, 301.1052; found, 301.1040.

R_f = 0.30 (30% ethyl acetate-hexane; UV).

Synthetic procedures for the synthesis of substituted MAHTs:

2-((phenylthio)carbonyl)butanoic acid **S2a**

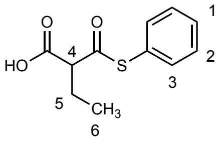

Following general method **E**, **S2a** was prepared from **S12a** (1.83 g, 10.6 mmol, 1 equiv), *N*,*N*-diisopropylethylamine (2.00 mL, 11.7 mmol, 1.10 equiv), trimethylsilyl chloride (1.50 mL, 11.7 mmol, 1.10 equiv), and thiophenol (1.10 mL, 6.40 mmol, 1.05 equiv). The residue was purified by column chromatography (eluting with 5% ethyl acetate–hexane initially, grading to 80% ethyl acetate–hexane). The product was obtained as a white solid (2.27 g, 95%).

^{1}H and ^{13}C NMR data for **S2a** prepared in this way were in agreement with the literature.(*61*)

^{1}H NMR (500 MHz, CDCl$_3$) δ 7.48 – 7.39 (m, 5H, H$_{1\text{-}3}$), 3.65 (t, *J* = 7.4 Hz, 1H, H$_4$), 2.13 – 1.97 (m, 2H, H$_5$), 1.06 (t, *J* = 7.4 Hz, 3H, H$_6$).

^{13}C NMR (126 MHz, CDCl$_3$) δ 193.3 (C), 174.3 (C), 134.6 (2 × CH), 123.0 (CH), 129.5 (2 × CH), 126.8 (C), 60.8 (CH), 23.3 (CH$_2$), 11.9 (CH$_3$).

HRMS-CI (m/z): [M + Na]$^+$ calcd for $C_{11}H_{12}O_3SNa$, 247.0405; found, 247.0393.

R$_f$ = 0.30 (50% ethyl acetate-hexane; UV).

2-((phenylthio)carbonyl)heptanoic acid **S2b**

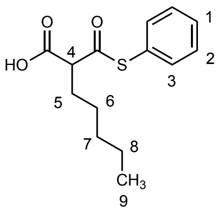

Following general method **E**, **S2b** was prepared from **S12b** (1.50 g, 7.00 mmol, 1 equiv.), *N*,*N*-diisopropylethylamine (1.30 mL, 7.70 mmol, 1.10 equiv.), trimethylsilyl chloride (977 µL, 7.70 mmol, 1.10 equiv.), and thiophenol (750 µL, 7.36 mmol, 1.05 equiv.). The residue was purified by column chromatography (eluting with 5% ethyl acetate–hexane initially, grading to 80% ethyl acetate–hexane). The product was obtained as a white solid (1.47 g, 79%).

^{1}H NMR (500 MHz, CDCl$_3$) δ 7.50 – 7.34 (m, 5H, H$_{1,2,3}$), 3.71 (t, *J* = 7.4 Hz, 1H, H$_4$), 2.05 – 1.96 (m, 2H, H$_5$), 1.47 – 1.38 (m, 2H, H$_6$), 1.38-1.28 (m, 4H,H$_{7,8}$), 0.91 (t, 3H, H$_9$).

^{13}C NMR (126 MHz, CDCl$_3$) δ 193.4 (C), 174.5 (C), 134.6 (2 × CH), 129.9 (C), 129.5 (2 × CH), 126.9 (C), 59.3 (CH), 31.5 (CH$_2$), 29.8 (CH$_2$), 27.0 (CH$_2$), 22.4 (CH$_2$), 14.1 (CH$_3$).

HRMS-CI (m/z): [M + H]$^+$ calcd for $C_{14}H_{19}O_3S$, 267.1055; found, 267.1073.

R$_f$ = 0.35 (40% ethyl acetate-hexane; UV).

5-phenyl-2-((phenylthio)carbonyl)pentanoic acid **S2c**

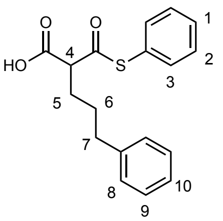

Following general method **E**, **S2c** was prepared from **S12c** (4.50 g, 17.2 mmol, 1 equiv.), *N*,*N*-diisopropylethylamine (3.30 mL, 18.9 mmol, 1.10 equiv.), trimethylsilyl chloride (2.40 mL, 18.9 mmol, 1.10 equiv.), and thiophenol (1.80 mL, 18.0 mmol, 1.05 equiv.). The residue was purified by column chromatography (eluting with 5% ethyl acetate–hexane initially, grading to 80% ethyl acetate–hexane). The product was obtained as a colorless oil (4.46 g, 82%).

^{1}H NMR (500 MHz, CDCl$_3$) δ 7.44 (s, 5H, H$_{1\text{-}3}$), 7.33 – 7.27 (m, 2H, H$_9$), 7.24 – 7.17 (m, 3H, H$_{8,10}$), 3.73 (t, *J* = 7.4 Hz, 1H, H$_4$), 2.72 – 2.65 (m, 2H, H$_7$), 2.10 – 2.01 (m, 2H, H$_5$), 1.82 – 1.70 (m, 2H, H$_6$).

^{13}C NMR (126 MHz, CDCl$_3$) δ 193.2 (C), 174.1 (C), 141.5 (C), 134.6 (2 × CH), 130.0 (CH), 129.5 (2 × CH), 128.6 (2 × CH), 128.5 (2 × CH), 126.8 (C), 126.1 (CH), 59.1 (CH), 35.6 (CH$_2$), 29.3 (CH$_2$), 29.0 (CH$_2$).

HRMS-CI (m/z): [M + Na]$^+$ calcd for $C_{18}H_{18}O_3SNa$, 337.0874; found, 337.0868.

R$_f$ = 0.30 (50% ethyl acetate-hexane; UV).

4-(benzyloxy)-2-((phenylthio)carbonyl)butanoic acid **S2d**

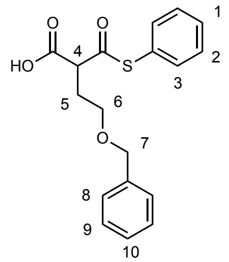

Following general method **E**, **S2d** was prepared from **S12d** (3.00 g, 10.8 mmol, 1 equiv), *N*,*N*-diisopropylethylamine (2.10 mL, 11.9 mmol, 1.10 equiv), trimethylsilyl chloride (1.50 mL, 11.9 mmol, 1.10 equiv), and thiophenol (1.20 mL, 11.3 mmol, 1.05 equiv). The residue was purified by column chromatography (eluting with 5% ethyl acetate–hexane initially, grading to 80% ethyl acetate–hexane). The product was obtained as a colorless oil (3.11 g, 87%).

^{1}H NMR (500 MHz, $CDCl_3$) δ 7.45 – 7.38 (m, 5H, $H_{1\text{-}3}$), 7.38 – 7.27 (m, 5H, $H_{8\text{-}10}$), 4.53 (s, 2H, H_7), 4.03 (t, *J* = 7.2 Hz, 1H, H_4), 3.63 – 3.55 (m, 2H, H_6), 2.34 – 2.29 (m, 2H, H_5).

^{13}C NMR (126 MHz, $CDCl_3$) δ 193.2 (C), 174.0 (C), 138.0 (C), 134.6 (2 × CH), 129.9 (CH), 129.4 (2 × CH), 128.6 (2 × CH), 127.94 (2 × CH), 127.89 (CH), 126.9 (C), 73.3 (CH_2), 67.0 (CH_2), 56.2 (CH), 29.7 (CH_2).

HRMS-CI (m/z): $[M + Na]^+$ calcd for $C_{18}H_{18}O_4SNa$, 353.0823; found, 353.0805.

R_f = 0.20 (50% ethyl acetate-hexane; UV).

2-((phenylthio)carbonyl)pent-4-enoic acid **S2e**

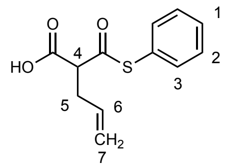

Following general method **E**, **S2e** was prepared from **S12e** (3.12 g, 16.9 mmol, 1 equiv), *N*,*N*-diisopropylethylamine (3.20 mL, 18.6 mmol, 1.10 equiv), trimethylsilyl chloride (2.40 mL, 18.6 mmol, 1.10 equiv), and thiophenol (1.80 mL, 17.8 mmol, 1.05 equiv). The residue was purified by column chromatography (eluting with 5% ethyl acetate–hexane initially, grading to 70% ethyl acetate–hexane). The product was obtained as a white solid (3.31 g, 82%).

^{1}H and ^{13}C NMR data for **S2e** prepared in this way were in agreement with the literature.(*62*)

^{1}H NMR (500 MHz, $CDCl_3$) δ 11.38 (bs, 1H, COOH), 7.48 – 7.39 (m, 5H, $H_{1\text{-}3}$), 5.82 (ddt, *J* = 17.0, 10.2, 6.8 Hz, 1H, H_6), 5.26 – 5.11 (m, 2H, H_7), 3.82 (t, *J* = 7.4 Hz, 1H, H_4), 2.79 – 2.72 (m, 2H, H_5).

^{13}C NMR (126 MHz, $CDCl_3$) δ 192.5 (C), 174.1 (C), 134.5 (2 × CH), 133.1 (CH), 123.0 (CH), 129.4 (2 × CH), 126.6 (C), 118.6 (CH_2), 58.8 (CH), 33.5 (CH_2).

HRMS-CI (m/z): $[M + Na]^+$ calcd for $C_{12}H_{12}O_3SNa$, 259.0405; found, 259.0401.

R_f = 0.25 (50% ethyl acetate-hexane; UV).

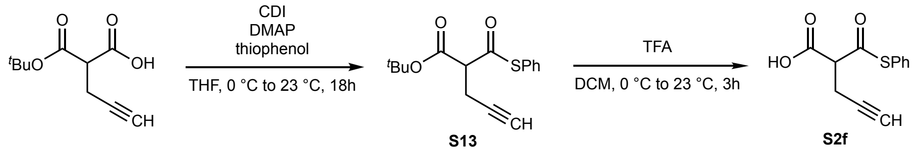

S13 **S2f**

tert-butyl 2-((phenylthio)carbonyl)pent-4-ynoate **S13**

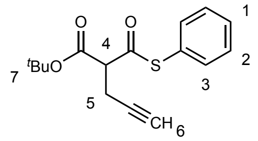

To a solution 2-(*tert*-butoxycarbonyl)pent-4-ynoic acid (2.54 g, 12.8 mmol, 1 equiv) in tetrahydrofuran (27.5 mL, 0.46 M) at 0°C was added 1,1′-carbonyldiimidazole (2.49 g, 15.4 mmol, 1.20 eq). The reaction mixture was stirred for 30 min at 0°C followed by 3 h at 23 °C. To the reaction solution was added 4-(dimethylamino)pyridine (0.47 g, 3.85 mmol, 0.30 equiv) and thiophenol (1.70 mL, 16.7 mmol, 1.3 eq) sequentially. The reaction was stirred at 23 °C for 19 h. The product mixture was concentrated. The residue was dissolved in ethyl acetate and washed three times with potassium carbonate solution (1.00 M) and twice with hydrogen chloride solution (1.00 M). The combined organic extracts were dried over sodium sulfate and

concentrated. The residue was purified by column chromatography (eluting with 5% ethyl acetate-hexane initially, grading to 40% ethyl acetate-hexane) to give **S13** as a colorless liquid (3.21 g, 86%).

^{1}H NMR (500 MHz, $CDCl_3$) δ 7.42 (s, 5H, $H_{1\text{-}3}$), 3.80 (t, J = 7.6 Hz, 1H, H_4), 2.78 – 2.81 (m, 2H, H_5), 2.05 (t, J = 2.7 Hz, 1H, H_6), 1.51 (s, 9H, H_7).

^{13}C NMR (126 MHz, $CDCl_3$) δ 192.2 (C), 166.1 (C), 134.6 (2 × CH), 129.9 (CH), 129.4 (2 × CH), 126.9 (C), 83.2 (C), 80.0 (C), 70.7 (CH), 59.3 (CH), 28.0 (3 × CH_3), 18.8 (CH_2).

HRMS-CI (m/z): $[M + Na]^+$ calcd for $C_{16}H_{18}O_3SNa$, 313.0874; found, 313.0869.

R_f = 0.62 (10% ethyl acetate-hexane; UV).

2-((phenylthio)carbonyl)pent-4-ynoic acid **S2f**

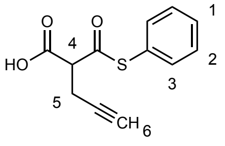

To a stirred solution of *tert*-butyl 2-((phenylthio)carbonyl)pent-4-ynoate **S13** (3.21 g, 11.0 mmol, 1 equiv) in dichloromethane (55.3 mL, 0.20 M) at 0 °C was added trifluoroacetic acid (36.8 mL, 481 mmol, 43.5 equiv). After being stirred at 0 °C for 1 h and at 23 °C for 2 h toluene was added to the reaction mixture and the mixture was concentrated. The crude compound was purified by column chromatography (eluting with 5% ethyl acetate-hexane initially, grading to 80% ethyl acetate-hexane) to give **S2f** as a brown liquid (2.10 g, 81%).

^{1}H NMR (500 MHz, $CDCl_3$) δ 7.47 – 7.41 (m, 5H, $H_{1\text{-}3}$), 3.95 (t, J = 7.5 Hz, 1H, H_4), 2.87 (dd, J = 7.6, 2.7 Hz, 2H, H_5), 2.11 (t, J = 2.7 Hz, 1H, H_6).

^{13}C NMR (126 MHz, $CDCl_3$) δ 191.6 (C), 172.1 (C), 134.6 (2 × CH), 130.2 (CH), 129.6 (2 × CH), 126.4 (C), 79.3 (C), 71.4 (CH), 57.8 (CH), 19.0 (CH_2).

HRMS-CI (m/z): $[M + Na]^+$ calcd for $C_{12}H_{10}O_3SNa$, 257.0248; found, 257.0243.

R_f = 0.32 (40% ethyl acetate-hexane; UV).

5-methoxy-5-oxo-2-((phenylthio)carbonyl)pentanoic acid **S2g**

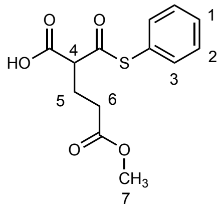

Following general method **F**, **S2g** was prepared from methyl 3-(2,2-dimethyl-4,6-dioxo-1,3-dioxan-5-yl)propanoate **S12g** (5.0 g, 21.7 mmol, 1 equiv), *N*,*N*-diisopropylethylamine (4.20 mL, 23.9 mmol, 1.10 equiv), trimethylsilyl chloride (2.40 mL, 23.9 mmol, 1.10 equiv), and thiophenol (2.30 mL, 22.8 mmol, 1.05 equiv). The residue was purified by column chromatography (eluting with 5% ethyl acetate–hexane initially, grading to 100% ethyl acetate–hexane). The product was obtained as a white solid (4.22 g, 68%).

^{1}H NMR (500 MHz, $CDCl_3$) δ 7.47 – 7.39 (m, 5H, $H_{1\text{-}3}$), 3.87 (t, J = 7.3 Hz, 1H, H_4), 3.70 (s, 3H, H_7), 2.49 (t, J = 7.1 Hz, 2H, H_6), 2.35 – 2.27 (m, 2H, H_5).

^{13}C NMR (126 MHz, $CDCl_3$) δ 192.9 (C), 173.3 (C), 173.0 (C), 134.6 (2 × CH), 130.0 (CH), 129.5 (2 × CH), 126.6 (C), 57.8 (CH), 52.0 (CH_3), 31.1 (CH_2), 24.5 (CH_2).

HRMS-CI (m/z): $[M + H]^+$ calcd for $C_{13}H_{15}O_5S$, 283.0640; found, 283.0632.

R_f = 0.20 (50% ethyl acetate-hexane; UV).

Catalog of HPLC spectra:

HPLC (AD-H, *n*-hexane/^{i}PrOH=90:10, 1.0 mL/min, 298 K, 254 nm) for (*S*,*S*)-**5a** and (*S*,*R*)-**6a**:

Racemic aldol product:

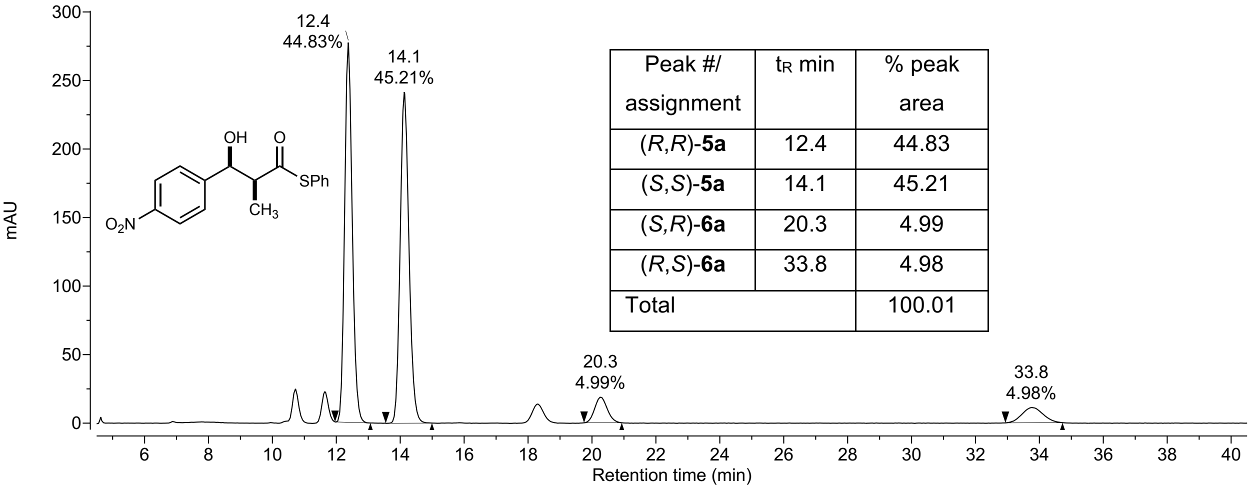

Peak #/ assignment	t_R min	% peak area
(*R*,*R*)-**5a**	12.4	44.83
(*S*,*S*)-**5a**	14.1	45.21
(*S*,*R*)-**6a**	20.3	4.99
(*R*,*S*)-**6a**	33.8	4.98
Total		100.01

Syn-aldol product:

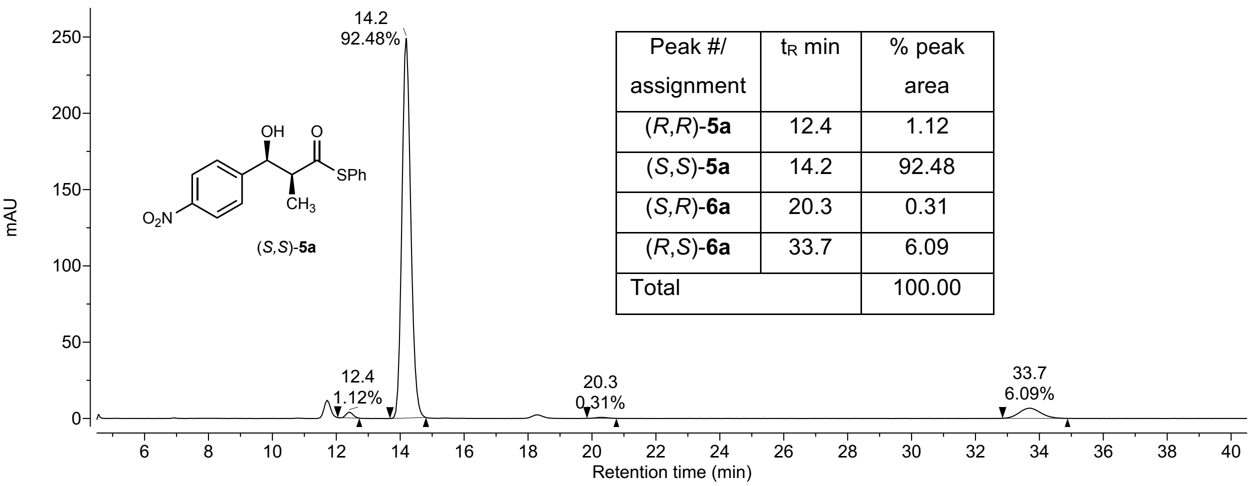

Peak #/ assignment	t_R min	% peak area
(*R*,*R*)-**5a**	12.4	1.12
(*S*,*S*)-**5a**	14.2	92.48
(*S*,*R*)-**6a**	20.3	0.31
(*R*,*S*)-**6a**	33.7	6.09
Total		100.00

Anti-aldol product:

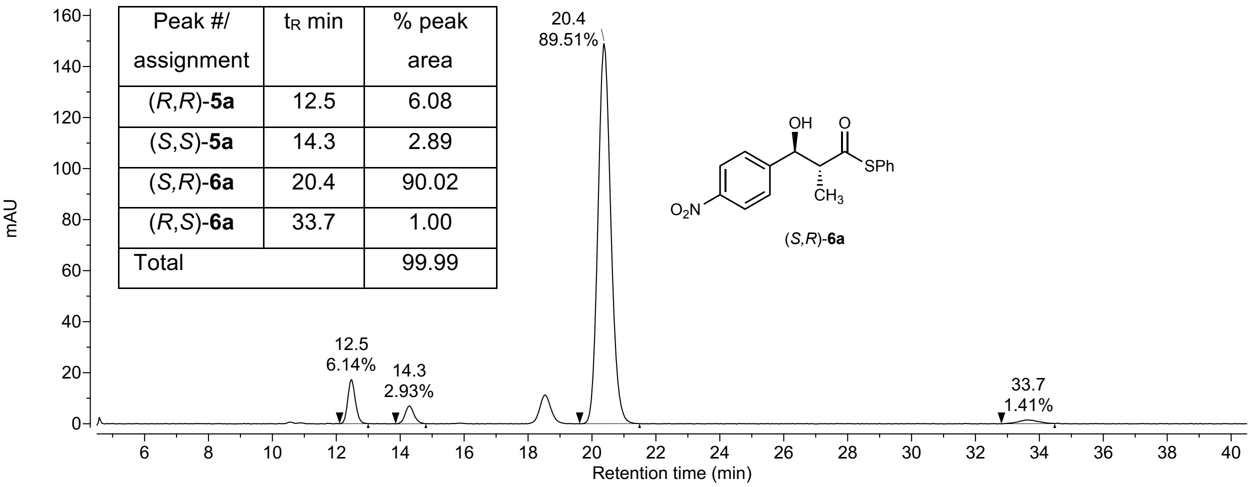

Peak #/ assignment	t_R min	% peak area
(*R*,*R*)-**5a**	12.5	6.08
(*S*,*S*)-**5a**	14.3	2.89
(*S*,*R*)-**6a**	20.4	90.02
(*R*,*S*)-**6a**	33.7	1.00
Total		99.99

HPLC (AD-H, *n*-hexane/^{i}PrOH=90:10, 1.0 mL/min, 298 K, 254 nm) for (*R*,*R*)-**5a** and (*R*,*S*)-**6a**:

Racemic aldol product:

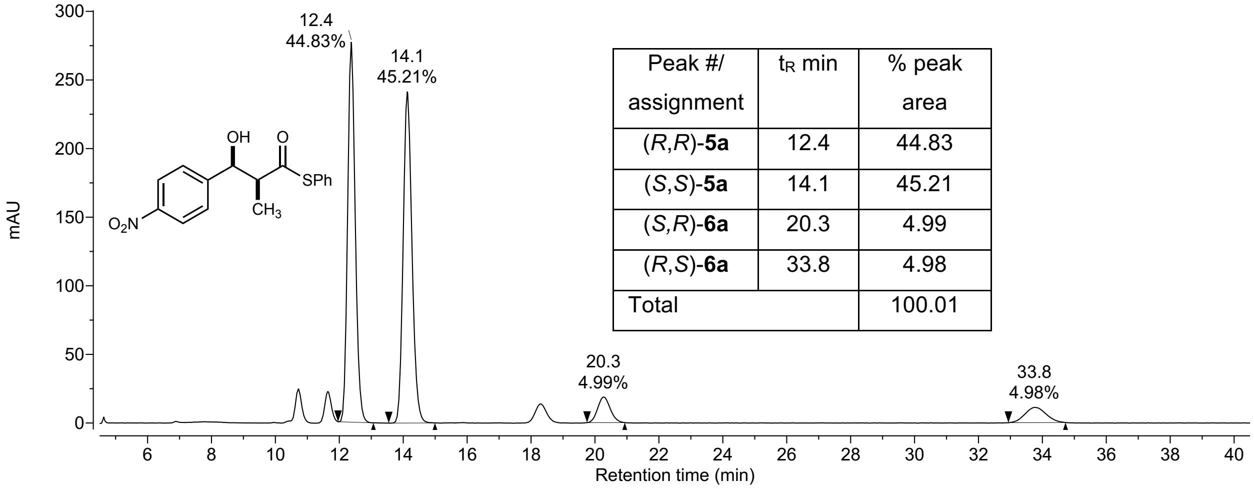

Peak #/ assignment	t_R min	% peak area
(*R*,*R*)-**5a**	12.4	44.83
(*S*,*S*)-**5a**	14.1	45.21
(*S*,*R*)-**6a**	20.3	4.99
(*R*,*S*)-**6a**	33.8	4.98
Total		100.01

Syn-aldol product:

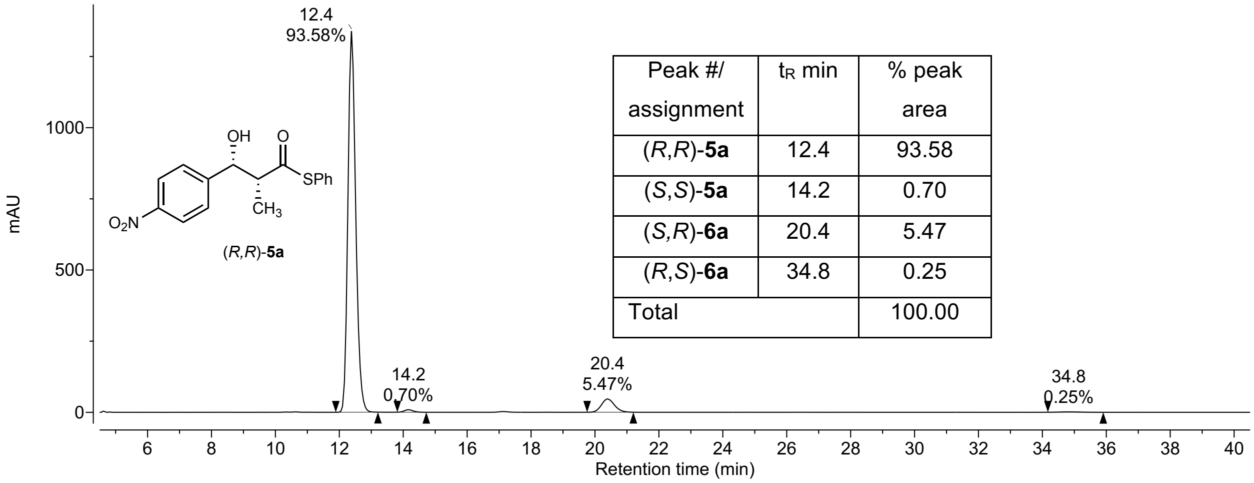

Peak #/ assignment	t_R min	% peak area
(*R*,*R*)-**5a**	12.4	93.58
(*S*,*S*)-**5a**	14.2	0.70
(*S*,*R*)-**6a**	20.4	5.47
(*R*,*S*)-**6a**	34.8	0.25
Total		100.00

Anti-aldol product:

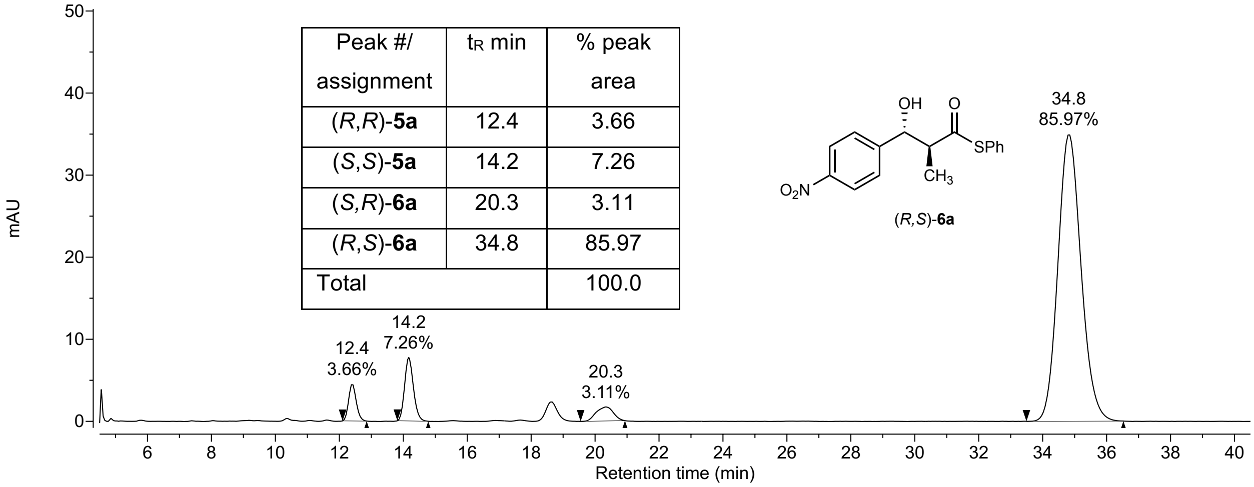

Peak #/ assignment	t_R min	% peak area
(*R*,*R*)-**5a**	12.4	3.66
(*S*,*S*)-**5a**	14.2	7.26
(*S*,*R*)-**6a**	20.3	3.11
(*R*,*S*)-**6a**	34.8	85.97
Total		100.0

HPLC (AD-H, *n*-hexane/^{i}PrOH=90:10, 1.0 mL/min, 298 K, 254 nm) for **5b** and **6b**:

Racemic aldol product:

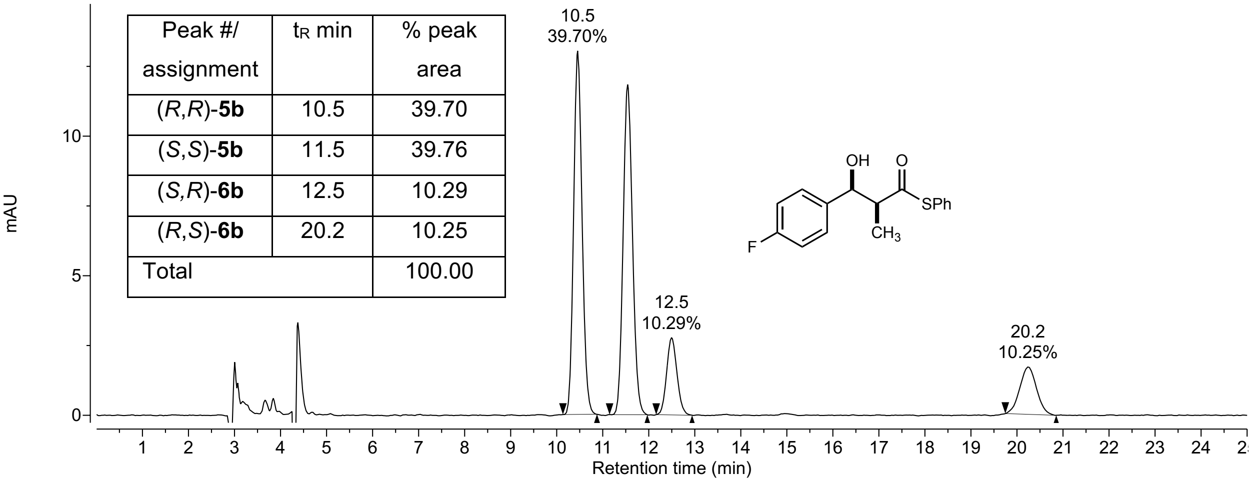

Peak #/ assignment	t_R min	% peak area
(*R*,*R*)-**5b**	10.5	39.70
(*S*,*S*)-**5b**	11.5	39.76
(*S*,*R*)-**6b**	12.5	10.29
(*R*,*S*)-**6b**	20.2	10.25
Total		100.00

Syn-aldol product:

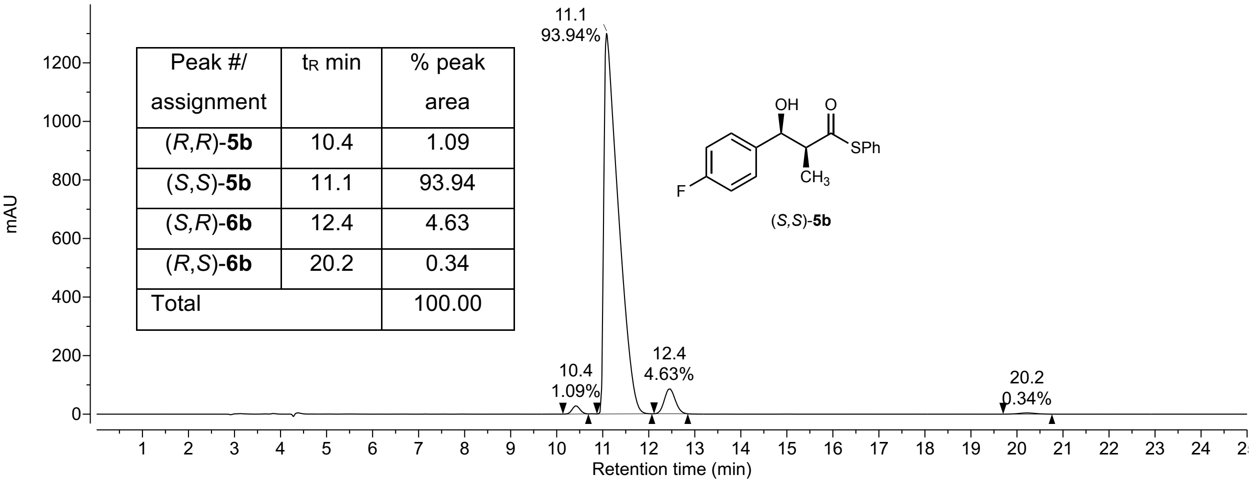

Peak #/ assignment	t_R min	% peak area
(*R*,*R*)-**5b**	10.4	1.09
(*S*,*S*)-**5b**	11.1	93.94
(*S*,*R*)-**6b**	12.4	4.63
(*R*,*S*)-**6b**	20.2	0.34
Total		100.00

Anti-aldol product:

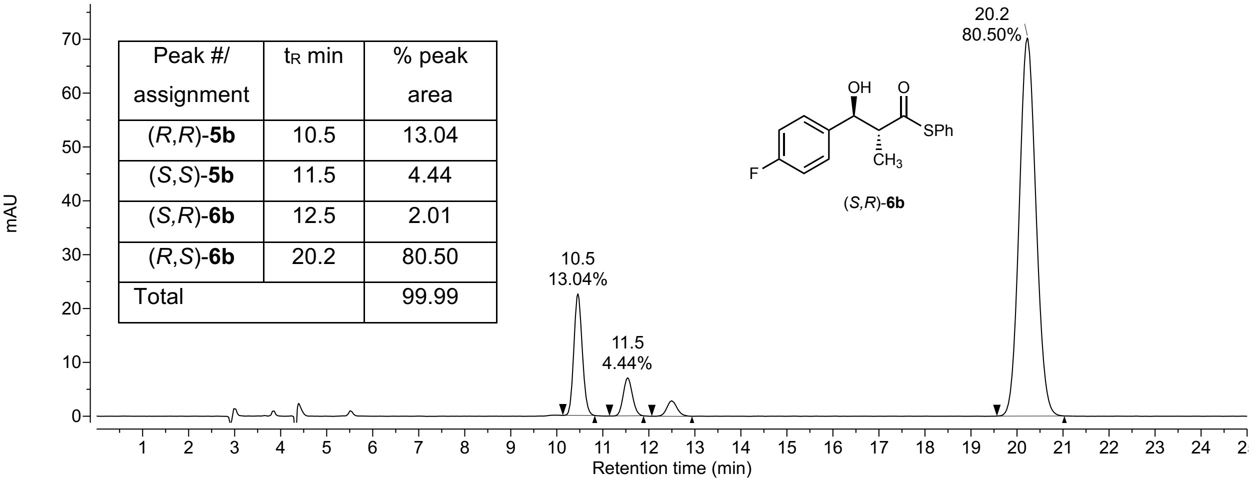

Peak #/ assignment	t_R min	% peak area
(*R*,*R*)-**5b**	10.5	13.04
(*S*,*S*)-**5b**	11.5	4.44
(*S*,*R*)-**6b**	12.5	2.01
(*R*,*S*)-**6b**	20.2	80.50
Total		99.99

HPLC (AD-H, *n*-hexane/^{i}PrOH=90:10, 1.0 mL/min, 298 K, 254 nm) for **5c** and **6c**:

Racemic aldol product:

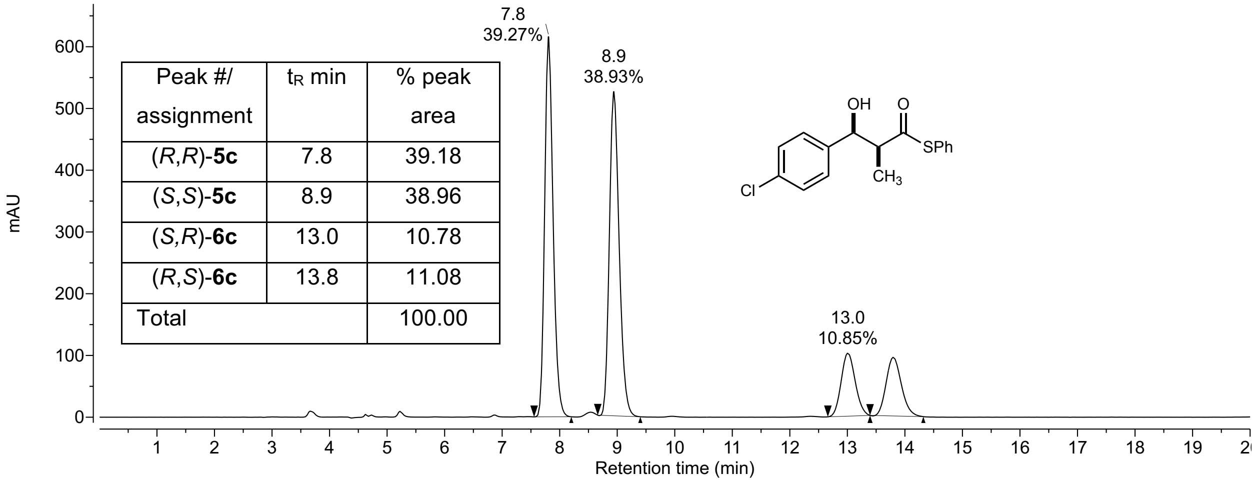

Peak #/ assignment	t_R min	% peak area
(*R*,*R*)-**5c**	7.8	39.18
(*S*,*S*)-**5c**	8.9	38.96
(*S*,*R*)-**6c**	13.0	10.78
(*R*,*S*)-**6c**	13.8	11.08
Total		100.00

Syn-aldol product:

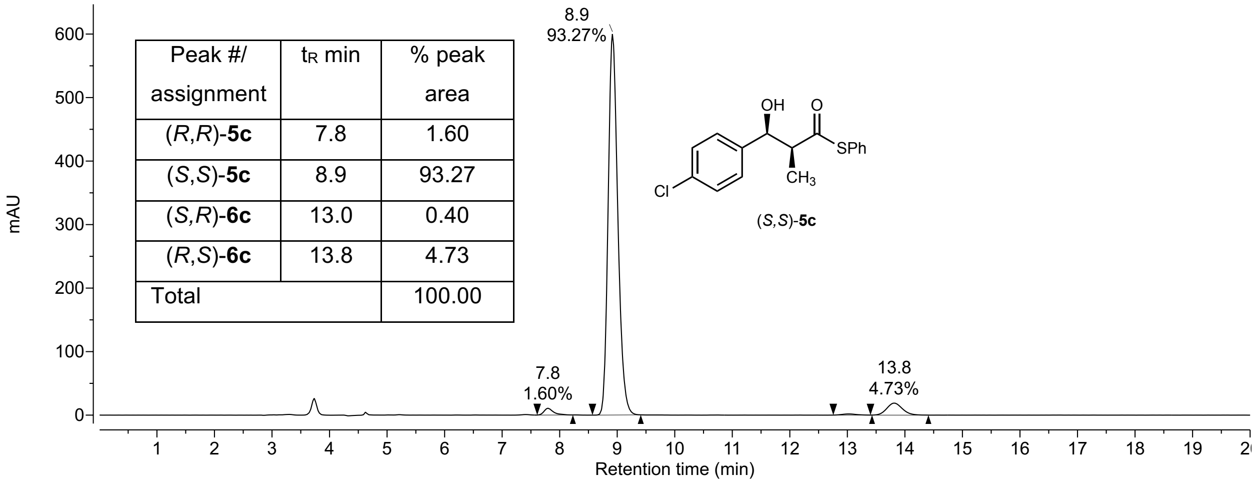

Peak #/ assignment	t_R min	% peak area
(*R*,*R*)-**5c**	7.8	1.60
(*S*,*S*)-**5c**	8.9	93.27
(*S*,*R*)-**6c**	13.0	0.40
(*R*,*S*)-**6c**	13.8	4.73
Total		100.00

Anti-aldol product:

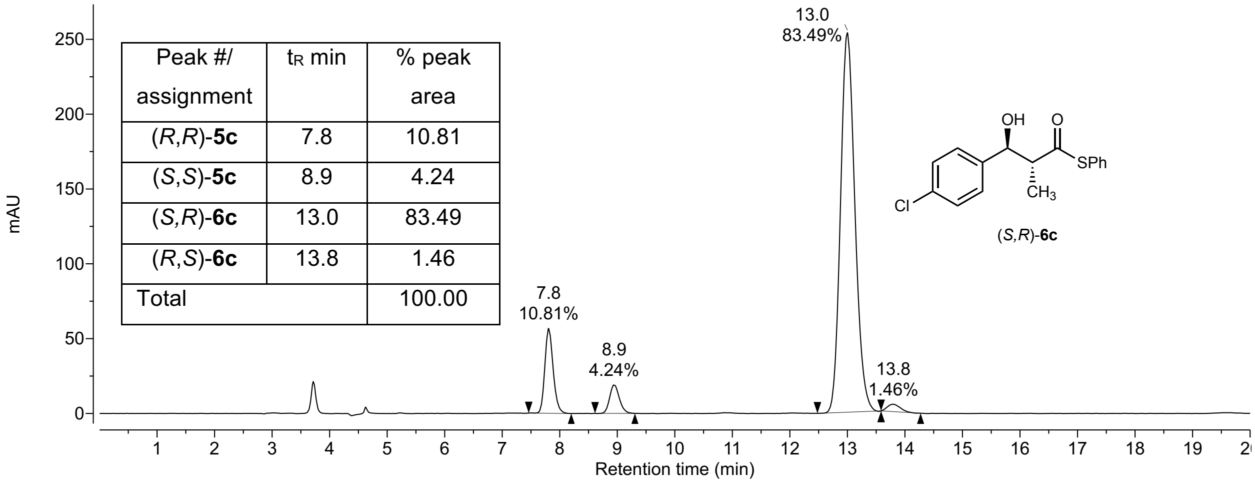

Peak #/ assignment	t_R min	% peak area
(*R*,*R*)-**5c**	7.8	10.81
(*S*,*S*)-**5c**	8.9	4.24
(*S*,*R*)-**6c**	13.0	83.49
(*R*,*S*)-**6c**	13.8	1.46
Total		100.00

HPLC (AD-H, n-hexane/^{i}PrOH=90:10, 1.0 mL/min, 298 K, 254 nm) for **5d** and **6d**:

Racemic aldol product:

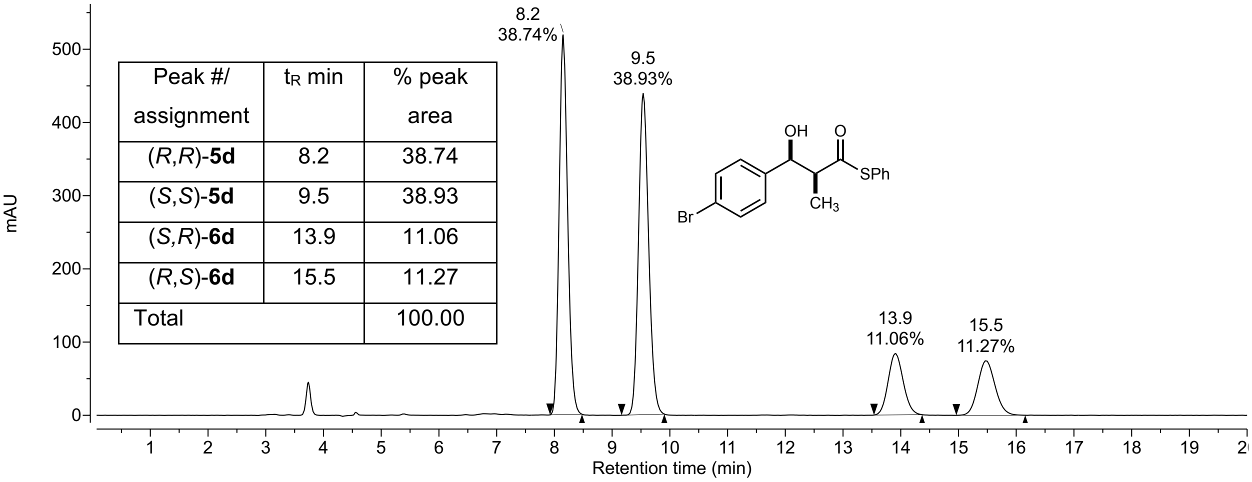

Peak #/ assignment	t_R min	% peak area
(R,R)-**5d**	8.2	38.74
(S,S)-**5d**	9.5	38.93
(S,R)-**6d**	13.9	11.06
(R,S)-**6d**	15.5	11.27
Total		100.00

Syn-aldol product:

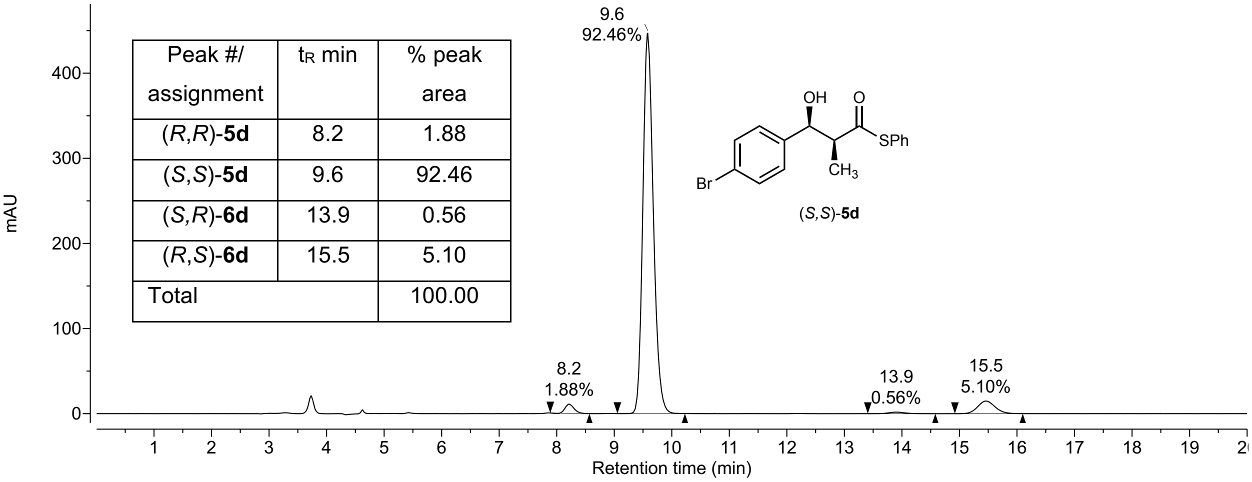

Peak #/ assignment	t_R min	% peak area
(R,R)-**5d**	8.2	1.88
(S,S)-**5d**	9.6	92.46
(S,R)-**6d**	13.9	0.56
(R,S)-**6d**	15.5	5.10
Total		100.00

Anti-aldol product:

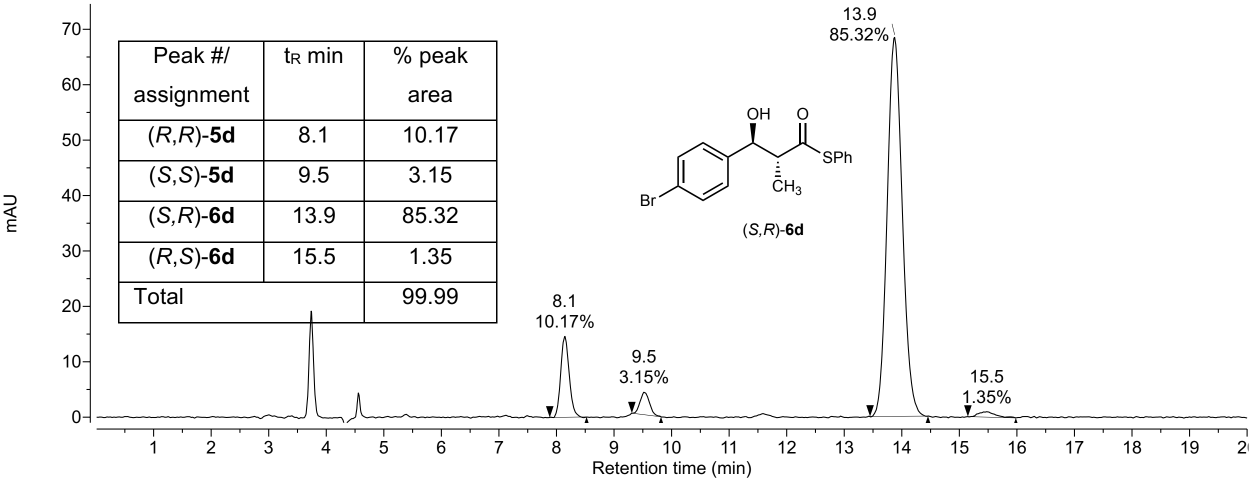

Peak #/ assignment	t_R min	% peak area
(R,R)-**5d**	8.1	10.17
(S,S)-**5d**	9.5	3.15
(S,R)-**6d**	13.9	85.32
(R,S)-**6d**	15.5	1.35
Total		99.99

HPLC (AD-H, *n*-hexane/^{i}PrOH=90:10, 1.0 mL/min, 298 K, 254 nm) for **5e** and **6e**:

Racemic aldol product:

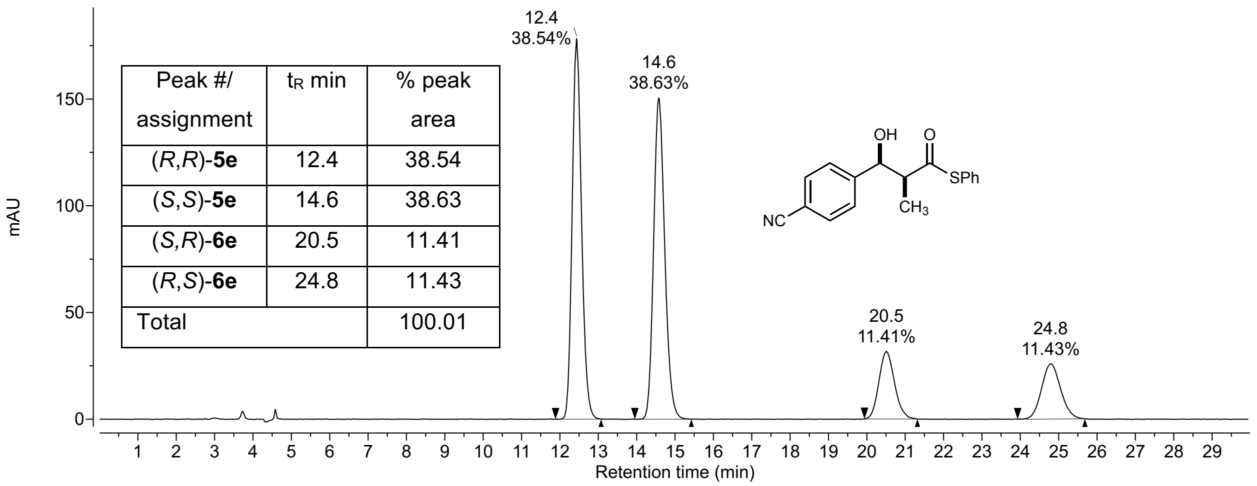

Peak #/ assignment	t_R min	% peak area
(*R*,*R*)-**5e**	12.4	38.54
(*S*,*S*)-**5e**	14.6	38.63
(*S*,*R*)-**6e**	20.5	11.41
(*R*,*S*)-**6e**	24.8	11.43
Total		100.01

Syn-aldol product:

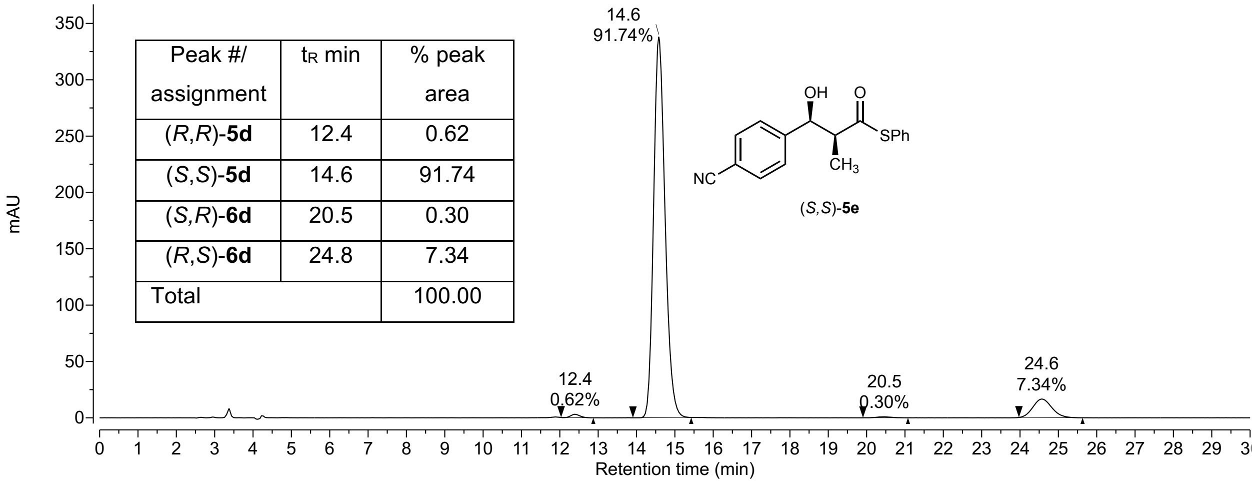

Peak #/ assignment	t_R min	% peak area
(*R*,*R*)-**5d**	12.4	0.62
(*S*,*S*)-**5d**	14.6	91.74
(*S*,*R*)-**6d**	20.5	0.30
(*R*,*S*)-**6d**	24.8	7.34
Total		100.00

Anti-aldol product:

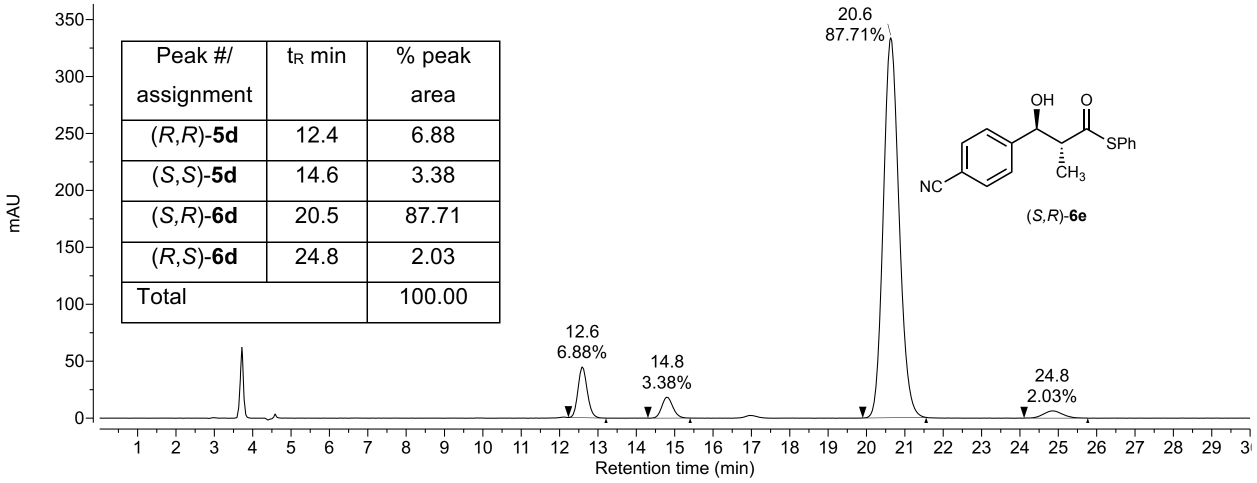

Peak #/ assignment	t_R min	% peak area
(*R*,*R*)-**5d**	12.4	6.88
(*S*,*S*)-**5d**	14.6	3.38
(*S*,*R*)-**6d**	20.5	87.71
(*R*,*S*)-**6d**	24.8	2.03
Total		100.00

HPLC (AD-H, *n*-hexane/^{i}PrOH=90:10, 1.0 mL/min, 298 K, 254 nm) for **5f** and **6f**:

Racemic aldol product:

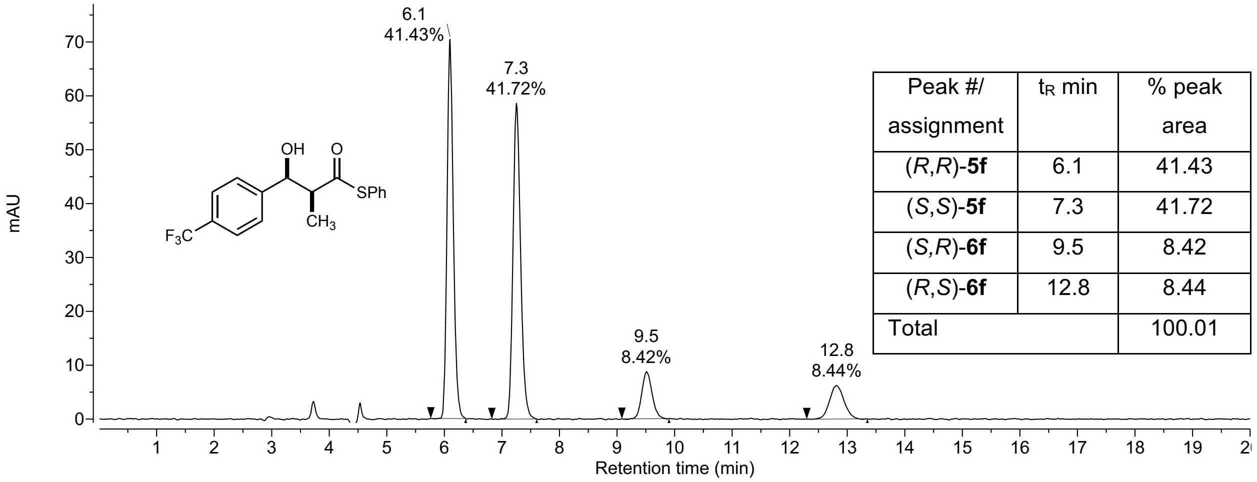

Peak #/ assignment	t_R min	% peak area
(*R*,*R*)-**5f**	6.1	41.43
(*S*,*S*)-**5f**	7.3	41.72
(*S*,*R*)-**6f**	9.5	8.42
(*R*,*S*)-**6f**	12.8	8.44
Total		100.01

Syn-aldol product:

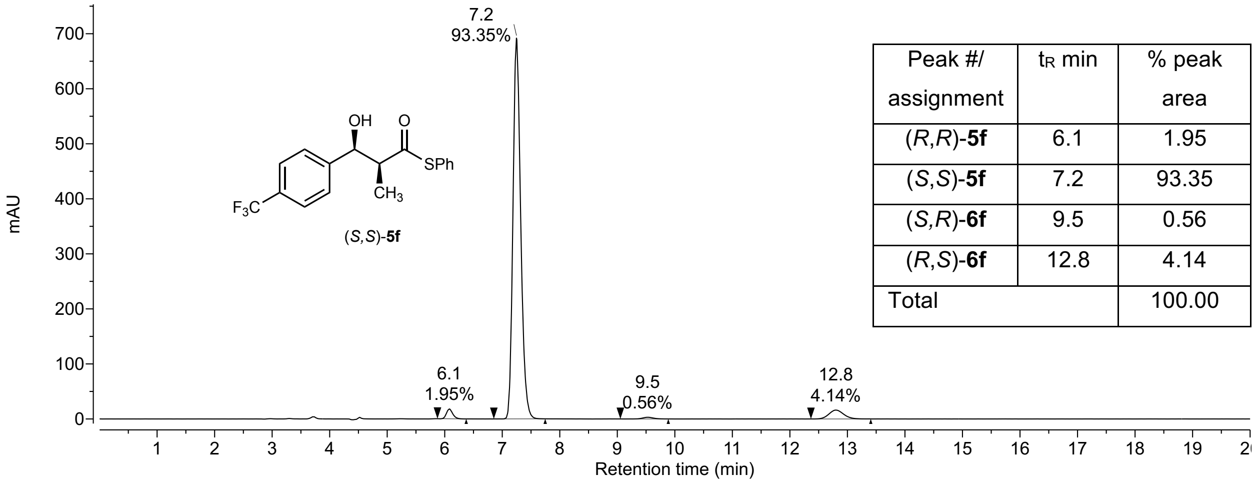

Peak #/ assignment	t_R min	% peak area
(*R*,*R*)-**5f**	6.1	1.95
(*S*,*S*)-**5f**	7.2	93.35
(*S*,*R*)-**6f**	9.5	0.56
(*R*,*S*)-**6f**	12.8	4.14
Total		100.00

Anti-aldol product:

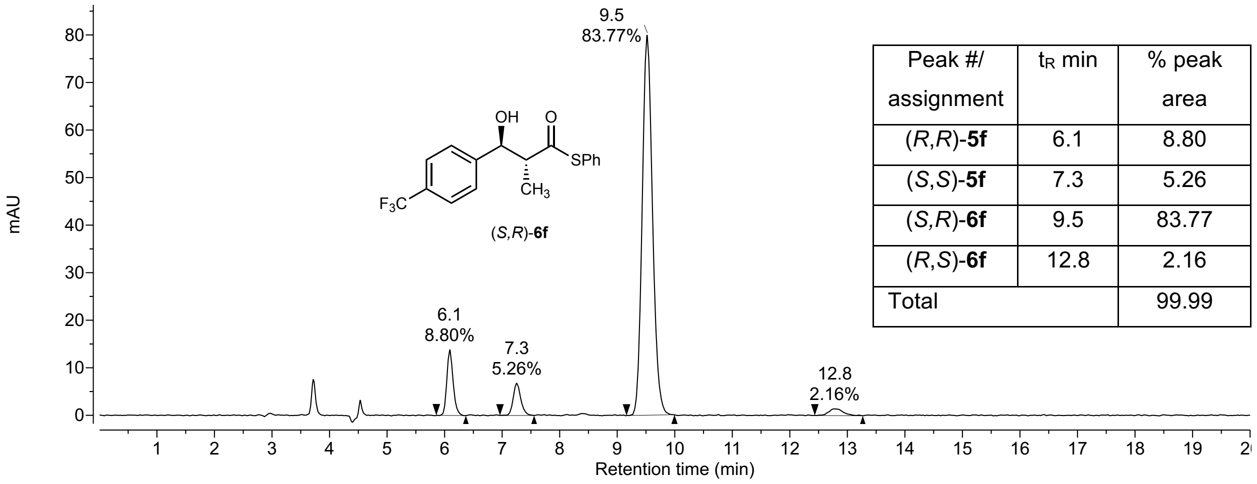

Peak #/ assignment	t_R min	% peak area
(*R*,*R*)-**5f**	6.1	8.80
(*S*,*S*)-**5f**	7.3	5.26
(*S*,*R*)-**6f**	9.5	83.77
(*R*,*S*)-**6f**	12.8	2.16
Total		99.99

HPLC (AD-H, *n*-hexane/EtOH=85:15, 1.0 mL/min, 298 K, 254 nm) for **5g** and **6g**:

Racemic aldol product:

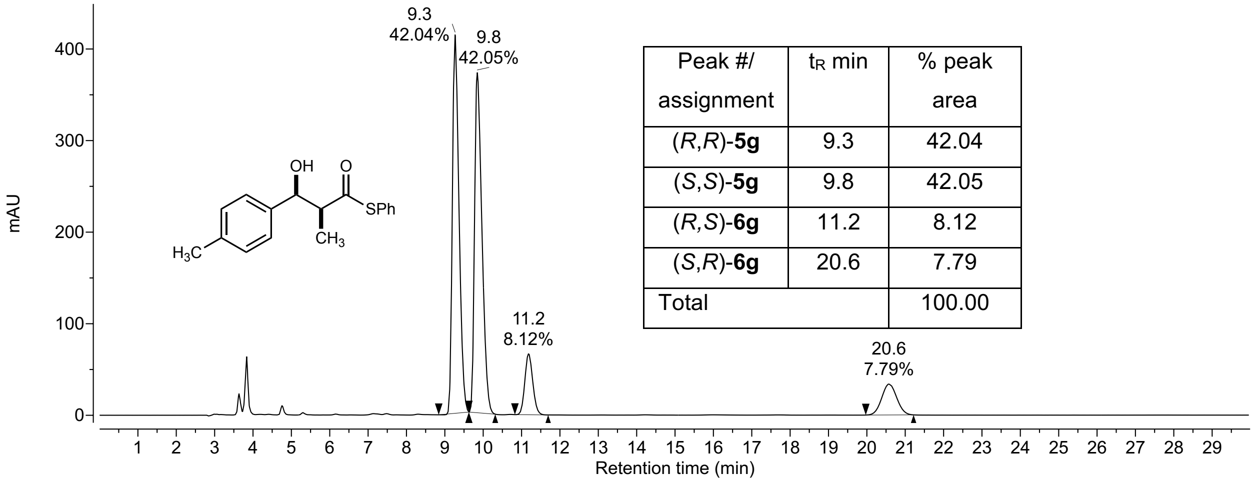

Peak #/ assignment	t_R min	% peak area
(*R*,*R*)-**5g**	9.3	42.04
(*S*,*S*)-**5g**	9.8	42.05
(*R*,*S*)-**6g**	11.2	8.12
(*S*,*R*)-**6g**	20.6	7.79
Total		100.00

Syn-aldol product:

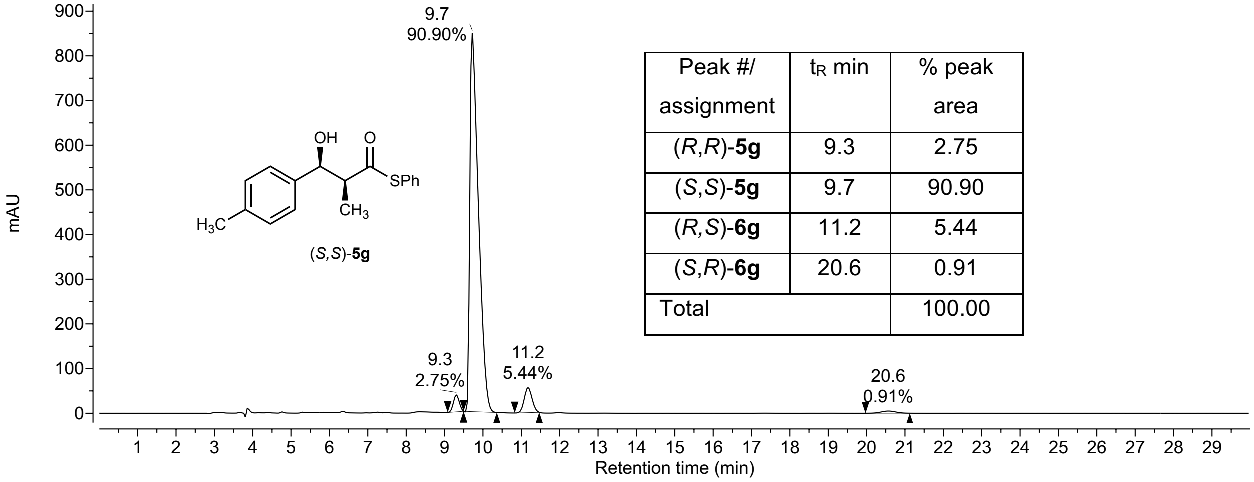

Peak #/ assignment	t_R min	% peak area
(*R*,*R*)-**5g**	9.3	2.75
(*S*,*S*)-**5g**	9.7	90.90
(*R*,*S*)-**6g**	11.2	5.44
(*S*,*R*)-**6g**	20.6	0.91
Total		100.00

Anti-aldol product:

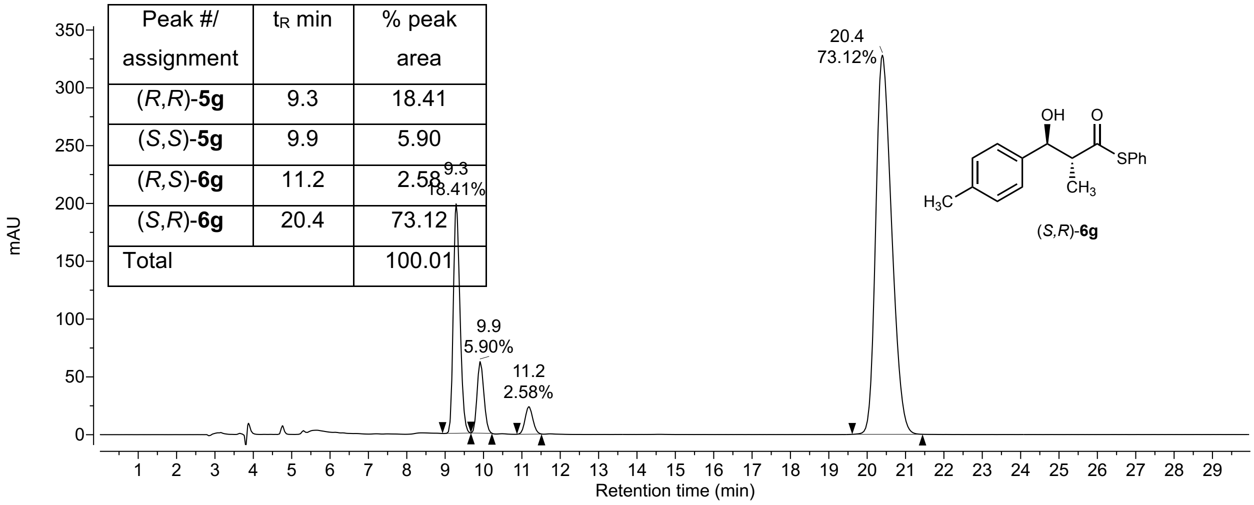

Peak #/ assignment	t_R min	% peak area
(*R*,*R*)-**5g**	9.3	18.41
(*S*,*S*)-**5g**	9.9	5.90
(*R*,*S*)-**6g**	11.2	2.58
(*S*,*R*)-**6g**	20.4	73.12
Total		100.01

HPLC (AD-H, *n*-hexane/^{i}PrOH=90:10, 1.0 mL/min, 298 K, 254 nm) for **5h** and **6h**:

Racemic aldol product:

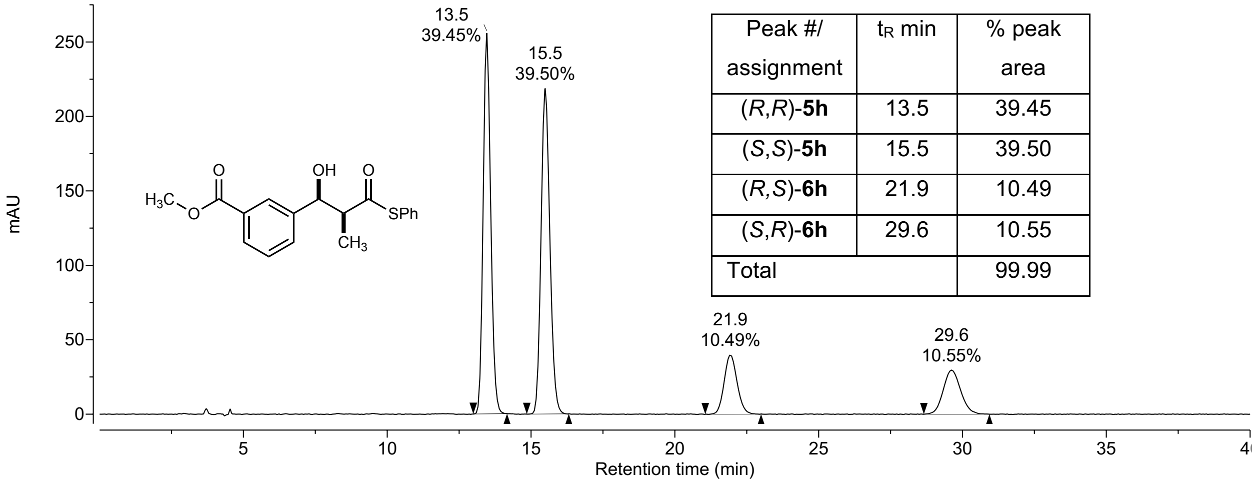

Peak #/ assignment	t_R min	% peak area
(*R*,*R*)-**5h**	13.5	39.45
(*S*,*S*)-**5h**	15.5	39.50
(*R*,*S*)-**6h**	21.9	10.49
(*S*,*R*)-**6h**	29.6	10.55
Total		99.99

Syn-aldol product:

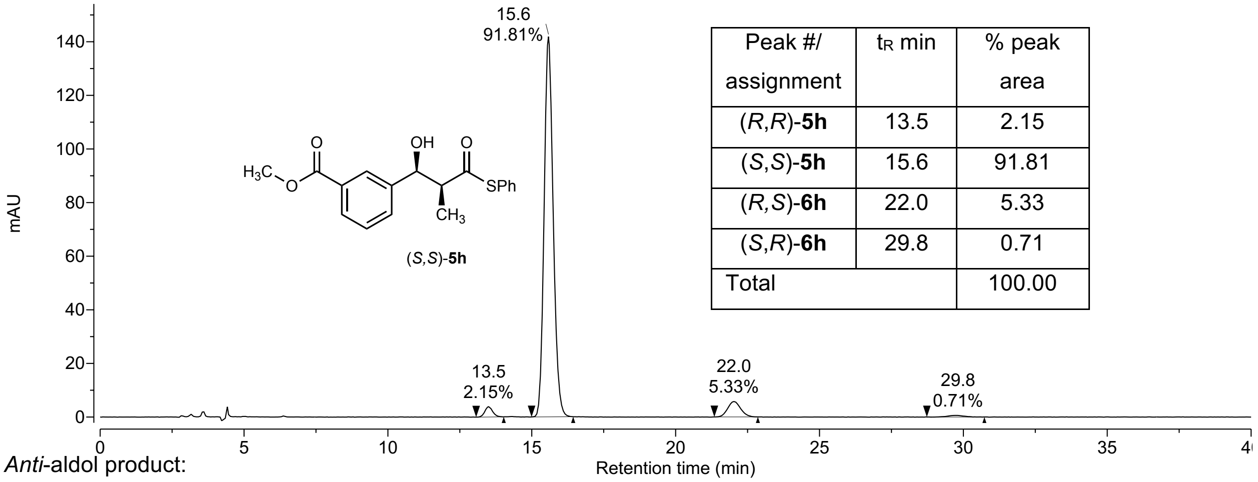

Peak #/ assignment	t_R min	% peak area
(*R*,*R*)-**5h**	13.5	2.15
(*S*,*S*)-**5h**	15.6	91.81
(*R*,*S*)-**6h**	22.0	5.33
(*S*,*R*)-**6h**	29.8	0.71
Total		100.00

Anti-aldol product:

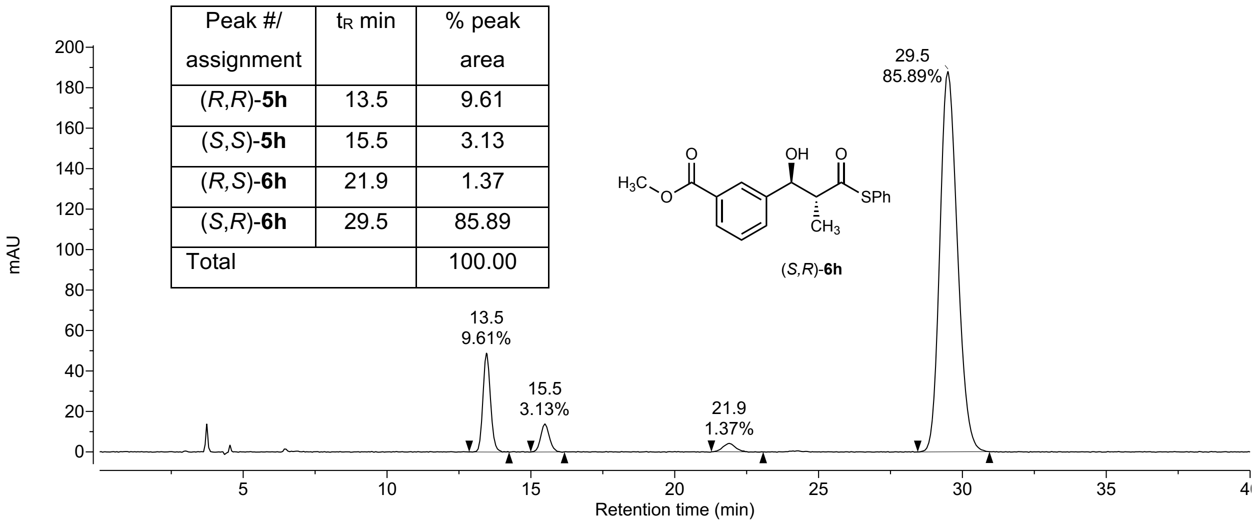

Peak #/ assignment	t_R min	% peak area
(*R*,*R*)-**5h**	13.5	9.61
(*S*,*S*)-**5h**	15.5	3.13
(*R*,*S*)-**6h**	21.9	1.37
(*S*,*R*)-**6h**	29.5	85.89
Total		100.00

HPLC (OD-H, *n*-hexane/^{i}PrOH=88:12, 1.0 mL/min, 298 K, 254 nm) for **5i** and **6i**:

Racemic aldol product:

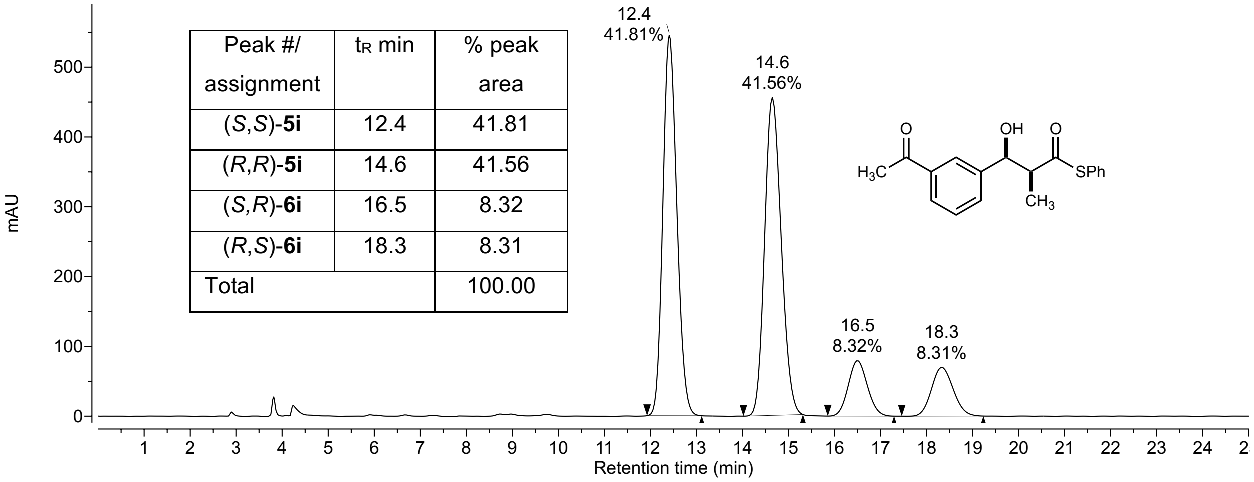

Peak #/ assignment	t_R min	% peak area
(*S*,*S*)-**5i**	12.4	41.81
(*R*,*R*)-**5i**	14.6	41.56
(*S*,*R*)-**6i**	16.5	8.32
(*R*,*S*)-**6i**	18.3	8.31
Total		100.00

Syn-aldol product:

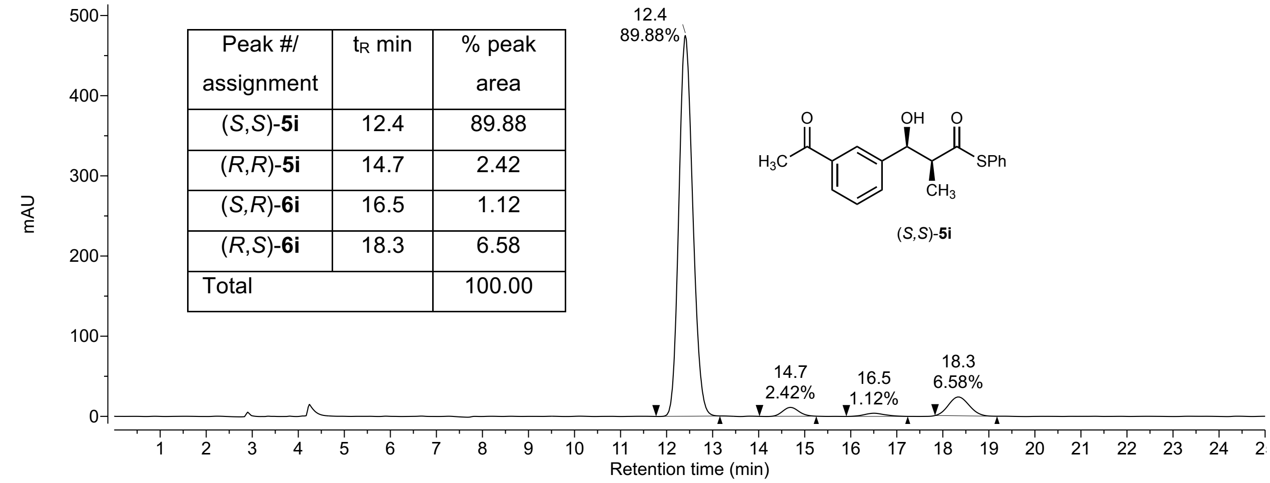

Peak #/ assignment	t_R min	% peak area
(*S*,*S*)-**5i**	12.4	89.88
(*R*,*R*)-**5i**	14.7	2.42
(*S*,*R*)-**6i**	16.5	1.12
(*R*,*S*)-**6i**	18.3	6.58
Total		100.00

Anti-aldol product:

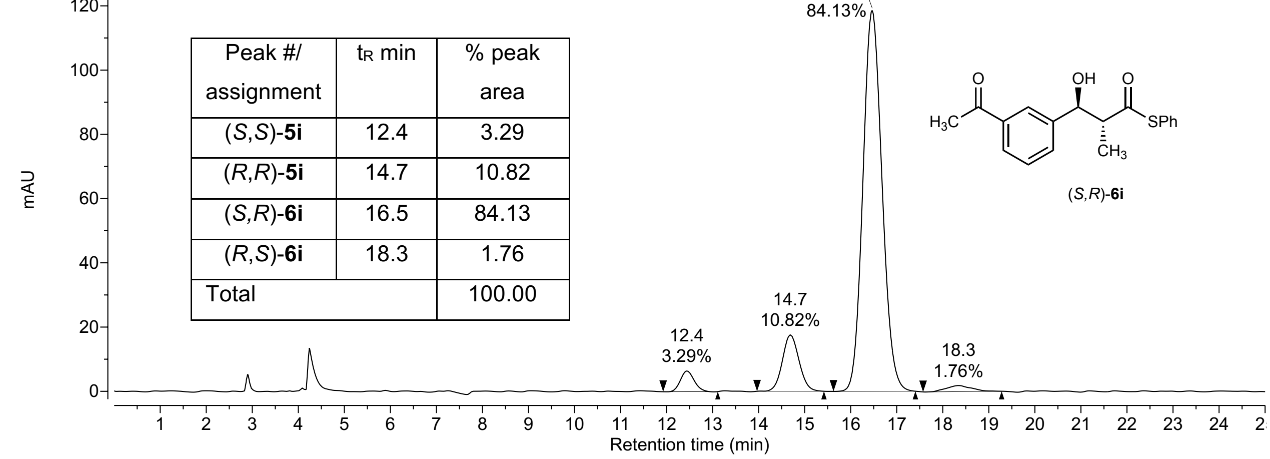

Peak #/ assignment	t_R min	% peak area
(*S*,*S*)-**5i**	12.4	3.29
(*R*,*R*)-**5i**	14.7	10.82
(*S*,*R*)-**6i**	16.5	84.13
(*R*,*S*)-**6i**	18.3	1.76
Total		100.00

HPLC (AD-H, *n*-hexane/^{i}PrOH=90:10, 1.0 mL/min, 298 K, 254 nm) for **5j** and **6j**:

Racemic aldol product:

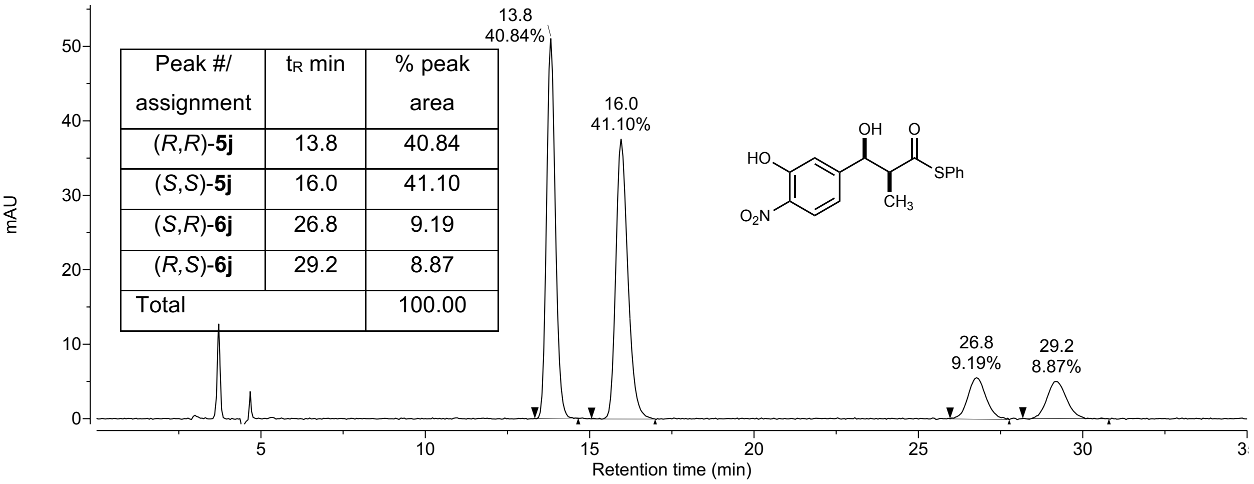

Peak #/ assignment	t_R min	% peak area
(*R*,*R*)-**5j**	13.8	40.84
(*S*,*S*)-**5j**	16.0	41.10
(*S*,*R*)-**6j**	26.8	9.19
(*R*,*S*)-**6j**	29.2	8.87
Total		100.00

Syn-aldol product:

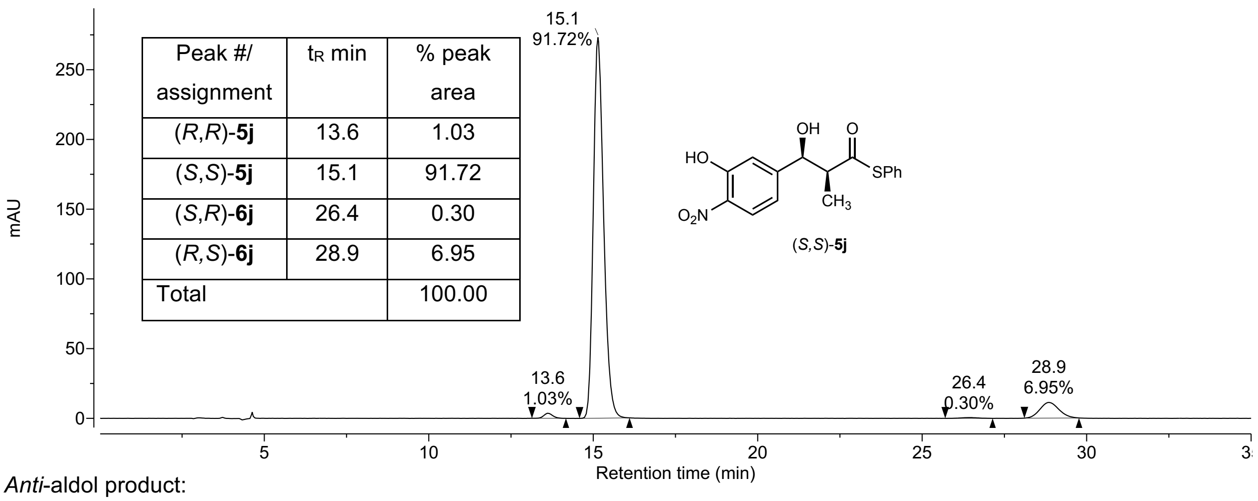

Peak #/ assignment	t_R min	% peak area
(*R*,*R*)-**5j**	13.6	1.03
(*S*,*S*)-**5j**	15.1	91.72
(*S*,*R*)-**6j**	26.4	0.30
(*R*,*S*)-**6j**	28.9	6.95
Total		100.00

Anti-aldol product:

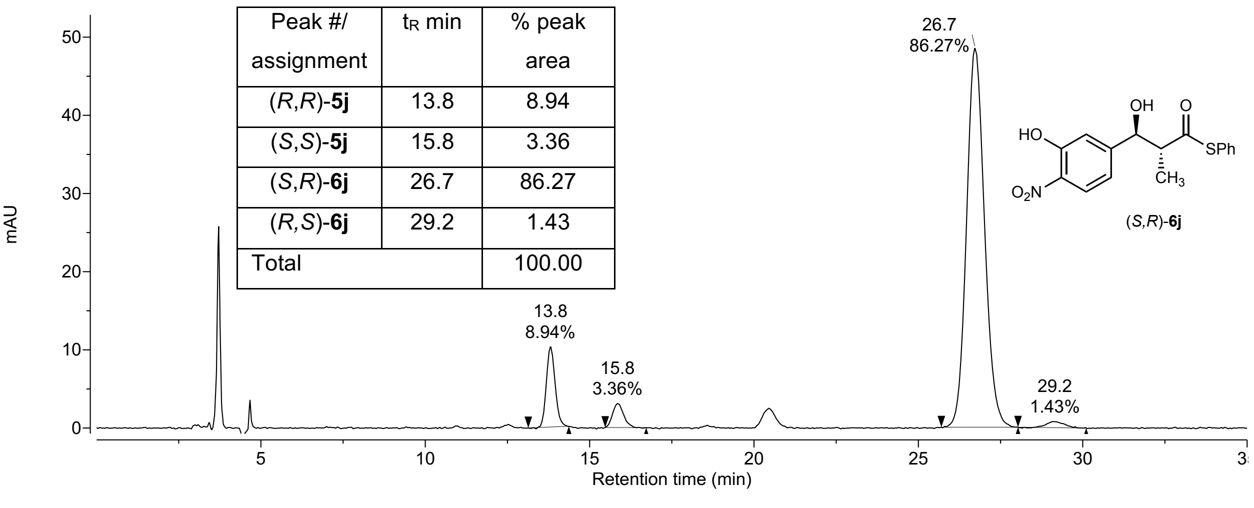

Peak #/ assignment	t_R min	% peak area
(*R*,*R*)-**5j**	13.8	8.94
(*S*,*S*)-**5j**	15.8	3.36
(*S*,*R*)-**6j**	26.7	86.27
(*R*,*S*)-**6j**	29.2	1.43
Total		100.00

HPLC (AD-H, *n*-hexane/^{i}PrOH=90:10, 1.0 mL/min, 298 K, 254 nm) for **5k** and **6k**:

Racemic aldol product:

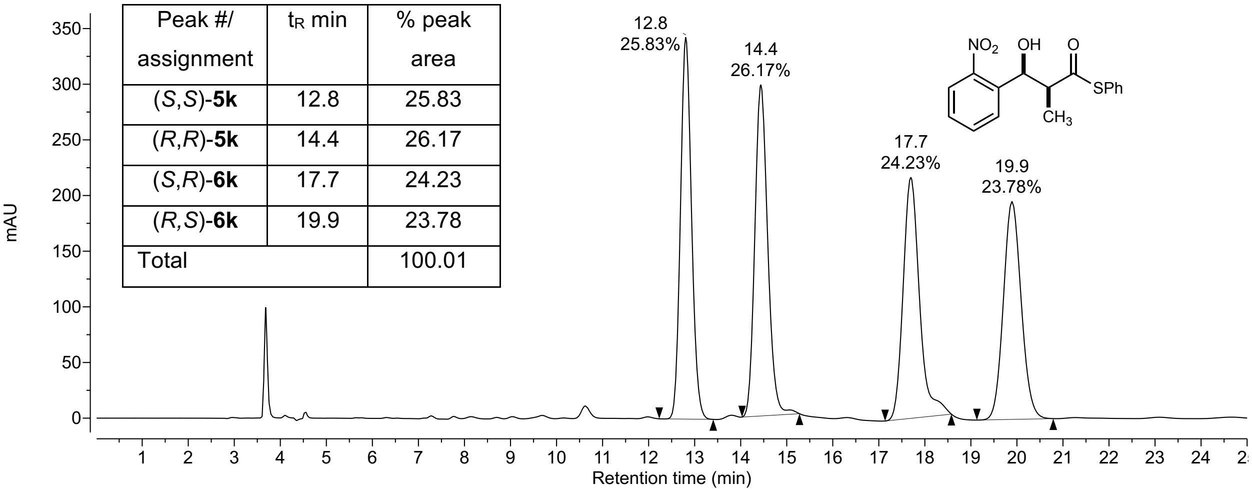

Peak #/ assignment	t_R min	% peak area
(*S*,*S*)-**5k**	12.8	25.83
(*R*,*R*)-**5k**	14.4	26.17
(*S*,*R*)-**6k**	17.7	24.23
(*R*,*S*)-**6k**	19.9	23.78
Total		100.01

Syn-aldol product:

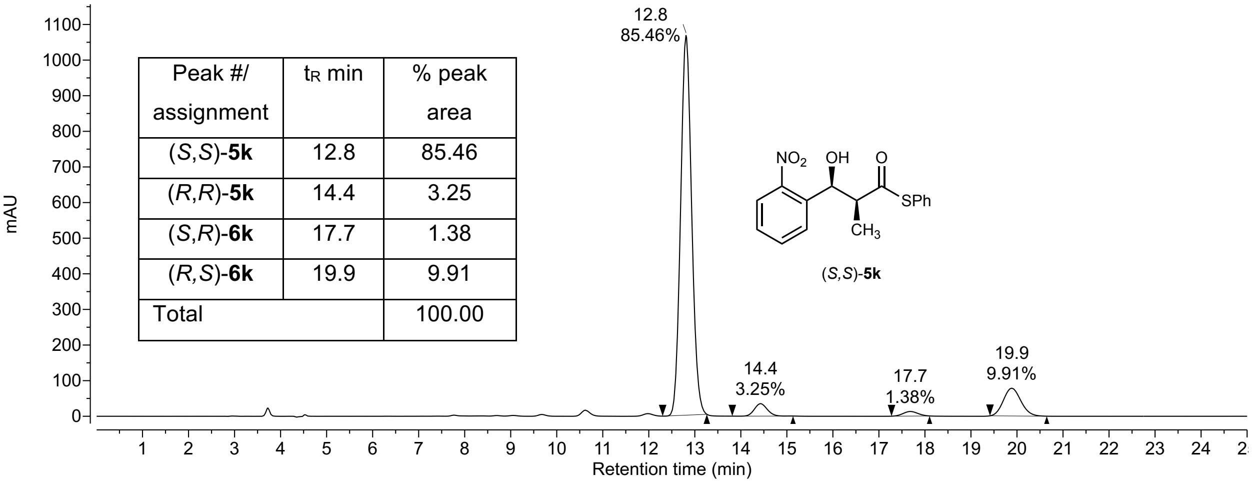

Peak #/ assignment	t_R min	% peak area
(*S*,*S*)-**5k**	12.8	85.46
(*R*,*R*)-**5k**	14.4	3.25
(*S*,*R*)-**6k**	17.7	1.38
(*R*,*S*)-**6k**	19.9	9.91
Total		100.00

Anti-aldol product:

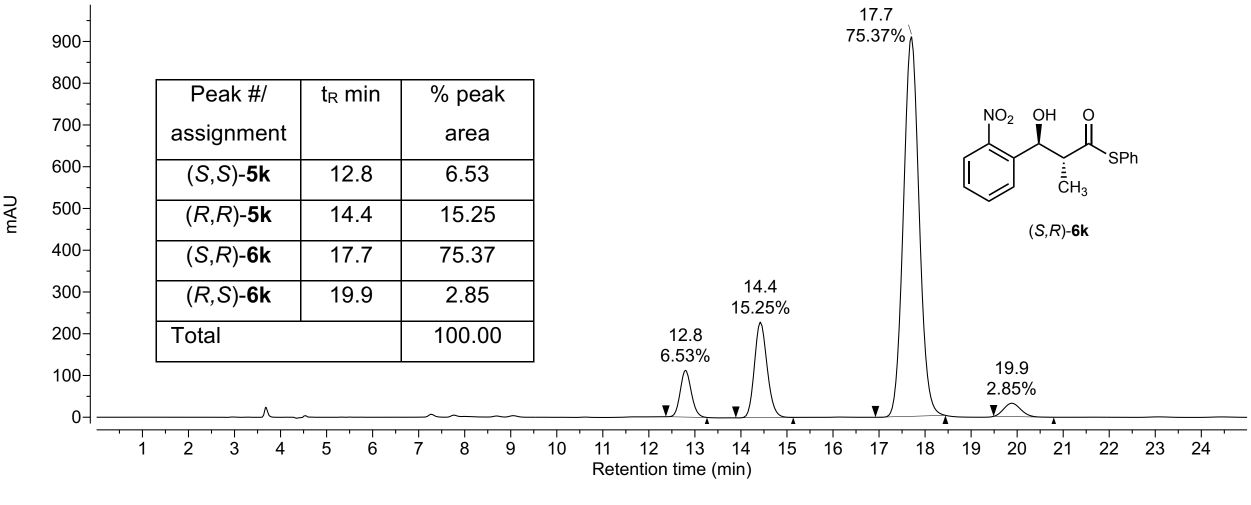

Peak #/ assignment	t_R min	% peak area
(*S*,*S*)-**5k**	12.8	6.53
(*R*,*R*)-**5k**	14.4	15.25
(*S*,*R*)-**6k**	17.7	75.37
(*R*,*S*)-**6k**	19.9	2.85
Total		100.00

HPLC (OD-H, *n*-hexane/^{i}PrOH=90:10, 1.0 mL/min, 298 K, 254 nm) for **5l** and **6l**:

Racemic aldol product:

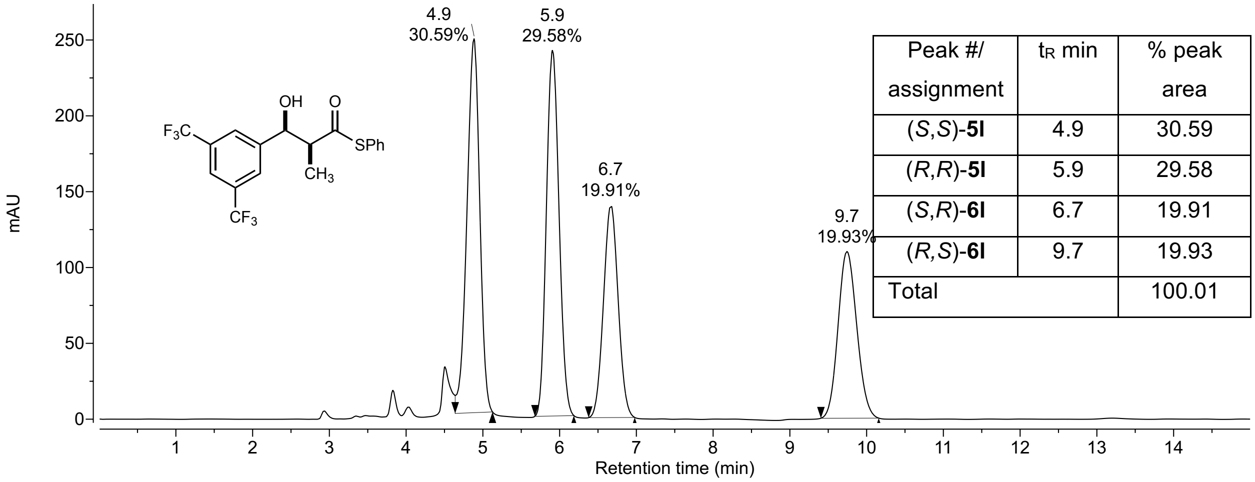

Peak #/ assignment	t_R min	% peak area
(*S*,*S*)-**5l**	4.9	30.59
(*R*,*R*)-**5l**	5.9	29.58
(*S*,*R*)-**6l**	6.7	19.91
(*R*,*S*)-**6l**	9.7	19.93
Total		100.01

Syn-aldol product:

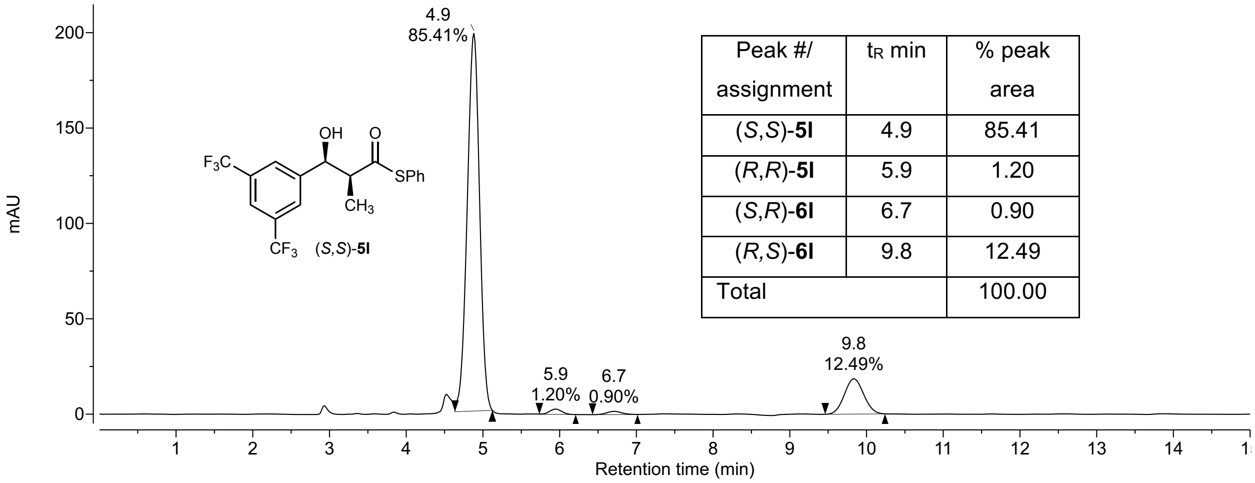

Peak #/ assignment	t_R min	% peak area
(*S*,*S*)-**5l**	4.9	85.41
(*R*,*R*)-**5l**	5.9	1.20
(*S*,*R*)-**6l**	6.7	0.90
(*R*,*S*)-**6l**	9.8	12.49
Total		100.00

Anti-aldol product:

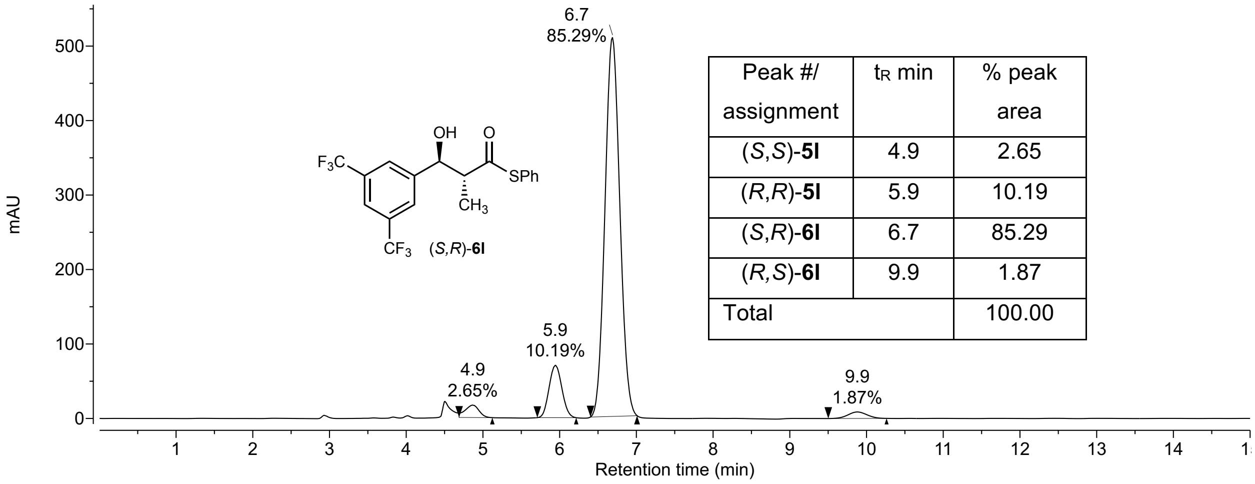

Peak #/ assignment	t_R min	% peak area
(*S*,*S*)-**5l**	4.9	2.65
(*R*,*R*)-**5l**	5.9	10.19
(*S*,*R*)-**6l**	6.7	85.29
(*R*,*S*)-**6l**	9.9	1.87
Total		100.00

HPLC (AD-H, *n*-hexane/^{i}PrOH=92:8, 1.0 mL/min, 298 K, 254 nm) for **5m** and **6m**:

Racemic aldol product:

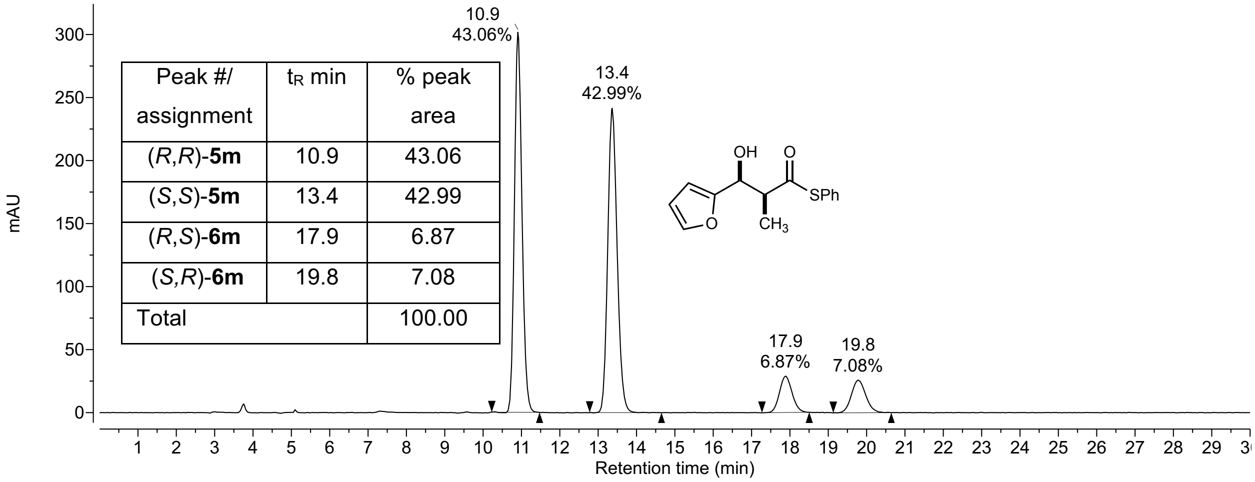

Peak #/ assignment	t_R min	% peak area
(*R*,*R*)-**5m**	10.9	43.06
(*S*,*S*)-**5m**	13.4	42.99
(*R*,*S*)-**6m**	17.9	6.87
(*S*,*R*)-**6m**	19.8	7.08
Total		100.00

Syn-aldol product:

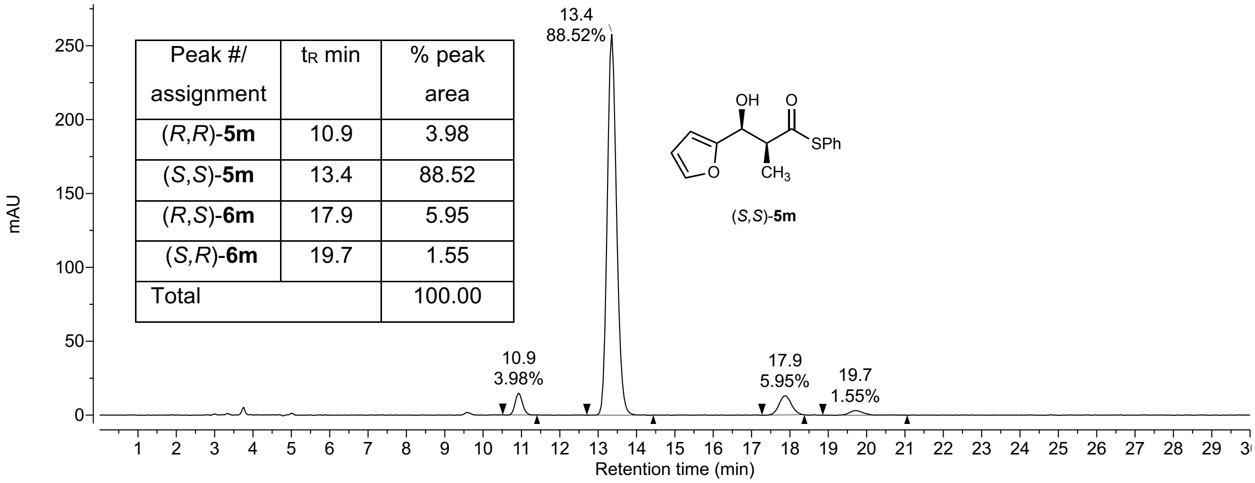

Peak #/ assignment	t_R min	% peak area
(*R*,*R*)-**5m**	10.9	3.98
(*S*,*S*)-**5m**	13.4	88.52
(*R*,*S*)-**6m**	17.9	5.95
(*S*,*R*)-**6m**	19.7	1.55
Total		100.00

Anti-aldol product:

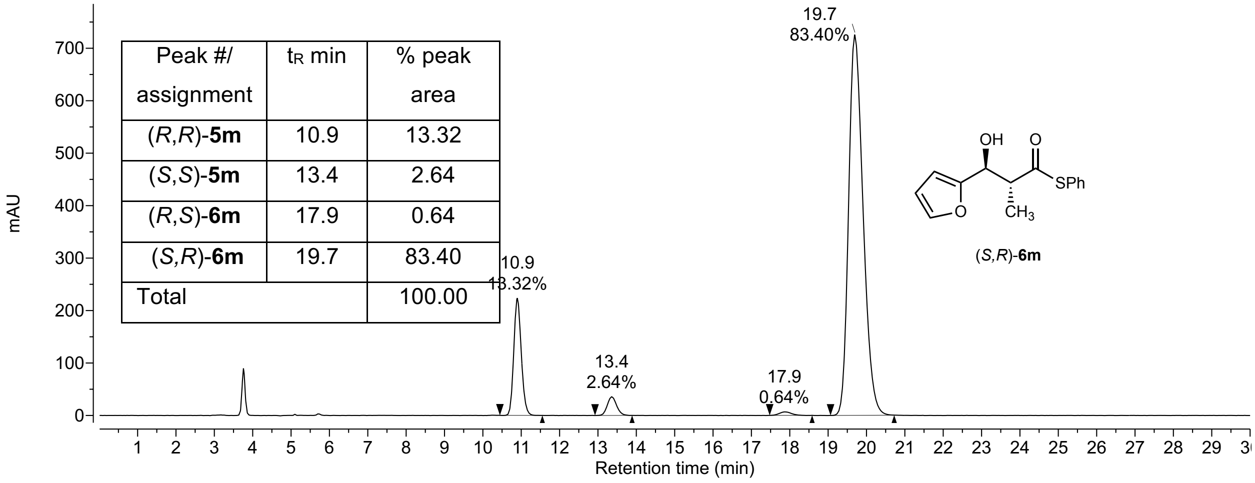

Peak #/ assignment	t_R min	% peak area
(*R*,*R*)-**5m**	10.9	13.32
(*S*,*S*)-**5m**	13.4	2.64
(*R*,*S*)-**6m**	17.9	0.64
(*S*,*R*)-**6m**	19.7	83.40
Total		100.00

HPLC (AD-H, *n*-hexane/EtOH=92:8, 1.0 mL/min, 298 K, 254 nm) for **5n** and **6n**:

Racemic aldol product:

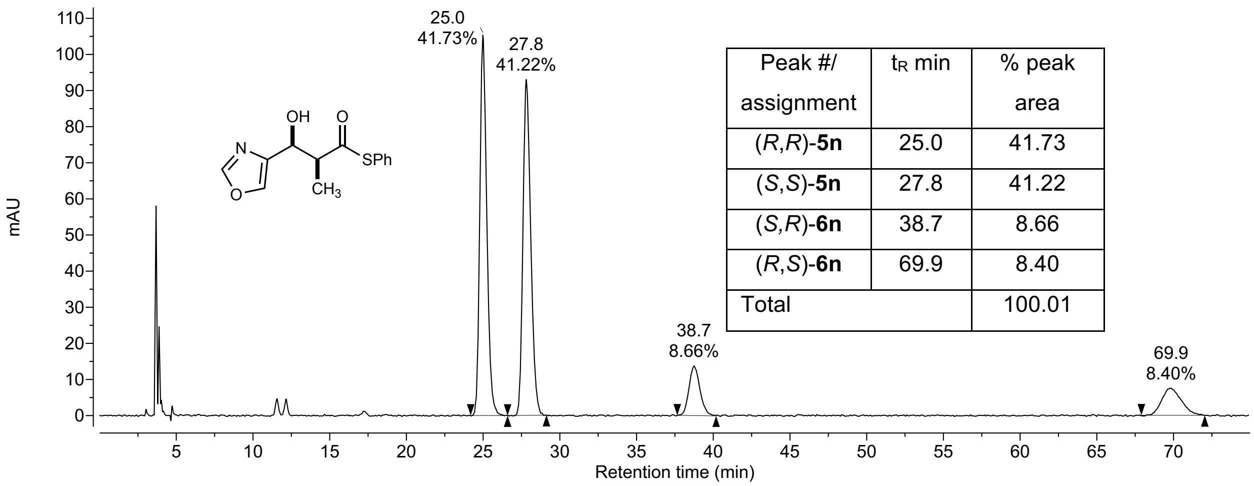

Peak #/ assignment	t_R min	% peak area
(*R*,*R*)-**5n**	25.0	41.73
(*S*,*S*)-**5n**	27.8	41.22
(*S*,*R*)-**6n**	38.7	8.66
(*R*,*S*)-**6n**	69.9	8.40
Total		100.01

Syn-aldol product:

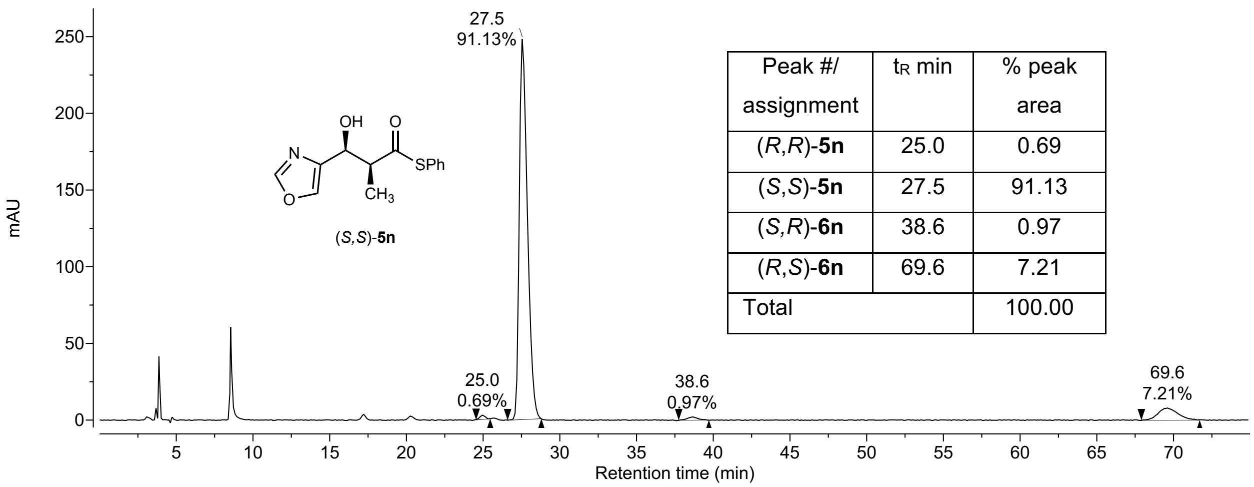

Peak #/ assignment	t_R min	% peak area
(*R*,*R*)-**5n**	25.0	0.69
(*S*,*S*)-**5n**	27.5	91.13
(*S*,*R*)-**6n**	38.6	0.97
(*R*,*S*)-**6n**	69.6	7.21
Total		100.00

Anti-aldol product:

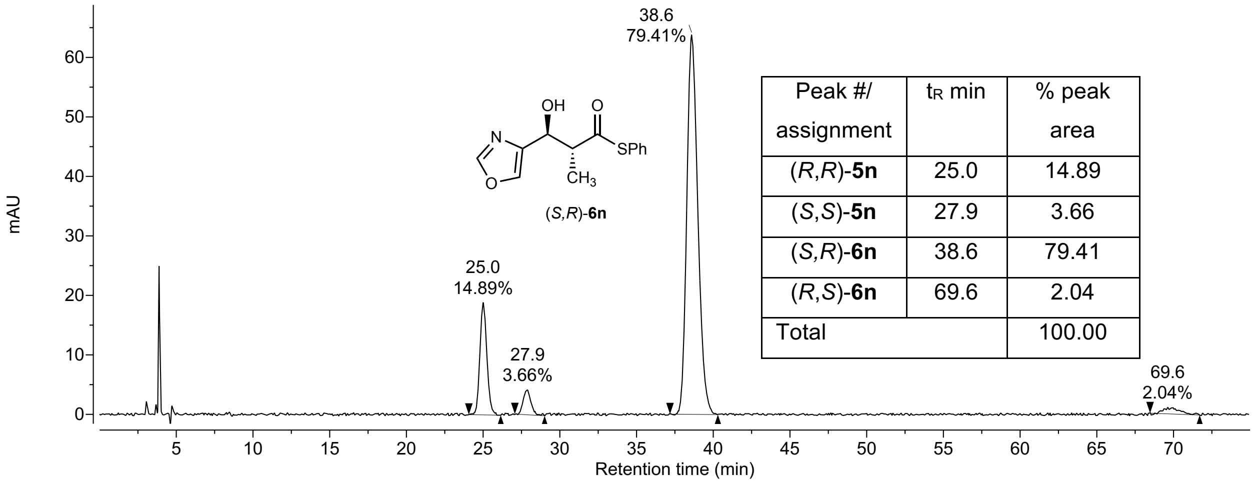

Peak #/ assignment	t_R min	% peak area
(*R*,*R*)-**5n**	25.0	14.89
(*S*,*S*)-**5n**	27.9	3.66
(*S*,*R*)-**6n**	38.6	79.41
(*R*,*S*)-**6n**	69.6	2.04
Total		100.00

HPLC (AD-H, *n*-hexane/EtOH=90:10, 1.0 mL/min, 298 K, 254 nm) for **5o** and **6o**:

Racemic aldol product:

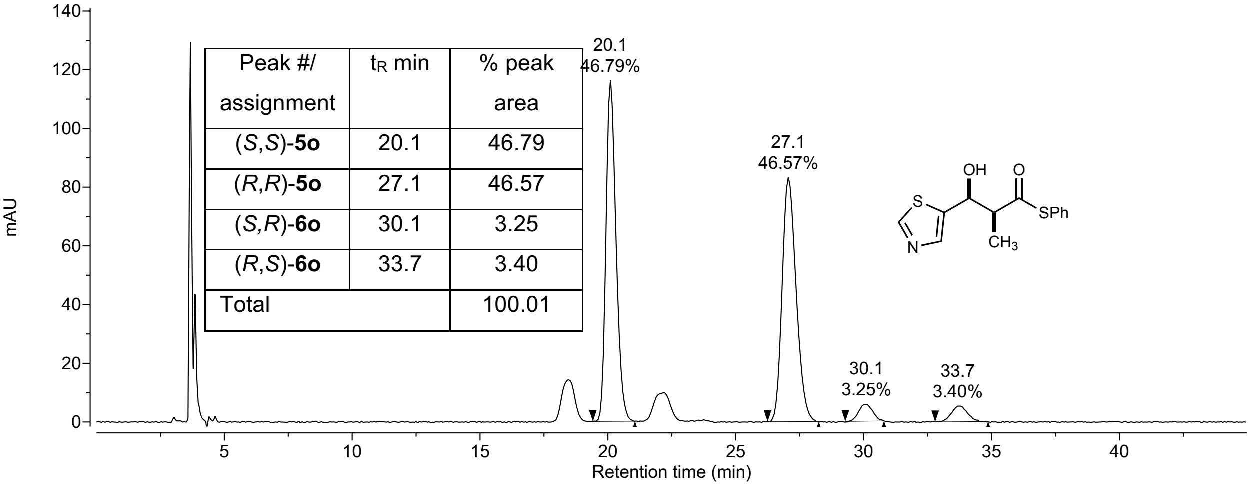

Peak #/ assignment	t_R min	% peak area
(*S*,*S*)-**5o**	20.1	46.79
(*R*,*R*)-**5o**	27.1	46.57
(*S*,*R*)-**6o**	30.1	3.25
(*R*,*S*)-**6o**	33.7	3.40
Total		100.01

Syn-aldol product:

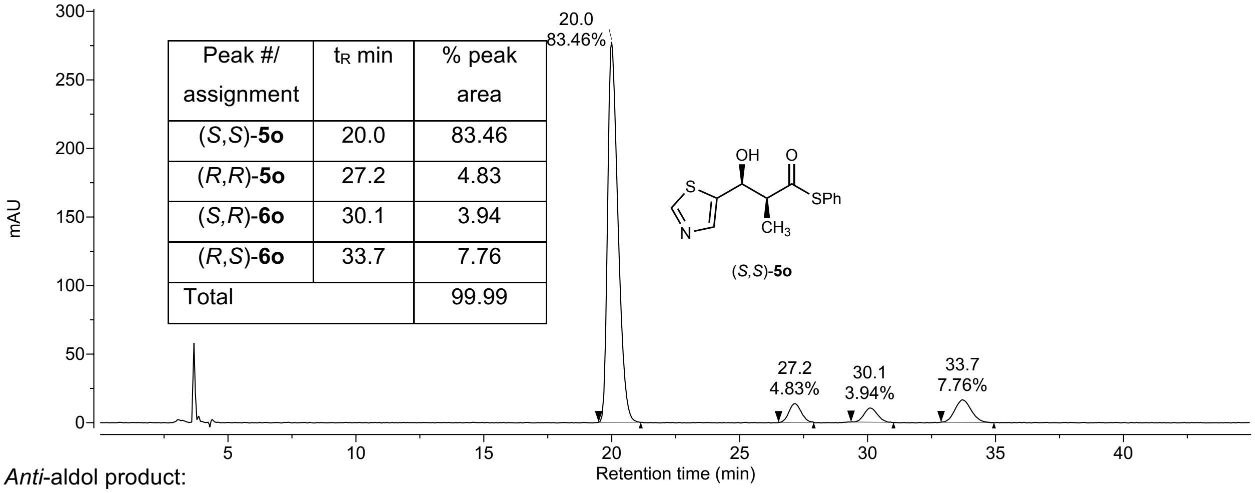

Peak #/ assignment	t_R min	% peak area
(*S*,*S*)-**5o**	20.0	83.46
(*R*,*R*)-**5o**	27.2	4.83
(*S*,*R*)-**6o**	30.1	3.94
(*R*,*S*)-**6o**	33.7	7.76
Total		99.99

Anti-aldol product:

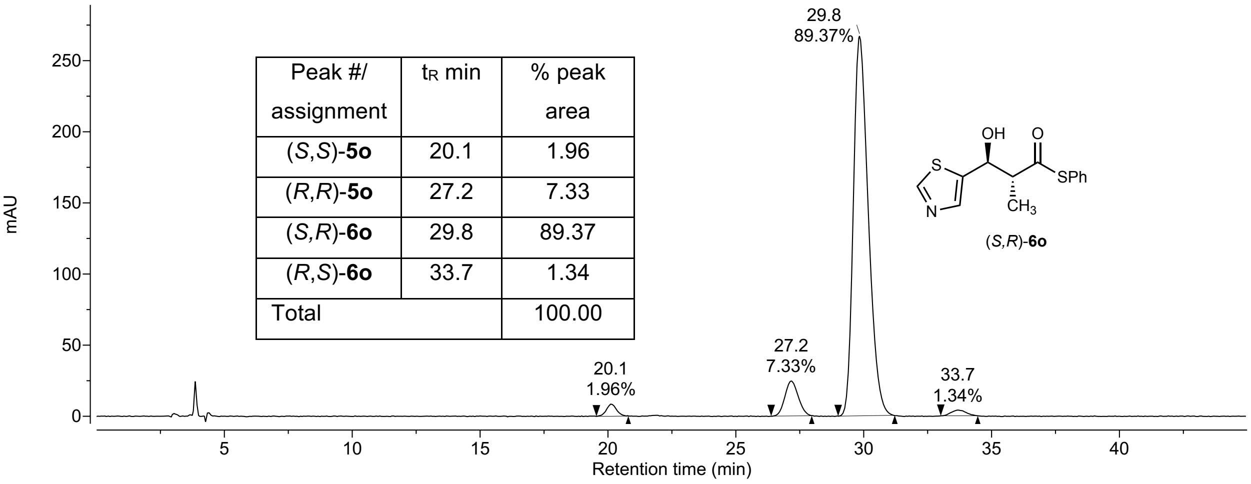

Peak #/ assignment	t_R min	% peak area
(*S*,*S*)-**5o**	20.1	1.96
(*R*,*R*)-**5o**	27.2	7.33
(*S*,*R*)-**6o**	29.8	89.37
(*R*,*S*)-**6o**	33.7	1.34
Total		100.00

HPLC (AD-H, *n*-hexane/^{i}PrOH=90:10, 1.0 mL/min, 298 K, 254 nm) for **5p** and **6p**:

Racemic aldol product:

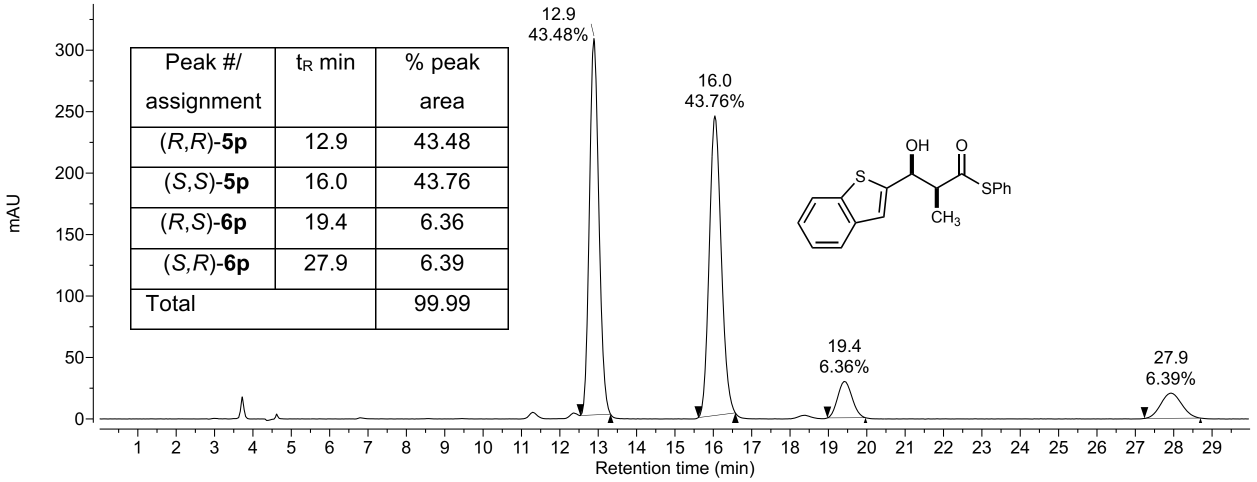

Peak #/ assignment	t_R min	% peak area
(*R*,*R*)-**5p**	12.9	43.48
(*S*,*S*)-**5p**	16.0	43.76
(*R*,*S*)-**6p**	19.4	6.36
(*S*,*R*)-**6p**	27.9	6.39
Total		99.99

Syn-aldol product:

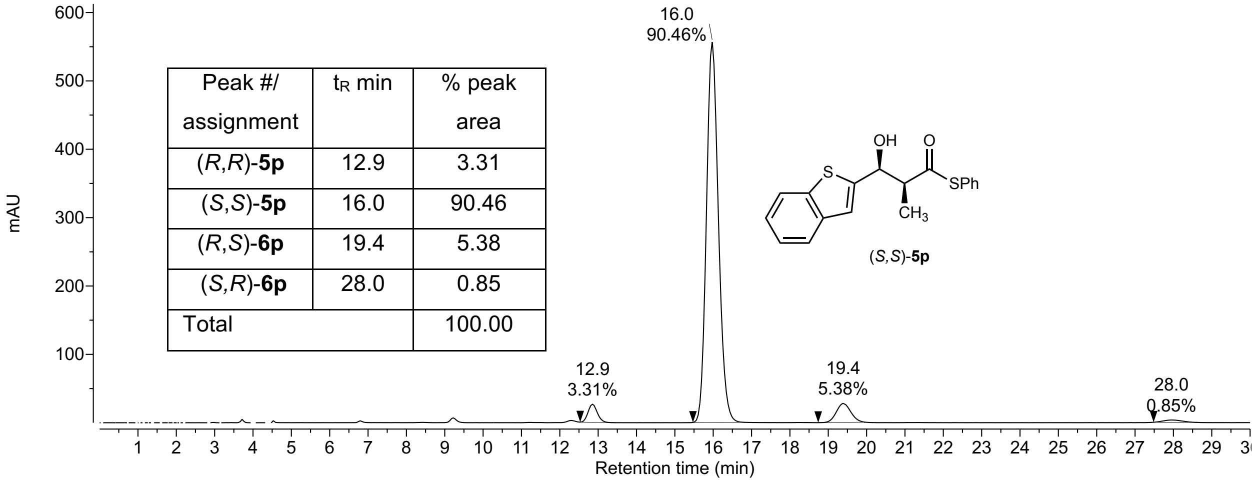

Peak #/ assignment	t_R min	% peak area
(*R*,*R*)-**5p**	12.9	3.31
(*S*,*S*)-**5p**	16.0	90.46
(*R*,*S*)-**6p**	19.4	5.38
(*S*,*R*)-**6p**	28.0	0.85
Total		100.00

Anti-aldol product:

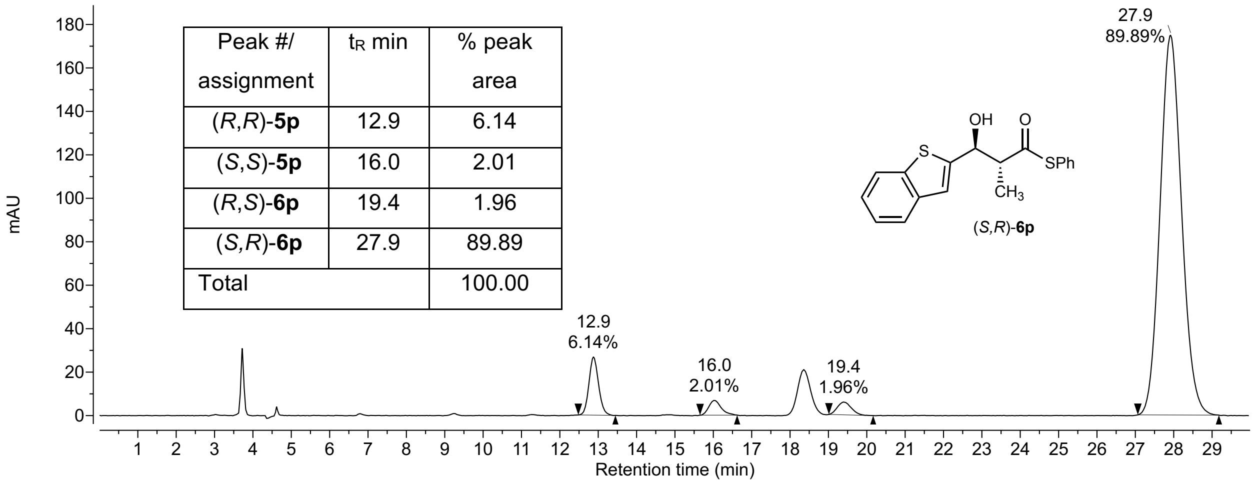

Peak #/ assignment	t_R min	% peak area
(*R*,*R*)-**5p**	12.9	6.14
(*S*,*S*)-**5p**	16.0	2.01
(*R*,*S*)-**6p**	19.4	1.96
(*S*,*R*)-**6p**	27.9	89.89
Total		100.00

HPLC (AD-H, *n*-hexane/^{i}PrOH=90:10, 1.0 mL/min, 298 K, 254 nm) for **5q** and **6q**:

Racemic aldol product:

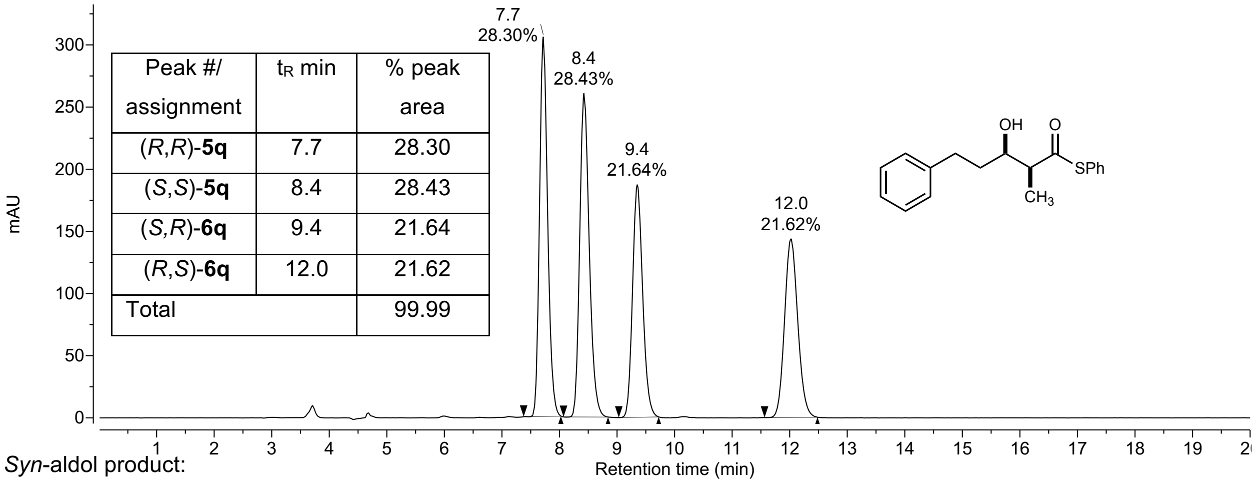

Peak #/ assignment	t_R min	% peak area
(*R*,*R*)-**5q**	7.7	28.30
(*S*,*S*)-**5q**	8.4	28.43
(*S*,*R*)-**6q**	9.4	21.64
(*R*,*S*)-**6q**	12.0	21.62
Total		99.99

Syn-aldol product:

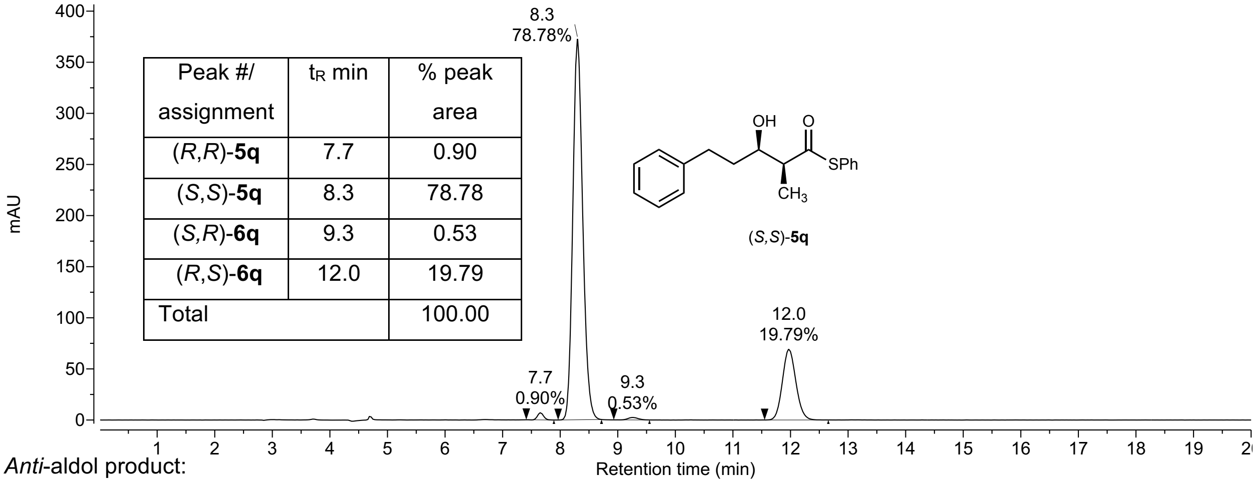

Peak #/ assignment	t_R min	% peak area
(*R*,*R*)-**5q**	7.7	0.90
(*S*,*S*)-**5q**	8.3	78.78
(*S*,*R*)-**6q**	9.3	0.53
(*R*,*S*)-**6q**	12.0	19.79
Total		100.00

Anti-aldol product:

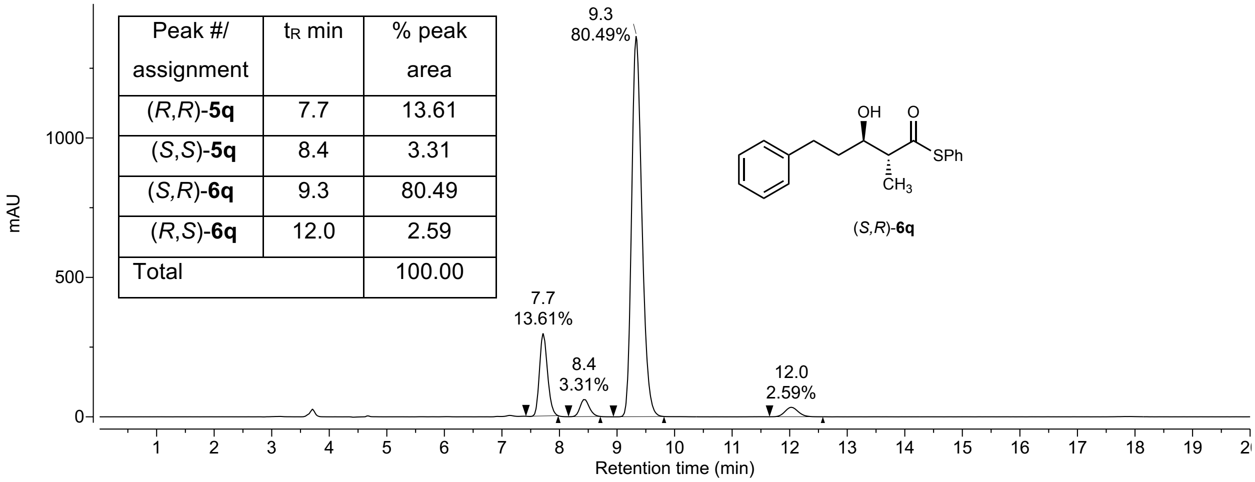

Peak #/ assignment	t_R min	% peak area
(*R*,*R*)-**5q**	7.7	13.61
(*S*,*S*)-**5q**	8.4	3.31
(*S*,*R*)-**6q**	9.3	80.49
(*R*,*S*)-**6q**	12.0	2.59
Total		100.00

HPLC (AD-H, *n*-hexane/^{i}PrOH=93:7, 1.0 mL/min, 298 K, 254 nm) for **5r** and **6r**:

Racemic aldol product:

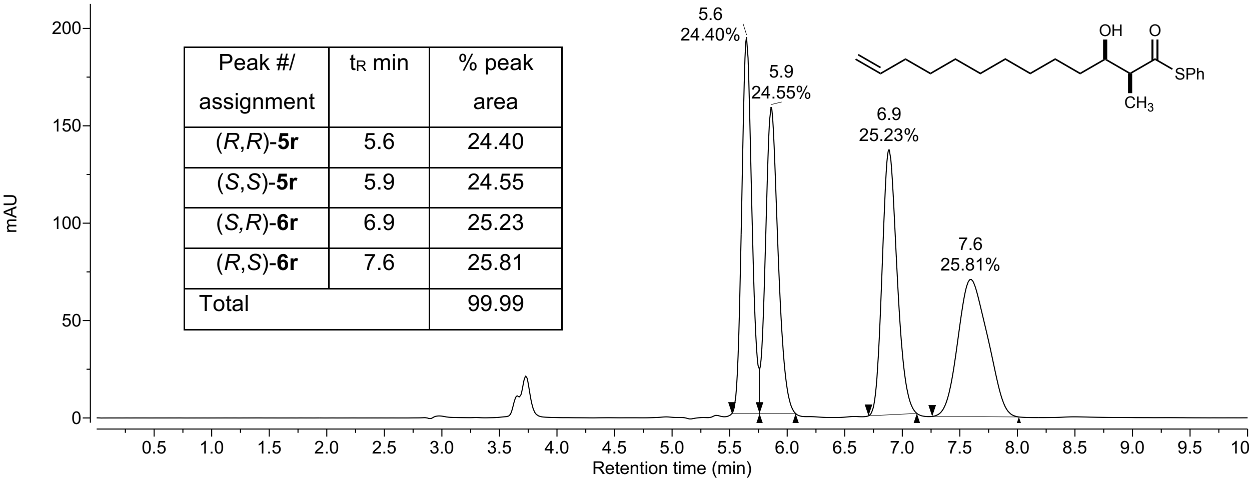

Peak #/ assignment	t_R min	% peak area
(*R*,*R*)-**5r**	5.6	24.40
(*S*,*S*)-**5r**	5.9	24.55
(*S*,*R*)-**6r**	6.9	25.23
(*R*,*S*)-**6r**	7.6	25.81
Total		99.99

Syn-aldol product:

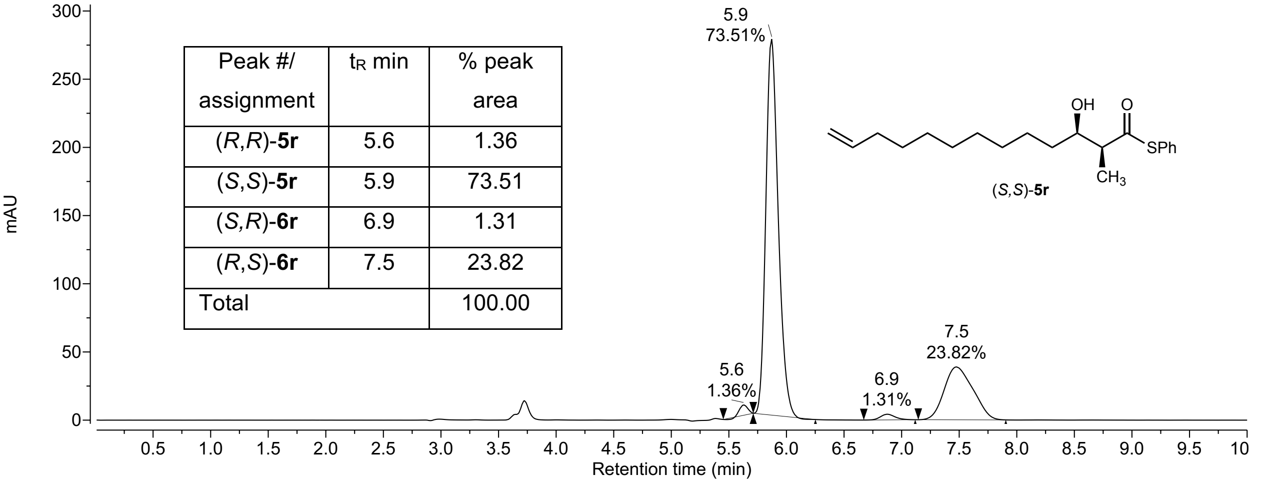

Peak #/ assignment	t_R min	% peak area
(*R*,*R*)-**5r**	5.6	1.36
(*S*,*S*)-**5r**	5.9	73.51
(*S*,*R*)-**6r**	6.9	1.31
(*R*,*S*)-**6r**	7.5	23.82
Total		100.00

Anti-aldol product:

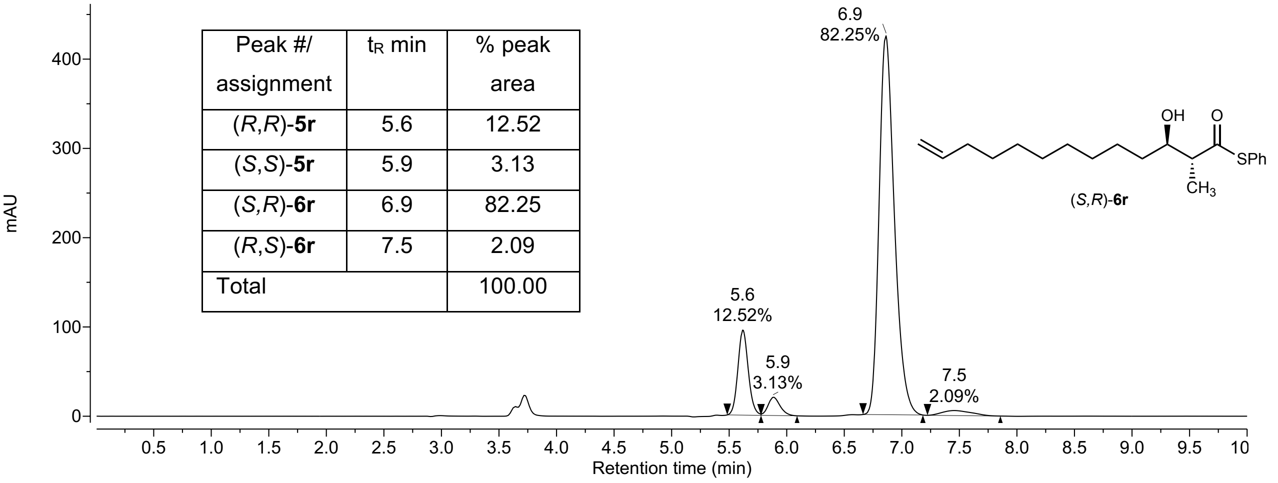

Peak #/ assignment	t_R min	% peak area
(*R*,*R*)-**5r**	5.6	12.52
(*S*,*S*)-**5r**	5.9	3.13
(*S*,*R*)-**6r**	6.9	82.25
(*R*,*S*)-**6r**	7.5	2.09
Total		100.00

HPLC (AD-H, *n*-hexane/EtOH=90:10, 1.0 mL/min, 298 K, 254 nm) for **5s** and **6s**:

Racemic aldol product:

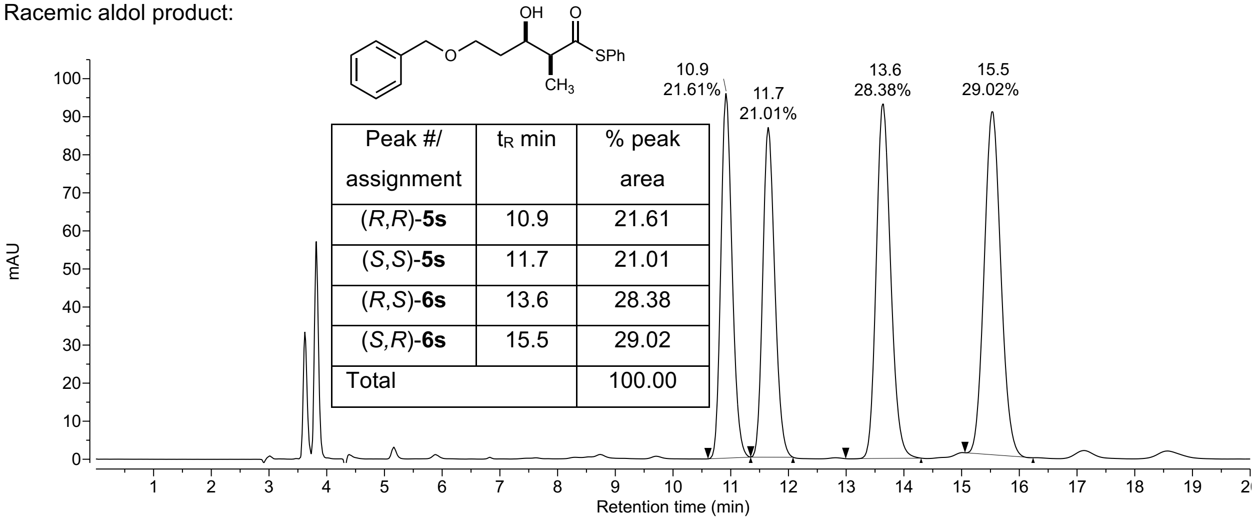

Peak #/ assignment	t_R min	% peak area
(*R*,*R*)-**5s**	10.9	21.61
(*S*,*S*)-**5s**	11.7	21.01
(*R*,*S*)-**6s**	13.6	28.38
(*S*,*R*)-**6s**	15.5	29.02
Total		100.00

Syn-aldol product:

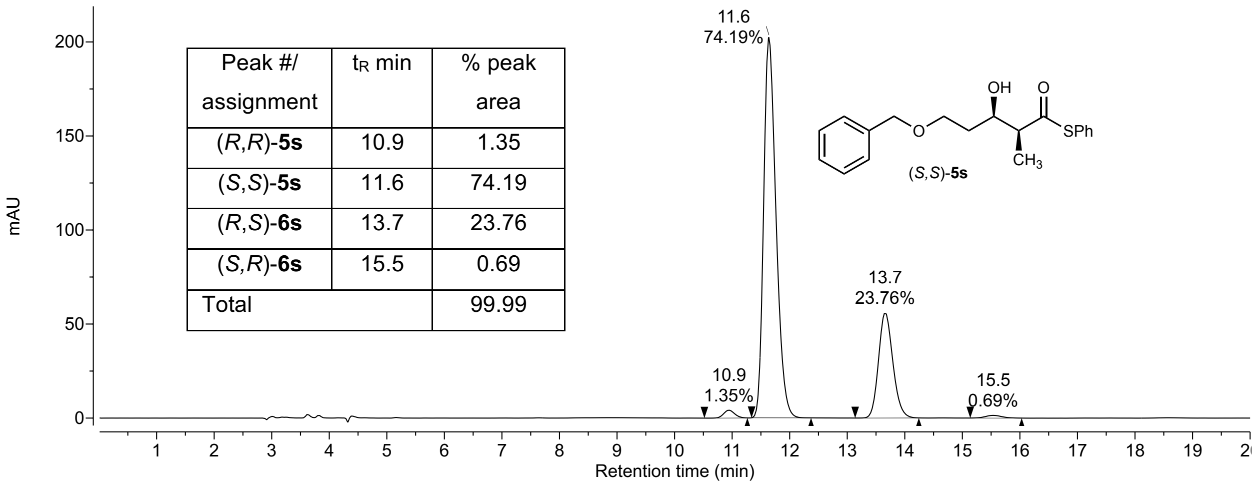

Peak #/ assignment	t_R min	% peak area
(*R*,*R*)-**5s**	10.9	1.35
(*S*,*S*)-**5s**	11.6	74.19
(*R*,*S*)-**6s**	13.7	23.76
(*S*,*R*)-**6s**	15.5	0.69
Total		99.99

Anti-aldol product:

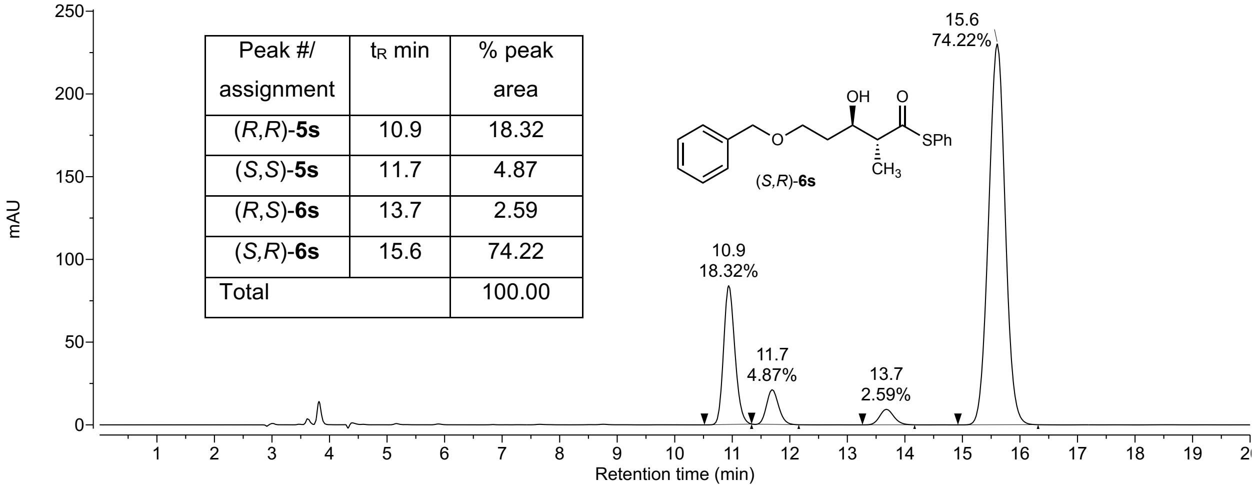

Peak #/ assignment	t_R min	% peak area
(*R*,*R*)-**5s**	10.9	18.32
(*S*,*S*)-**5s**	11.7	4.87
(*R*,*S*)-**6s**	13.7	2.59
(*S*,*R*)-**6s**	15.6	74.22
Total		100.00

HPLC (AD-H, *n*-hexane/^{i}PrOH=92:8, 1.0 mL/min, 298 K, 254 nm) for **5t** and **6t**:

Racemic aldol product:

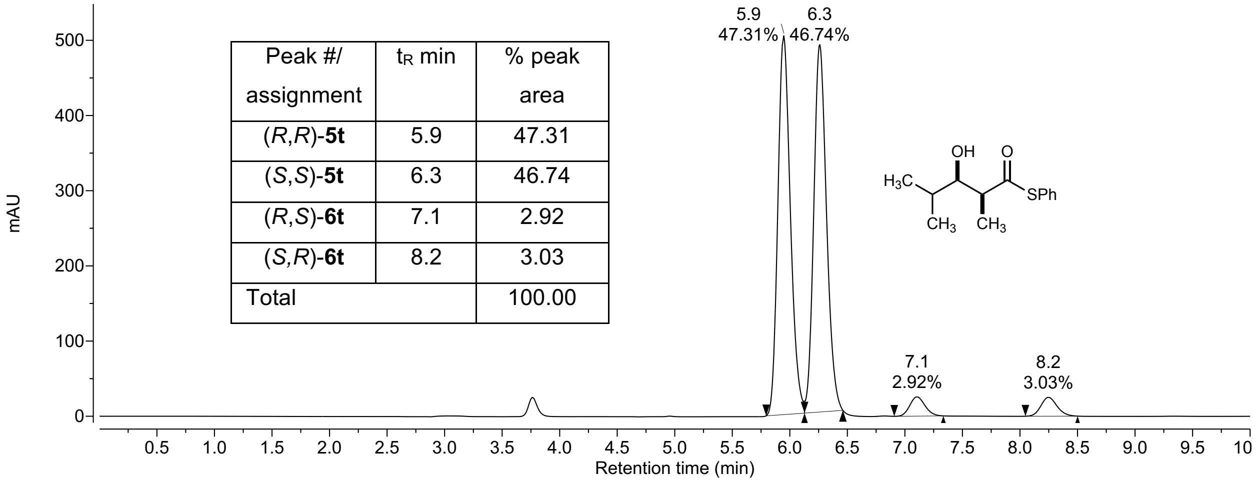

Peak #/ assignment	t_R min	% peak area
(*R*,*R*)-**5t**	5.9	47.31
(*S*,*S*)-**5t**	6.3	46.74
(*R*,*S*)-**6t**	7.1	2.92
(*S*,*R*)-**6t**	8.2	3.03
Total		100.00

Syn-aldol product:

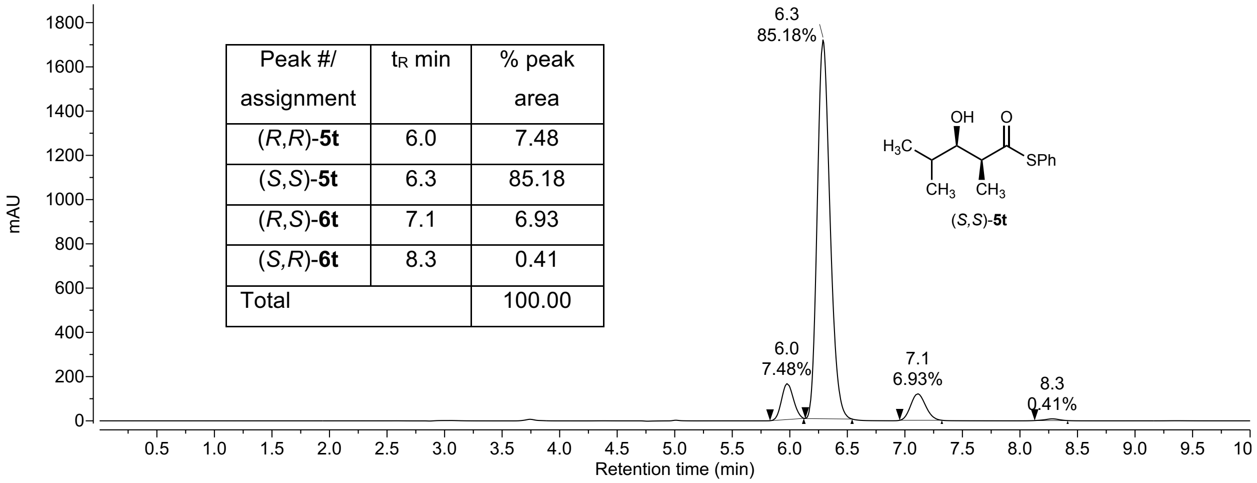

Peak #/ assignment	t_R min	% peak area
(*R*,*R*)-**5t**	6.0	7.48
(*S*,*S*)-**5t**	6.3	85.18
(*R*,*S*)-**6t**	7.1	6.93
(*S,R*)-**6t**	8.3	0.41
Total		100.00

Anti-aldol product:

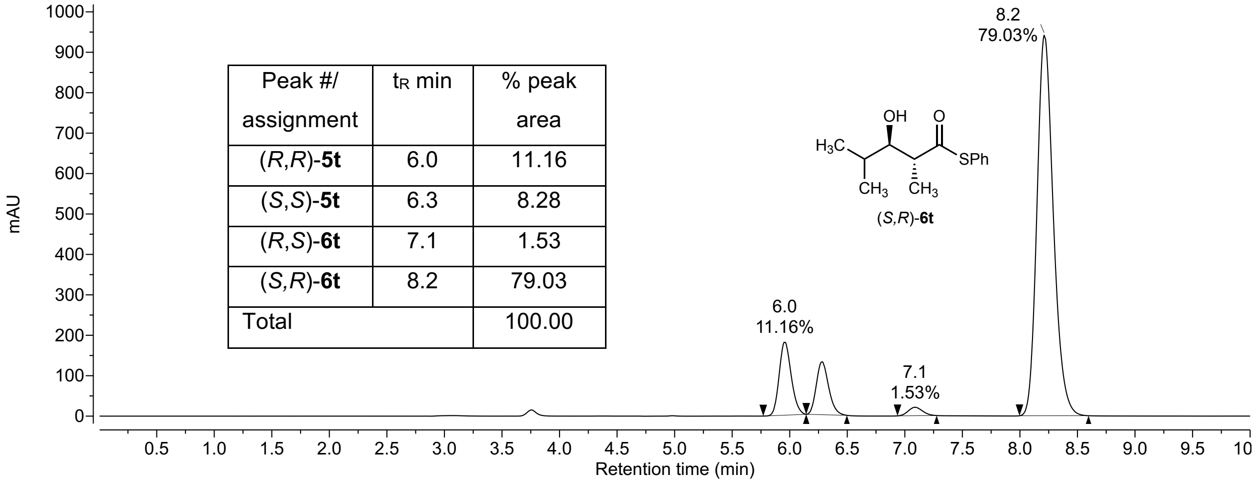

Peak #/ assignment	t_R min	% peak area
(*R*,*R*)-**5t**	6.0	11.16
(*S*,*S*)-**5t**	6.3	8.28
(*R*,*S*)-**6t**	7.1	1.53
(*S*,*R*)-**6t**	8.2	79.03
Total		100.00

HPLC (AD-H, *n*-hexane/EtOH=90:10, 1.0 mL/min, 298 K, 254 nm) for **5u** and **6u**:

Racemic aldol product:

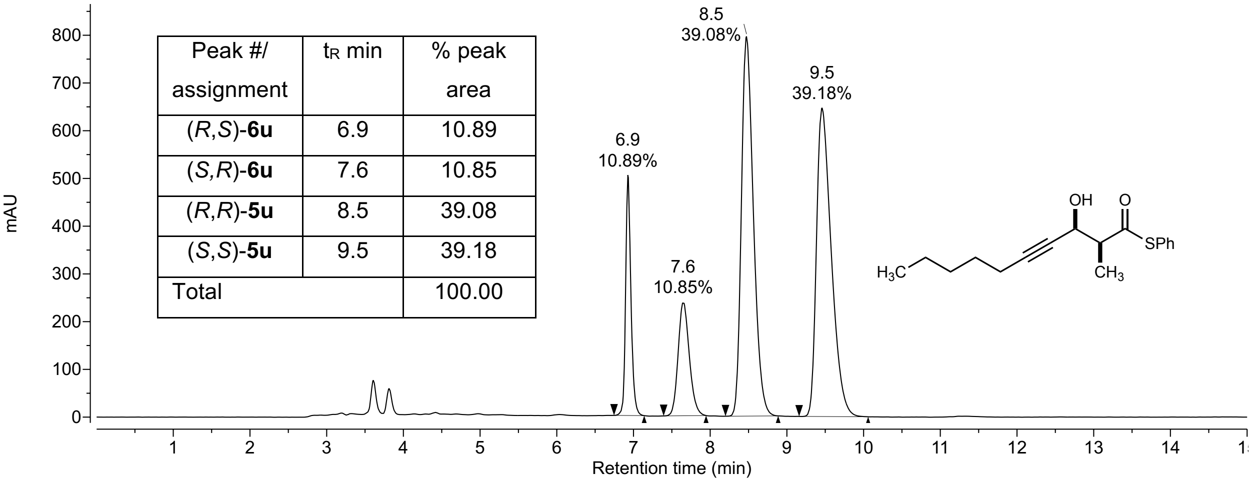

Peak #/ assignment	t_R min	% peak area
(*R*,*S*)-**6u**	6.9	10.89
(*S*,*R*)-**6u**	7.6	10.85
(*R*,*R*)-**5u**	8.5	39.08
(*S*,*S*)-**5u**	9.5	39.18
Total		100.00

Syn-aldol product:

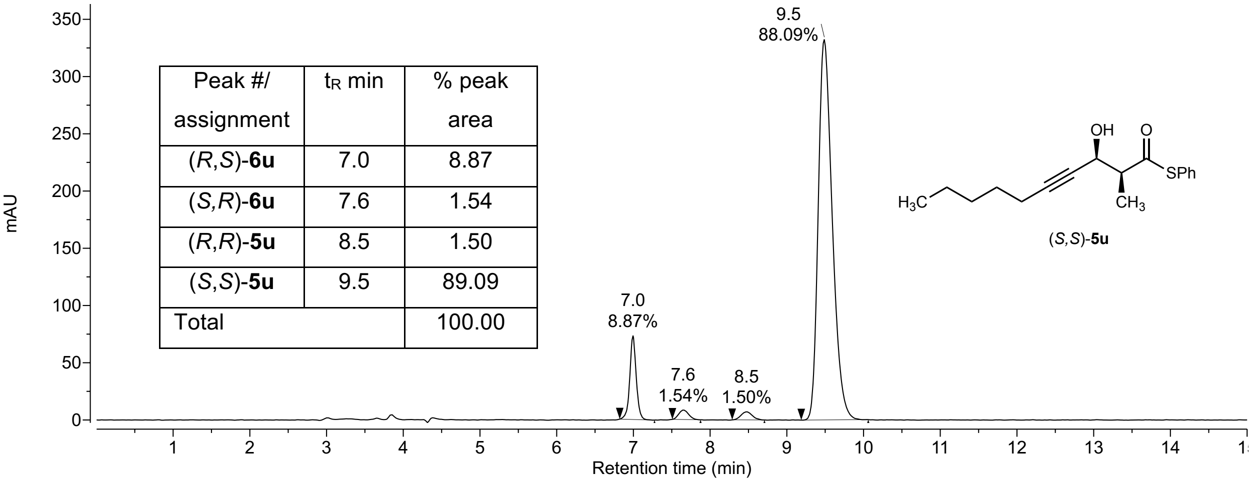

Peak #/ assignment	t_R min	% peak area
(*R*,*S*)-**6u**	7.0	8.87
(*S*,*R*)-**6u**	7.6	1.54
(*R*,*R*)-**5u**	8.5	1.50
(*S*,*S*)-**5u**	9.5	89.09
Total		100.00

Anti-aldol product:

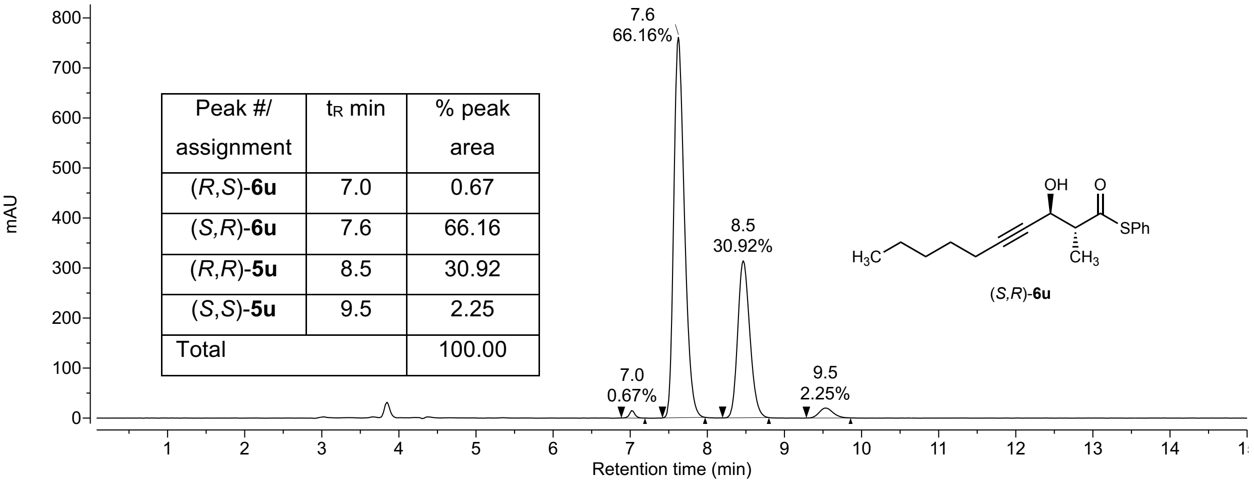

Peak #/ assignment	t_R min	% peak area
(*R*,*S*)-**6u**	7.0	0.67
(*S*,*R*)-**6u**	7.6	66.16
(*R*,*R*)-**5u**	8.5	30.92
(*S*,*S*)-**5u**	9.5	2.25
Total		100.00

HPLC (AD-H, *n*-hexane/EtOH=96:4, 1.0 mL/min, 298 K, 254 nm) for **5v** and **6v**:

Racemic aldol product:

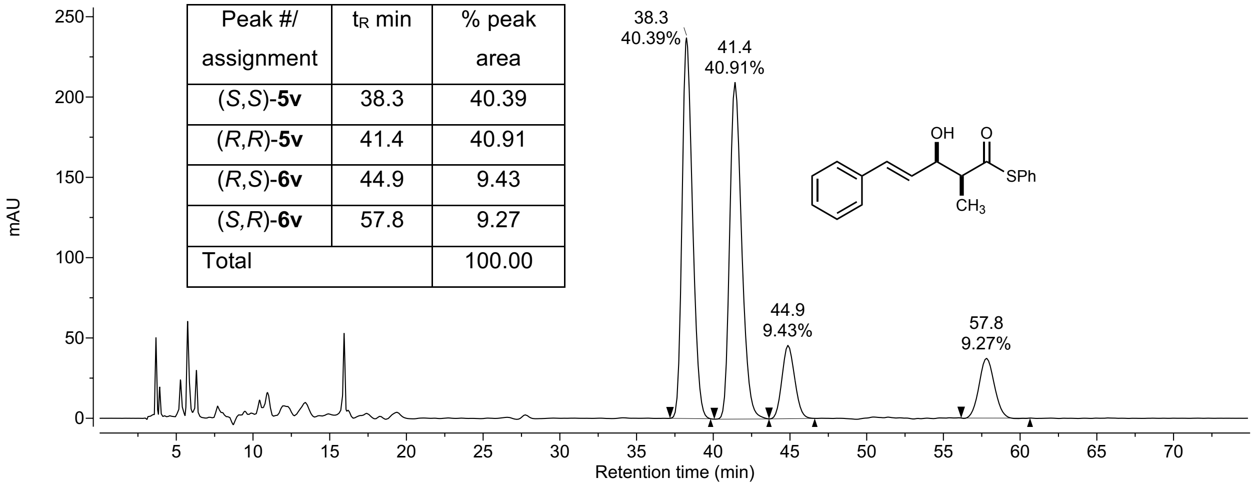

Peak #/ assignment	t_R min	% peak area
(*S*,*S*)-**5v**	38.3	40.39
(*R*,*R*)-**5v**	41.4	40.91
(*R*,*S*)-**6v**	44.9	9.43
(*S*,*R*)-**6v**	57.8	9.27
Total		100.00

Syn-aldol product:

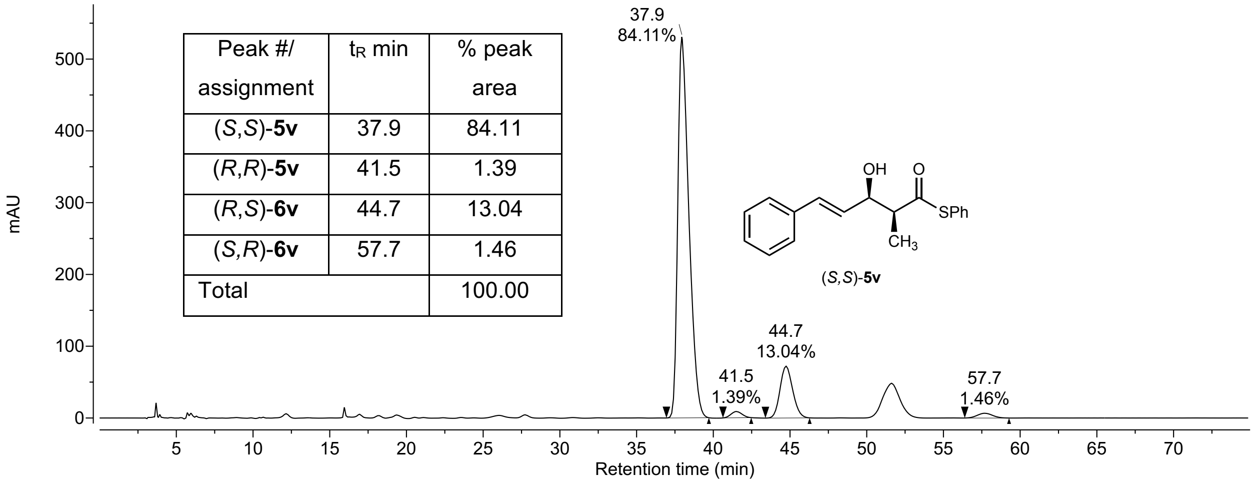

Peak #/ assignment	t_R min	% peak area
(*S*,*S*)-**5v**	37.9	84.11
(*R*,*R*)-**5v**	41.5	1.39
(*R*,*S*)-**6v**	44.7	13.04
(*S*,*R*)-**6v**	57.7	1.46
Total		100.00

Anti-aldol product:

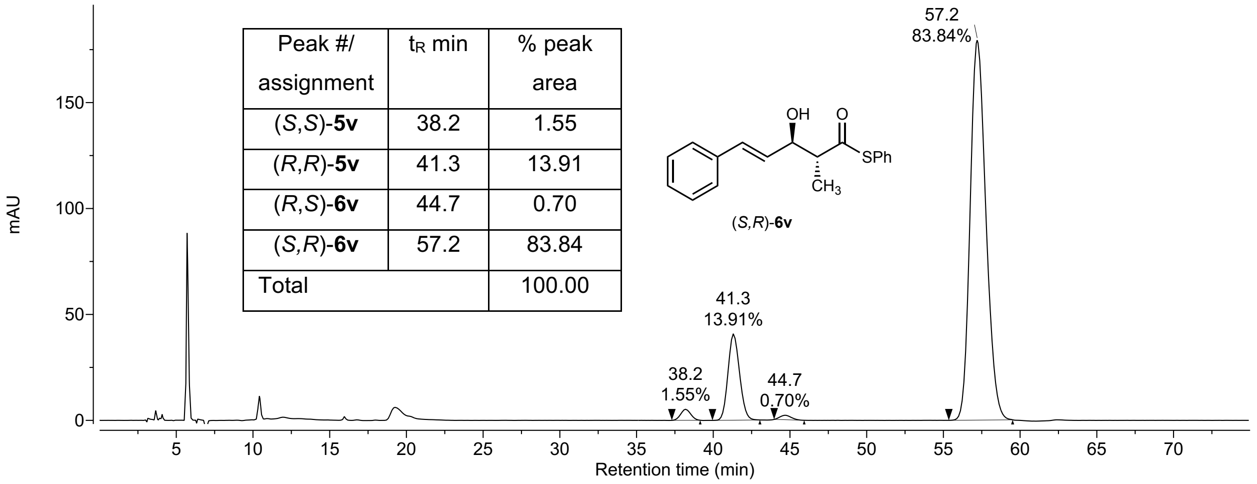

Peak #/ assignment	t_R min	% peak area
(*S*,*S*)-**5v**	38.2	1.55
(*R*,*R*)-**5v**	41.3	13.91
(*R*,*S*)-**6v**	44.7	0.70
(*S*,*R*)-**6v**	57.2	83.84
Total		100.00

HPLC (AD-H, *n*-hexane/EtOH=90:10, 1.0 mL/min, 298 K, 254 nm) for **5w**:

Racemic aldol product:

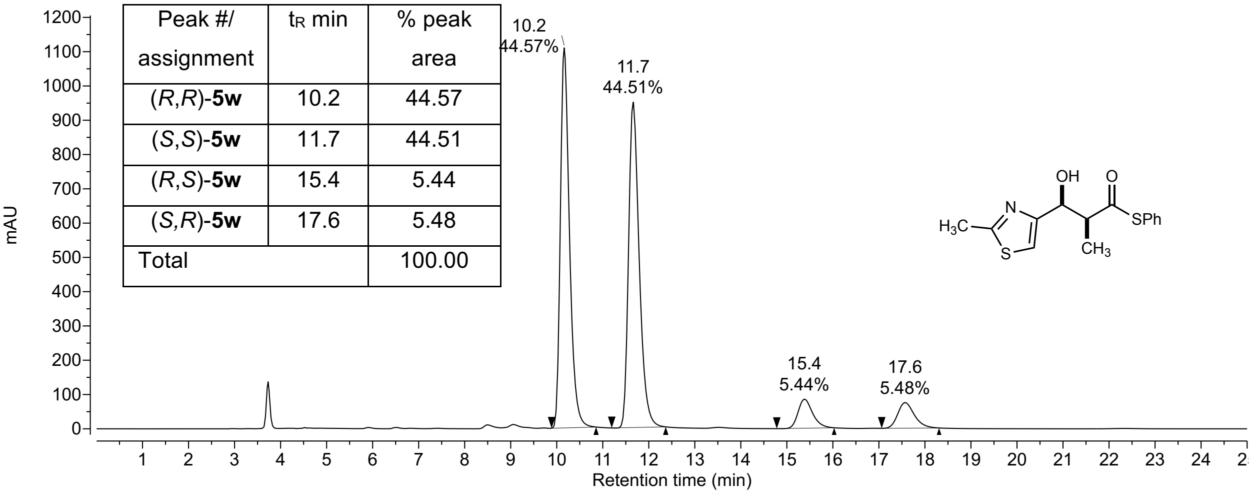

Peak #/ assignment	t_R min	% peak area
(*R*,*R*)-**5w**	10.2	44.57
(*S*,*S*)-**5w**	11.7	44.51
(*R*,*S*)-**5w**	15.4	5.44
(*S*,*R*)-**5w**	17.6	5.48
Total		100.00

Syn-aldol product:

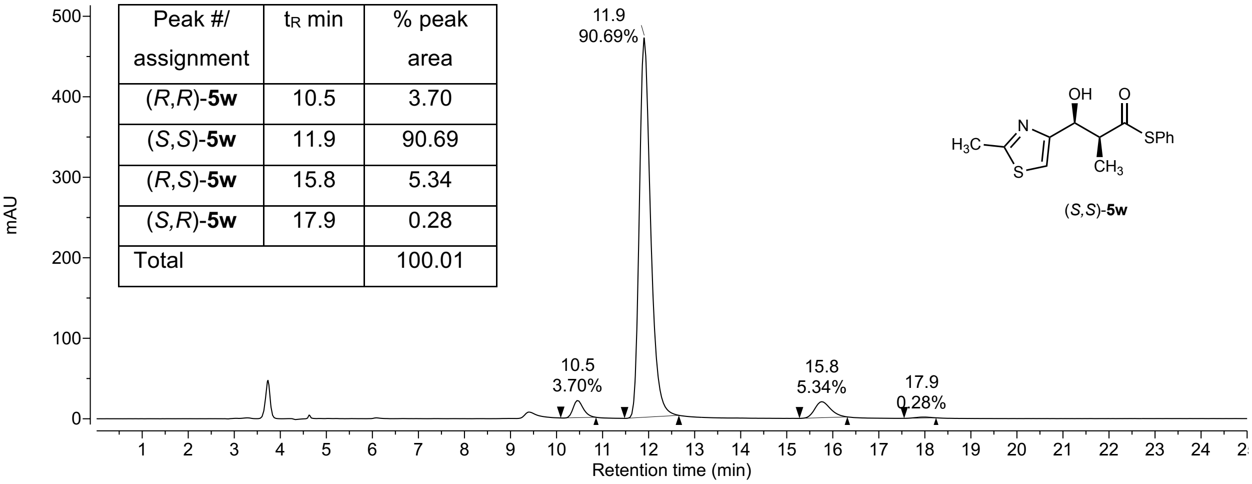

Peak #/ assignment	t_R min	% peak area
(*R*,*R*)-**5w**	10.5	3.70
(*S*,*S*)-**5w**	11.9	90.69
(*R*,*S*)-**5w**	15.8	5.34
(*S*,*R*)-**5w**	17.9	0.28
Total		100.01

HPLC (AD-H, *n*-hexane/^{i}PrOH=85:15, 1.0 mL/min, 298 K, 254 nm) for **6x**:

Racemic aldol product:

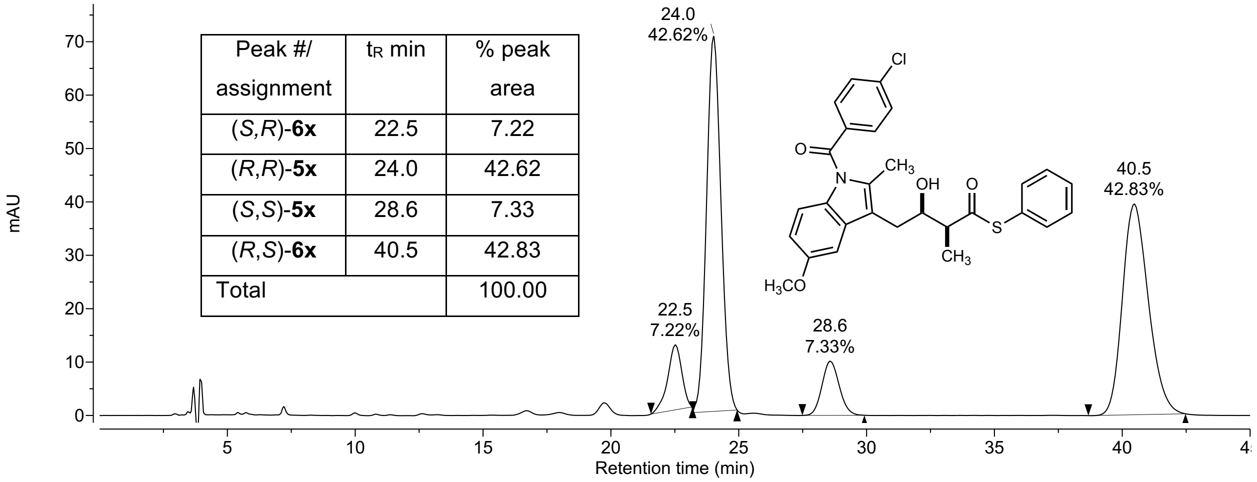

Peak #/ assignment	t_R min	% peak area
(*S*,*R*)-**6x**	22.5	7.22
(*R*,*R*)-**5x**	24.0	42.62
(*S*,*S*)-**5x**	28.6	7.33
(*R*,*S*)-**6x**	40.5	42.83
Total		100.00

Anti-aldol product (crude):

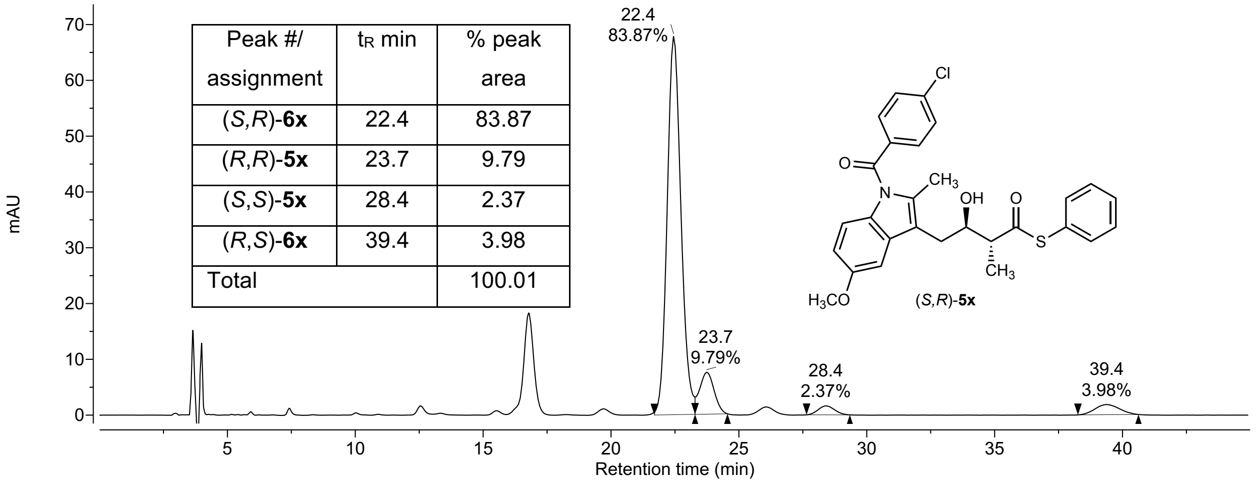

Peak #/ assignment	t_R min	% peak area
(*S*,*R*)-**6x**	22.4	83.87
(*R*,*R*)-**5x**	23.7	9.79
(*S*,*S*)-**5x**	28.4	2.37
(*R*,*S*)-**6x**	39.4	3.98
Total		100.01

Anti-aldol after recrystallization:

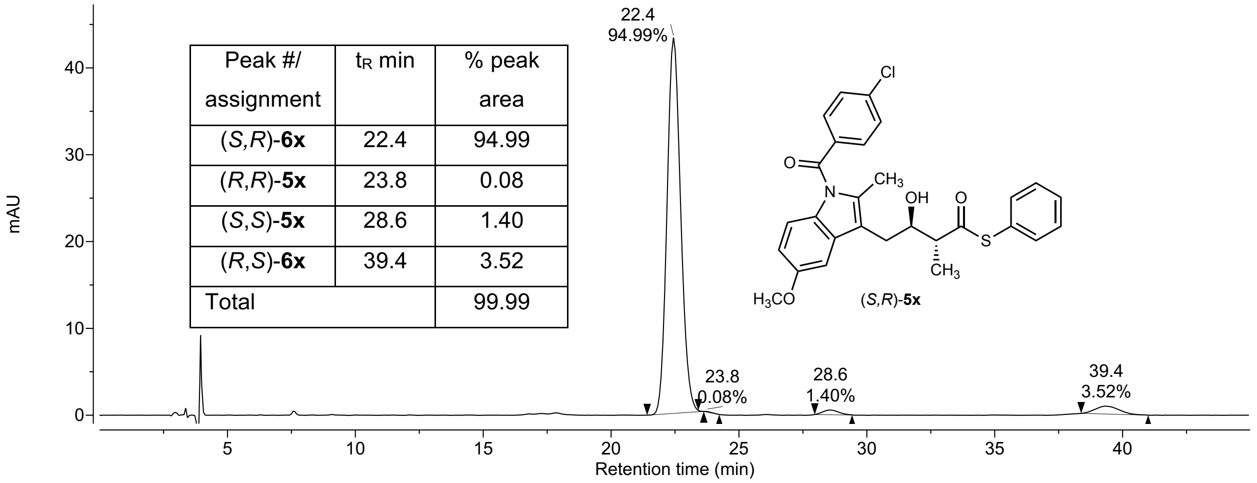

Peak #/ assignment	t_R min	% peak area
(*S*,*R*)-**6x**	22.4	94.99
(*R*,*R*)-**5x**	23.8	0.08
(*S*,*S*)-**5x**	28.6	1.40
(*R*,*S*)-**6x**	39.4	3.52
Total		99.99

HPLC (AD-H, *n*-hexane/^{i}PrOH=90:10, 1.0 mL/min, 298 K, 254 nm) for **S5a** and **S6a**:

Racemic aldol product:

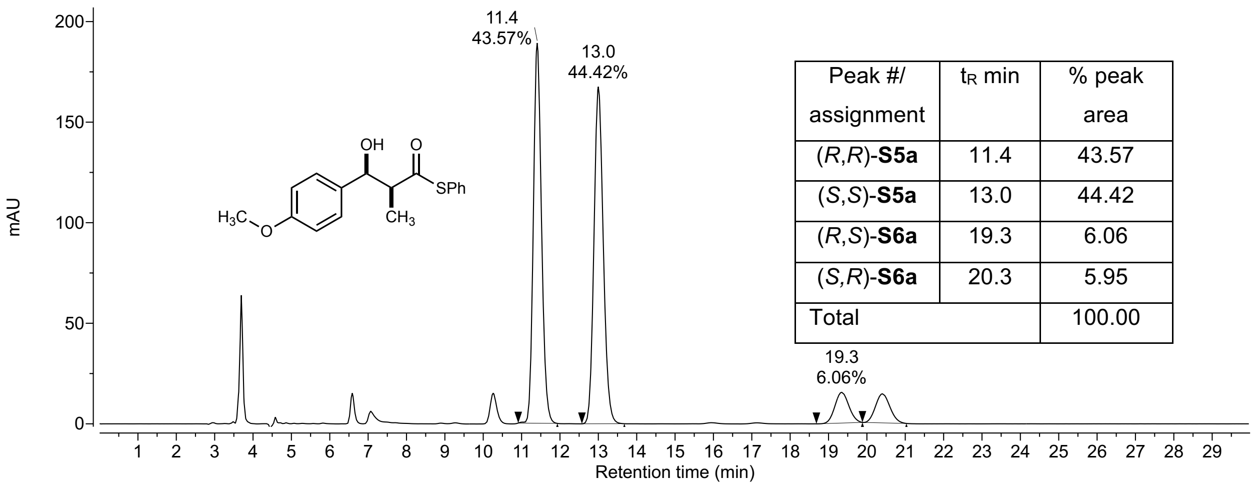

Peak #/ assignment	t_R min	% peak area
(*R*,*R*)-**S5a**	11.4	43.57
(*S*,*S*)-**S5a**	13.0	44.42
(*R*,*S*)-**S6a**	19.3	6.06
(*S*,*R*)-**S6a**	20.3	5.95
Total		100.00

Syn-aldol product:

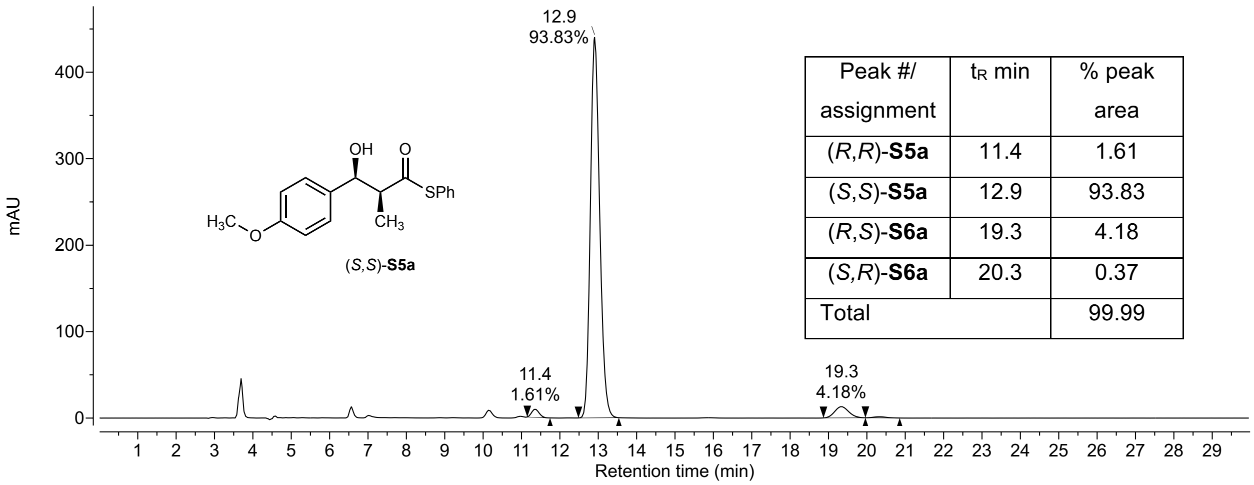

Peak #/ assignment	t_R min	% peak area
(*R*,*R*)-**S5a**	11.4	1.61
(*S*,*S*)-**S5a**	12.9	93.83
(*R*,*S*)-**S6a**	19.3	4.18
(*S*,*R*)-**S6a**	20.3	0.37
Total		99.99

Anti-aldol product:

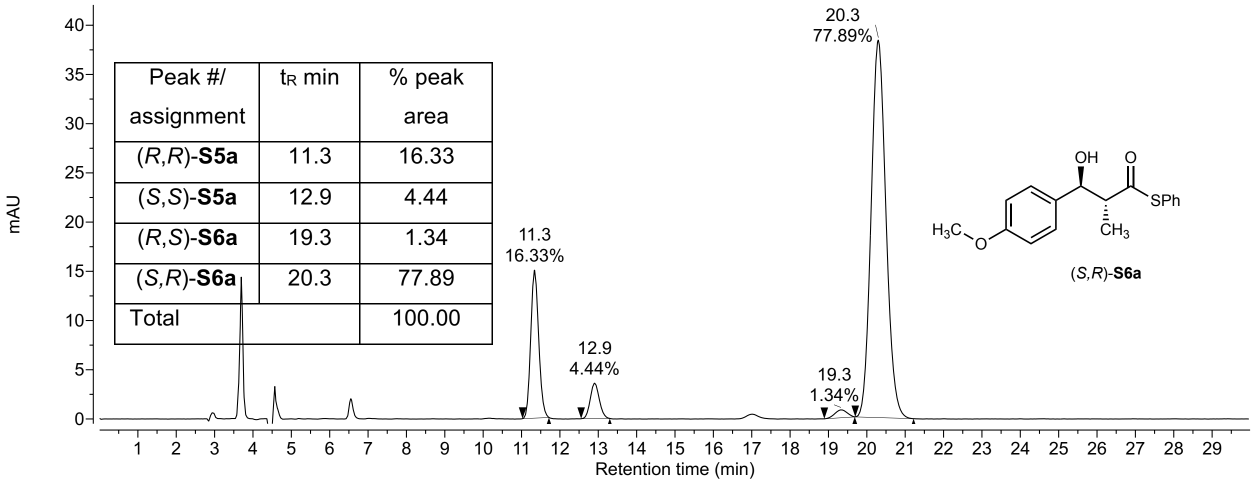

Peak #/ assignment	t_R min	% peak area
(*R*,*R*)-**S5a**	11.3	16.33
(*S*,*S*)-**S5a**	12.9	4.44
(*R*,*S*)-**S6a**	19.3	1.34
(*S*,*R*)-**S6a**	20.3	77.89
Total		100.00

HPLC (AD-H, *n*-hexane/EtOH=90:10, 1.0 mL/min, 298 K, 254 nm) for **S5b** and **S6b**:

Racemic aldol product:

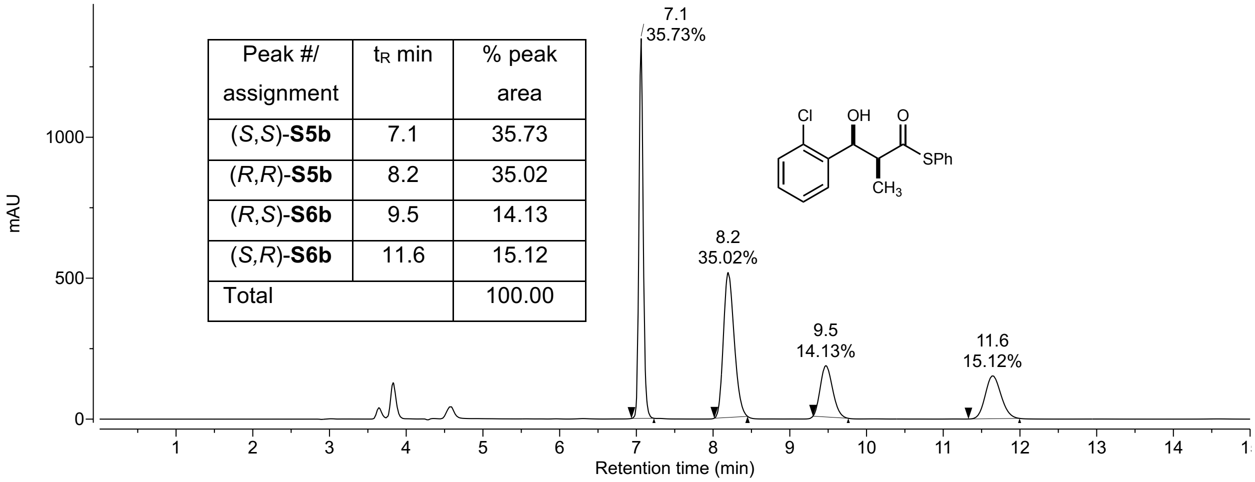

Peak #/ assignment	t_R min	% peak area
(*S*,*S*)-**S5b**	7.1	35.73
(*R*,*R*)-**S5b**	8.2	35.02
(*R*,*S*)-**S6b**	9.5	14.13
(*S*,*R*)-**S6b**	11.6	15.12
Total		100.00

Syn-aldol product:

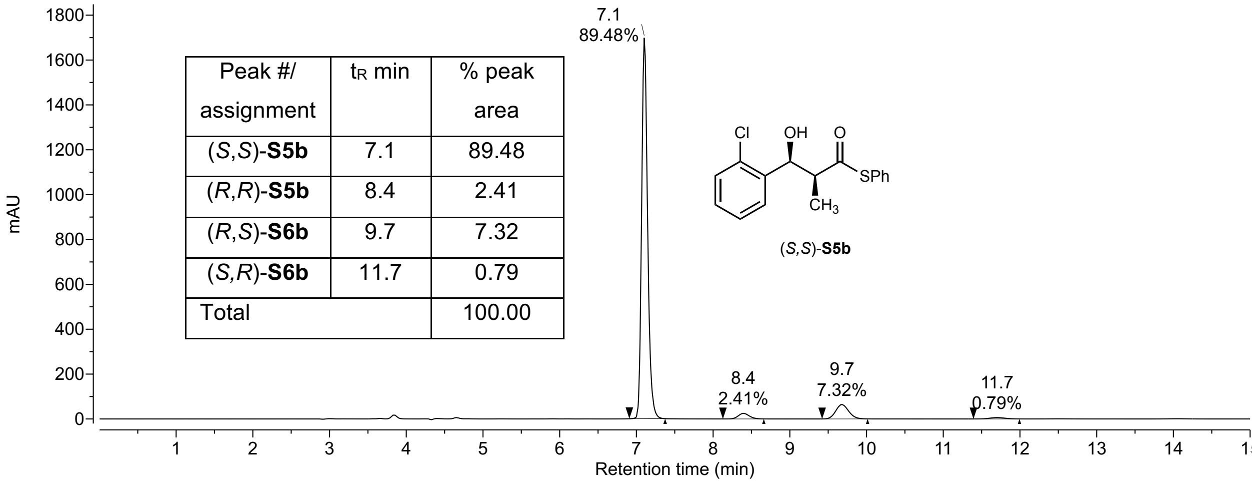

Peak #/ assignment	t_R min	% peak area
(*S*,*S*)-**S5b**	7.1	89.48
(*R*,*R*)-**S5b**	8.4	2.41
(*R*,*S*)-**S6b**	9.7	7.32
(*S*,*R*)-**S6b**	11.7	0.79
Total		100.00

Anti-aldol product:

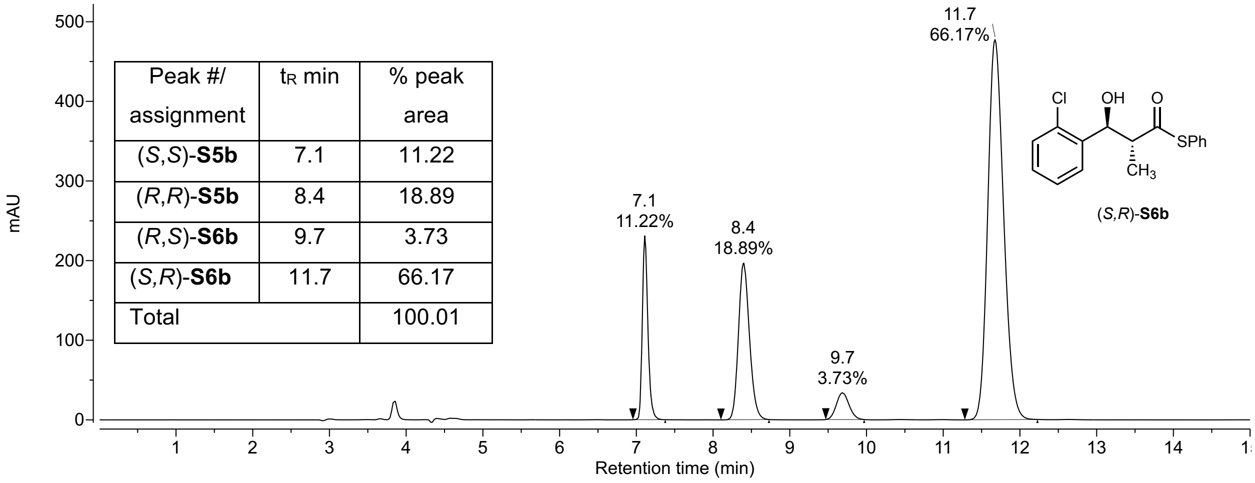

Peak #/ assignment	t_R min	% peak area
(*S*,*S*)-**S5b**	7.1	11.22
(*R*,*R*)-**S5b**	8.4	18.89
(*R*,*S*)-**S6b**	9.7	3.73
(*S*,*R*)-**S6b**	11.7	66.17
Total		100.01

HPLC (AD-H, *n*-hexane/^{i}PrOH=90:10, 1.0 mL/min, 298 K, 254 nm) for **S5c** and **S6c**:

Racemic aldol product:

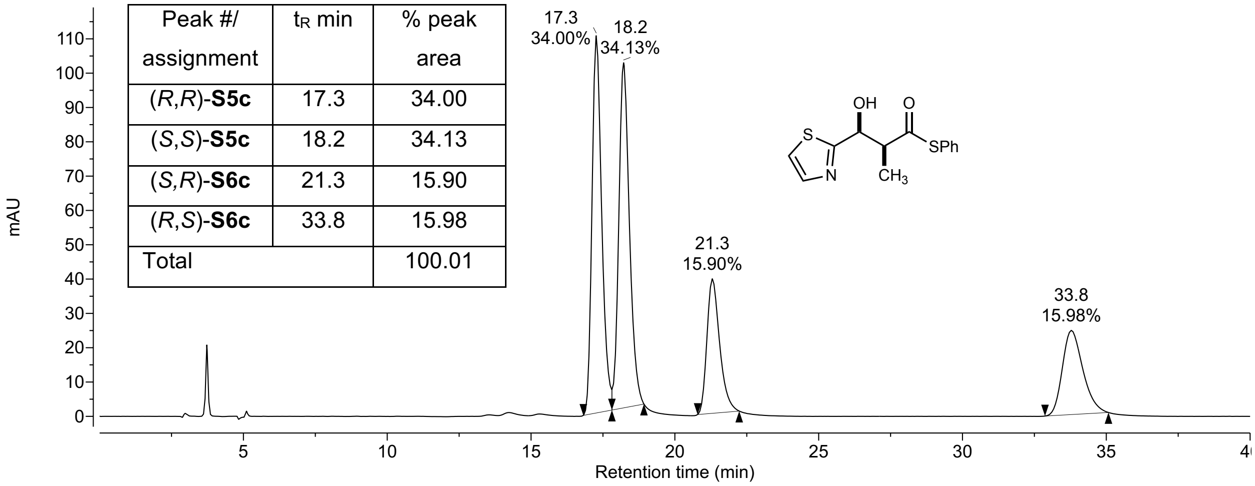

Peak #/ assignment	t_R min	% peak area
(*R*,*R*)-**S5c**	17.3	34.00
(*S*,*S*)-**S5c**	18.2	34.13
(*S*,*R*)-**S6c**	21.3	15.90
(*R*,*S*)-**S6c**	33.8	15.98
Total		100.01

Syn-aldol product:

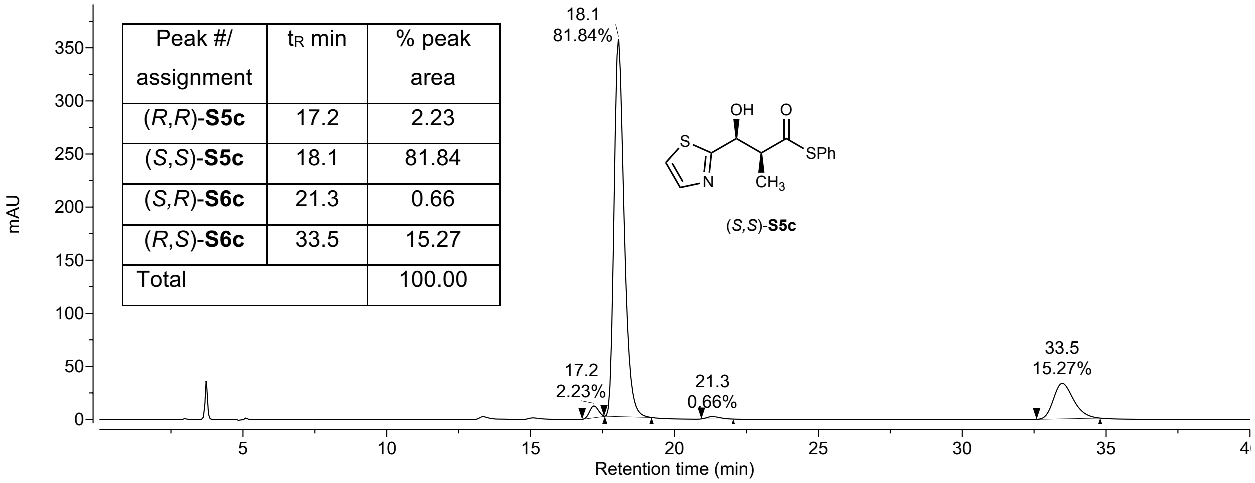

Peak #/ assignment	t_R min	% peak area
(*R*,*R*)-**S5c**	17.2	2.23
(*S*,*S*)-**S5c**	18.1	81.84
(*S*,*R*)-**S6c**	21.3	0.66
(*R*,*S*)-**S6c**	33.5	15.27
Total		100.00

Anti-aldol product:

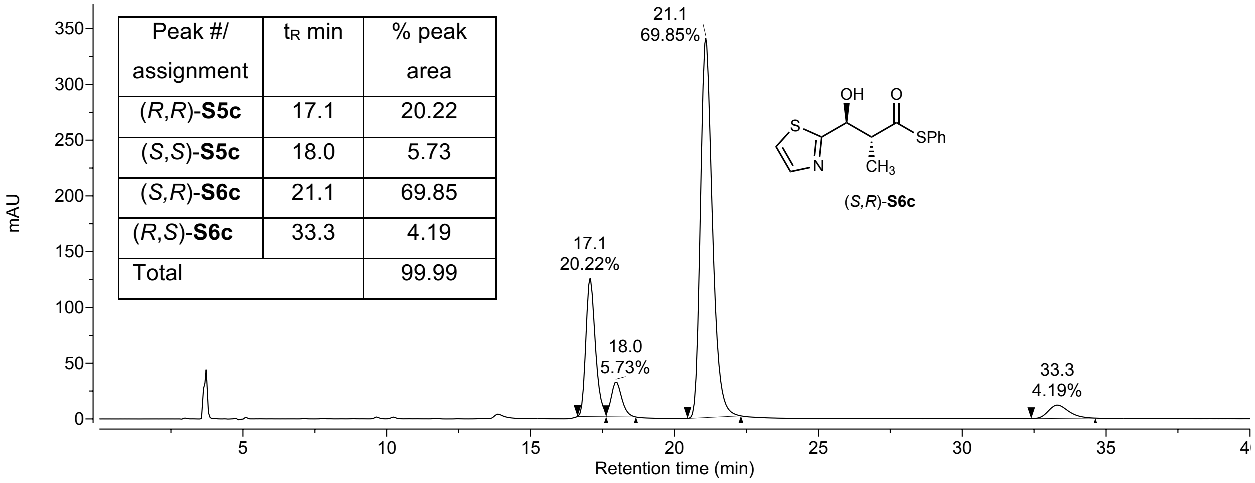

Peak #/ assignment	t_R min	% peak area
(*R*,*R*)-**S5c**	17.1	20.22
(*S*,*S*)-**S5c**	18.0	5.73
(*S*,*R*)-**S6c**	21.1	69.85
(*R*,*S*)-**S6c**	33.3	4.19
Total		99.99

HPLC (AD-H, *n*-hexane/^{i}PrOH=92:8, 1.0 mL/min, 298 K, 254 nm) for **S5d** and **S6d**:

Racemic aldol product:

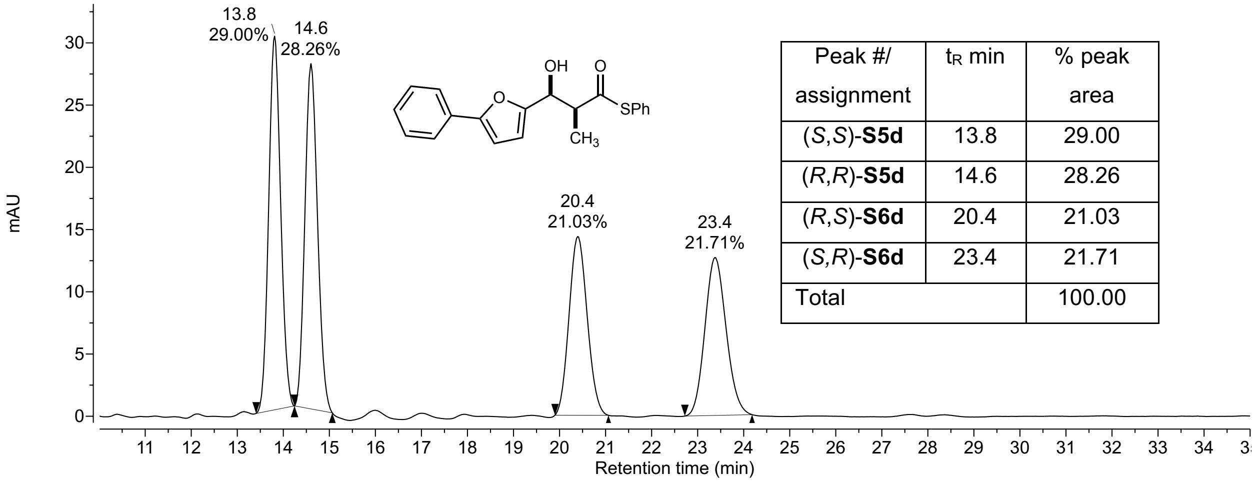

Peak #/ assignment	t_R min	% peak area
(*S*,*S*)-**S5d**	13.8	29.00
(*R*,*R*)-**S5d**	14.6	28.26
(*R*,*S*)-**S6d**	20.4	21.03
(*S*,*R*)-**S6d**	23.4	21.71
Total		100.00

Syn-aldol product:

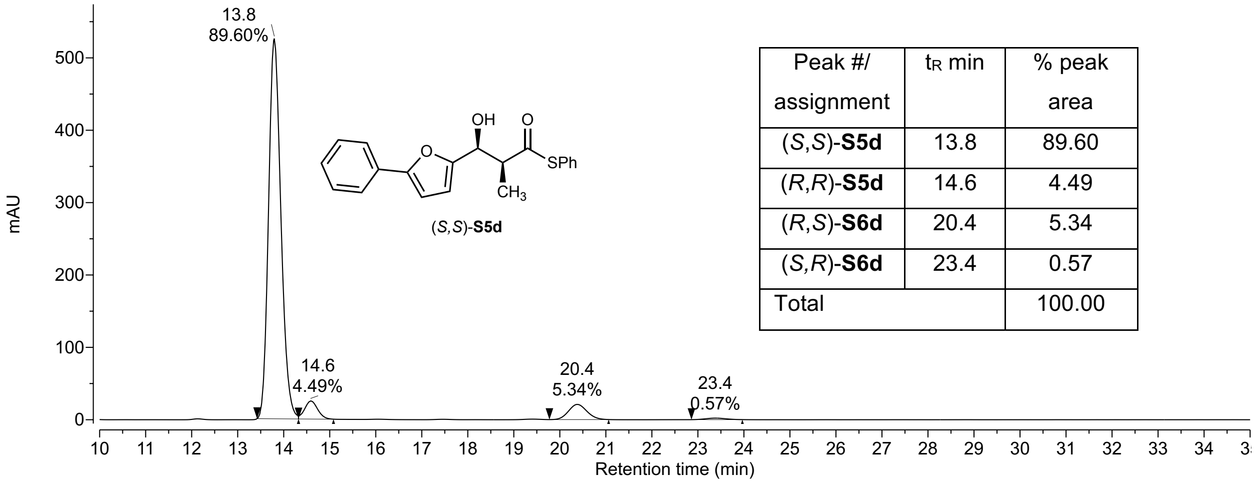

Peak #/ assignment	t_R min	% peak area
(*S*,*S*)-**S5d**	13.8	89.60
(*R*,*R*)-**S5d**	14.6	4.49
(*R*,*S*)-**S6d**	20.4	5.34
(*S*,*R*)-**S6d**	23.4	0.57
Total		100.00

Anti-aldol product:

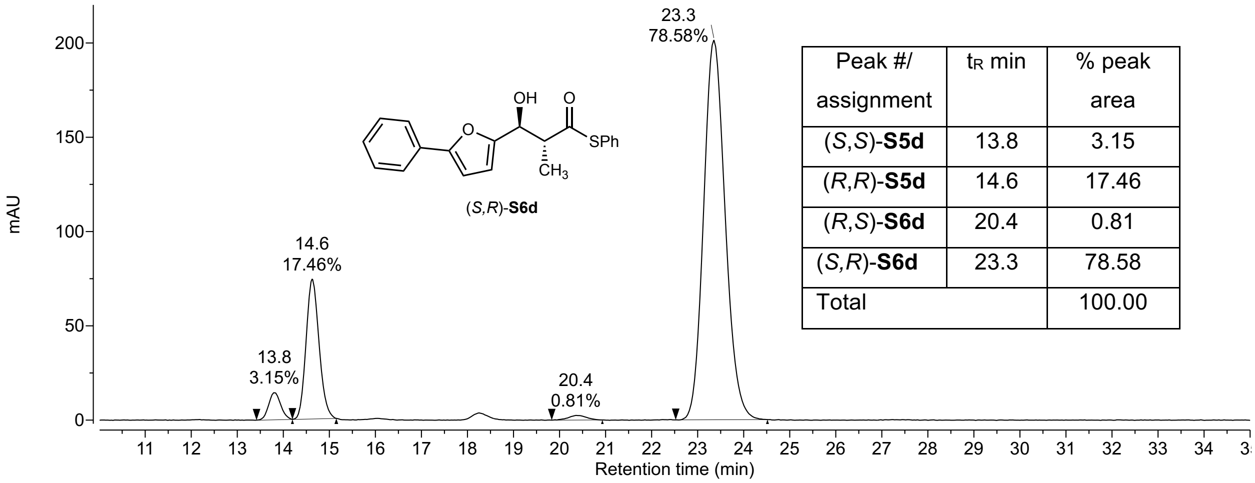

Peak #/ assignment	t_R min	% peak area
(*S*,*S*)-**S5d**	13.8	3.15
(*R*,*R*)-**S5d**	14.6	17.46
(*R*,*S*)-**S6d**	20.4	0.81
(*S*,*R*)-**S6d**	23.3	78.58
Total		100.00

HPLC (AD-H, *n*-hexane/EtOH=97:3, 1.0 mL/min, 298 K, 254 nm) for **S5e** and **S6e**:

Racemic aldol product:

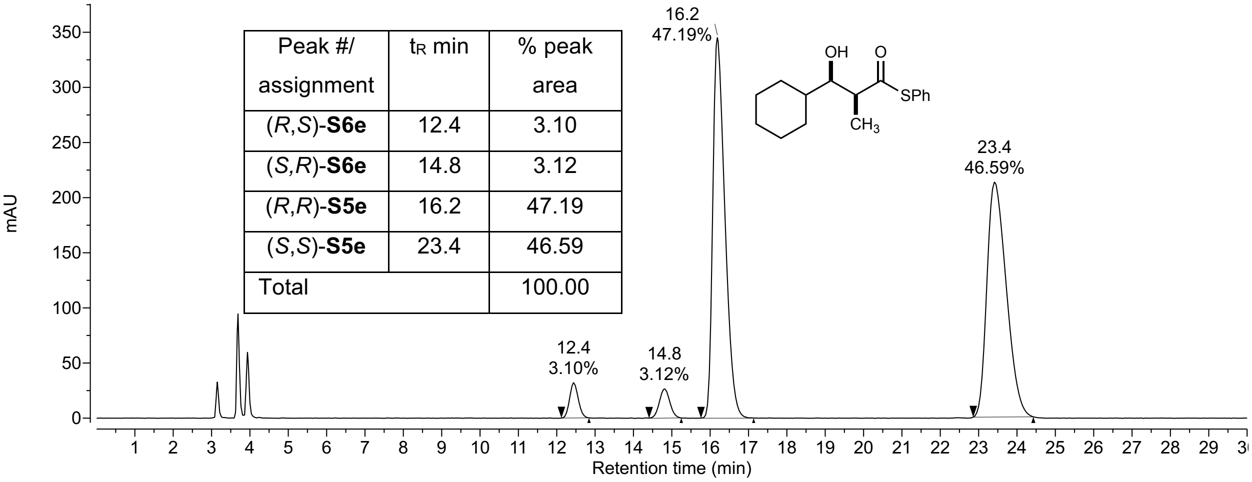

Peak #/ assignment	t_R min	% peak area
(*R*,*S*)-**S6e**	12.4	3.10
(*S*,*R*)-**S6e**	14.8	3.12
(*R*,*R*)-**S5e**	16.2	47.19
(*S*,*S*)-**S5e**	23.4	46.59
Total		100.00

Syn-aldol product:

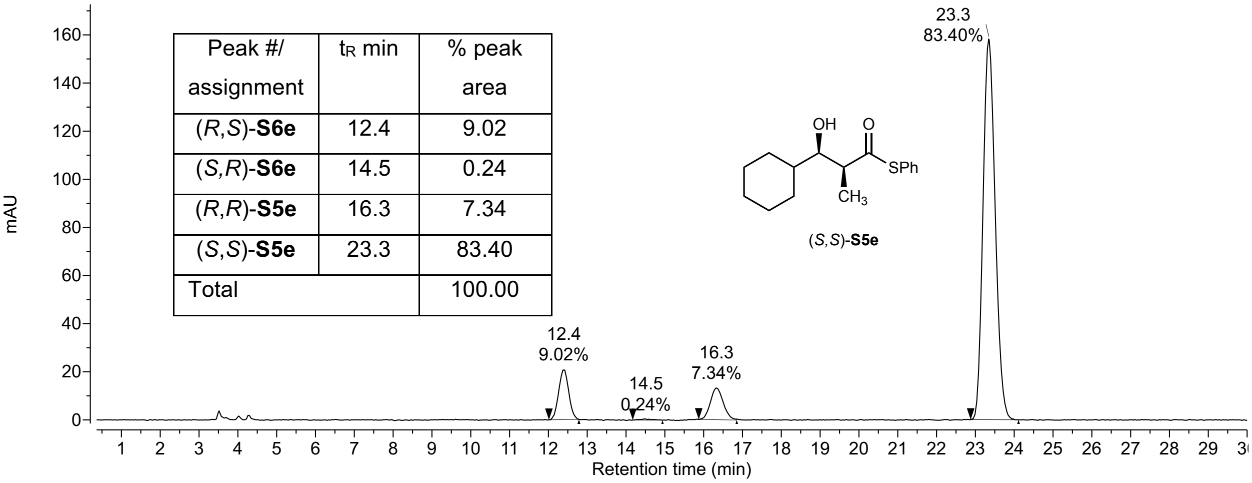

Peak #/ assignment	t_R min	% peak area
(*R*,*S*)-**S6e**	12.4	9.02
(*S*,*R*)-**S6e**	14.5	0.24
(*R*,*R*)-**S5e**	16.3	7.34
(*S*,*S*)-**S5e**	23.3	83.40
Total		100.00

Anti-aldol product:

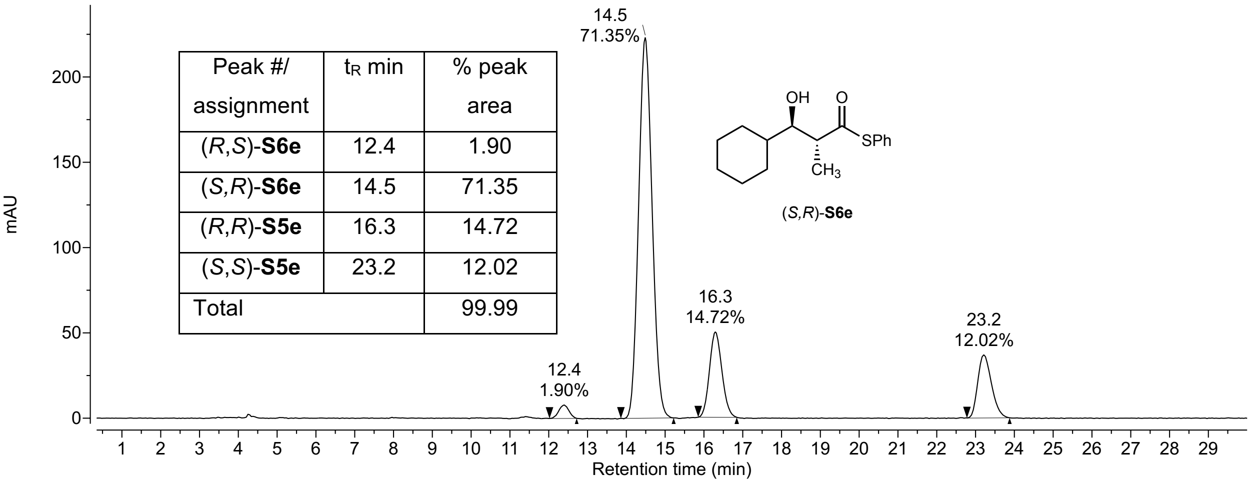

Peak #/ assignment	t_R min	% peak area
(*R*,*S*)-**S6e**	12.4	1.90
(*S*,*R*)-**S6e**	14.5	71.35
(*R*,*R*)-**S5e**	16.3	14.72
(*S*,*S*)-**S5e**	23.2	12.02
Total		99.99

HPLC (AD-H, *n*-hexane/^{i}PrOH=90:10, 1.0 mL/min, 298 K, 254 nm) for **S5f** and **S6f**:

Racemic aldol product:

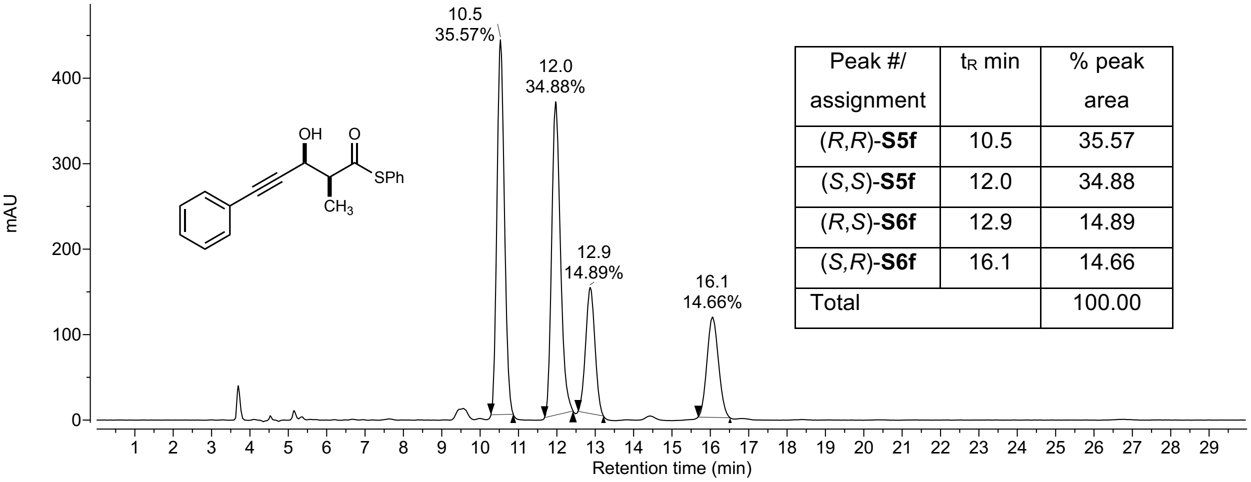

Peak #/ assignment	t_R min	% peak area
(*R*,*R*)-**S5f**	10.5	35.57
(*S*,*S*)-**S5f**	12.0	34.88
(*R*,*S*)-**S6f**	12.9	14.89
(*S*,*R*)-**S6f**	16.1	14.66
Total		100.00

Syn-aldol product:

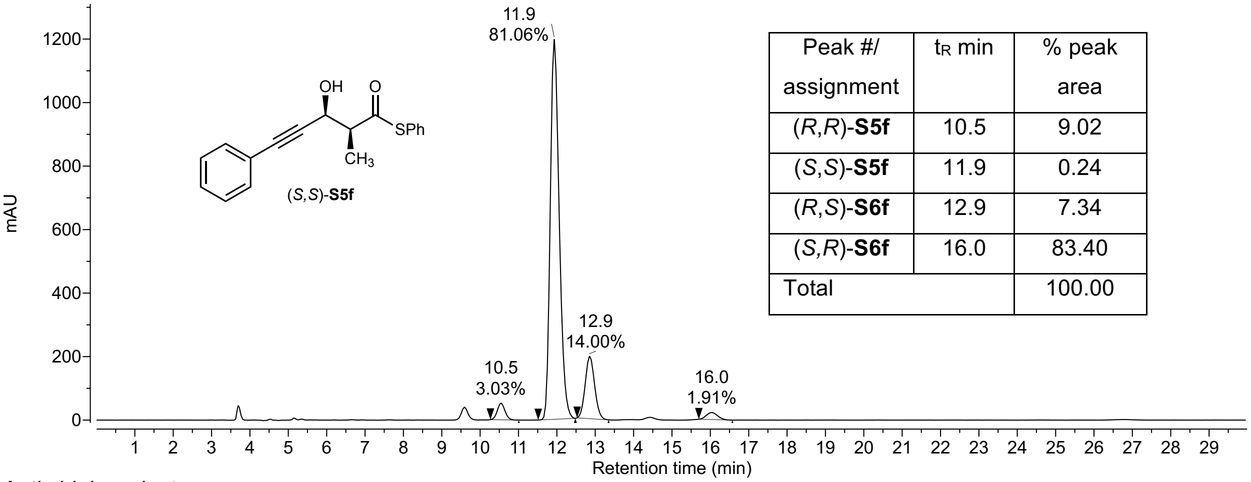

Peak #/ assignment	t_R min	% peak area
(*R*,*R*)-**S5f**	10.5	9.02
(*S*,*S*)-**S5f**	11.9	0.24
(*R*,*S*)-**S6f**	12.9	7.34
(*S*,*R*)-**S6f**	16.0	83.40
Total		100.00

Anti-aldol product:

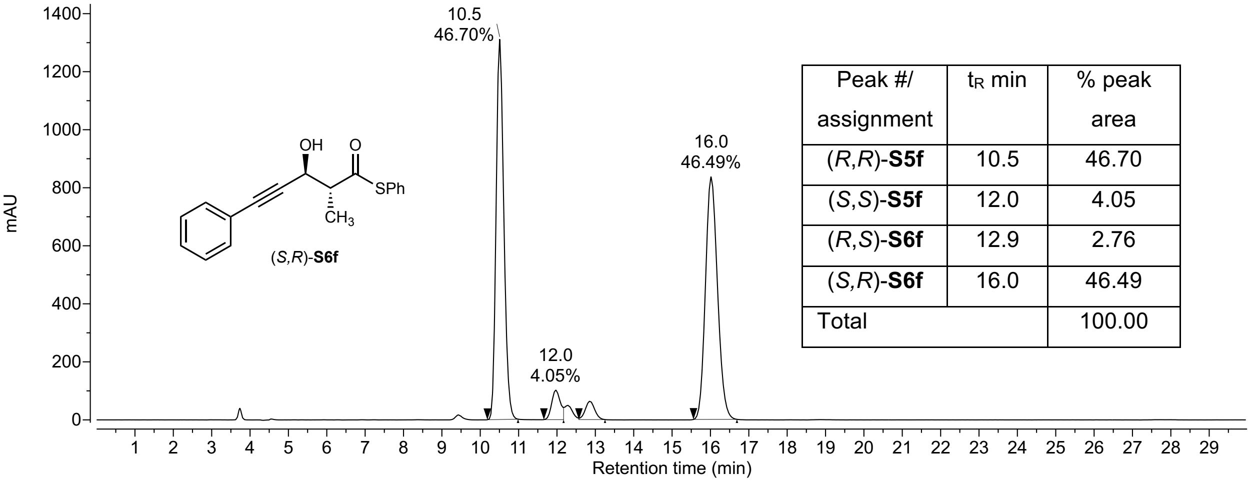

Peak #/ assignment	t_R min	% peak area
(*R*,*R*)-**S5f**	10.5	46.70
(*S*,*S*)-**S5f**	12.0	4.05
(*R*,*S*)-**S6f**	12.9	2.76
(*S*,*R*)-**S6f**	16.0	46.49
Total		100.00

HPLC (AD-H, *n*-hexane/EtOH=98:2, 1.0 mL/min, 298 K, 254 nm) for **S5g** and **S6g**:

Racemic aldol product:

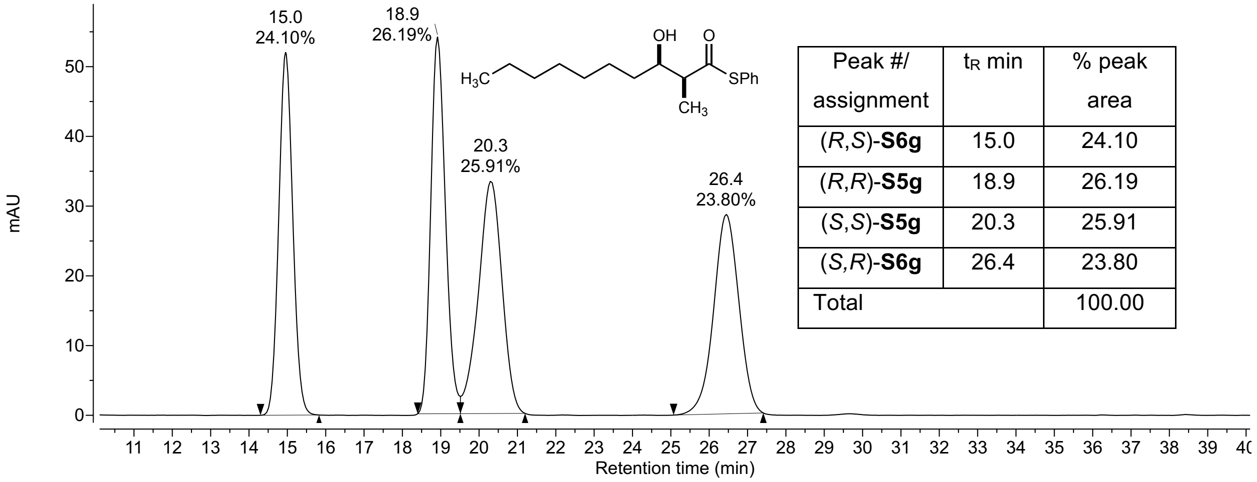

Peak #/ assignment	t_R min	% peak area
(*R*,*S*)-**S6g**	15.0	24.10
(*R*,*R*)-**S5g**	18.9	26.19
(*S*,*S*)-**S5g**	20.3	25.91
(*S*,*R*)-**S6g**	26.4	23.80
Total		100.00

Syn-aldol product:

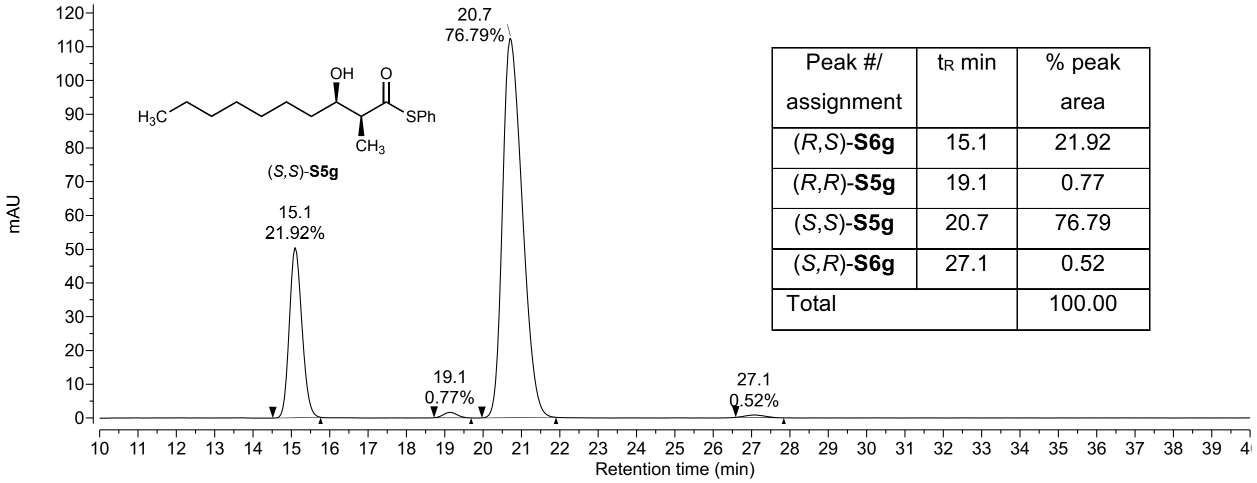

Peak #/ assignment	t_R min	% peak area
(*R*,*S*)-**S6g**	15.1	21.92
(*R*,*R*)-**S5g**	19.1	0.77
(*S*,*S*)-**S5g**	20.7	76.79
(*S*,*R*)-**S6g**	27.1	0.52
Total		100.00

Anti-aldol product:

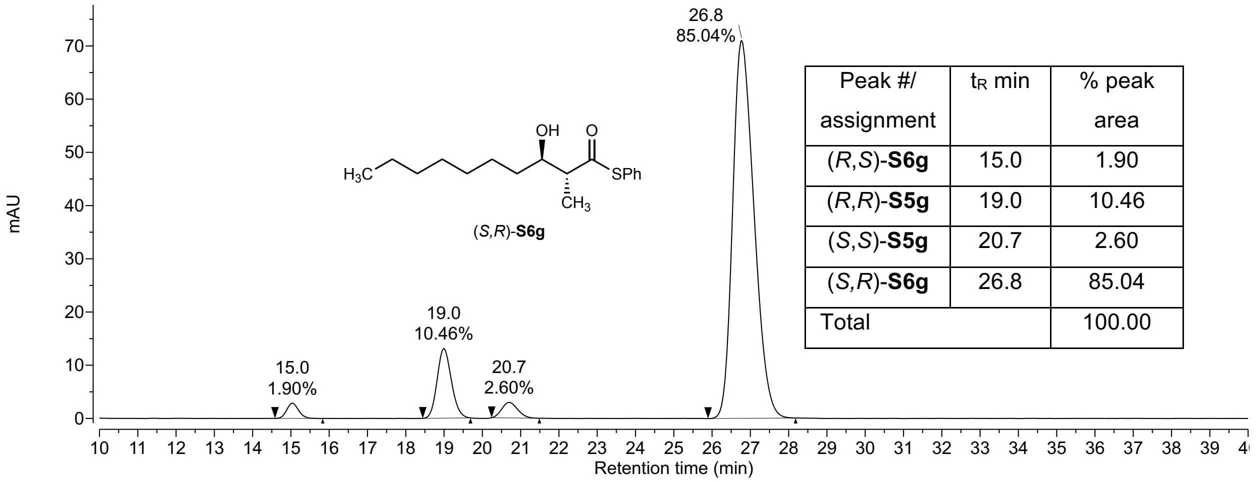

Peak #/ assignment	t_R min	% peak area
(*R*,*S*)-**S6g**	15.0	1.90
(*R*,*R*)-**S5g**	19.0	10.46
(*S*,*S*)-**S5g**	20.7	2.60
(*S*,*R*)-**S6g**	26.8	85.04
Total		100.00

HPLC (AD-H, *n*-hexane/^{i}PrOH=93:7, 1.0 mL/min, 298 K, 254 nm) for **S7h** and **S7h**:

Racemic aldol product:

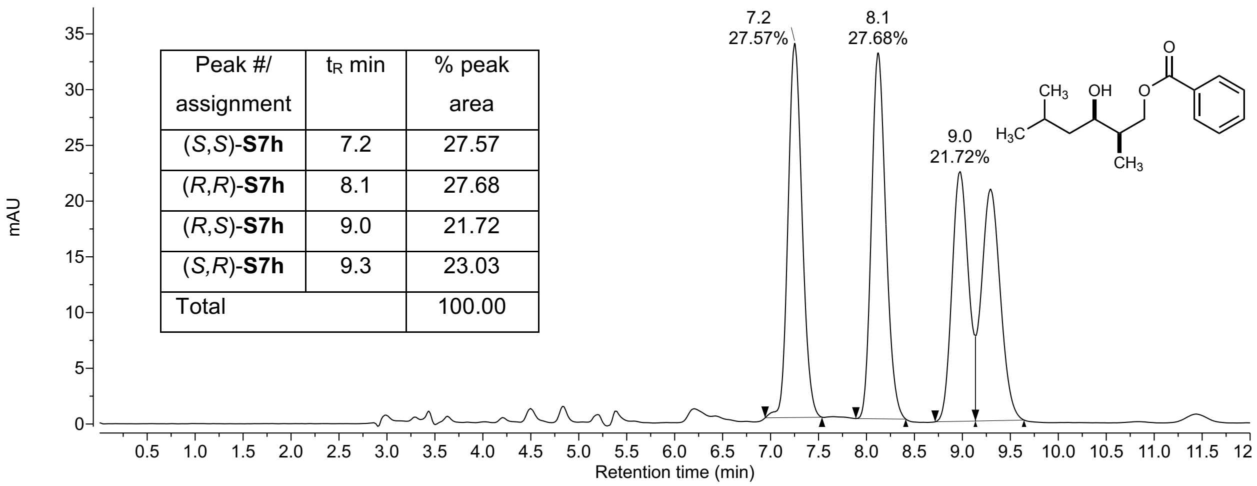

Peak #/ assignment	t_R min	% peak area
(*S*,*S*)-**S7h**	7.2	27.57
(*R*,*R*)-**S7h**	8.1	27.68
(*R*,*S*)-**S7h**	9.0	21.72
(*S*,*R*)-**S7h**	9.3	23.03
Total		100.00

Syn-aldol product:

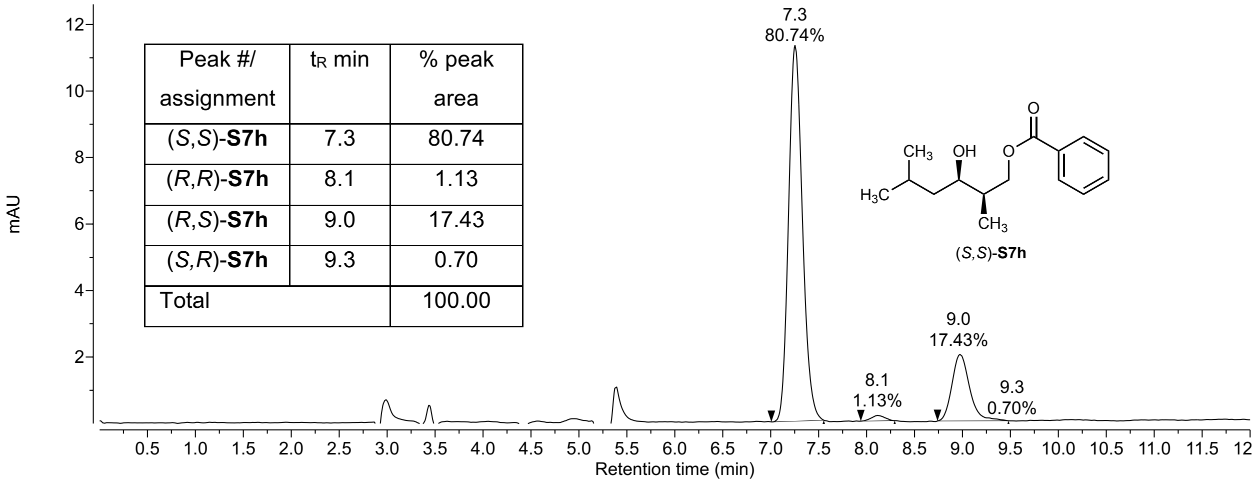

Peak #/ assignment	t_R min	% peak area
(*S*,*S*)-**S7h**	7.3	80.74
(*R*,*R*)-**S7h**	8.1	1.13
(*R*,*S*)-**S7h**	9.0	17.43
(*S*,*R*)-**S7h**	9.3	0.70
Total		100.00

Anti-aldol product:

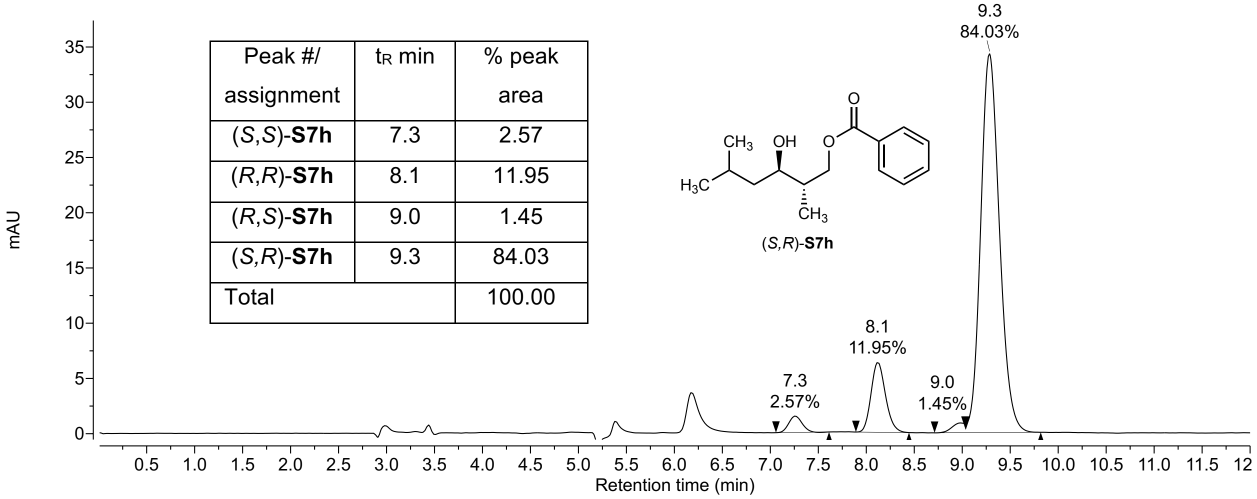

Peak #/ assignment	t_R min	% peak area
(*S*,*S*)-**S7h**	7.3	2.57
(*R*,*R*)-**S7h**	8.1	11.95
(*R*,*S*)-**S7h**	9.0	1.45
(*S*,*R*)-**S7h**	9.3	84.03
Total		100.00

HPLC (AD-H, *n*-hexane/^{i}PrOH=90:10, 1.0 mL/min, 298 K, 254 nm) for **7a**:

Racemic aldol product:

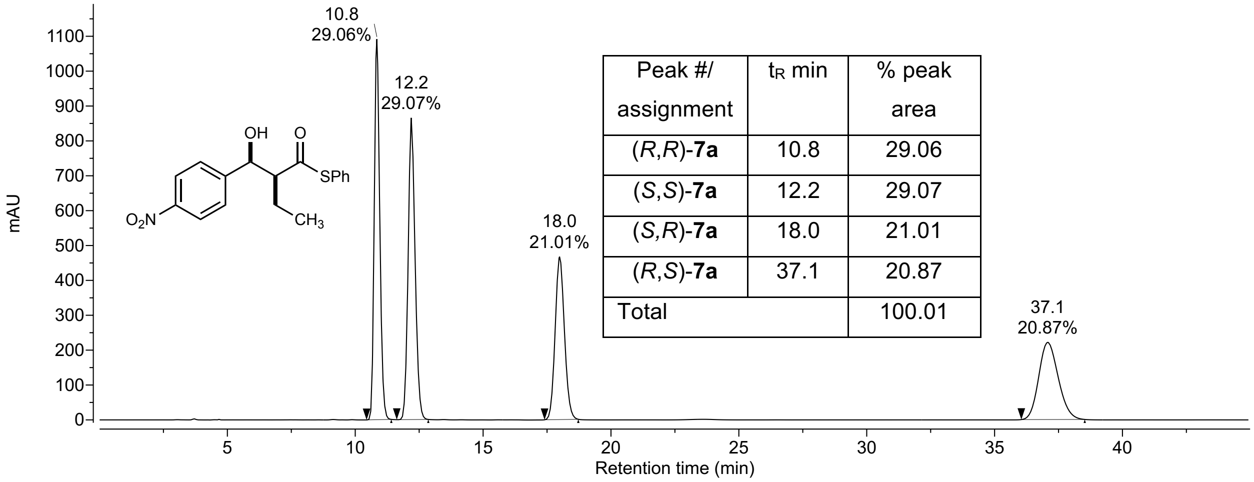

Peak #/ assignment	t_R min	% peak area
(*R*,*R*)-**7a**	10.8	29.06
(*S*,*S*)-**7a**	12.2	29.07
(*S*,*R*)-**7a**	18.0	21.01
(*R*,*S*)-**7a**	37.1	20.87
Total		100.01

Syn-aldol product:

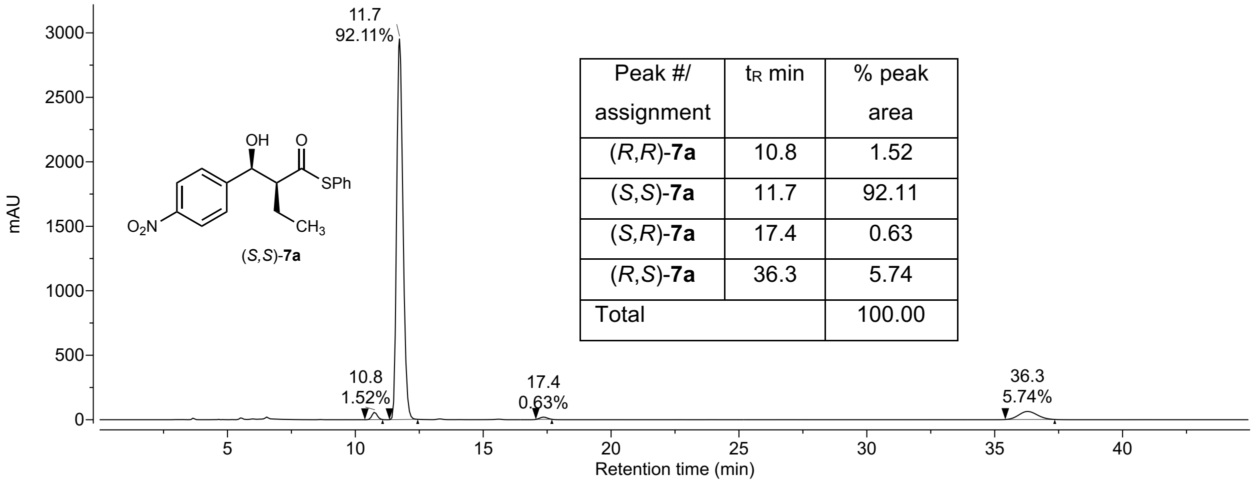

Peak #/ assignment	t_R min	% peak area
(*R*,*R*)-**7a**	10.8	1.52
(*S*,*S*)-**7a**	11.7	92.11
(*S*,*R*)-**7a**	17.4	0.63
(*R*,*S*)-**7a**	36.3	5.74
Total		100.00

HPLC (AD-H, *n*-hexane/^{i}PrOH=90:10, 1.0 mL/min, 298 K, 254 nm) for **7b**:

Racemic aldol product:

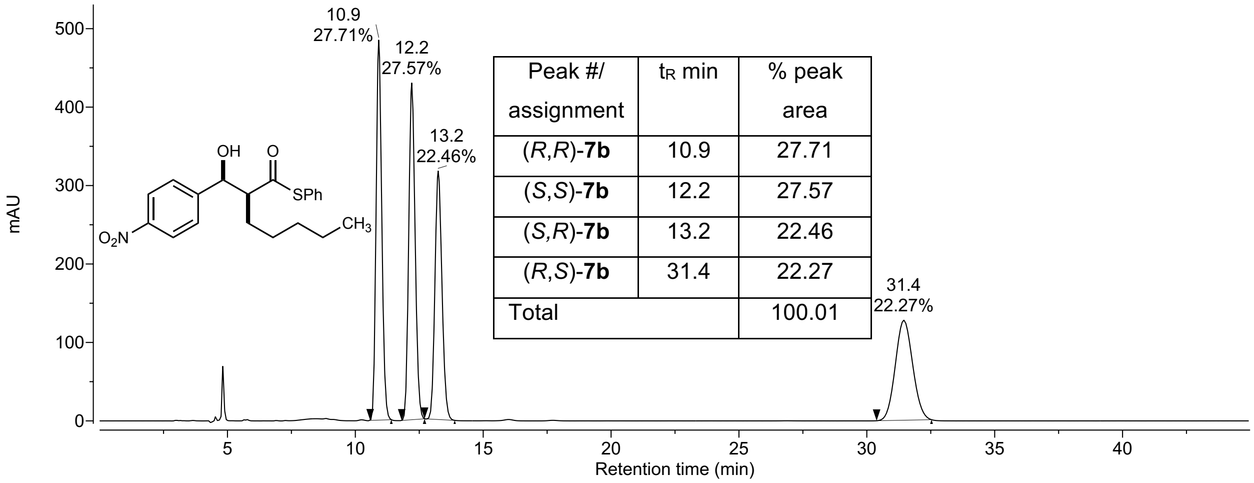

Peak #/ assignment	t_R min	% peak area
(*R*,*R*)-**7b**	10.9	27.71
(*S*,*S*)-**7b**	12.2	27.57
(*S*,*R*)-**7b**	13.2	22.46
(*R*,*S*)-**7b**	31.4	22.27
Total		100.01

Syn-aldol product:

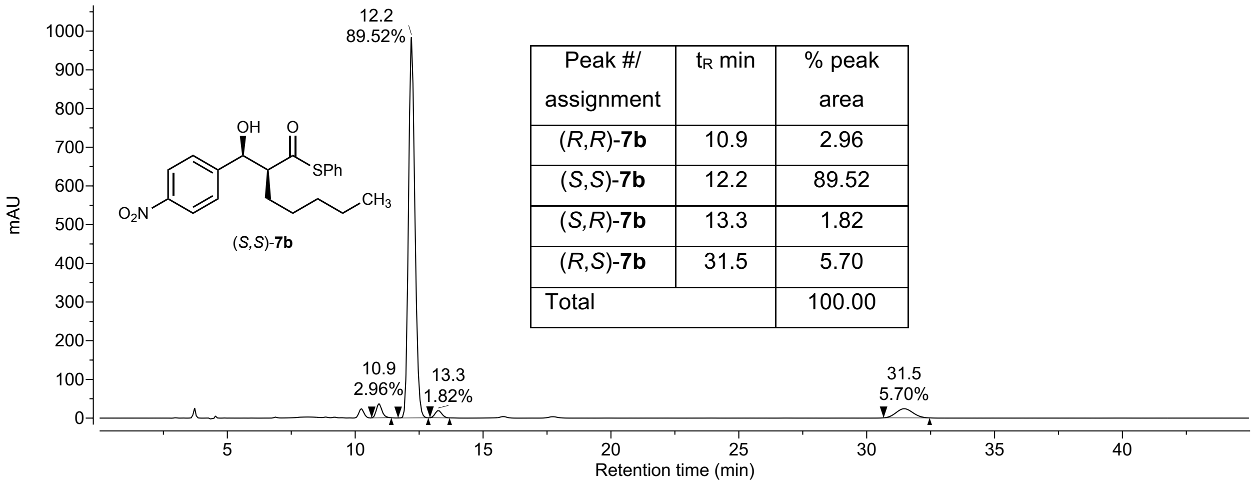

Peak #/ assignment	t_R min	% peak area
(*R*,*R*)-**7b**	10.9	2.96
(*S*,*S*)-**7b**	12.2	89.52
(*S*,*R*)-**7b**	13.3	1.82
(*R*,*S*)-**7b**	31.5	5.70
Total		100.00

HPLC (AD-H, *n*-hexane/^{i}PrOH=88:12, 1.0 mL/min, 298 K, 254 nm) for **7c**:

Racemic aldol product:

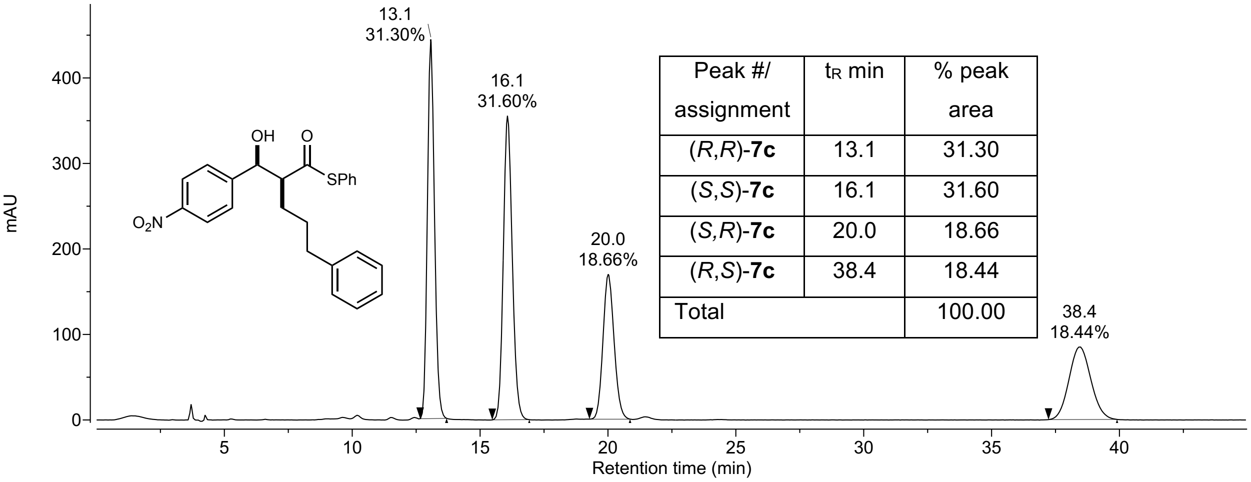

Peak #/ assignment	t_R min	% peak area
(*R*,*R*)-**7c**	13.1	31.30
(*S*,*S*)-**7c**	16.1	31.60
(*S*,*R*)-**7c**	20.0	18.66
(*R*,*S*)-**7c**	38.4	18.44
Total		100.00

Syn-aldol product:

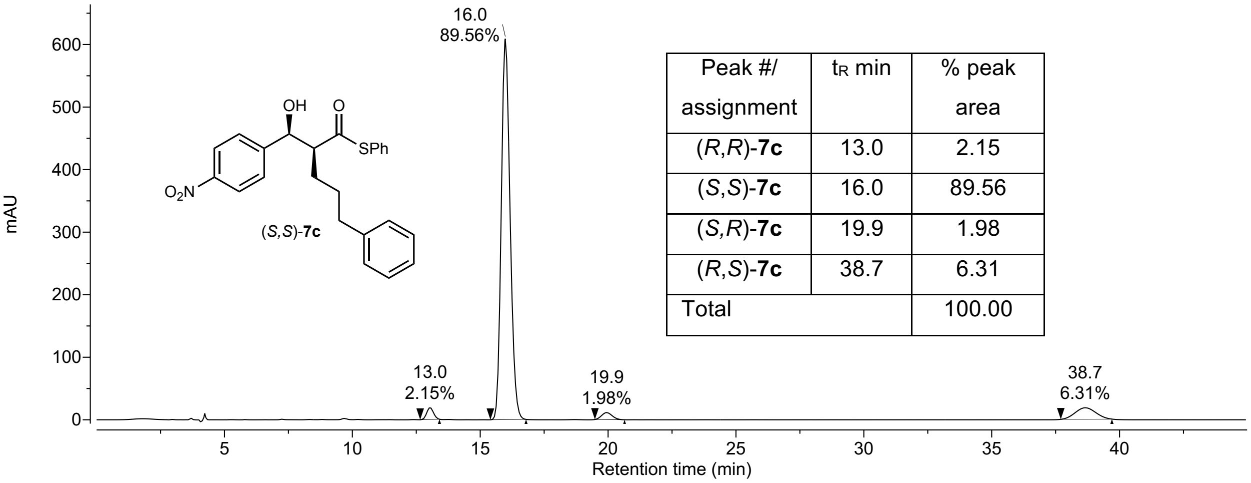

Peak #/ assignment	t_R min	% peak area
(*R*,*R*)-**7c**	13.0	2.15
(*S*,*S*)-**7c**	16.0	89.56
(*S*,*R*)-**7c**	19.9	1.98
(*R*,*S*)-**7c**	38.7	6.31
Total		100.00

HPLC (AD-H, *n*-hexane/^{i}PrOH=88:12, 1.0 mL/min, 298 K, 254 nm) for **7d**:

Racemic aldol product:

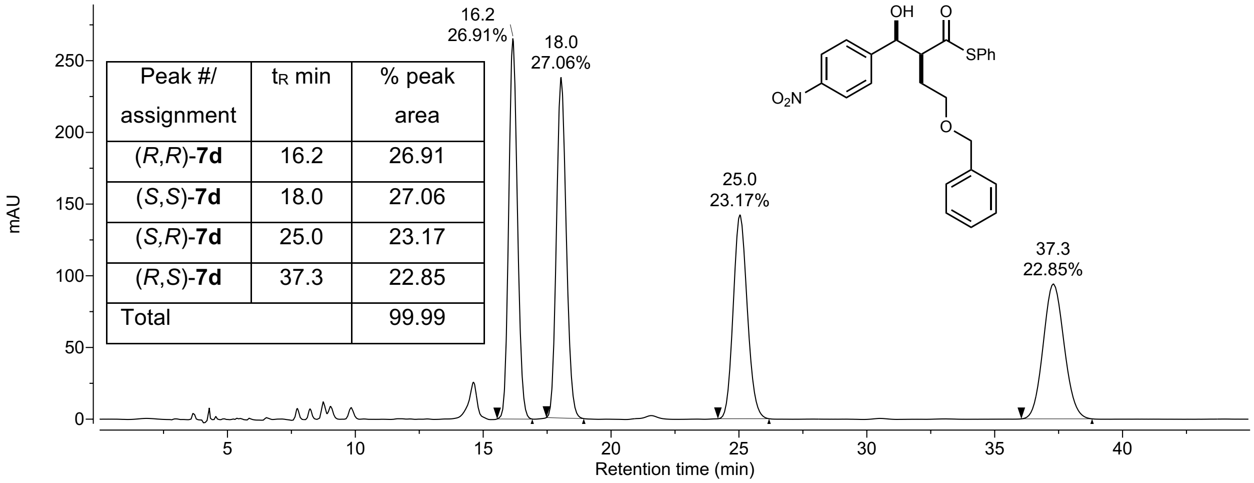

Peak #/ assignment	t_R min	% peak area
(*R*,*R*)-**7d**	16.2	26.91
(*S*,*S*)-**7d**	18.0	27.06
(*S*,*R*)-**7d**	25.0	23.17
(*R*,*S*)-**7d**	37.3	22.85
Total		99.99

Syn-aldol product:

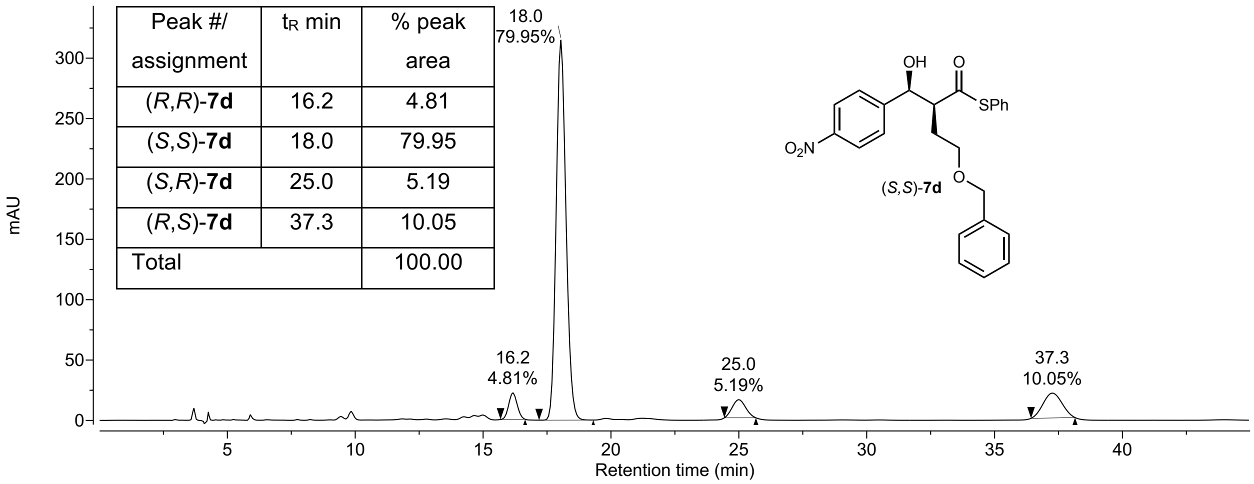

Peak #/ assignment	t_R min	% peak area
(*R*,*R*)-**7d**	16.2	4.81
(*S*,*S*)-**7d**	18.0	79.95
(*S*,*R*)-**7d**	25.0	5.19
(*R*,*S*)-**7d**	37.3	10.05
Total		100.00

HPLC (AD-H, *n*-hexane/^{i}PrOH=90:10, 1.0 mL/min, 298 K, 254 nm) for **7e**:

Racemic aldol product:

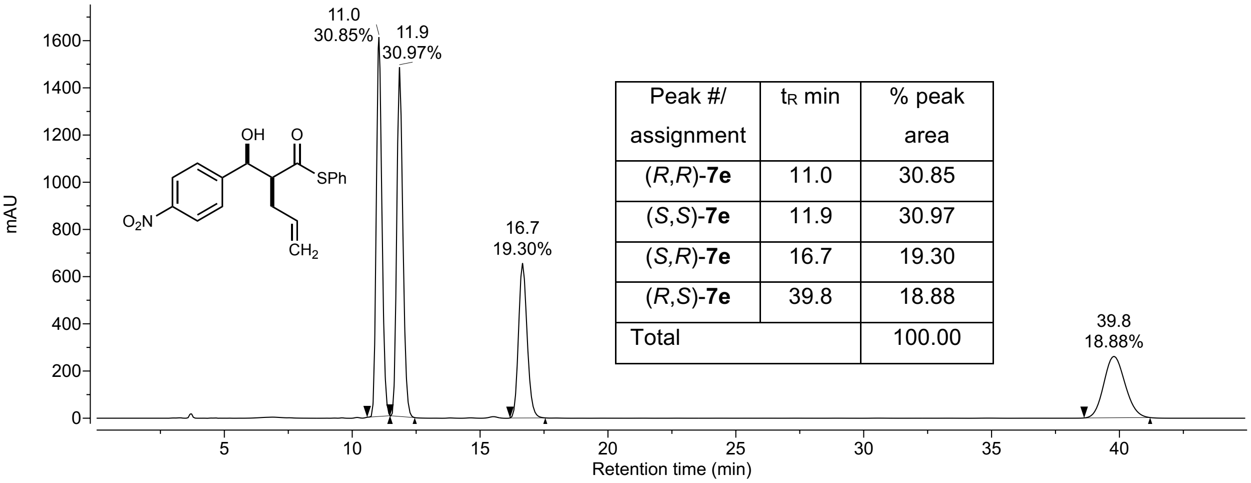

Peak #/ assignment	tR min	% peak area
(*R*,*R*)-**7e**	11.0	30.85
(*S*,*S*)-**7e**	11.9	30.97
(*S*,*R*)-**7e**	16.7	19.30
(*R*,*S*)-**7e**	39.8	18.88
Total		100.00

Syn-aldol product:

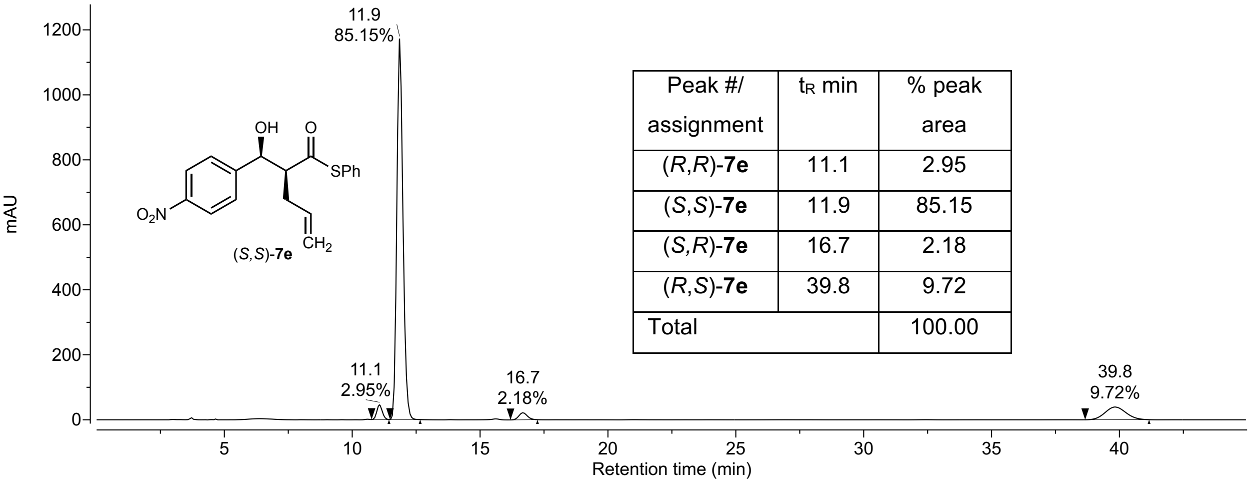

Peak #/ assignment	tR min	% peak area
(*R*,*R*)-**7e**	11.1	2.95
(*S*,*S*)-**7e**	11.9	85.15
(*S*,*R*)-**7e**	16.7	2.18
(*R*,*S*)-**7e**	39.8	9.72
Total		100.00

HPLC (AD-H, *n*-hexane/EtOH=85:15, 1.0 mL/min, 298 K, 254 nm) for **7f**:

Racemic aldol product:

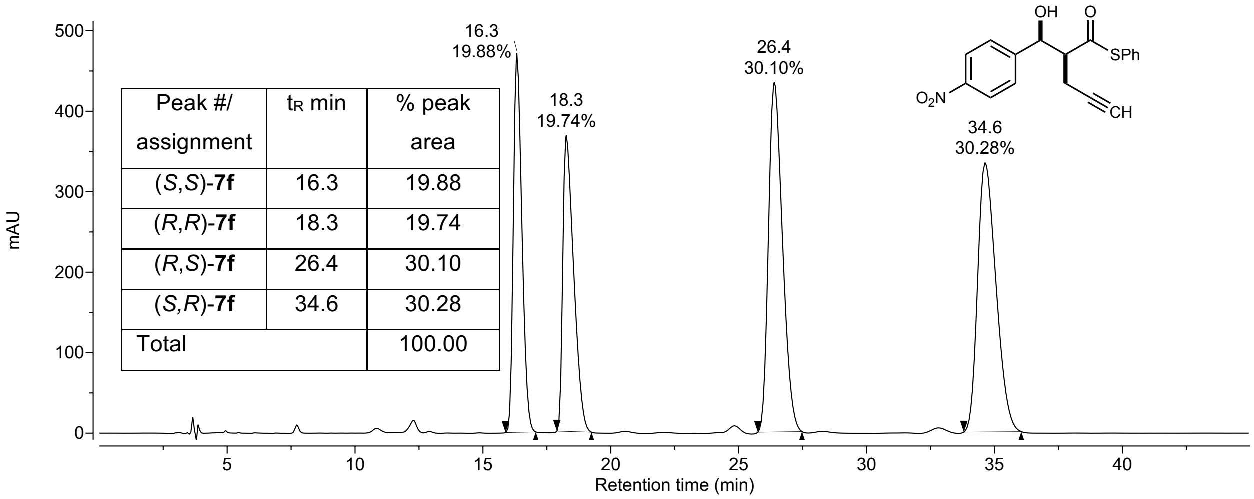

Peak #/ assignment	t_R min	% peak area
(*S*,*S*)-**7f**	16.3	19.88
(*R*,*R*)-**7f**	18.3	19.74
(*R*,*S*)-**7f**	26.4	30.10
(*S*,*R*)-**7f**	34.6	30.28
Total		100.00

Syn-aldol product:

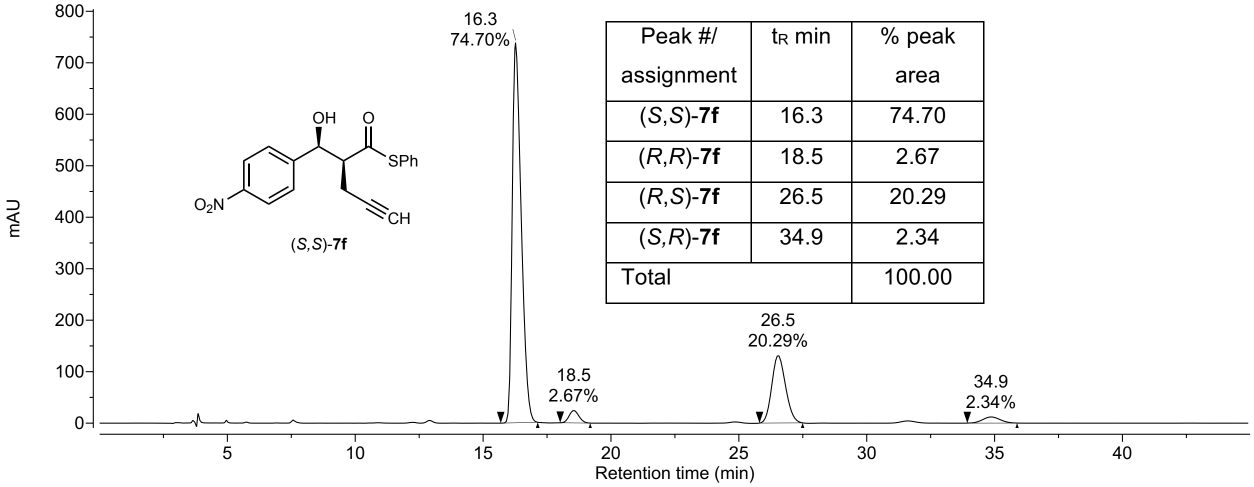

Peak #/ assignment	t_R min	% peak area
(*S*,*S*)-**7f**	16.3	74.70
(*R*,*R*)-**7f**	18.5	2.67
(*R*,*S*)-**7f**	26.5	20.29
(*S*,*R*)-**7f**	34.9	2.34
Total		100.00

HPLC (AD-H, *n*-hexane/EtOH=88:12, 1.0 mL/min, 298 K, 254 nm) for **7g**:

Racemic aldol product:

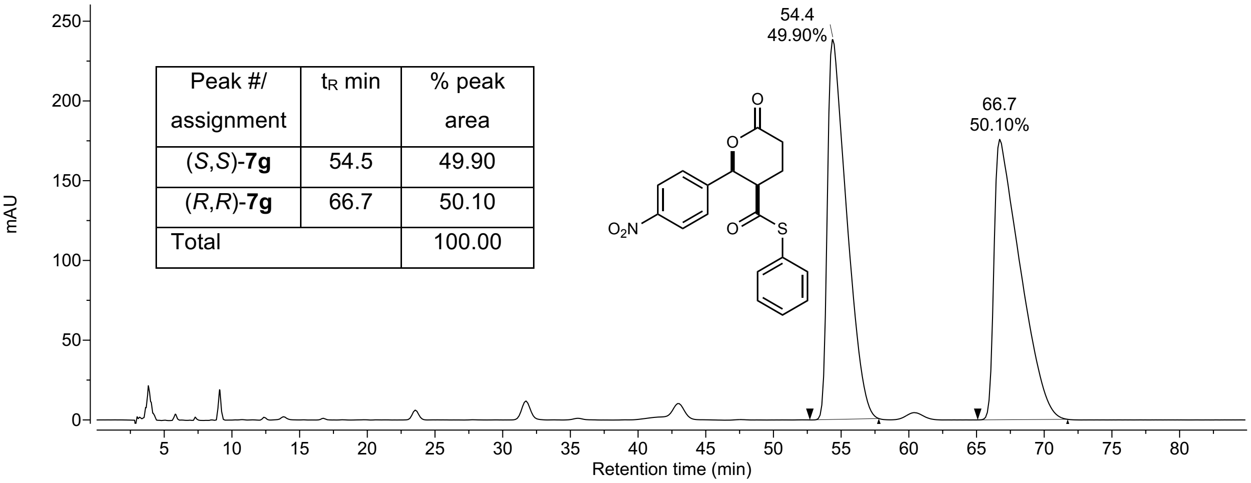

Peak #/ assignment	t_R min	% peak area
(*S*,*S*)-**7g**	54.5	49.90
(*R*,*R*)-**7g**	66.7	50.10
Total		100.00

Syn-aldol product:

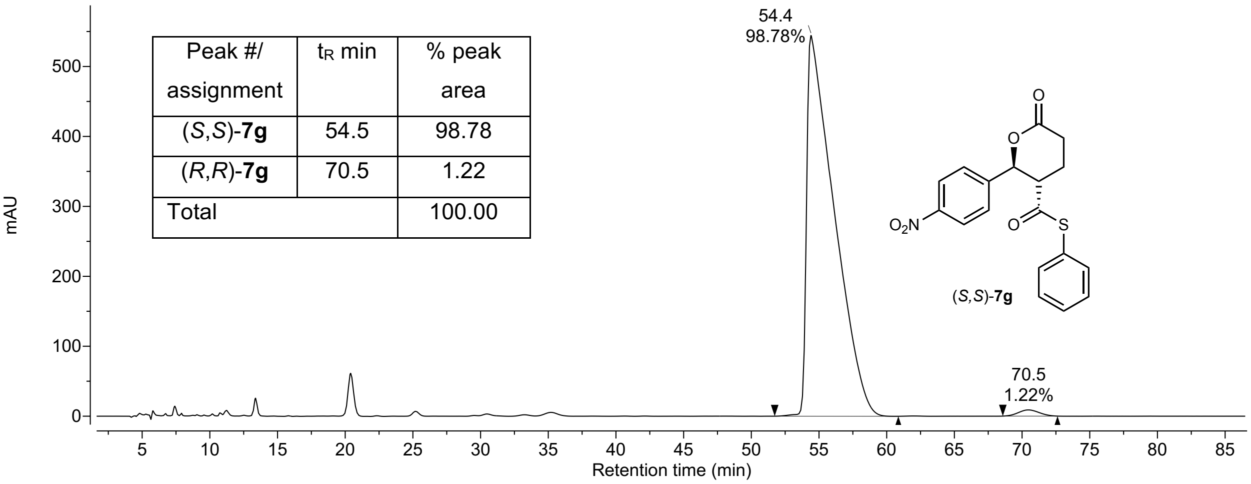

Peak #/ assignment	t_R min	% peak area
(*S*,*S*)-**7g**	54.5	98.78
(*R*,*R*)-**7g**	70.5	1.22
Total		100.00

Catalog of nuclear magnetic resonance spectra:

^1H NMR, 500 MHz, $CDCl_3$, (*S*,*S*)-**5a**

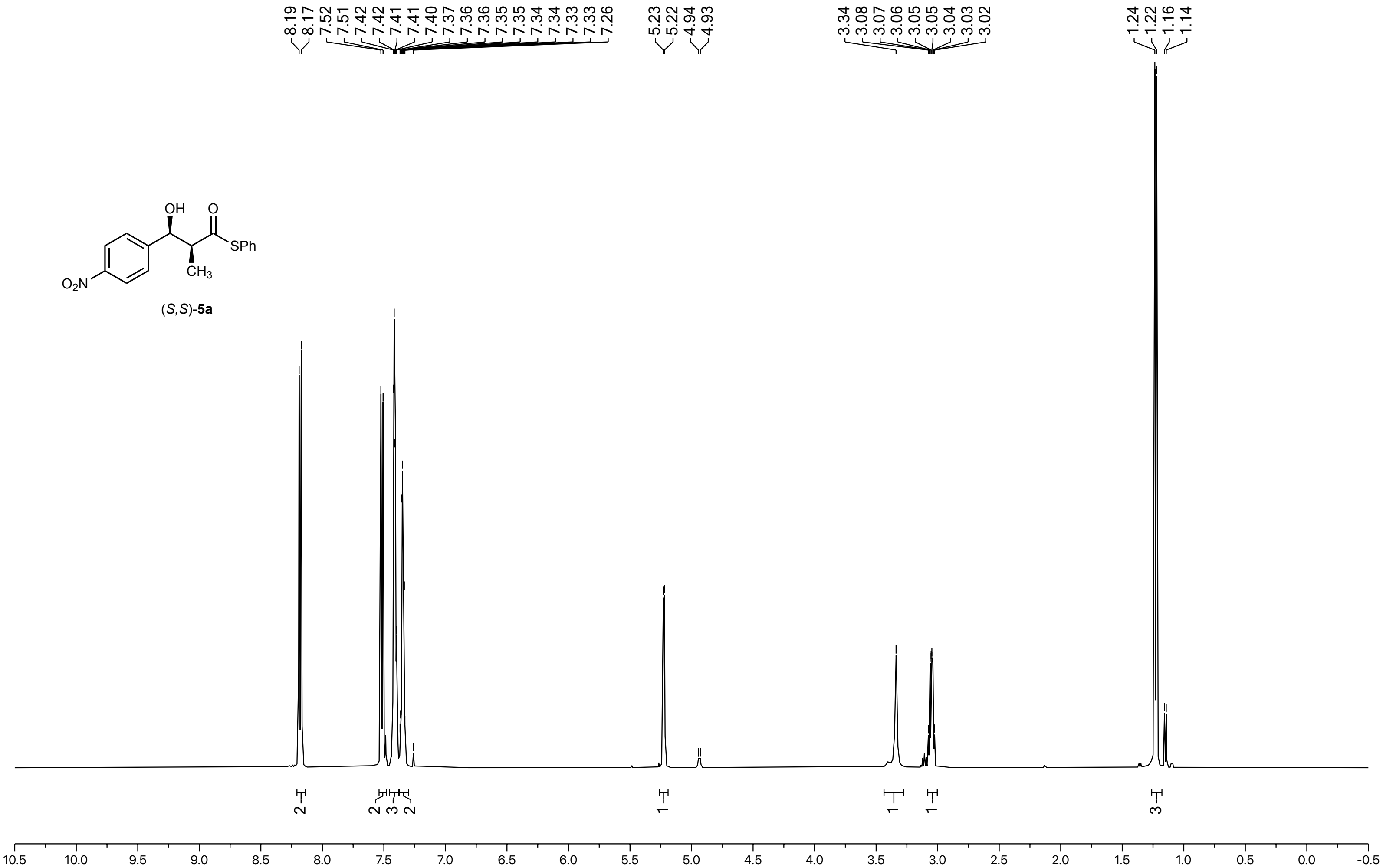

$^{13}C\{^{1}H\}$ NMR, 126 MHz, $CDCl_3$, (*S*,*S*)-**5a**

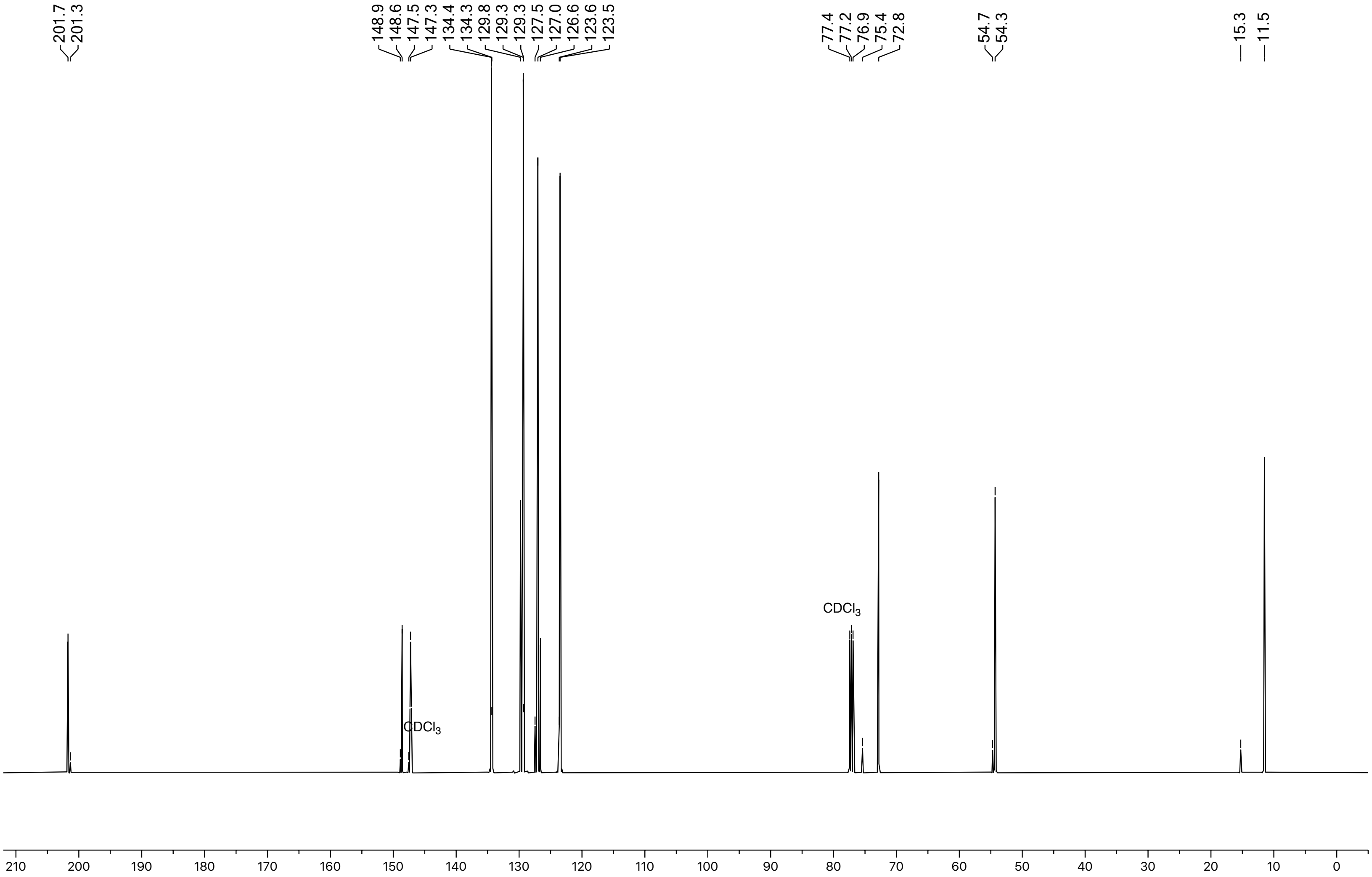

^{1}H NMR, 500 MHz, $CDCl_3$, (*S*,*R*)-**6a**

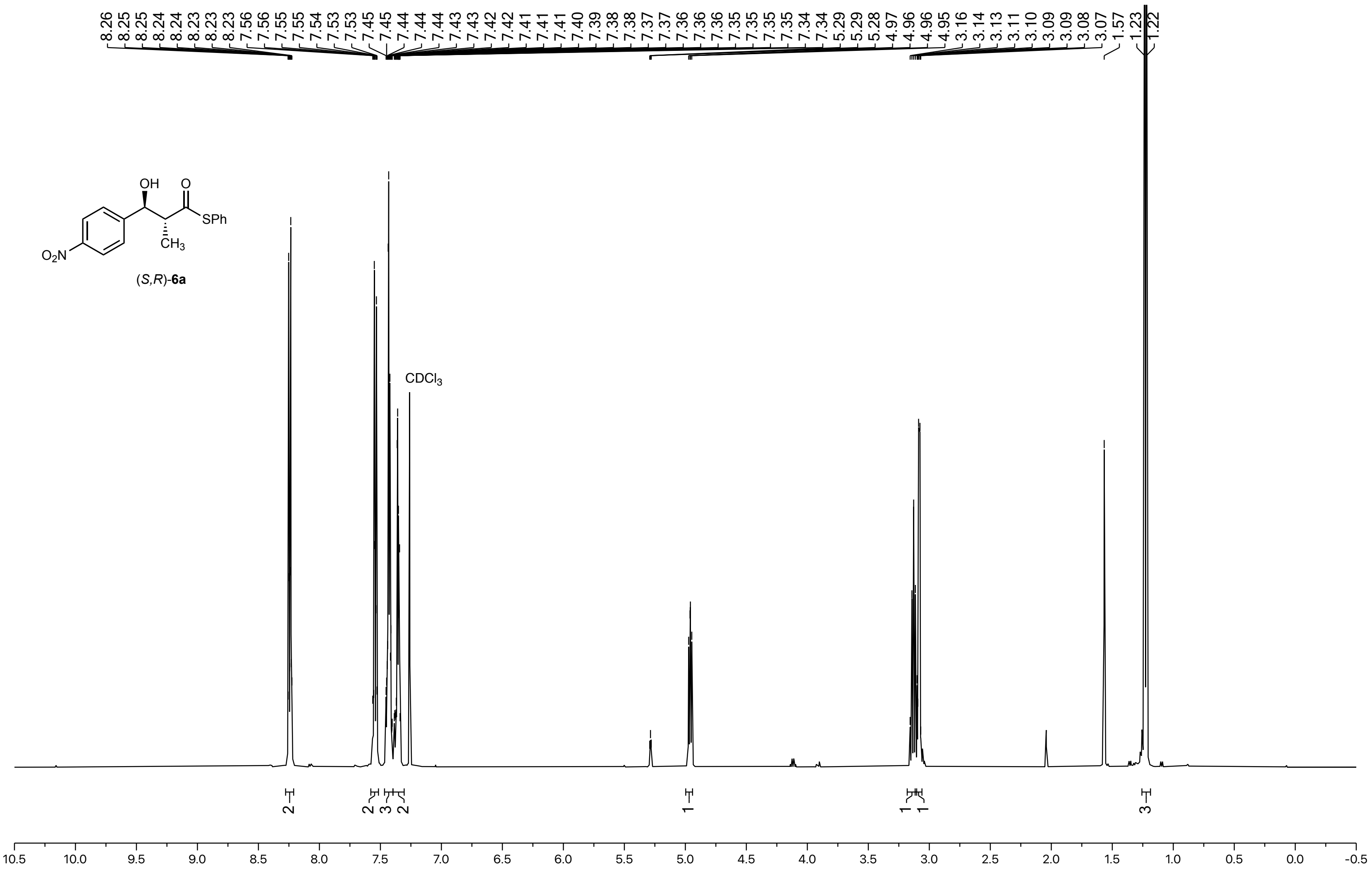

$^{13}C\{^{1}H\}$ NMR, 126 MHz, $CDCl_3$, (*S*,*R*)-**6a**

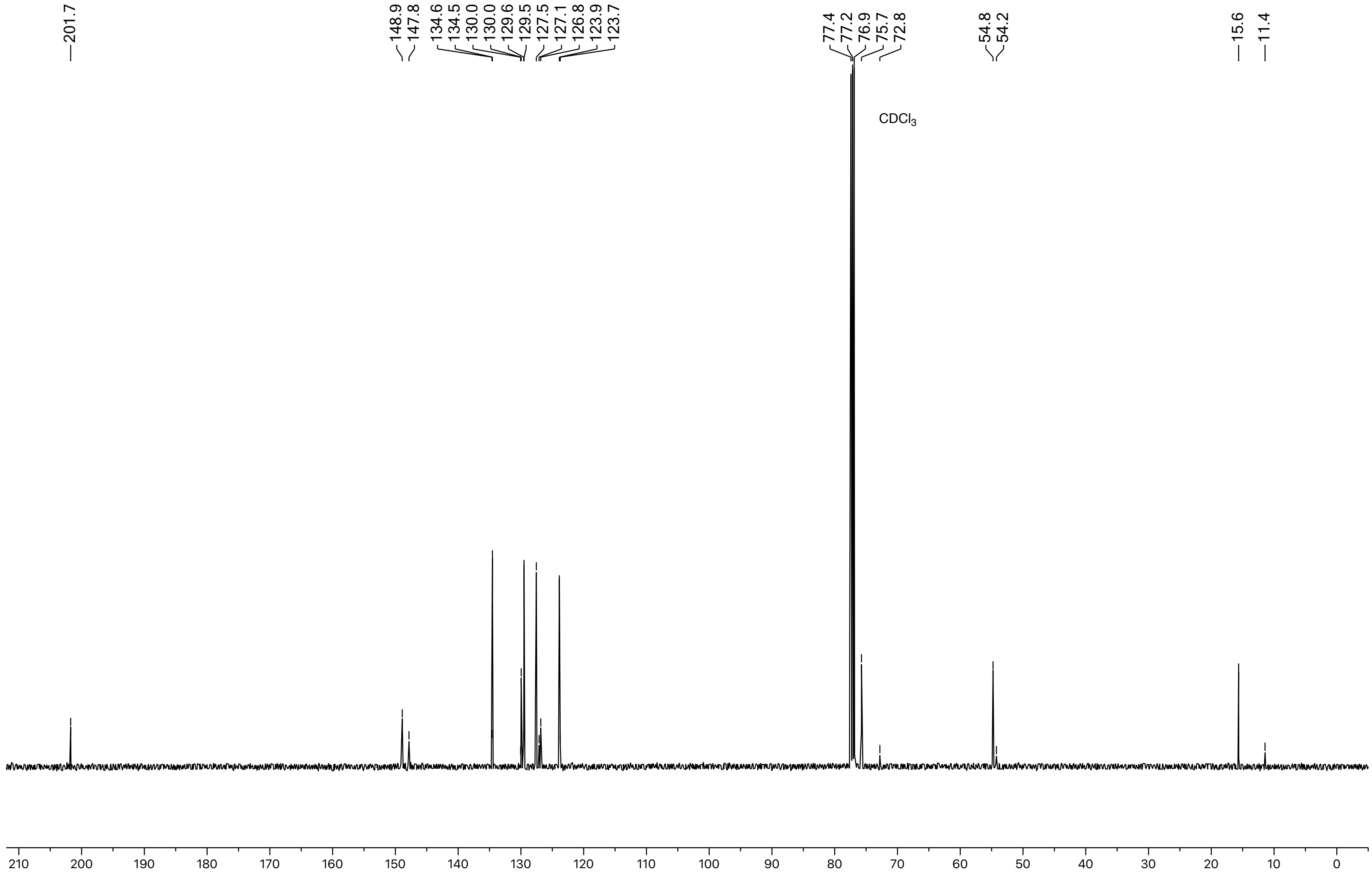

^{1}H NMR, 500 MHz, $CDCl_3$, (*R*,*R*)-**5a**

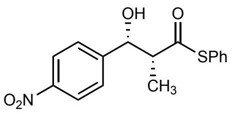

(*R*,*R*)-**5a**

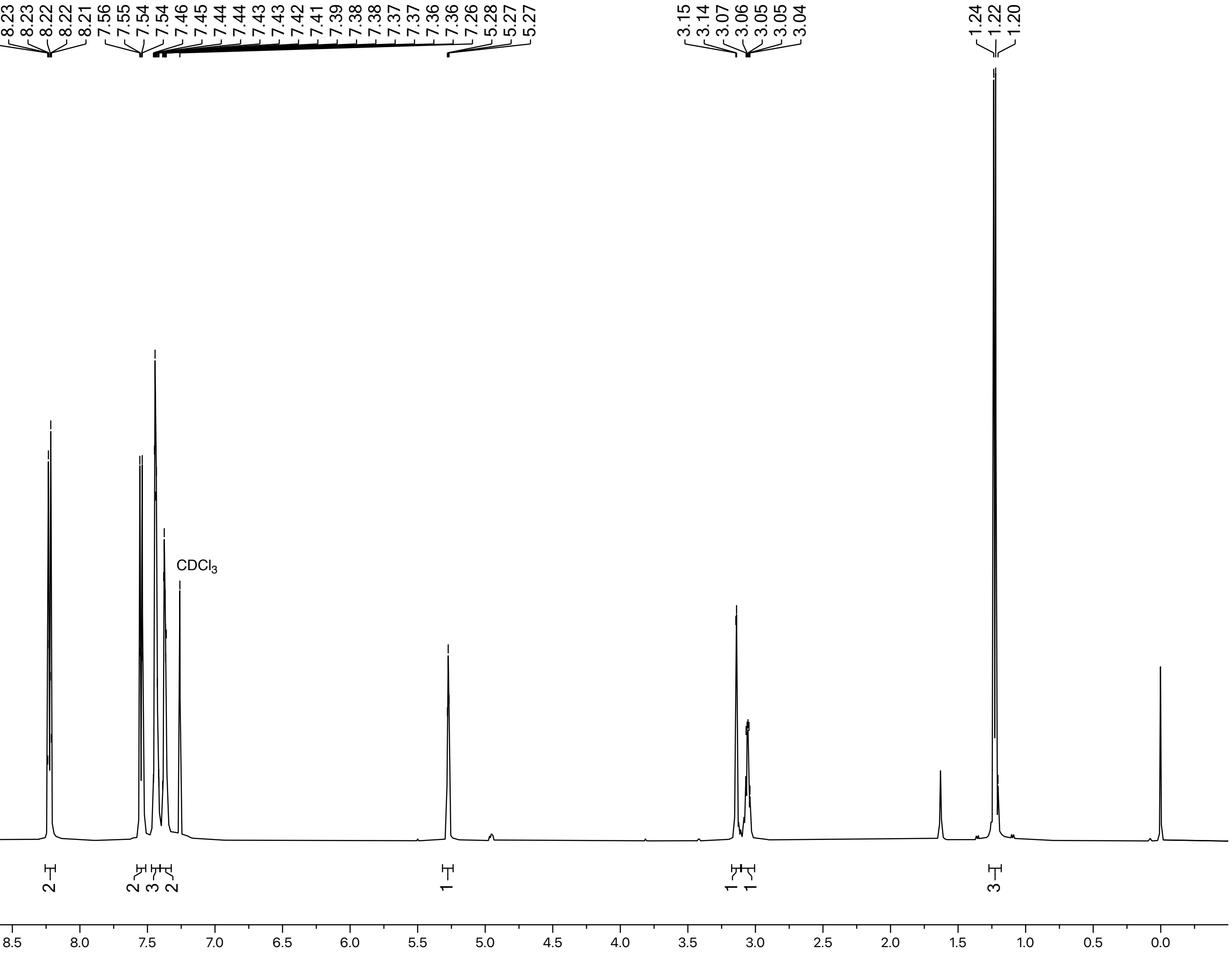

$^{13}C\{^{1}H\}$ NMR, 126 MHz, $CDCl_3$, (*R*,*R*)-**5a**

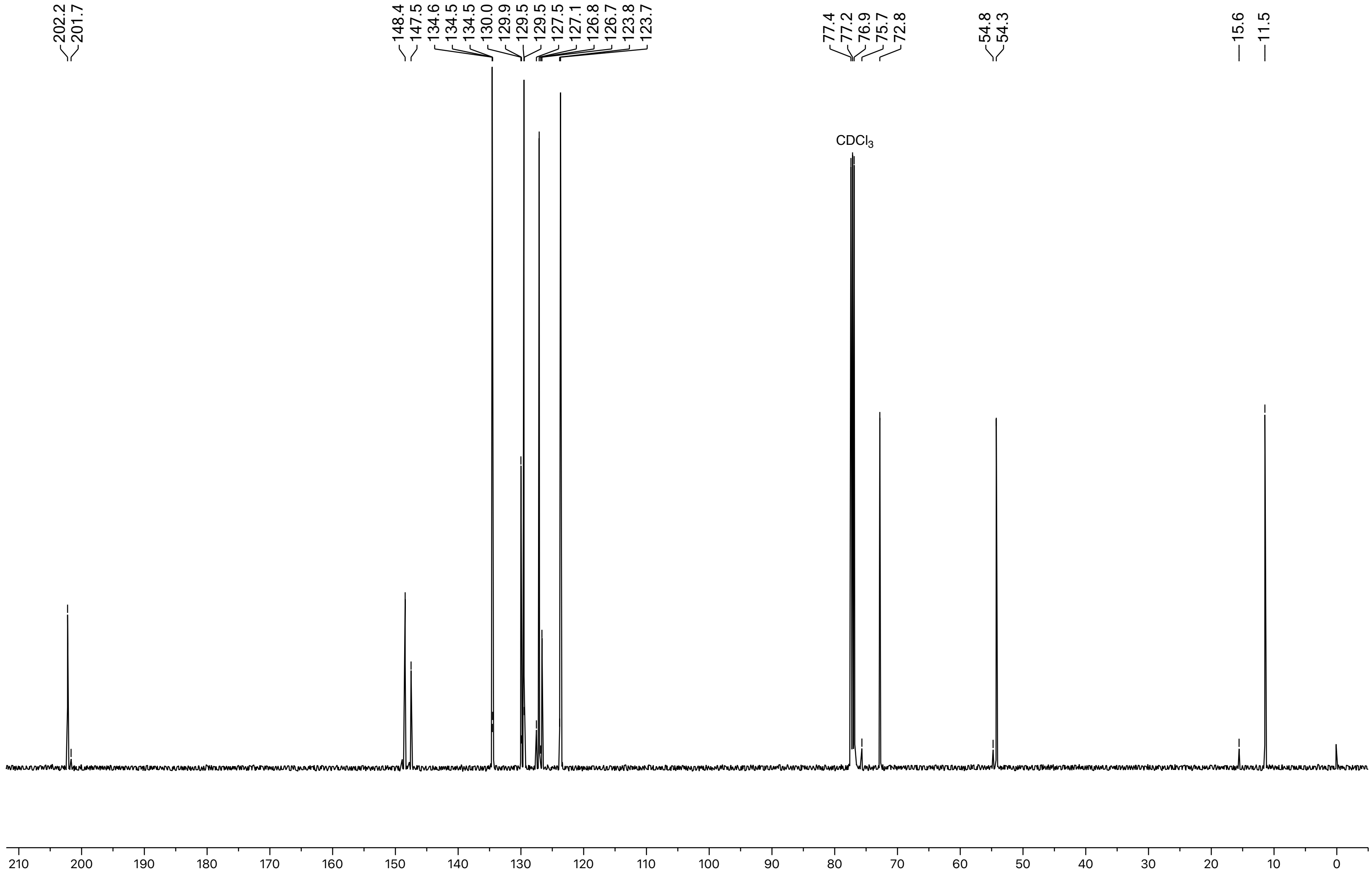

^{1}H NMR, 500 MHz, $CDCl_3$, (*R*,*S*)-**6a**

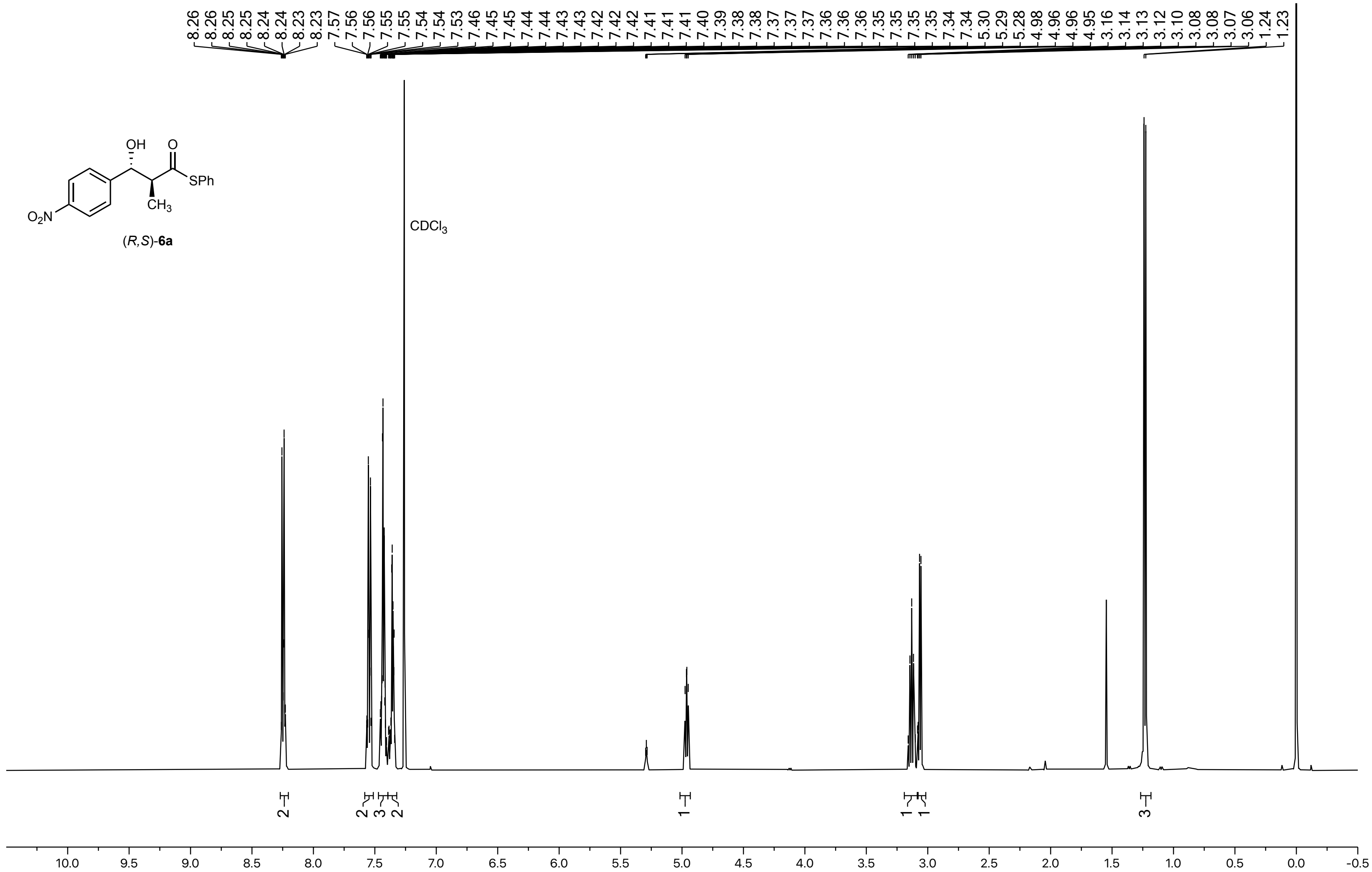

$^{13}C\{^{1}H\}$ NMR, 126 MHz, $CDCl_3$, (*R*,*S*)-**6a**

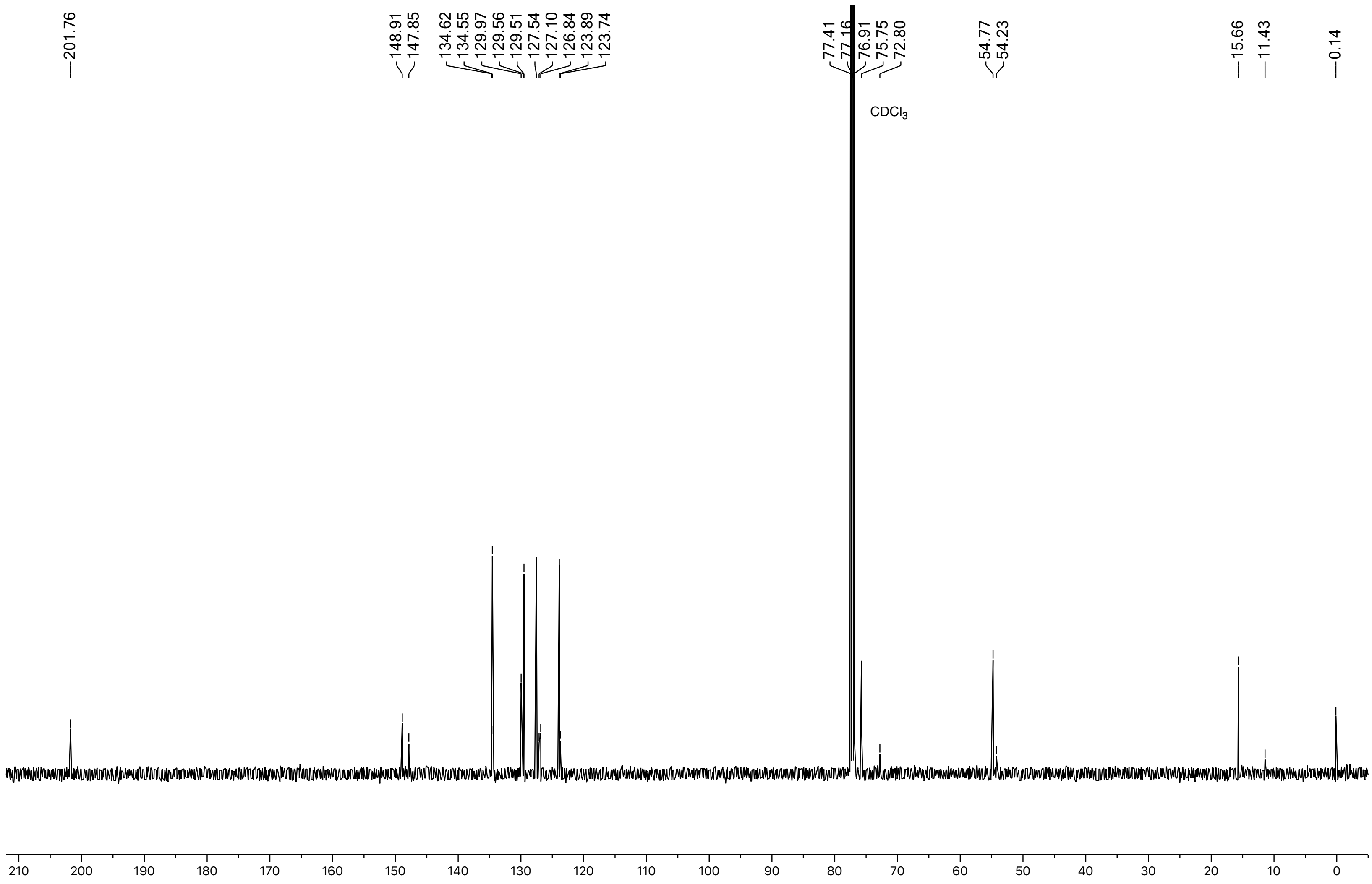

^1H NMR, 500 MHz, $CDCl_3$, **5b**

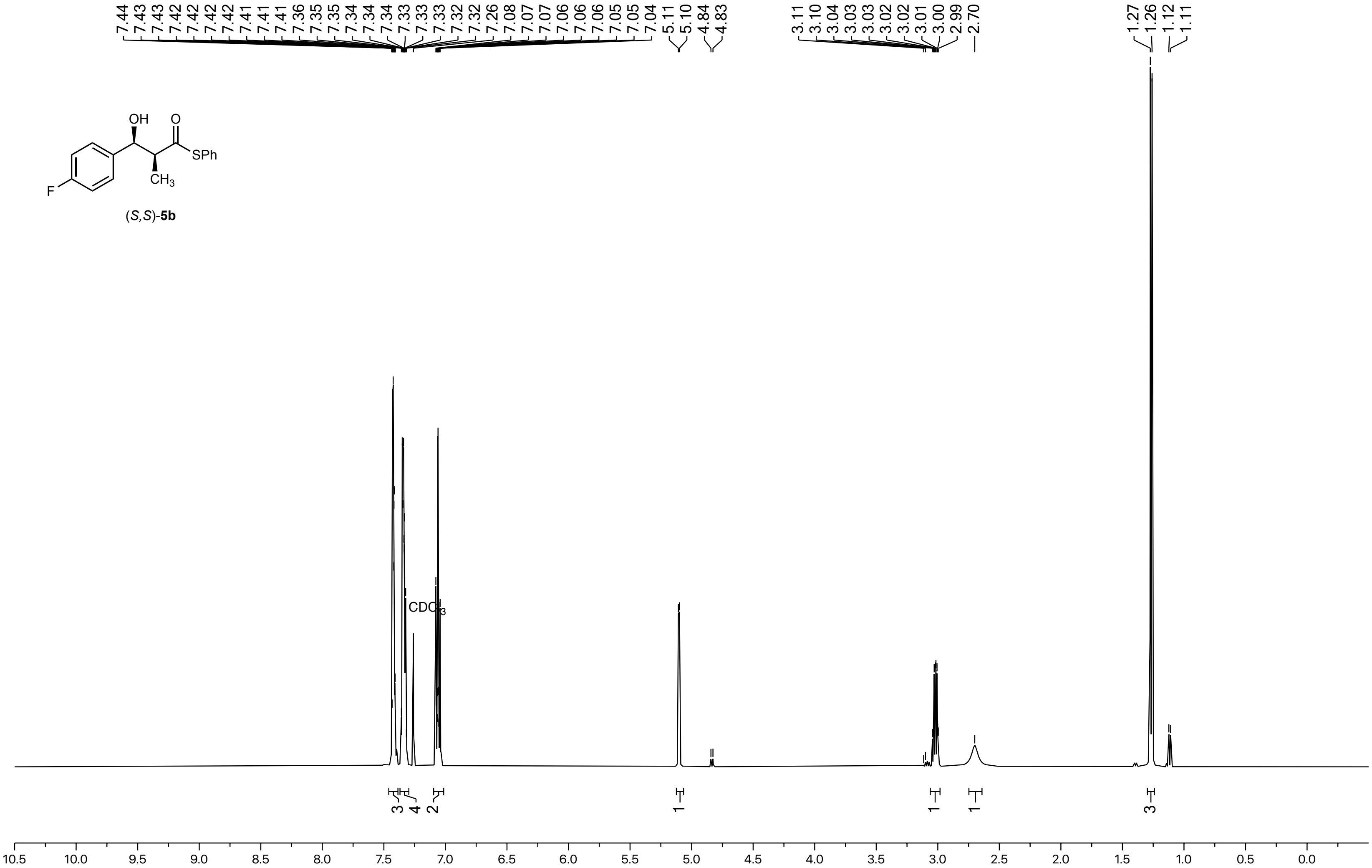

$^{13}C\{^{1}H\}$ NMR, 126 MHz, $CDCl_3$, **5b**

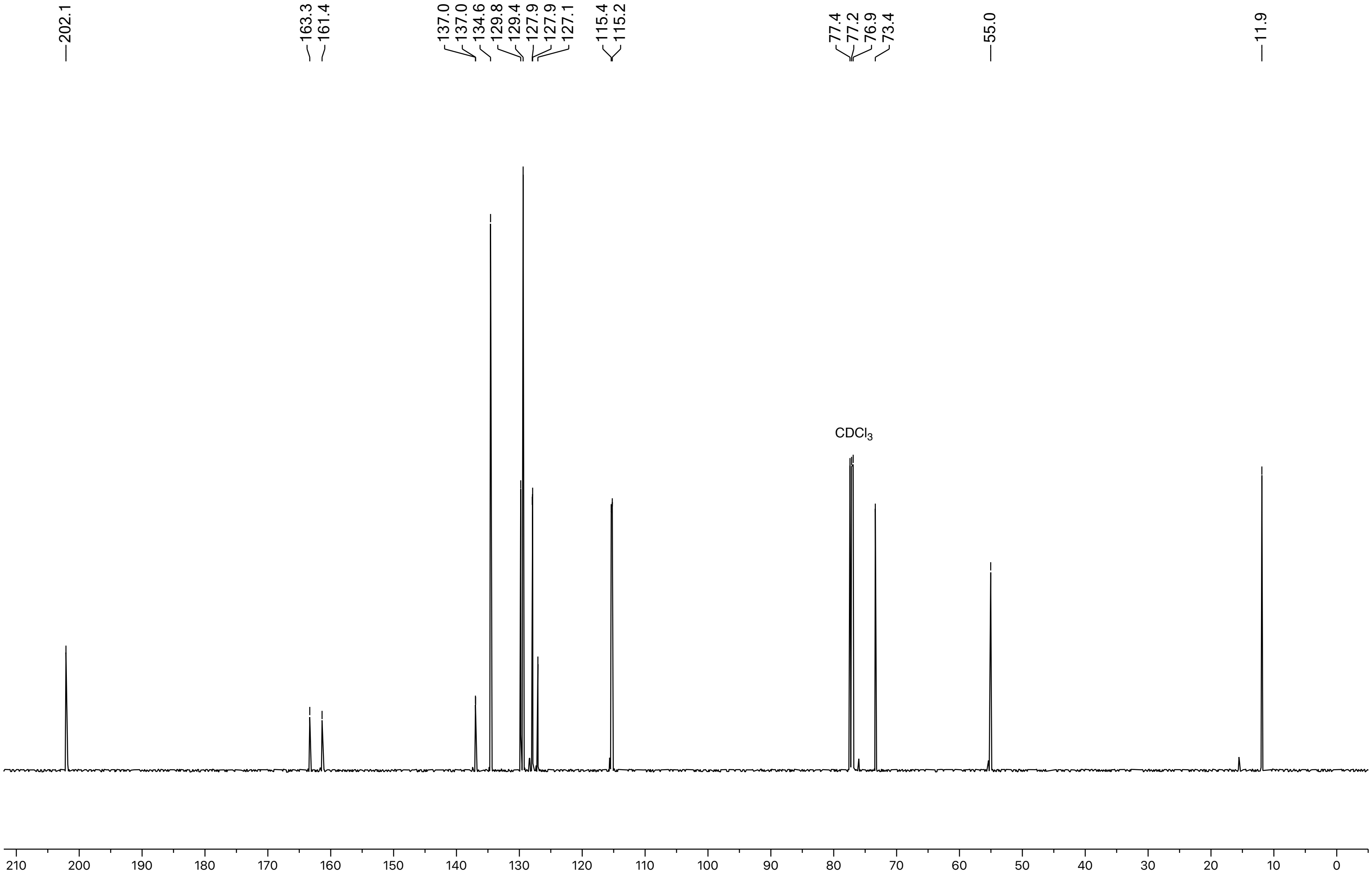

^{19}F NMR, 470 MHz, $CDCl_3$, **5b**

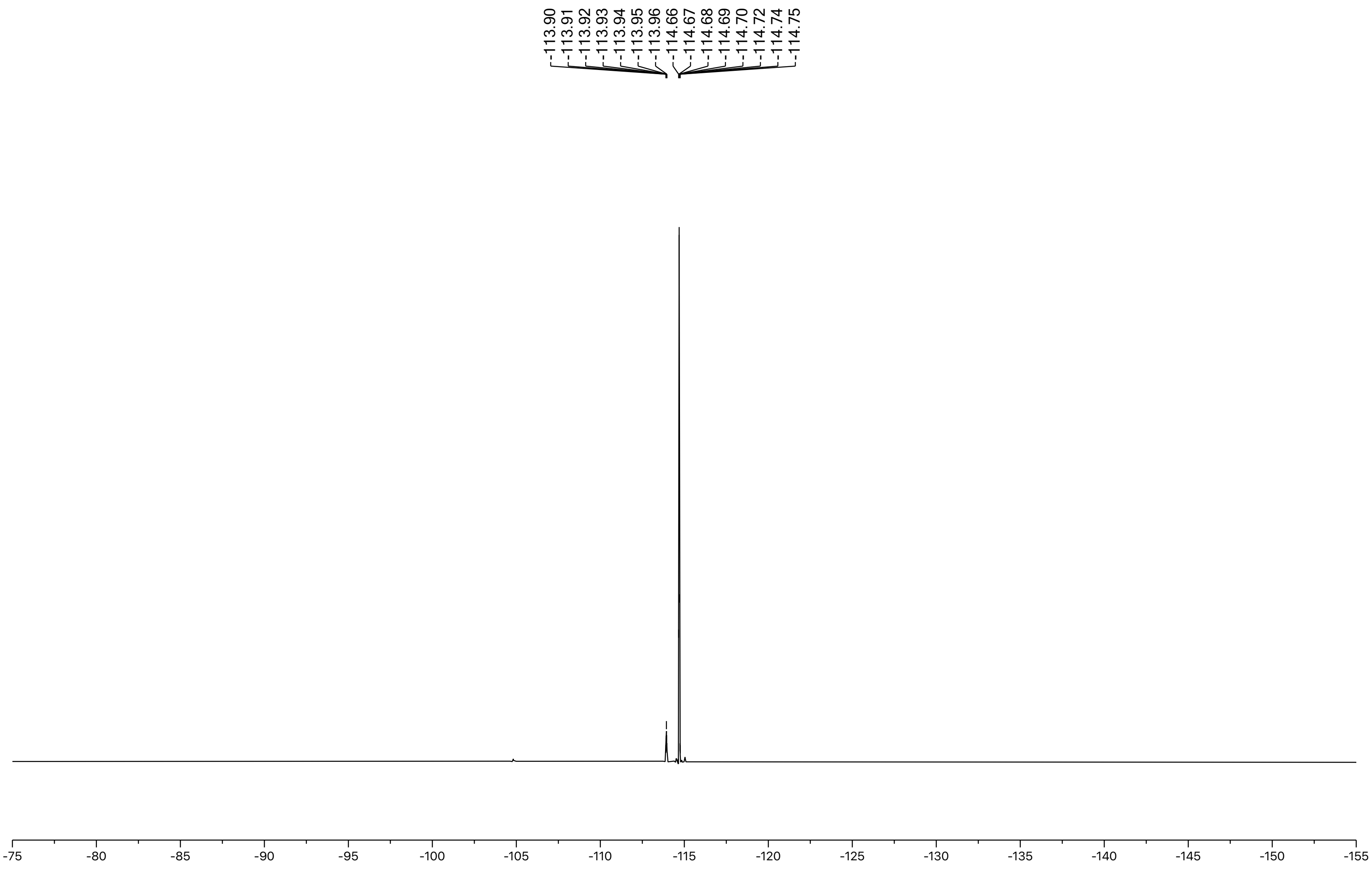

^{1}H NMR, 500 MHz, $CDCl_3$, **6b**

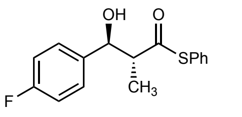

(*S*,*R*)-**6b**

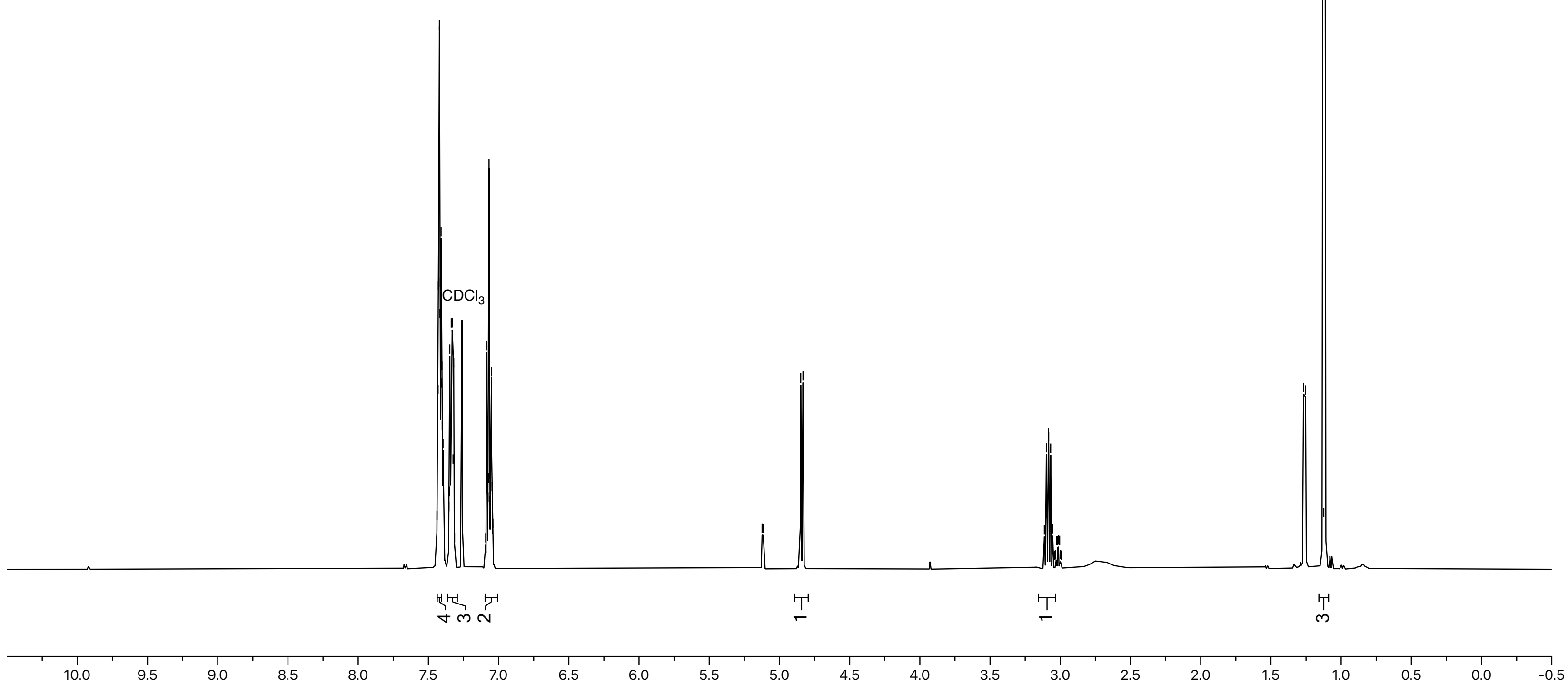

$^{13}C\{^{1}H\}$ NMR, 126 MHz, $CDCl_3$, **6b**

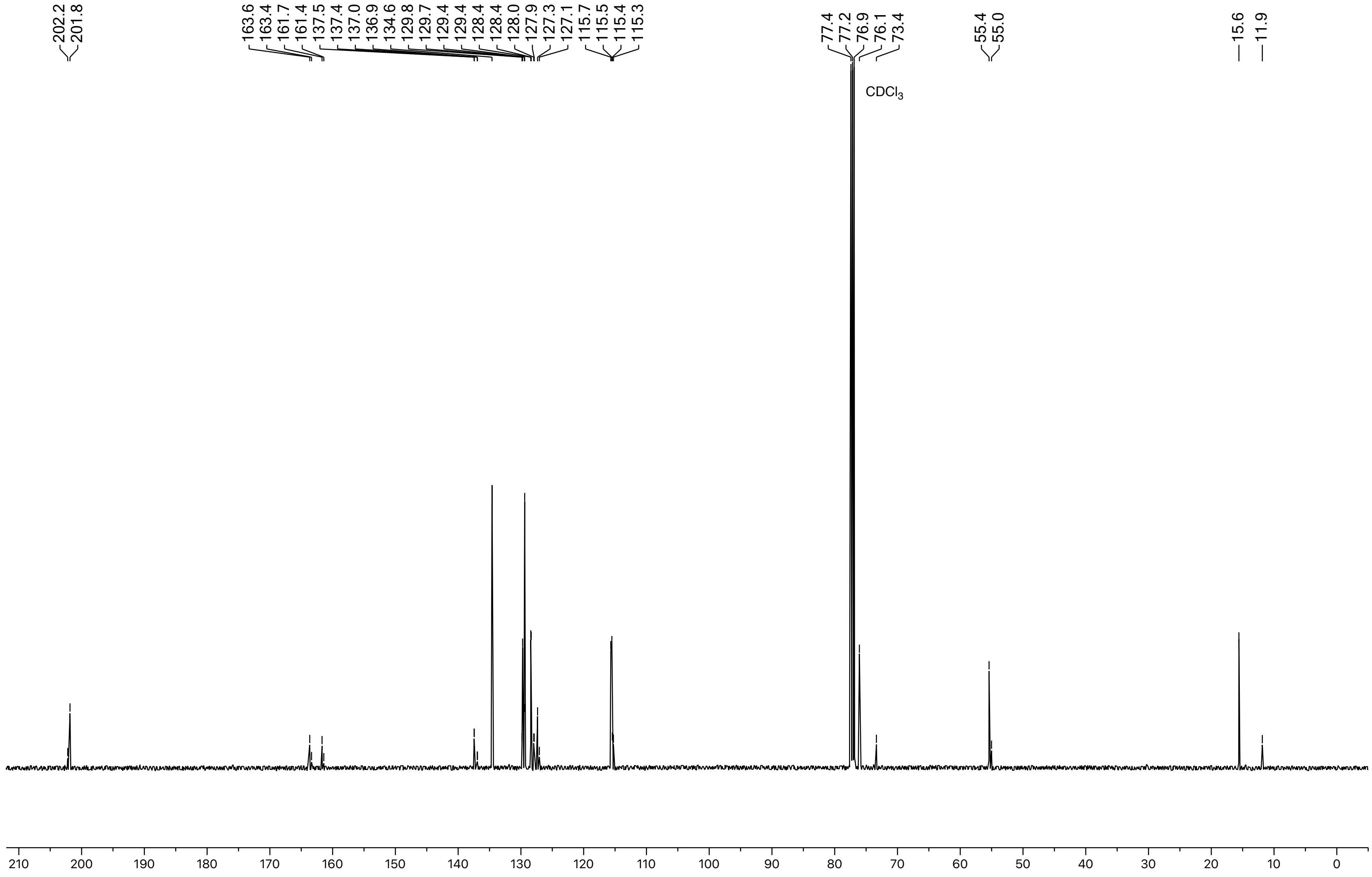

^{19}F NMR, 470 MHz, $CDCl_3$, **6b**

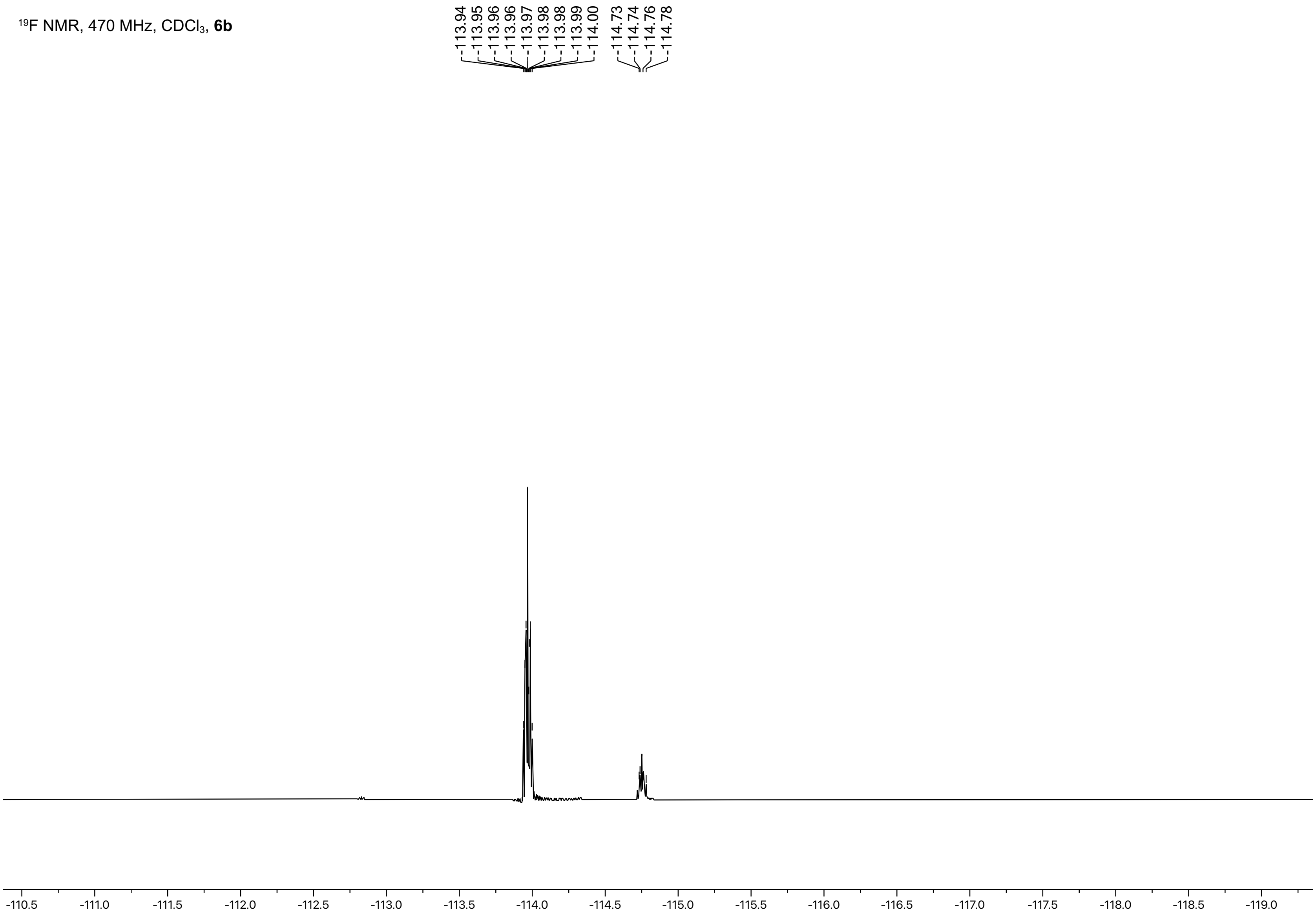

^{1}H NMR, 500 MHz, $CDCl_3$, **5c**

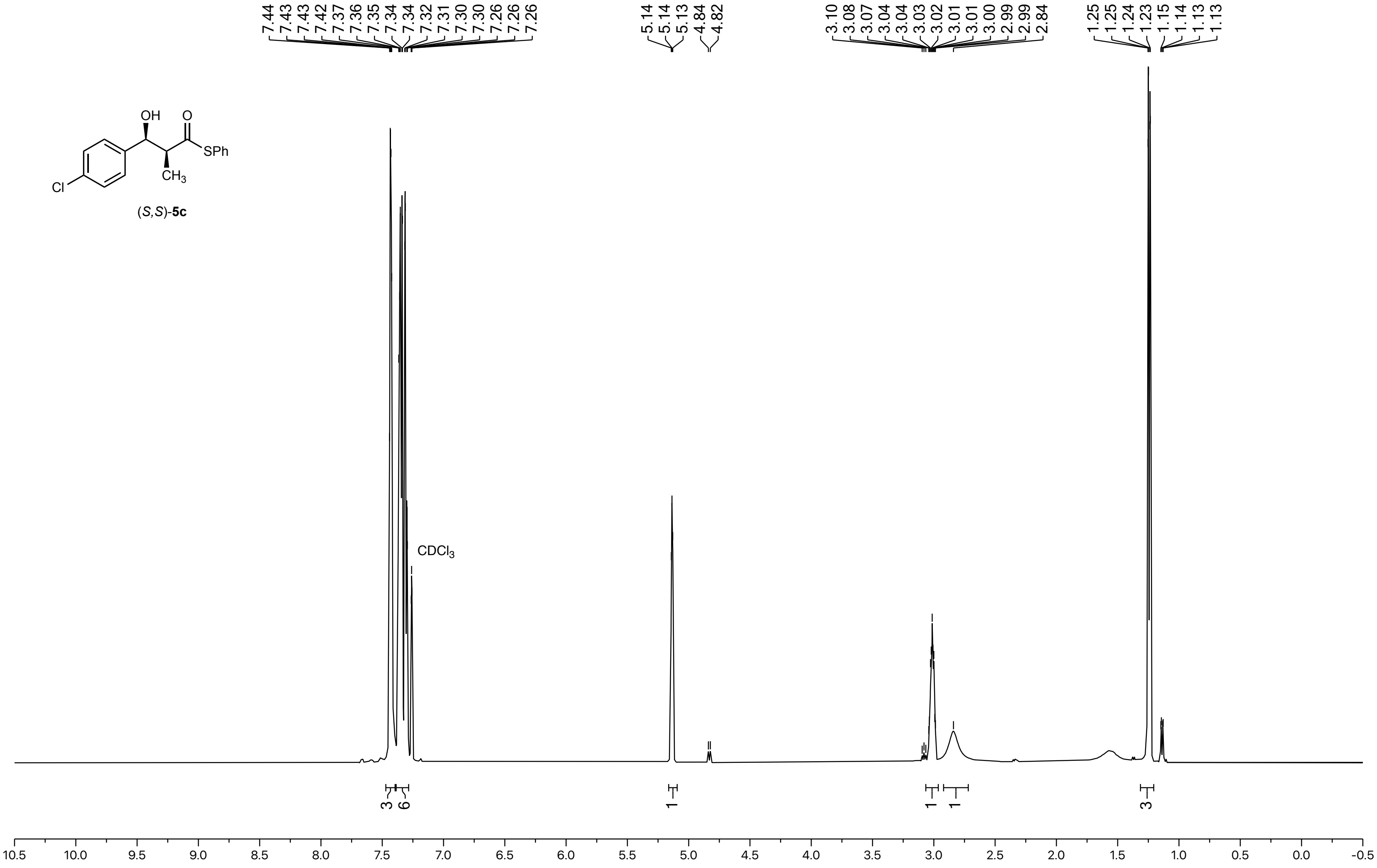

$^{13}C\{^{1}H\}$ NMR, 126 MHz, $CDCl_3$, **5c**

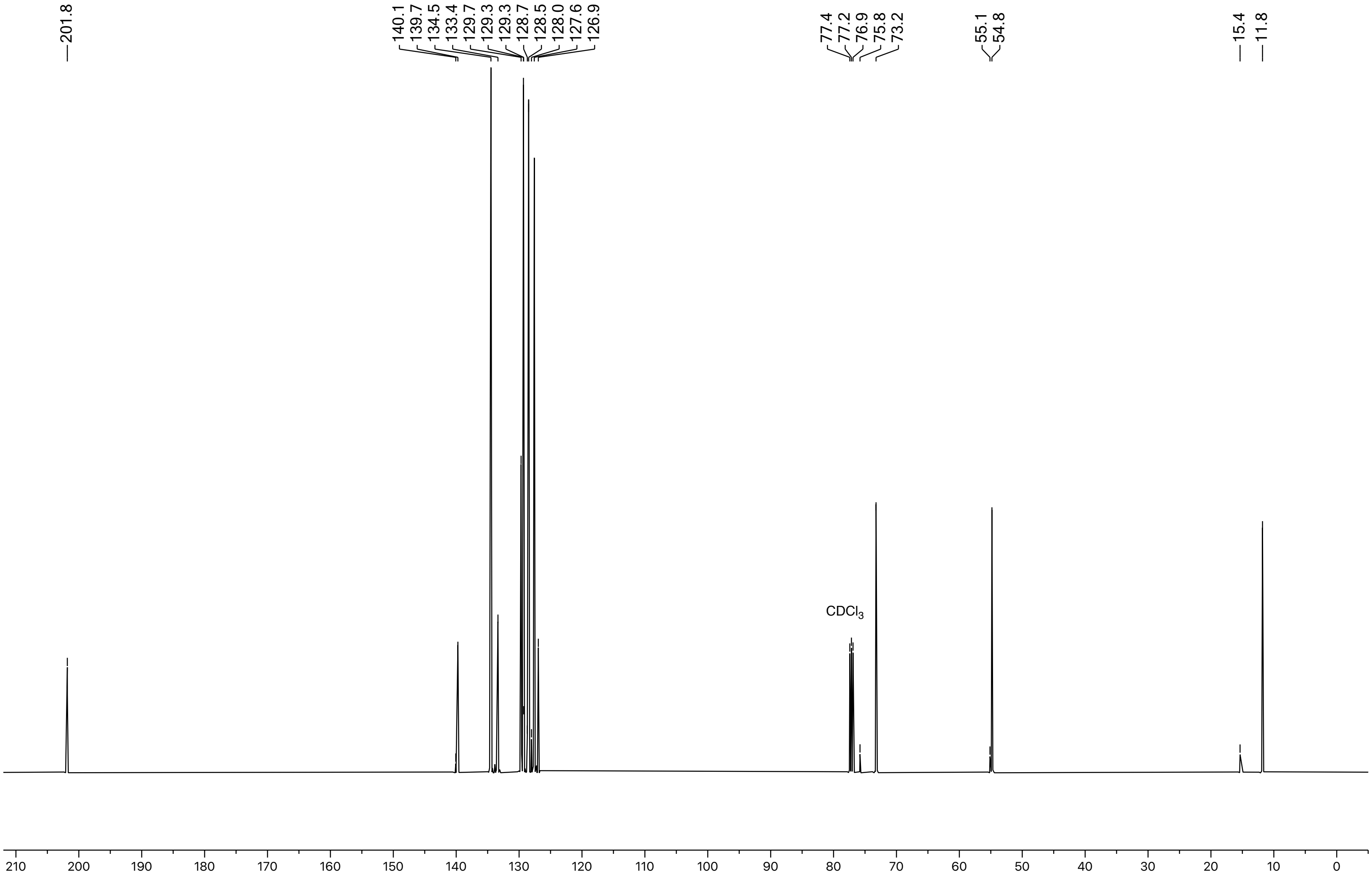

^{1}H NMR, 500 MHz, $CDCl_3$, **6c**

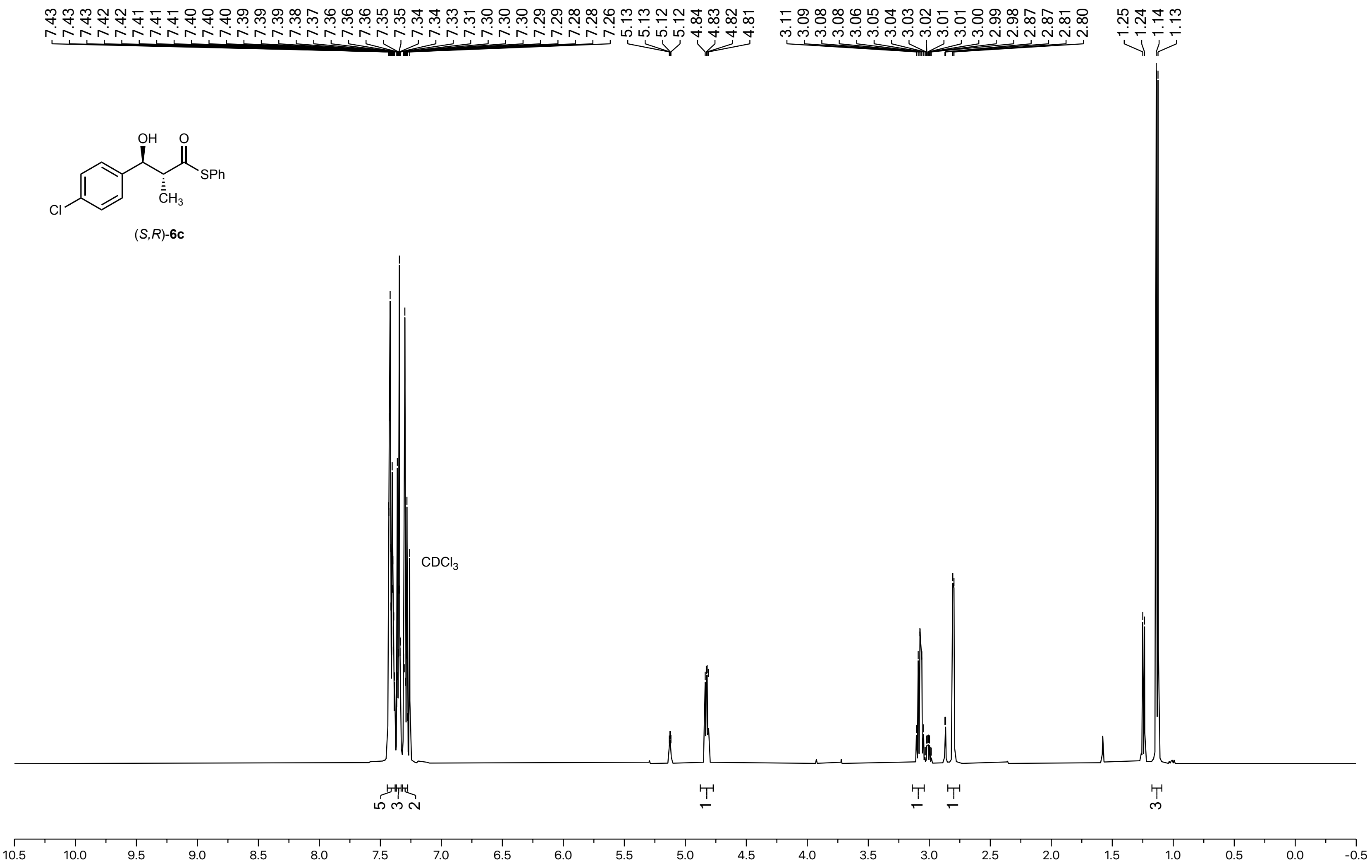

$^{13}C\{^1H\}$ NMR, 126 MHz, $CDCl_3$, **6c**

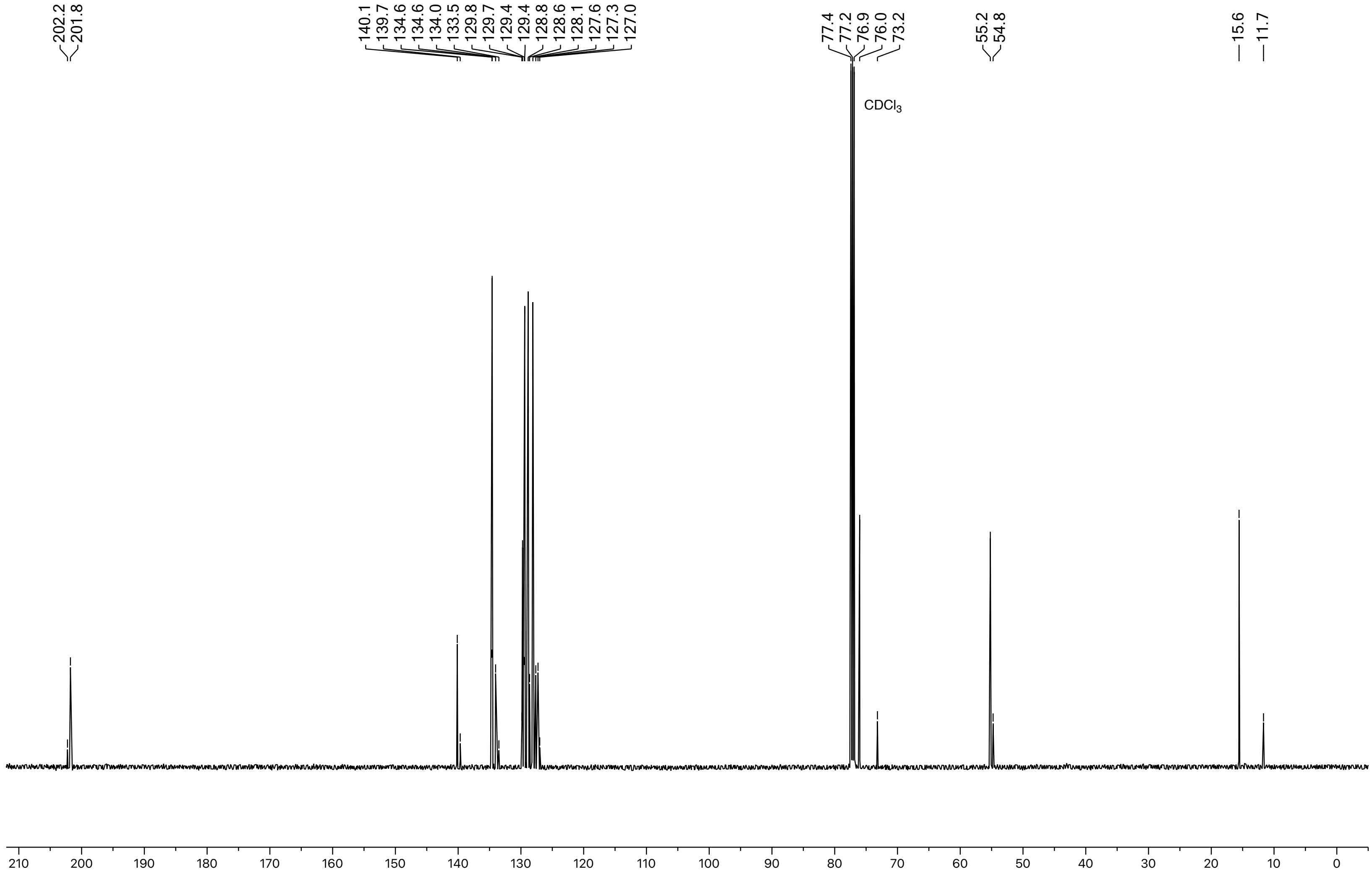

^{1}H NMR, 500 MHz, $CDCl_3$, **5d**

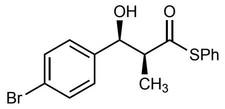

(*S*,*S*)-**5d**

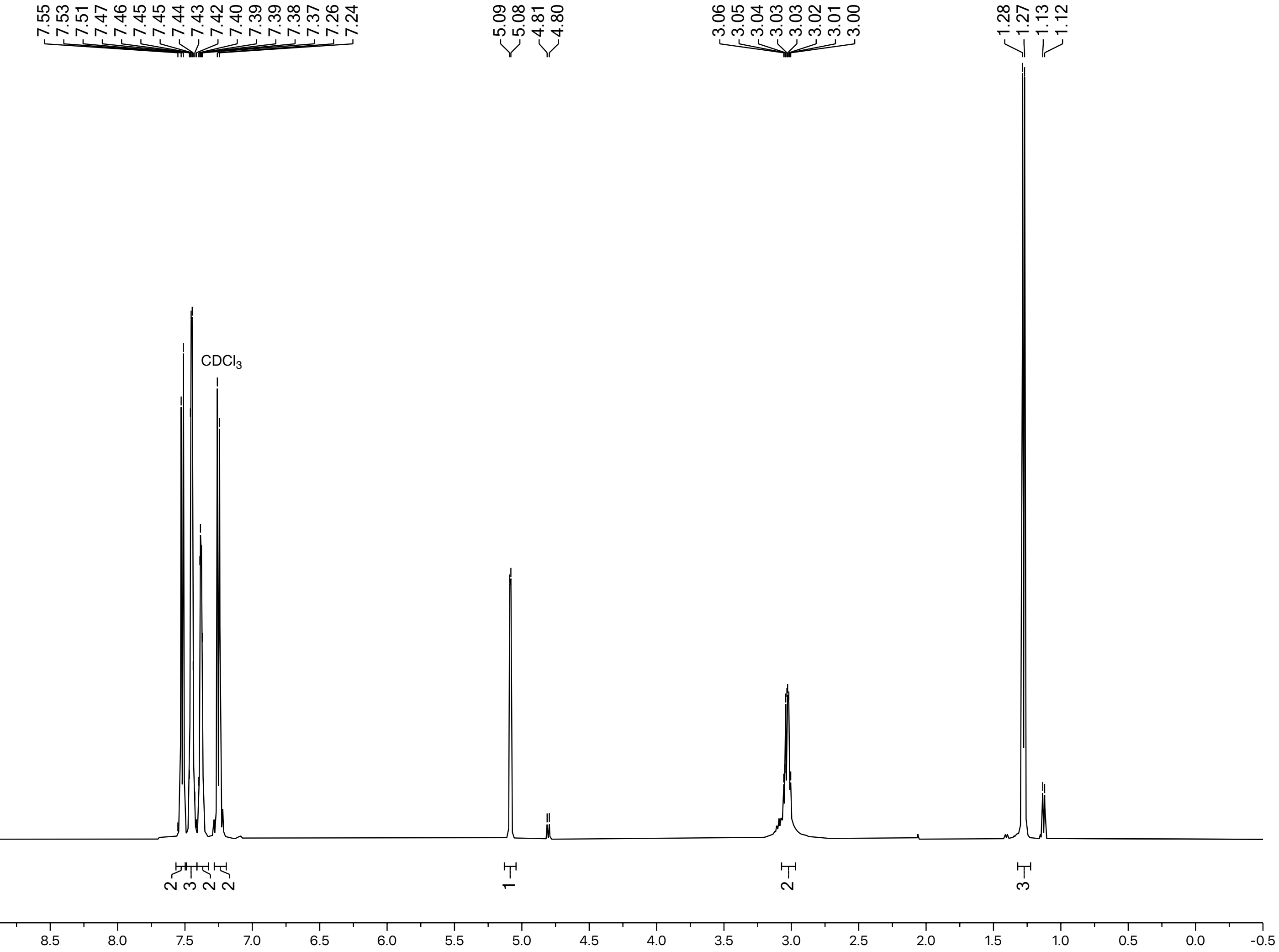

$^{13}C\{^{1}H\}$ NMR, 126 MHz, $CDCl_3$, **5d**

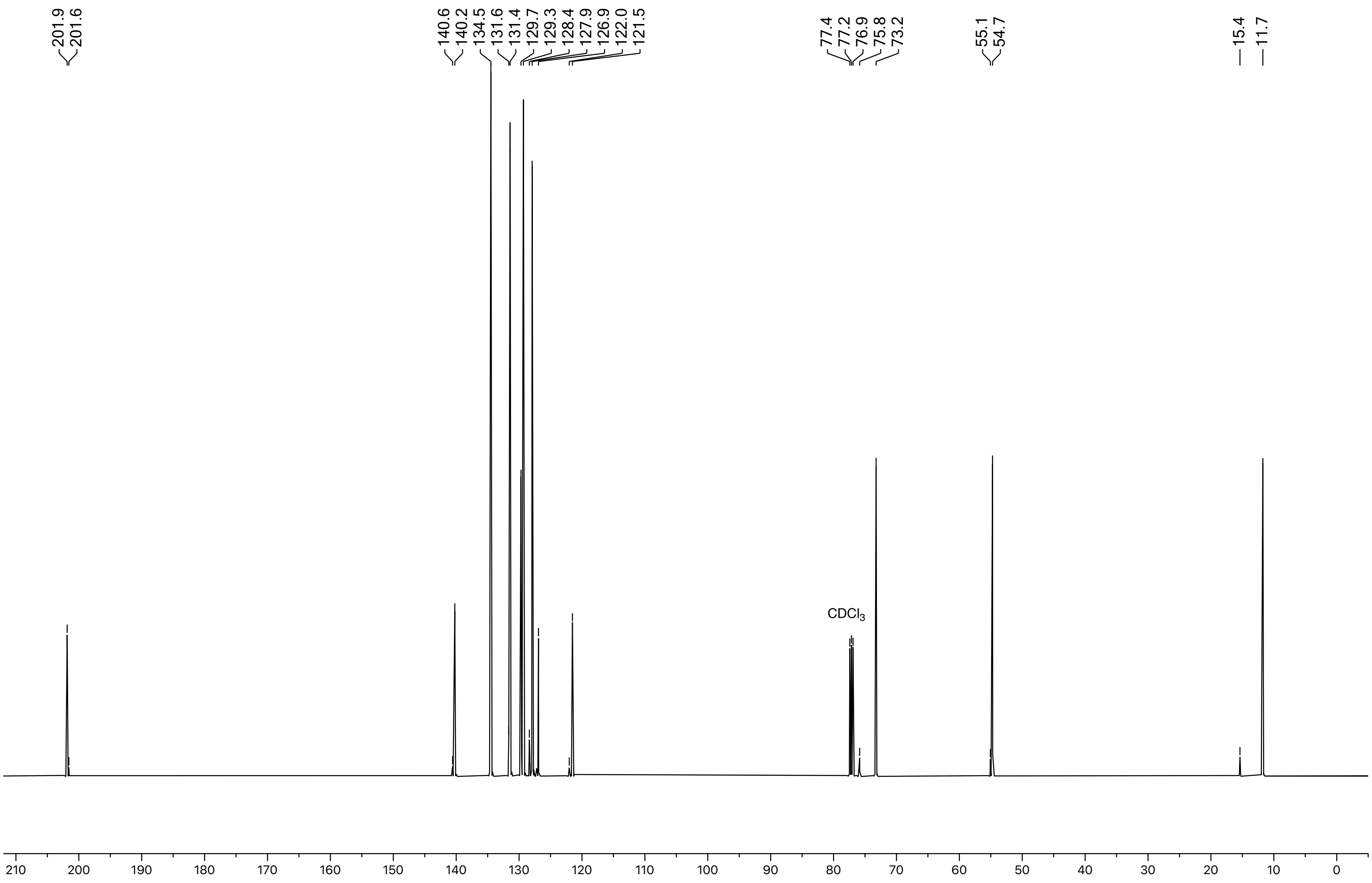

^1H NMR, 500 MHz, $CDCl_3$, **6d**

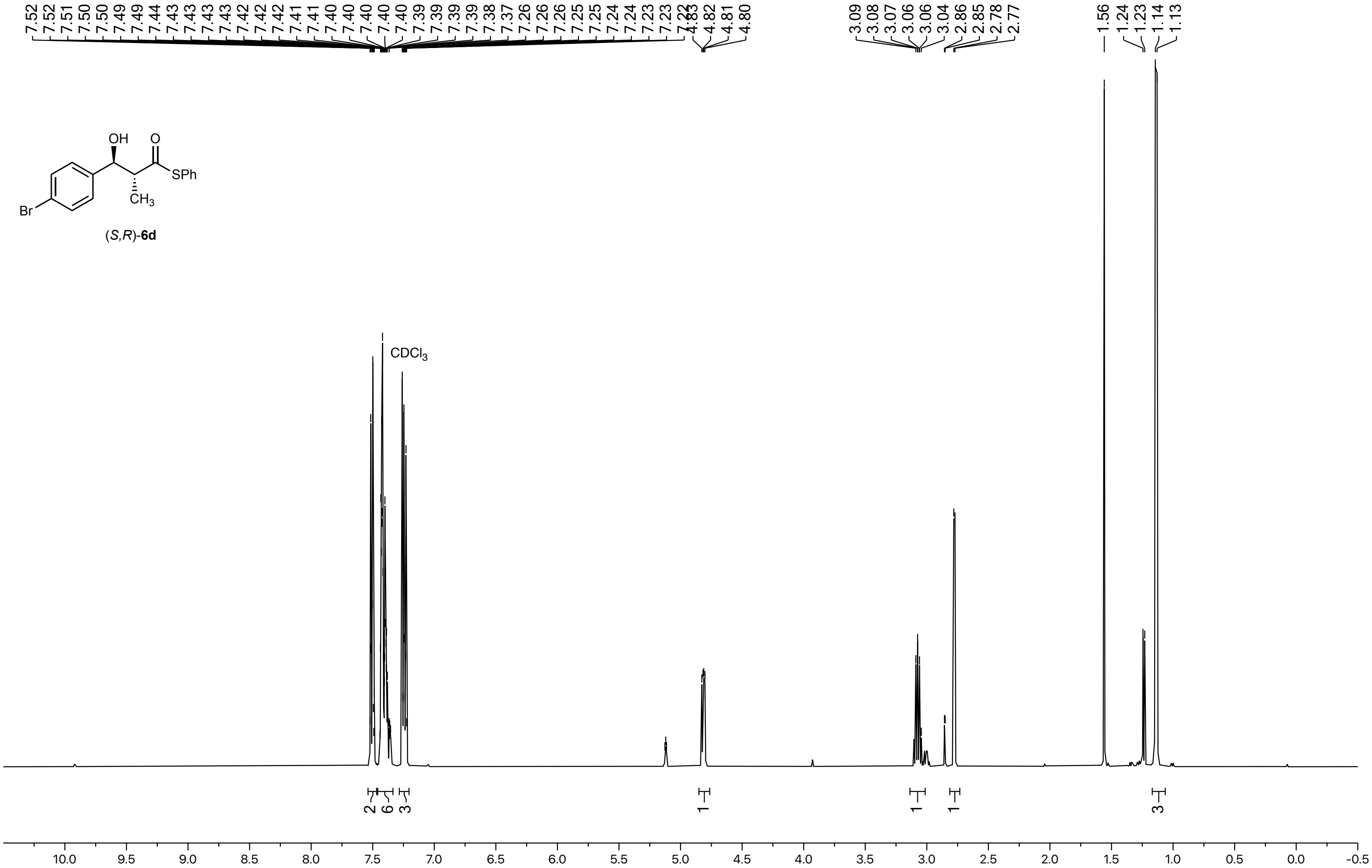

$^{13}C\{^1H\}$ NMR, 126 MHz, $CDCl_3$, **6d**

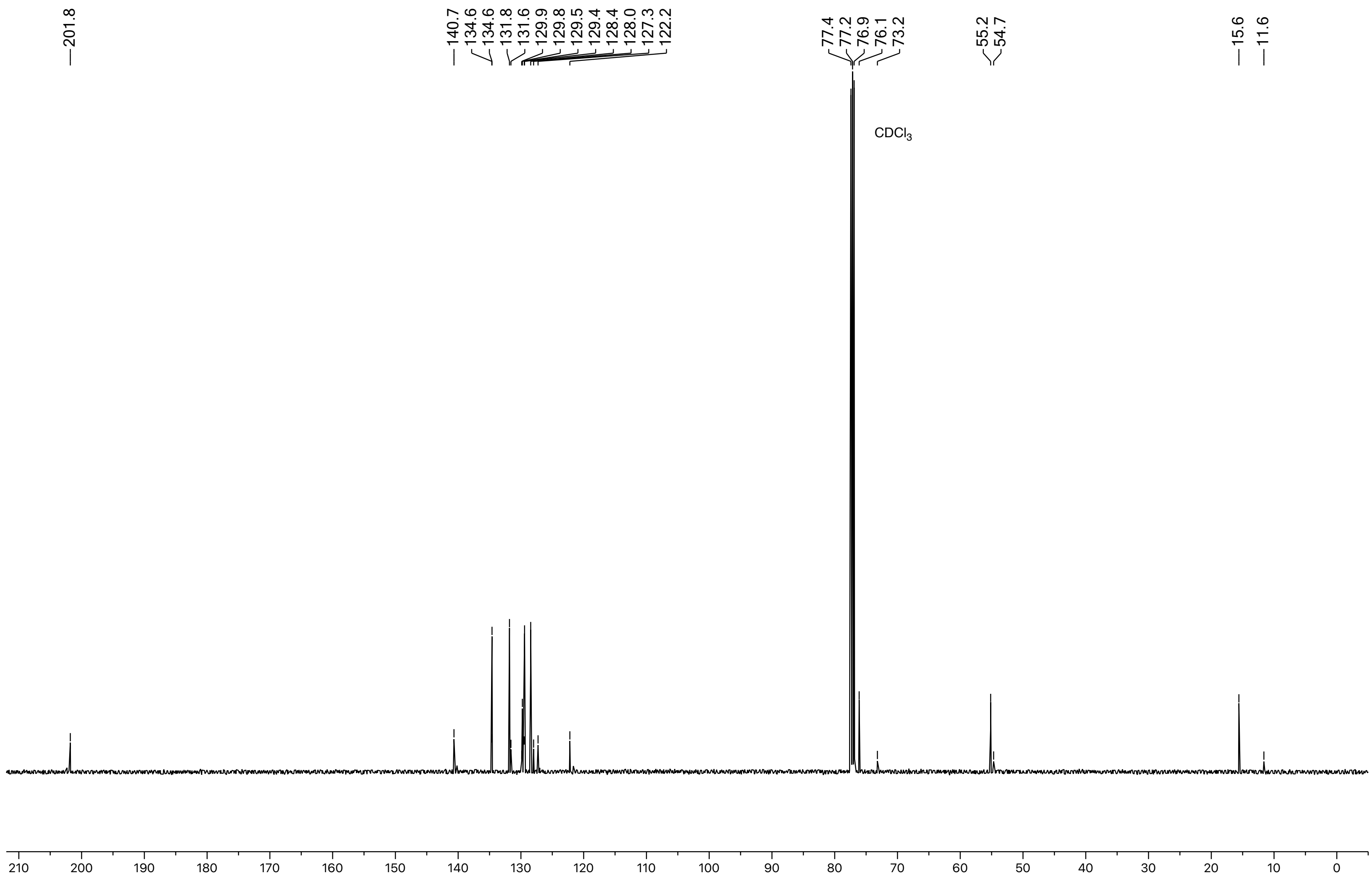

^{1}H NMR, 500 MHz, $CDCl_3$, **5e**

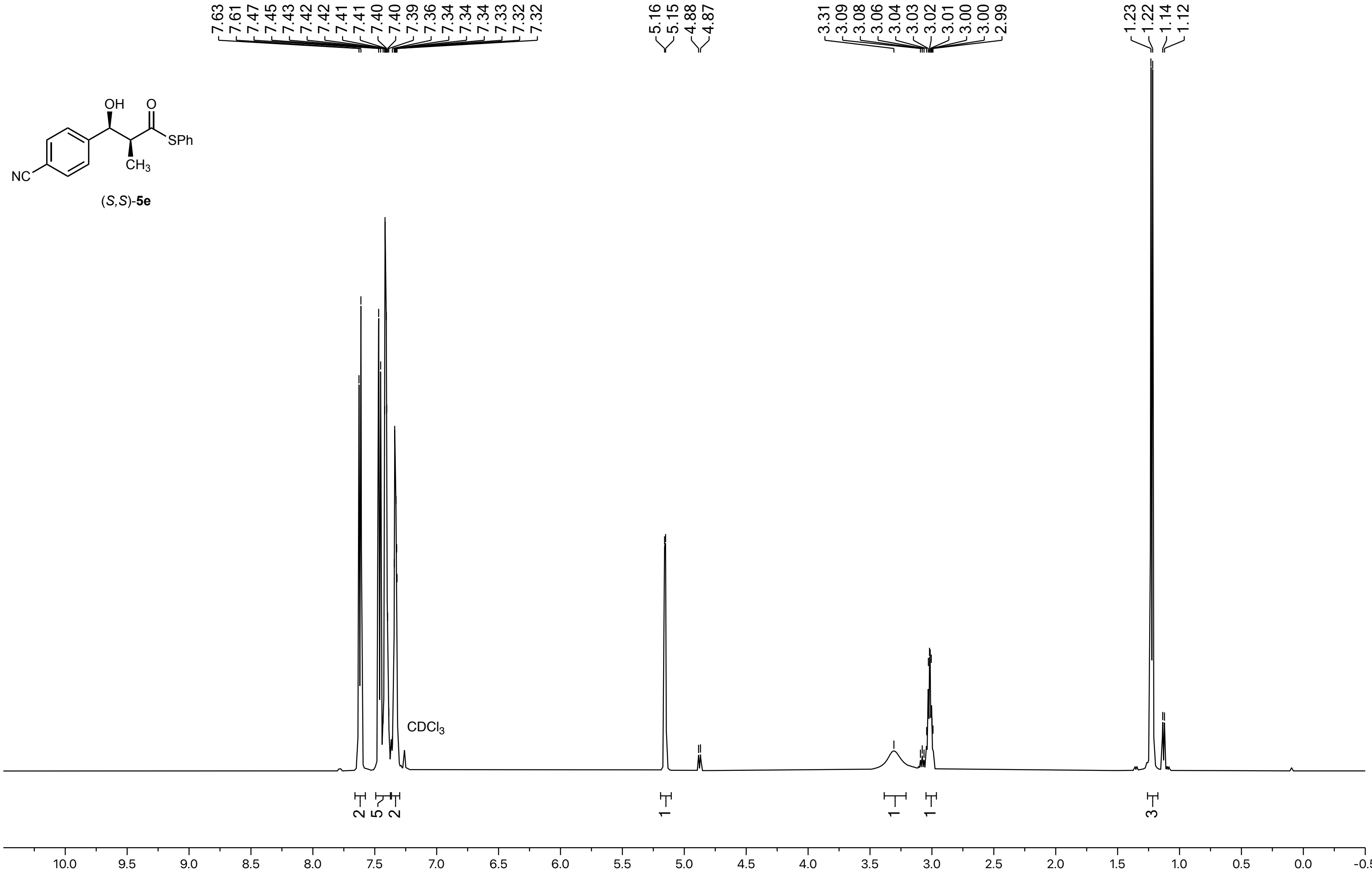

$^{13}C\{^{1}H\}$ NMR, 126 MHz, $CDCl_3$, **5e**

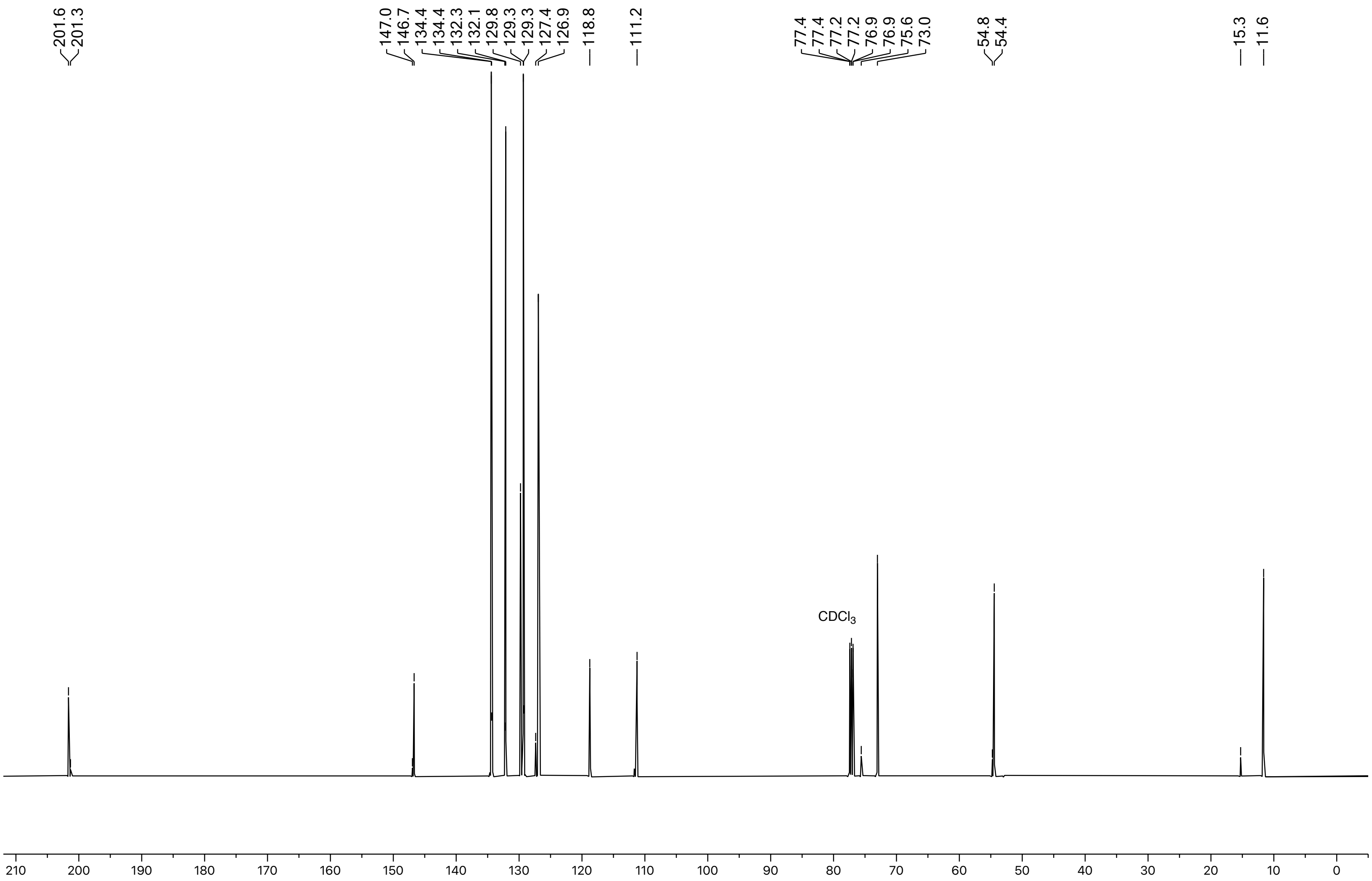

^{1}H NMR, 500 MHz, $CDCl_3$, **6e**

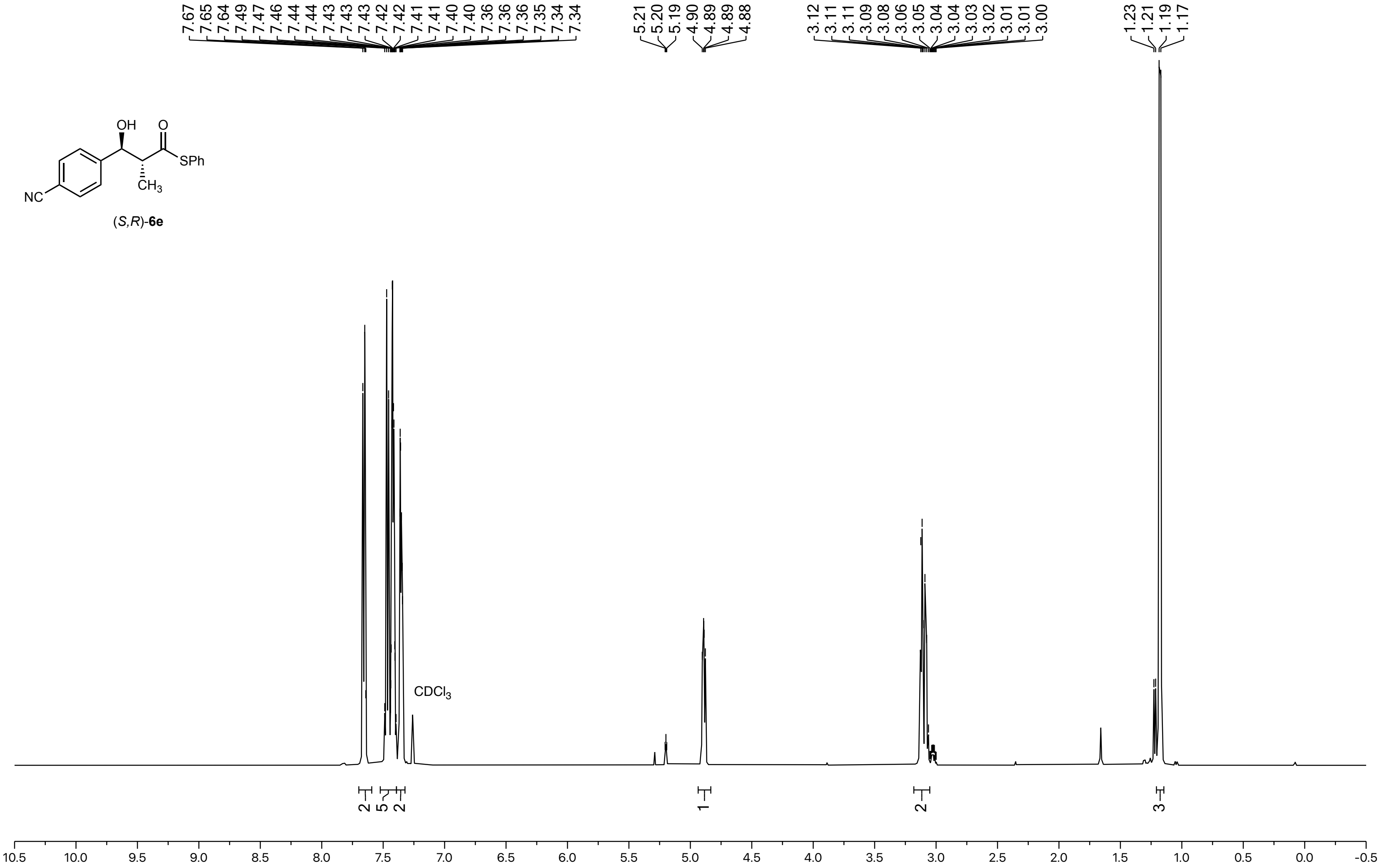

(*S*,*R*)-**6e**

$^{13}C\{^{1}H\}$ NMR, 126 MHz, $CDCl_3$, **6e**

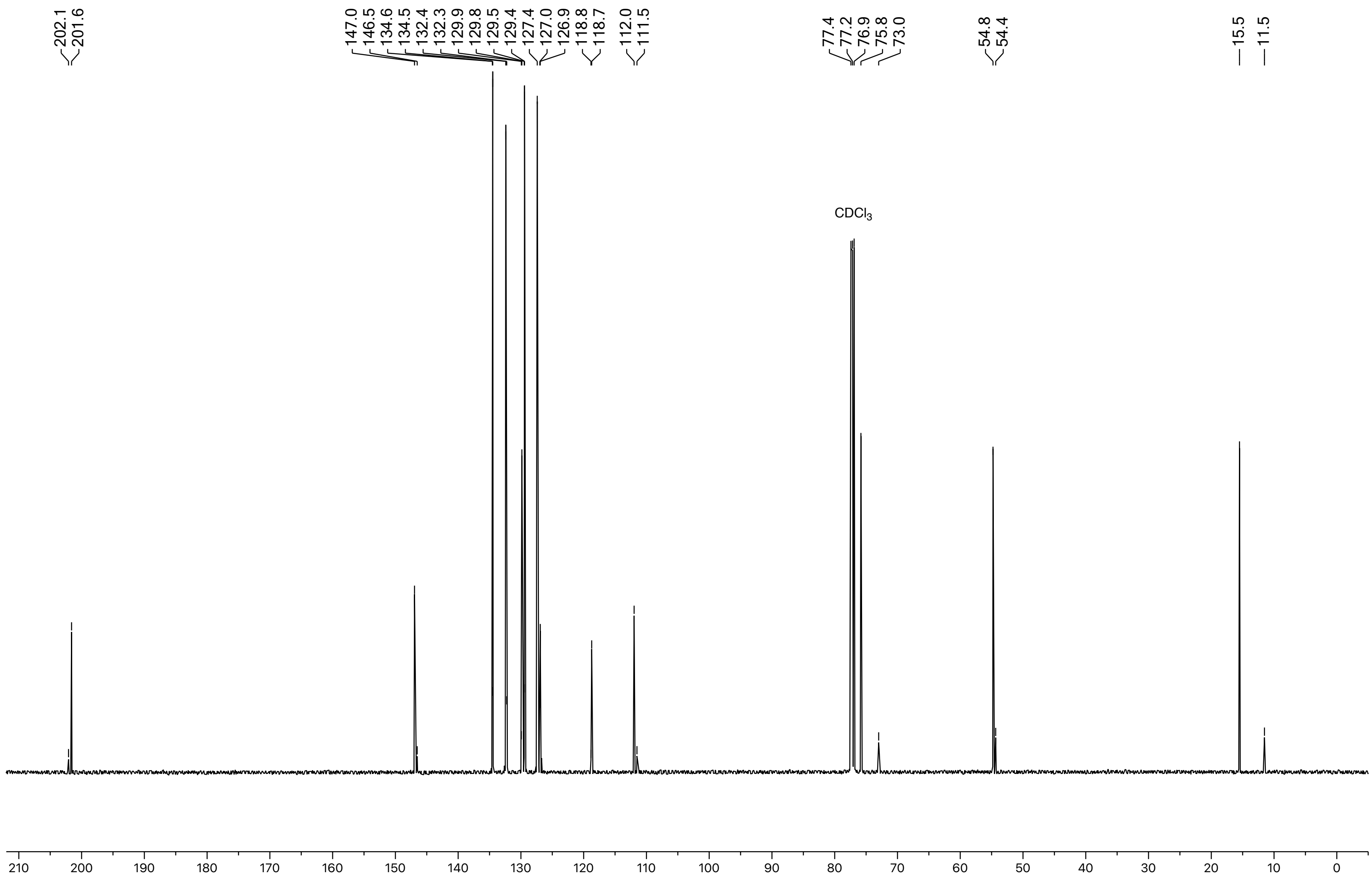

^{1}H NMR, 500 MHz, $CDCl_3$, **5f**

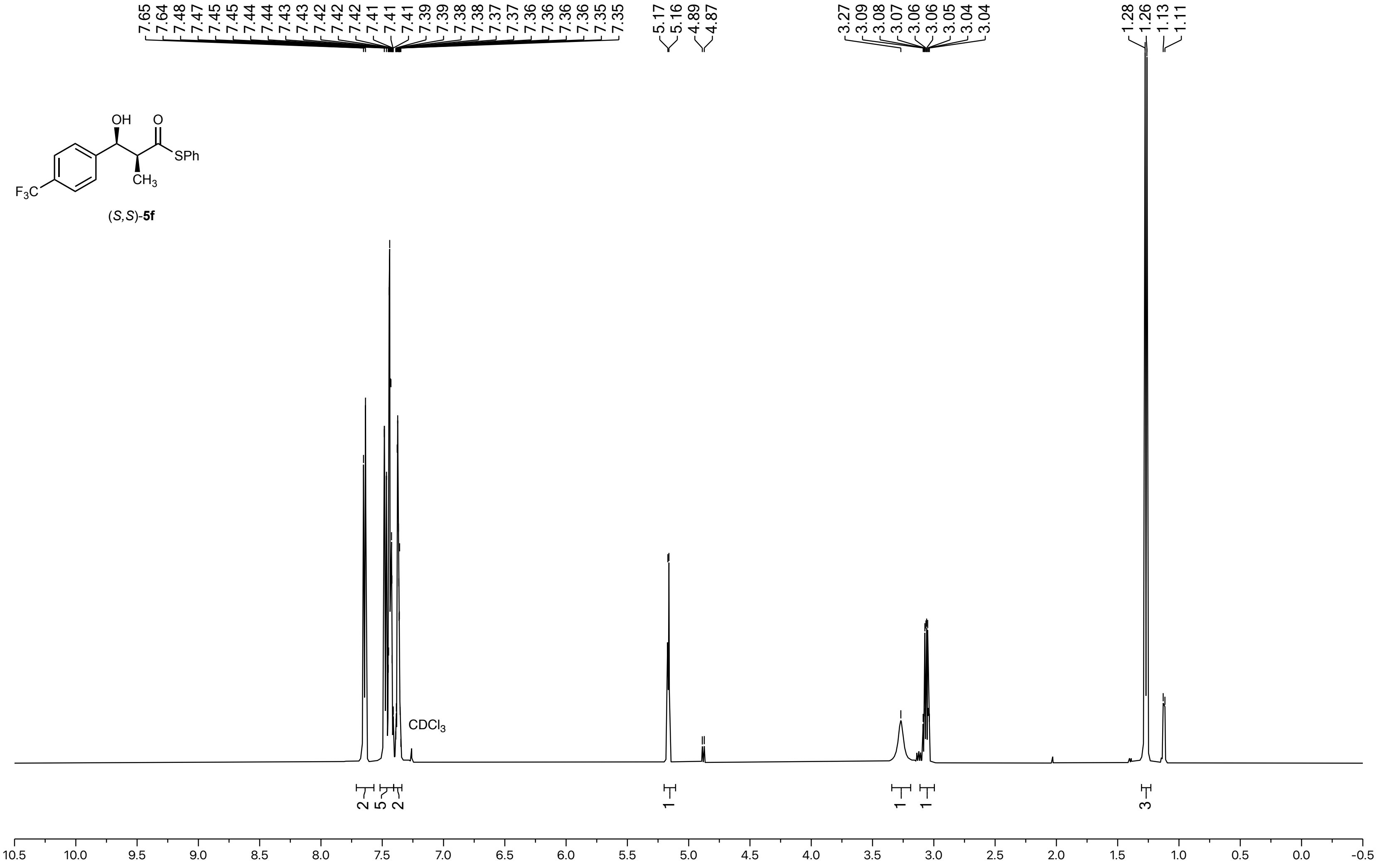

$^{13}C\{^{1}H\}$ NMR, 126 MHz, $CDCl_3$, **5f**

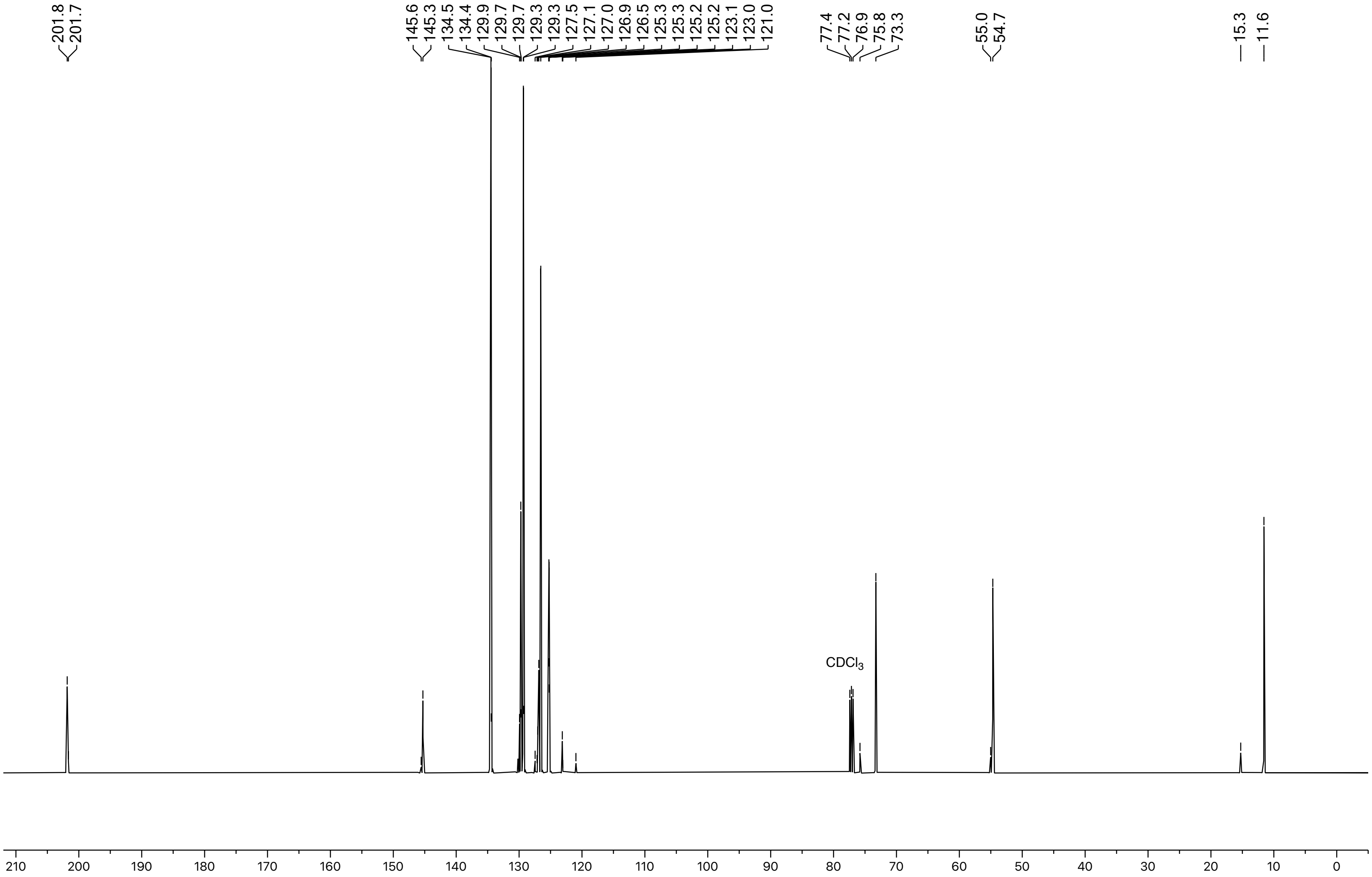

^{19}F NMR, 470 MHz, $CDCl_3$, **5f**

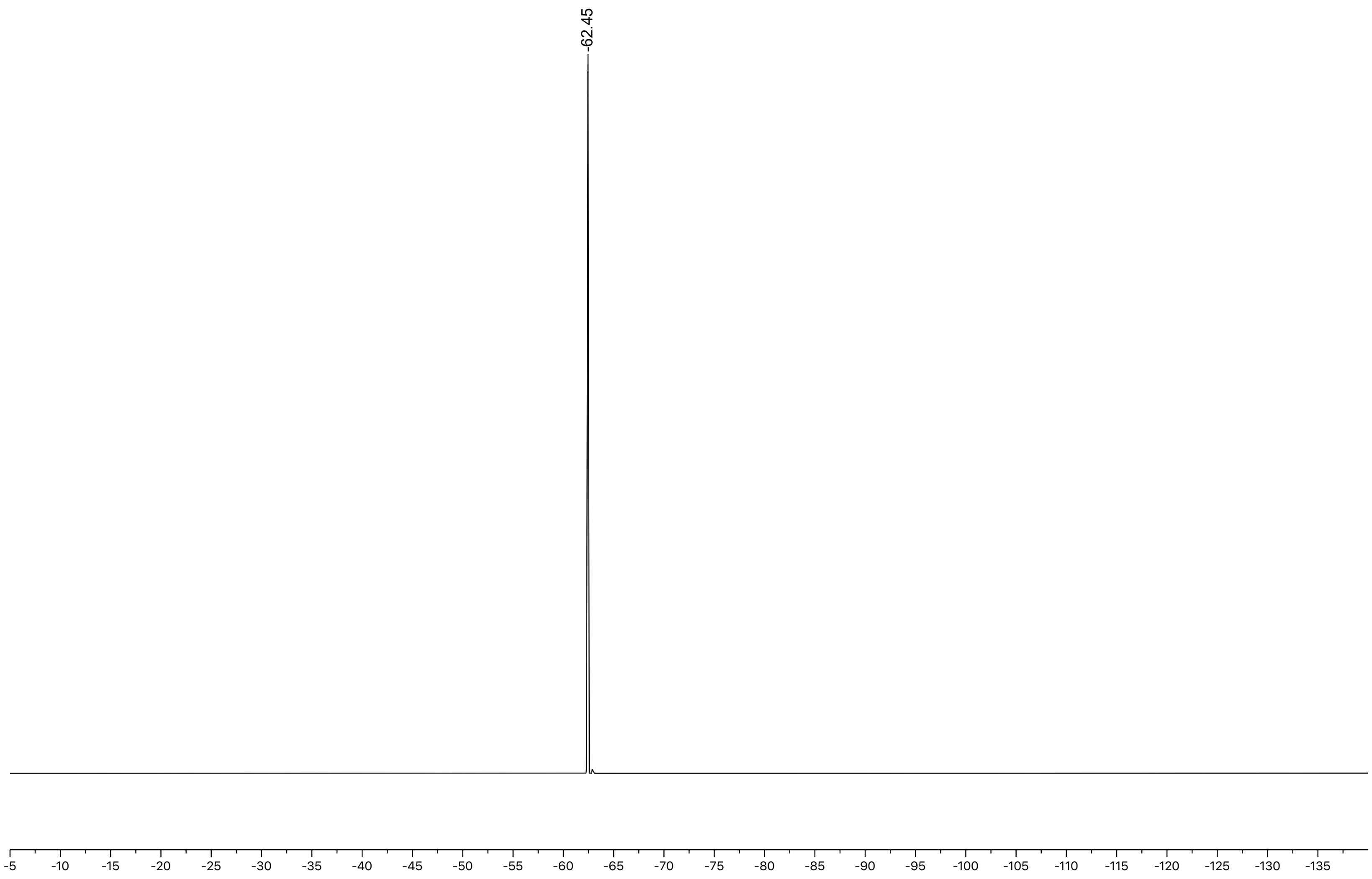

^{1}H NMR, 500 MHz, $CDCl_3$, **6f**

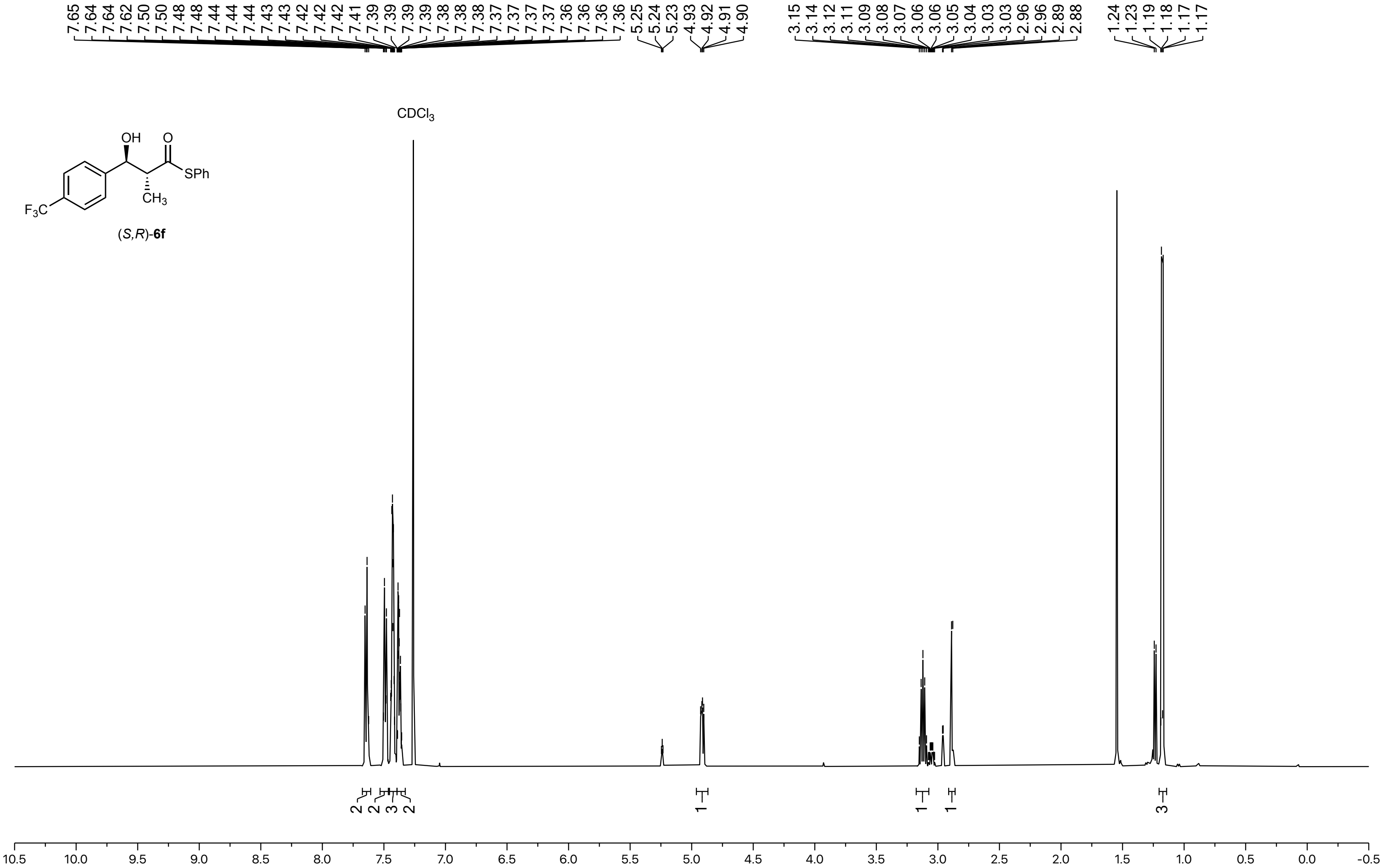

$^{13}C\{^{1}H\}$ NMR, 126 MHz, $CDCl_3$, **6f**

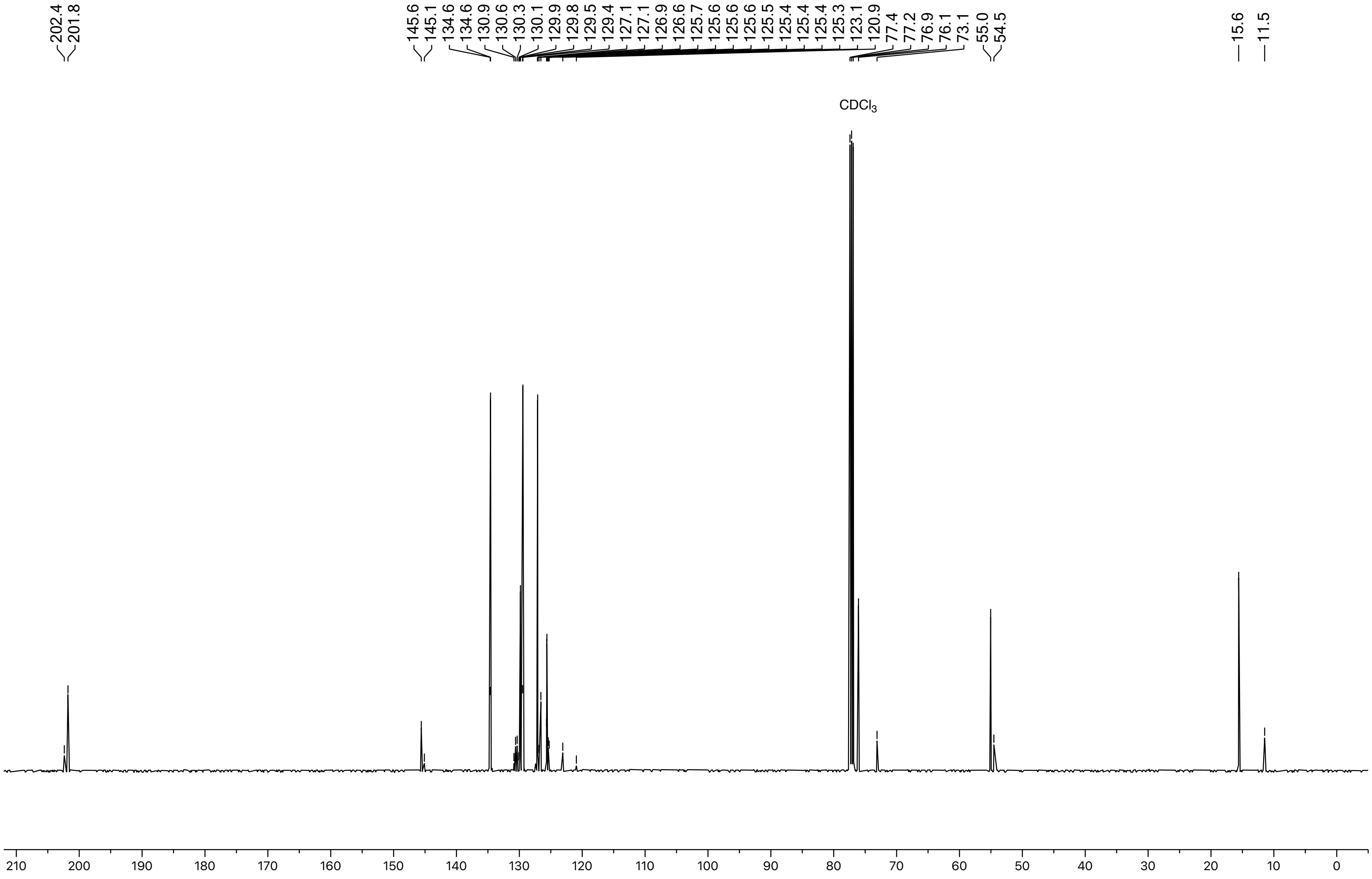

^{19}F NMR, 470 MHz, $CDCl_3$, **6f**

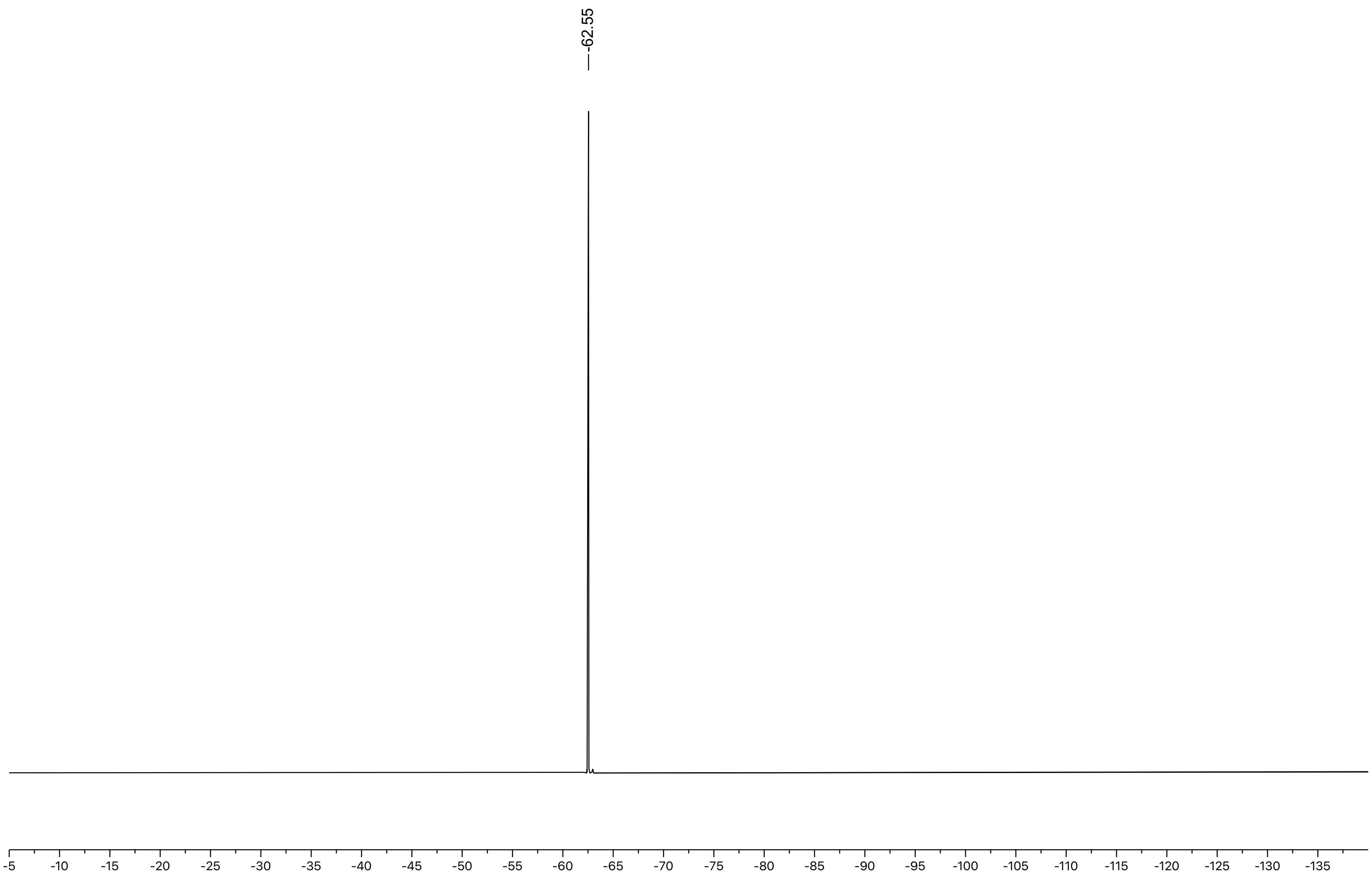

^1H NMR, 500 MHz, $CDCl_3$, **5g**

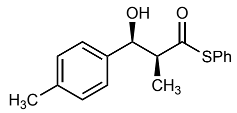

(*S*,*S*)-**5g**

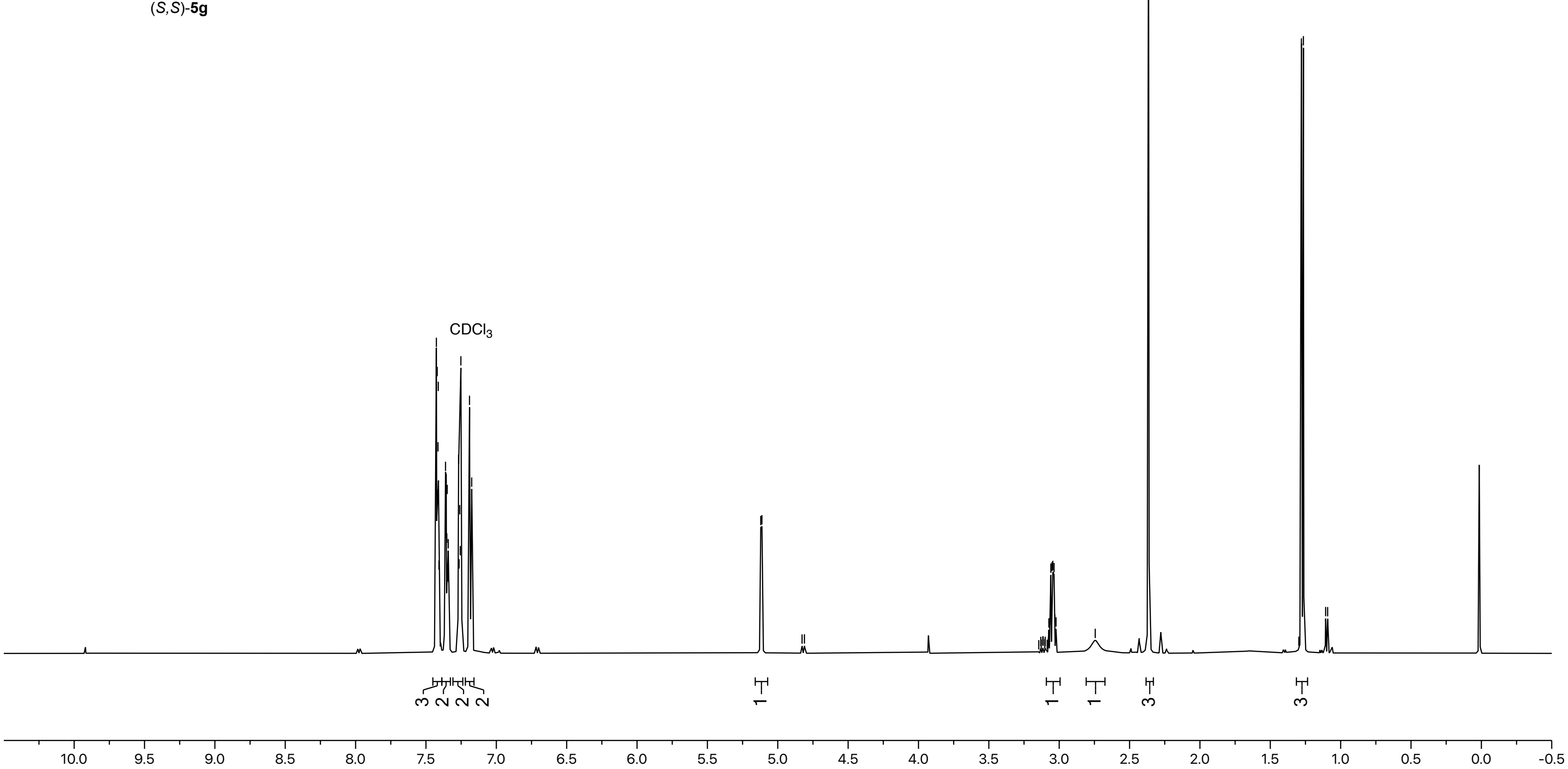

$^{13}C\{^{1}H\}$ NMR, 126 MHz, $CDCl_3$, **5g**

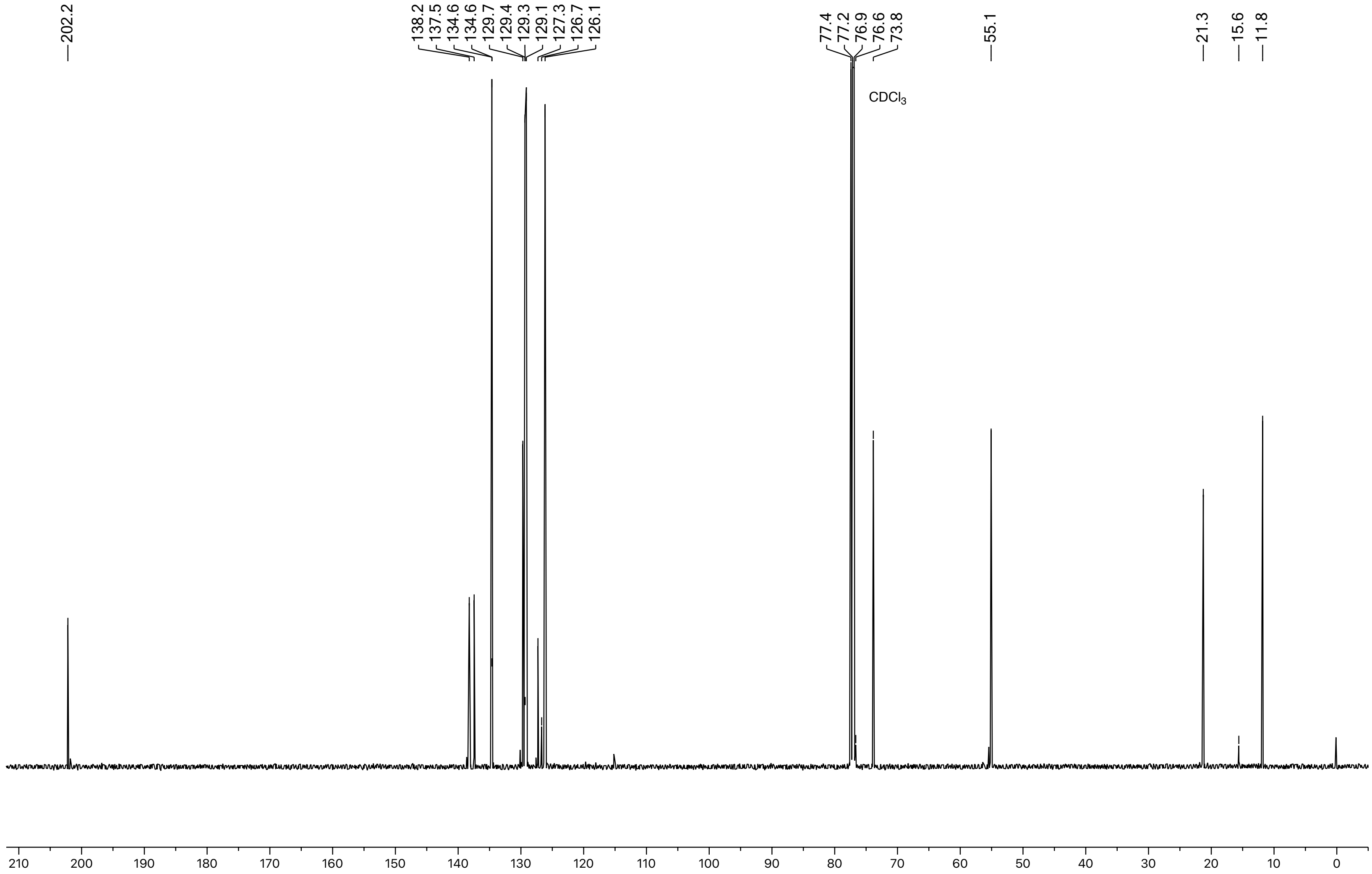

^{1}H NMR, 500 MHz, $CDCl_3$, **6g**

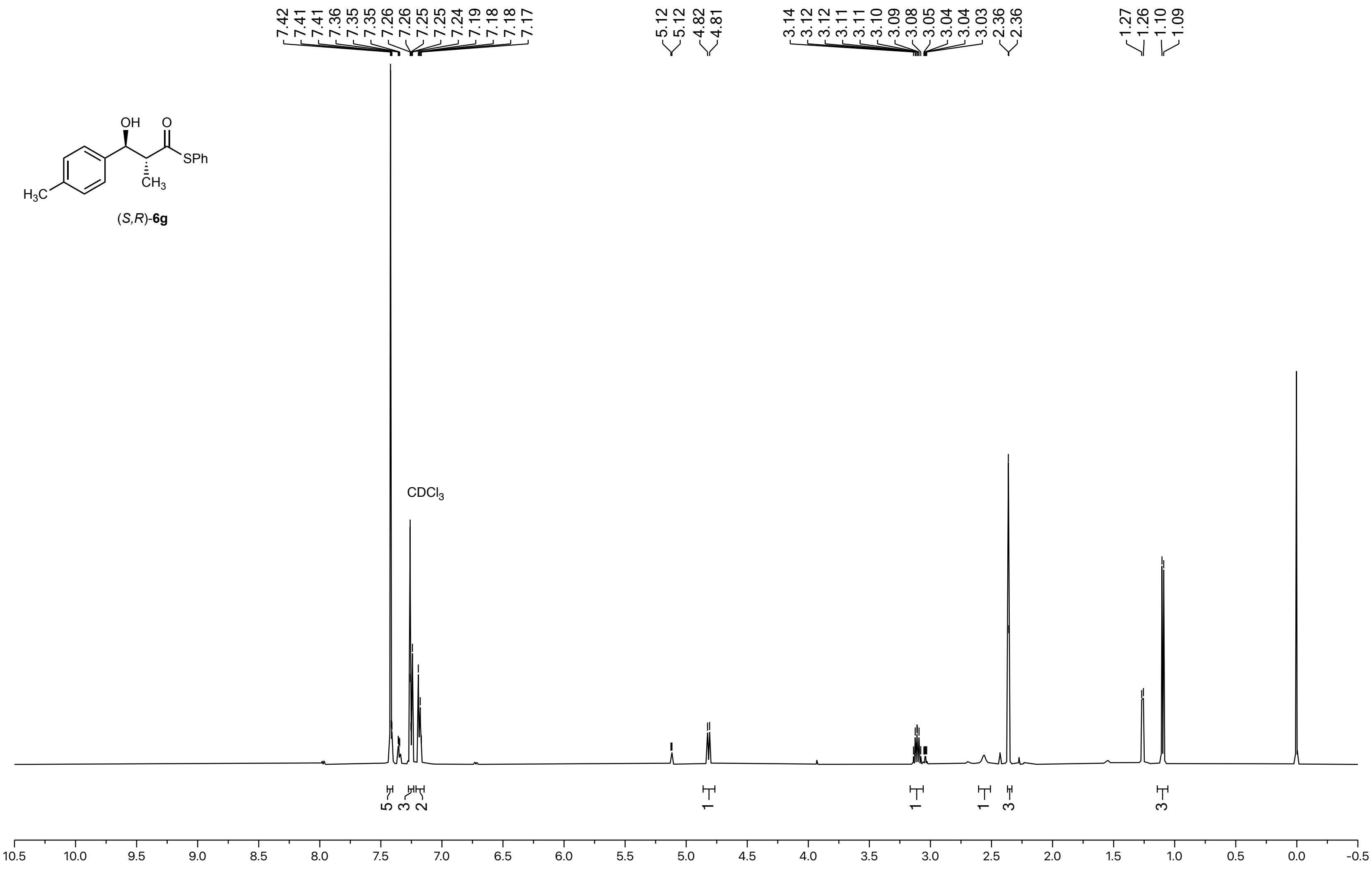

$^{13}C\{^1H\}$ NMR, 126 MHz, $CDCl_3$, **6g**

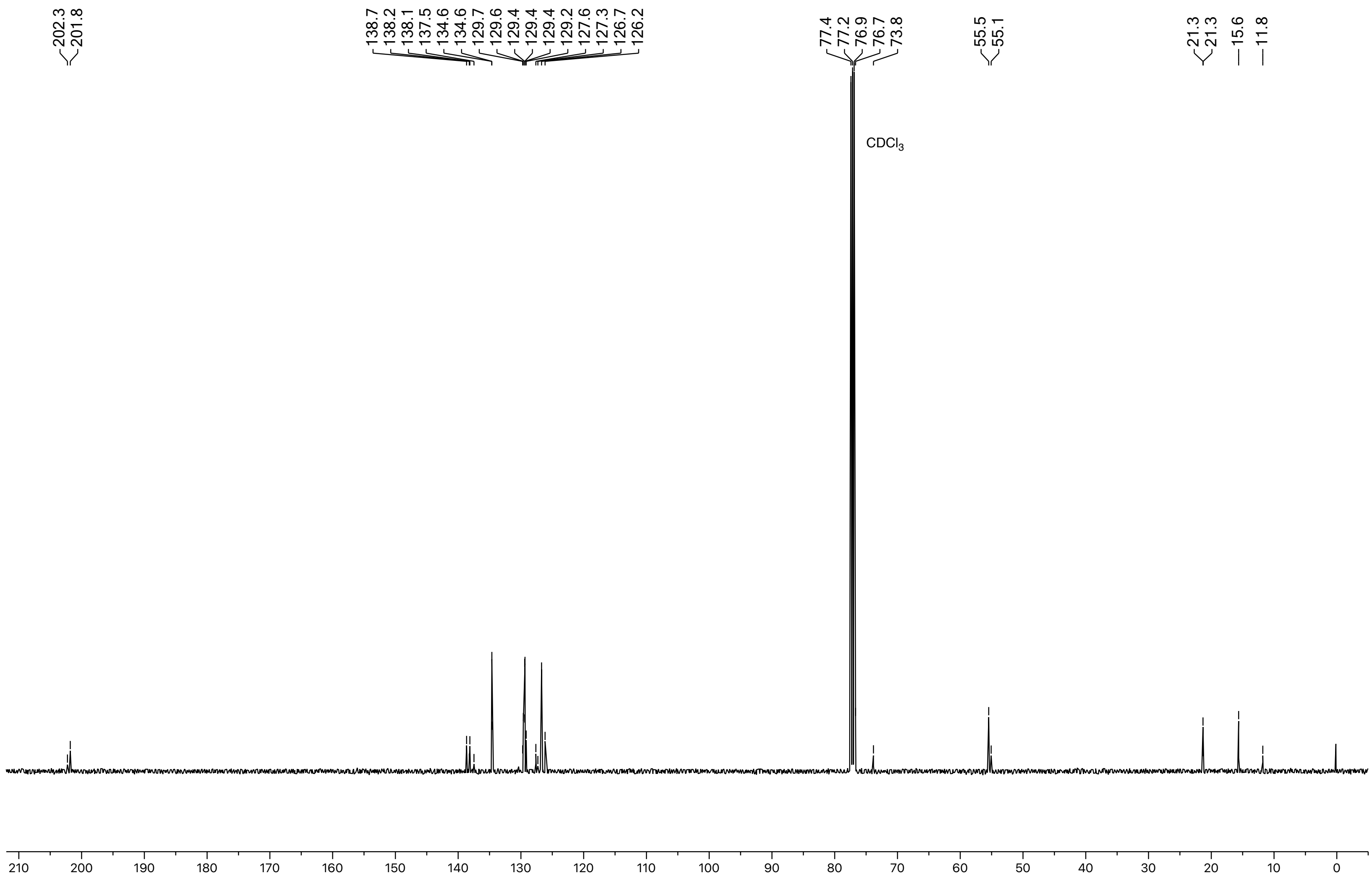

^1H NMR, 500 MHz, $CDCl_3$, **5h**

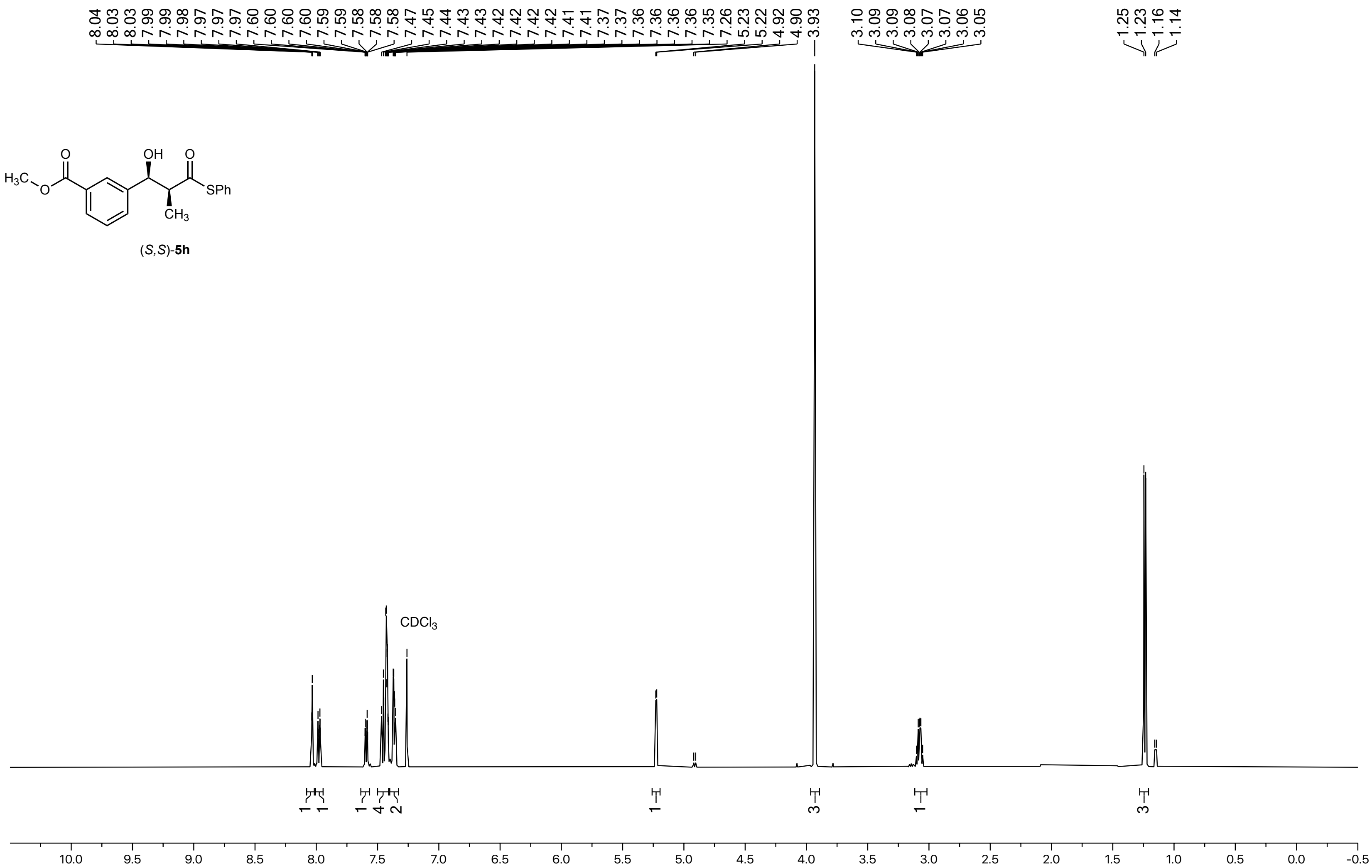

$^{13}C\{^1H\}$ NMR, 126 MHz, $CDCl_3$, **5h**

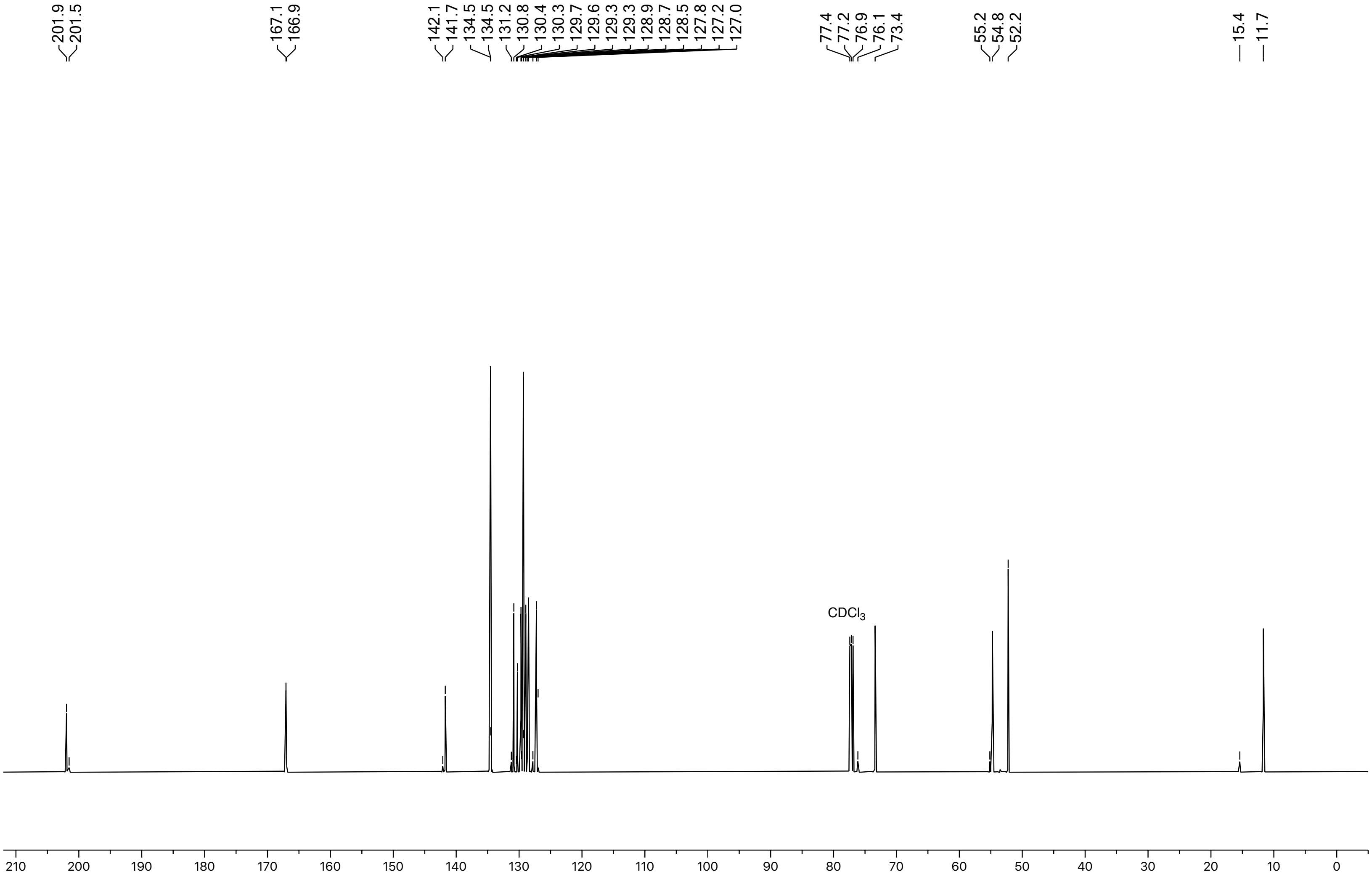

^{1}H NMR, 500 MHz, $CDCl_3$, **6h**

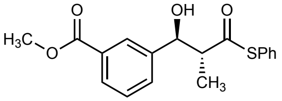

(*S*,*R*)-**6h**

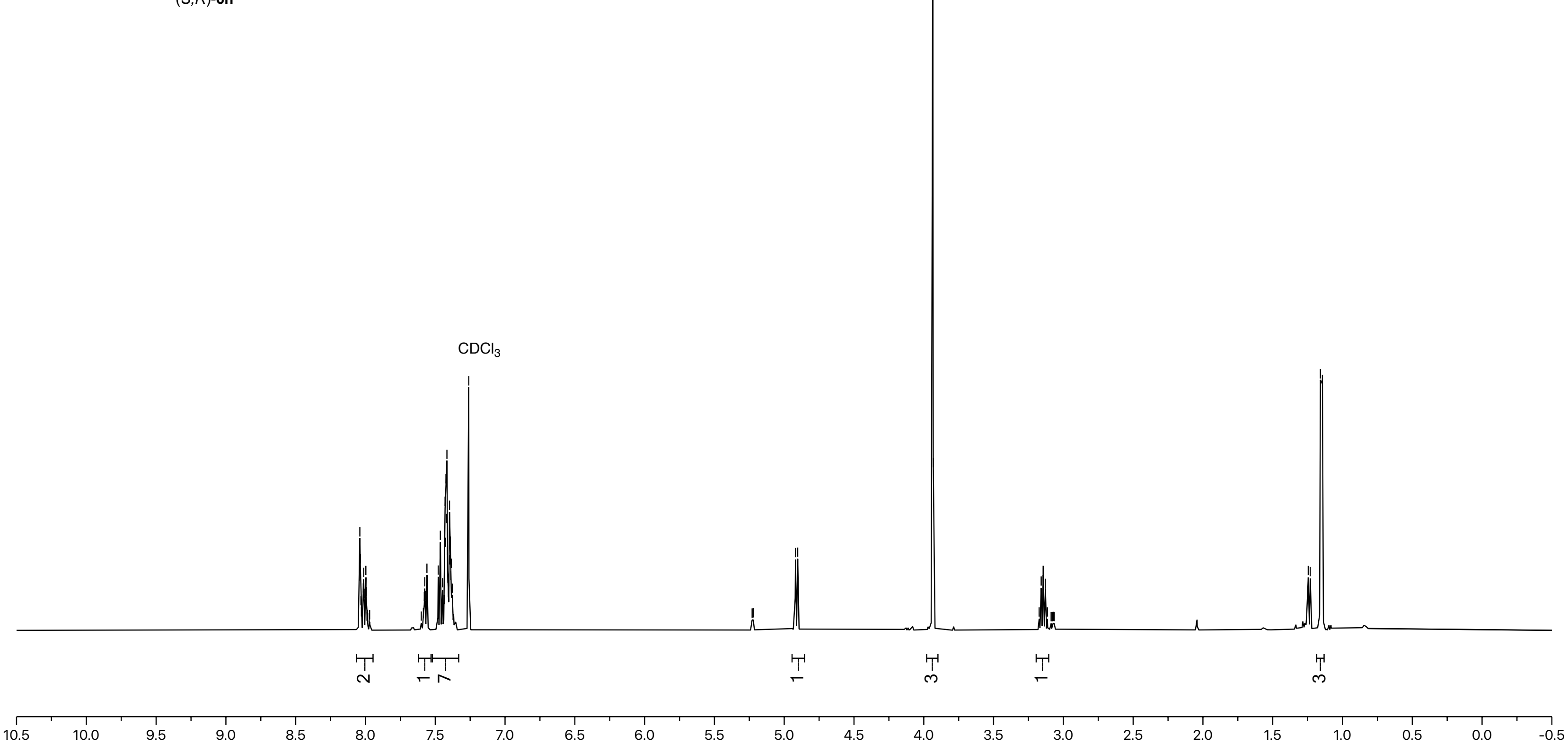

$^{13}C\{^{1}H\}$ NMR, 126 MHz, $CDCl_3$, **6h**

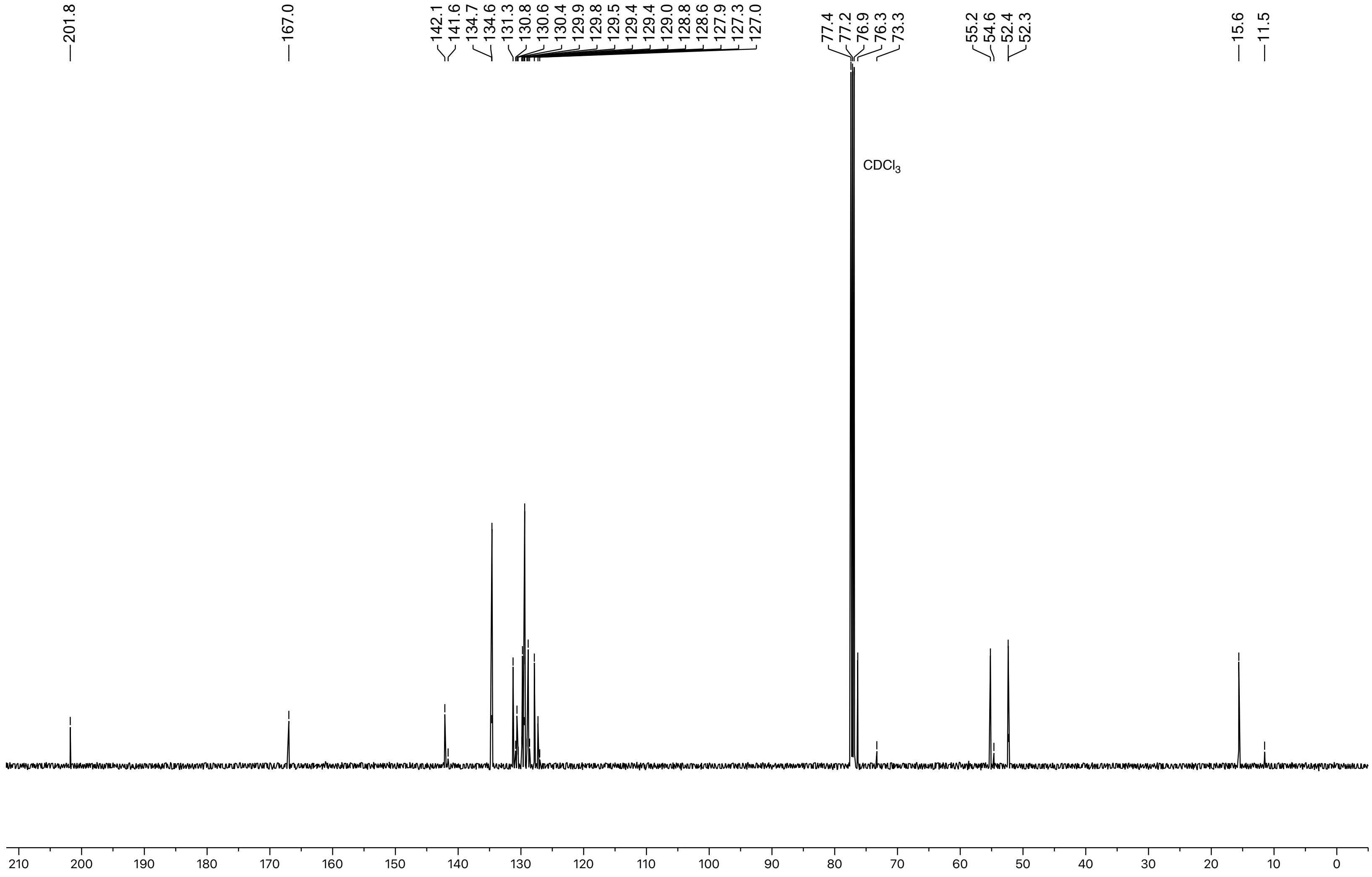

^{1}H NMR, 500 MHz, $CDCl_3$, **5i**

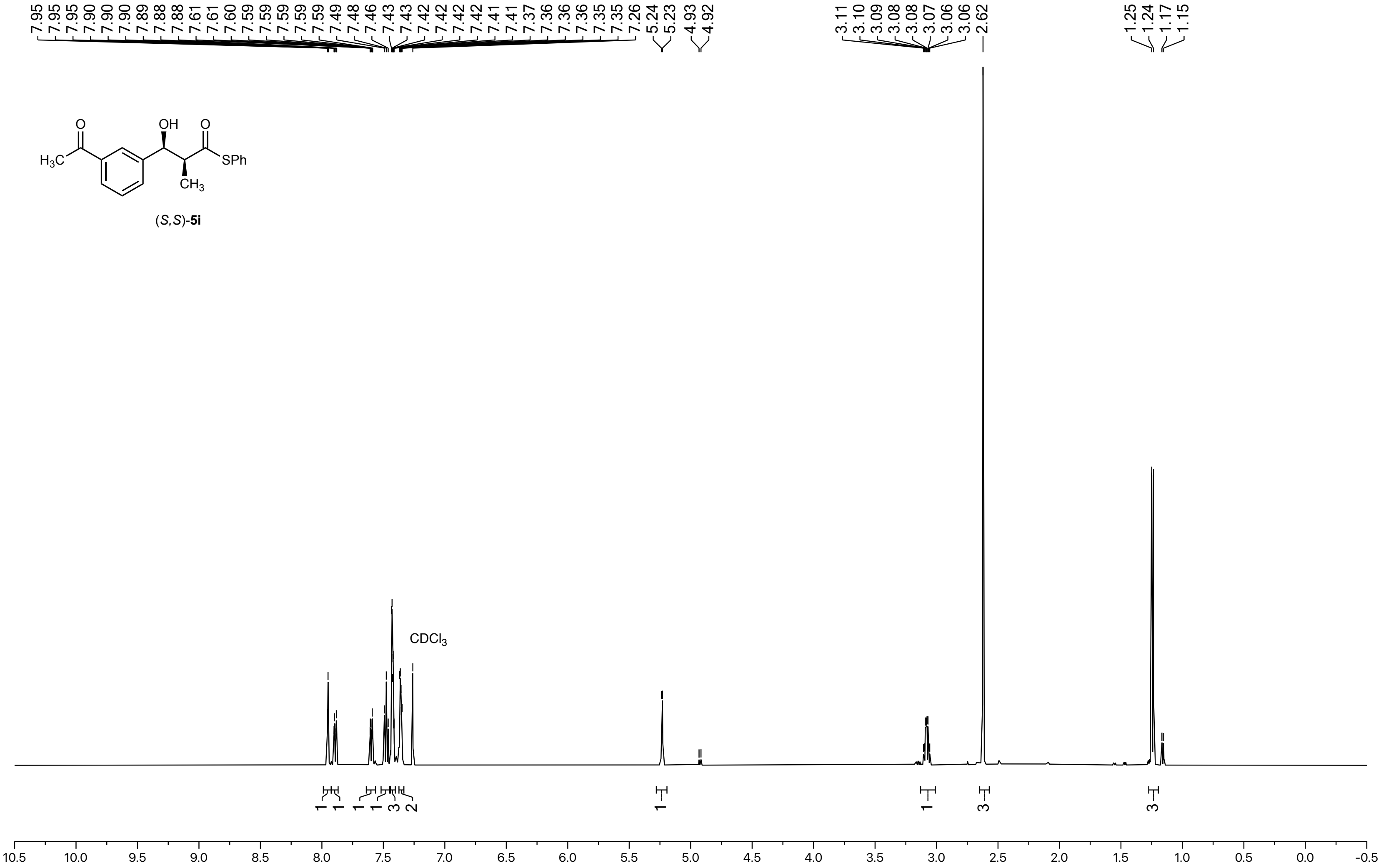

(*S*,*S*)-**5i**

$^{13}C\{^1H\}$ NMR, 126 MHz, $CDCl_3$, **5i**

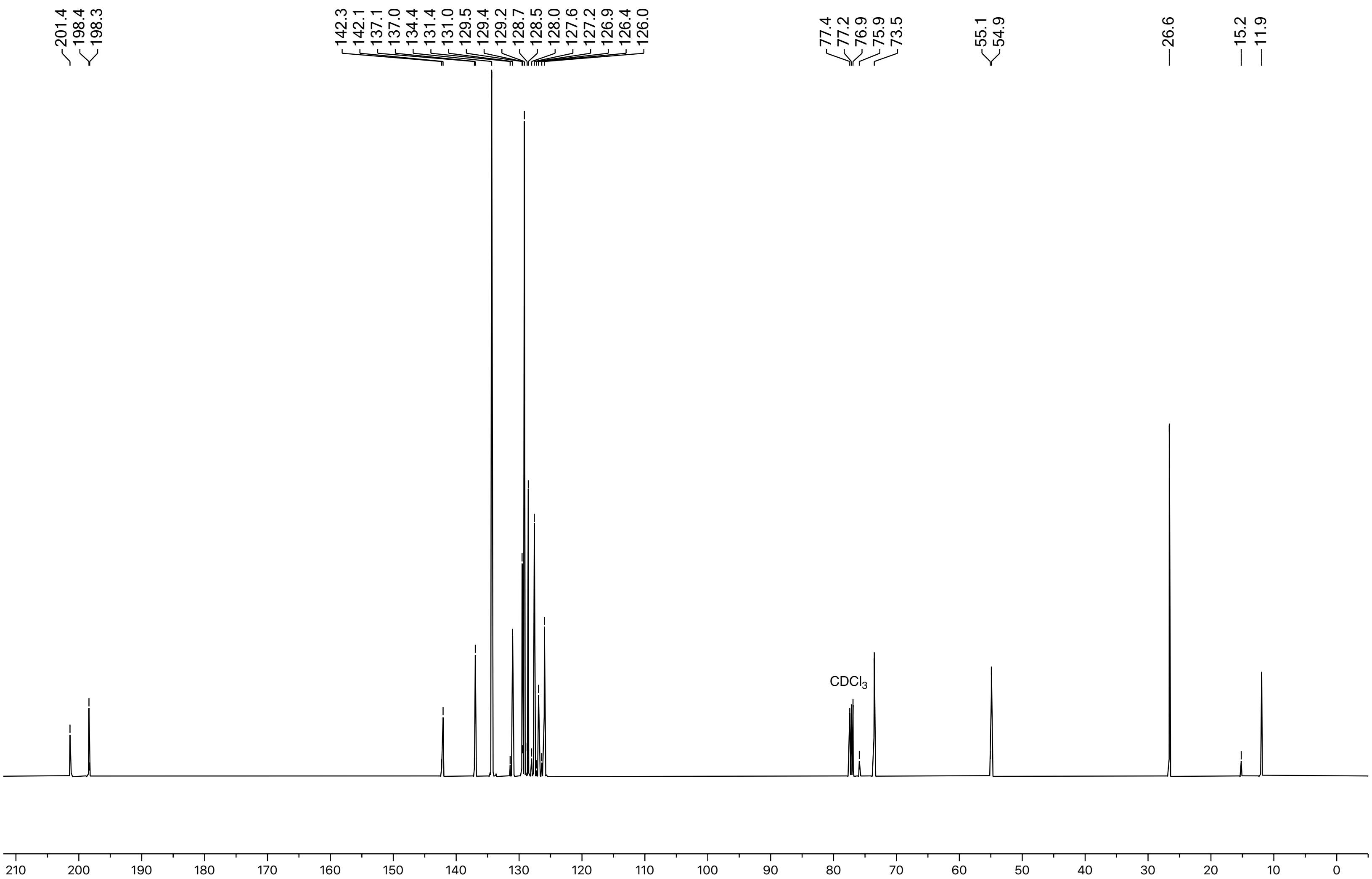

^{1}H NMR, 500 MHz, $CDCl_3$, **6i**

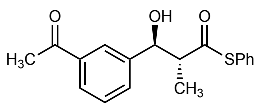

(*S*,*R*)-**6i**

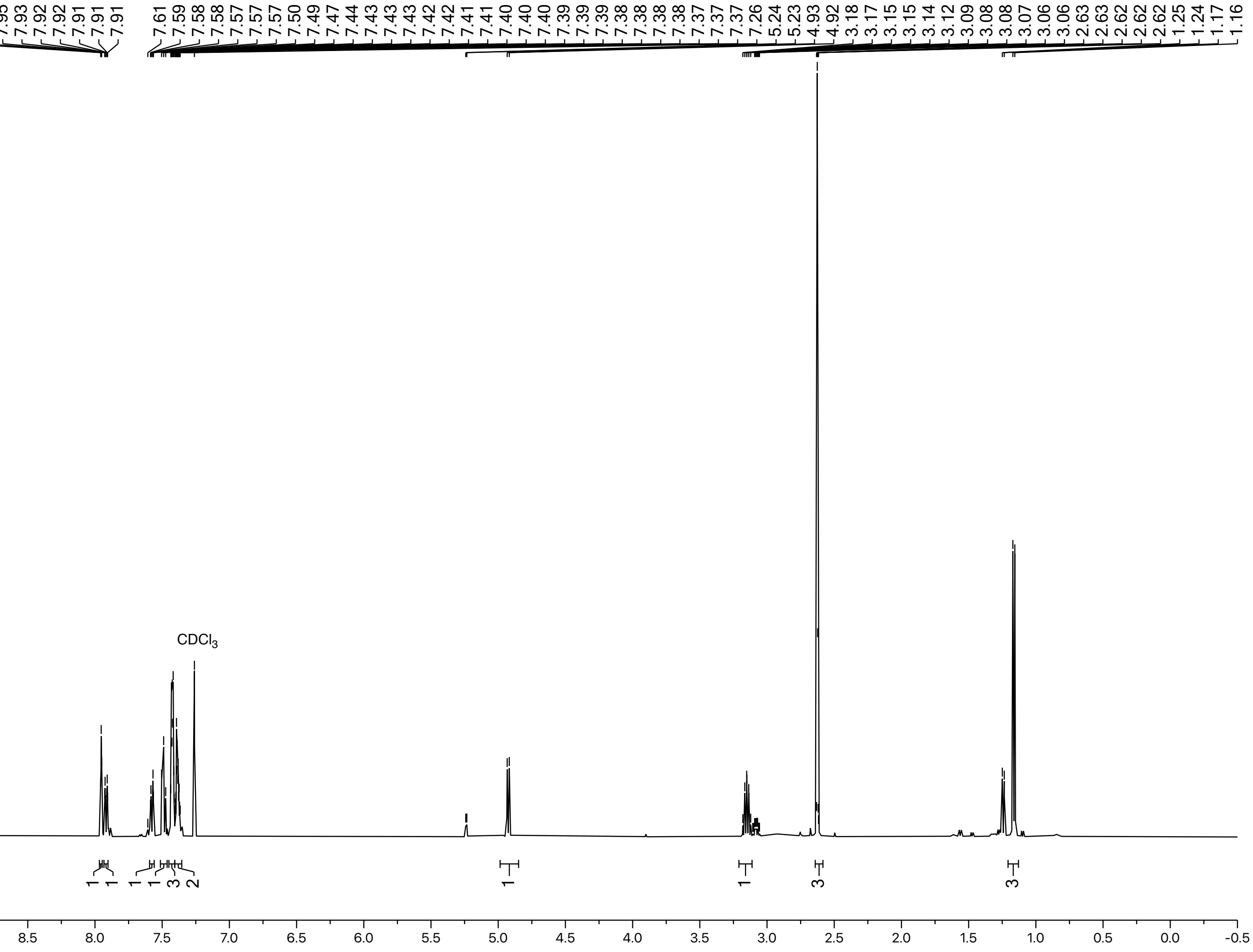

$^{13}C\{^{1}H\}$ NMR, 126 MHz, $CDCl_3$, **6i**

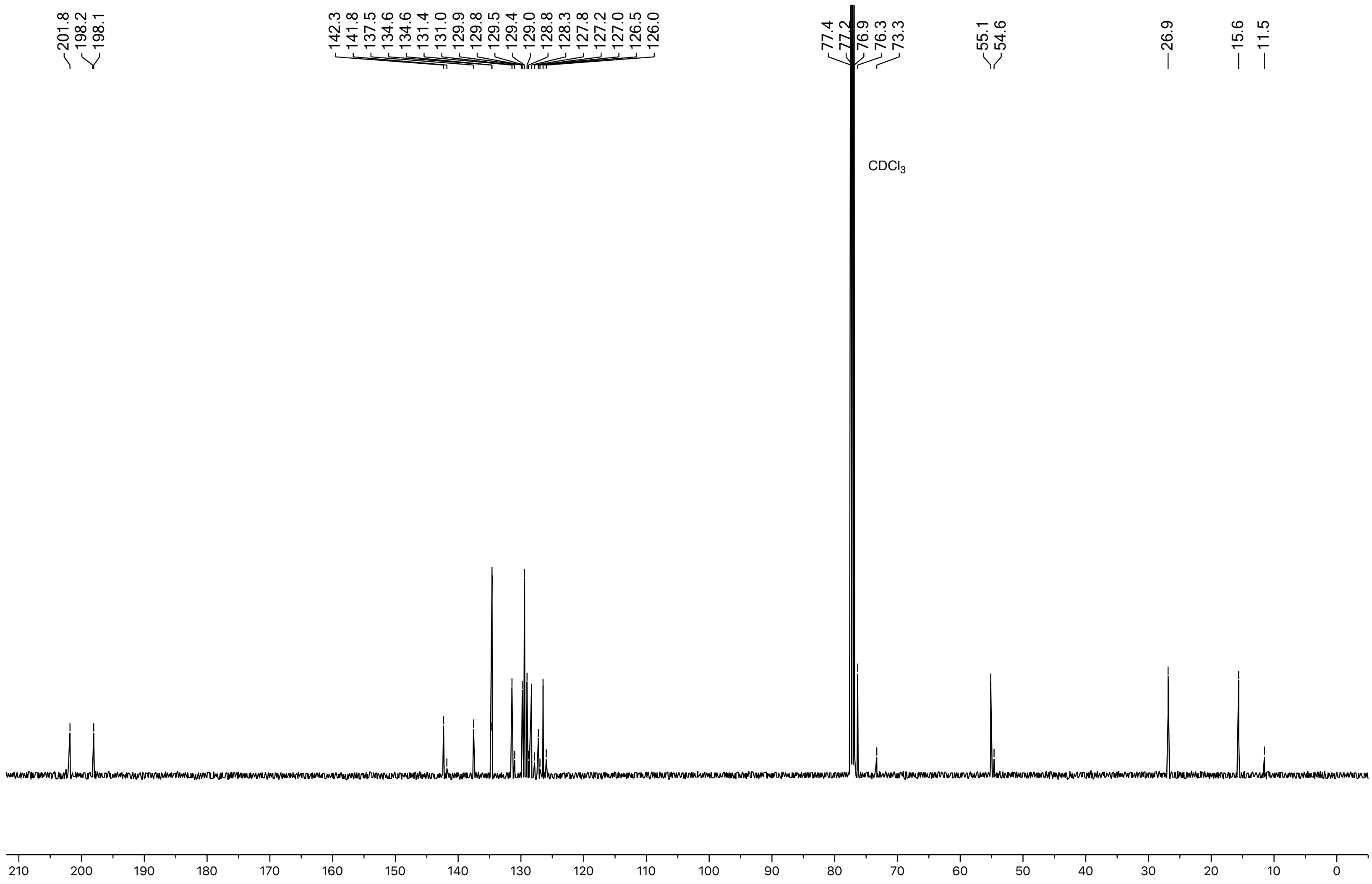

^1H NMR, 500 MHz, $CDCl_3$, **5j**

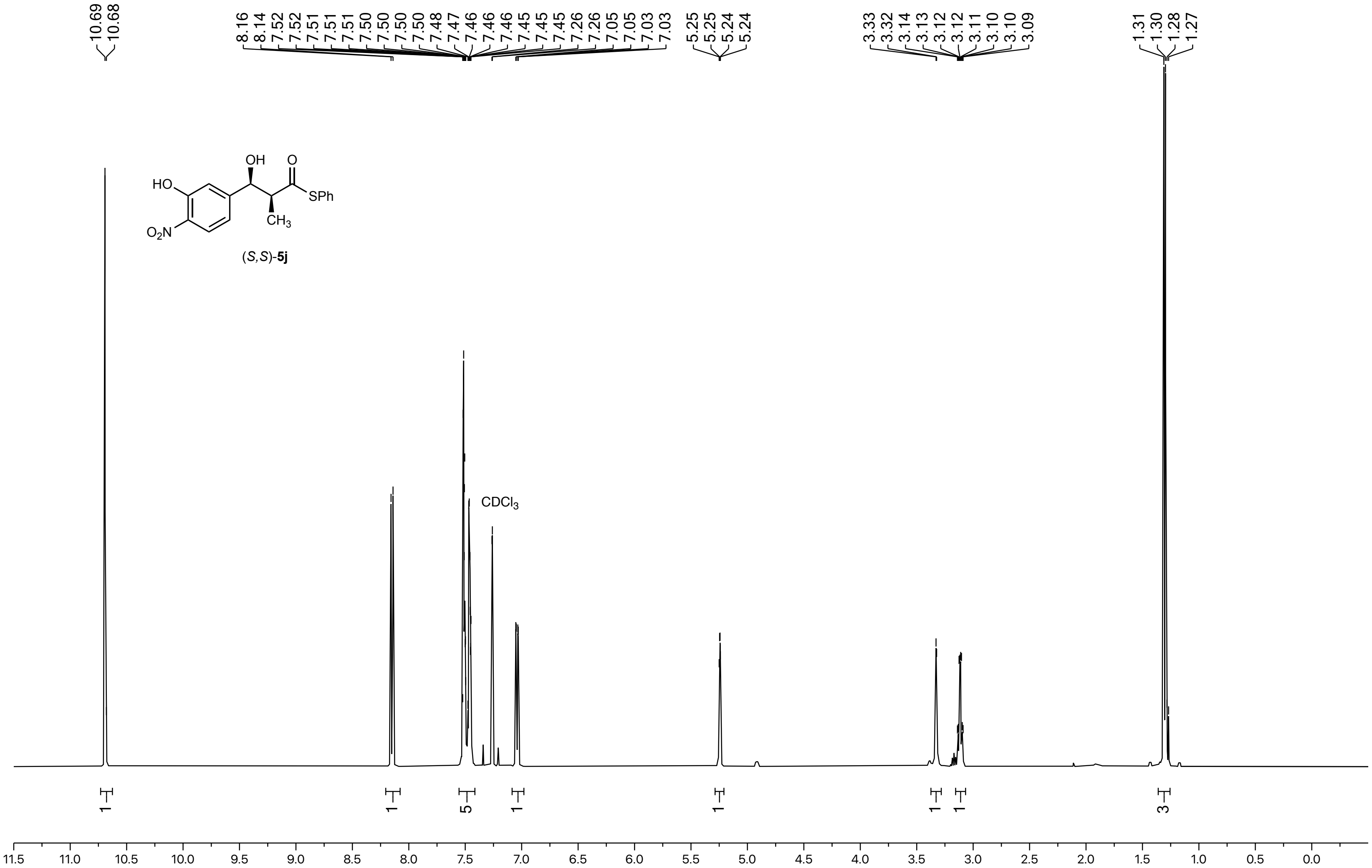

$^{13}C\{^{1}H\}$ NMR, 126 MHz, $CDCl_3$, **5j**

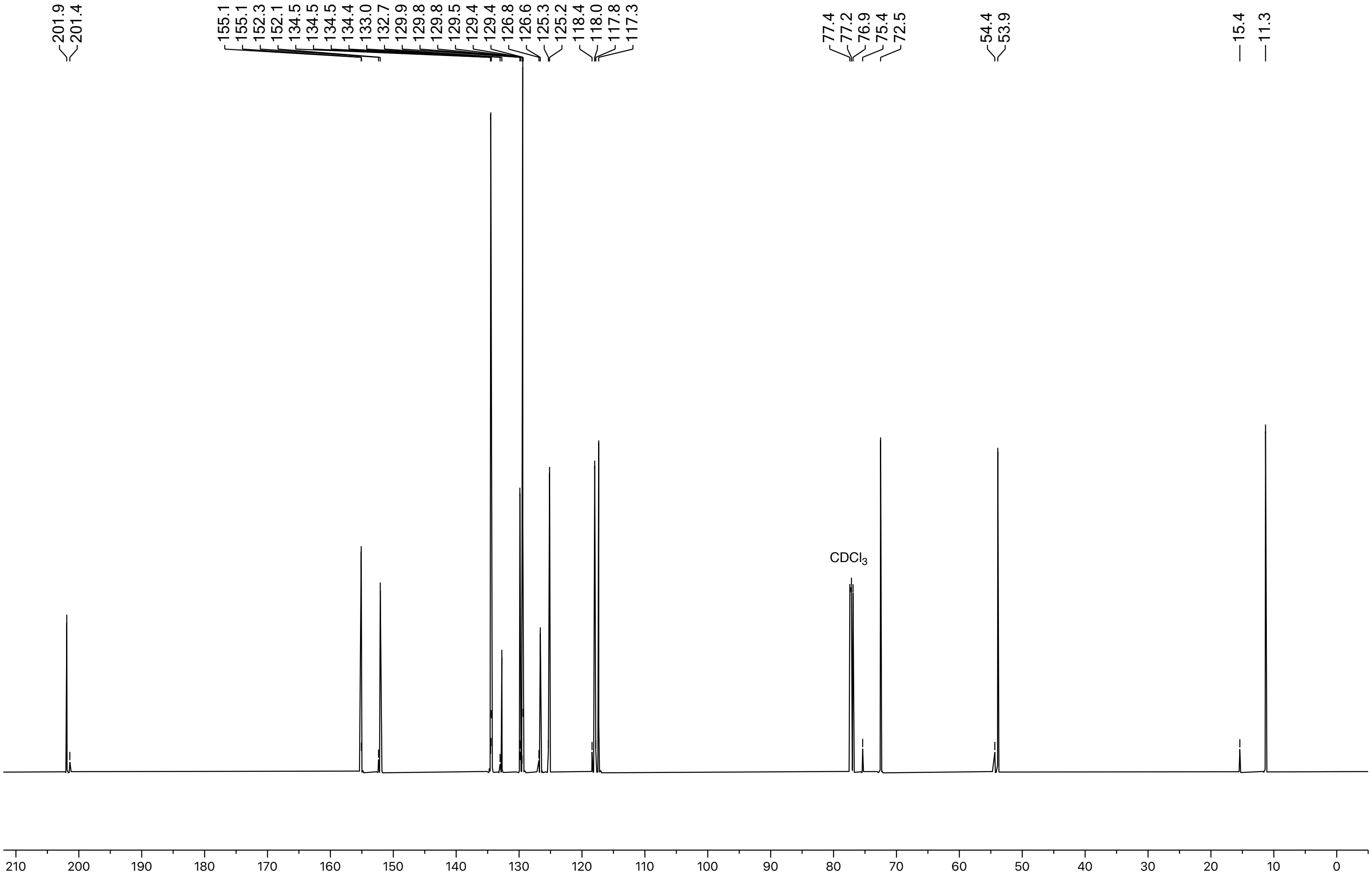

^{1}H NMR, 500 MHz, $CDCl_3$, **6j**

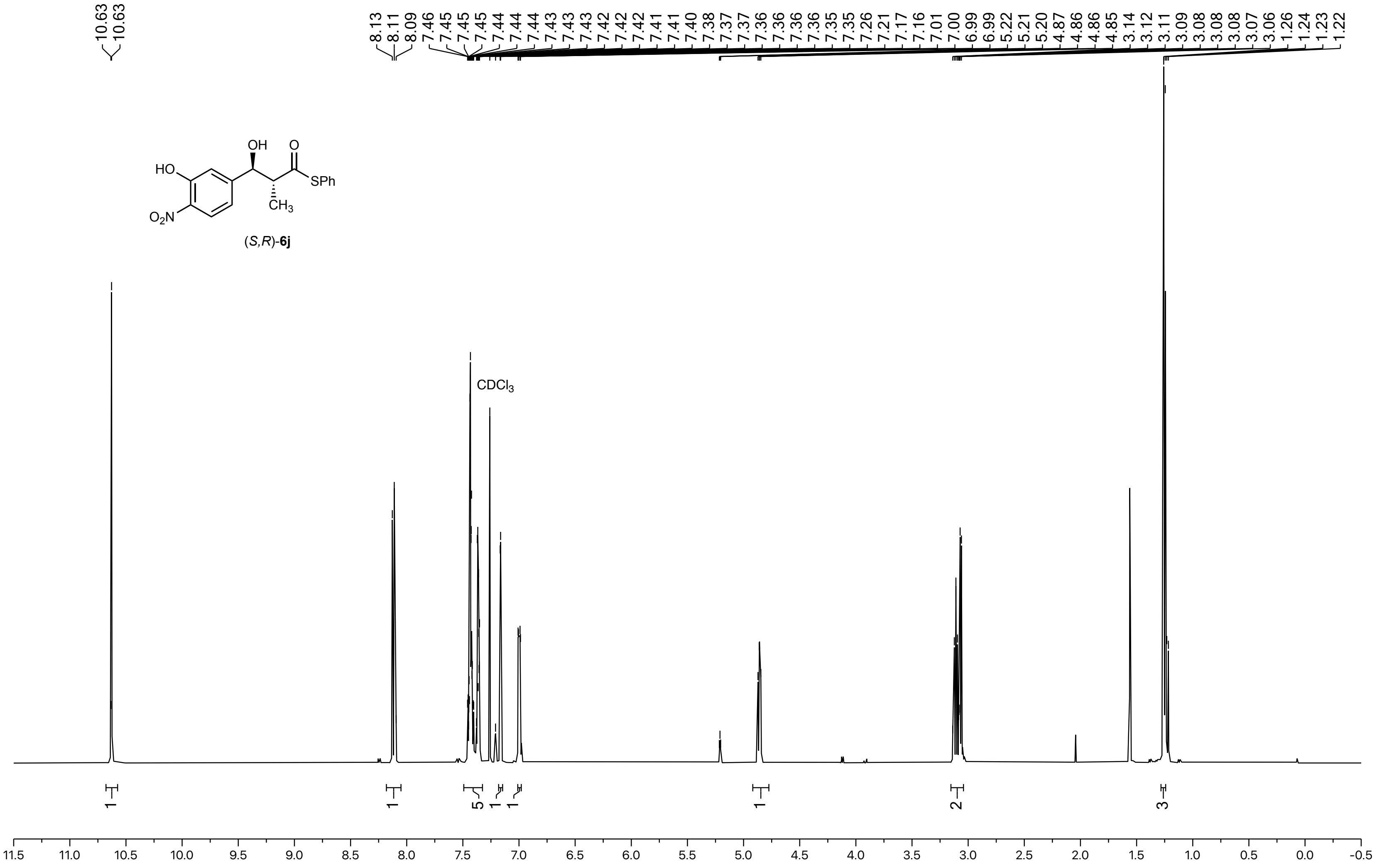

$^{13}C\{^{1}H\}$ NMR, 126 MHz, $CDCl_3$, **6j**

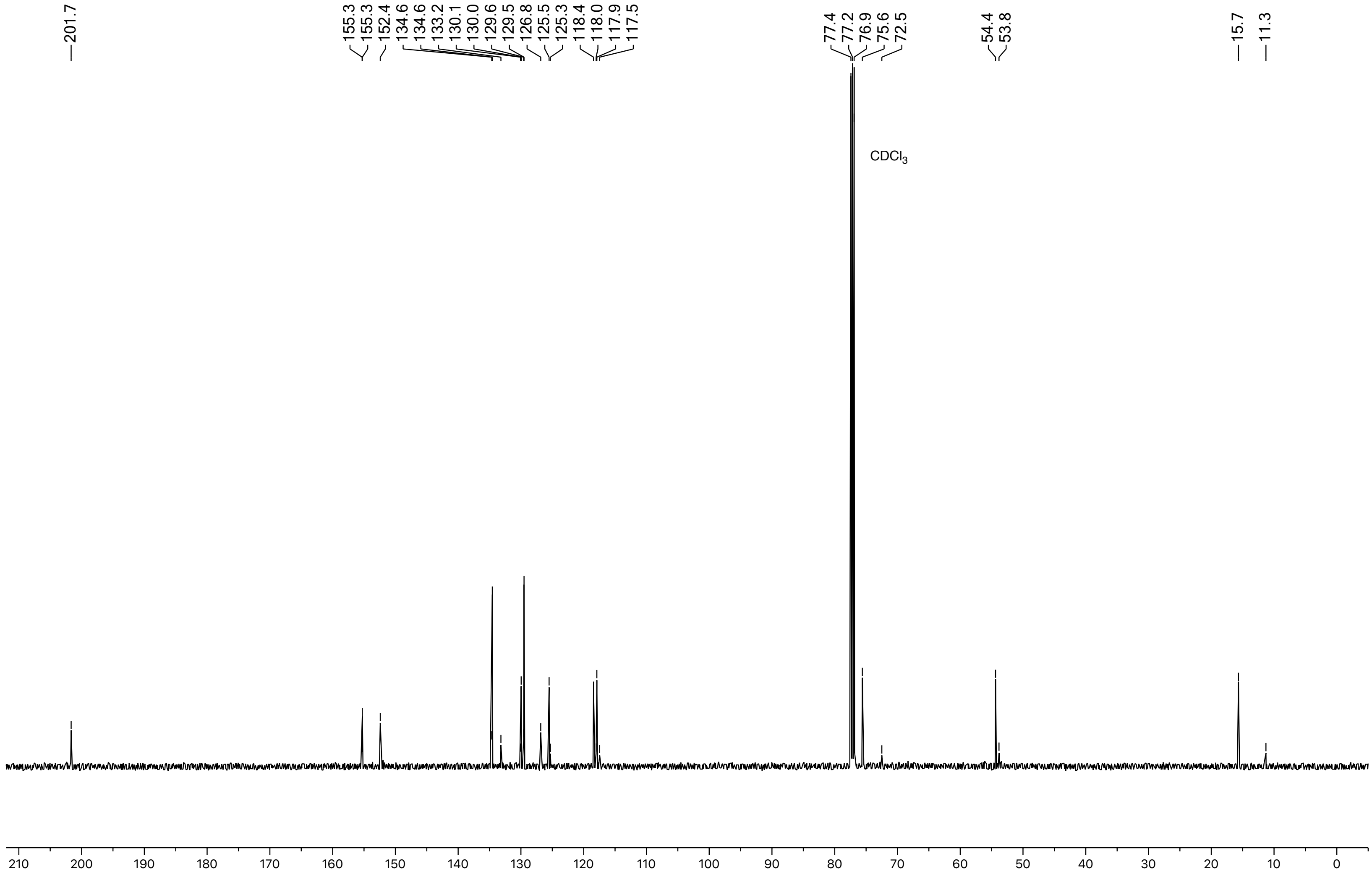

^{1}H NMR, 500 MHz, $CDCl_3$, **5k**

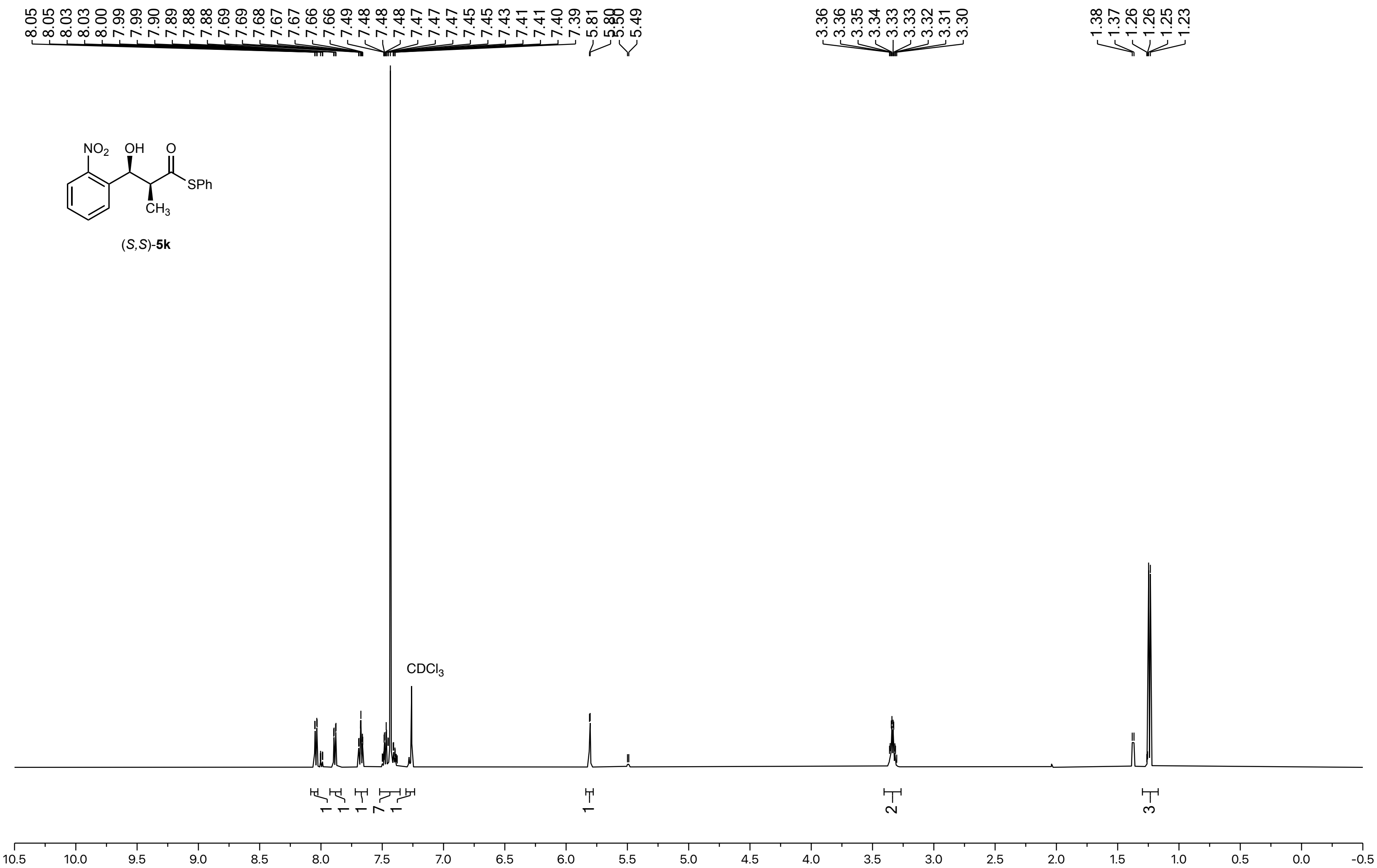

$^{13}C\{^{1}H\}$ NMR, 126 MHz, $CDCl_3$, **5k**

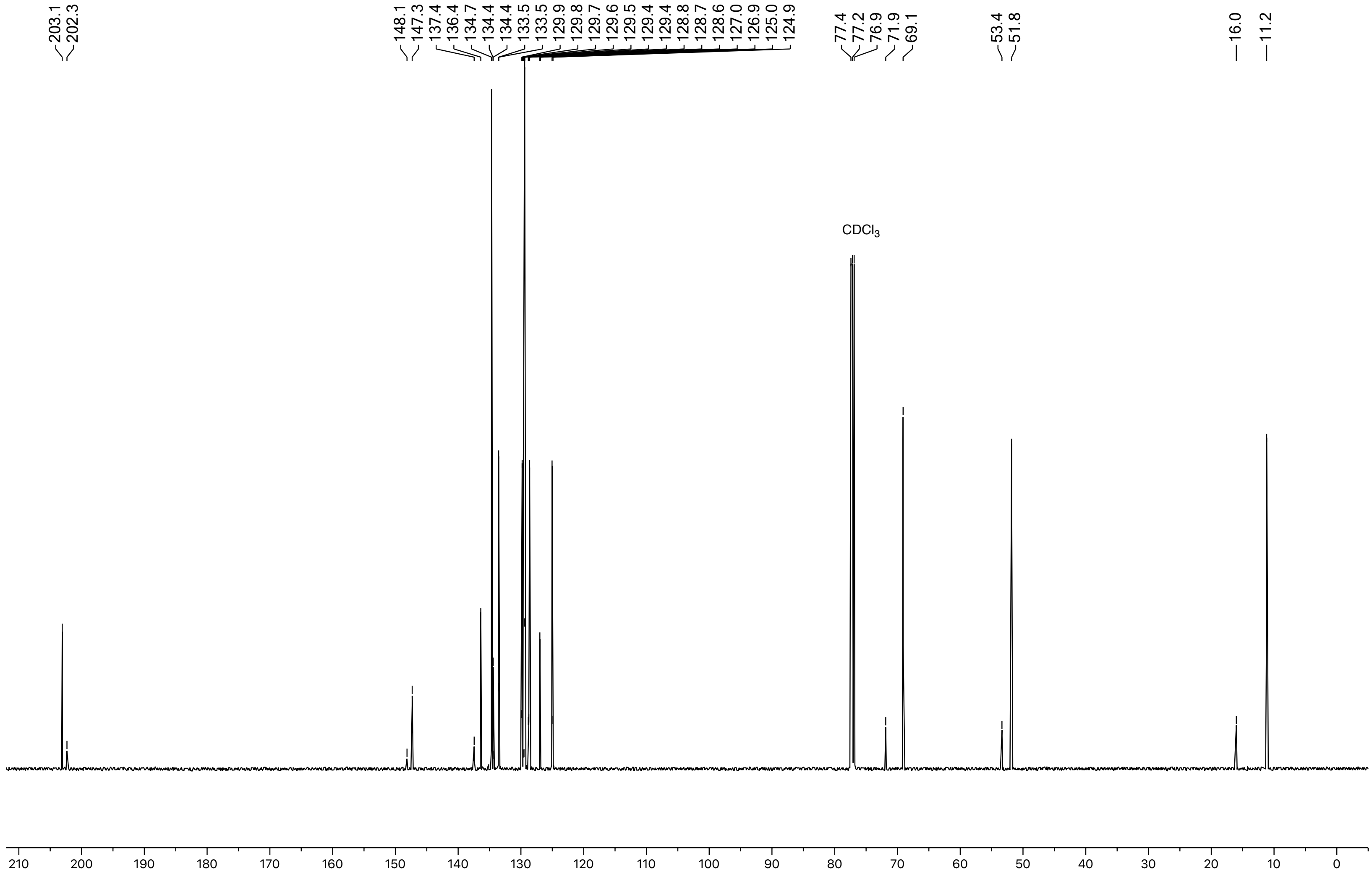

^{1}H NMR, 500 MHz, $CDCl_3$, **6k**

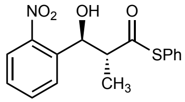

(*S*,*R*)-**6k**

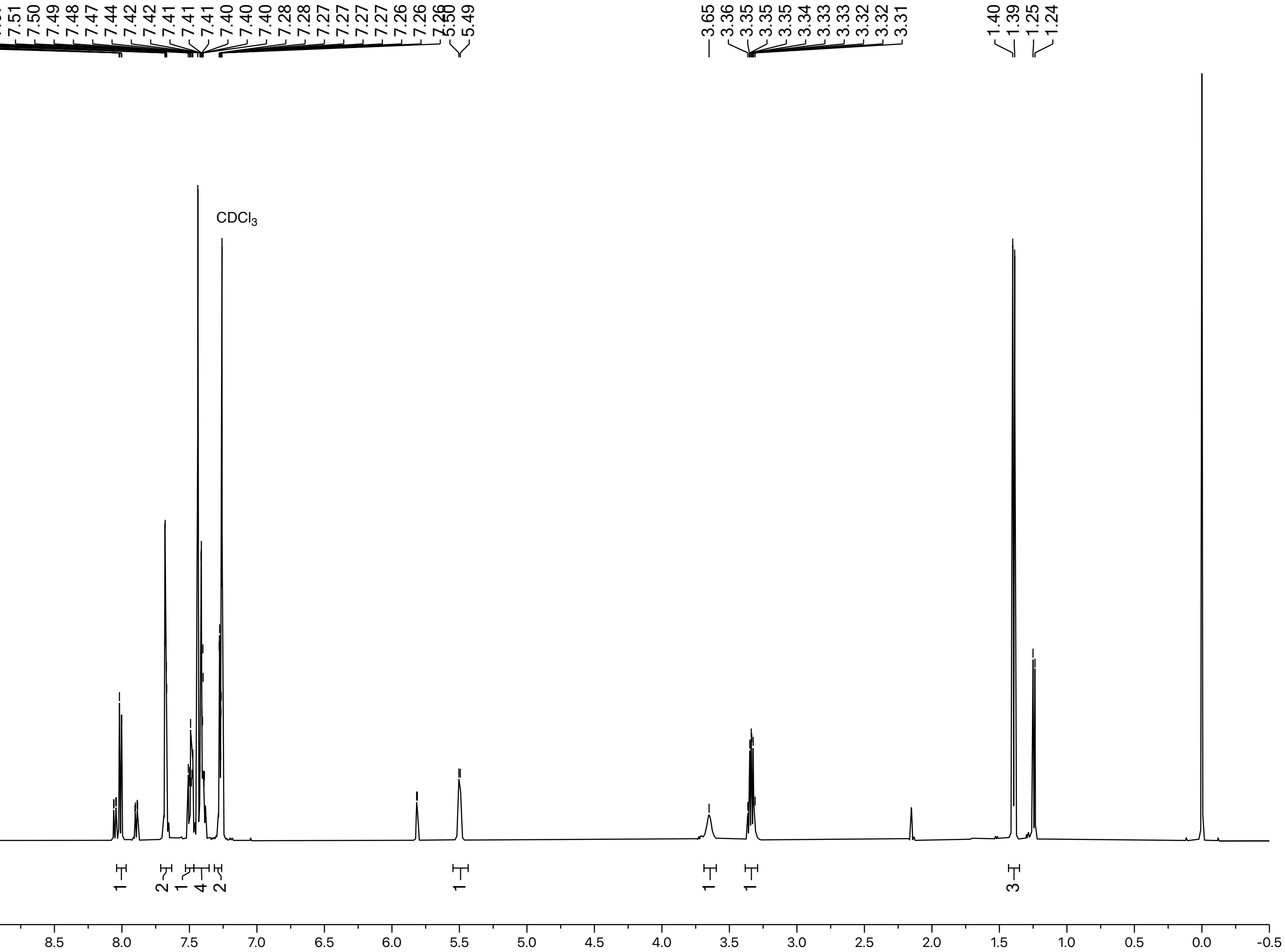

$^{13}C\{^{1}H\}$ NMR, 126 MHz, $CDCl_3$, **6k**

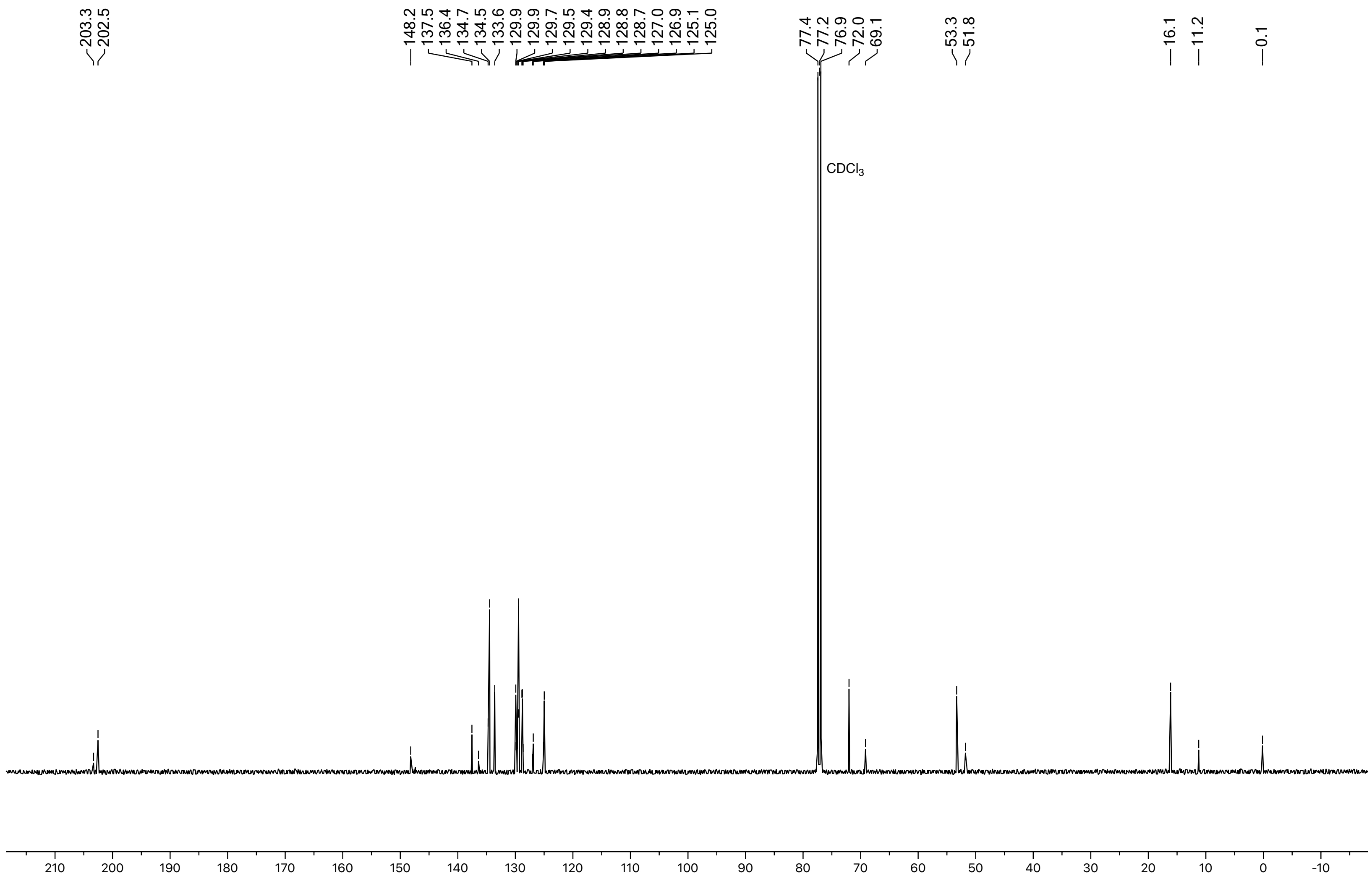

^{1}H NMR, 500 MHz, $CDCl_3$, **5l**

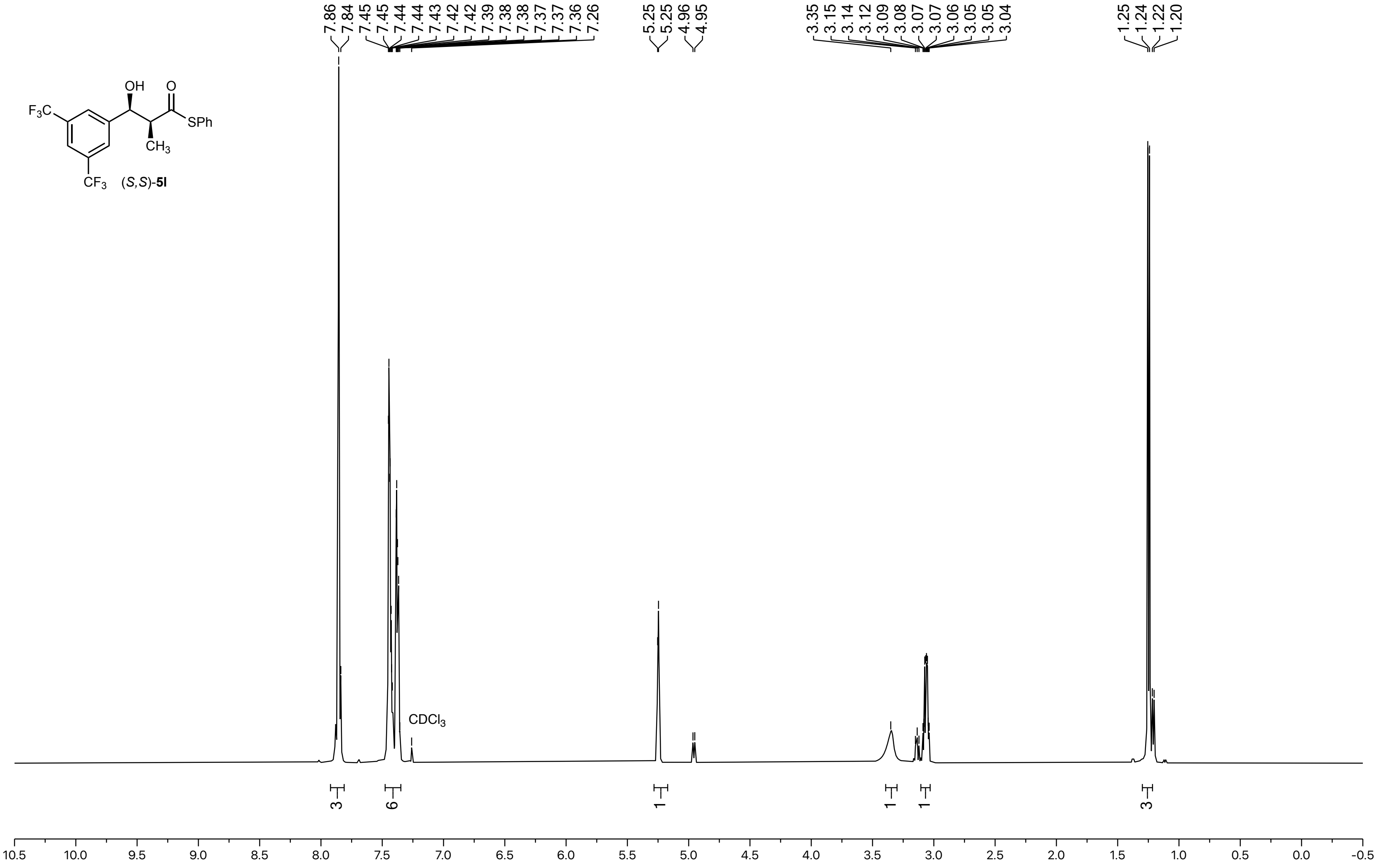

$^{13}C\{^{1}H\}$ NMR, 126 MHz, $CDCl_3$, **5l**

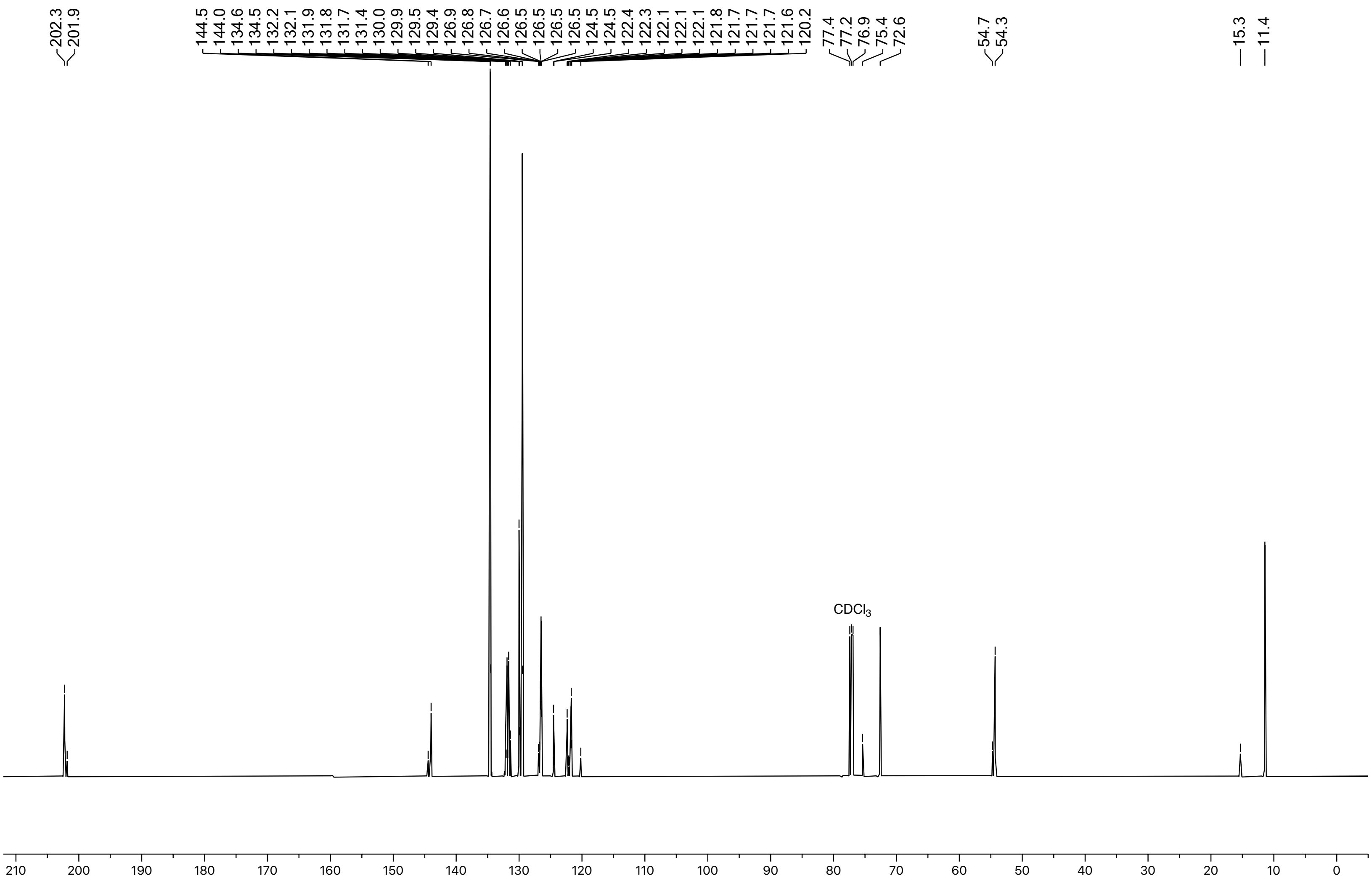

^{19}F NMR, 470 MHz, $CDCl_3$, **5l**

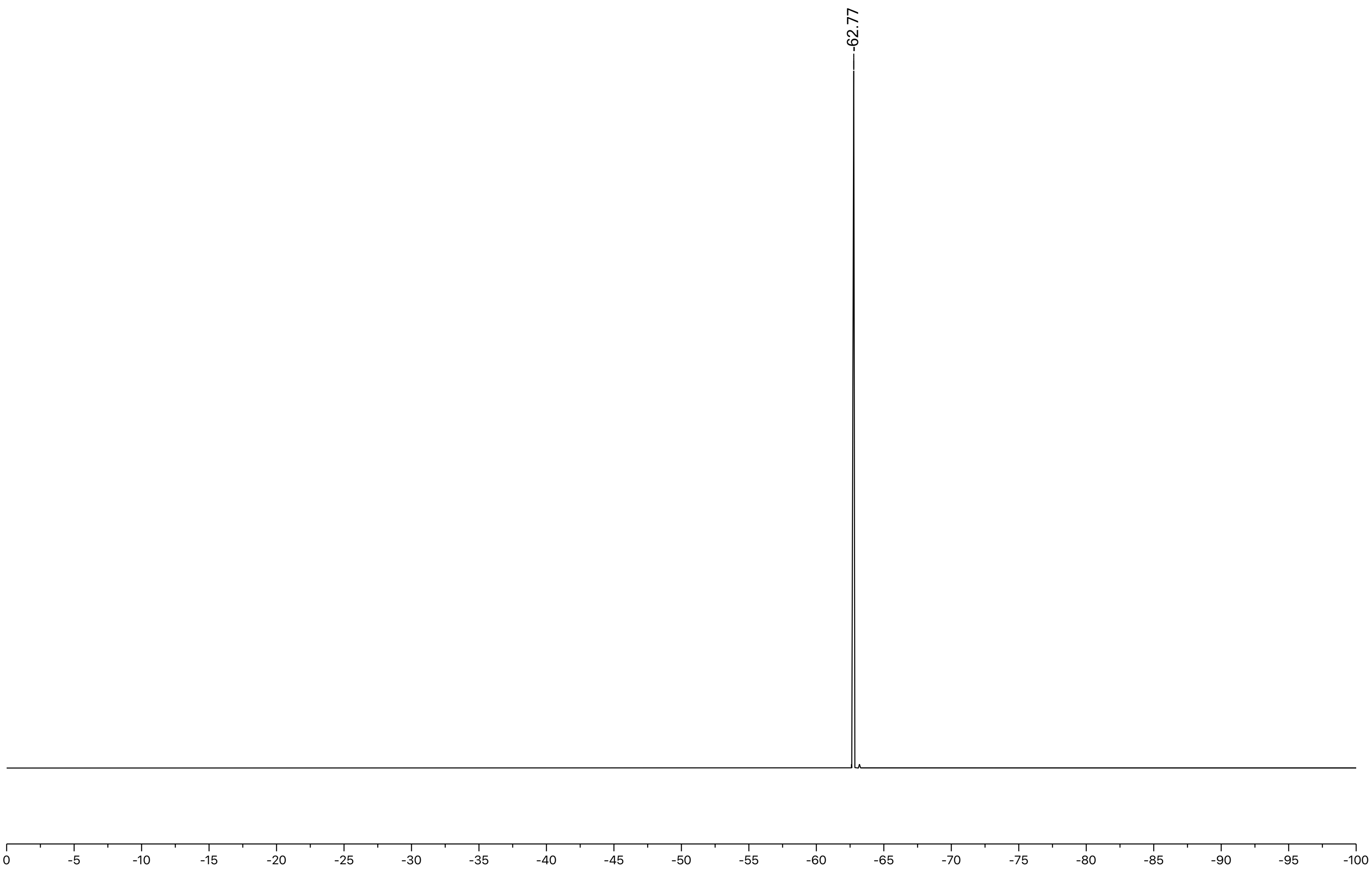

^1H NMR, 500 MHz, $CDCl_3$, **6l**

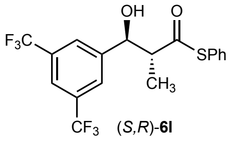

(*S*,*R*)-**6l**

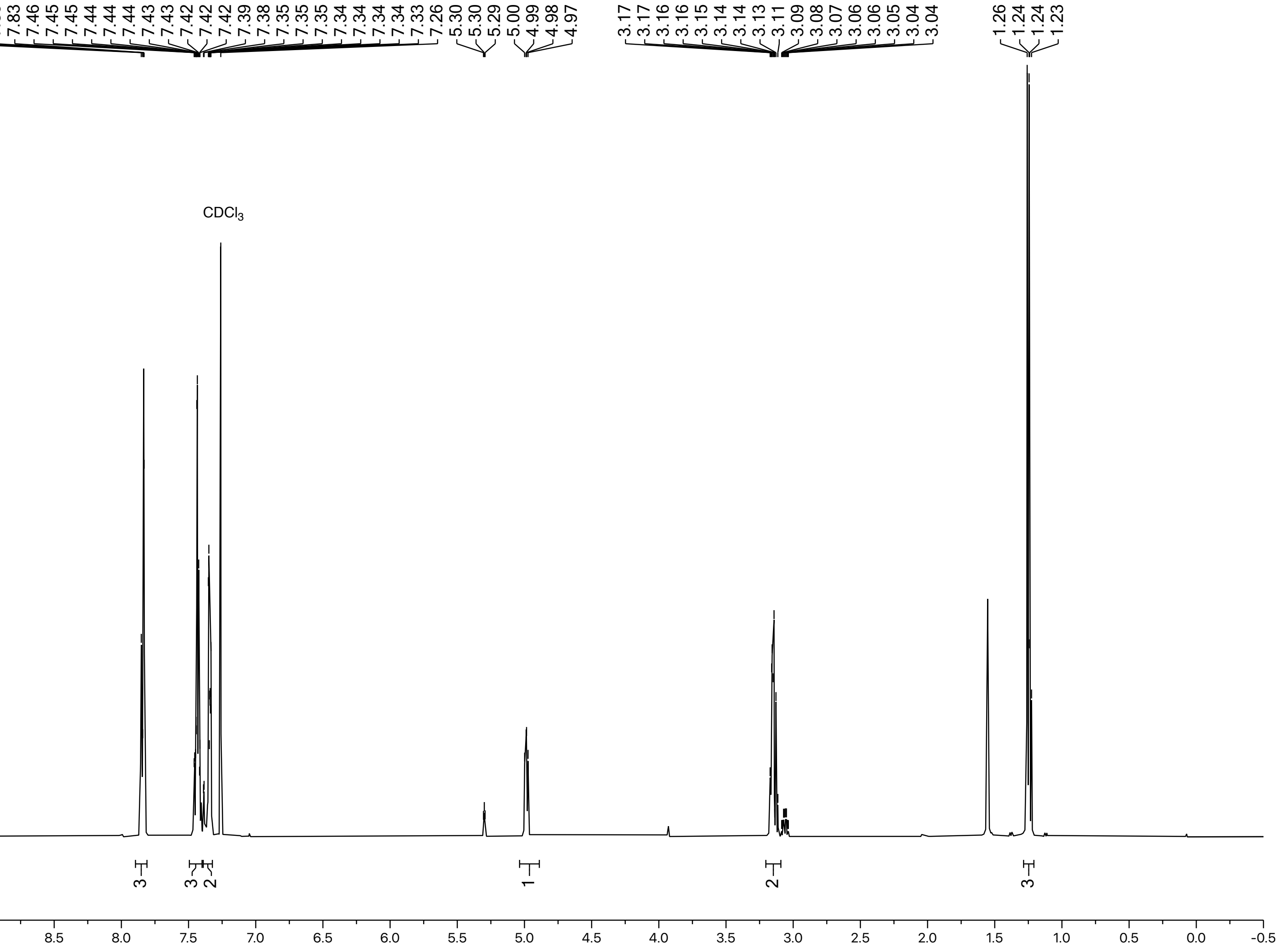

$^{13}C\{^{1}H\}$ NMR, 126 MHz, $CDCl_3$, **6l**

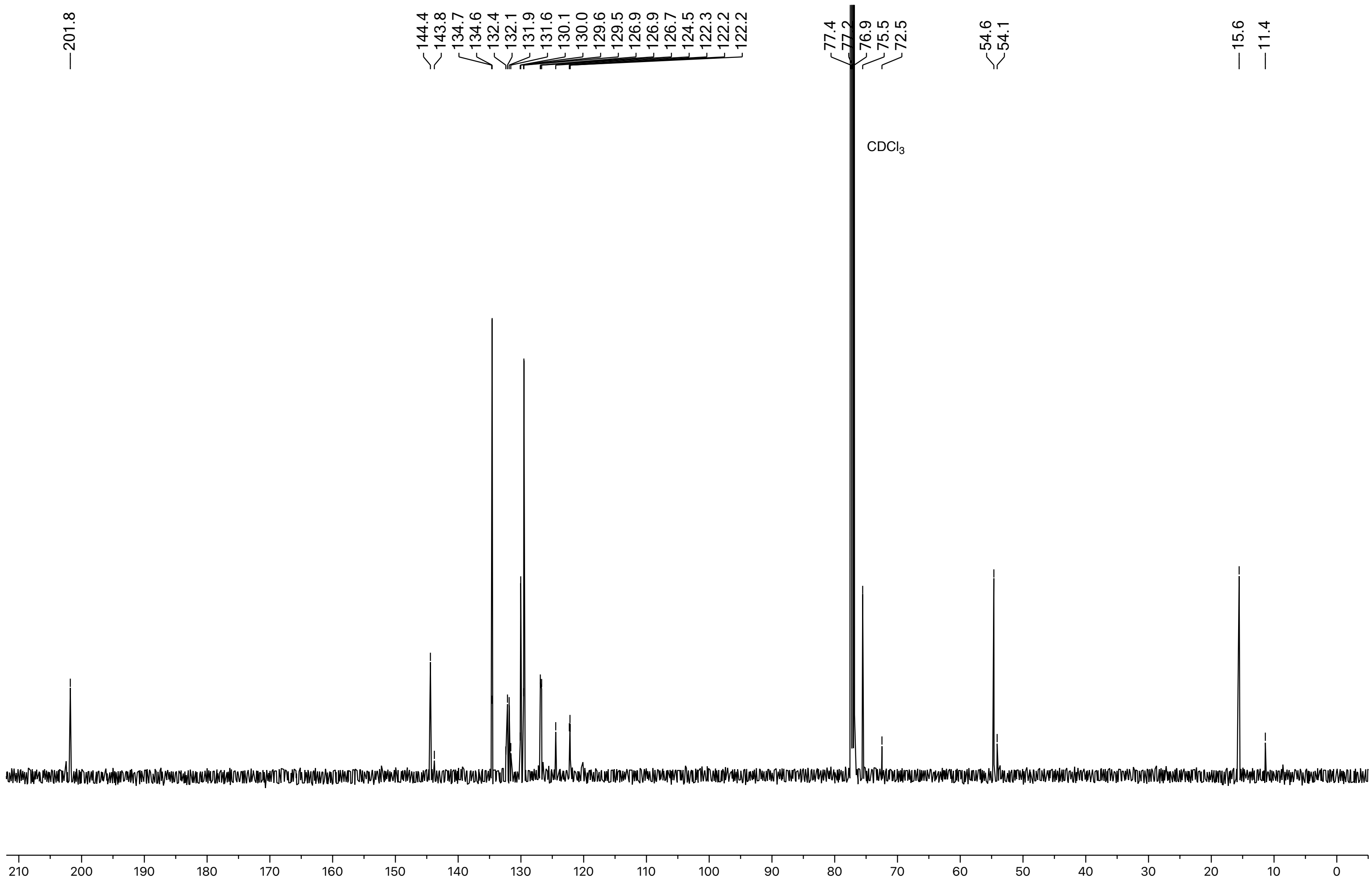

^{19}F NMR, 470 MHz, $CDCl_3$, **6l**

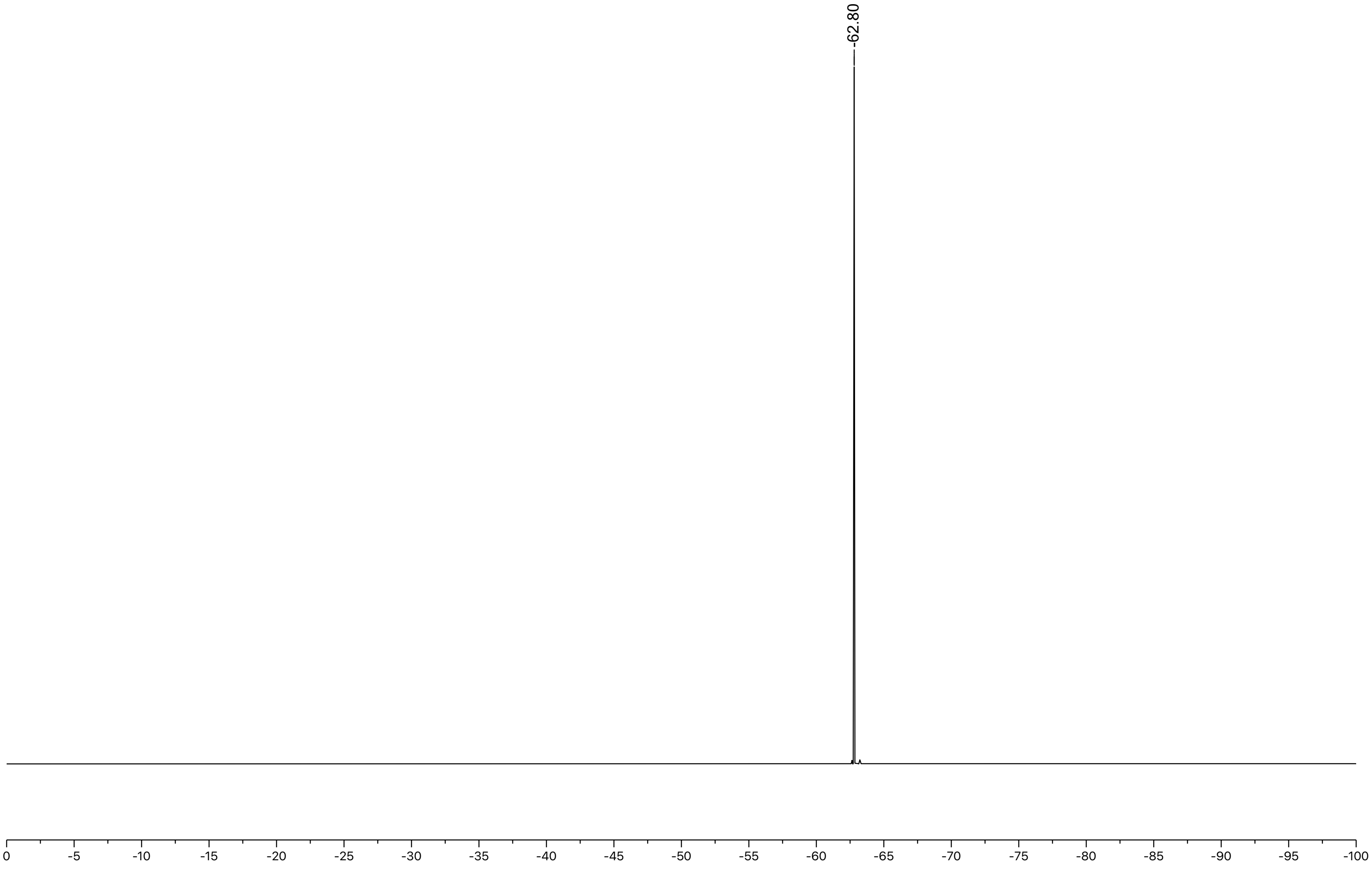

^{1}H NMR, 500 MHz, $CDCl_3$, **5m**

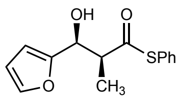

(*S*,*S*)-**5m**

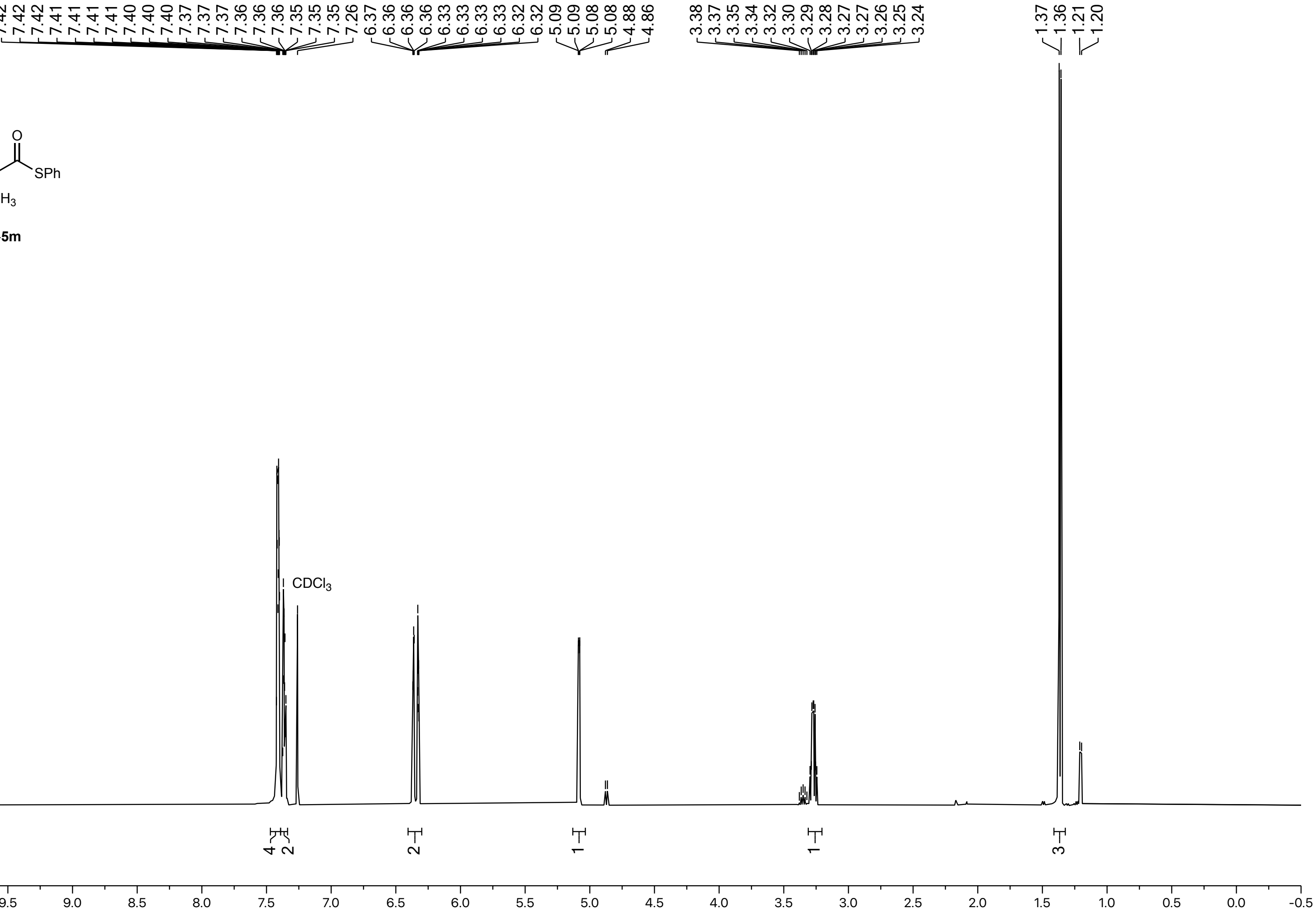

$^{13}C\{^{1}H\}$ NMR, 126 MHz, $CDCl_3$, **5m**

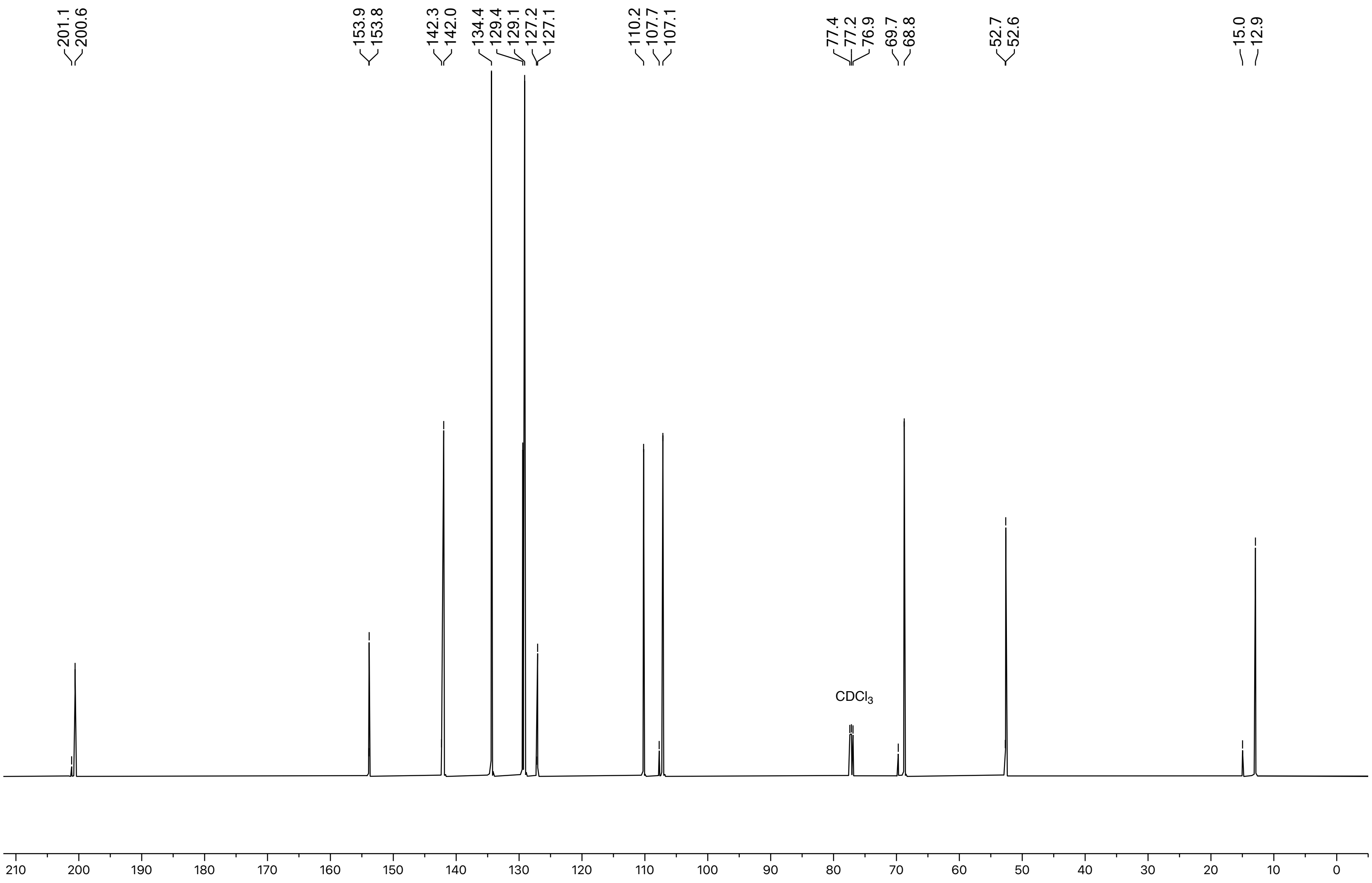

^{1}H NMR, 500 MHz, $CDCl_3$, **6m**

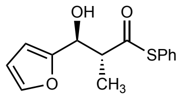

(*S*,*R*)-**6m**

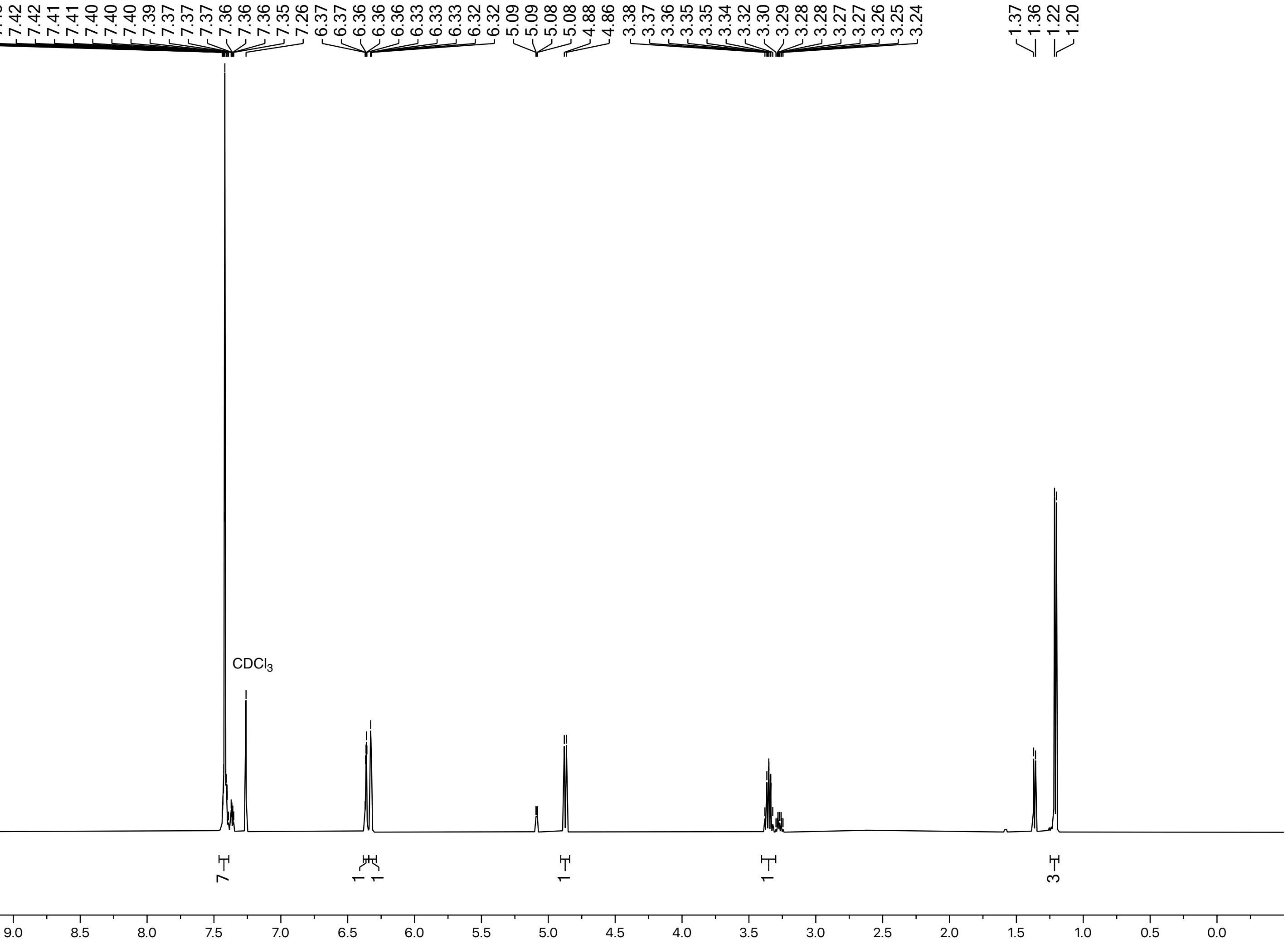

$^{13}C\{^{1}H\}$ NMR, 126 MHz, $CDCl_3$, **6m**

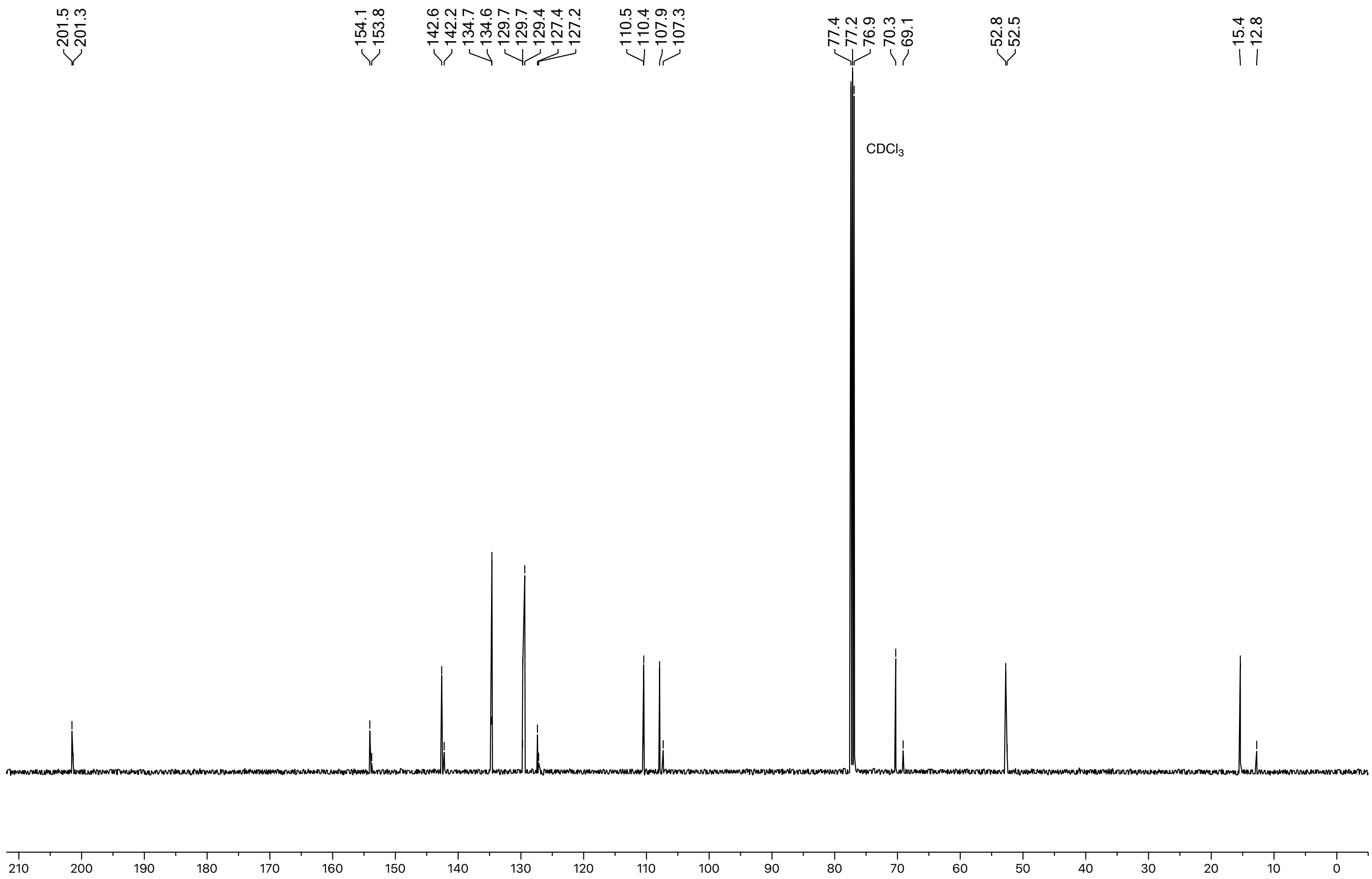

^{1}H NMR, 500 MHz, $CDCl_3$, **5n**

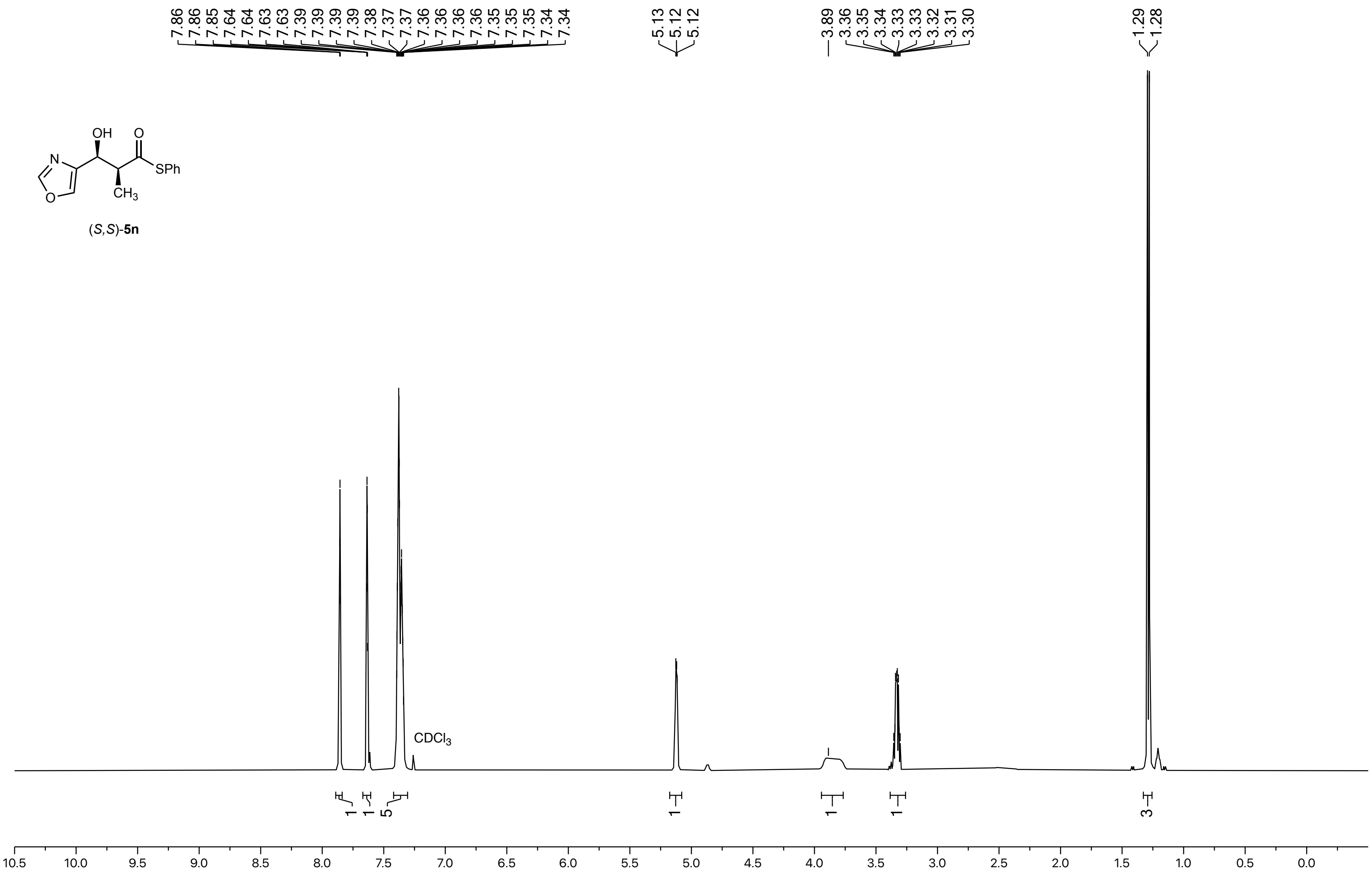

$^{13}C\{^{1}H\}$ NMR, 126 MHz, $CDCl_3$, **5n**

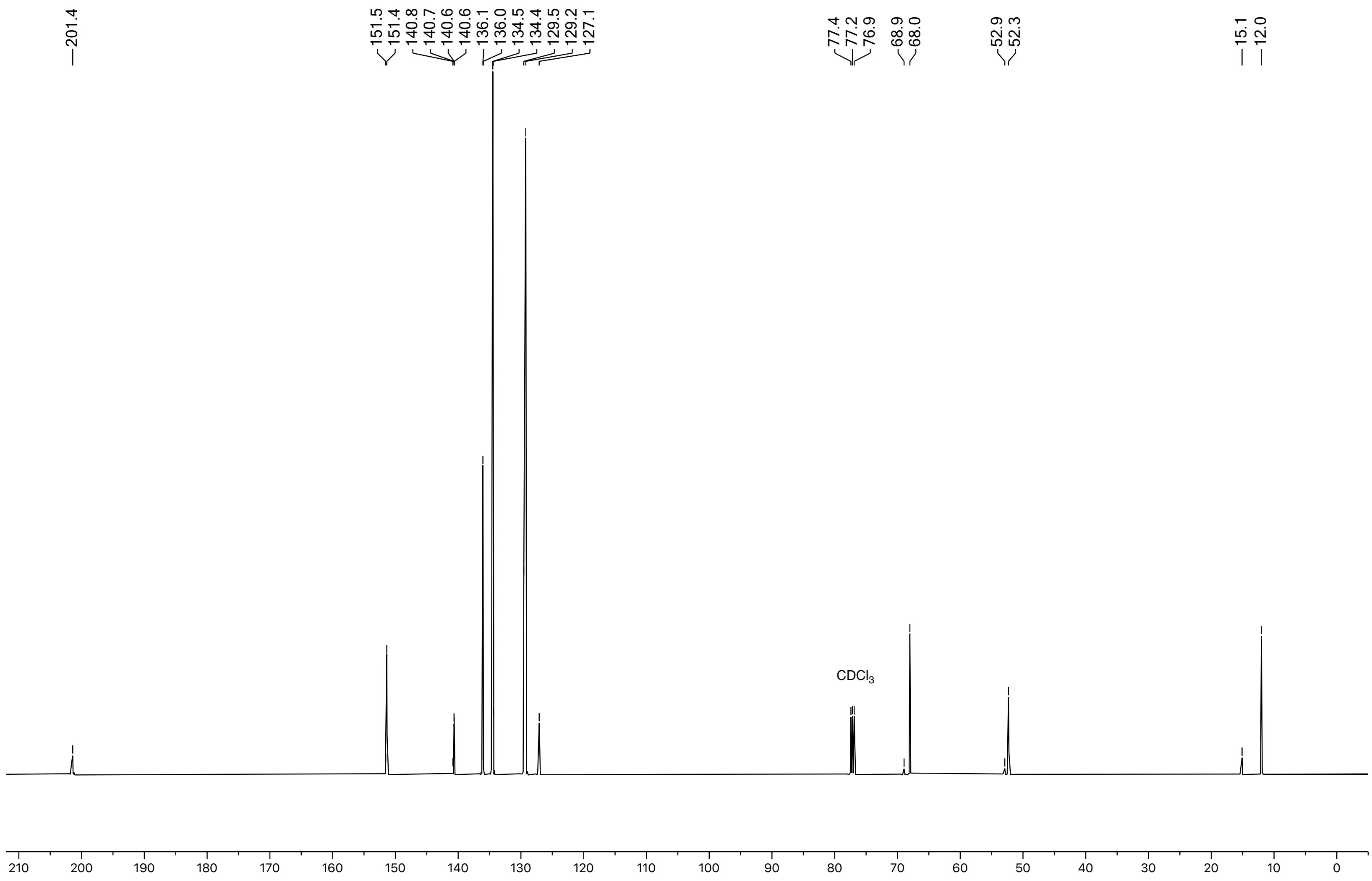

^1H NMR, 500 MHz, $CDCl_3$, **6n**

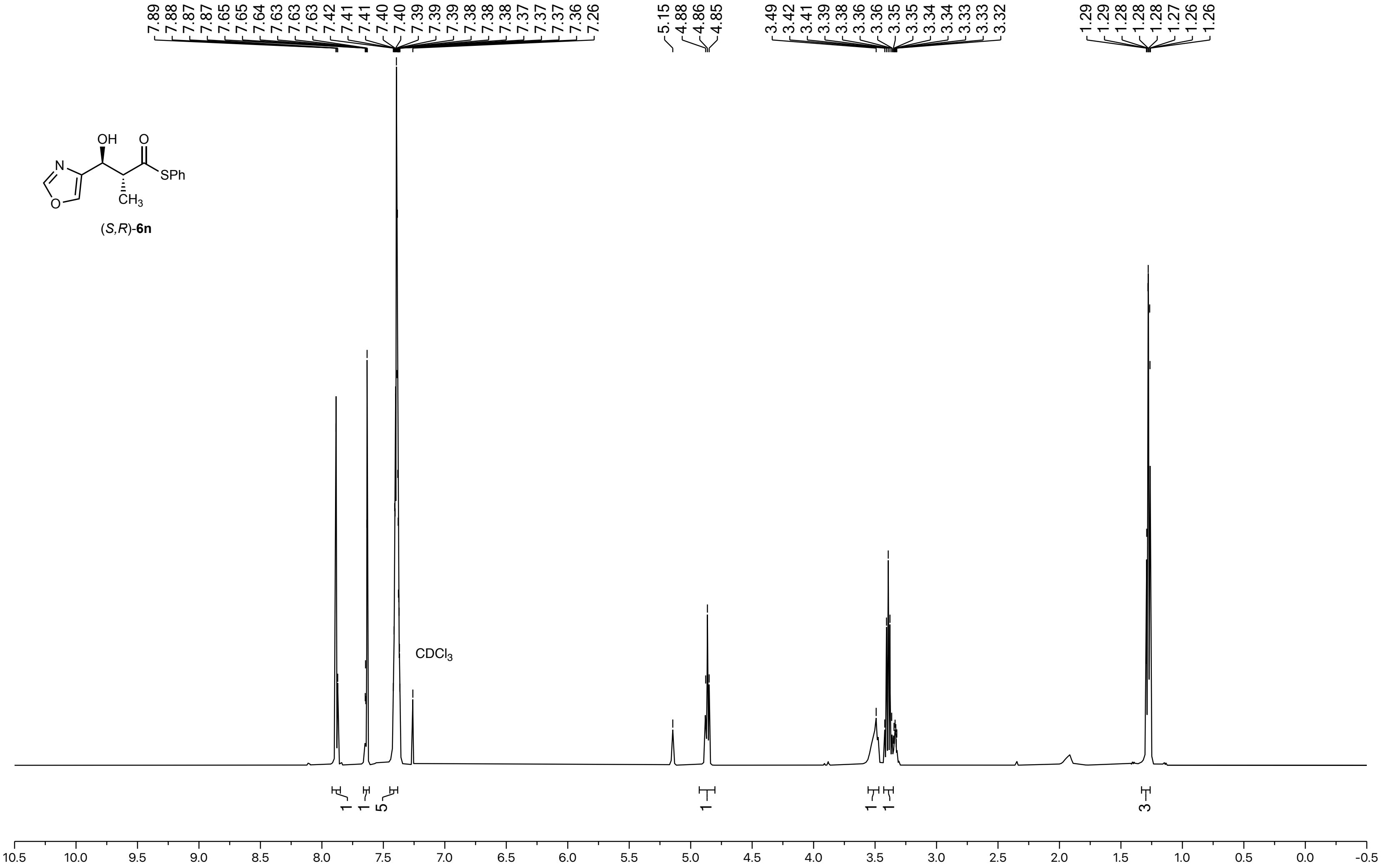

$^{13}C\{^{1}H\}$ NMR, 126 MHz, $CDCl_3$, **6n**

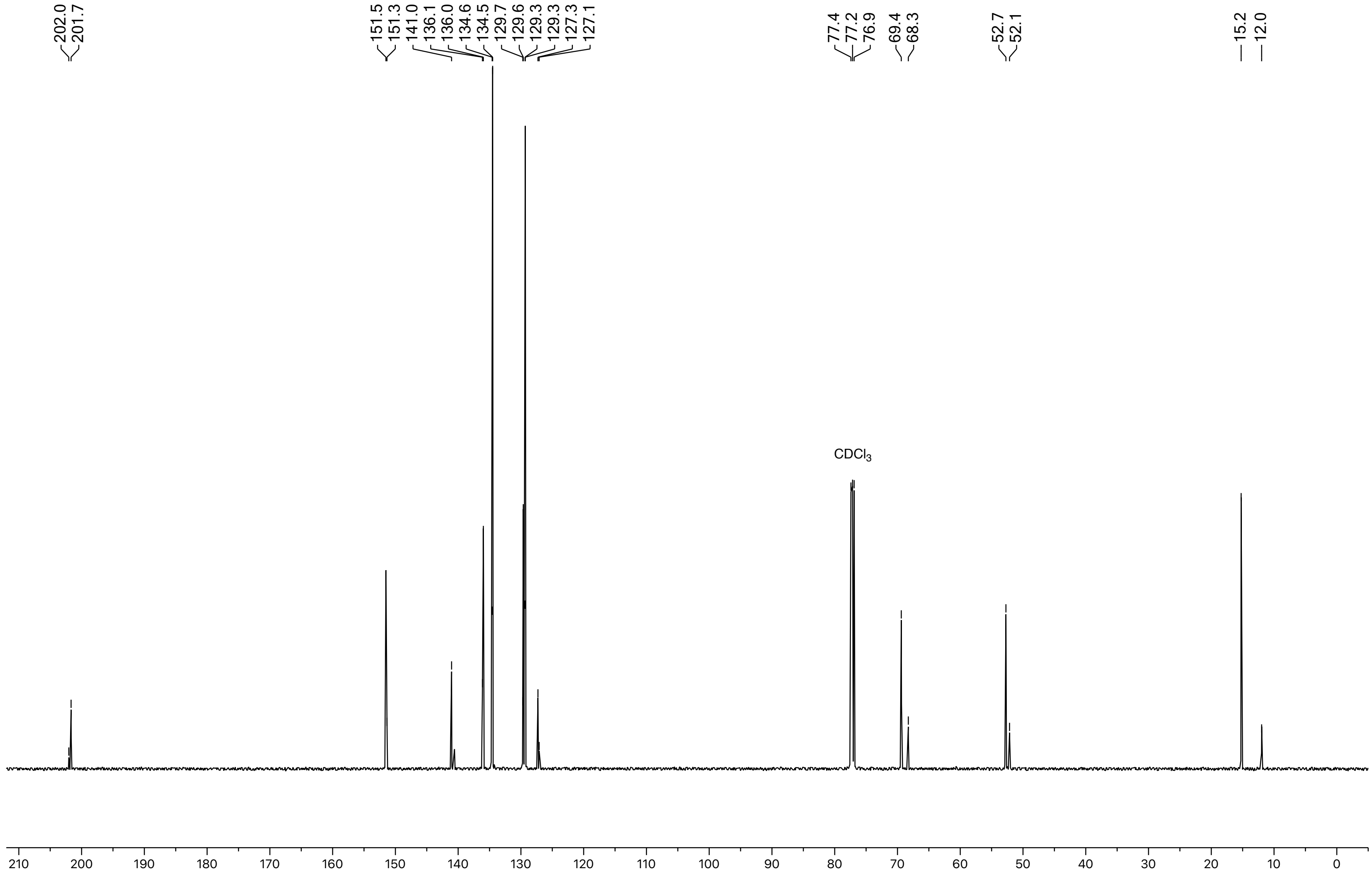

^{1}H NMR, 500 MHz, $CDCl_3$, **5o**

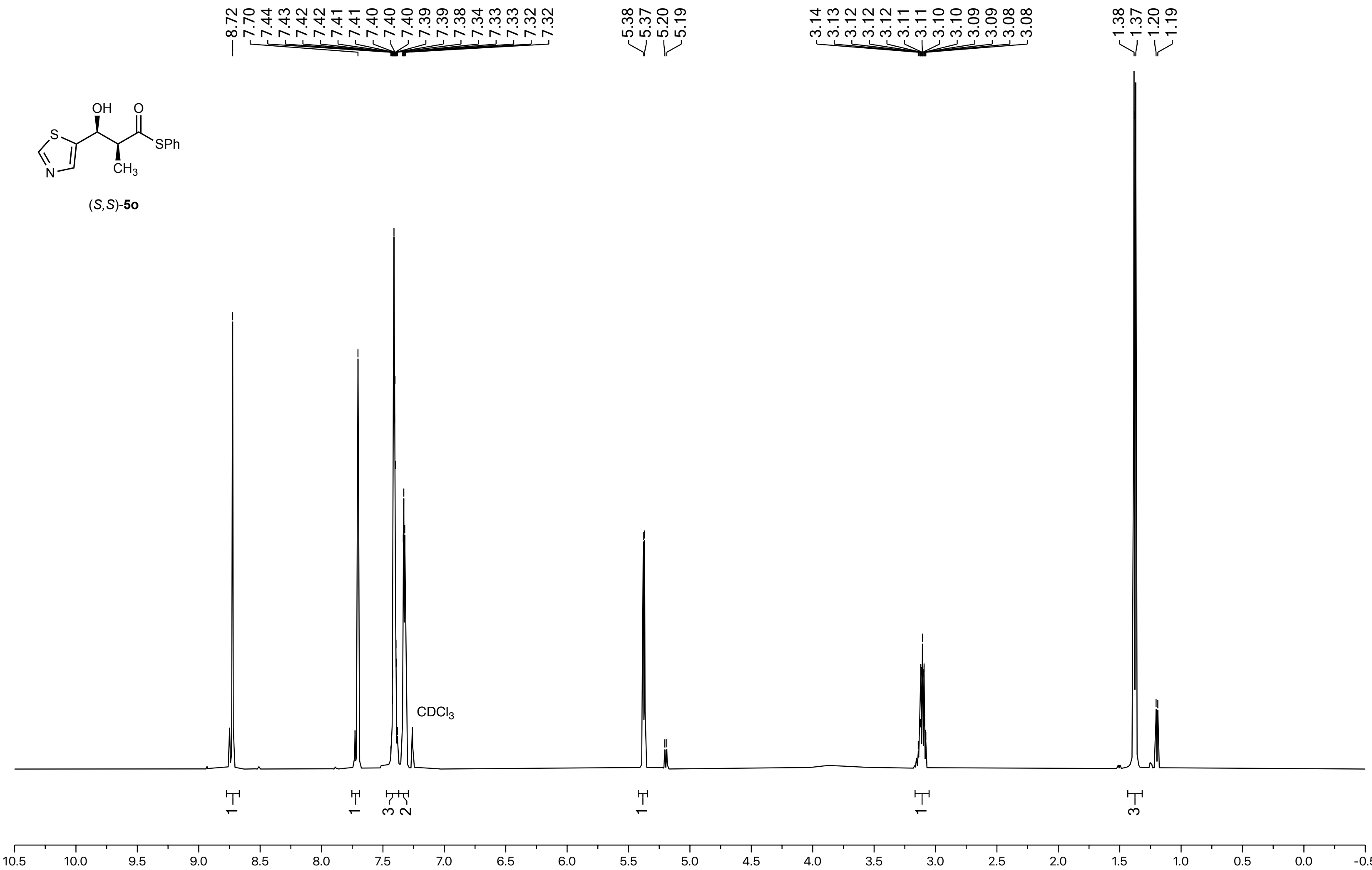

$^{13}C\{^1H\}$ NMR, 126 MHz, $CDCl_3$, **5o**

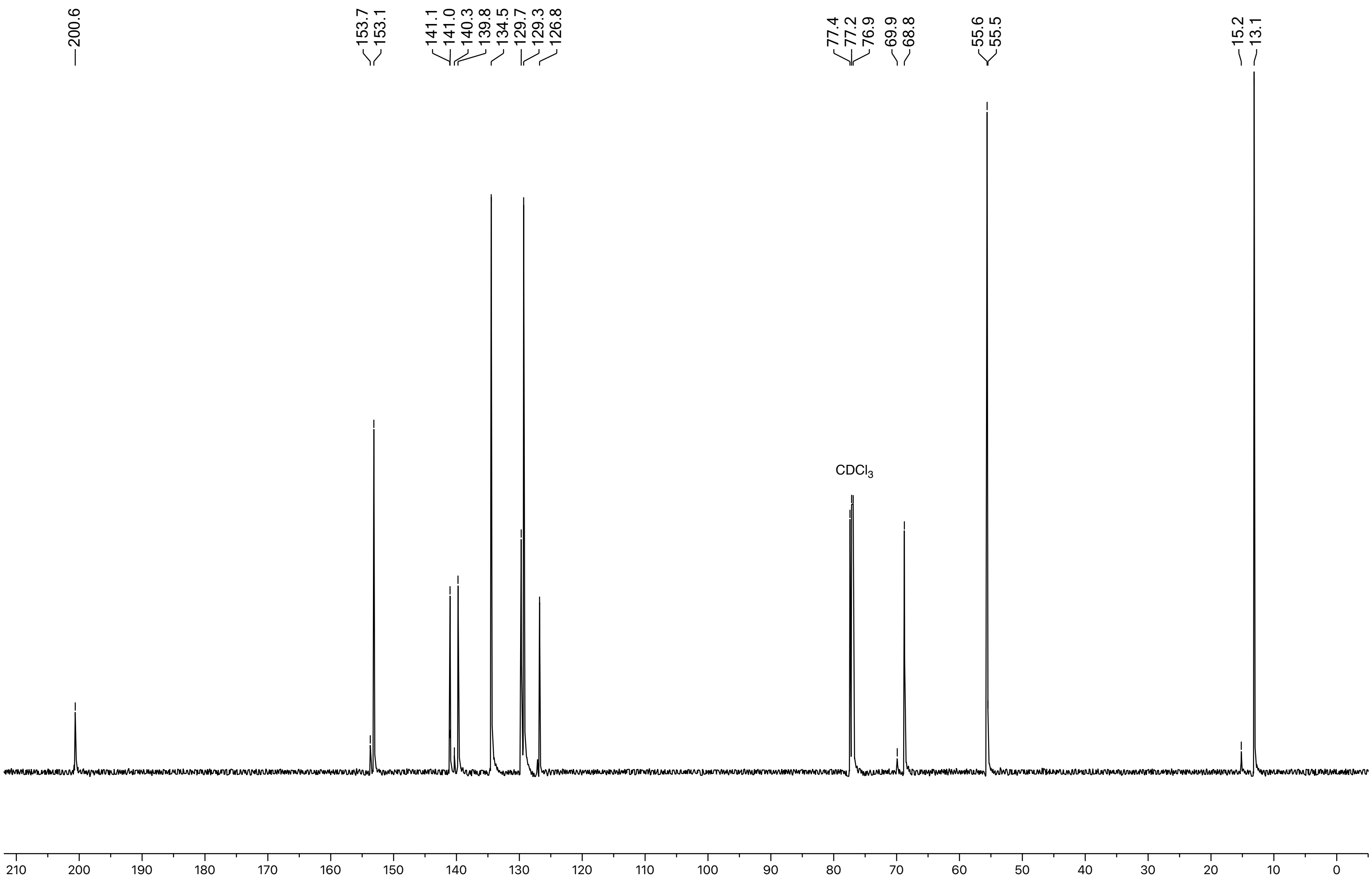

^{1}H NMR, 500 MHz, $CDCl_3$, **6o**

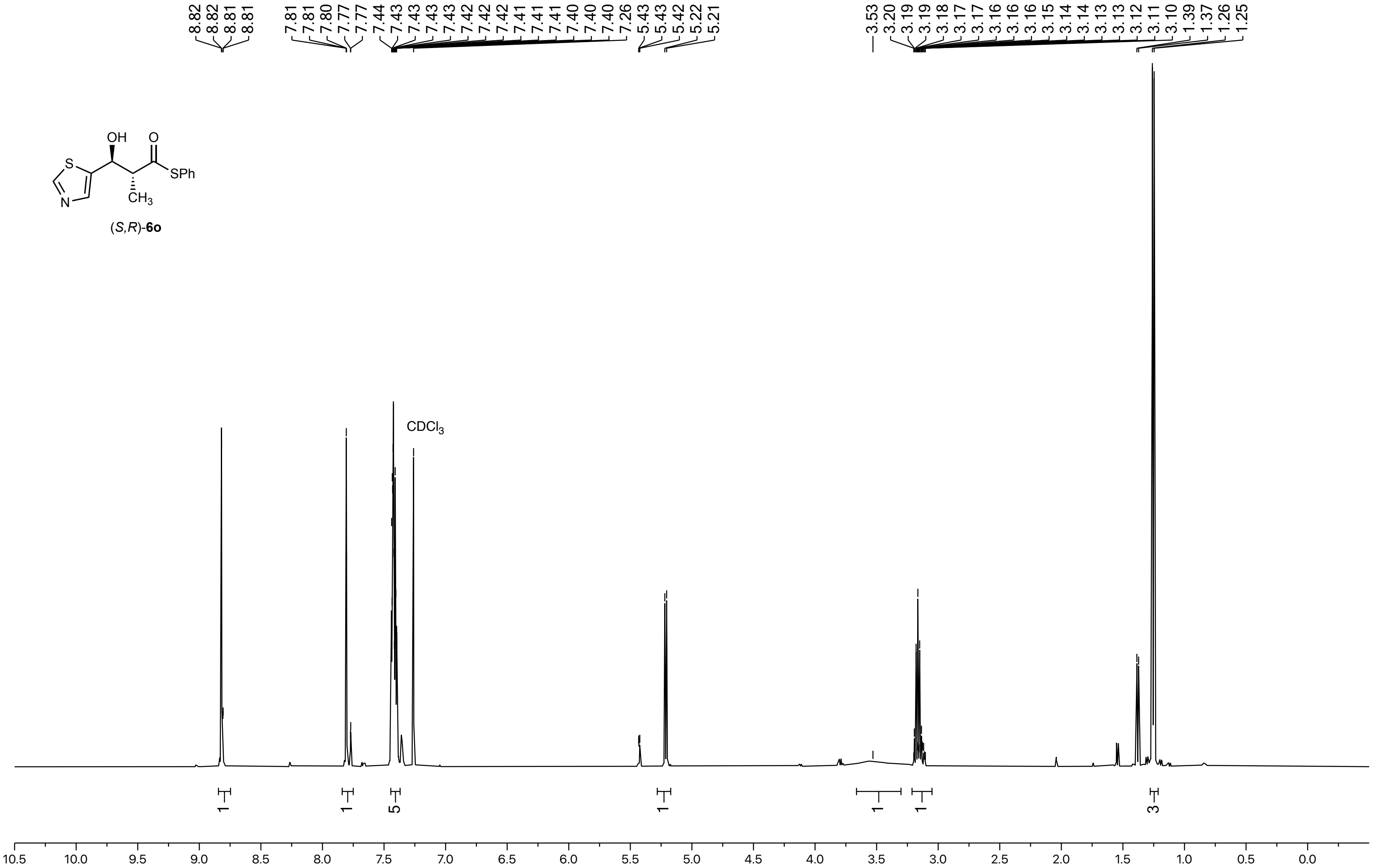

$^{13}C\{^{1}H\}$ NMR, 126 MHz, $CDCl_3$, **6o**

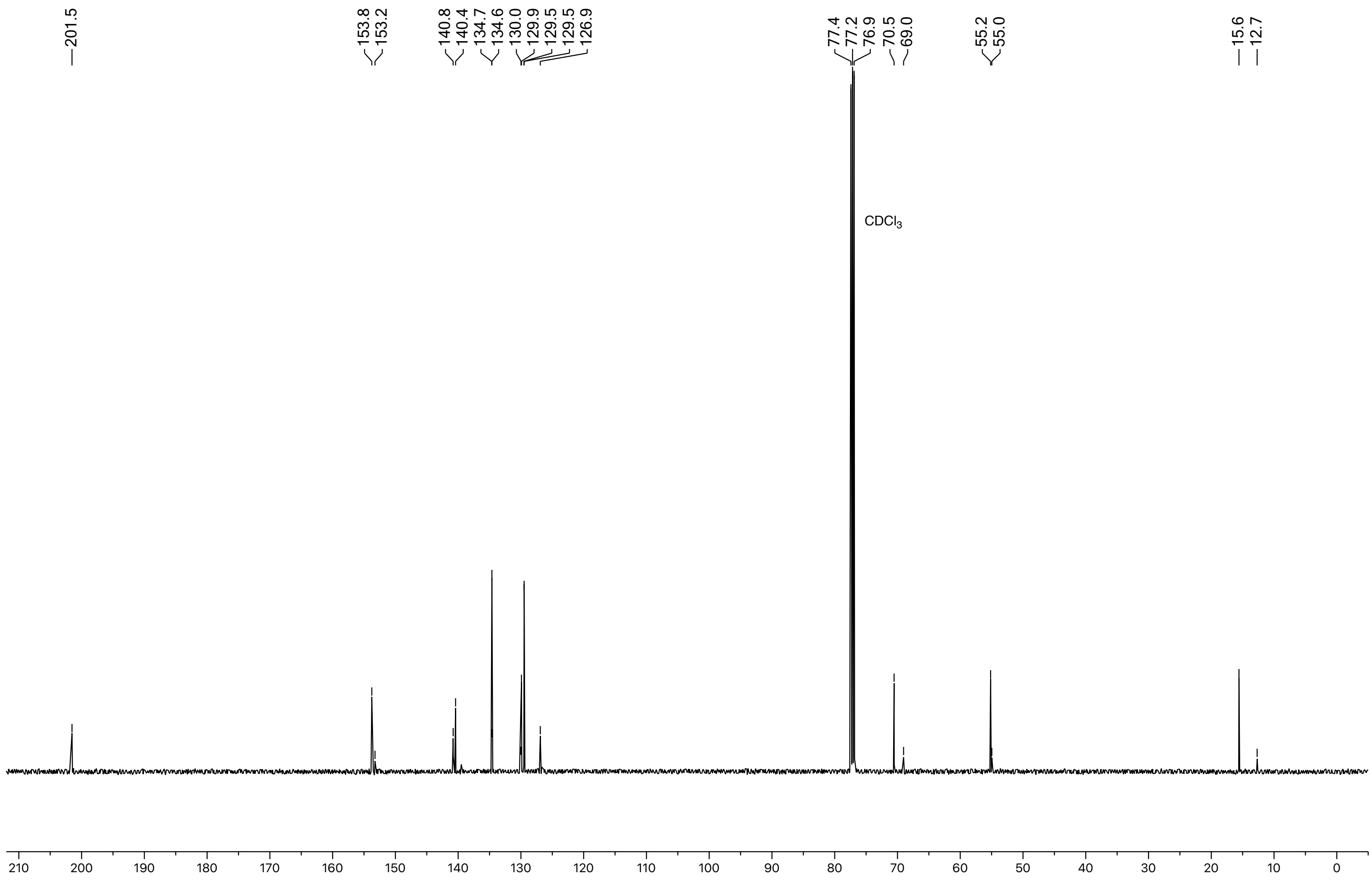

^{1}H NMR, 500 MHz, $CDCl_3$, **5p**

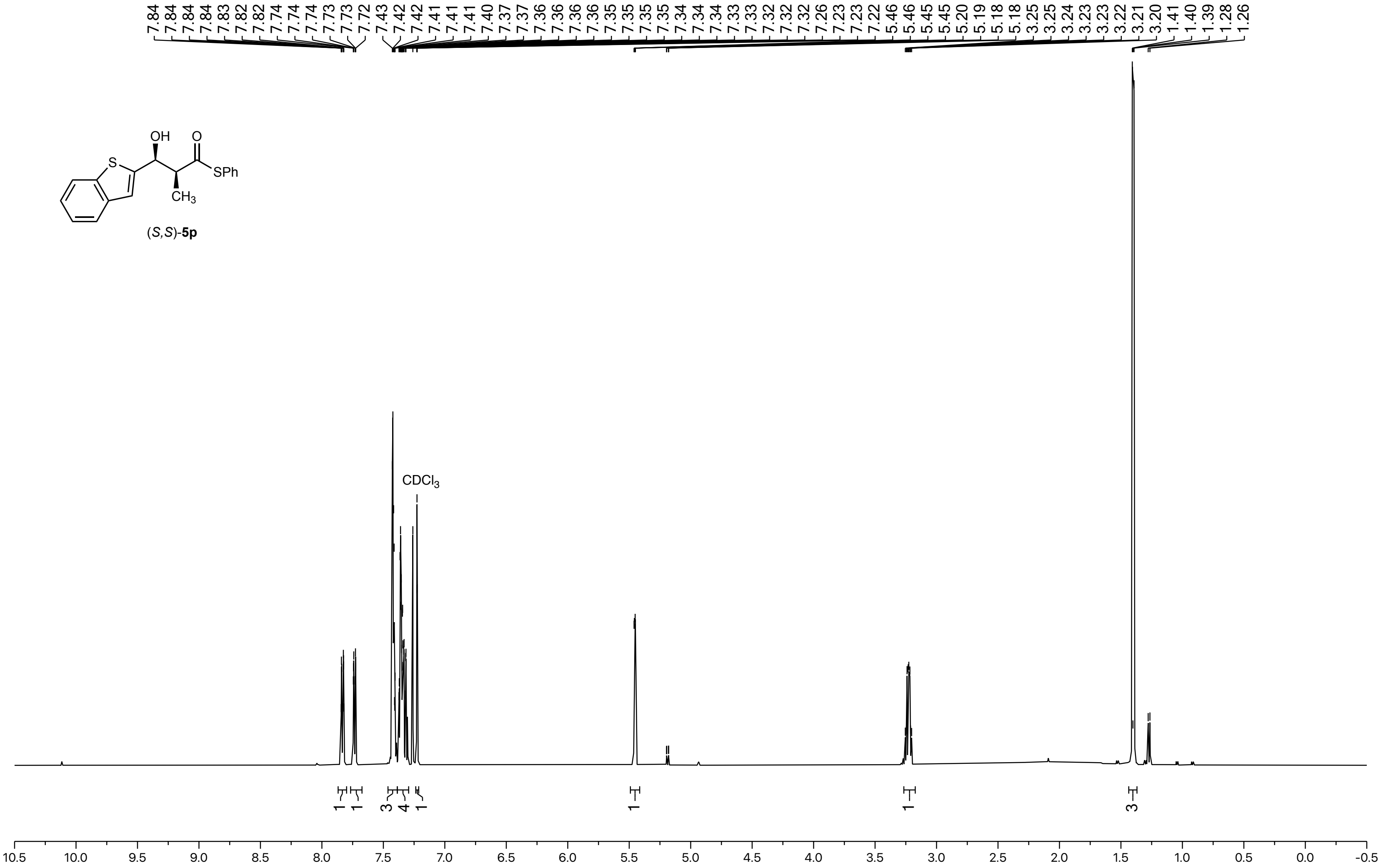

$^{13}C\{^{1}H\}$ NMR, 126 MHz, $CDCl_3$, **5p**

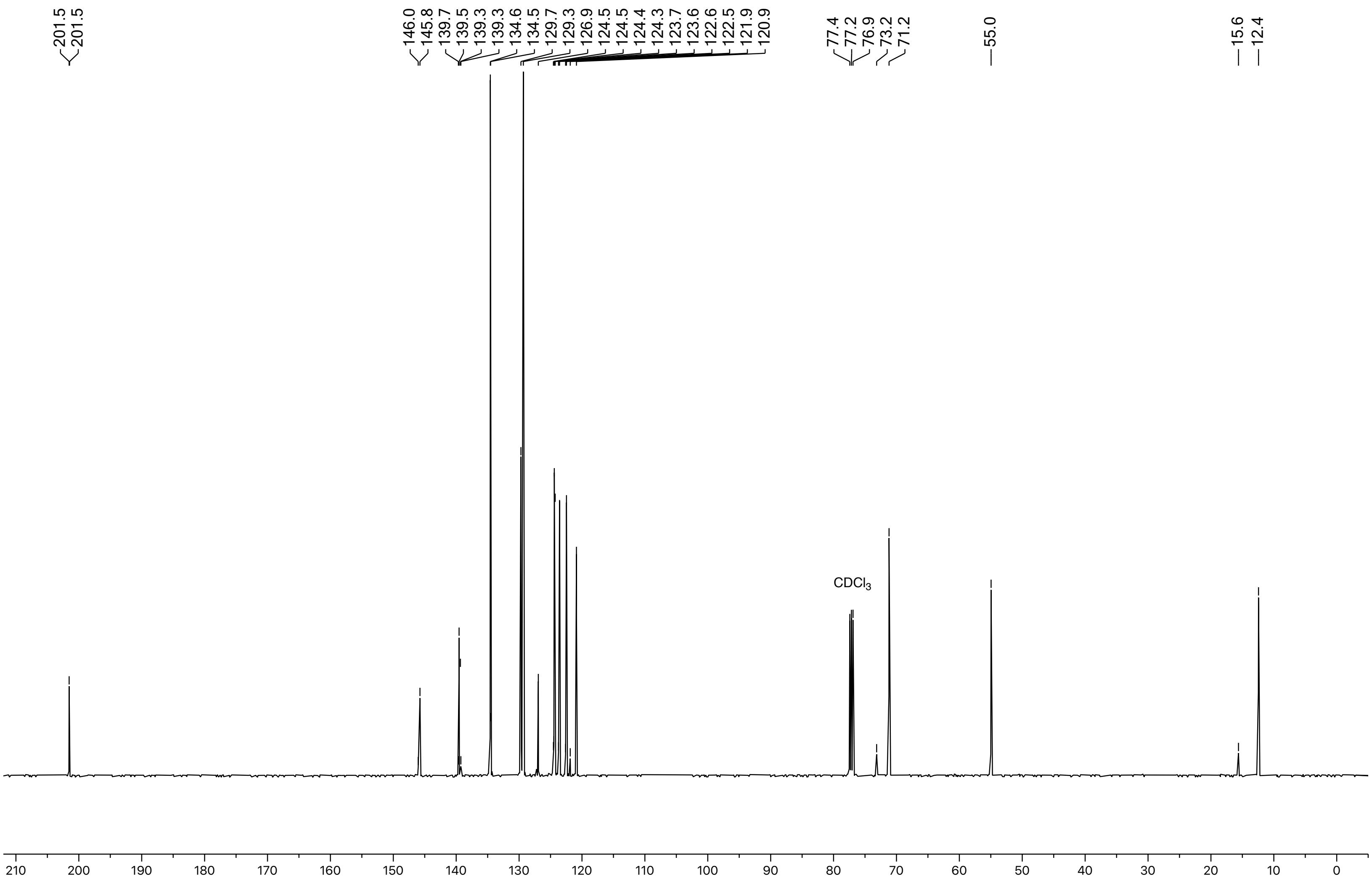

^{1}H NMR, 500 MHz, $CDCl_3$, **6p**

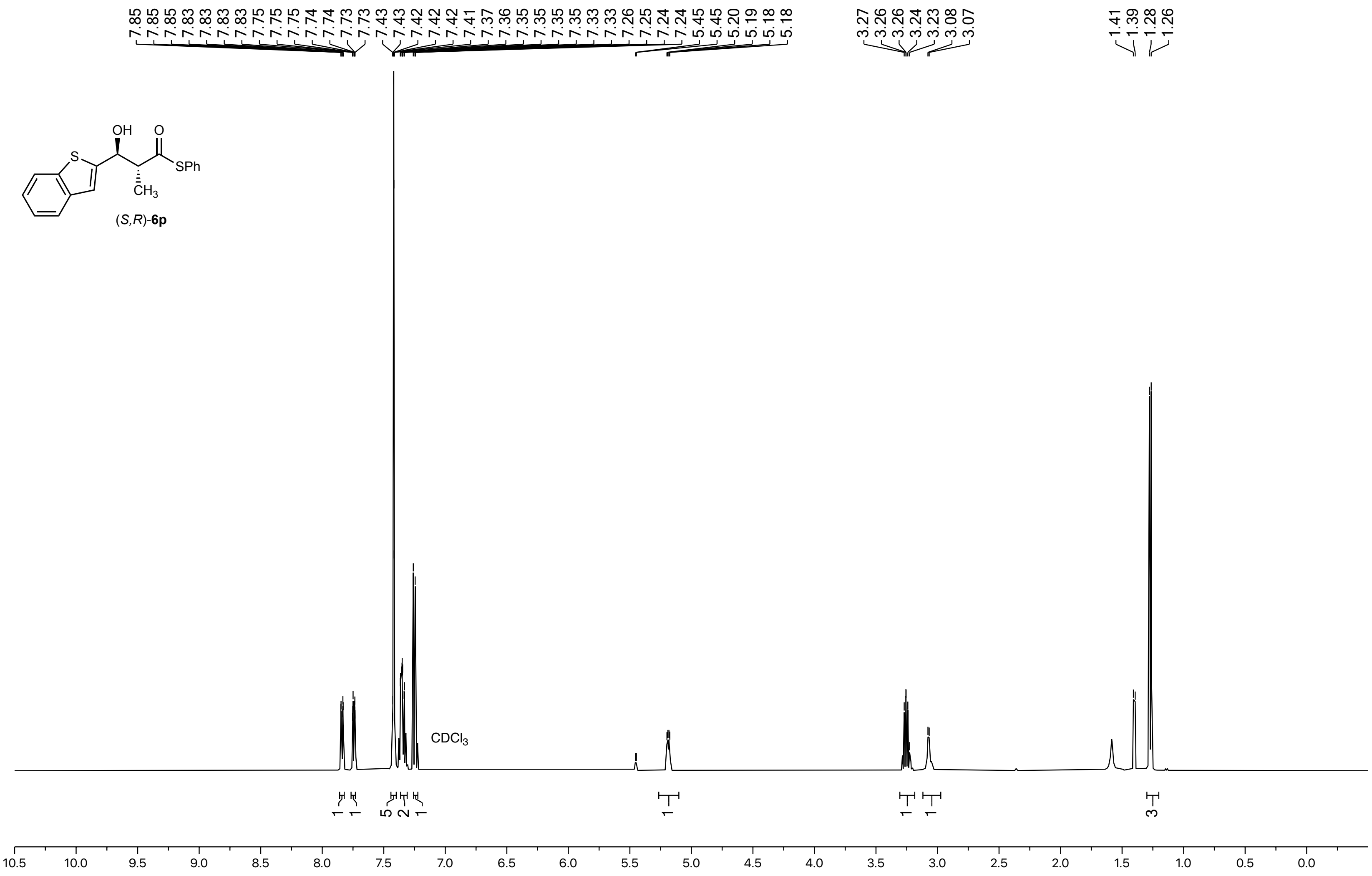

$^{13}C\{^1H\}$ NMR, 126 MHz, $CDCl_3$, **6p**

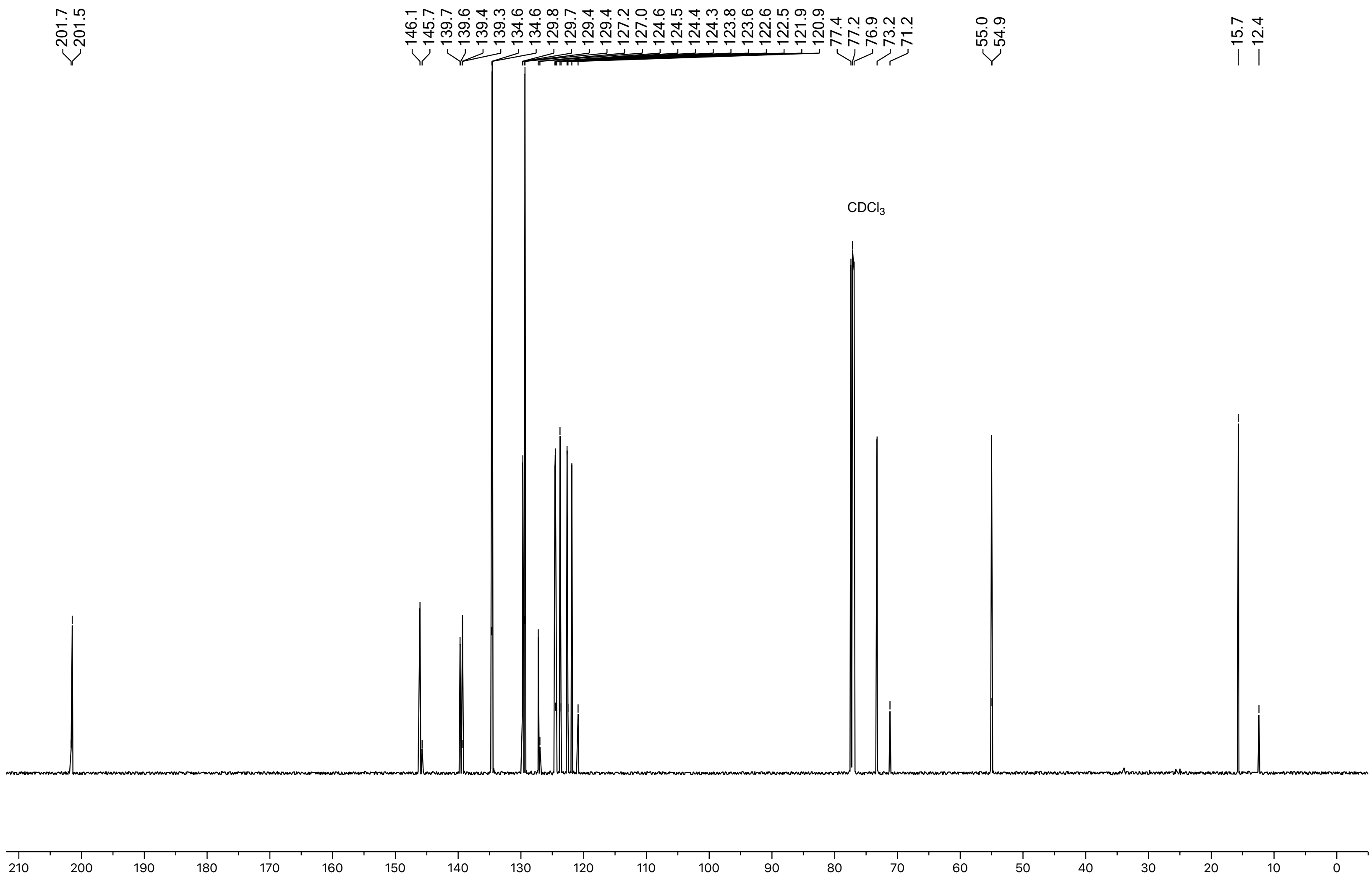

^{1}H NMR, 500 MHz, $CDCl_3$, **5q**

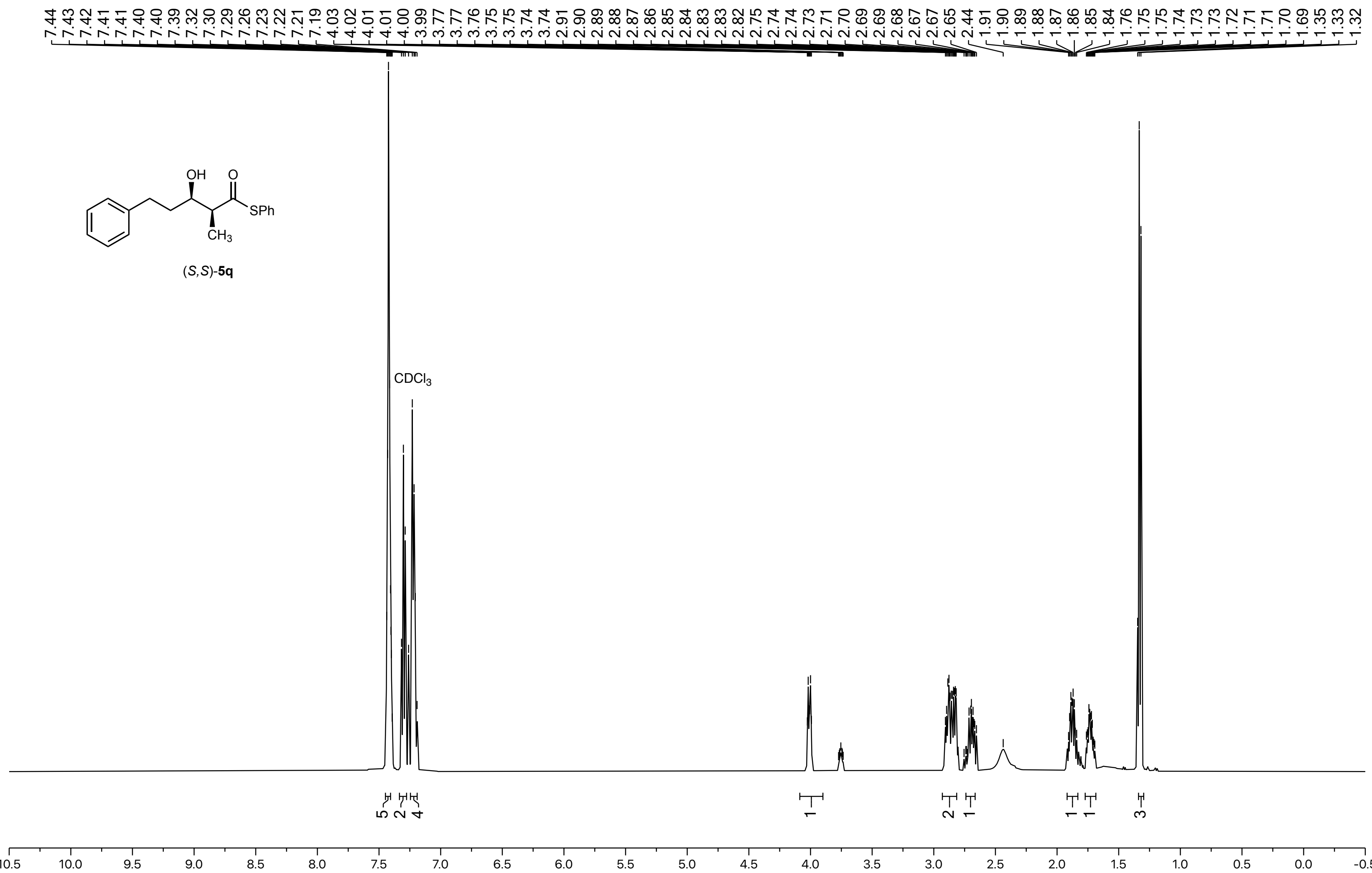

$^{13}C\{^{1}H\}$ NMR, 126 MHz, $CDCl_3$, **5q**

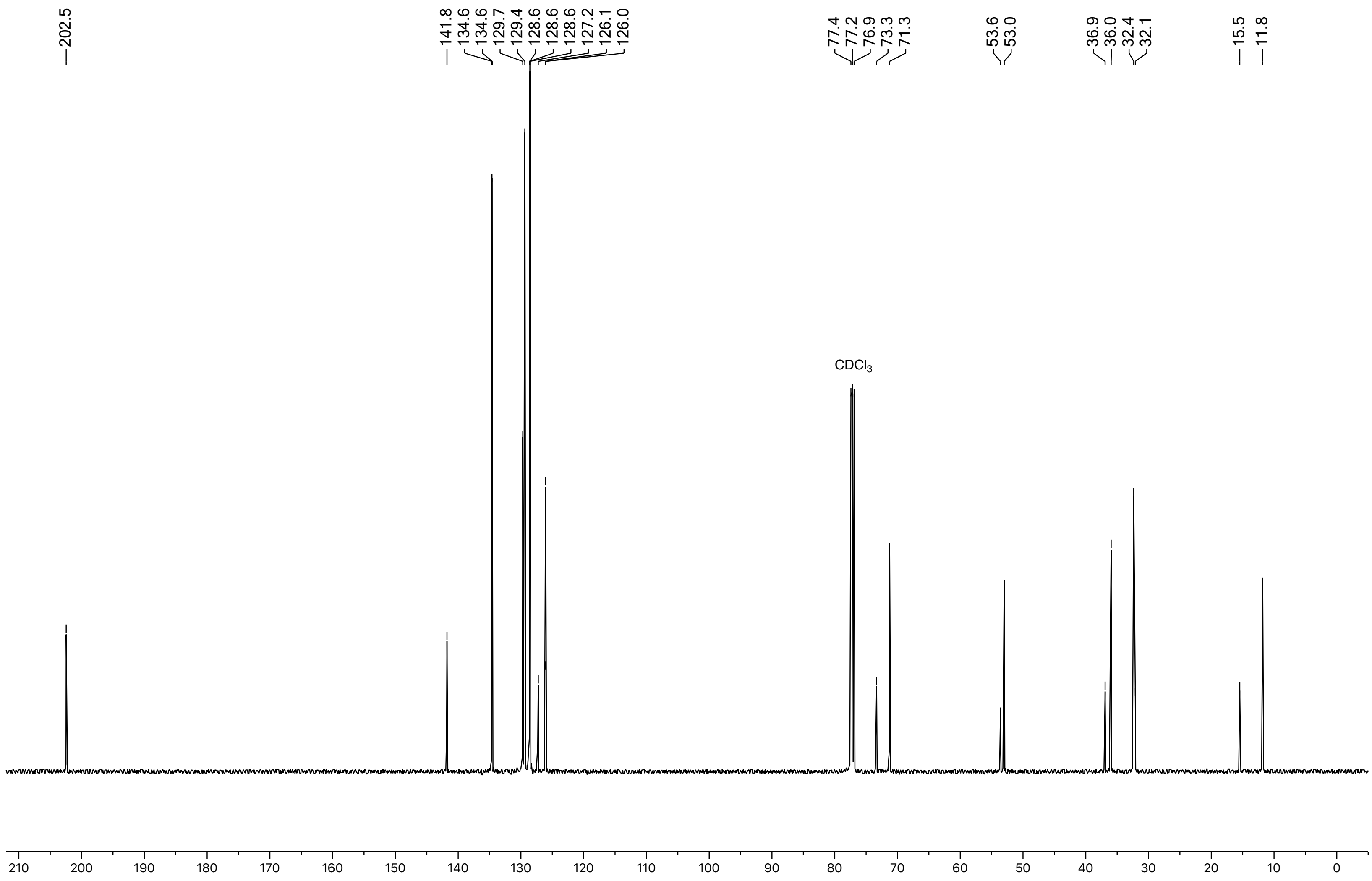

^1H NMR, 500 MHz, $CDCl_3$, **6q**

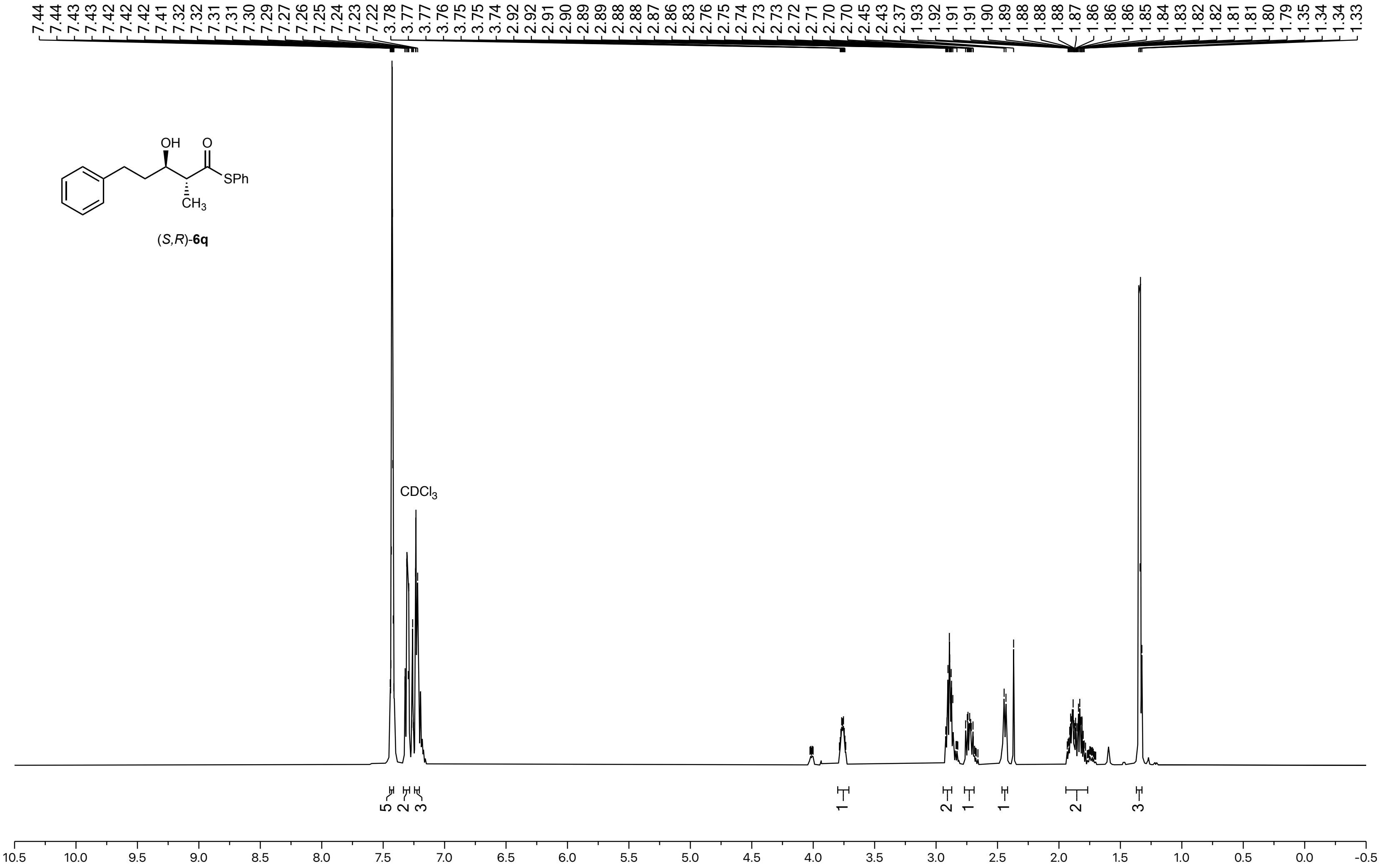

$^{13}C\{^{1}H\}$ NMR, 126 MHz, $CDCl_3$, **6q**

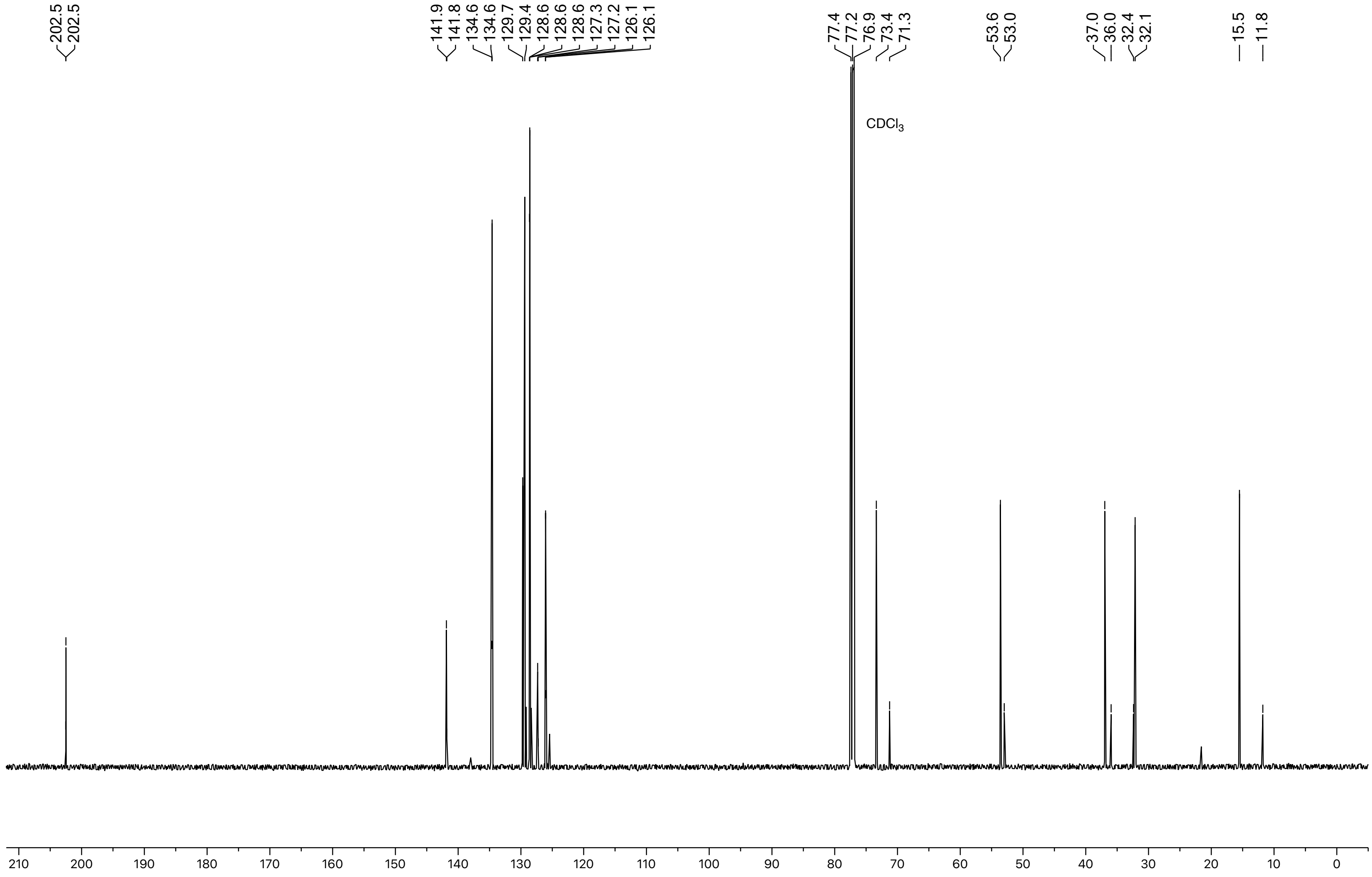

^{1}H NMR, 500 MHz, $CDCl_3$, **5r**

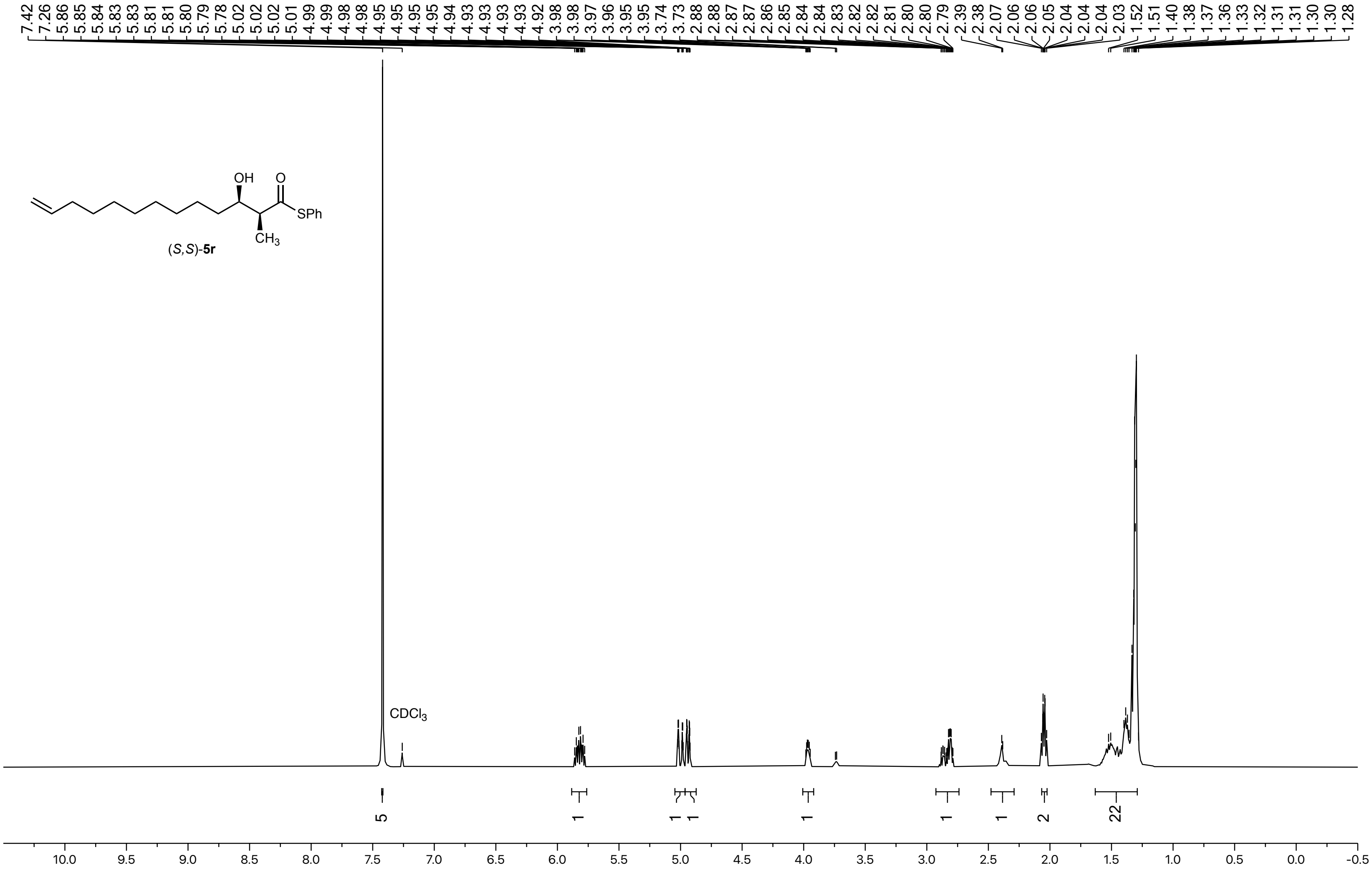

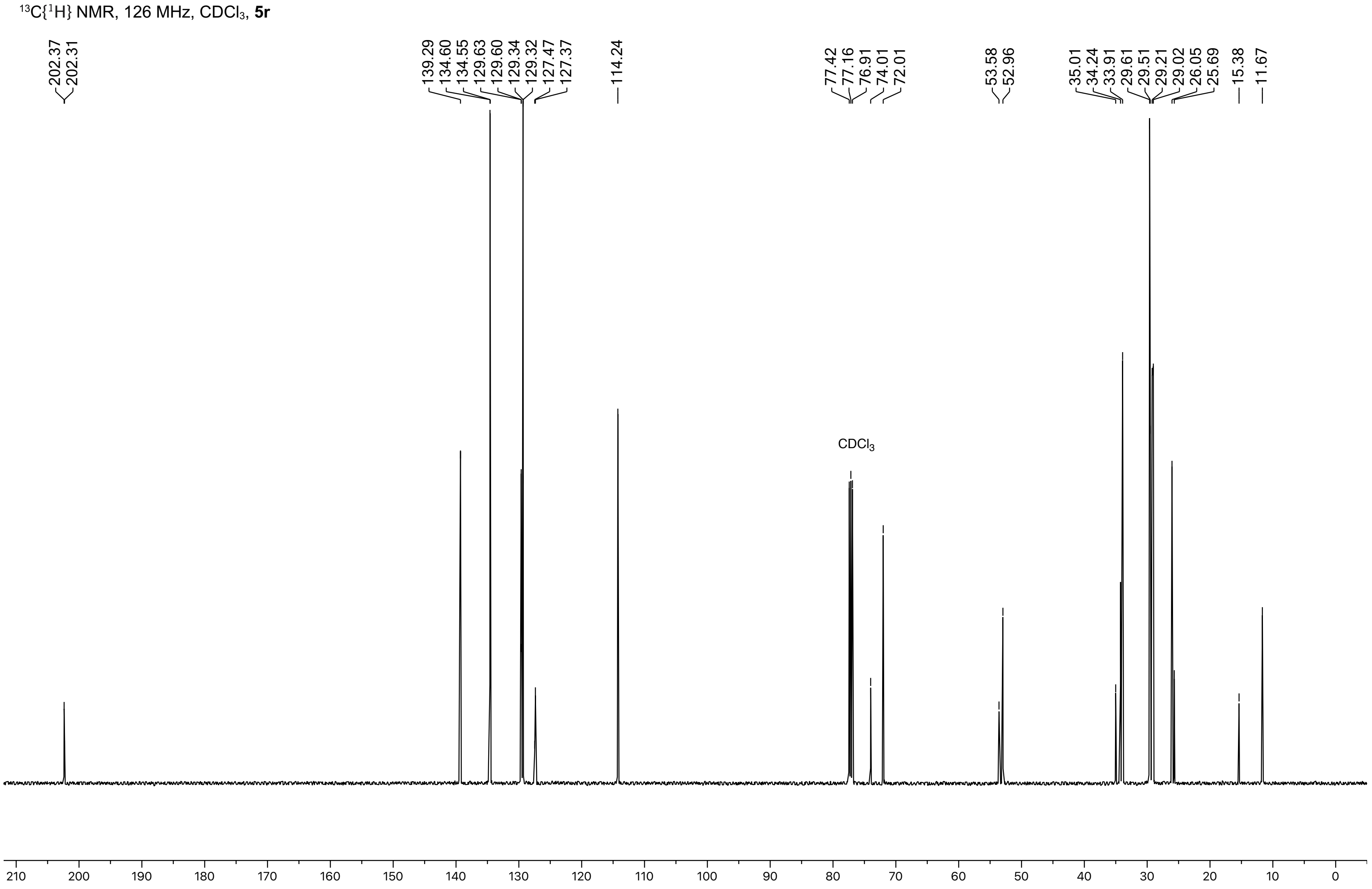

$^{13}C\{^1H\}$ NMR, 126 MHz, $CDCl_3$, **5r**
202.37
202.31
139.29
134.60
134.55
129.63
129.60
129.34
129.32
127.47
127.37
114.24
77.42
77.16
76.91
74.01
72.01
53.58
52.96
35.01
34.24
33.91
29.61
29.51
29.21
29.02
26.05
25.69
15.38
11.67
$CDCl_3$
210 200 190 180 170 160 150 140 130 120 110 100 90 80 70 60 50 40 30 20 10 0

^{1}H NMR, 500 MHz, $CDCl_3$, **6r**

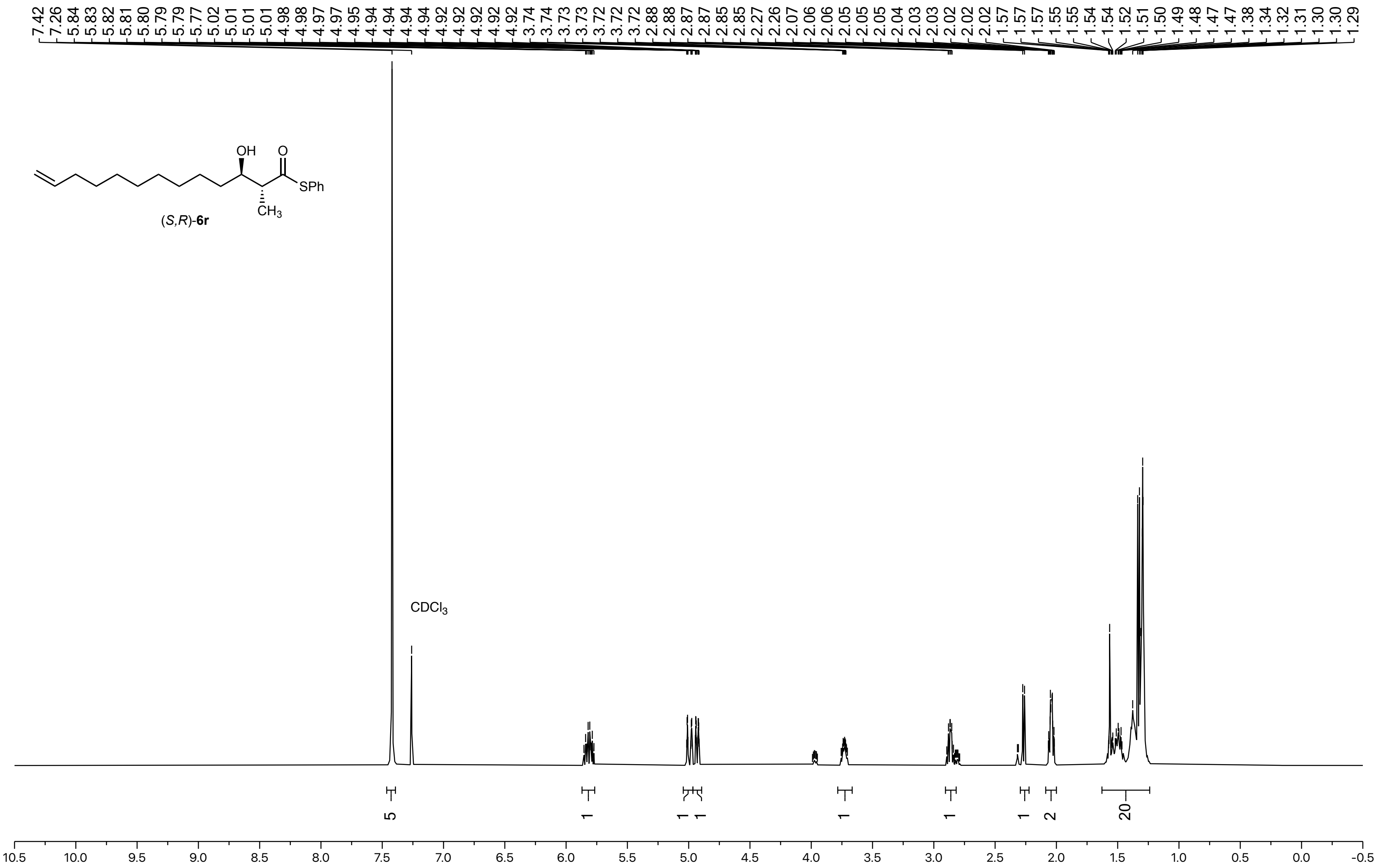

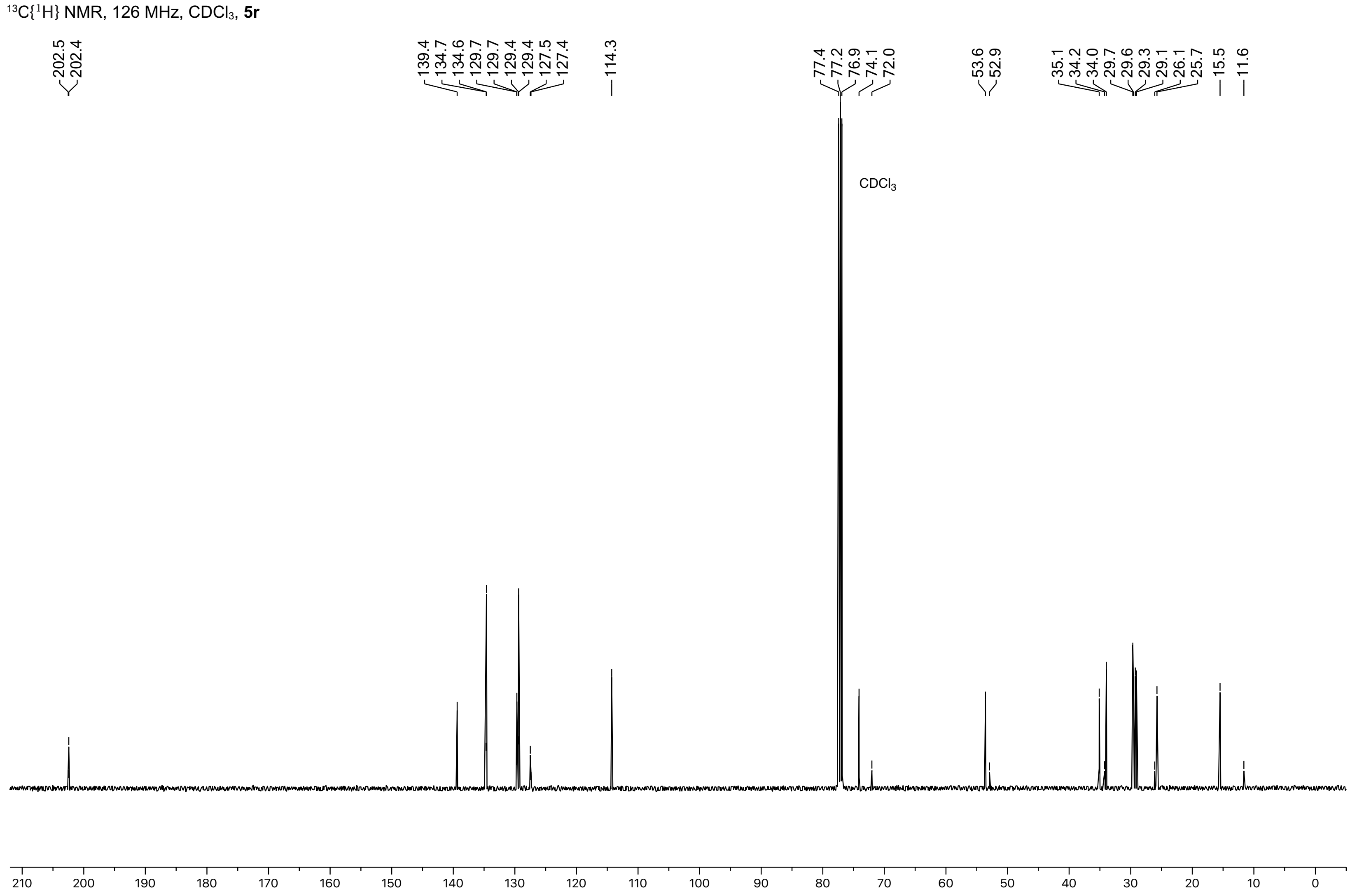

$^{13}C\{^{1}H\}$ NMR, 126 MHz, $CDCl_3$, **5r**
202.5
202.4
139.4
134.7
134.6
129.7
129.7
129.4
129.4
127.5
127.4
114.3
77.4
77.2
76.9
74.1
72.0
53.6
52.9
35.1
34.2
34.0
29.7
29.6
29.3
29.1
26.1
25.7
15.5
11.6
$CDCl_3$
210
200
190
180
170
160
150
140
130
120
110
100
90
80
70
60
50
40
30
20
10
0

^1H NMR, 500 MHz, $CDCl_3$, **5s**

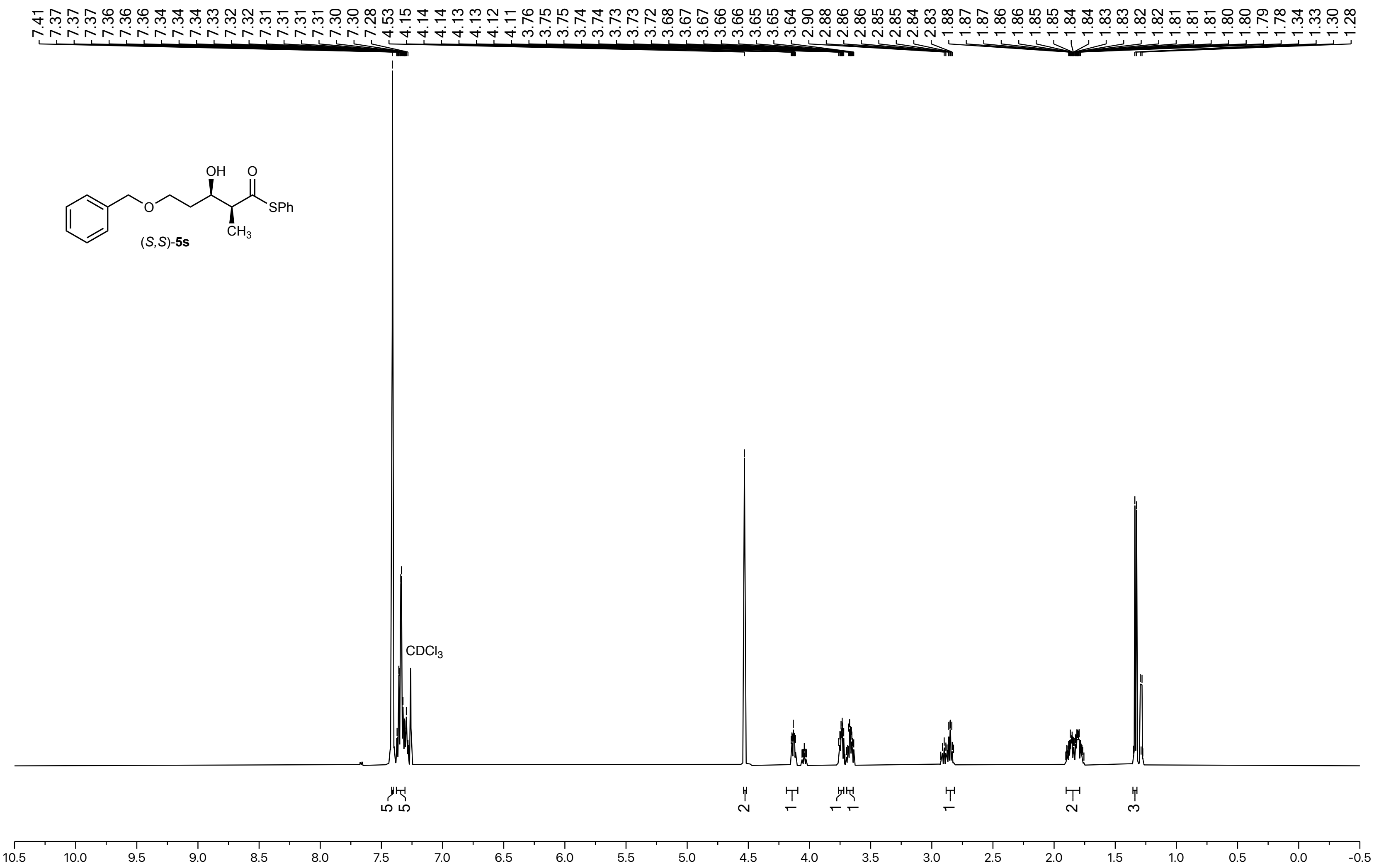

$^{13}C\{^{1}H\}$ NMR, 126 MHz, $CDCl_3$, **5s**

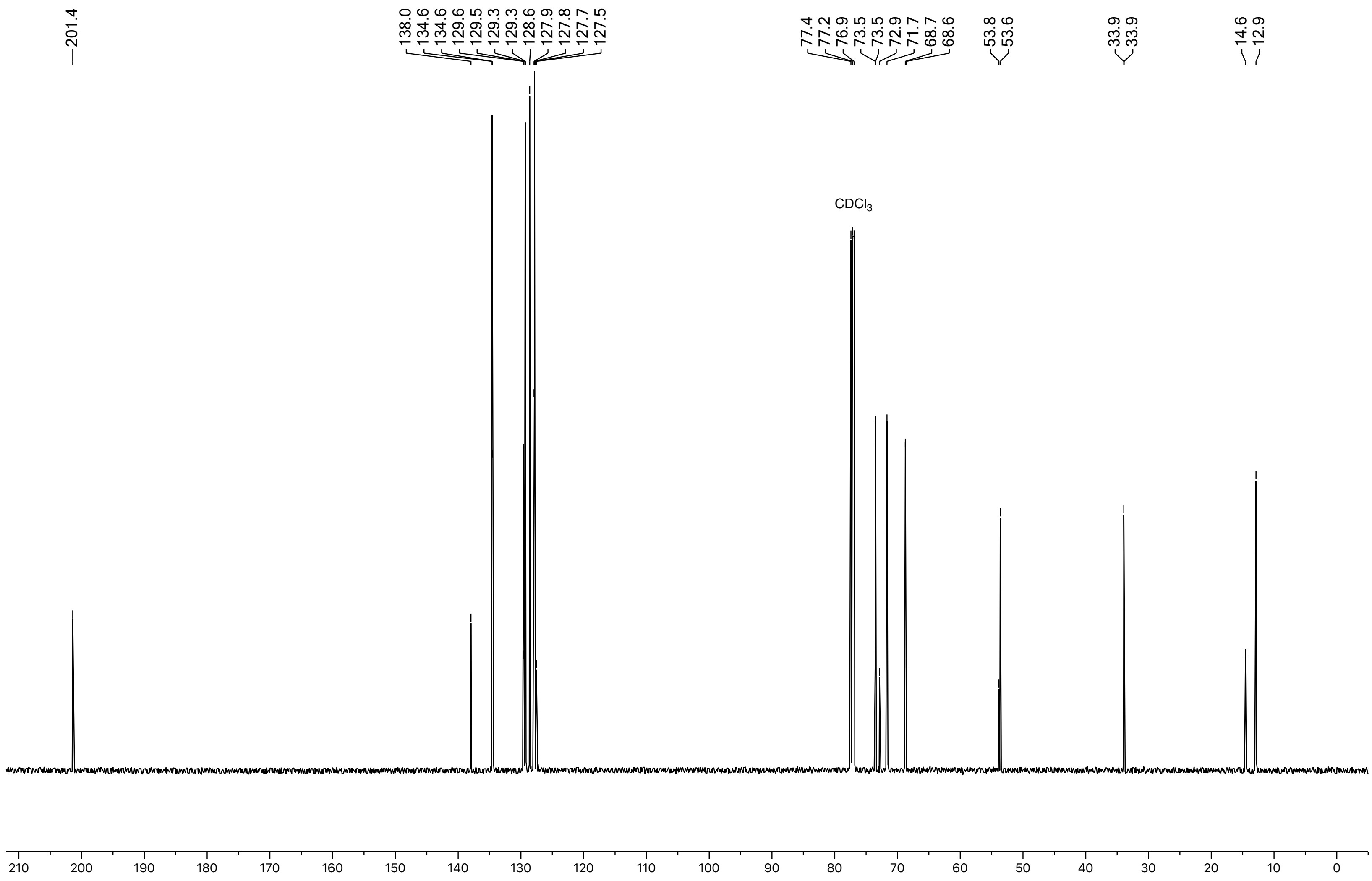

^1H NMR, 500 MHz, $CDCl_3$, **6s**

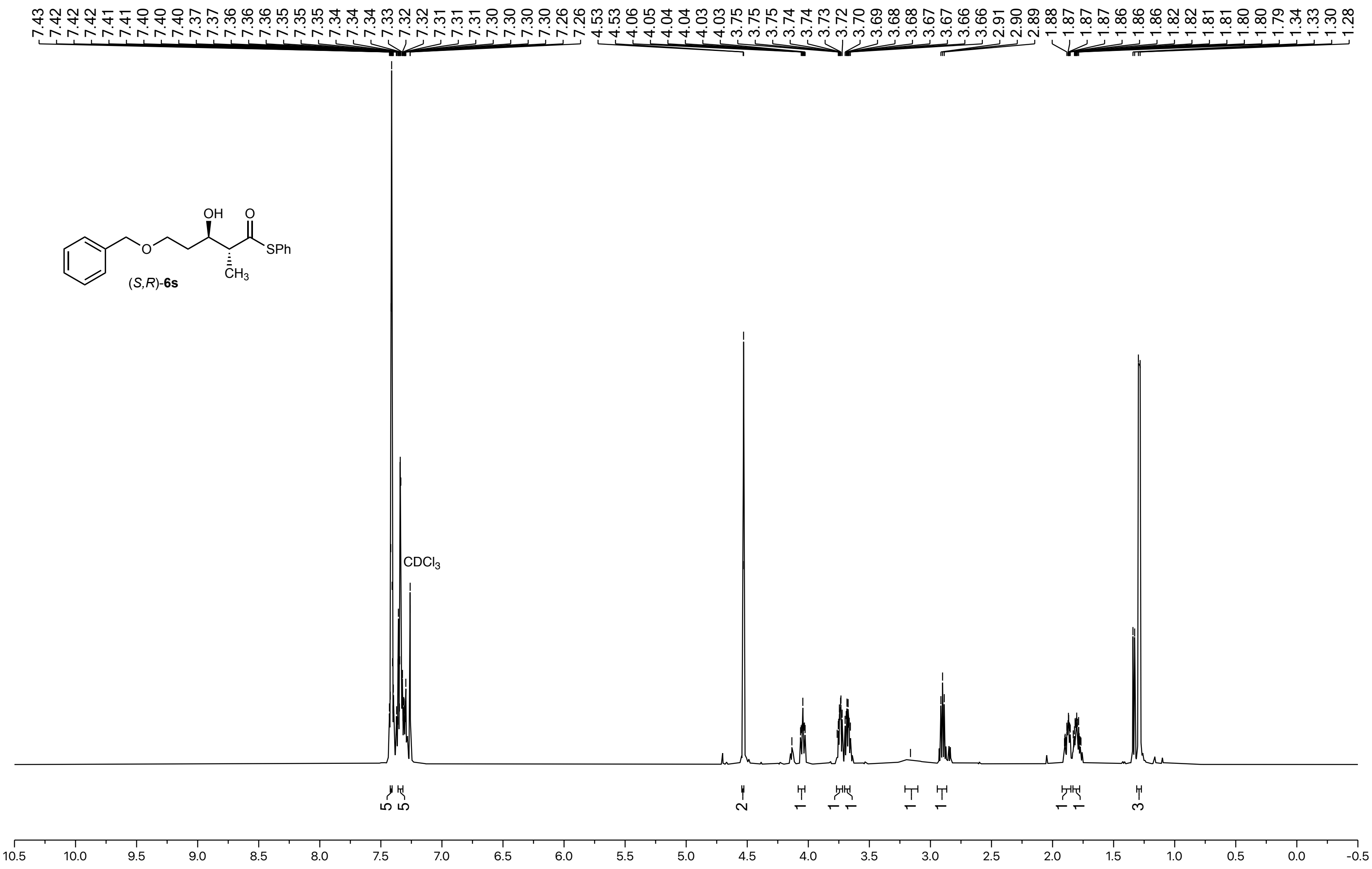

$^{13}C\{^{1}H\}$ NMR, 126 MHz, $CDCl_3$, **6s**

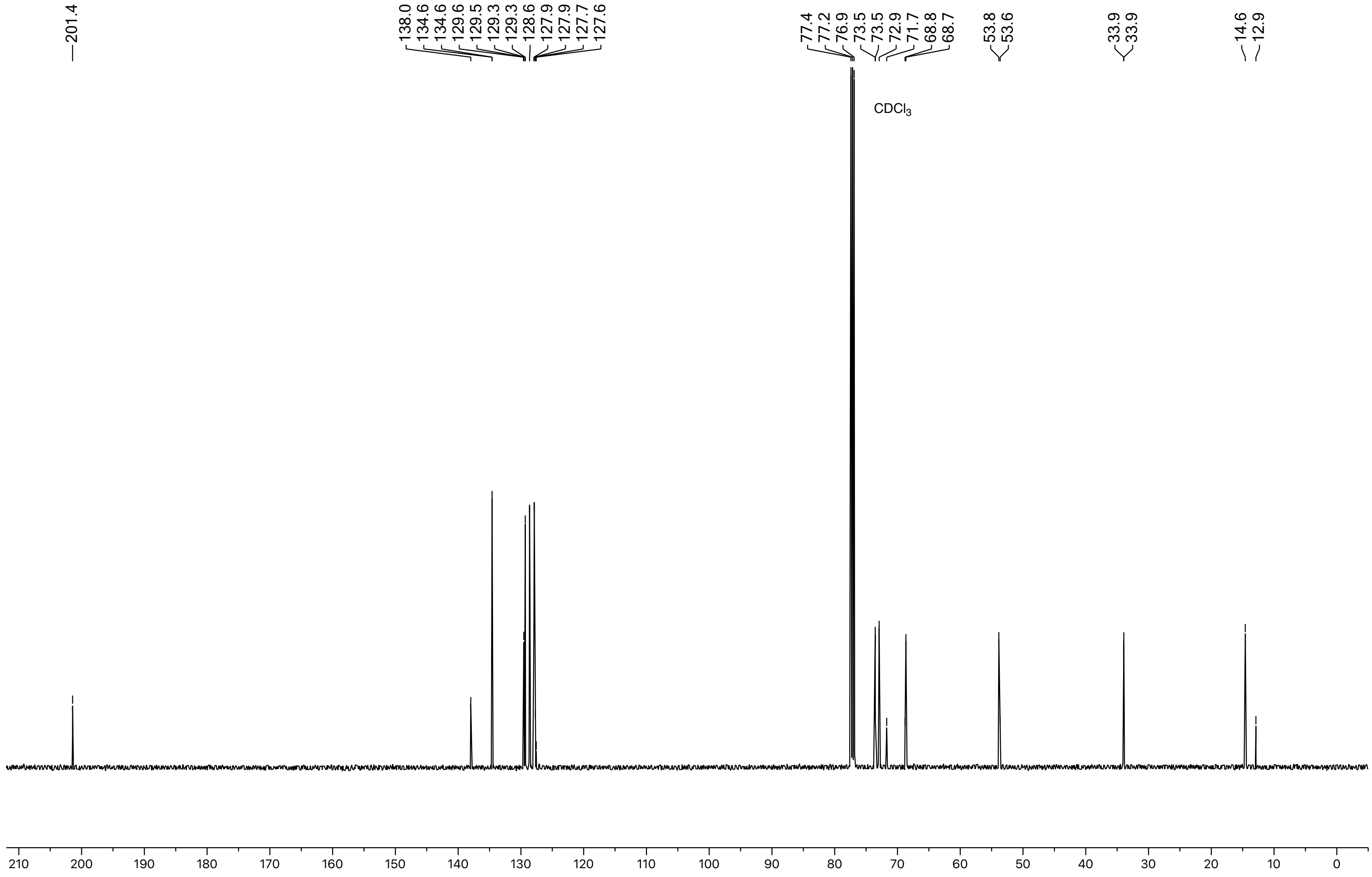

^1H NMR, 500 MHz, $CDCl_3$, **5t**

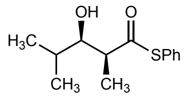

(*S*,*S*)-**5t**

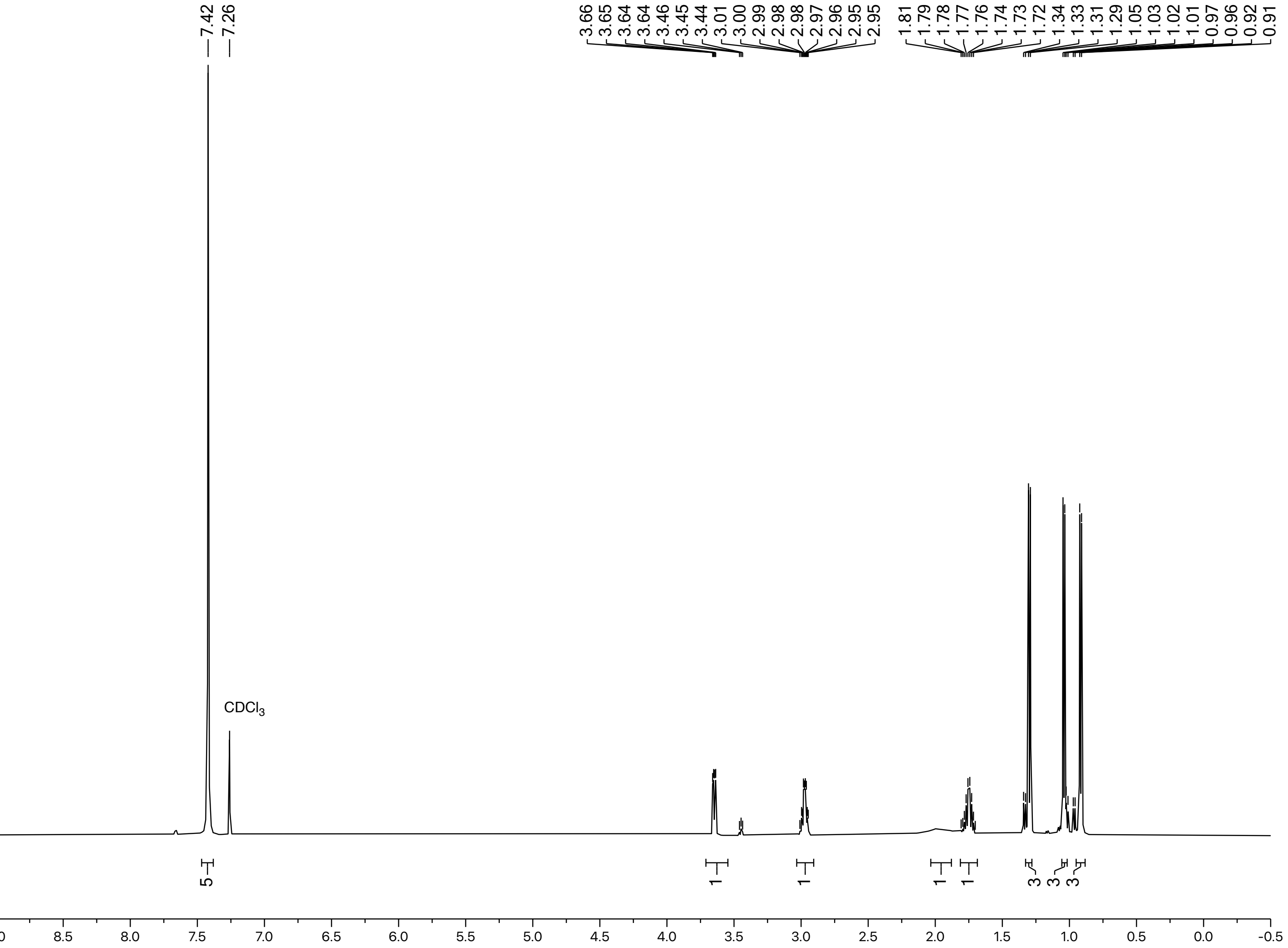

$^{13}C\{^{1}H\}$ NMR, 126 MHz, $CDCl_3$, **5t**

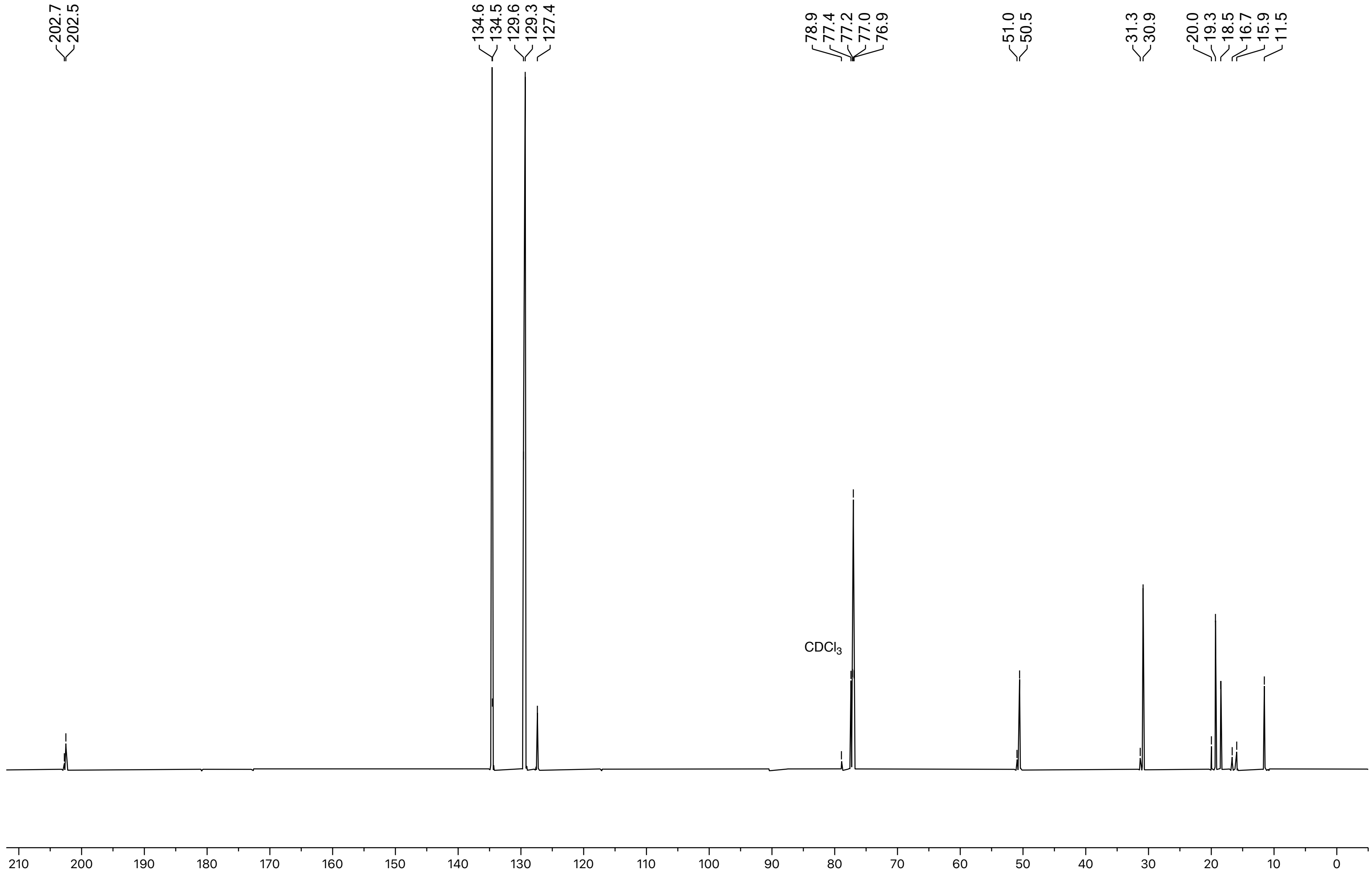

^{1}H NMR, 500 MHz, $CDCl_3$, **6t**

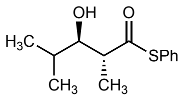

(*S*,*R*)-**6t**

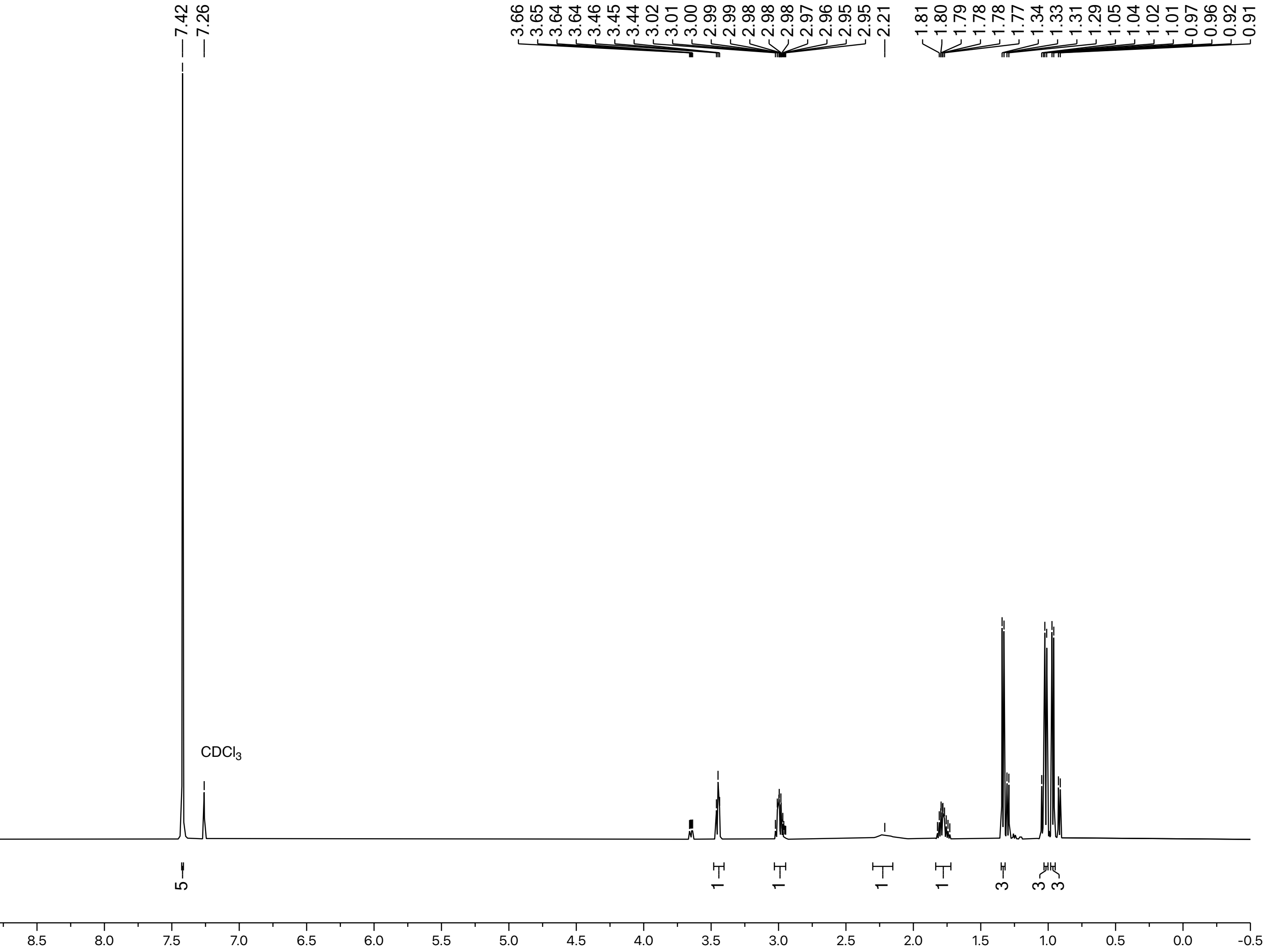

$^{13}C\{^{1}H\}$ NMR, 126 MHz, $CDCl_3$, **6t**

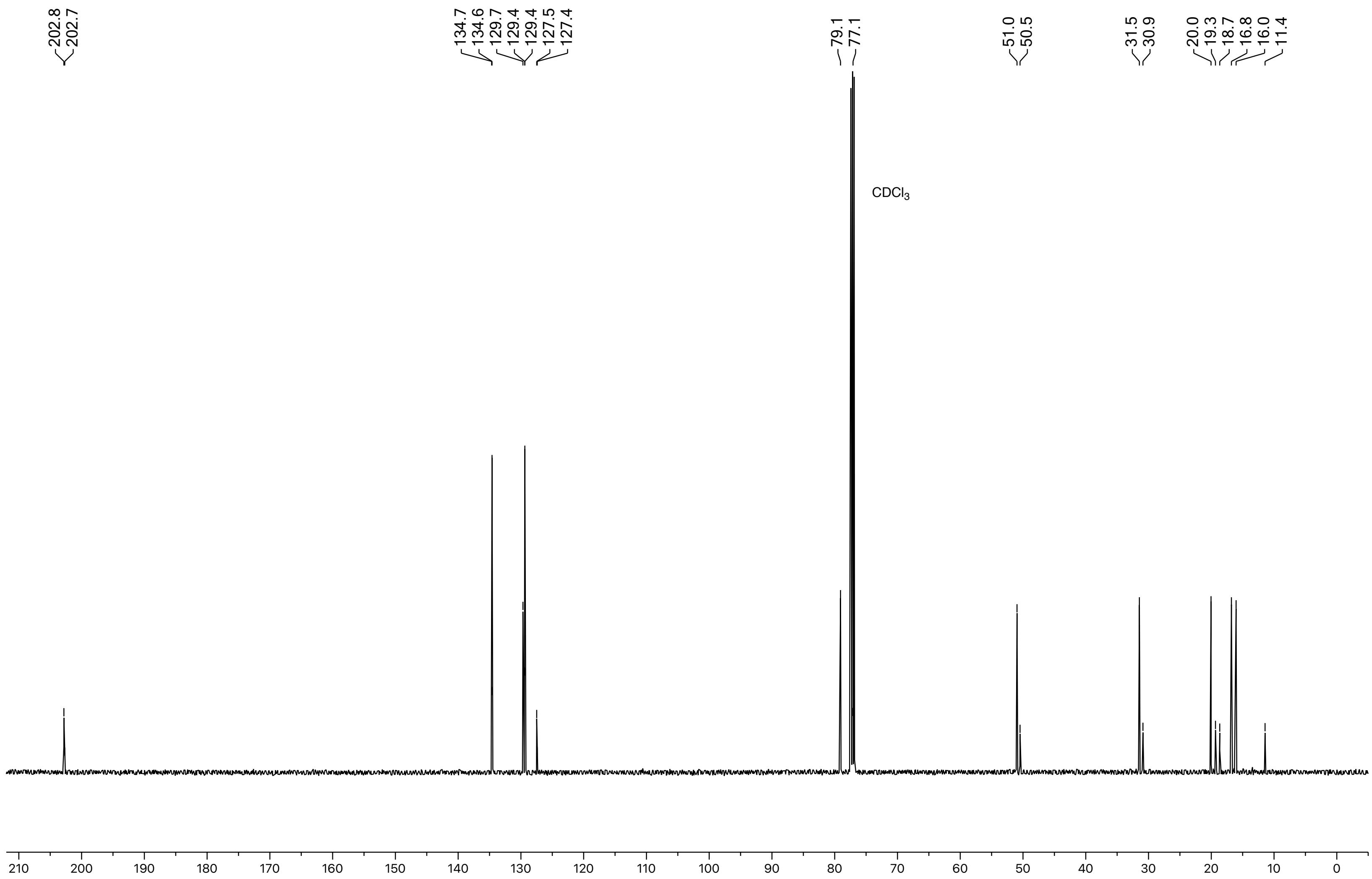

^{1}H NMR, 500 MHz, $CDCl_3$, **5u**

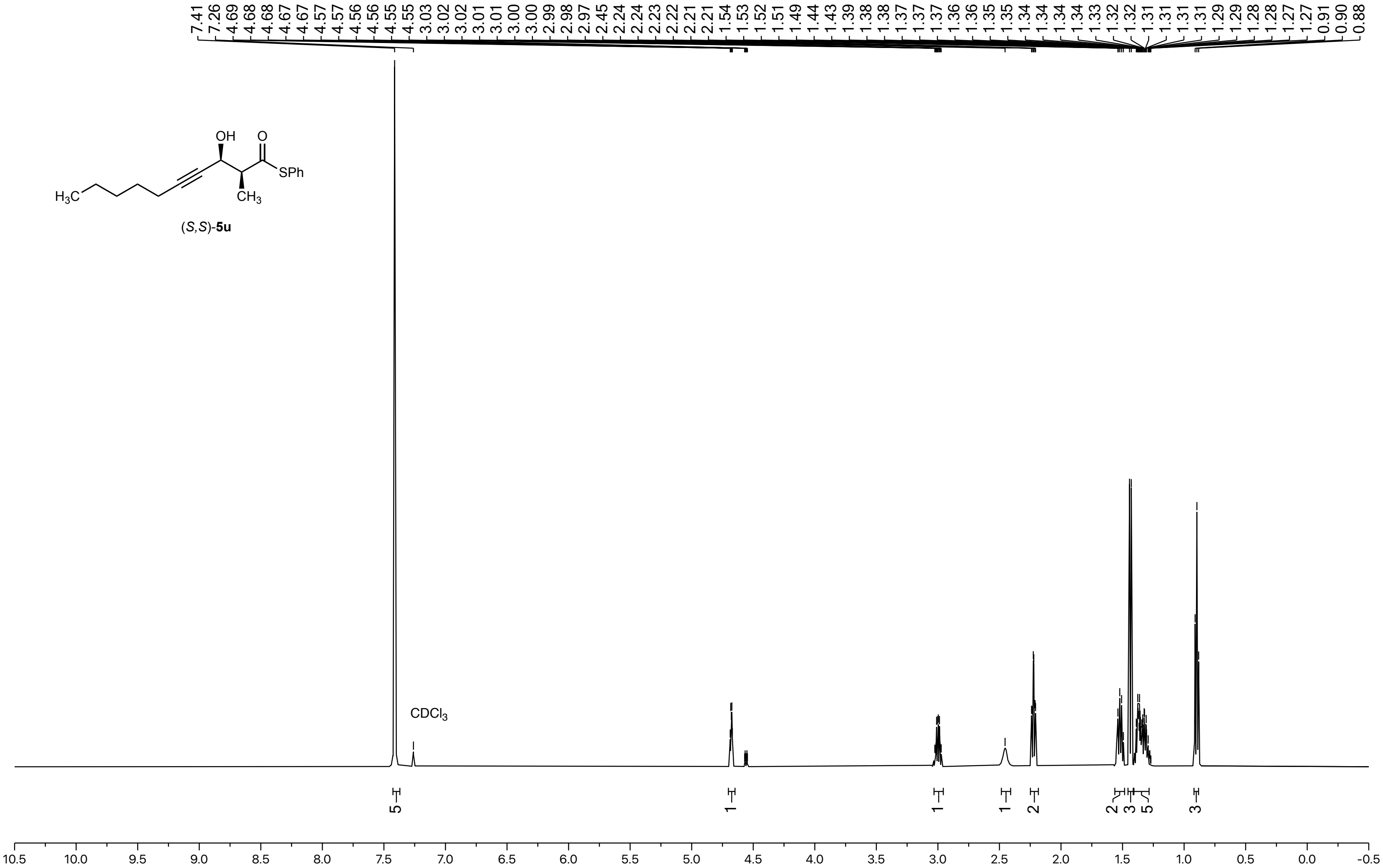

$^{13}C\{^{1}H\}$ NMR, 126 MHz, $CDCl_3$, **5u**

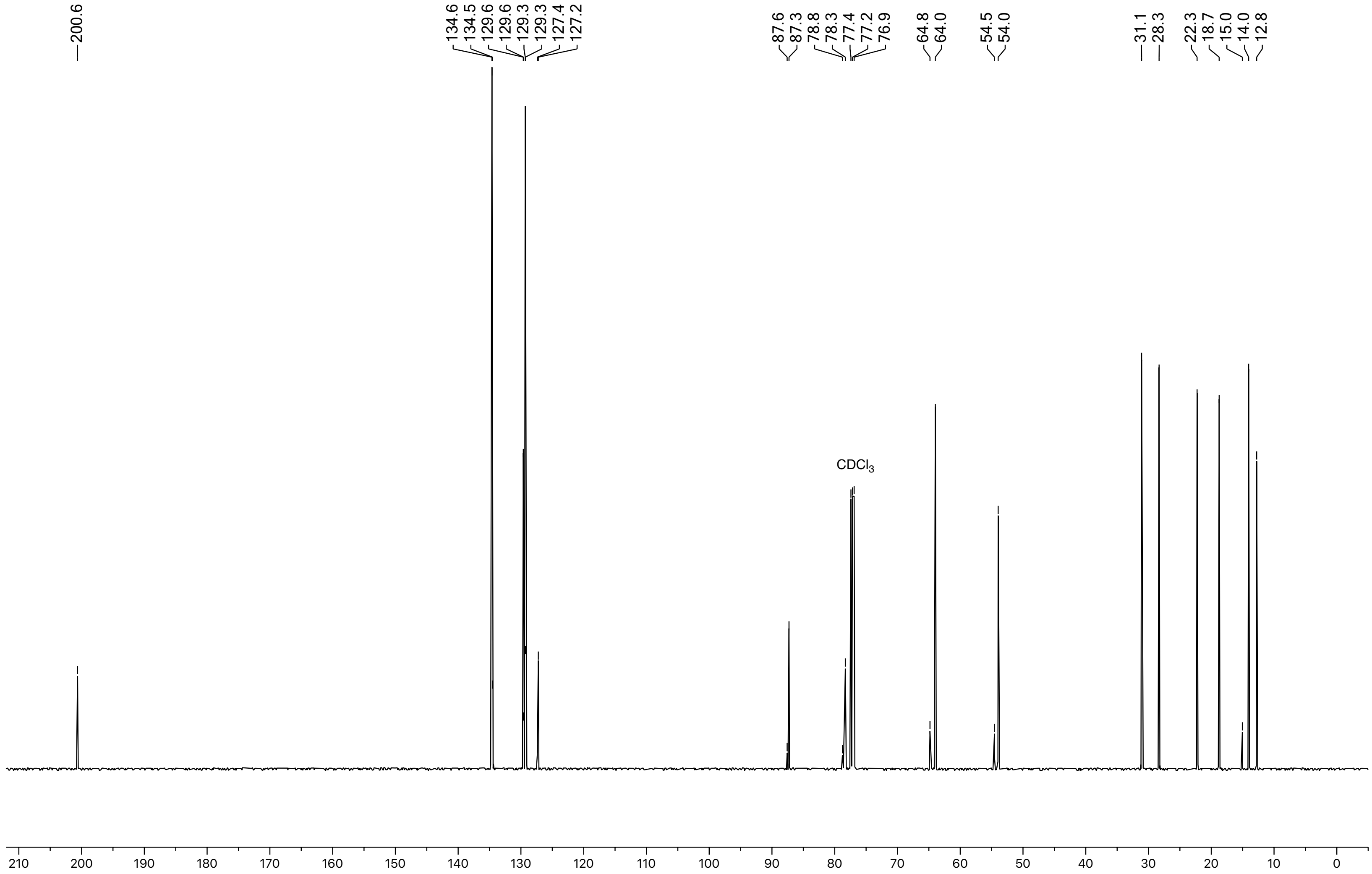

^{1}H NMR, 500 MHz, $CDCl_3$, **6u**

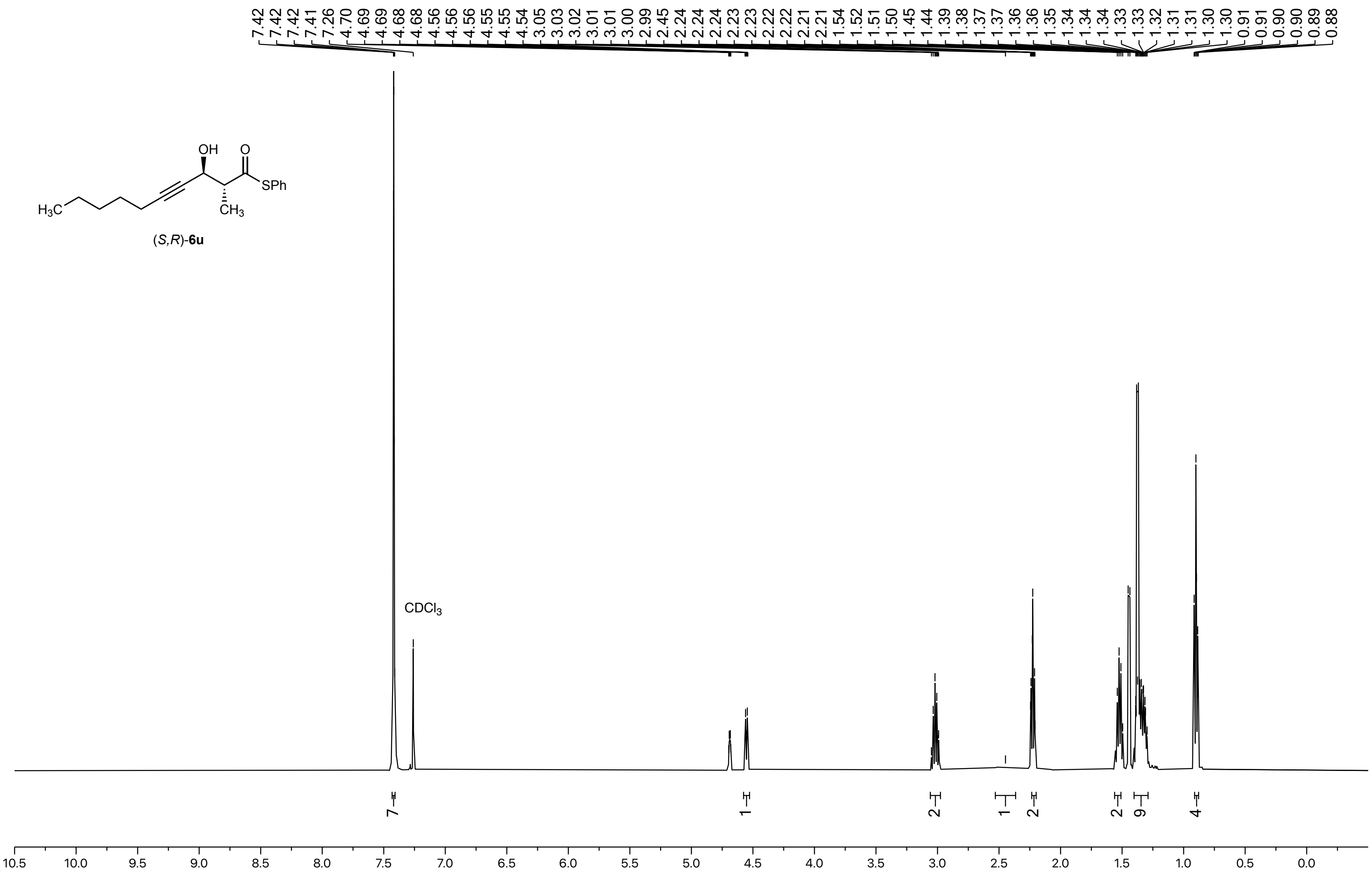

$^{13}C\{^1H\}$ NMR, 126 MHz, $CDCl_3$, **6u**

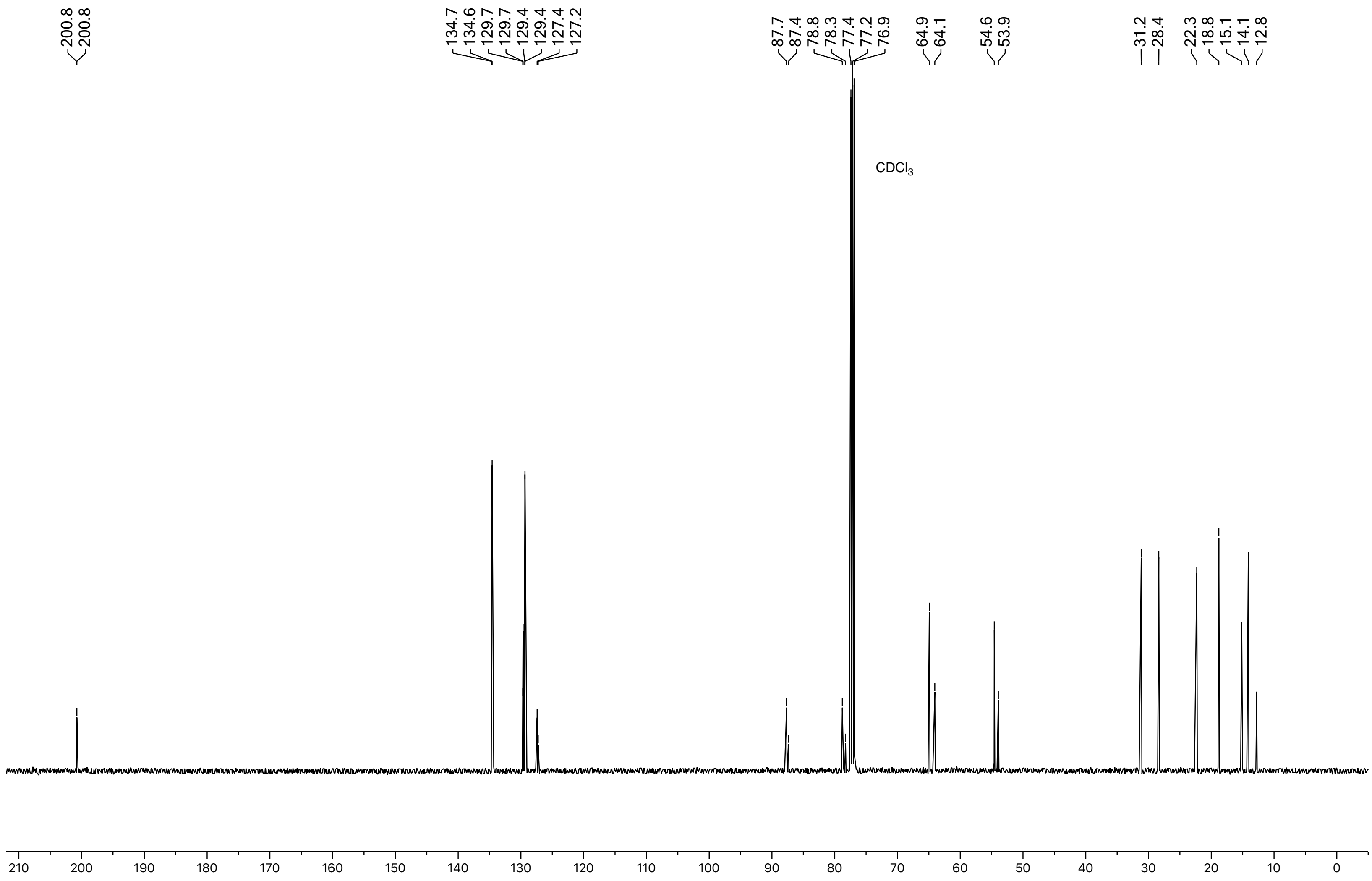

^{1}H NMR, 500 MHz, $CDCl_3$, **5v**

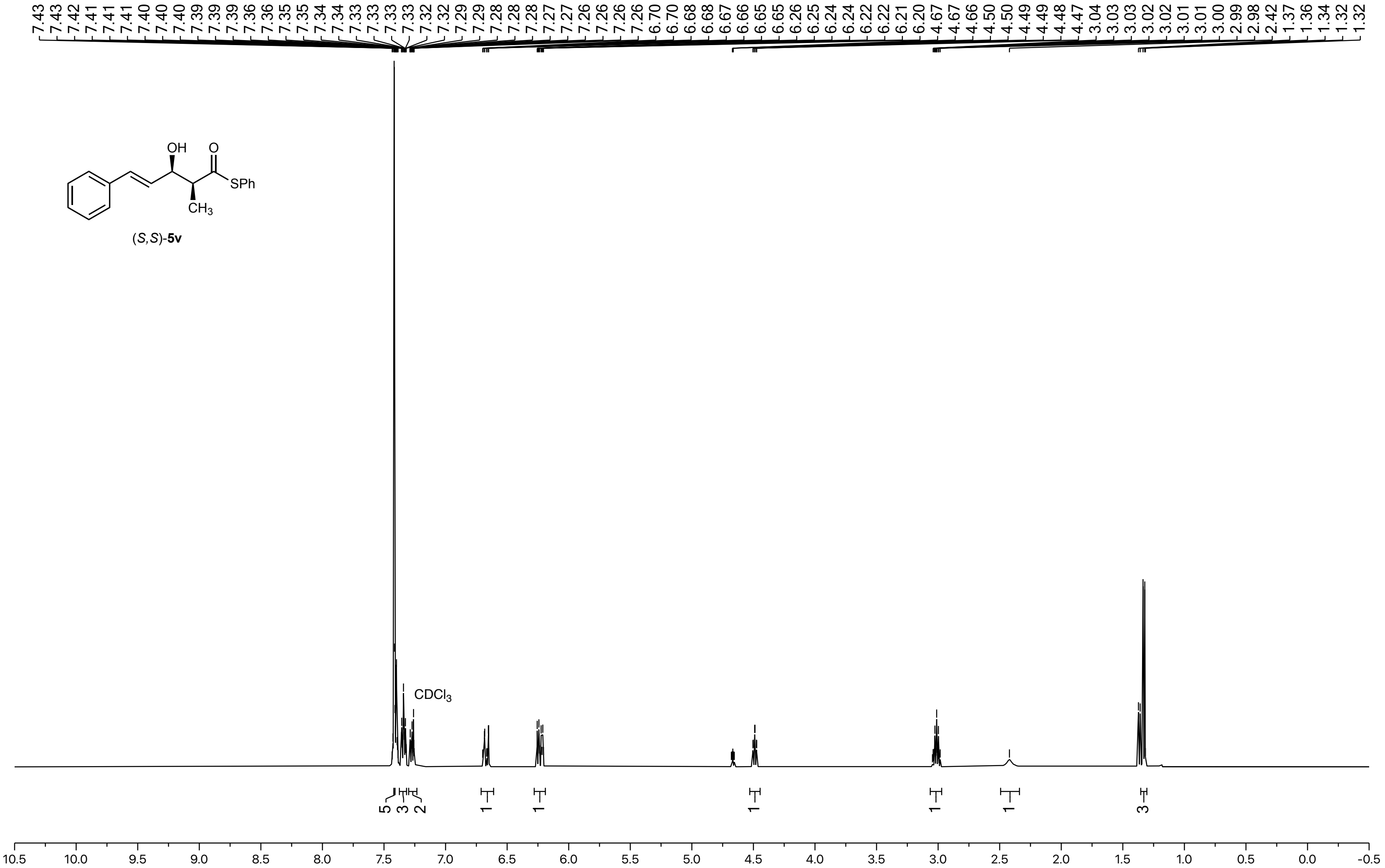

(*S*,*S*)-**5v**

$^{13}C\{^{1}H\}$ NMR, 126 MHz, $CDCl_3$, **5v**

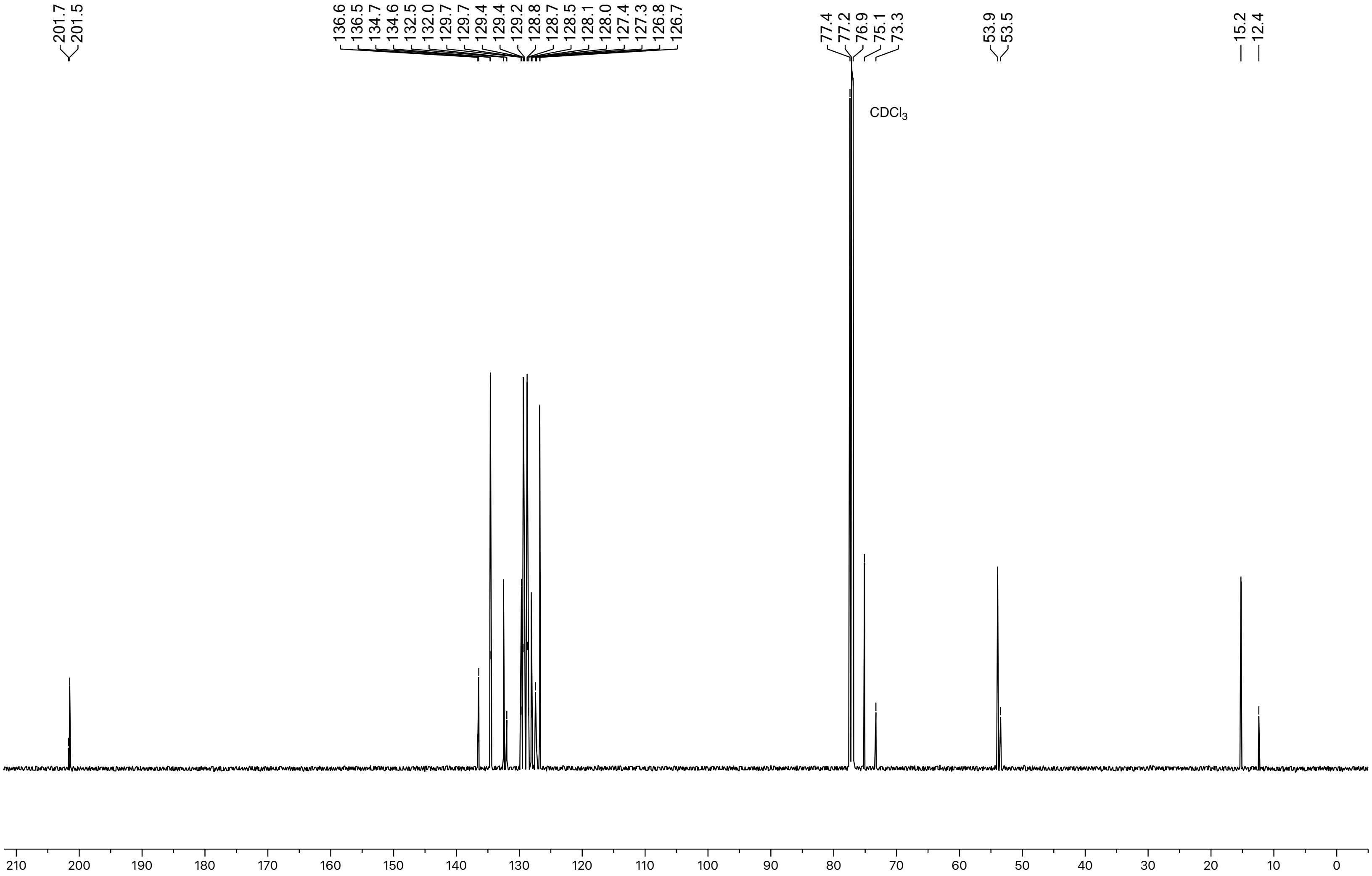

^{1}H NMR, 500 MHz, $CDCl_3$, **6v**

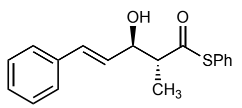

(*S*,*R*)-**6v**

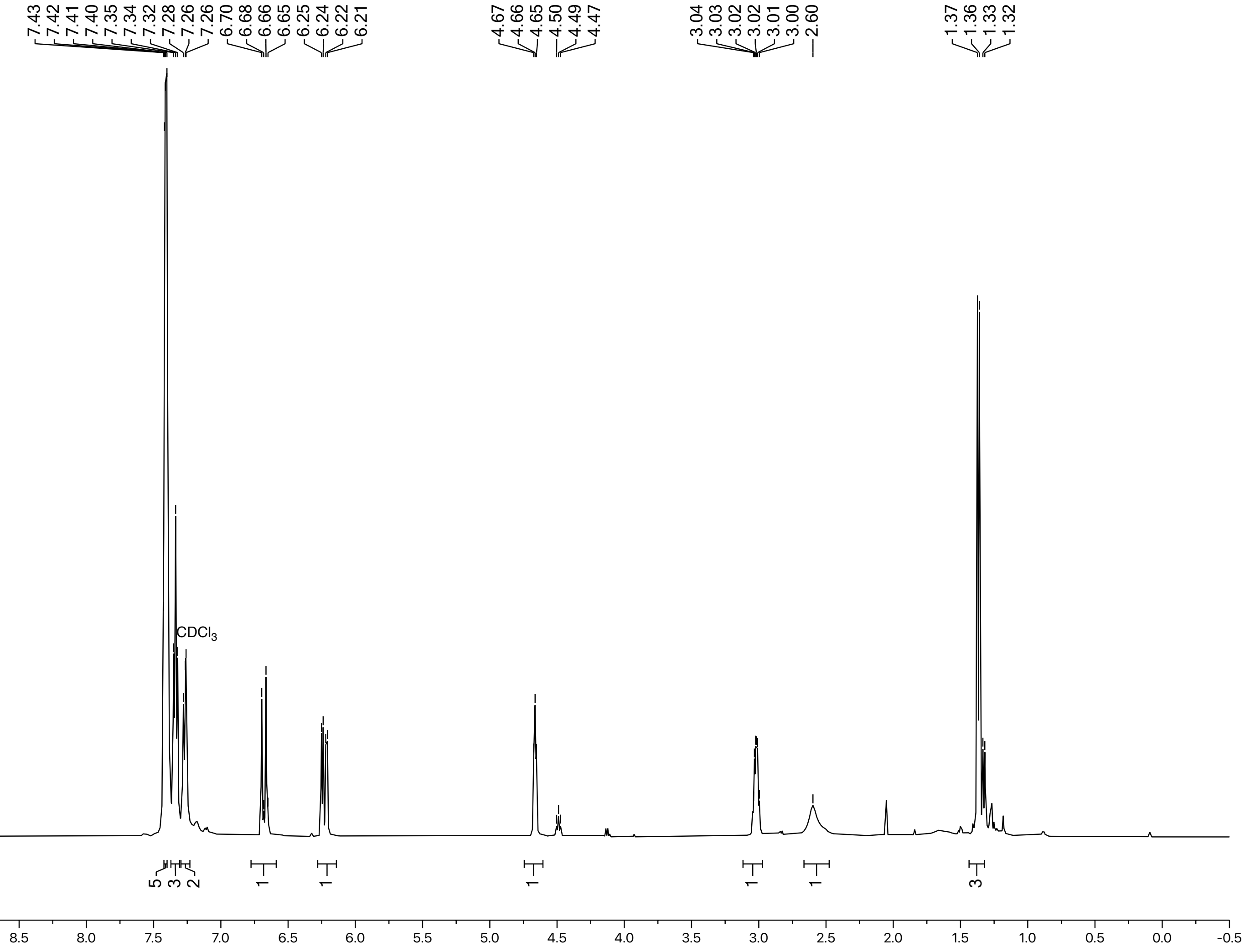

$^{13}C\{^{1}H\}$ NMR, 126 MHz, $CDCl_3$, **6v**

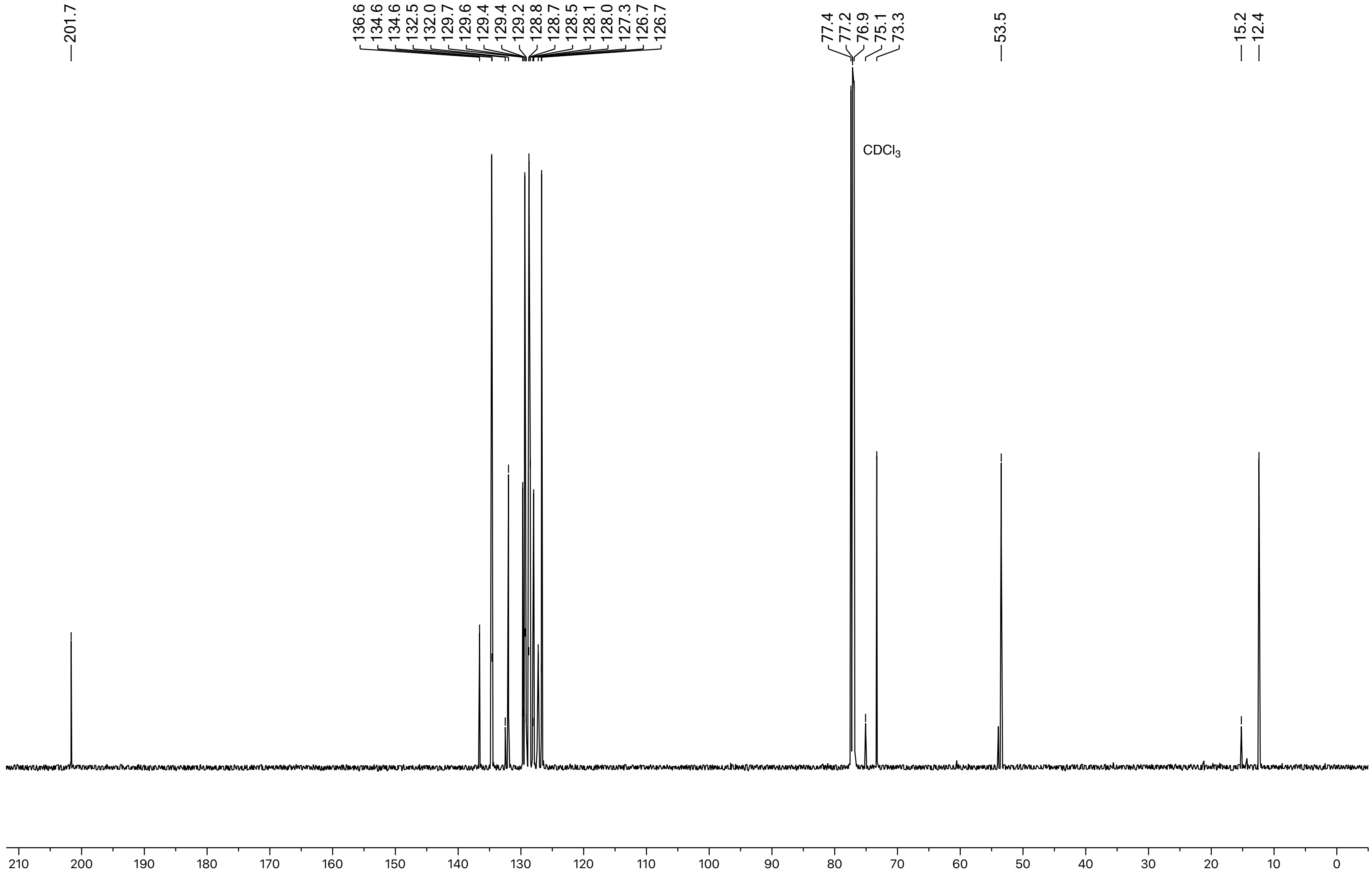

^1H NMR, 500 MHz, $CDCl_3$, **S5a**

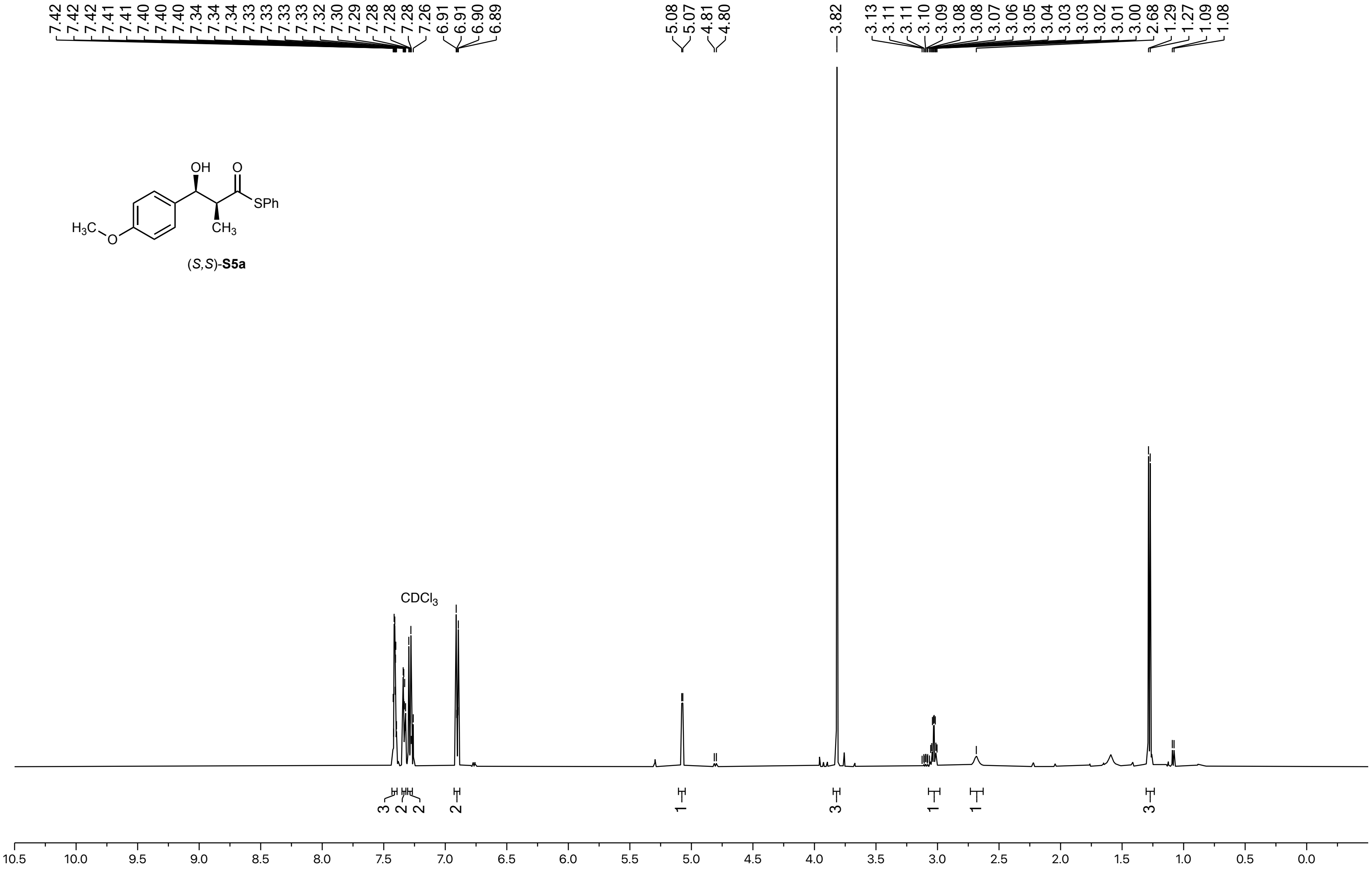

$^{13}C\{^{1}H\}$ NMR, 126 MHz, $CDCl_3$, **S5a**

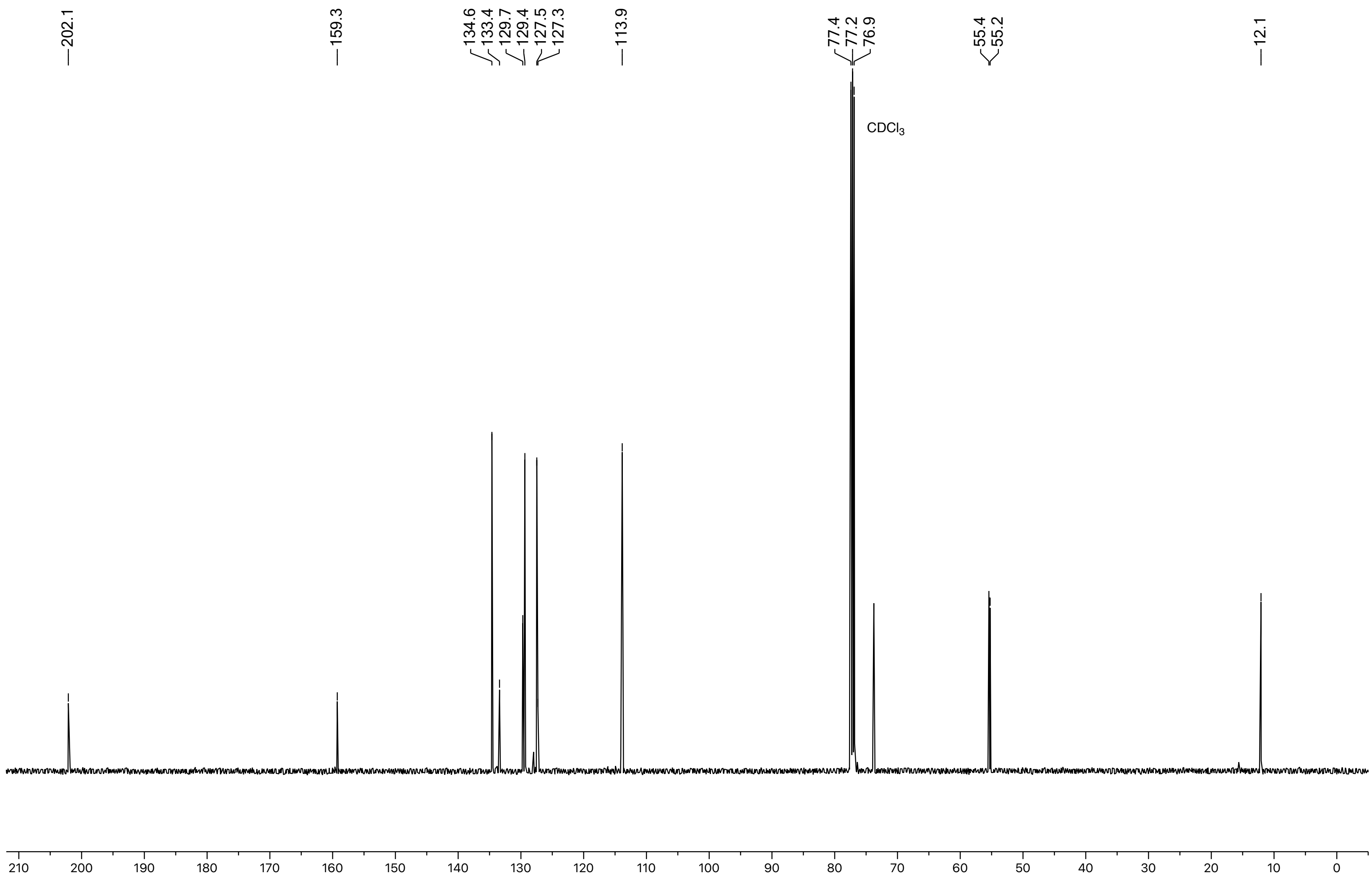

^{1}H NMR, 500 MHz, $CDCl_3$, **S6a**

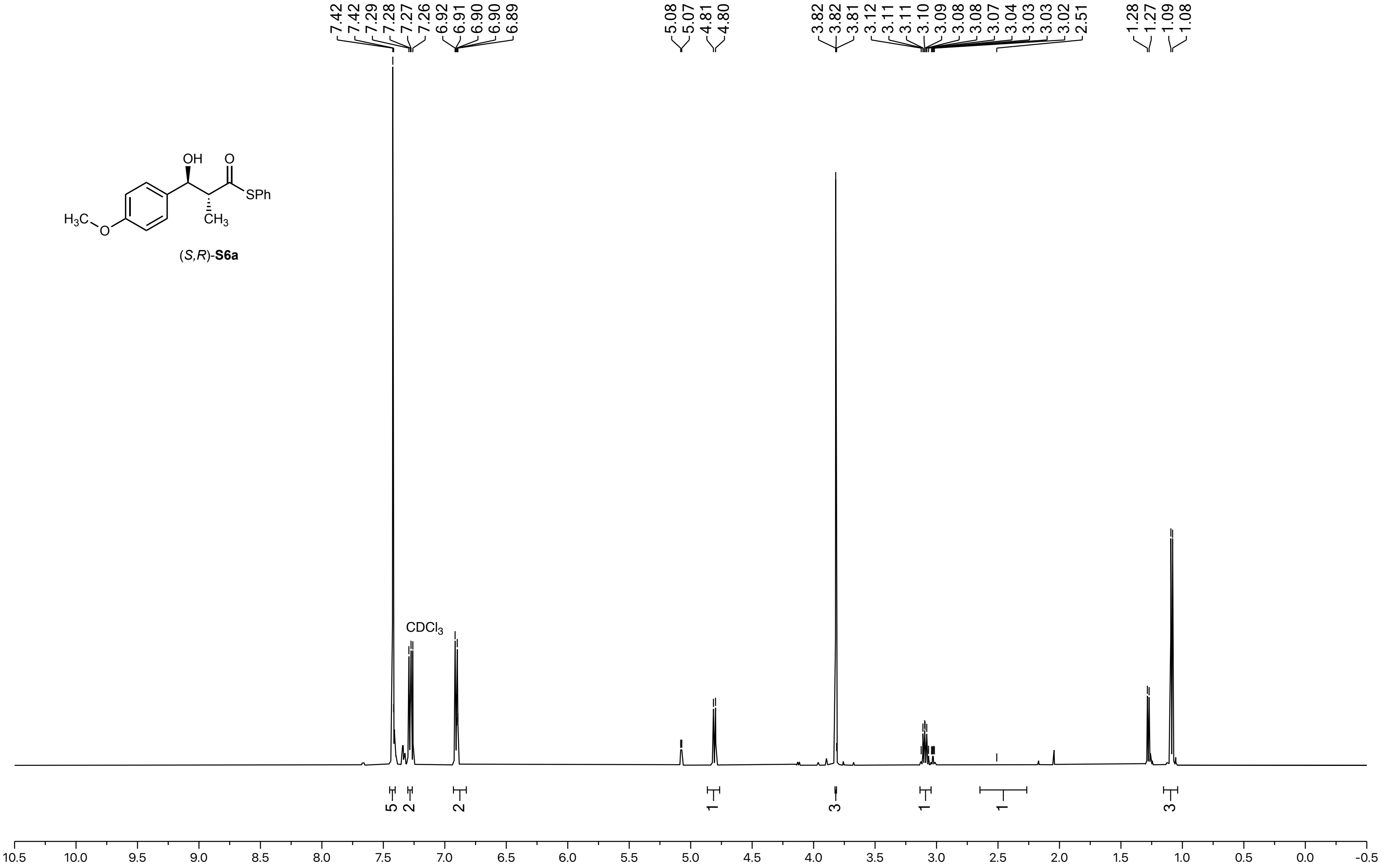

$^{13}C\{^{1}H\}$ NMR, 126 MHz, $CDCl_3$, **S6a**

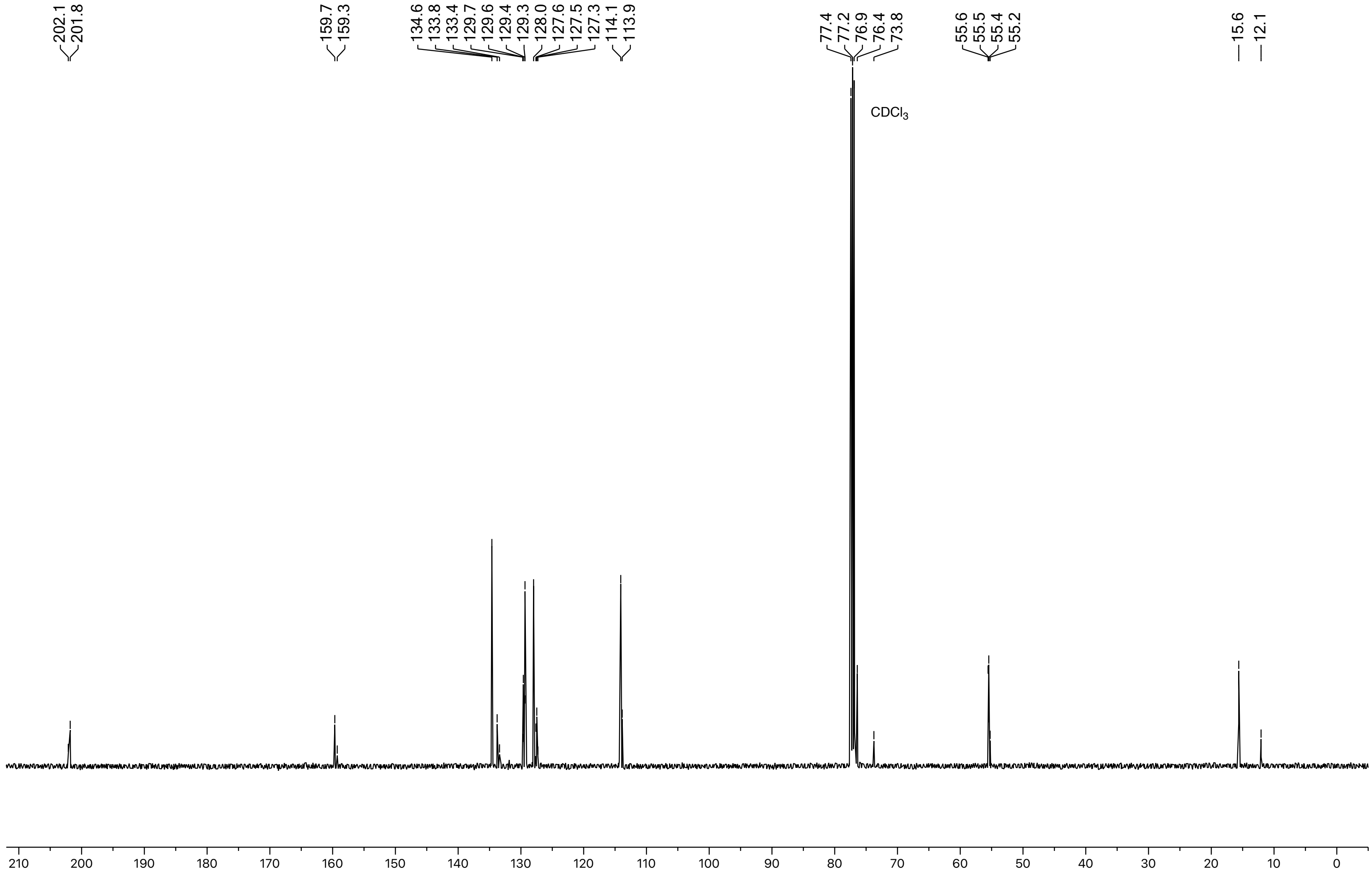

^{1}H NMR, 500 MHz, $CDCl_3$, **S5b**

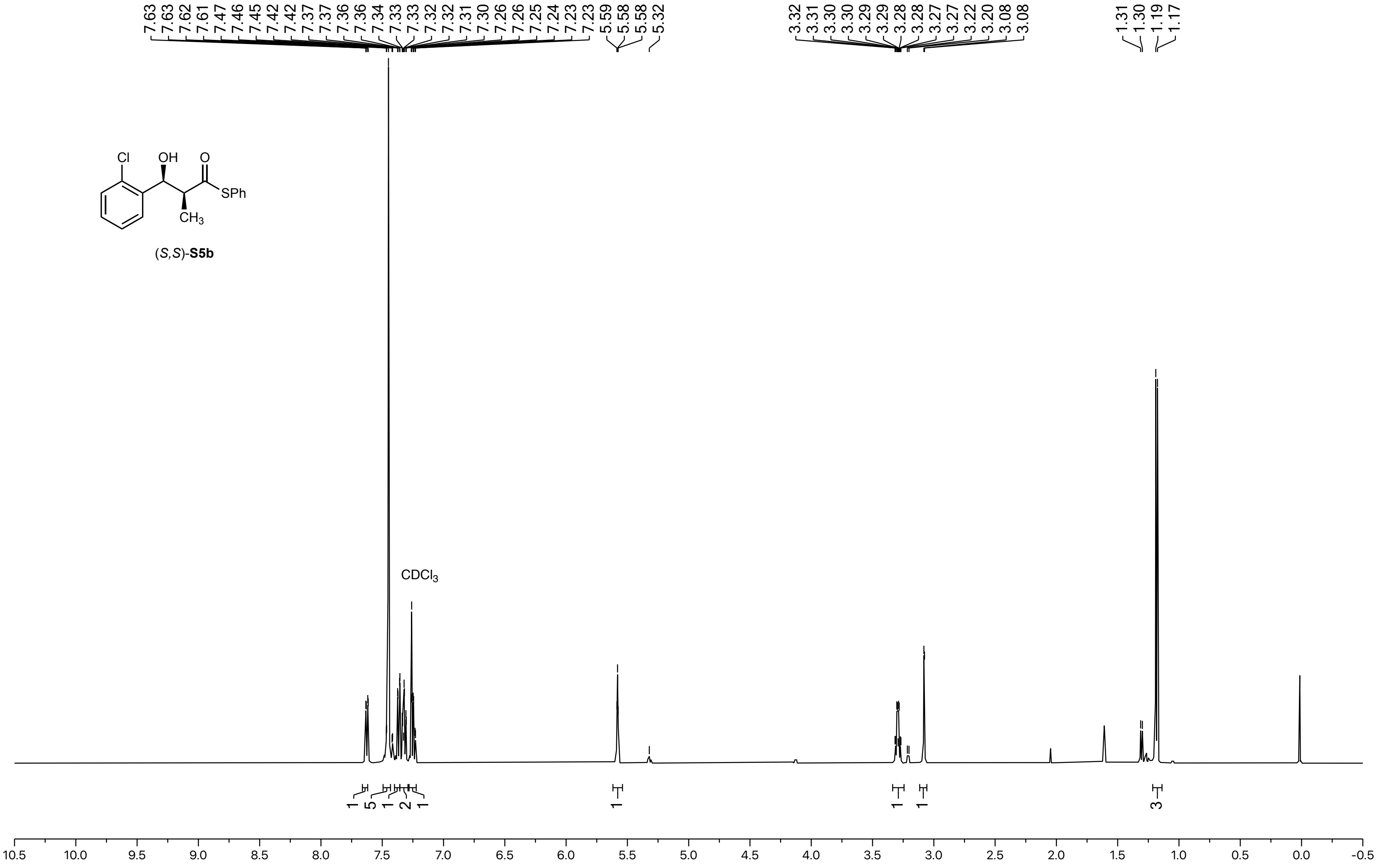

$^{13}C\{^1H\}$ NMR, 126 MHz, $CDCl_3$, **S5b**

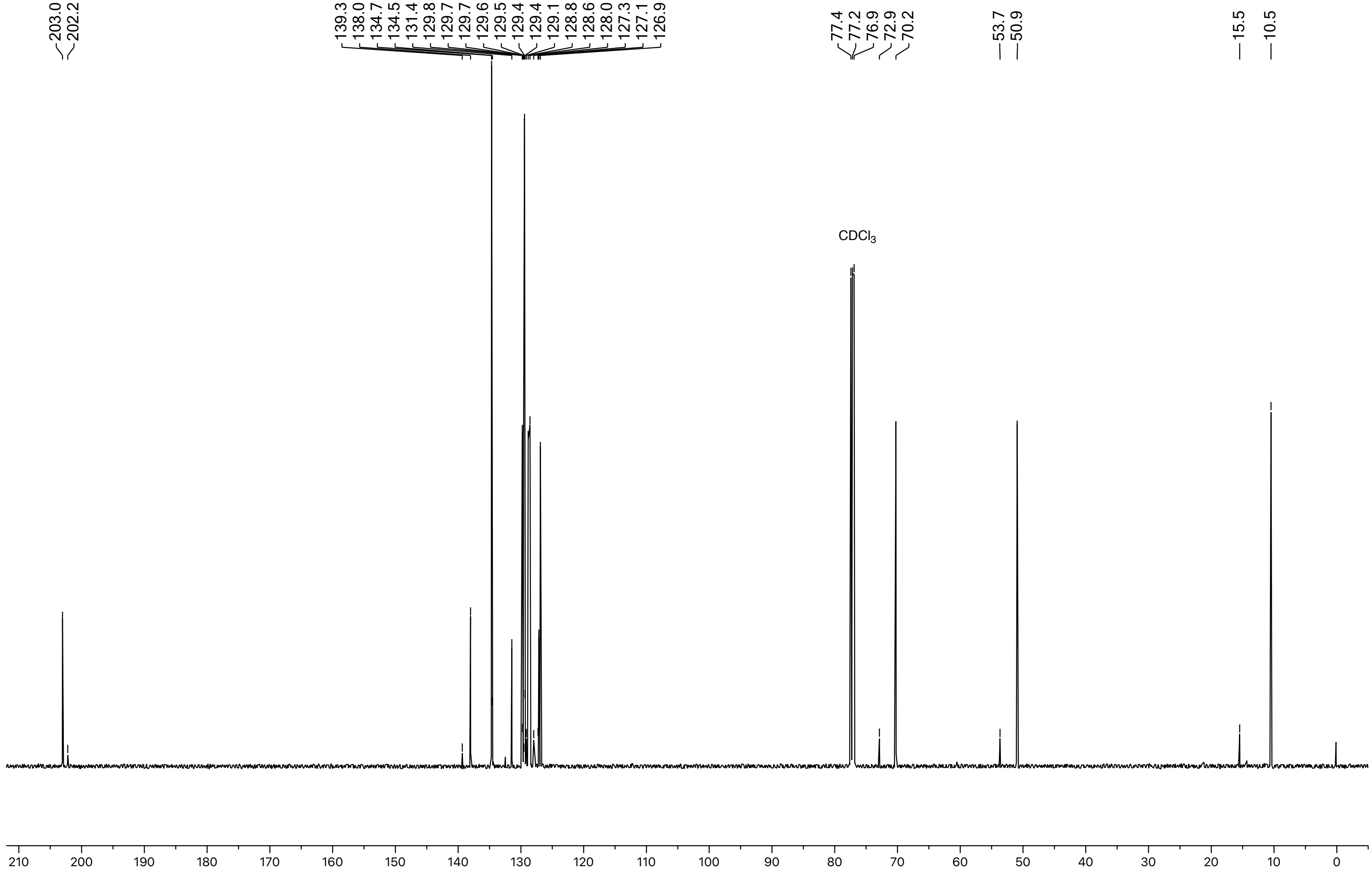

^1H NMR, 500 MHz, $CDCl_3$, **S6b**

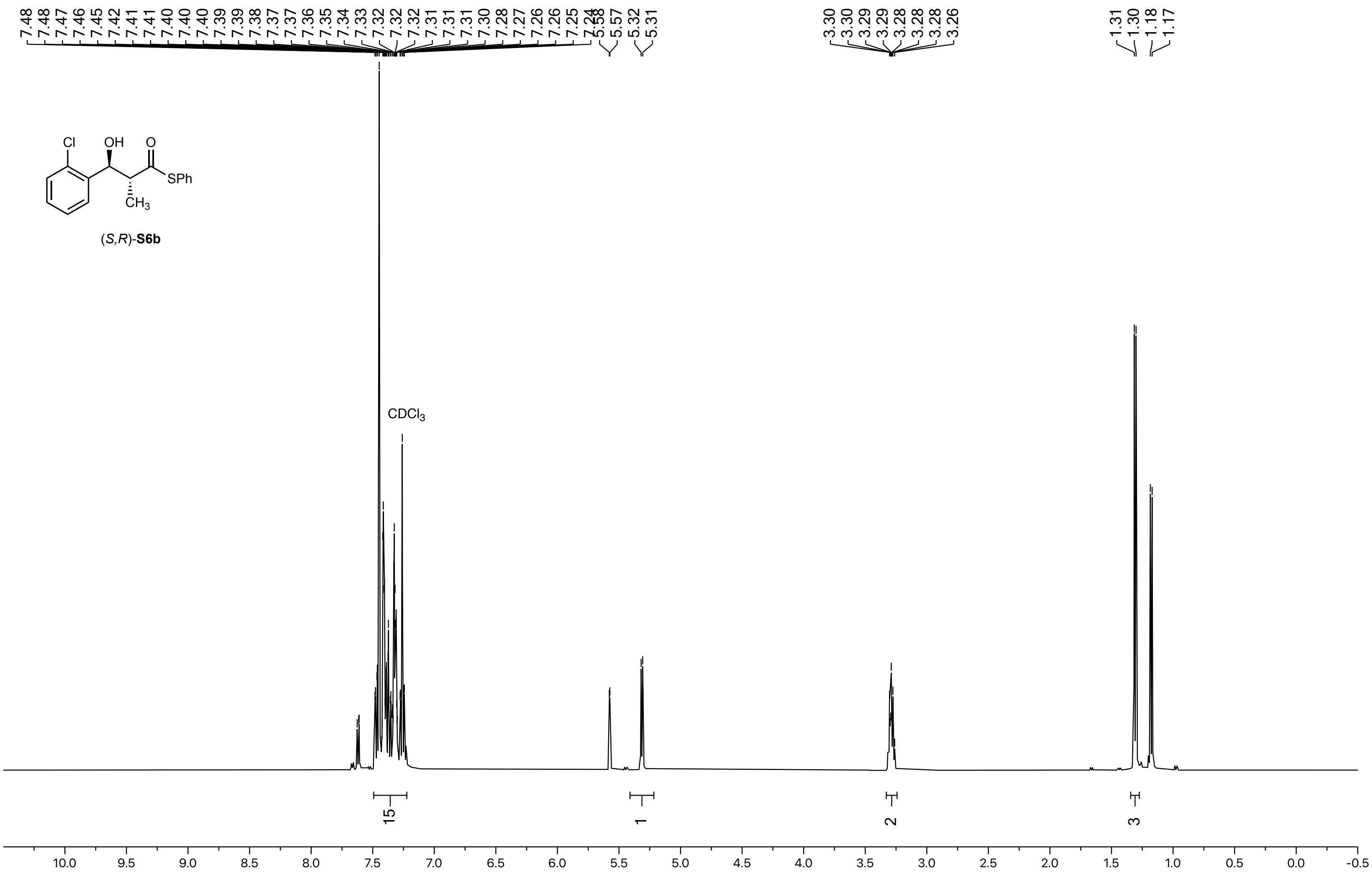

$^{13}C\{^1H\}$ NMR, 126 MHz, $CDCl_3$, **S6b**

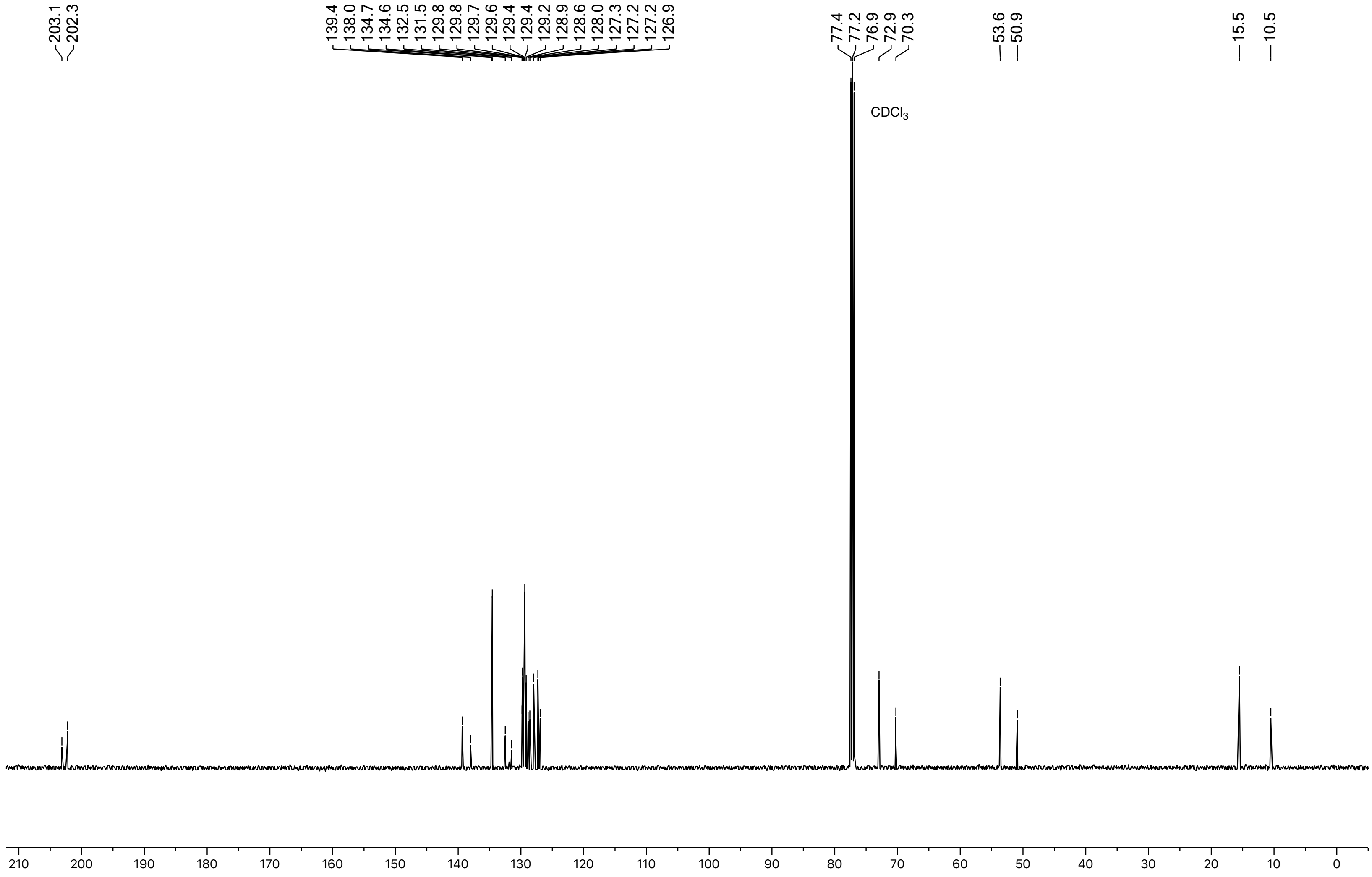

¹H NMR, 500 MHz, $CDCl_3$, **S5c**

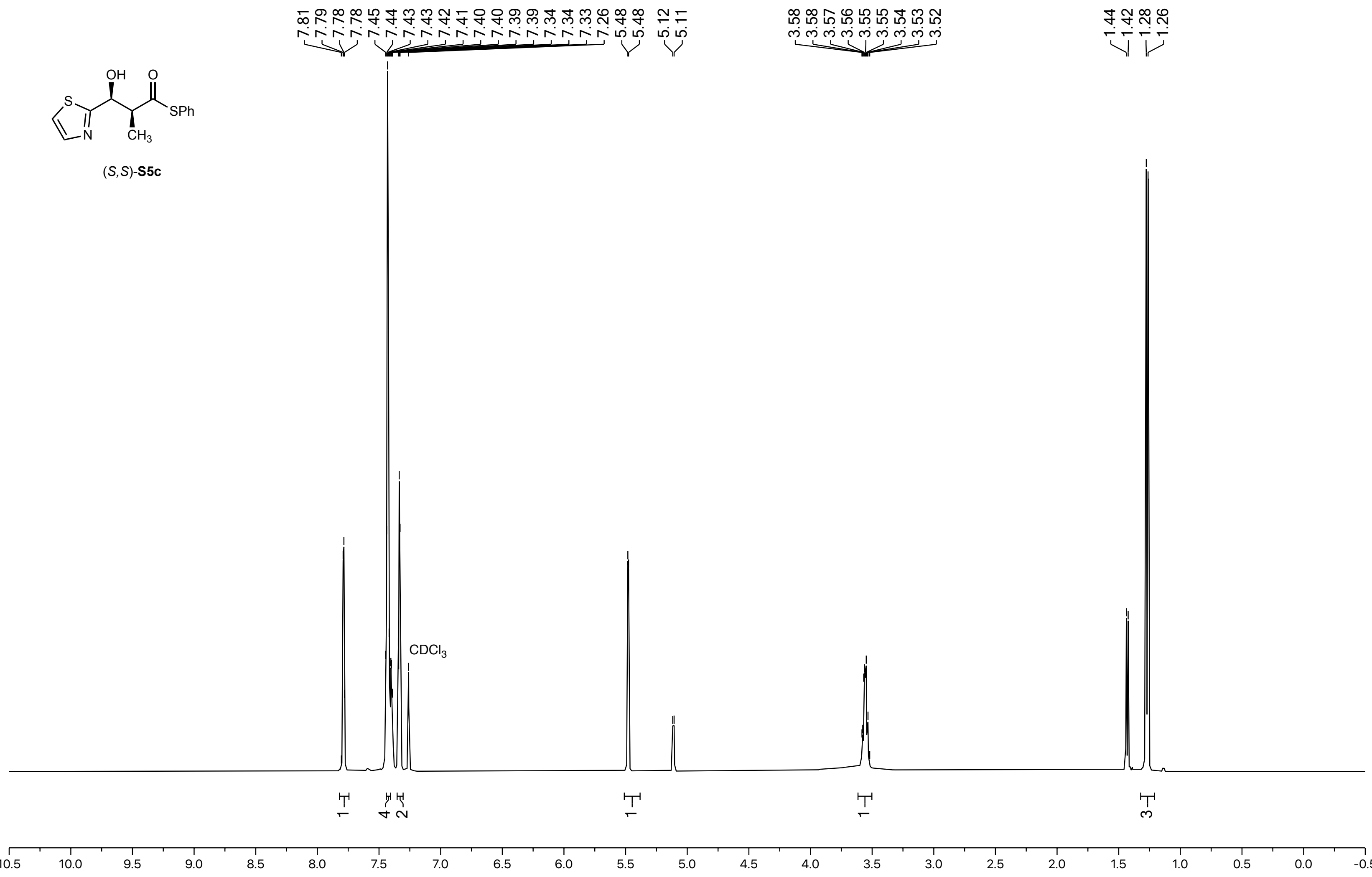

$^{13}C\{^{1}H\}$ NMR, 126 MHz, $CDCl_3$, **S5c**

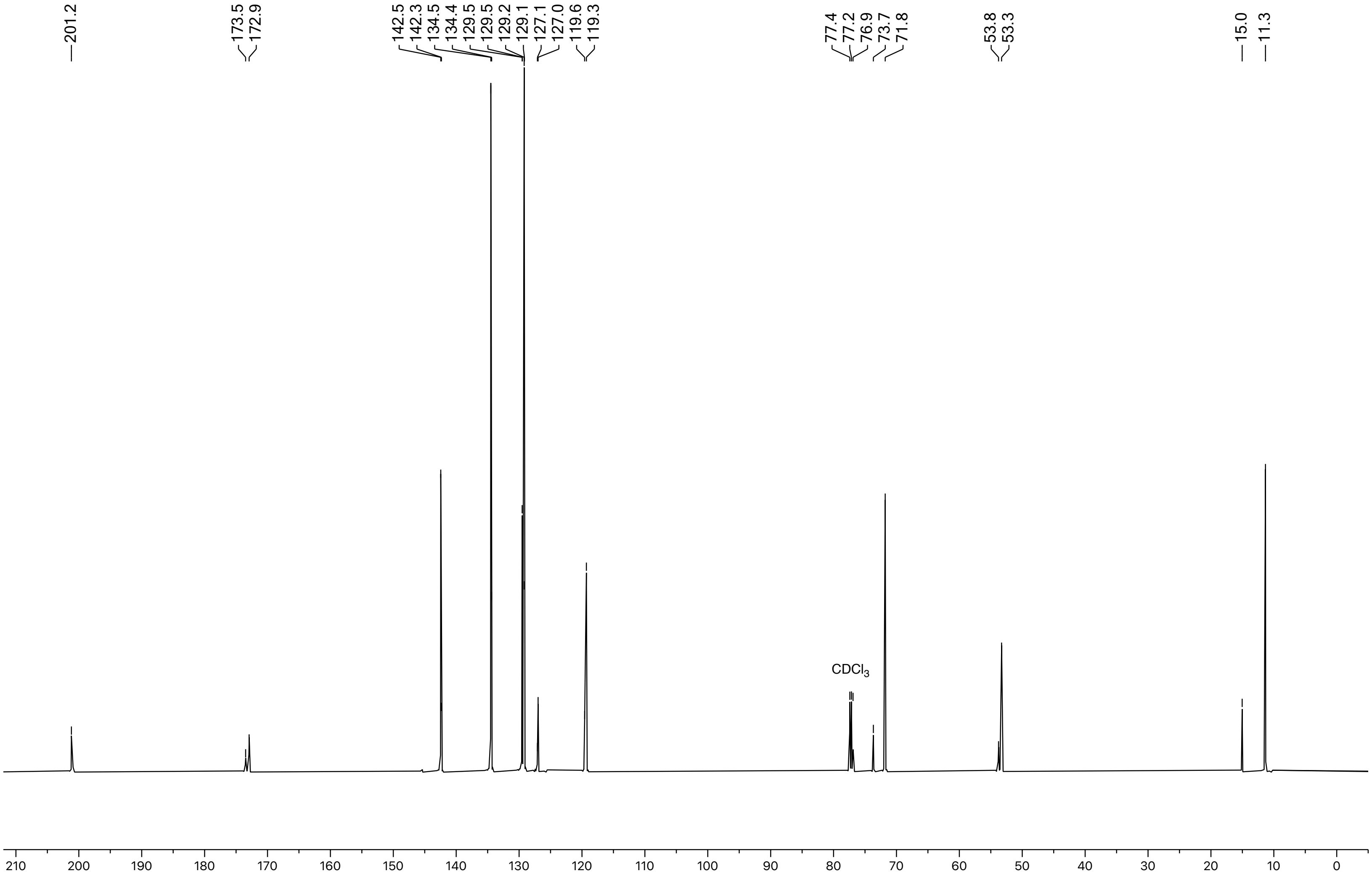

^{1}H NMR, 500 MHz, $CDCl_3$, **S6c**

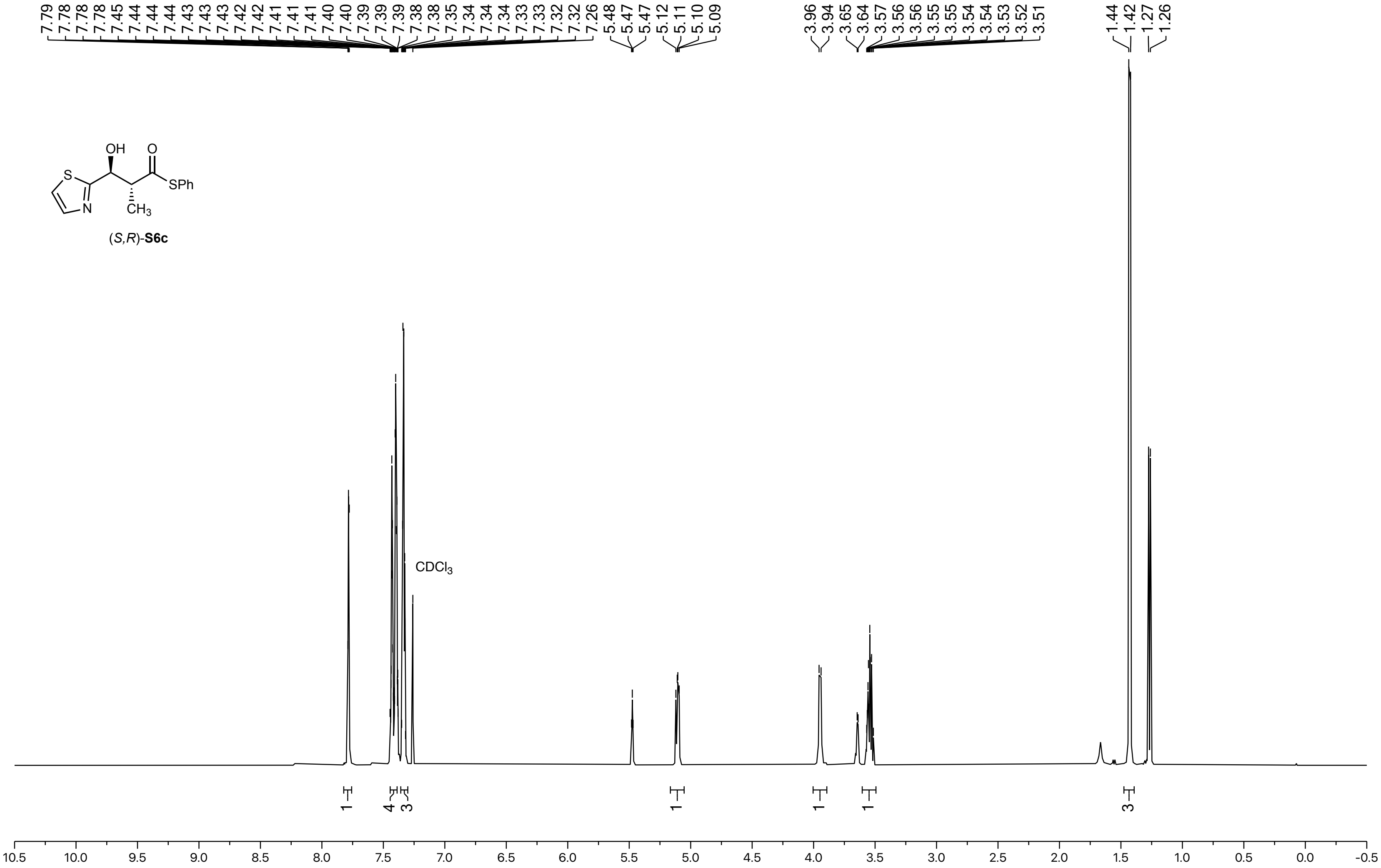

$^{13}C\{^{1}H\}$ NMR, 126 MHz, $CDCl_3$, **S5c**

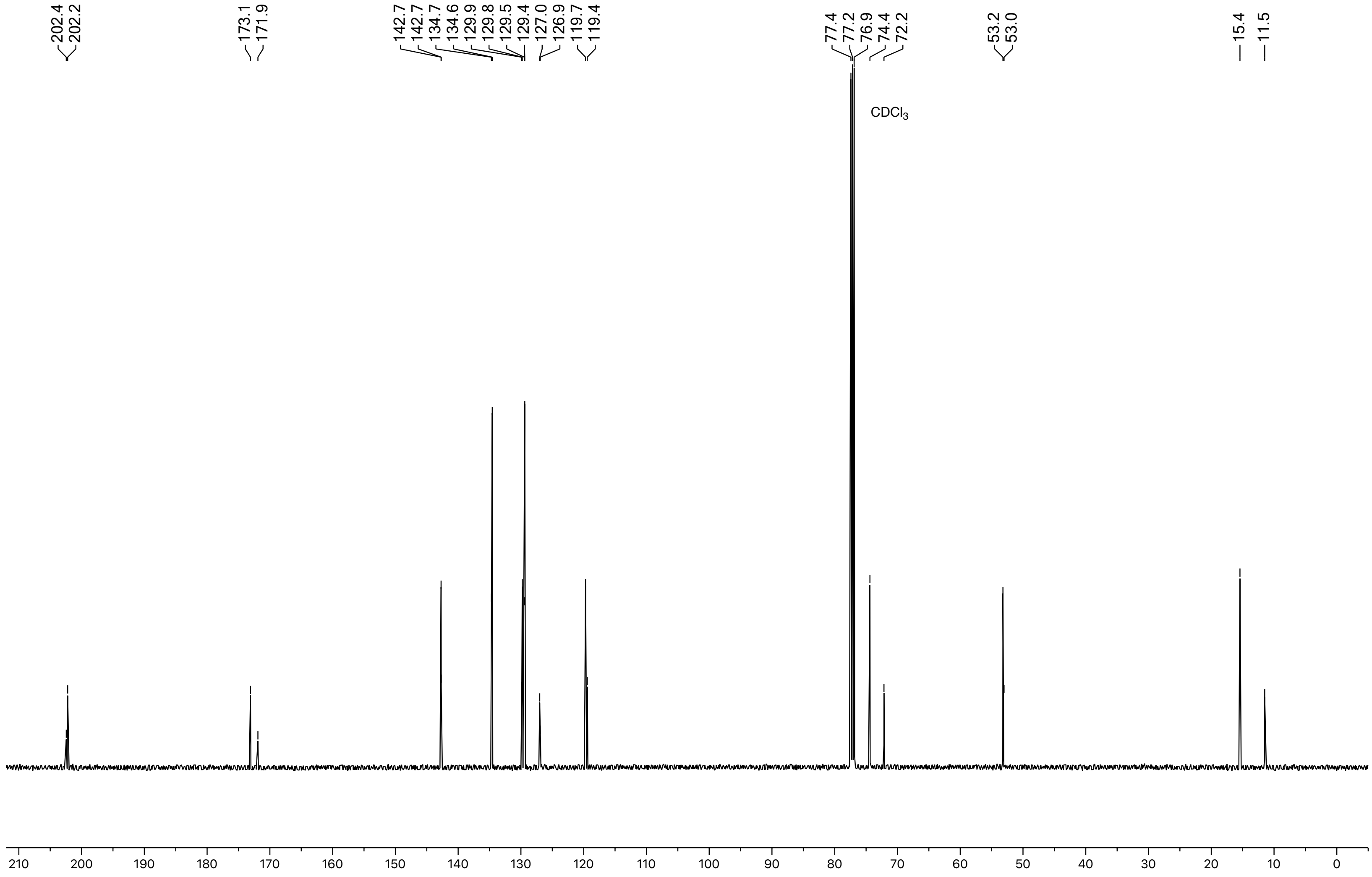

^1H NMR, 500 MHz, $CDCl_3$, **S5d**

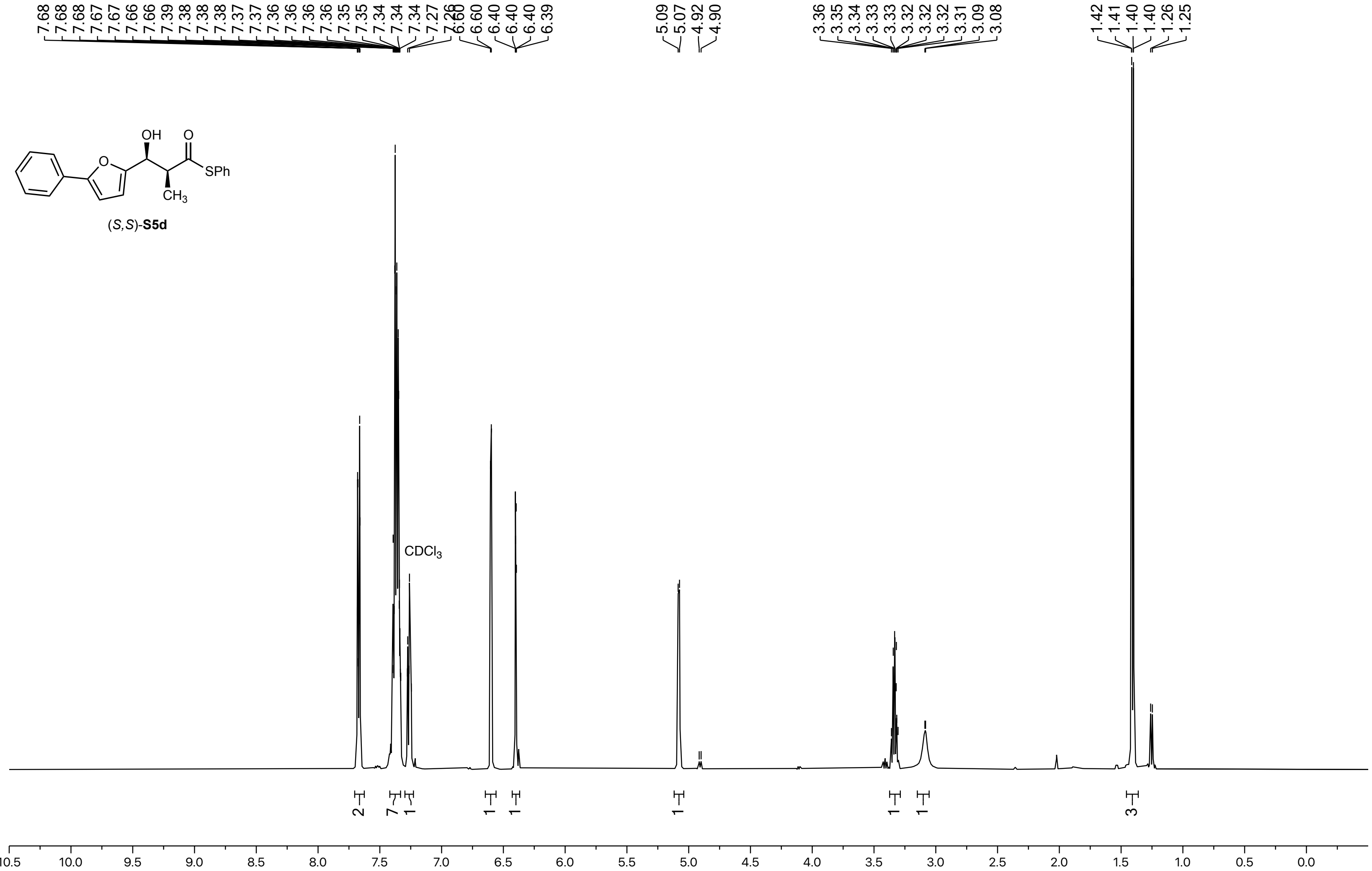

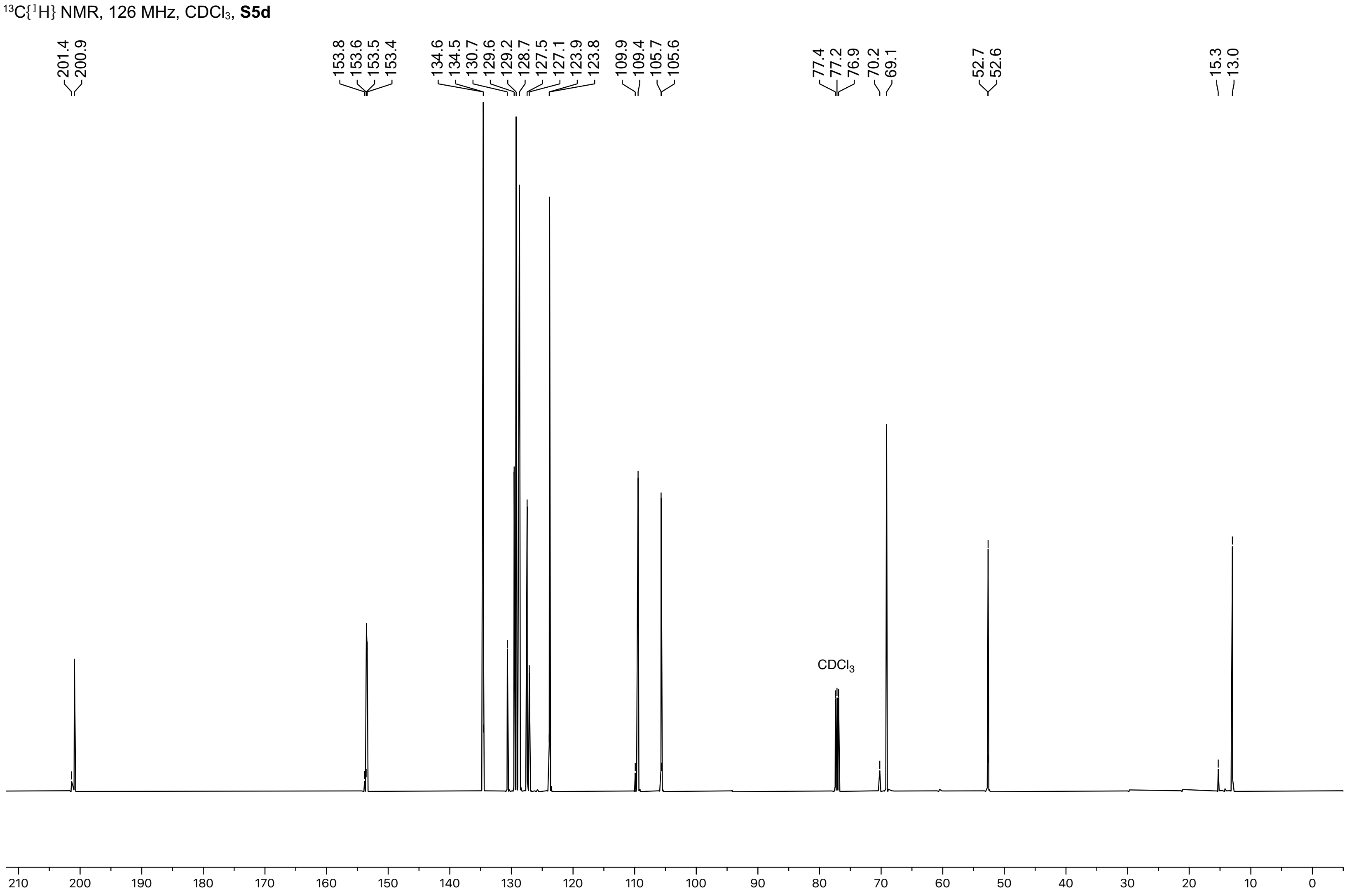
13C{1H} NMR, 126 MHz, CDCl3, S5d
201.4
200.9
153.8
153.6
153.5
153.4
134.6
134.5
130.7
129.6
129.2
128.7
127.5
127.1
123.9
123.8
109.9
109.4
105.7
105.6
77.4
77.2
76.9
70.2
69.1
52.7
52.6
15.3
13.0
CDCl3
210 200 190 180 170 160 150 140 130 120 110 100 90 80 70 60 50 40 30 20 10 0

^{1}H NMR, 500 MHz, $CDCl_3$, **S6d**

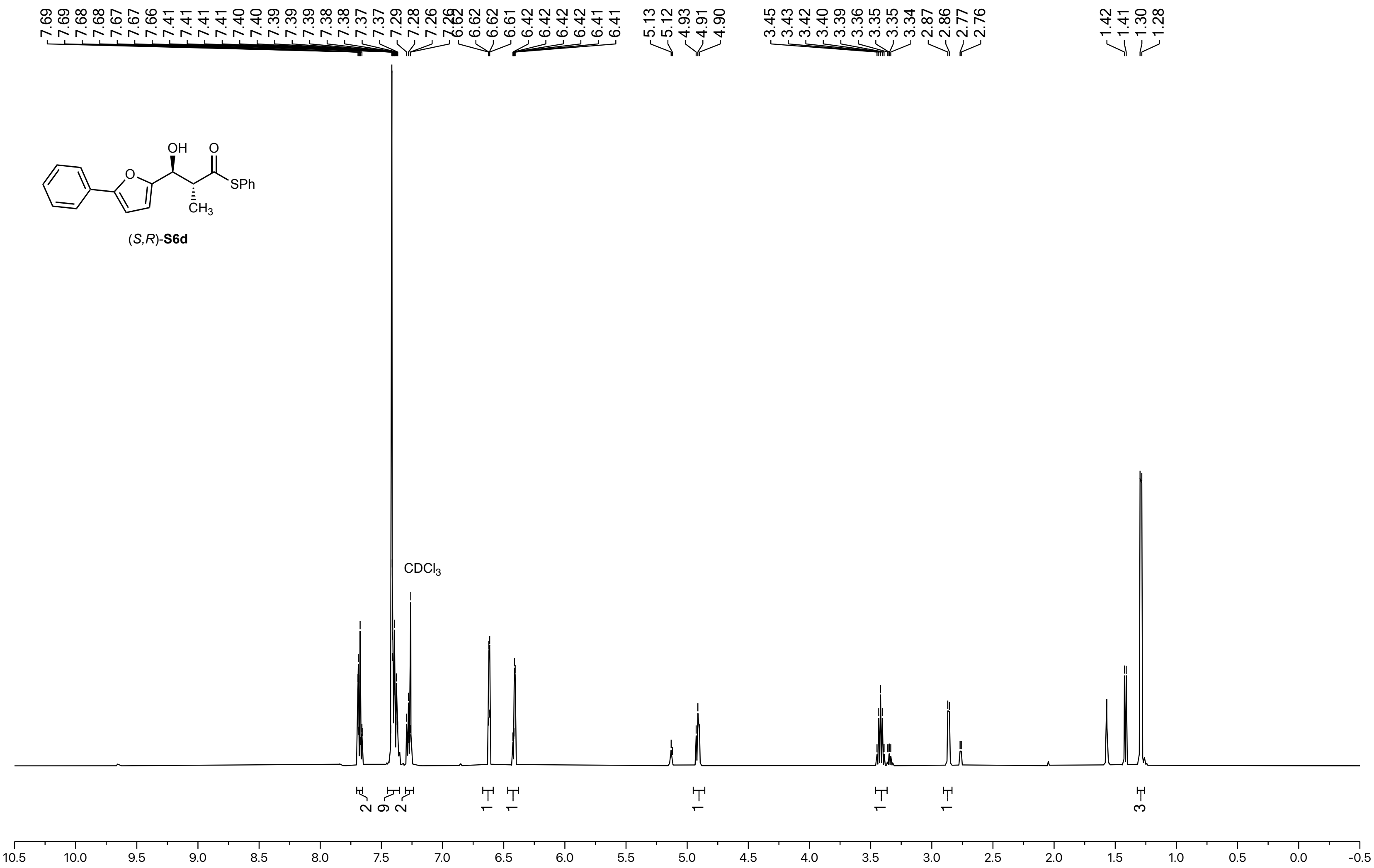

$^{13}C\{^{1}H\}$ NMR, 126 MHz, $CDCl_3$, **S6d**

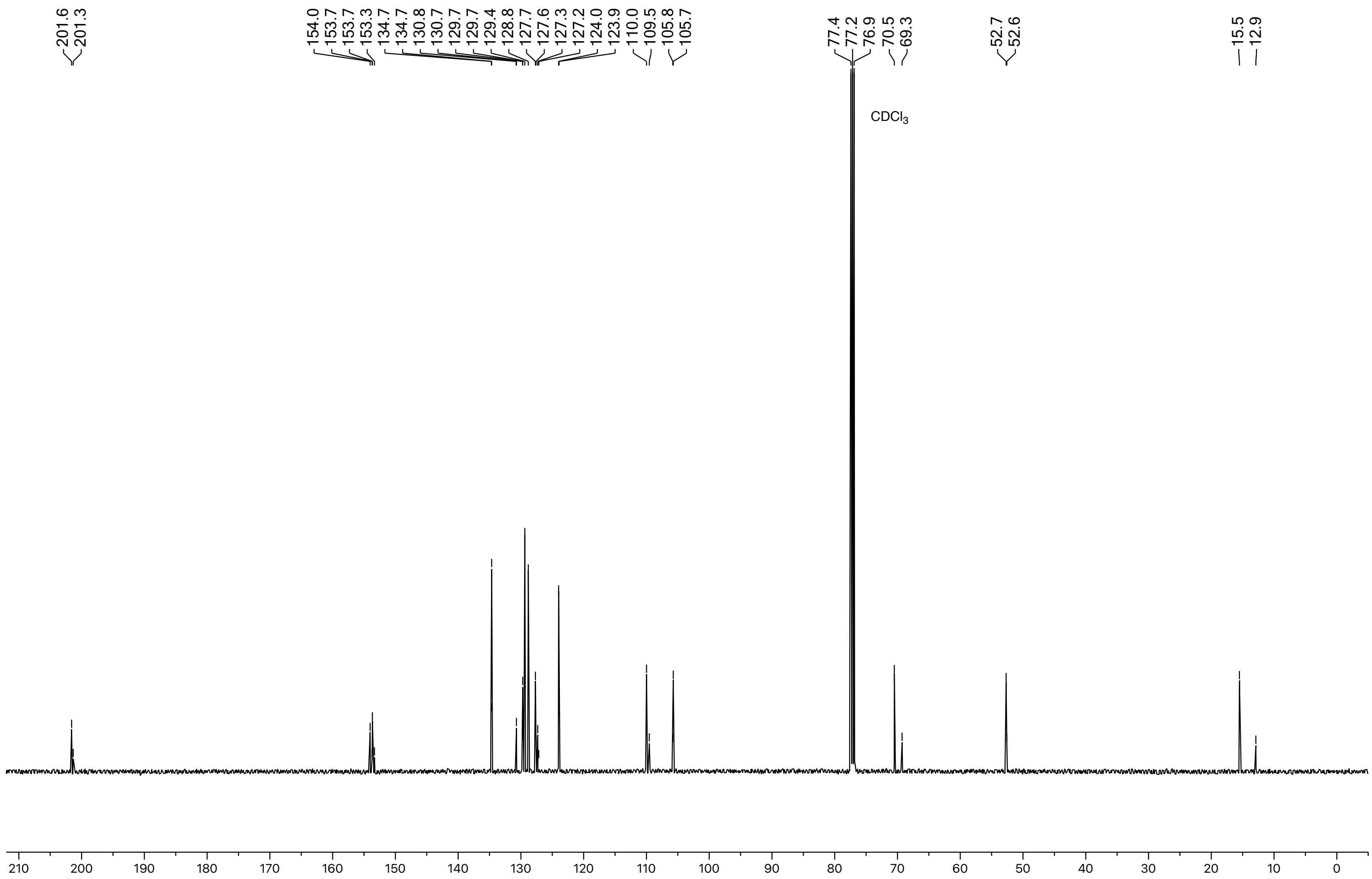

^{1}H NMR, 500 MHz, $CDCl_3$, **S5e**

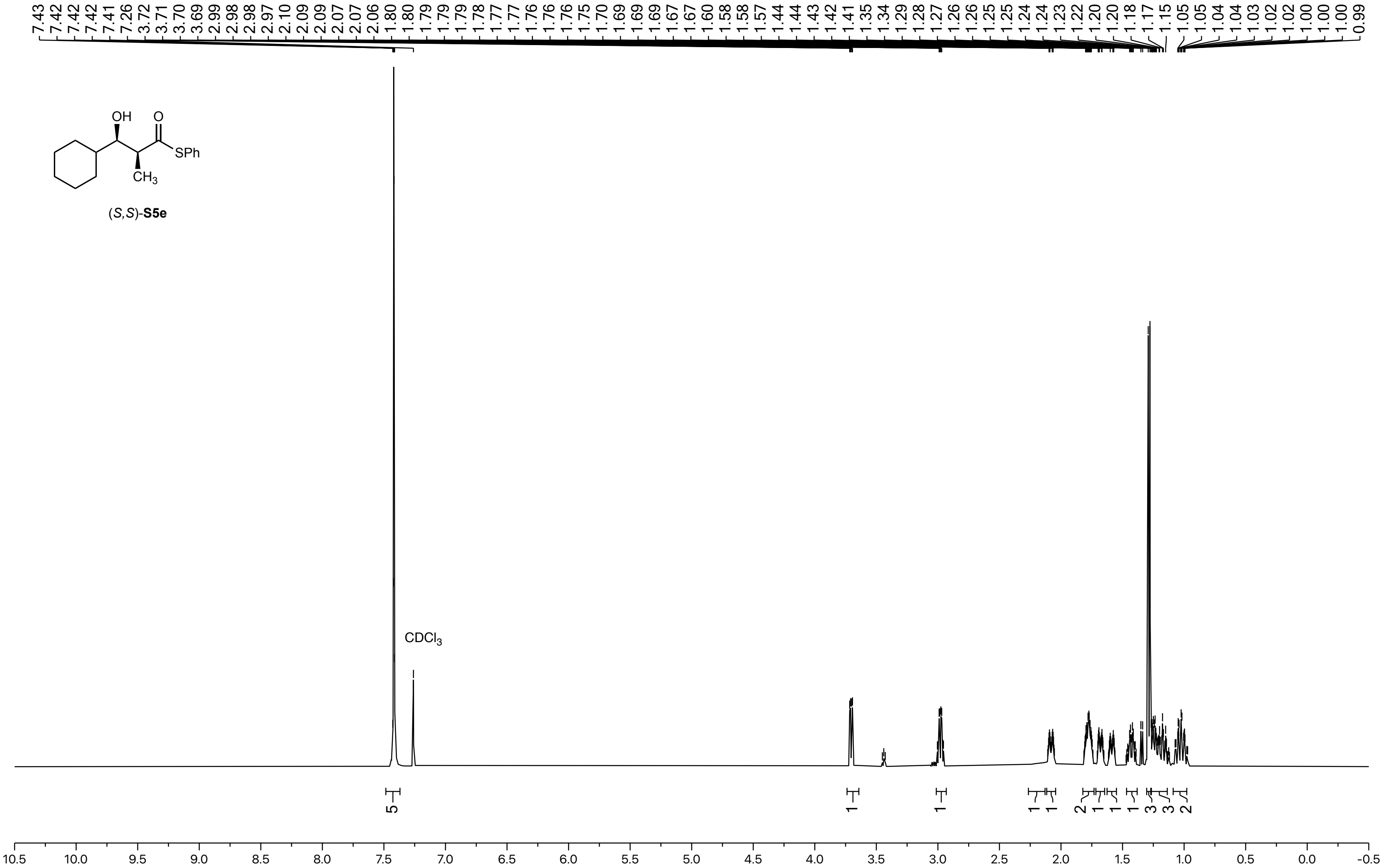

$^{13}C\{^{1}H\}$ NMR, 126 MHz, $CDCl_3$, **S5e**

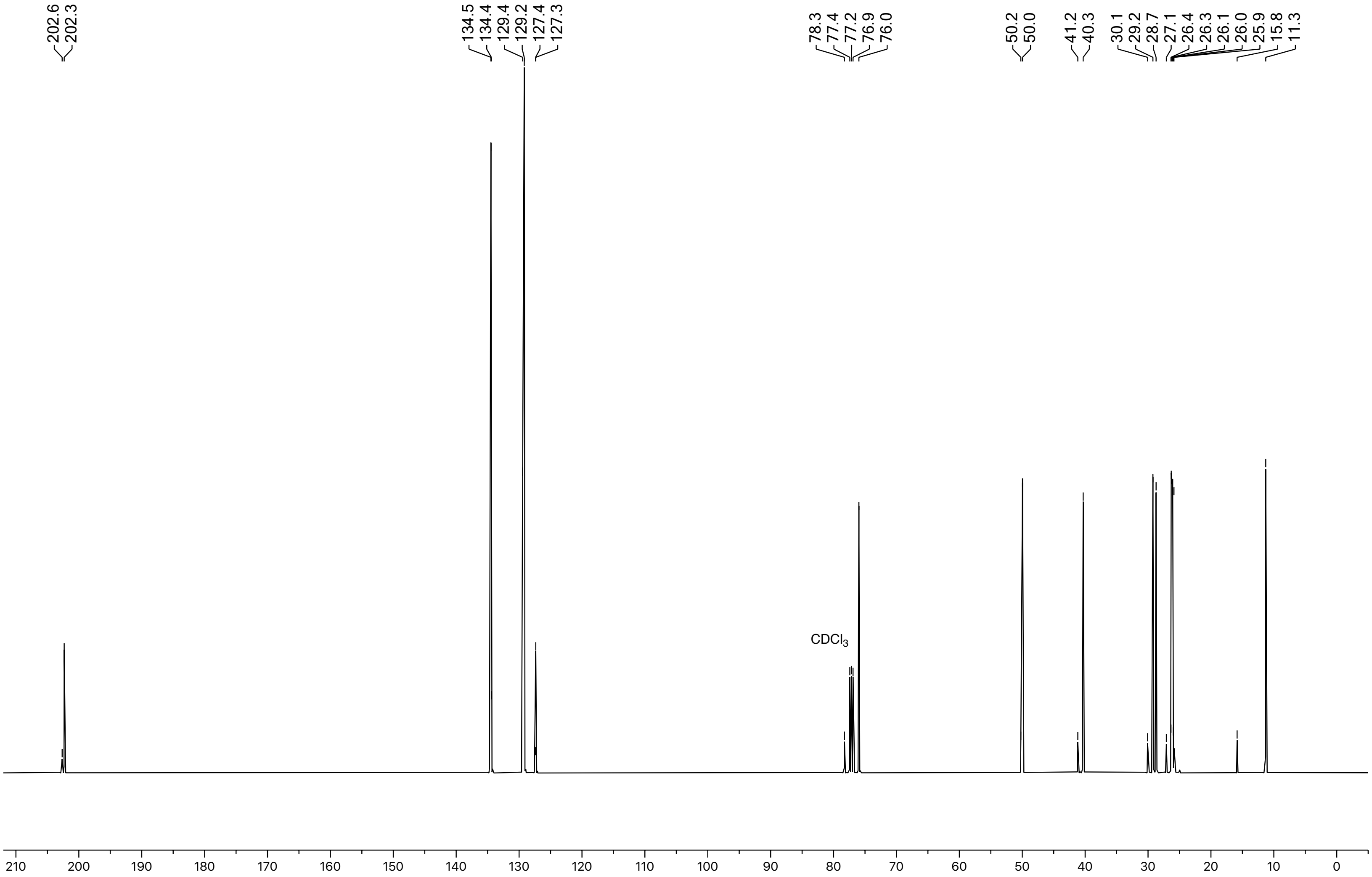

^{1}H NMR, 500 MHz, $CDCl_3$, **S6e**

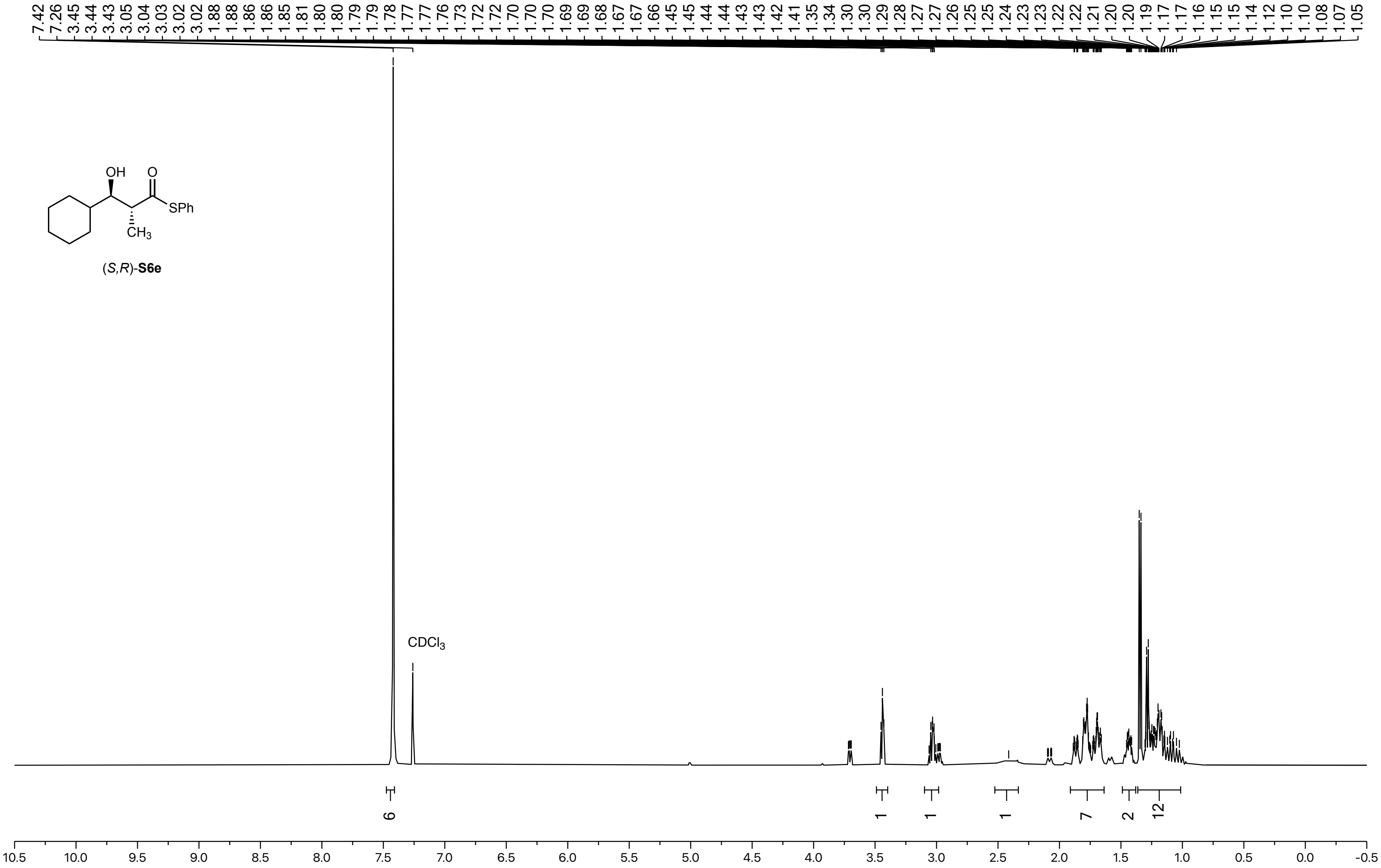

(*S*,*R*)-**S6e**

$^{13}C\{^{1}H\}$ NMR, 126 MHz, $CDCl_3$, **S6e**

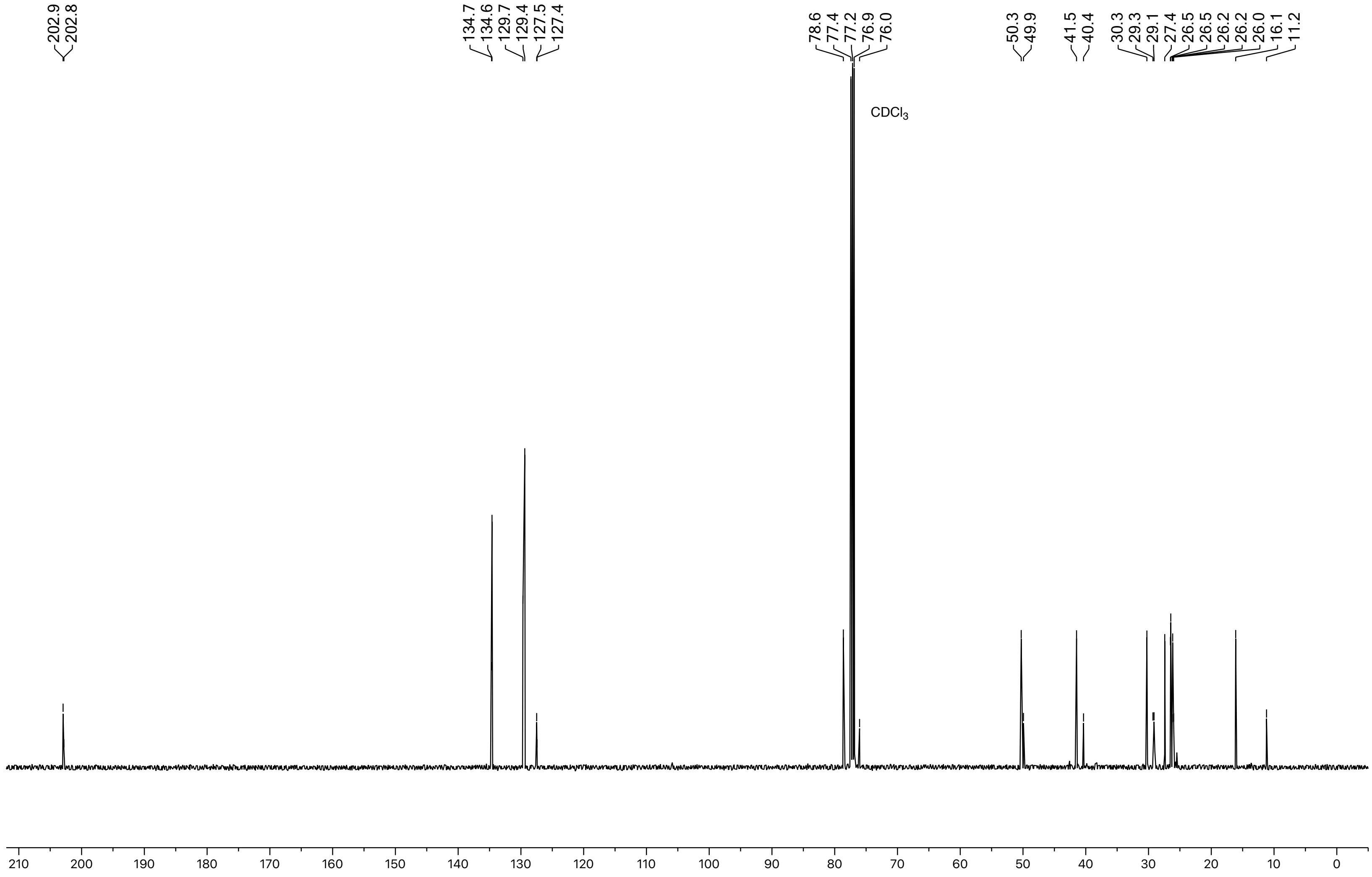

^{1}H NMR, 500 MHz, $CDCl_3$, **S5f**

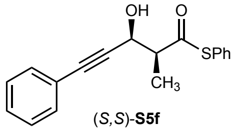

(*S*,*S*)-**S5f**

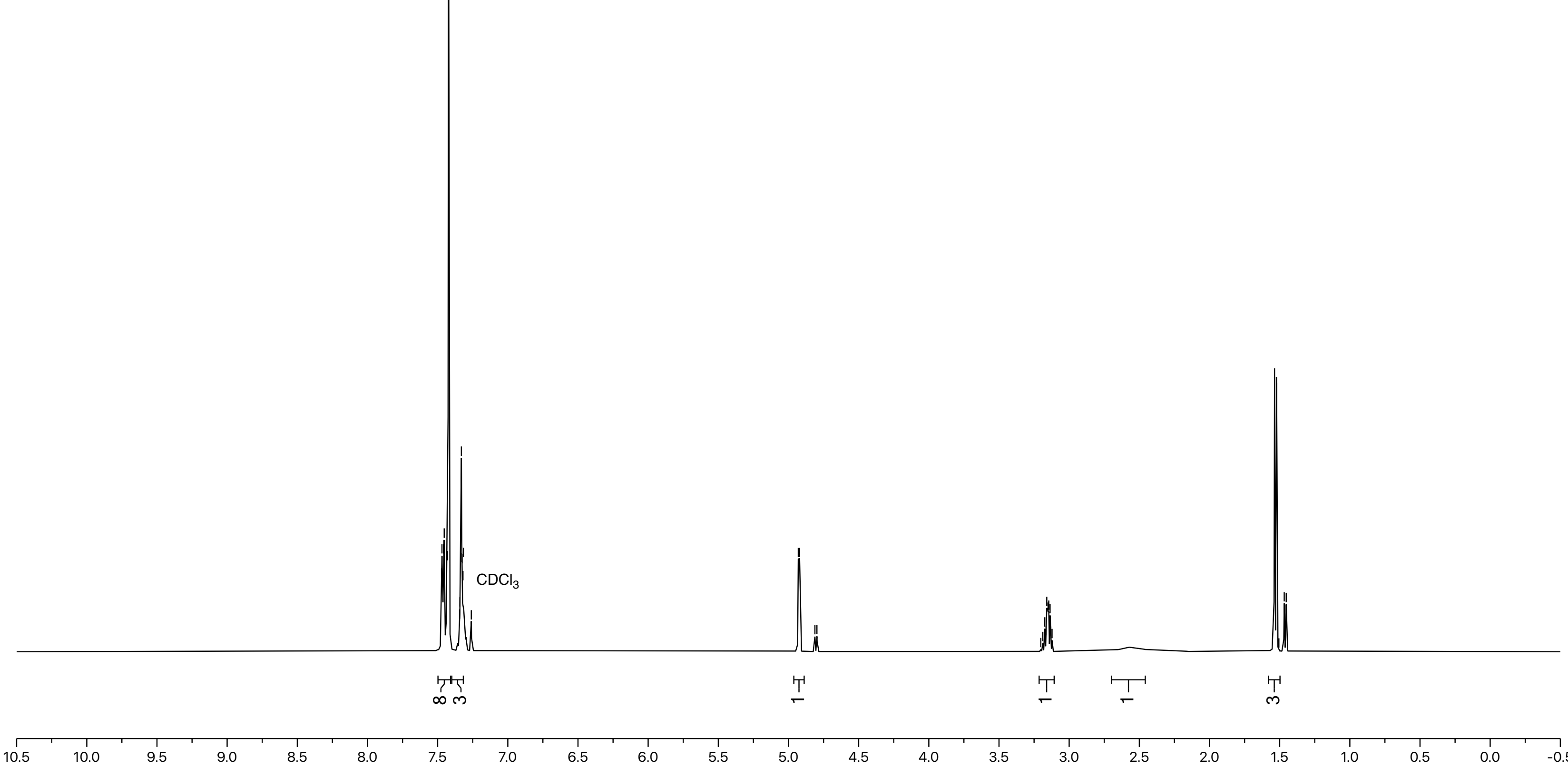

$^{13}C\{^{1}H\}$ NMR, 126 MHz, $CDCl_3$, **S5f**

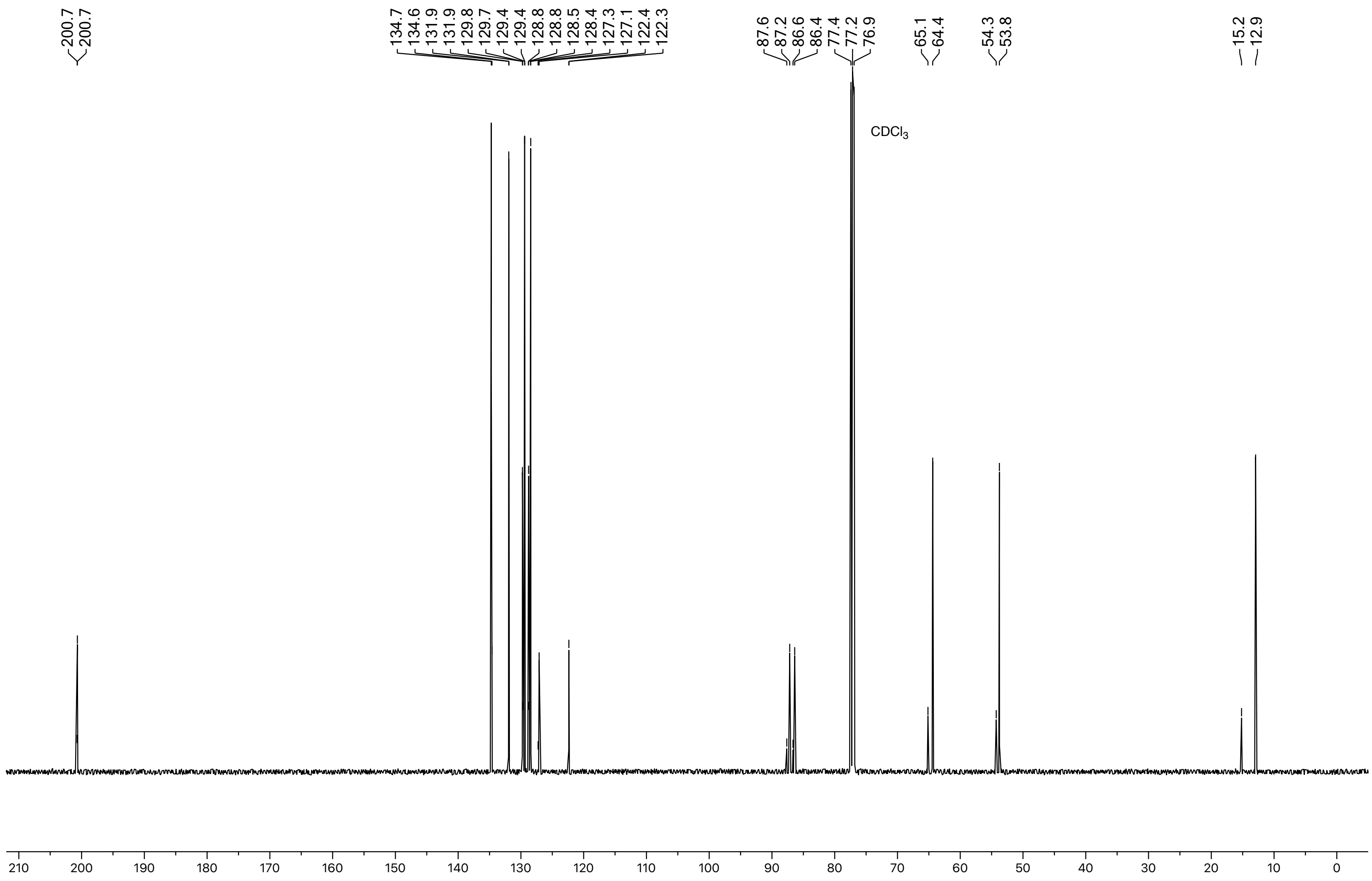

^{1}H NMR, 500 MHz, $CDCl_3$, **S6f**

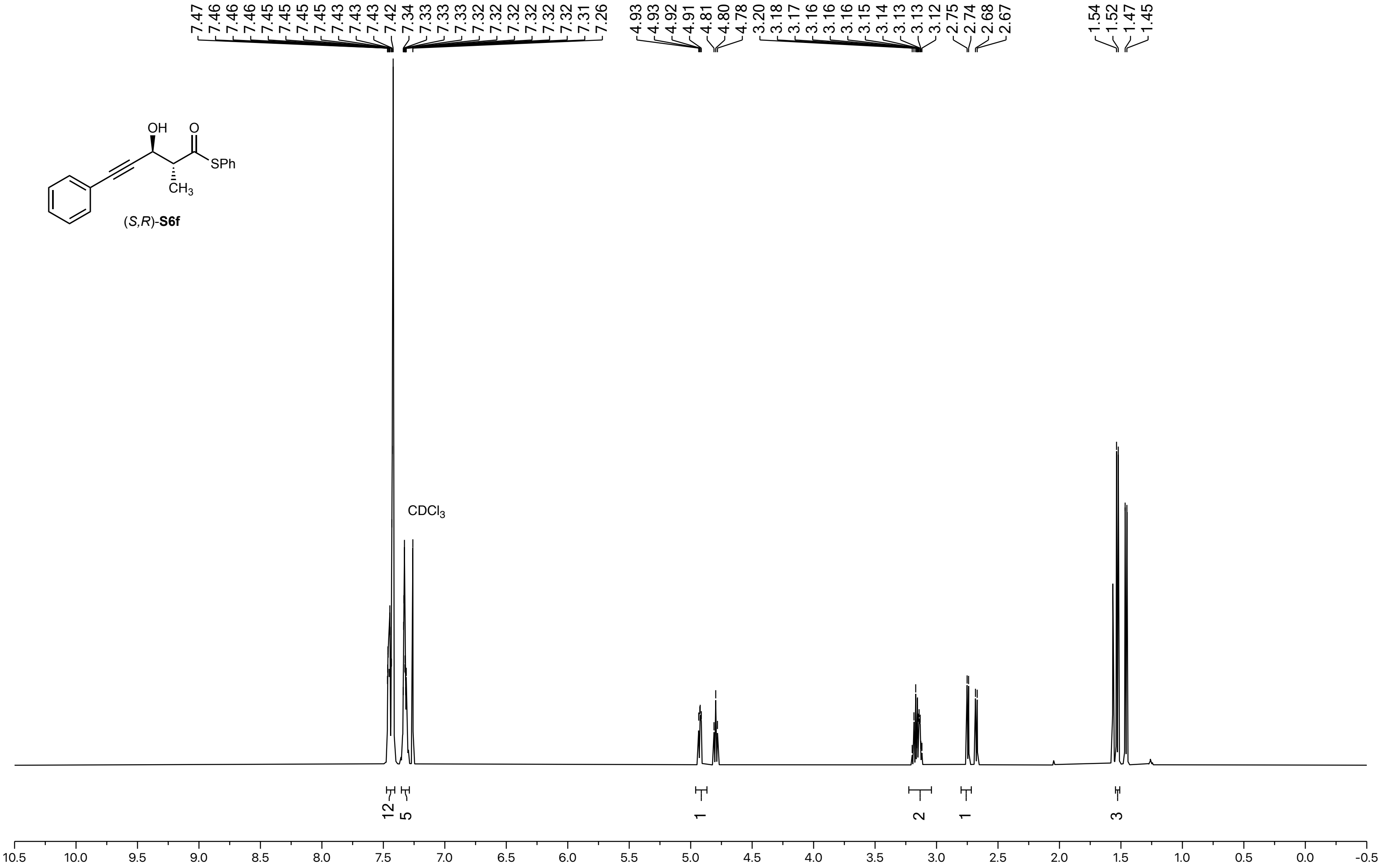

$^{13}C\{^{1}H\}$ NMR, 126 MHz, $CDCl_3$, **S6f**

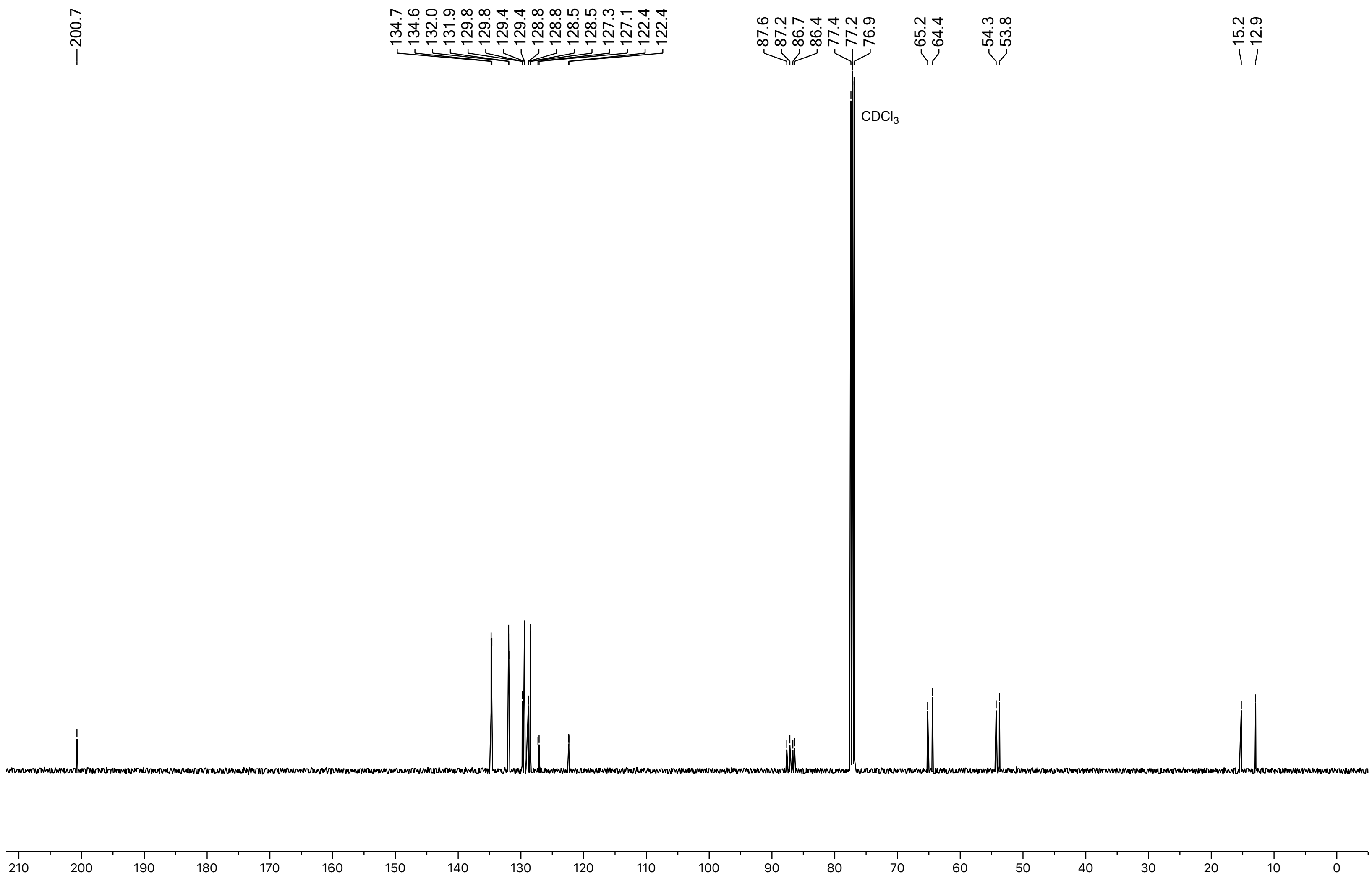

^{1}H NMR, 500 MHz, $CDCl_3$, **S5g**

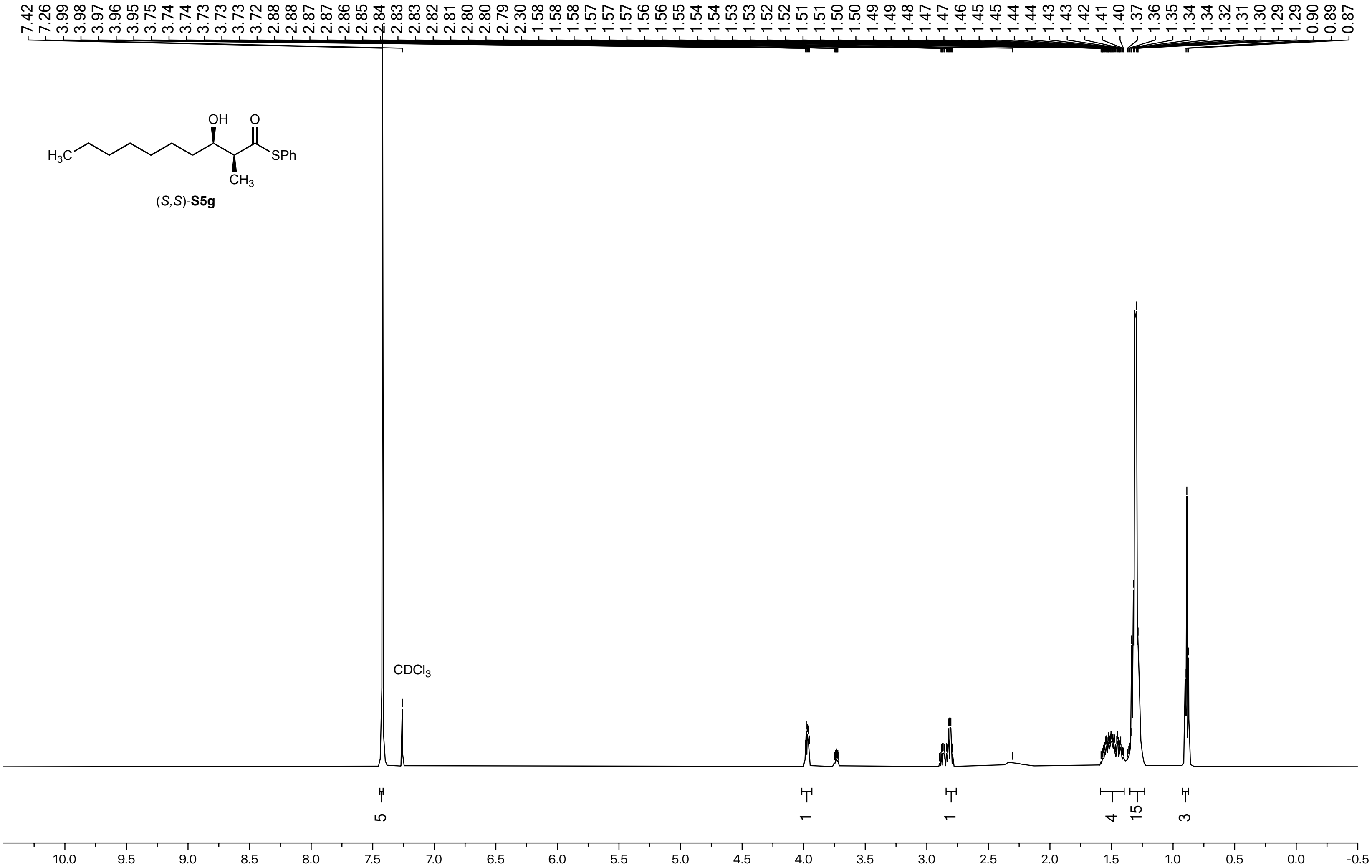

$^{13}C\{^{1}H\}$ NMR, 126 MHz, $CDCl_3$, **S6g**

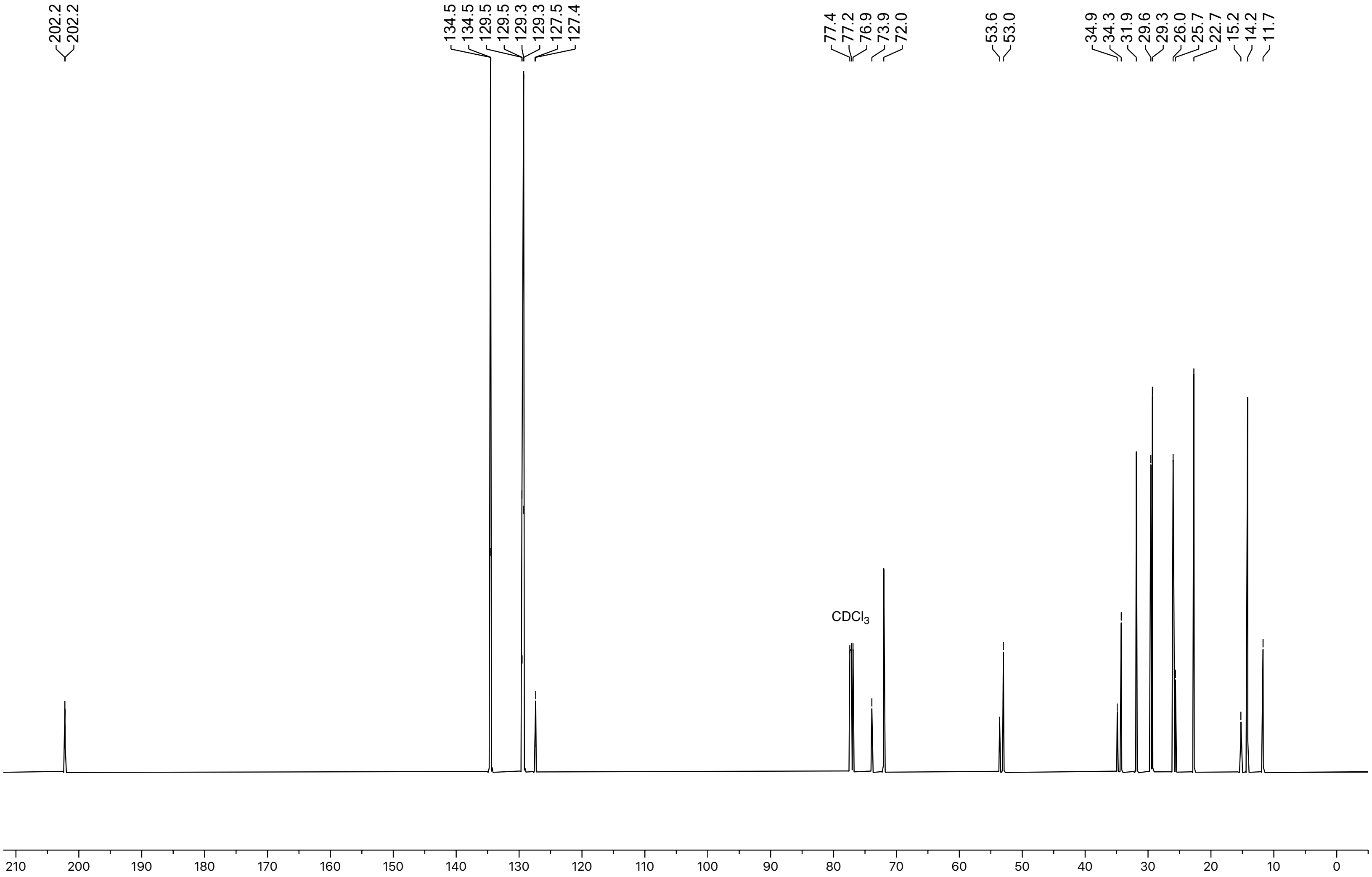

^1H NMR, 500 MHz, $CDCl_3$, **S6g**

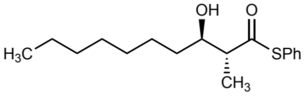

(*S*,*R*)-**S6g**

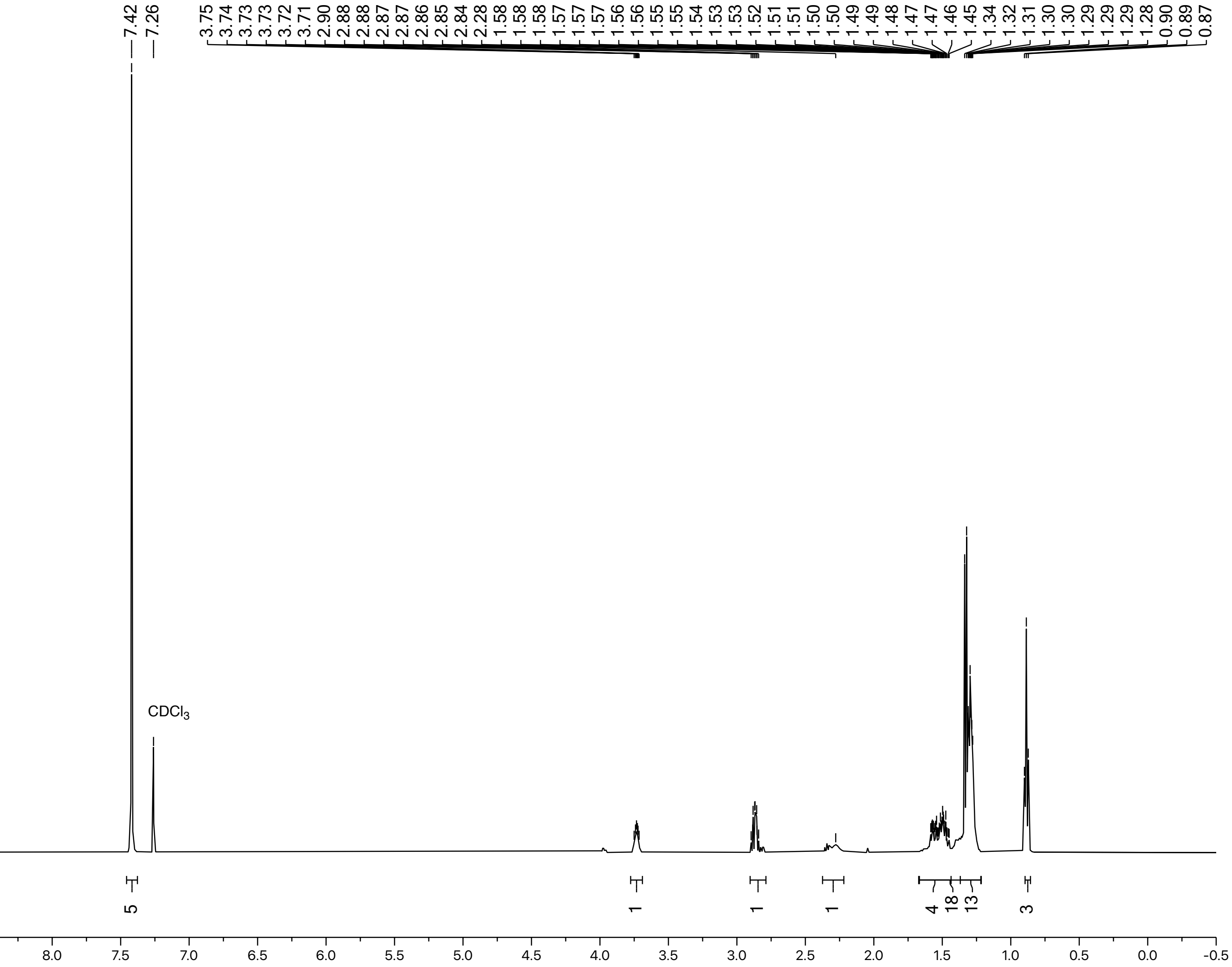

$^{13}C\{^1H\}$ NMR, 126 MHz, $CDCl_3$, **S6g**

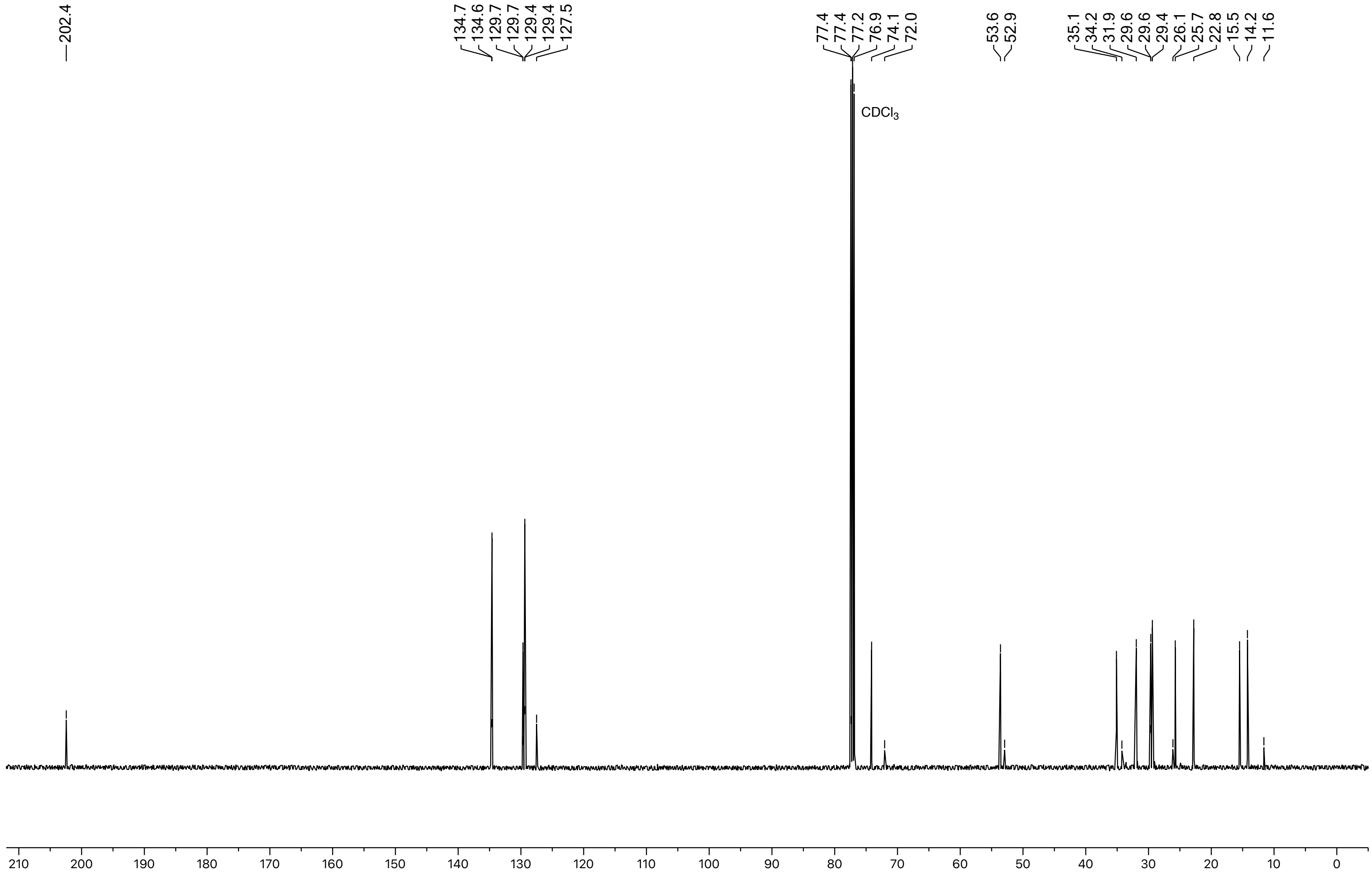

^{1}H NMR, 500 MHz, $CDCl_3$, **S5h**

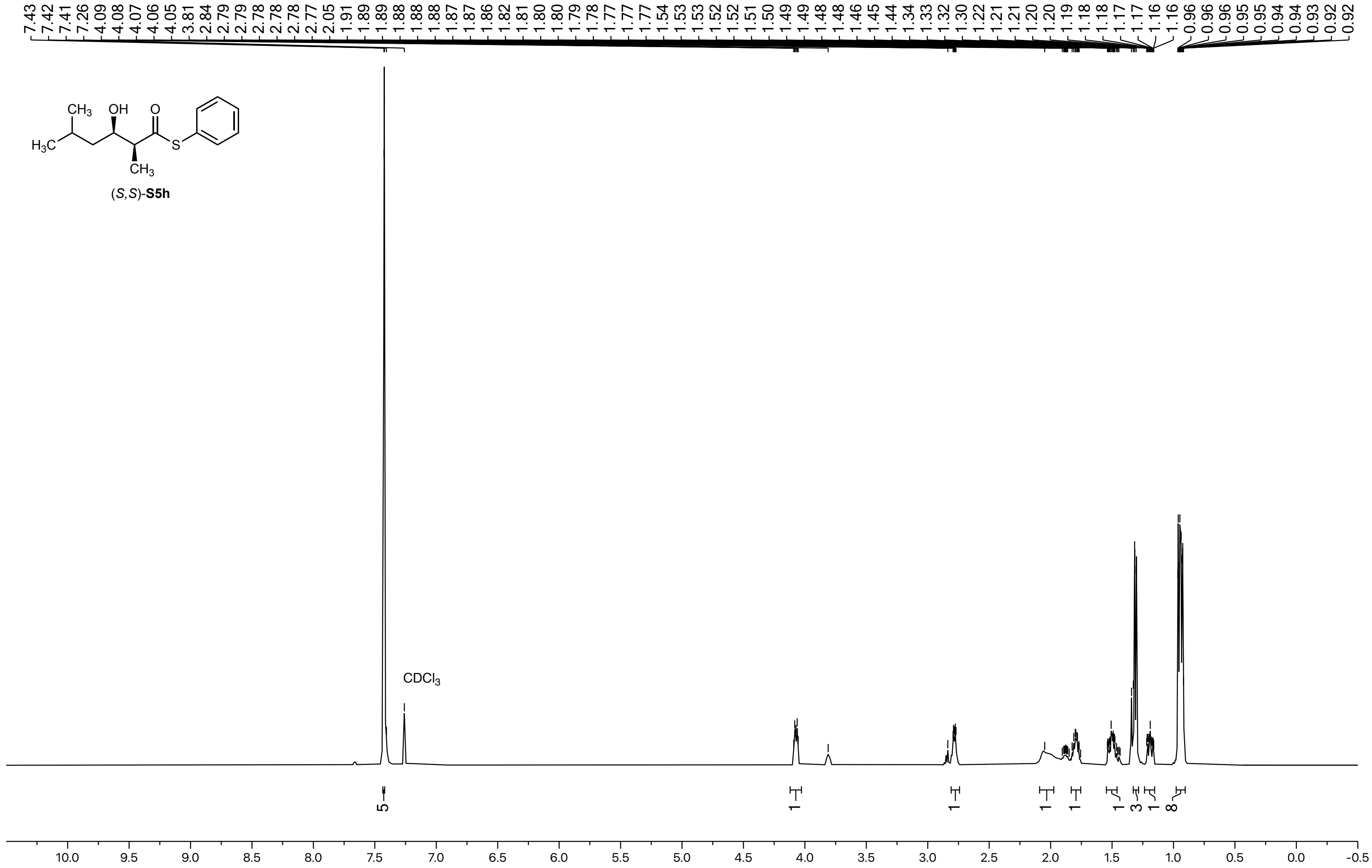

$^{13}C\{^{1}H\}$ NMR, 126 MHz, $CDCl_3$, **S5h**

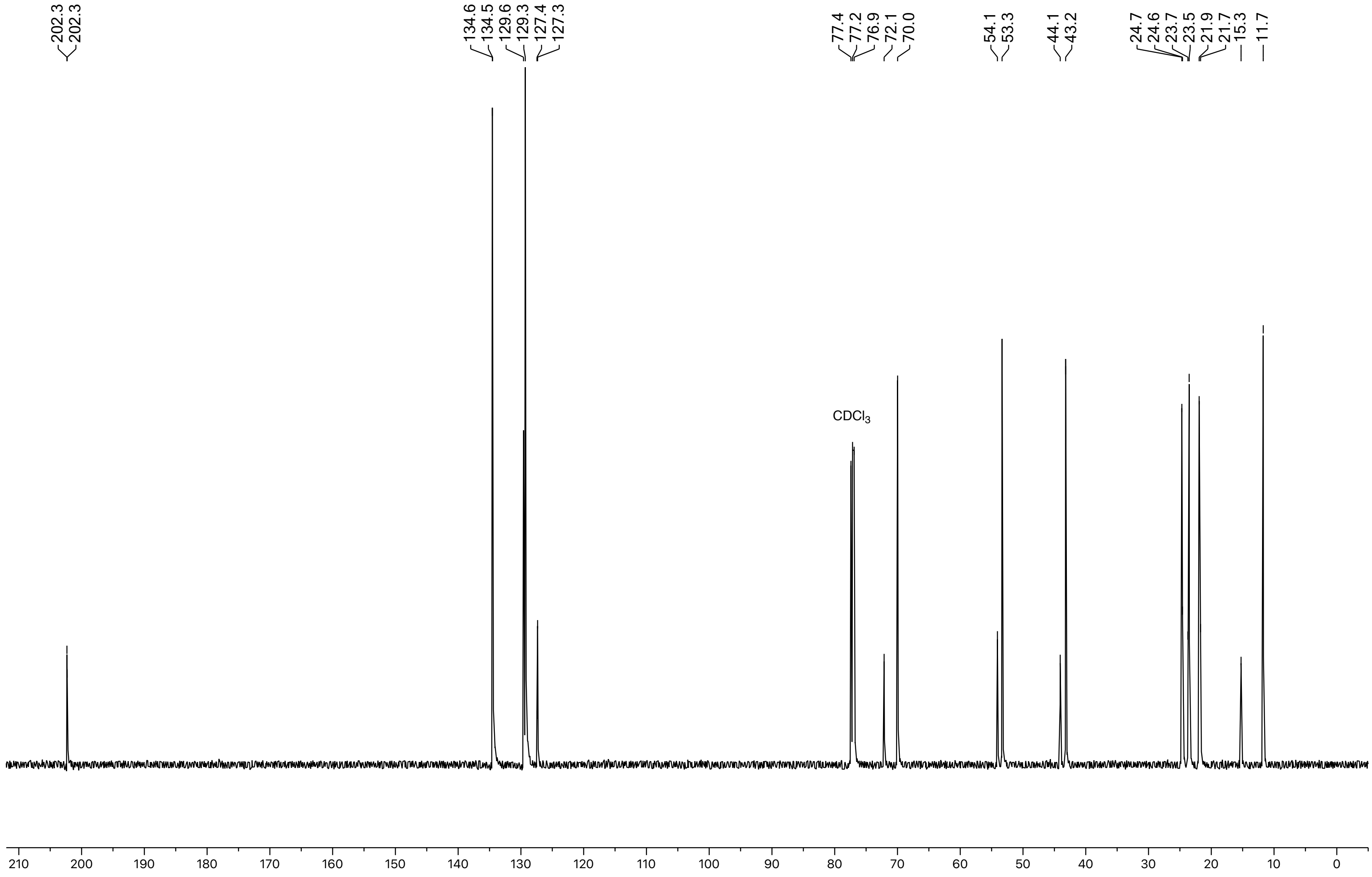

^{1}H NMR, 500 MHz, $CDCl_3$, **S6h**

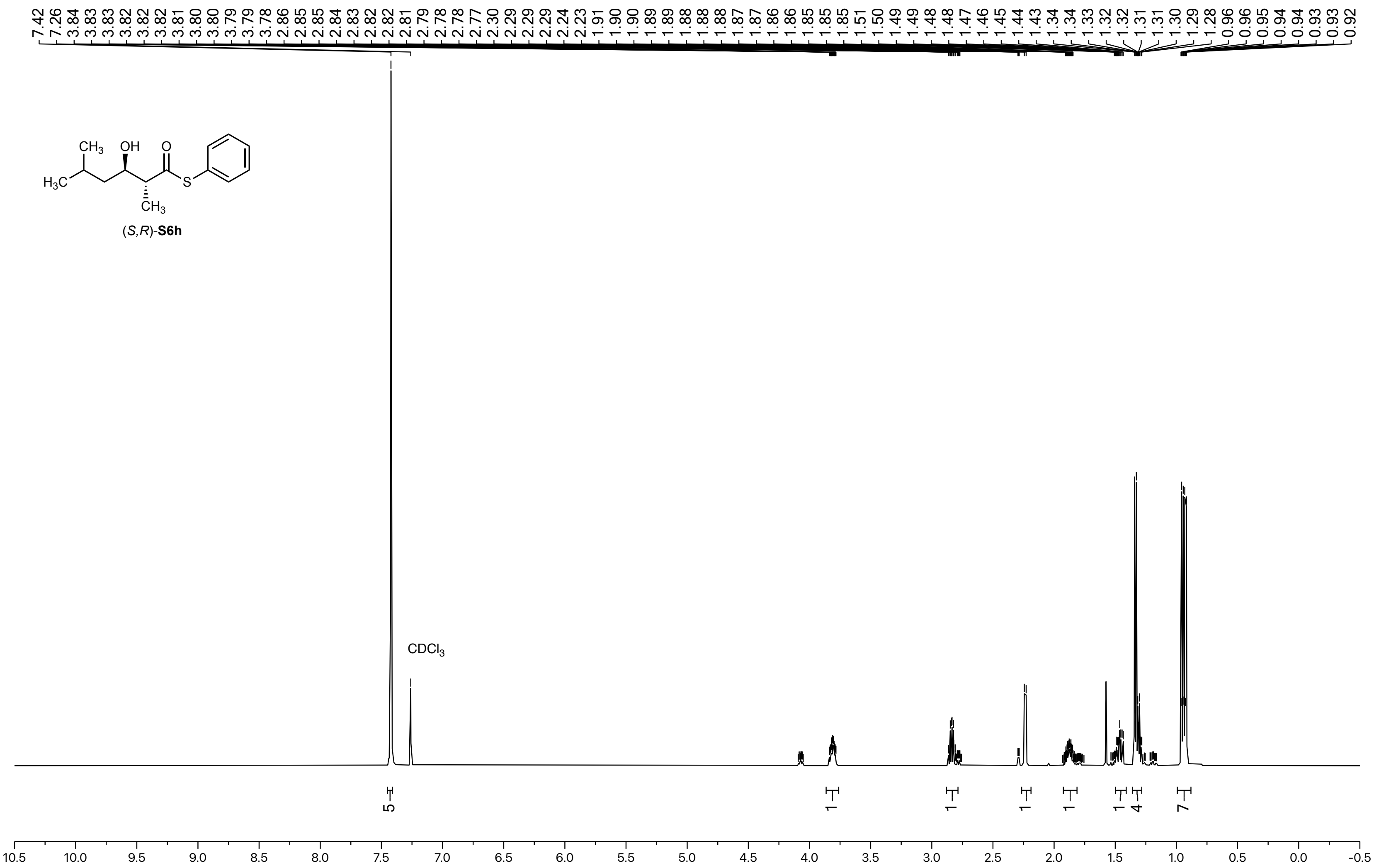

$^{13}C\{^1H\}$ NMR, 126 MHz, $CDCl_3$, **S6h**

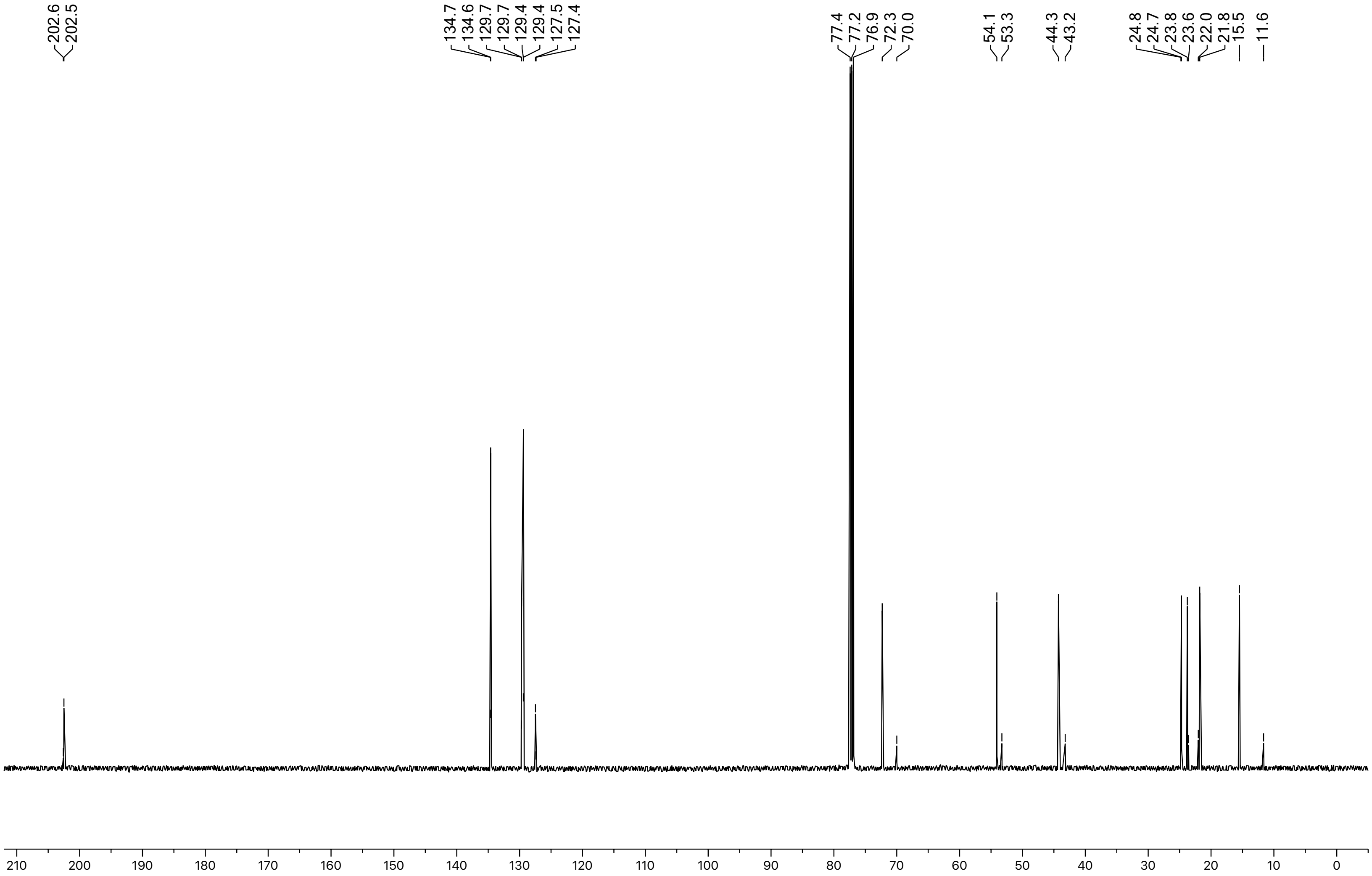

^{1}H NMR, 500 MHz, $CDCl_3$, **S7h**

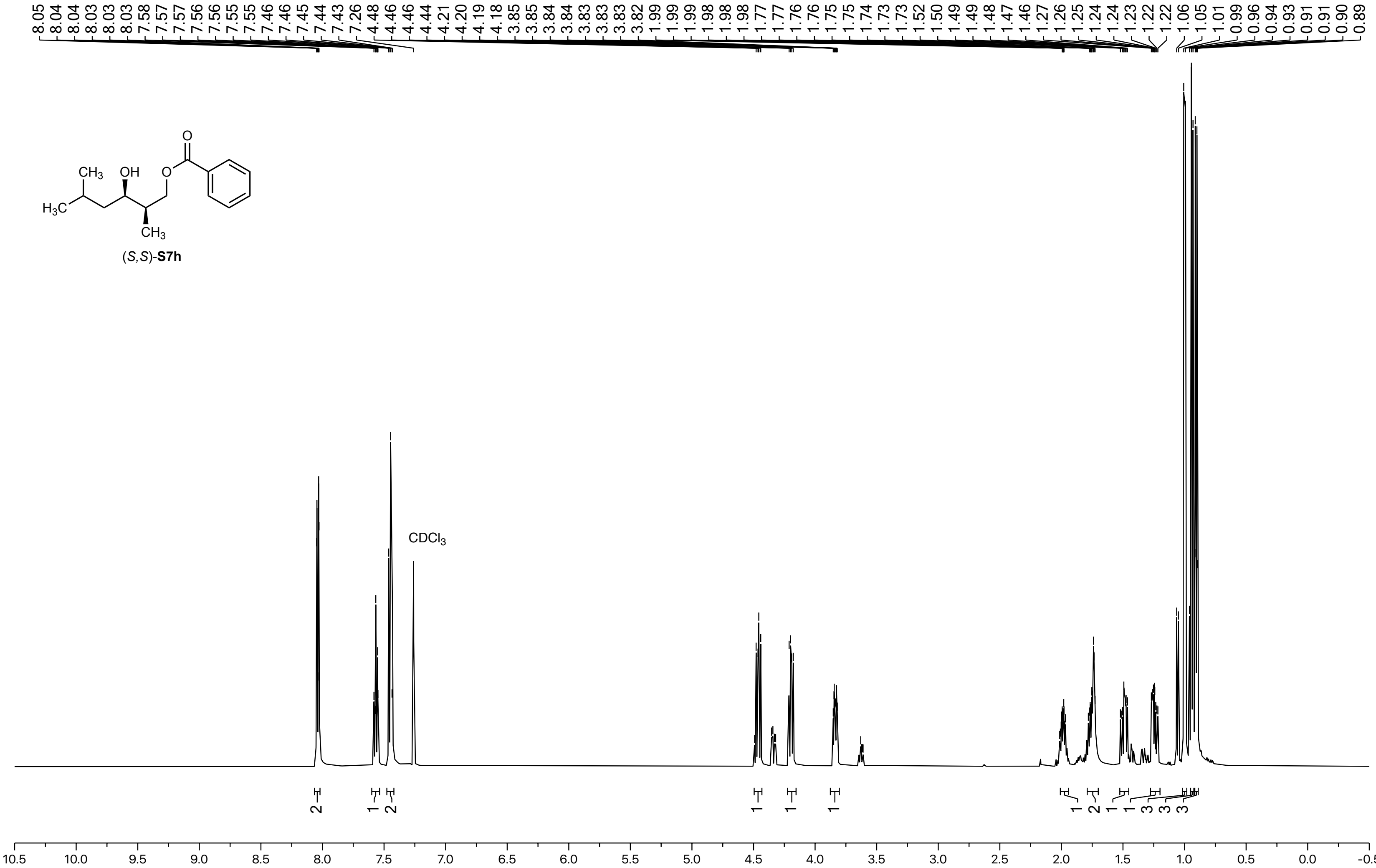

(*S*,*S*)-**S7h**

$^{13}C\{^{1}H\}$ NMR, 126 MHz, $CDCl_3$, **S7h**

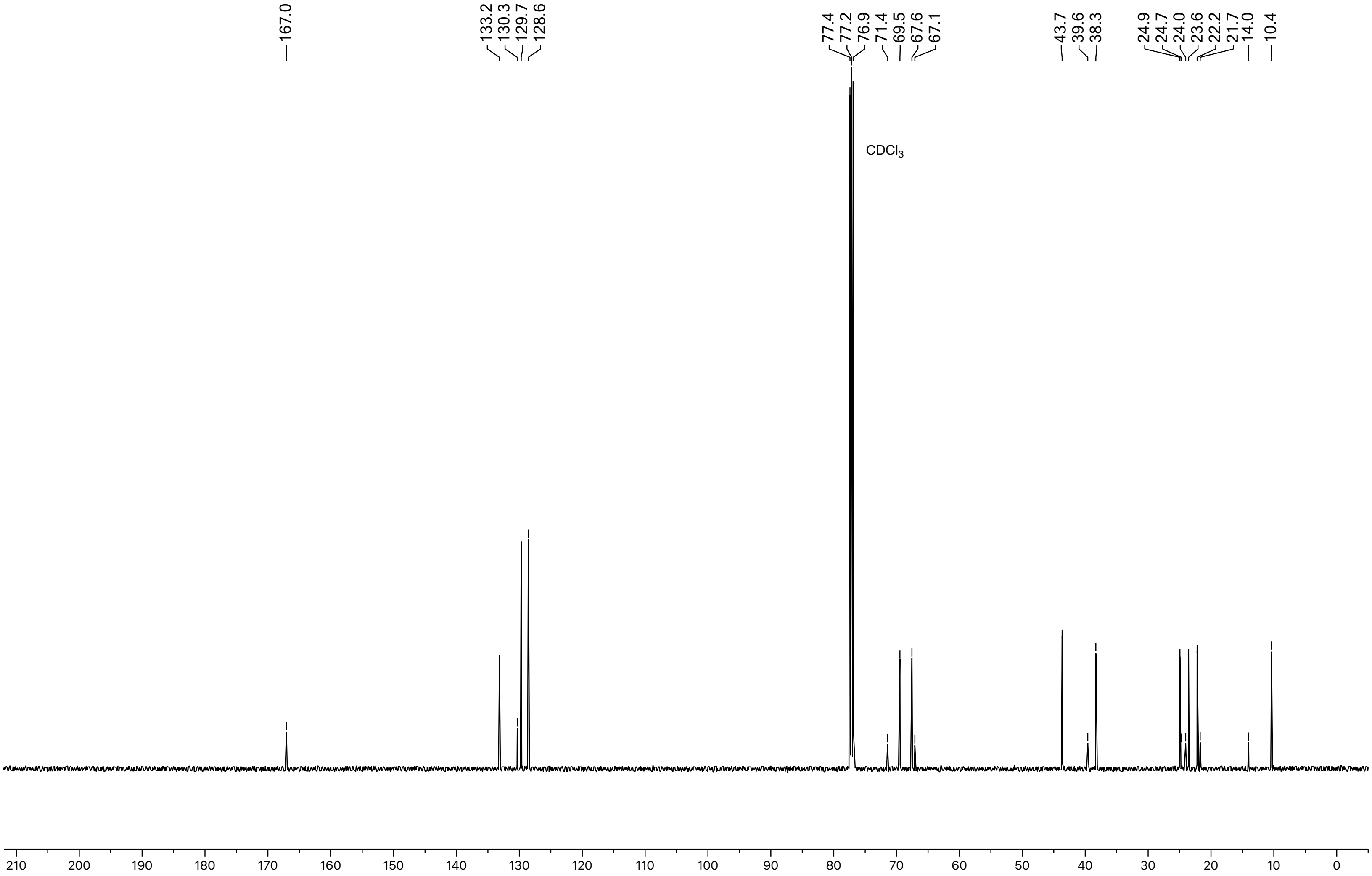

^{1}H NMR, 500 MHz, $CDCl_3$, **S8h**

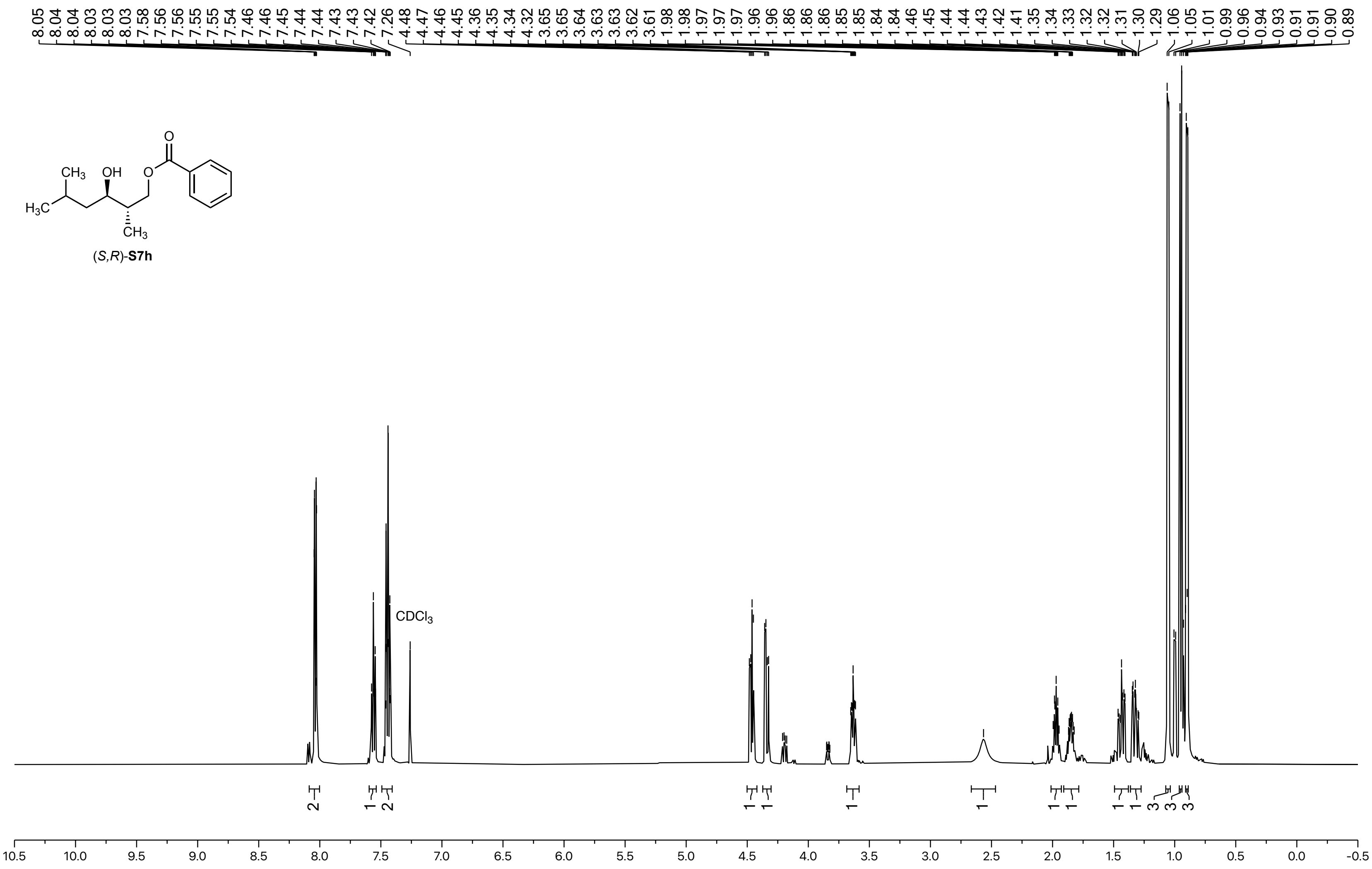

$^{13}C\{^{1}H\}$ NMR, 126 MHz, $CDCl_3$, **S8h**

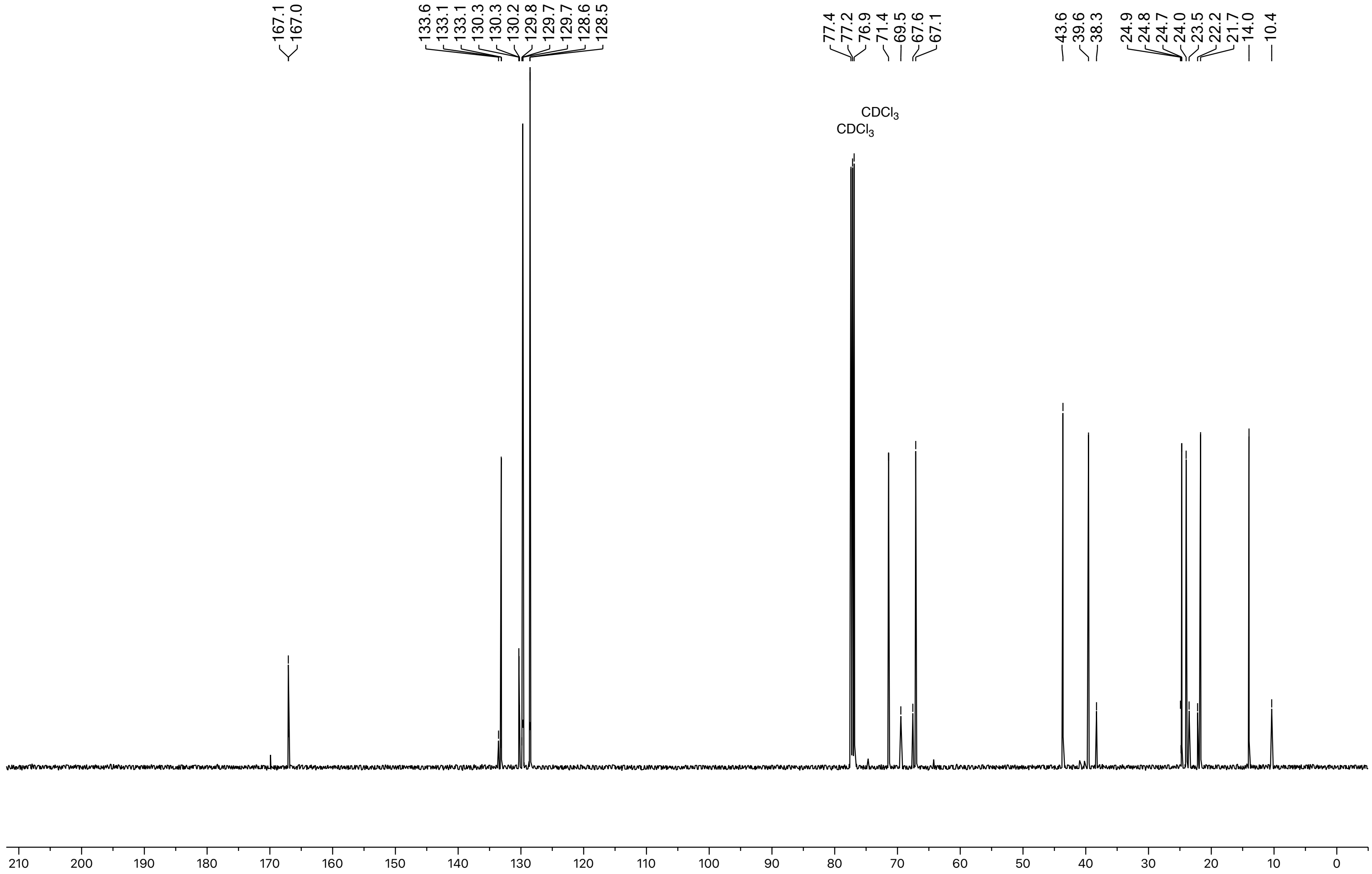

¹H NMR, 500 MHz, $CDCl_3$, **7a**

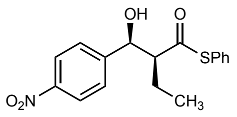

(*S*,*S*)-**7a**

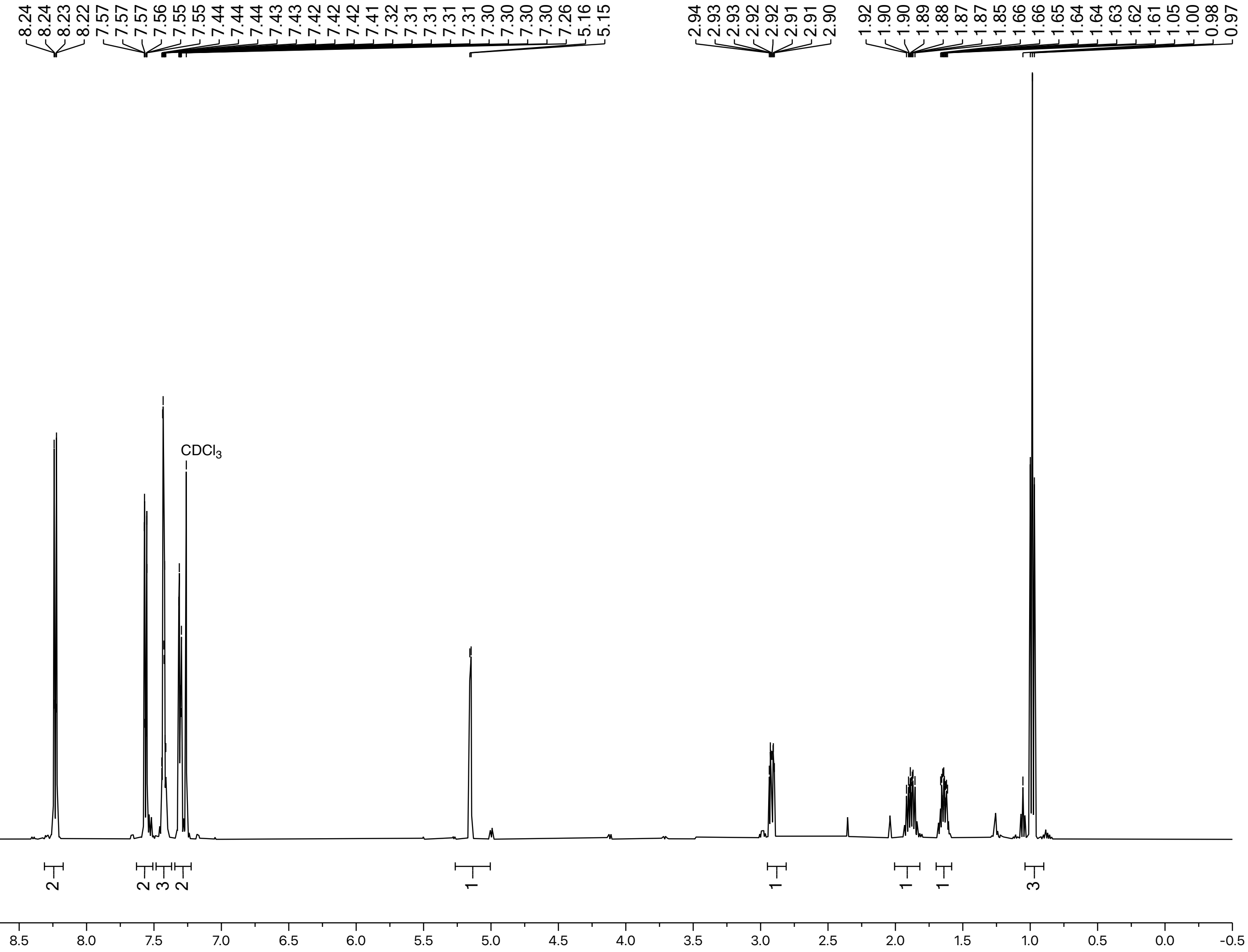

$^{13}C\{^1H\}$ NMR, 126 MHz, $CDCl_3$, **7a**

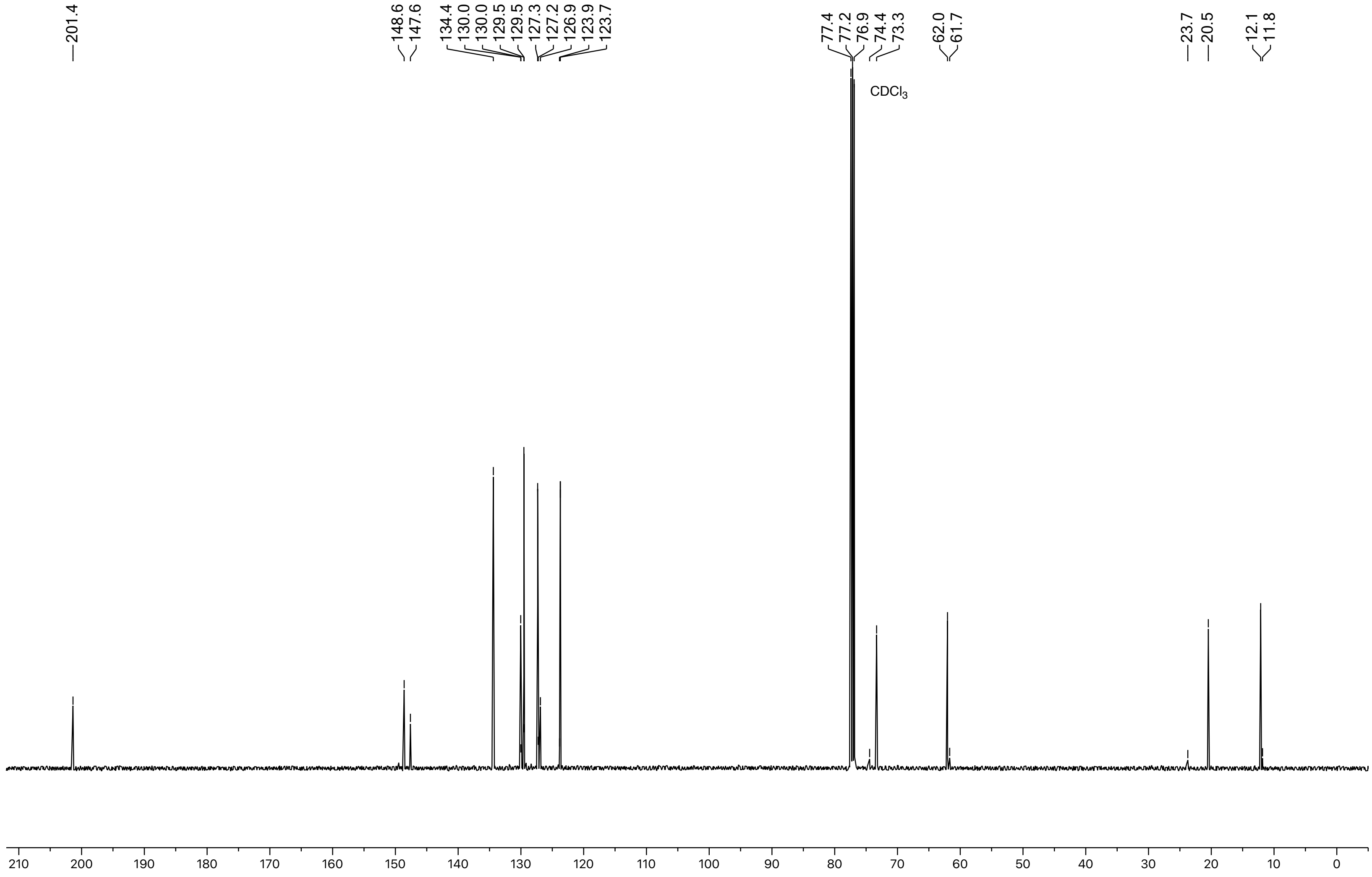

^{1}H NMR, 500 MHz, $CDCl_3$, **7b**

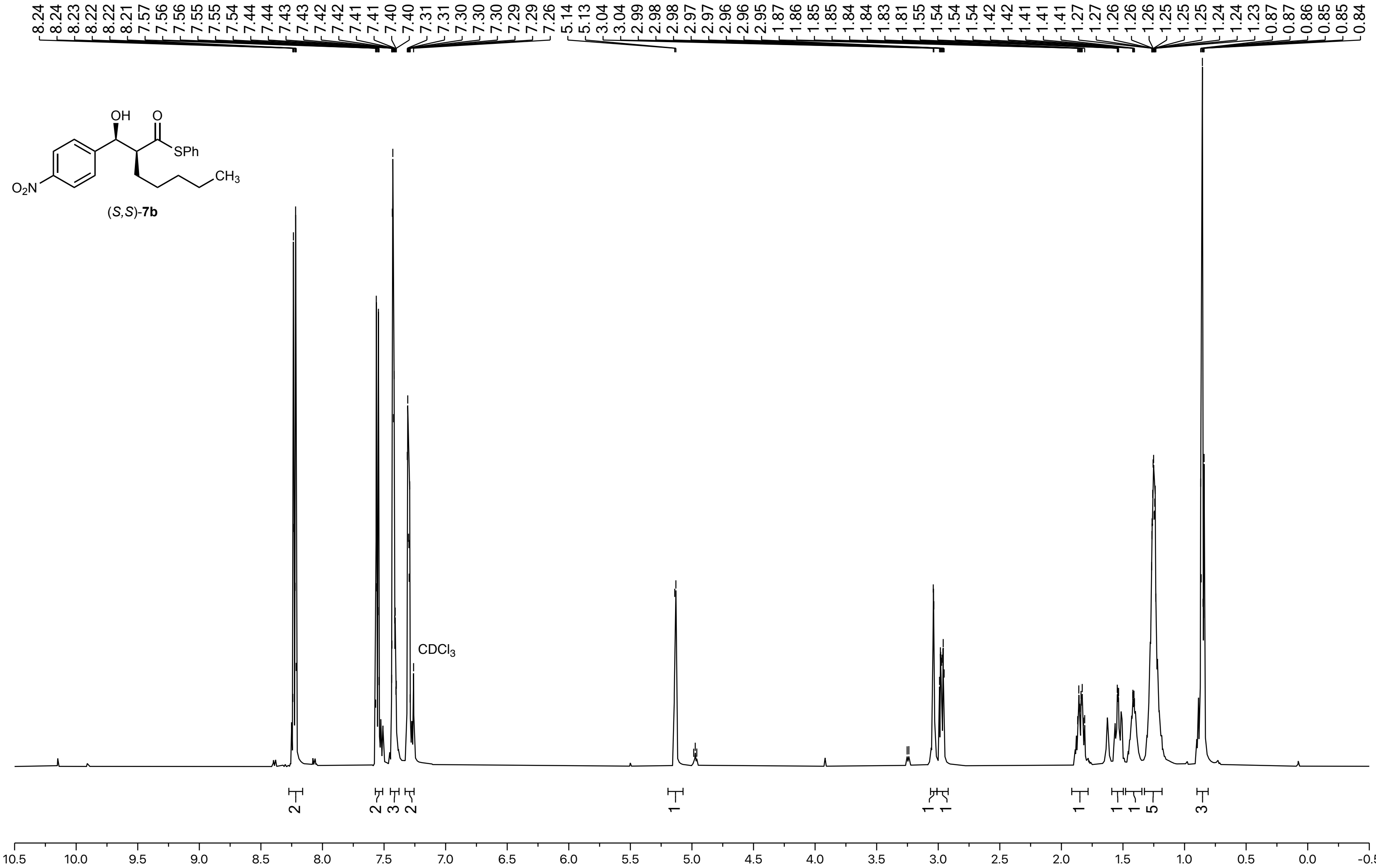

$^{13}C\{^1H\}$ NMR, 126 MHz, $CDCl_3$, **7b**

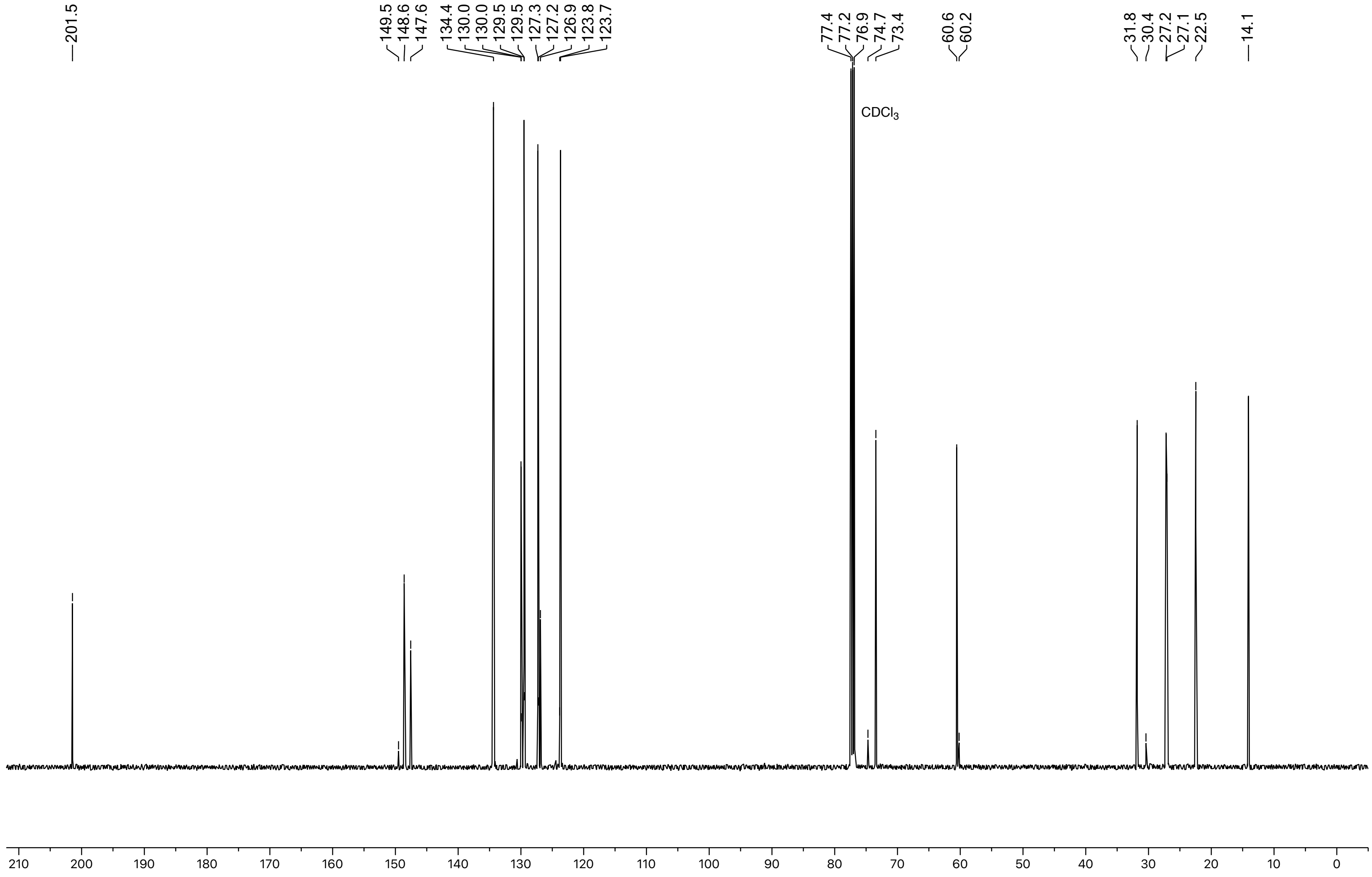

^1H NMR, 500 MHz, $CDCl_3$, **7c**

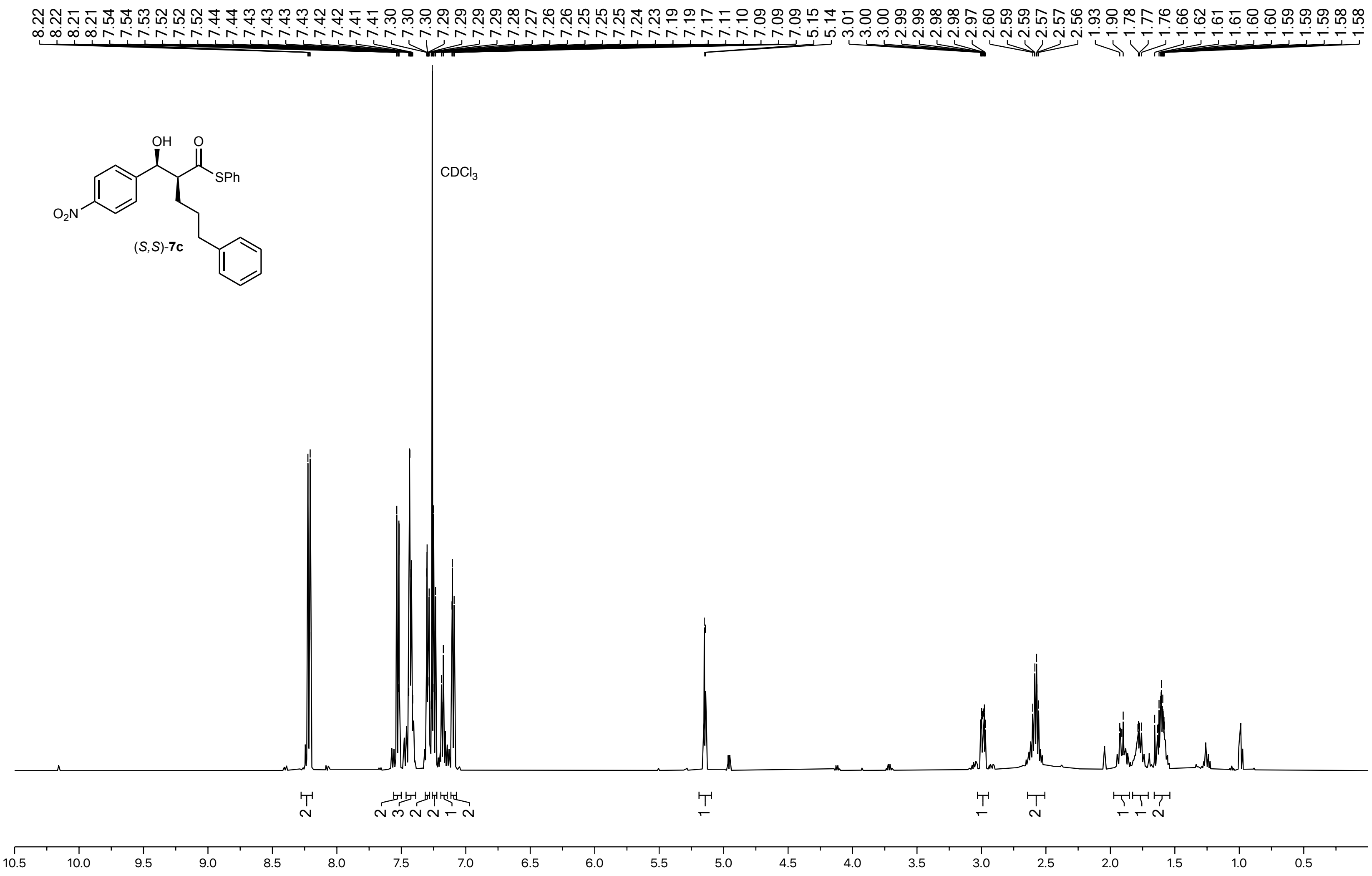

$^{13}C\{^{1}H\}$ NMR, 126 MHz, $CDCl_3$, **7c**

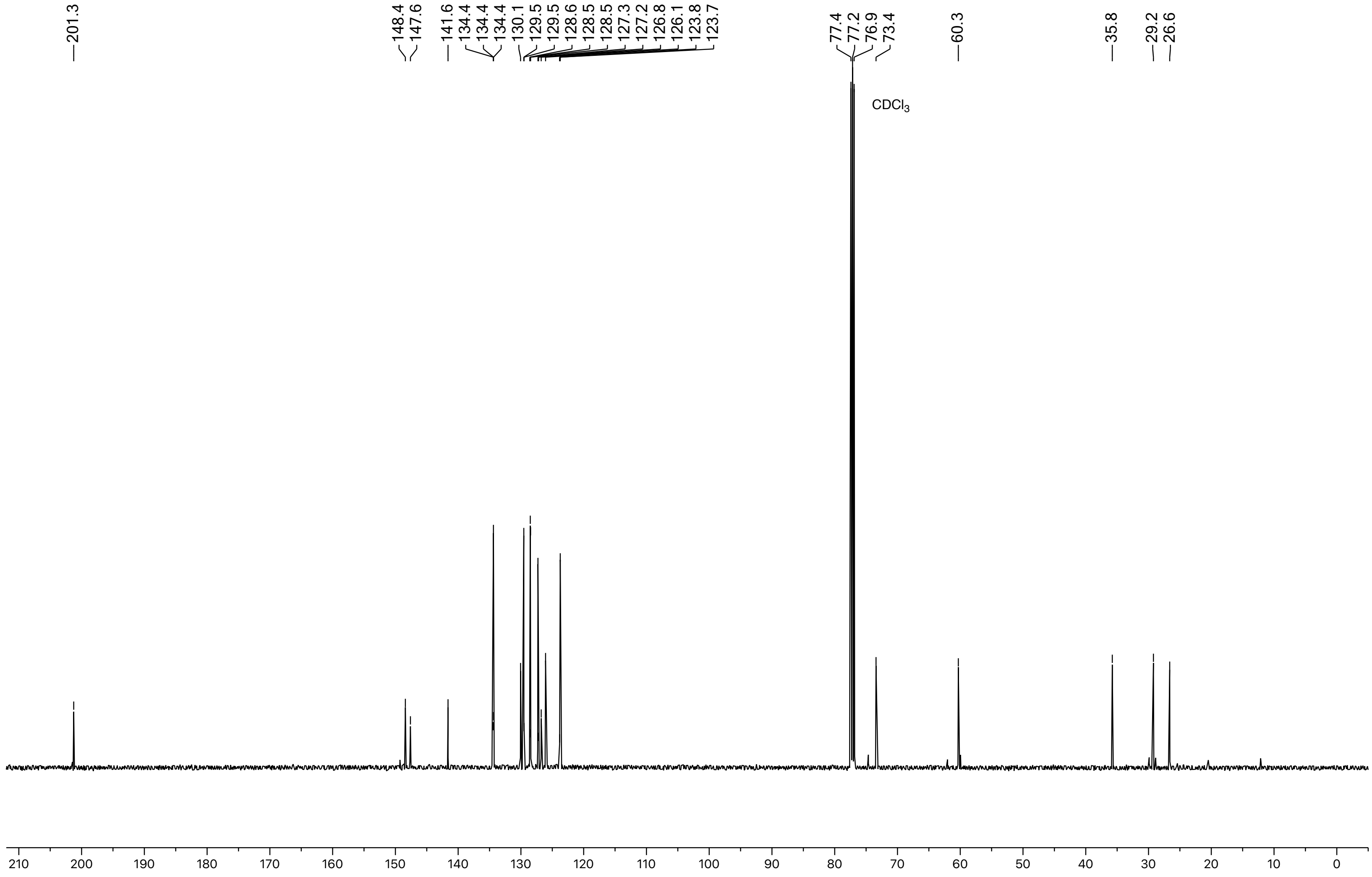

^{1}H NMR, 500 MHz, $CDCl_3$, **7d**

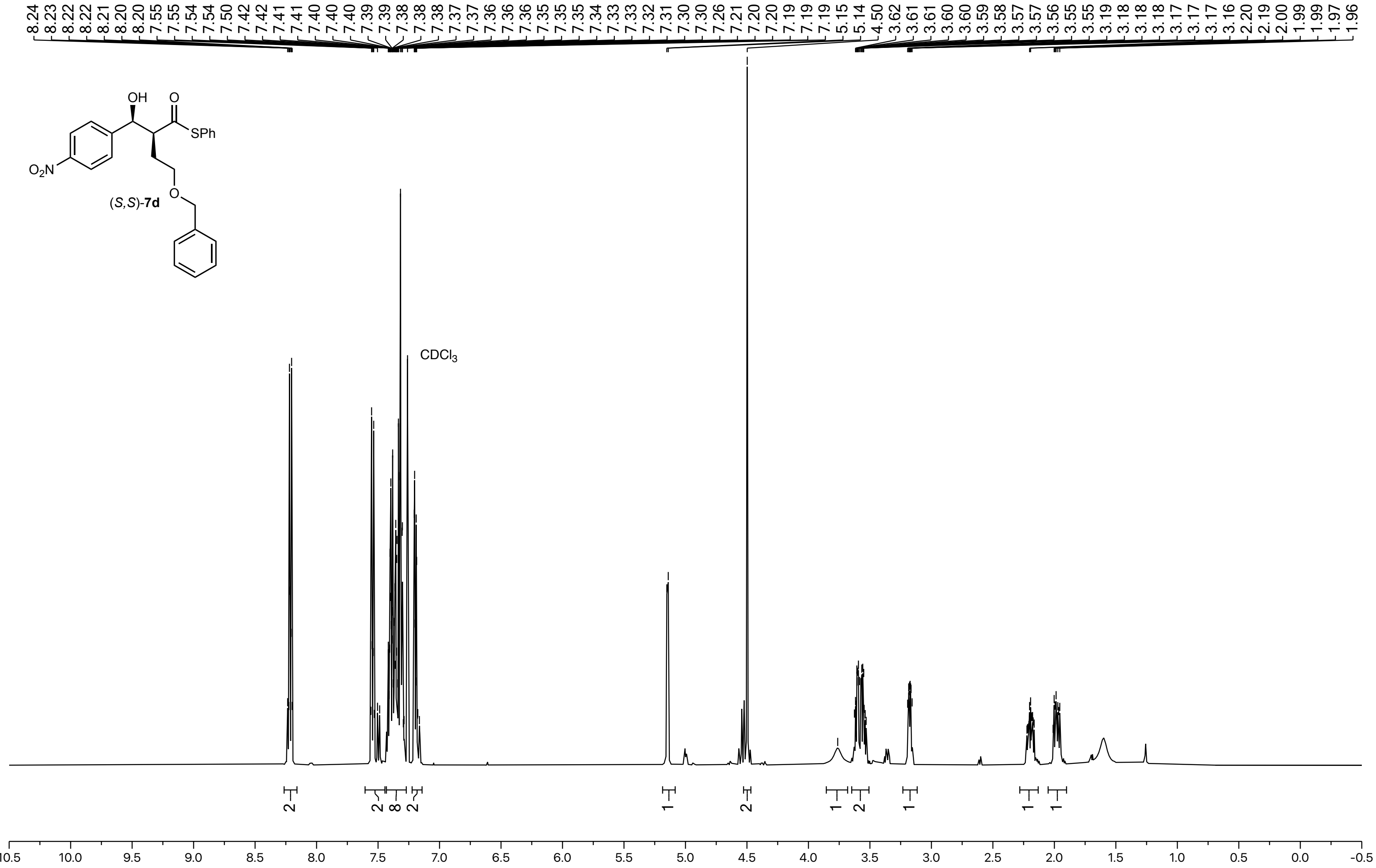

$^{13}C\{^{1}H\}$ NMR, 126 MHz, $CDCl_3$, **7d**

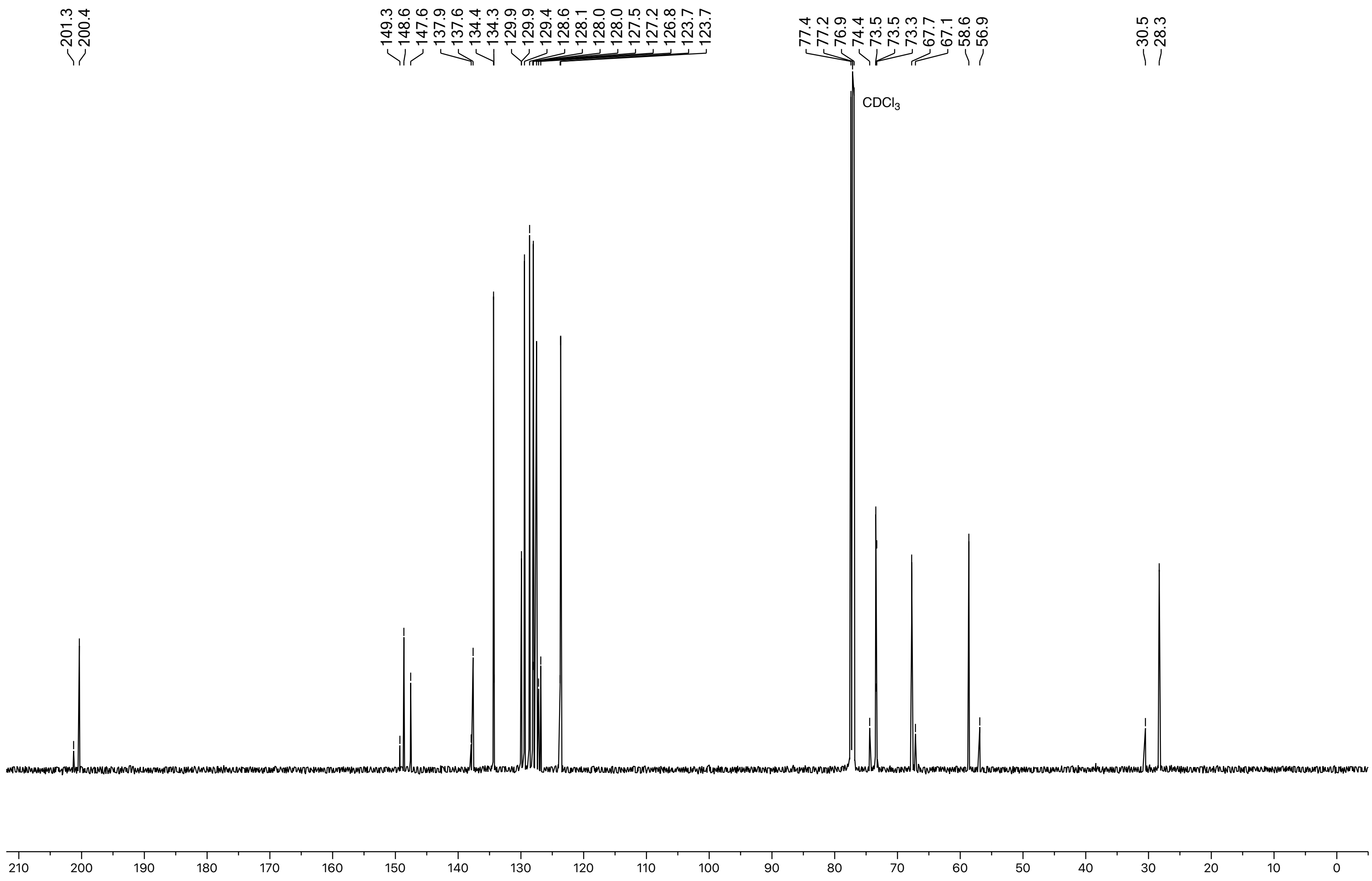

^{1}H NMR, 500 MHz, $CDCl_3$, **7e**

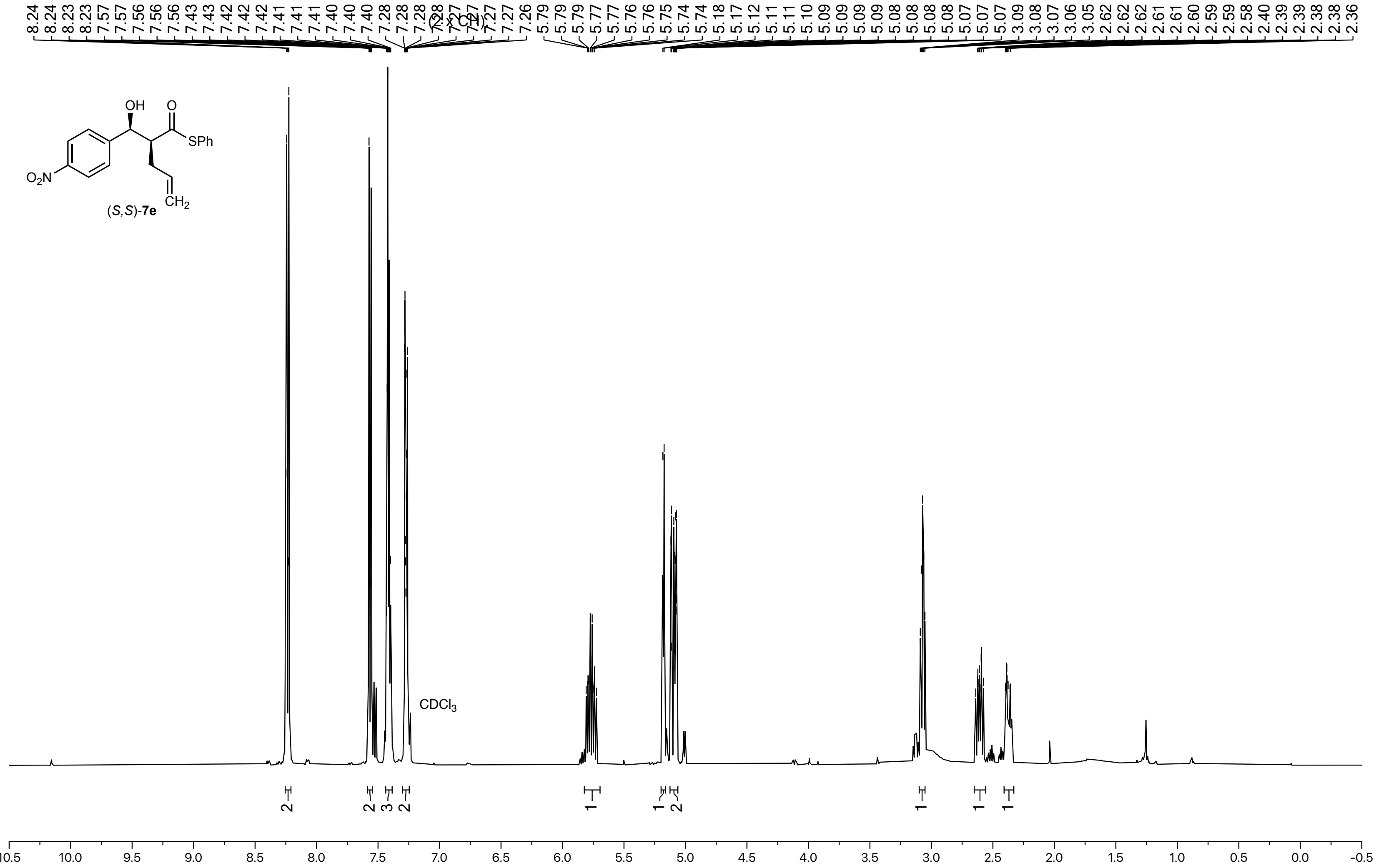

$^{13}C\{^{1}H\}$ NMR, 126 MHz, $CDCl_3$, **7e**

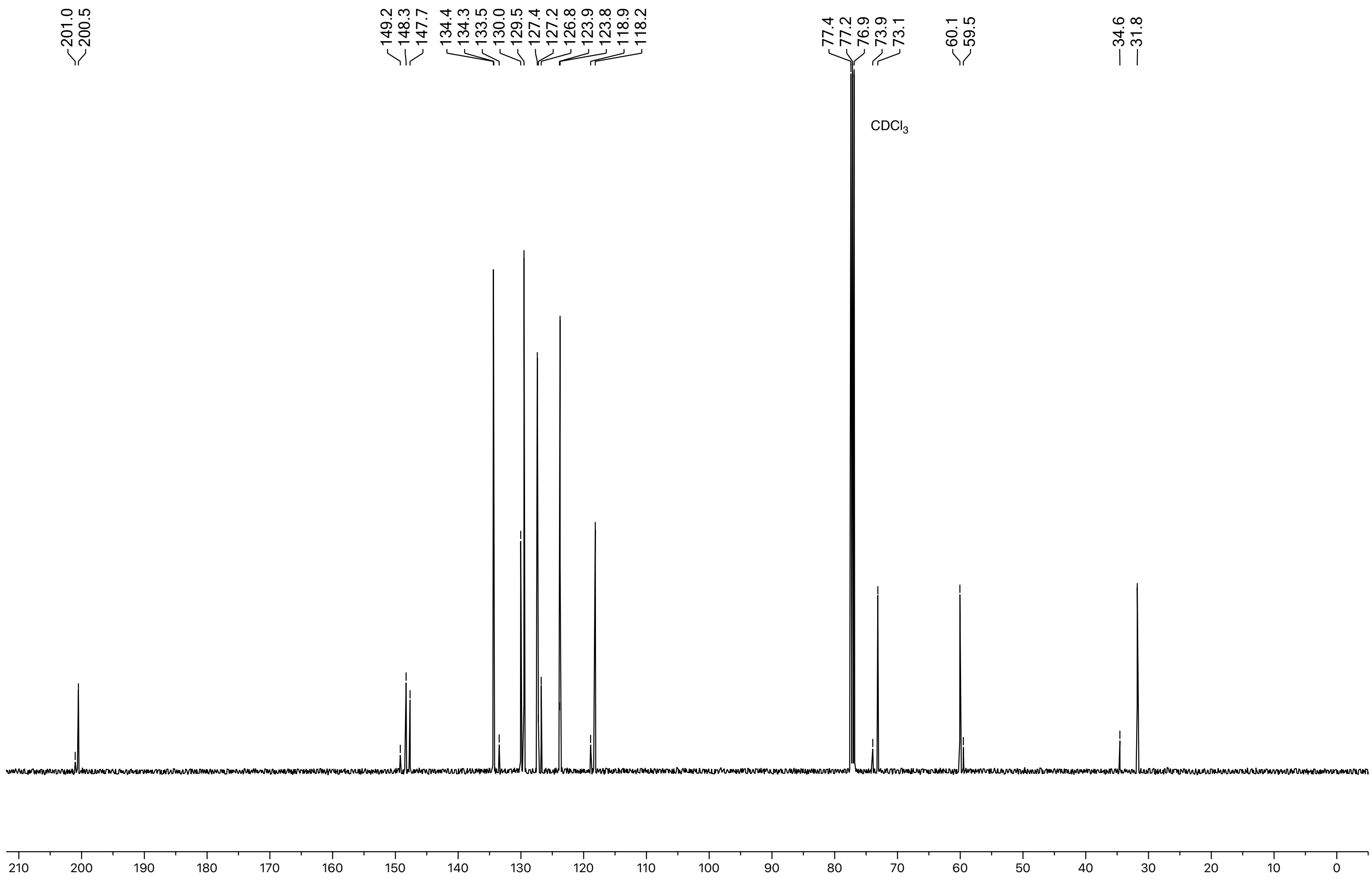

^1H NMR, 500 MHz, $CDCl_3$, **7f**

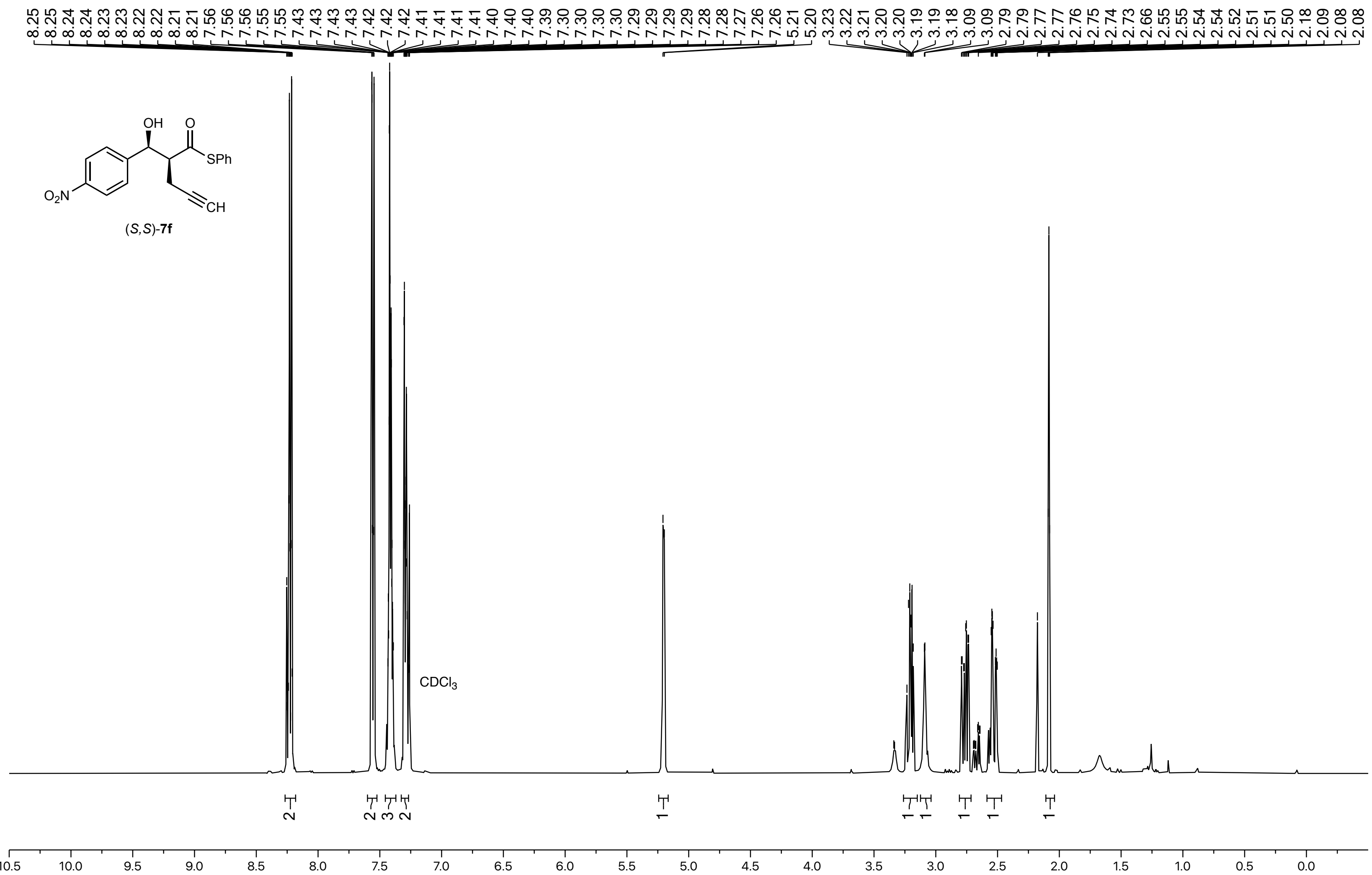

$^{13}C\{^{1}H\}$ NMR, 126 MHz, $CDCl_3$, **7f**

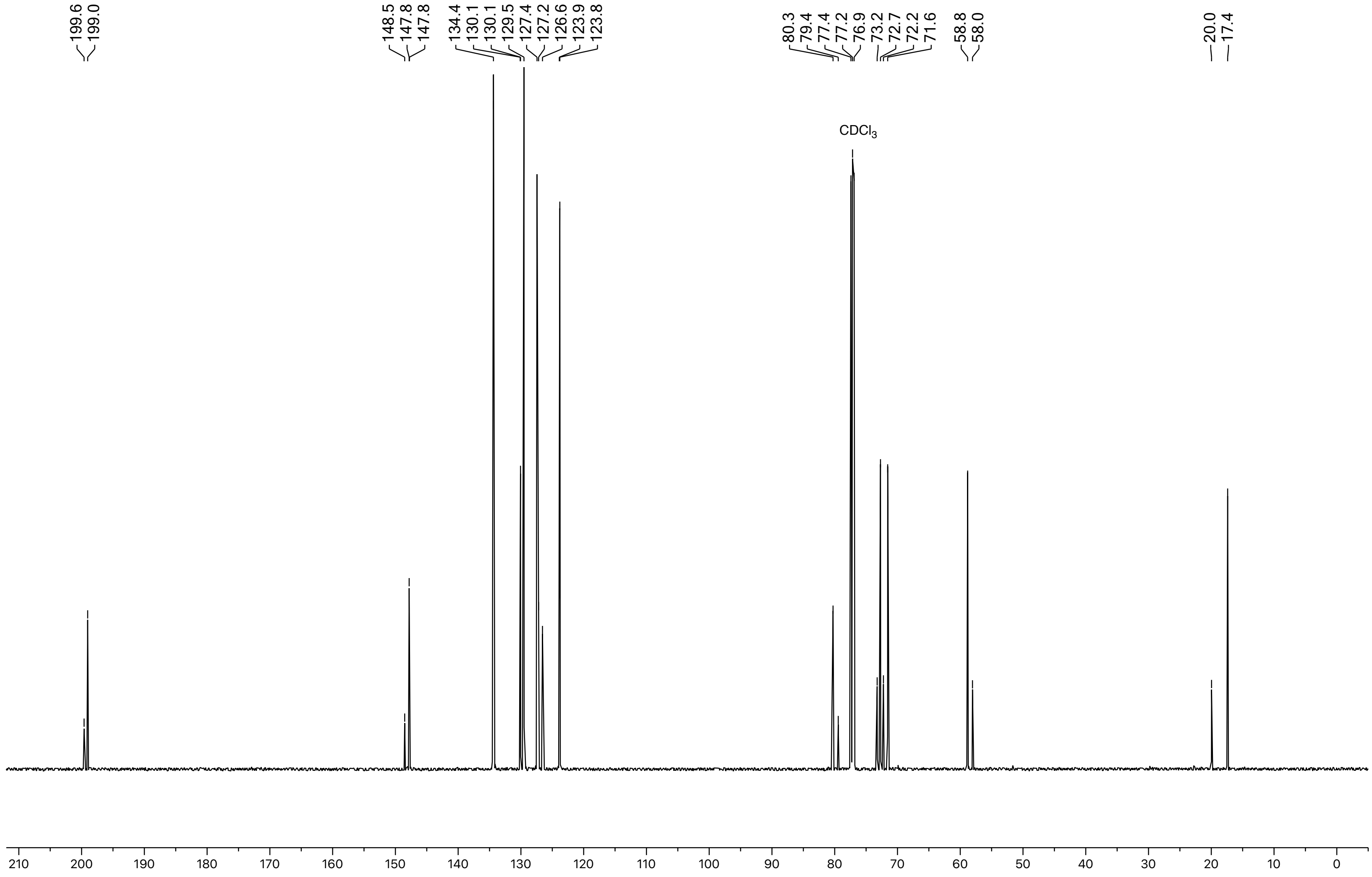

¹H NMR, 500 MHz, $CDCl_3$, **7g**

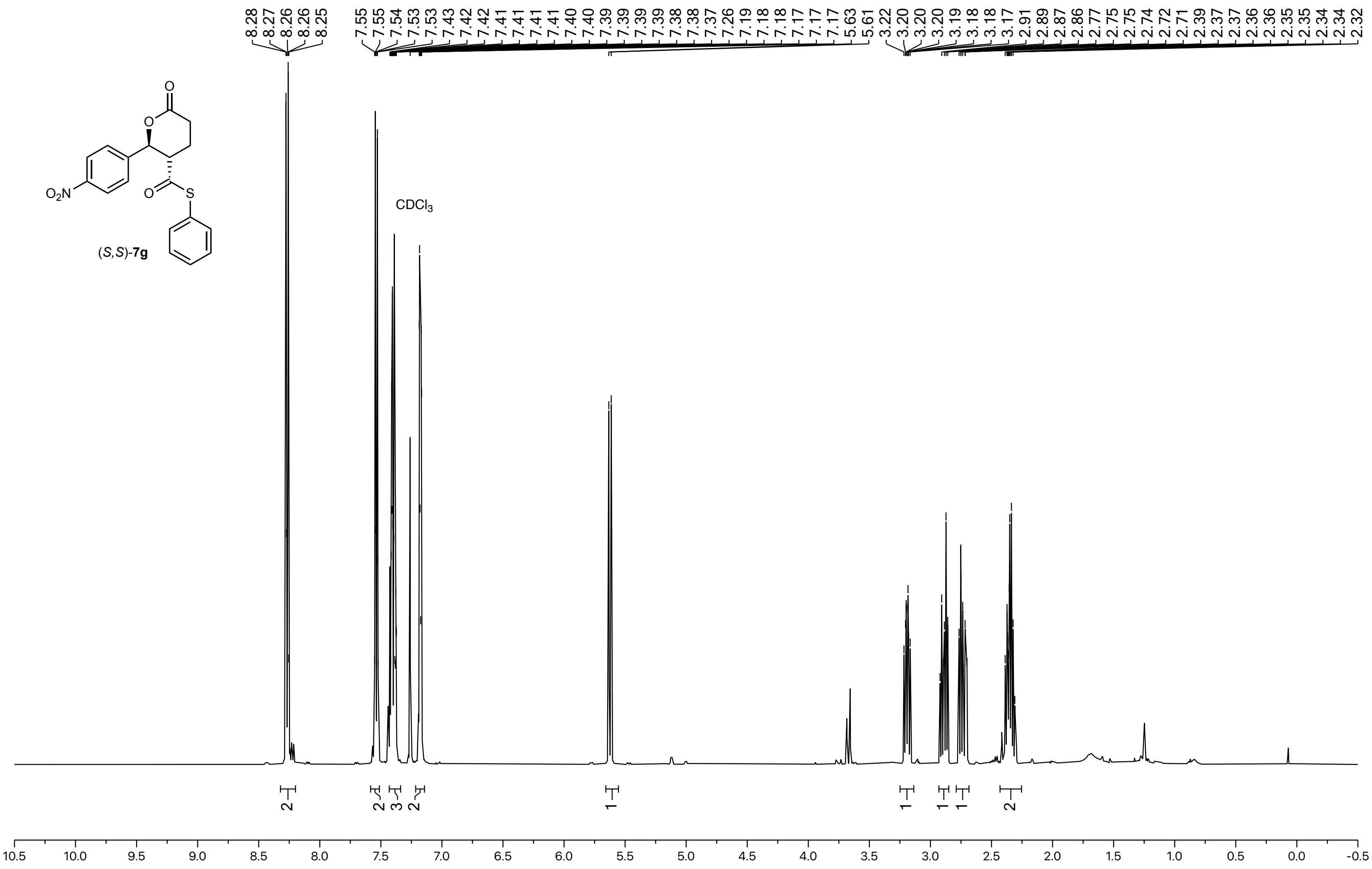

(*S*,*S*)-**7g**

$^{13}C\{^{1}H\}$ NMR, 126 MHz, $CDCl_3$, **7g**

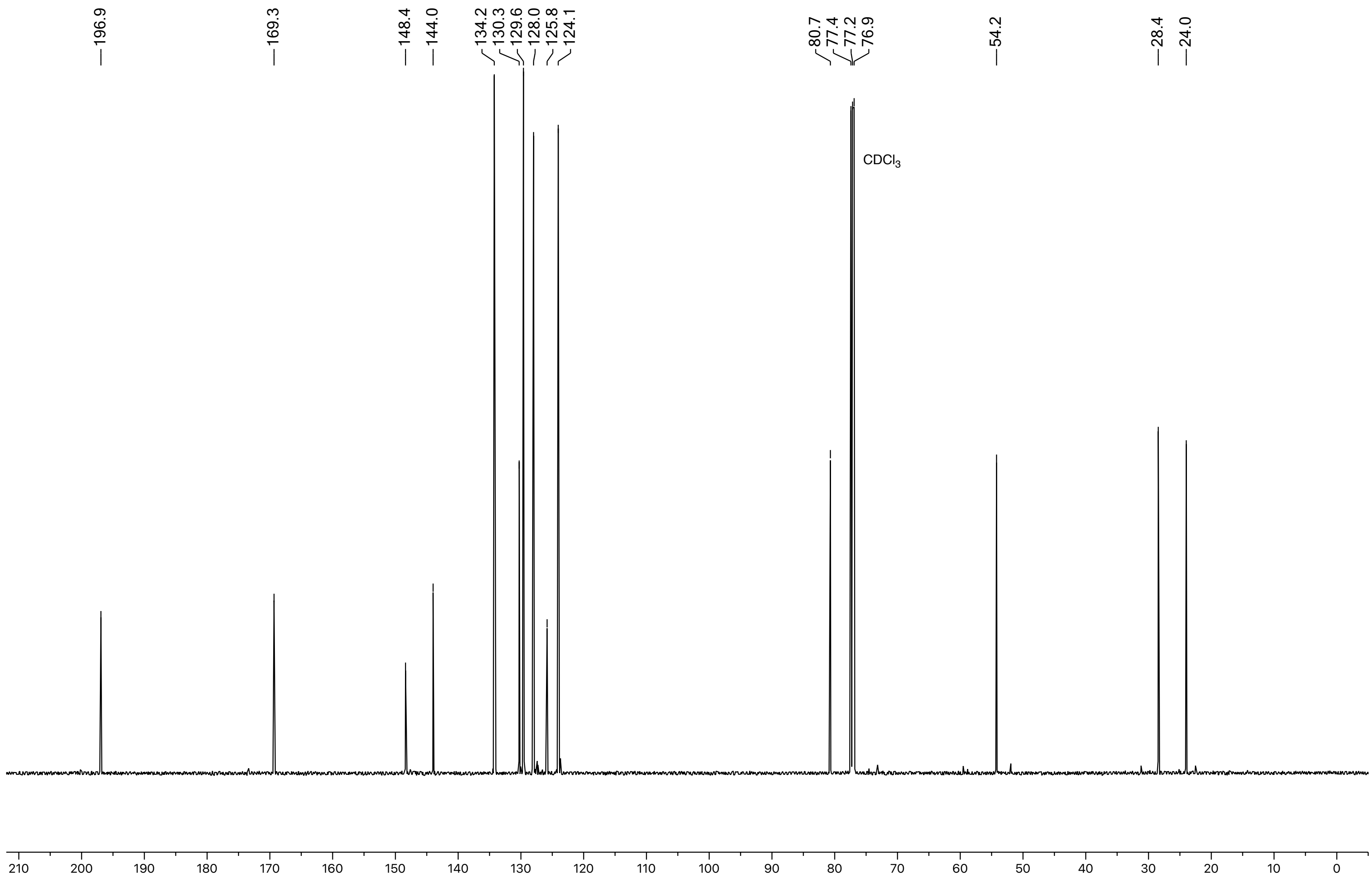

^1H NMR, 500 MHz, $CDCl_3$, **5w**

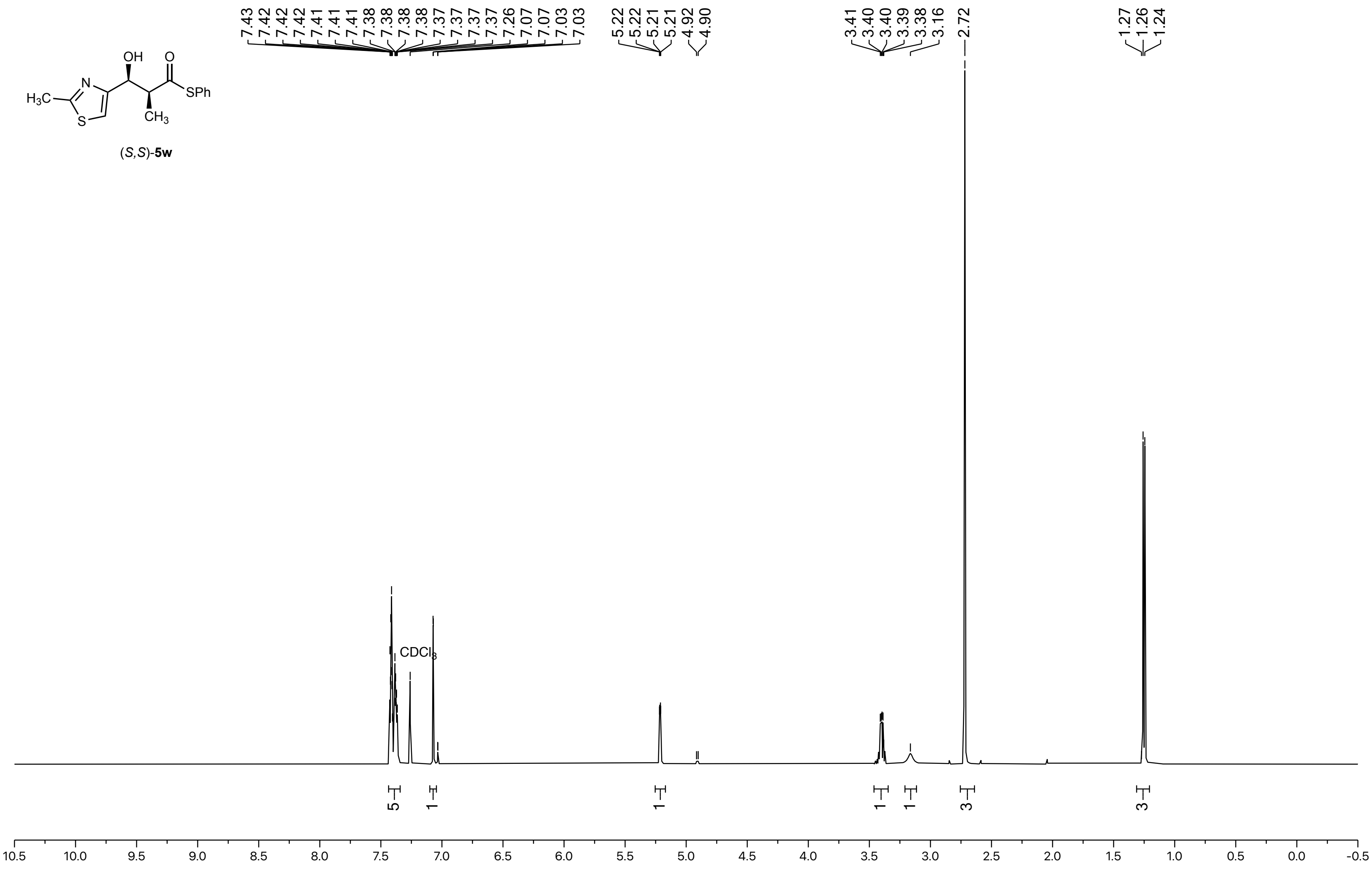

$^{13}C\{^{1}H\}$ NMR, 126 MHz, $CDCl_3$, **5w**

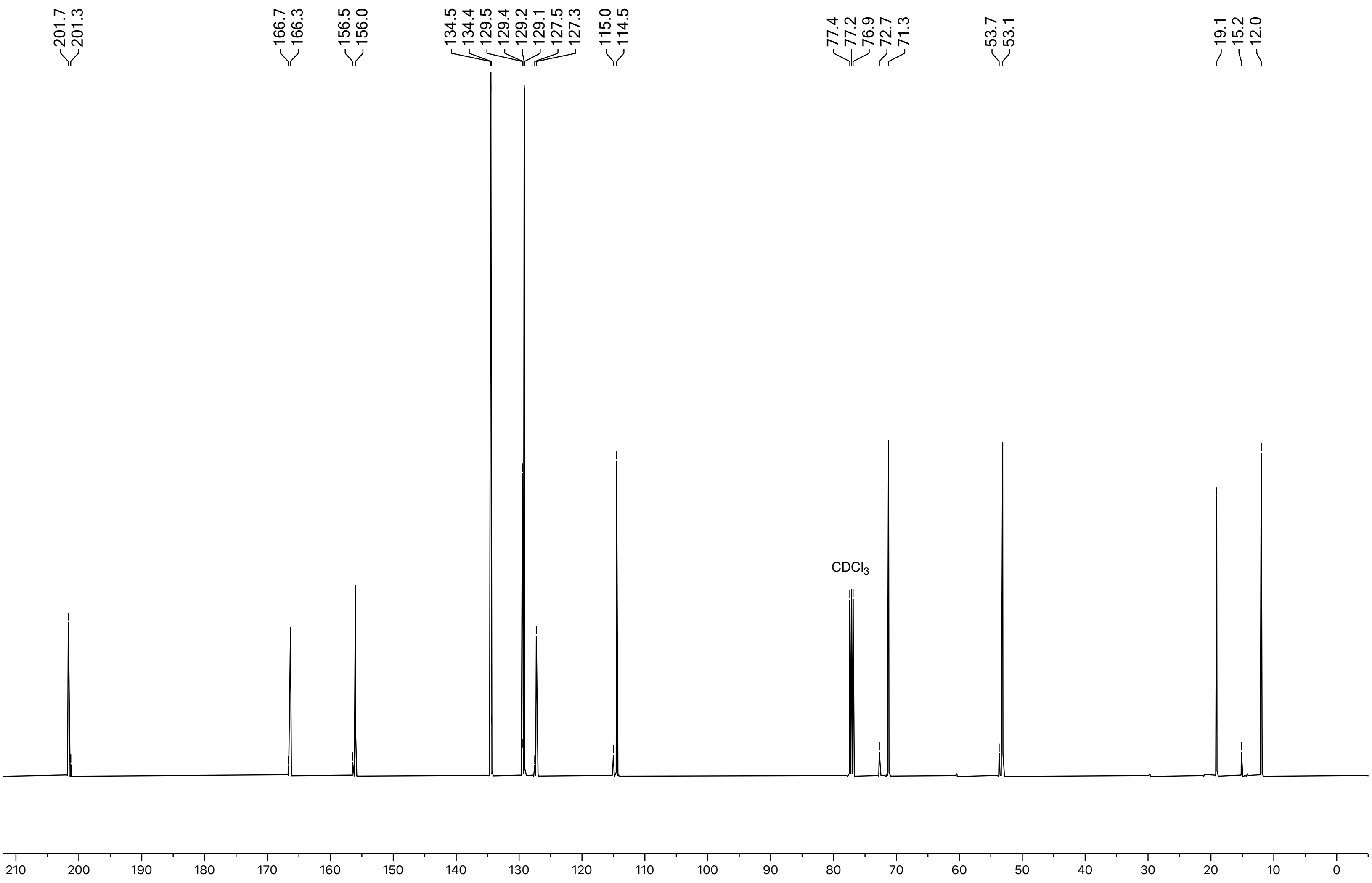

^{1}H NMR, 500 MHz, $CDCl_3$, **9**

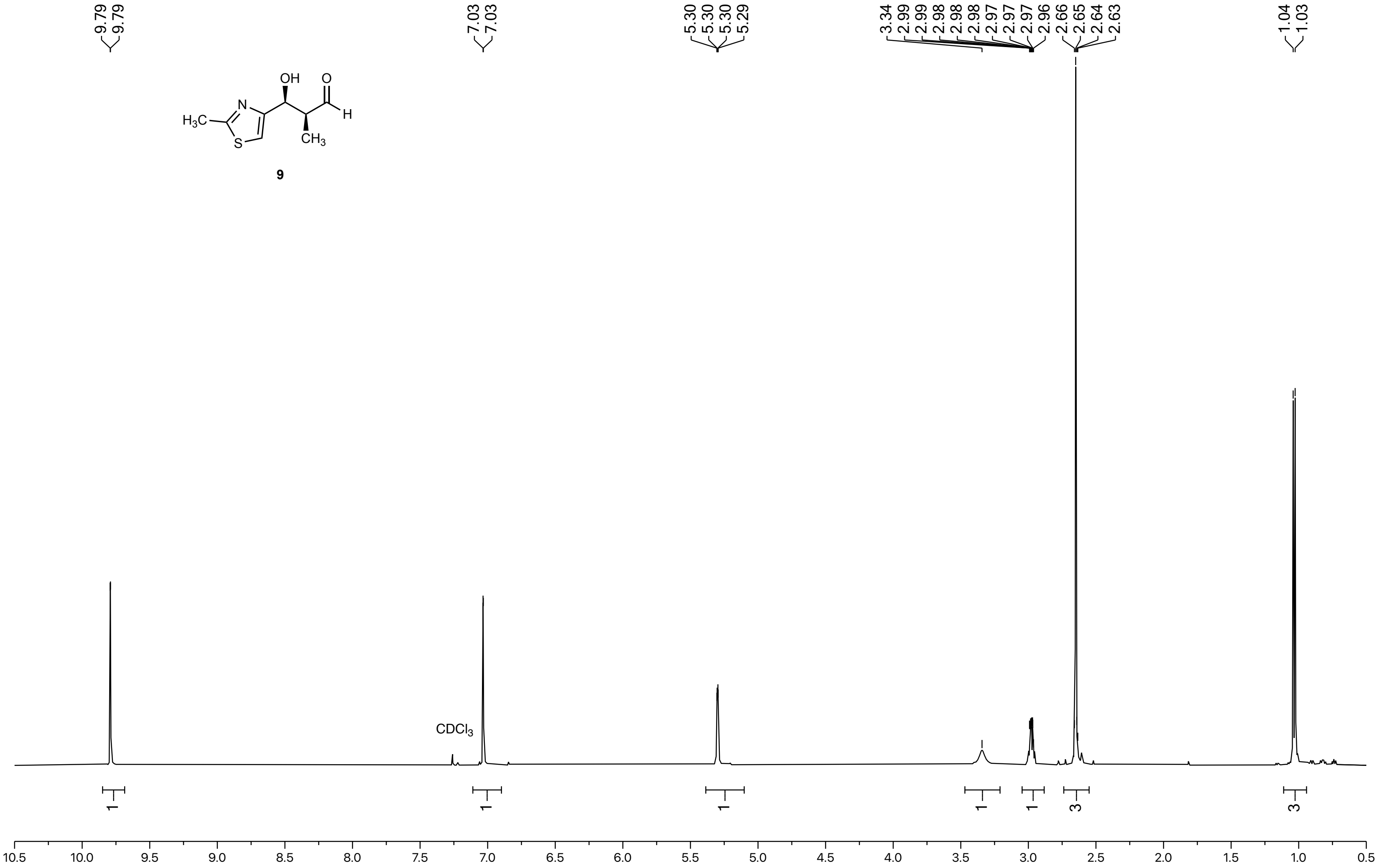

$^{13}C\{^{1}H\}$ NMR, 126 MHz, $CDCl_3$, **9**

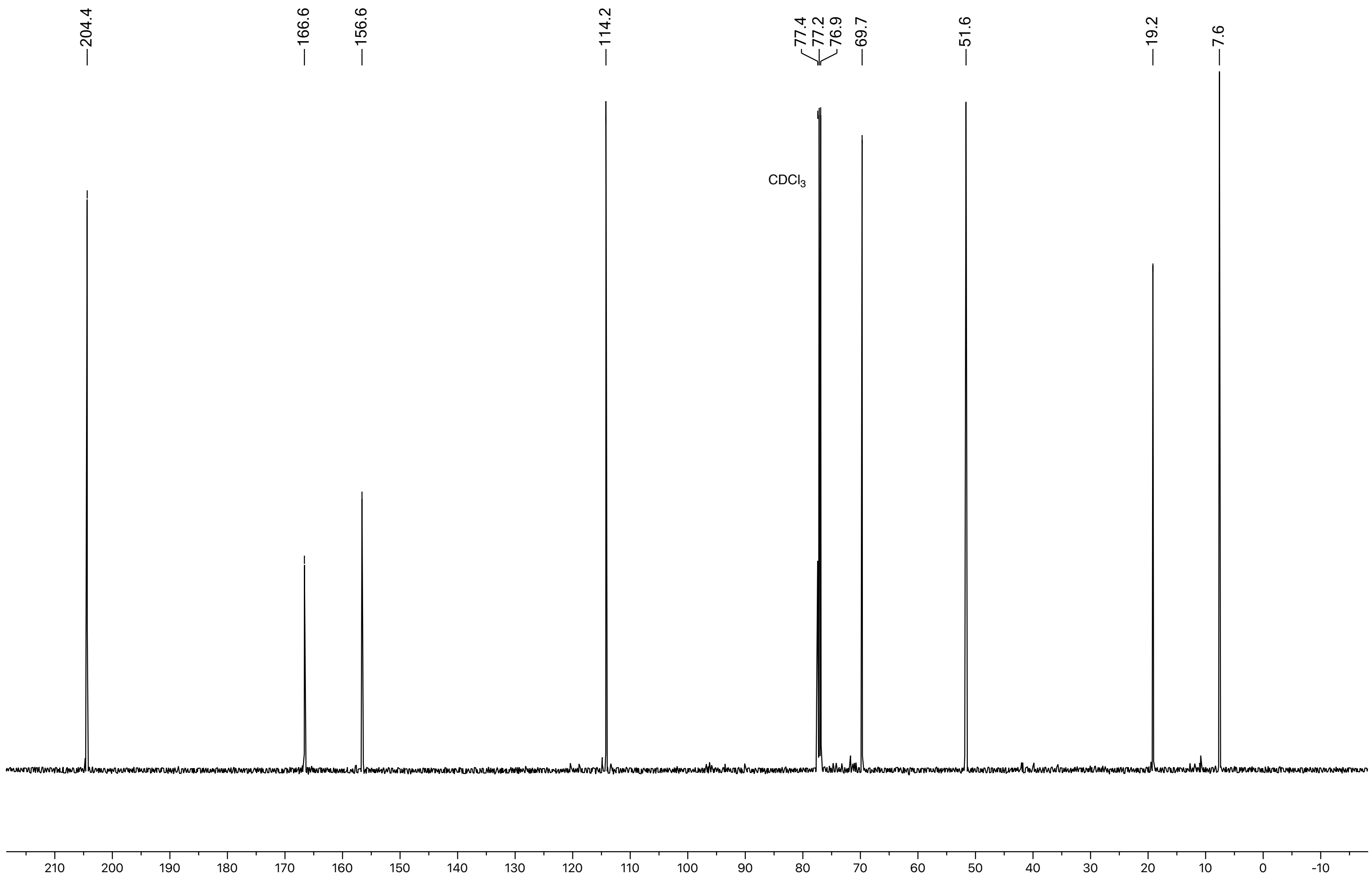

^{1}H NMR, 500 MHz, $CDCl_3$, **10**

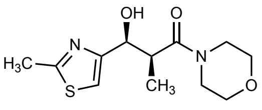

10

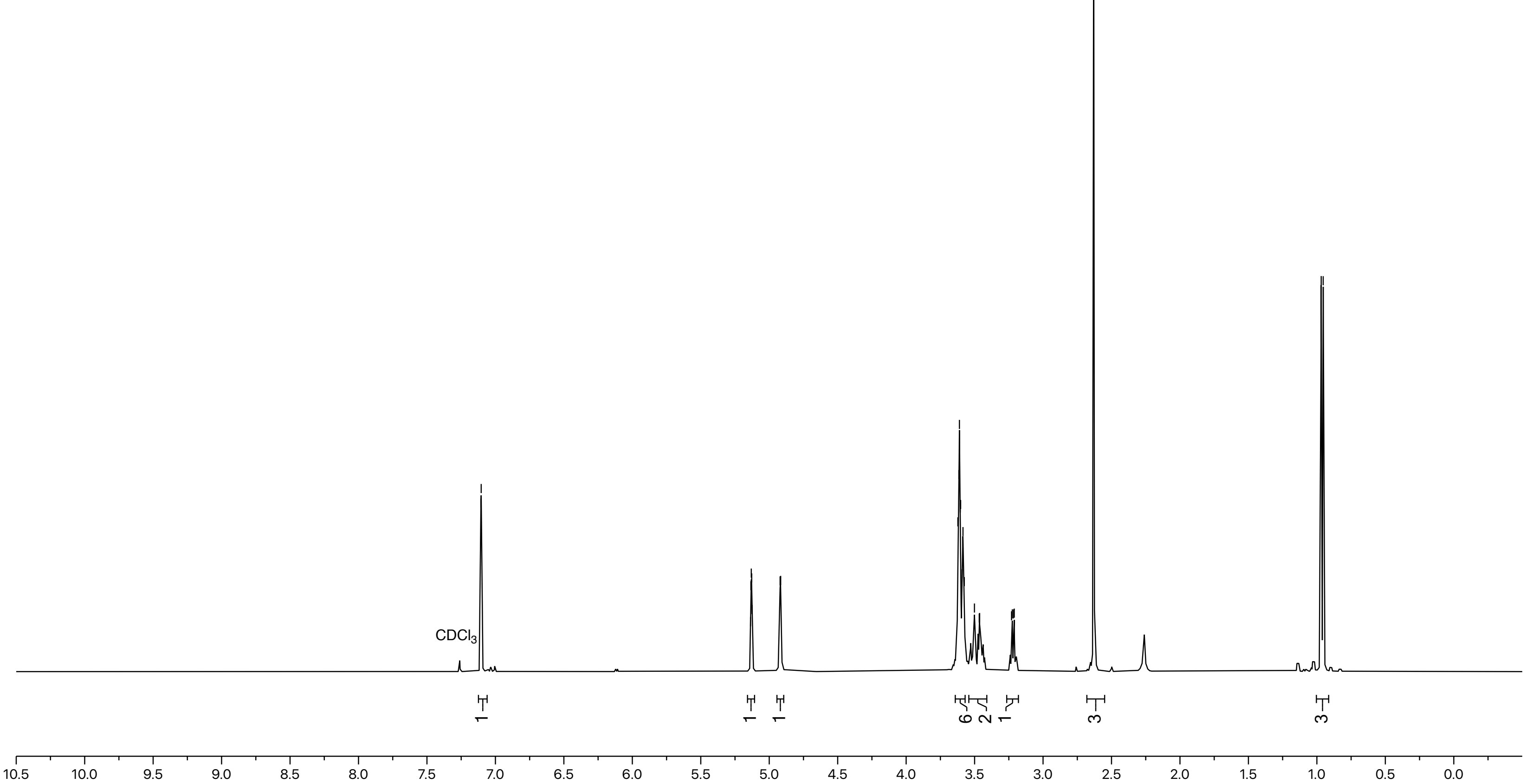

$^{13}C\{^{1}H\}$ NMR, 126 MHz, $CDCl_3$, **10**

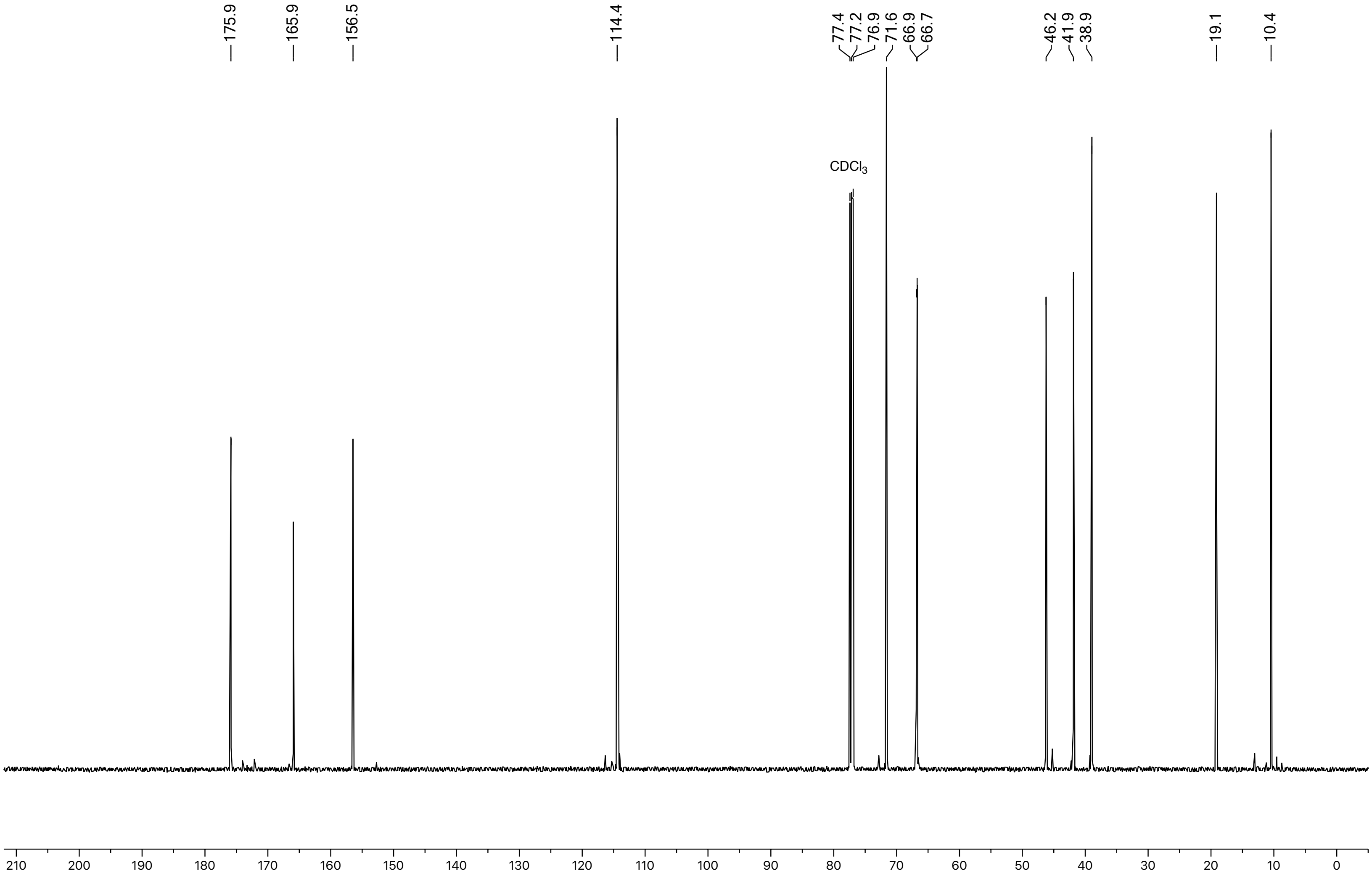

^{1}H NMR, 500 MHz, $CDCl_3$, **11**

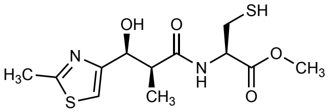

11

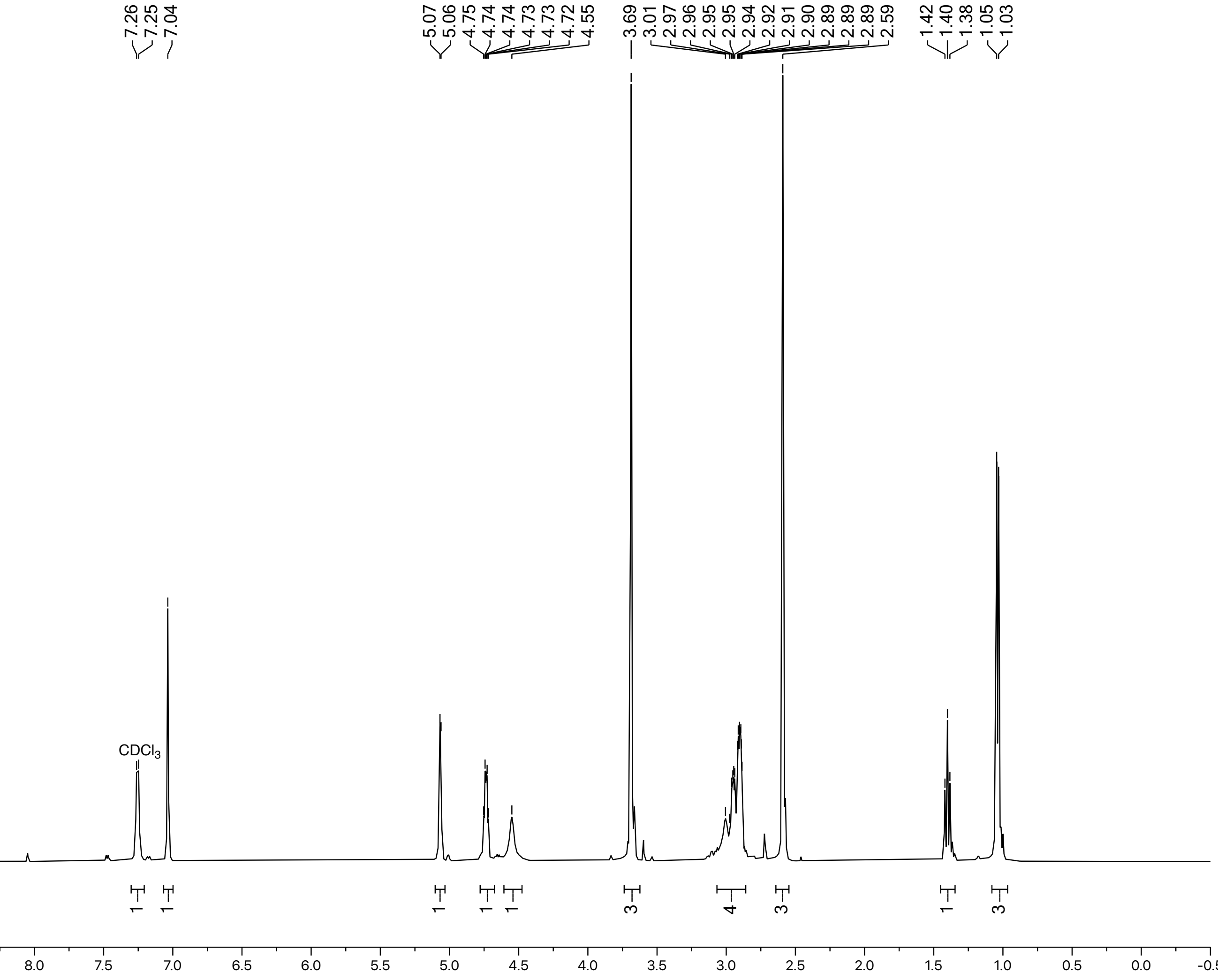

$^{13}C\{^{1}H\}$ NMR, 126 MHz, $CDCl_3$, **11**

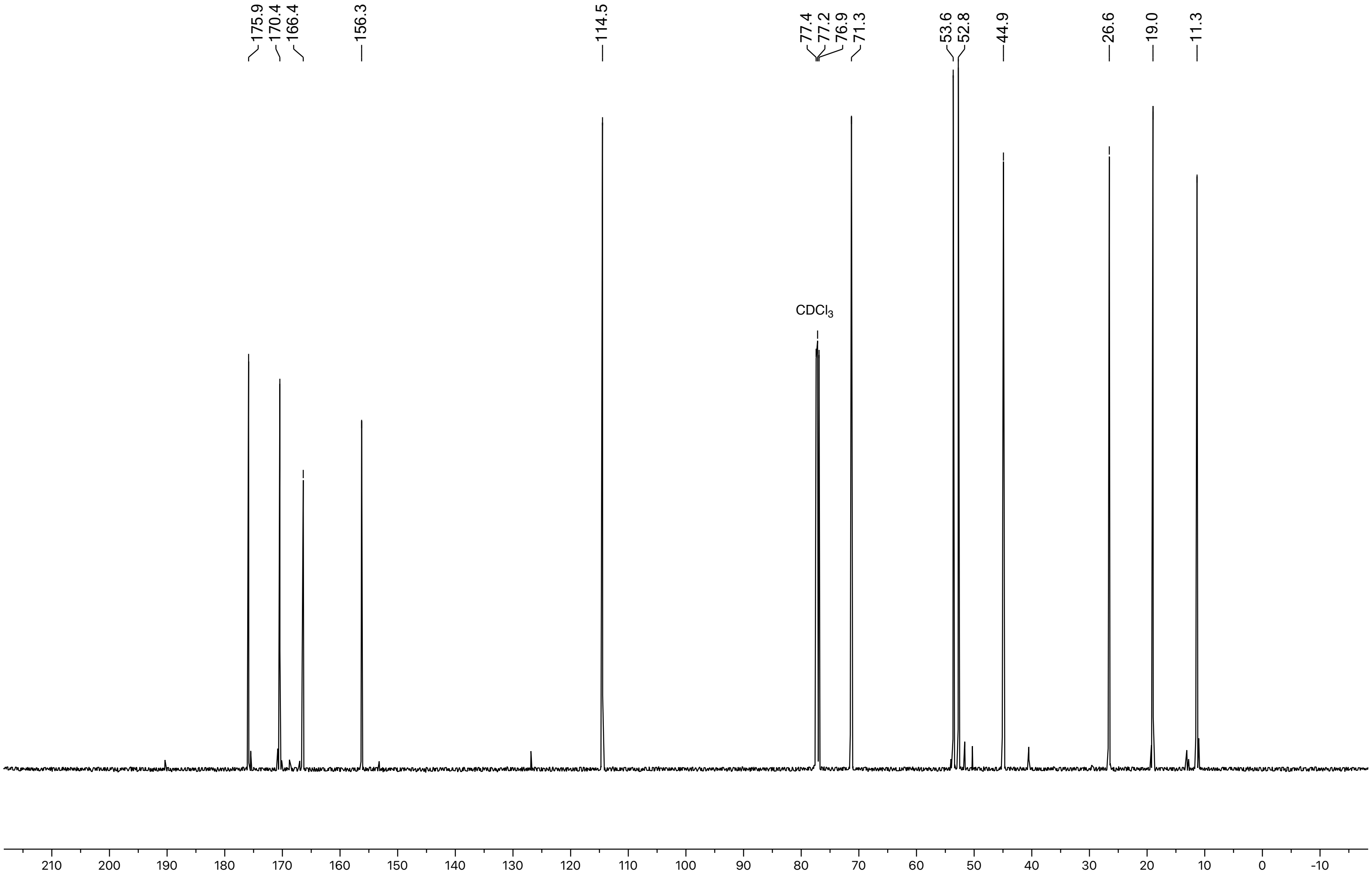

^{1}H NMR, 500 MHz, $CDCl_3$, **12**

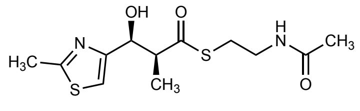

12

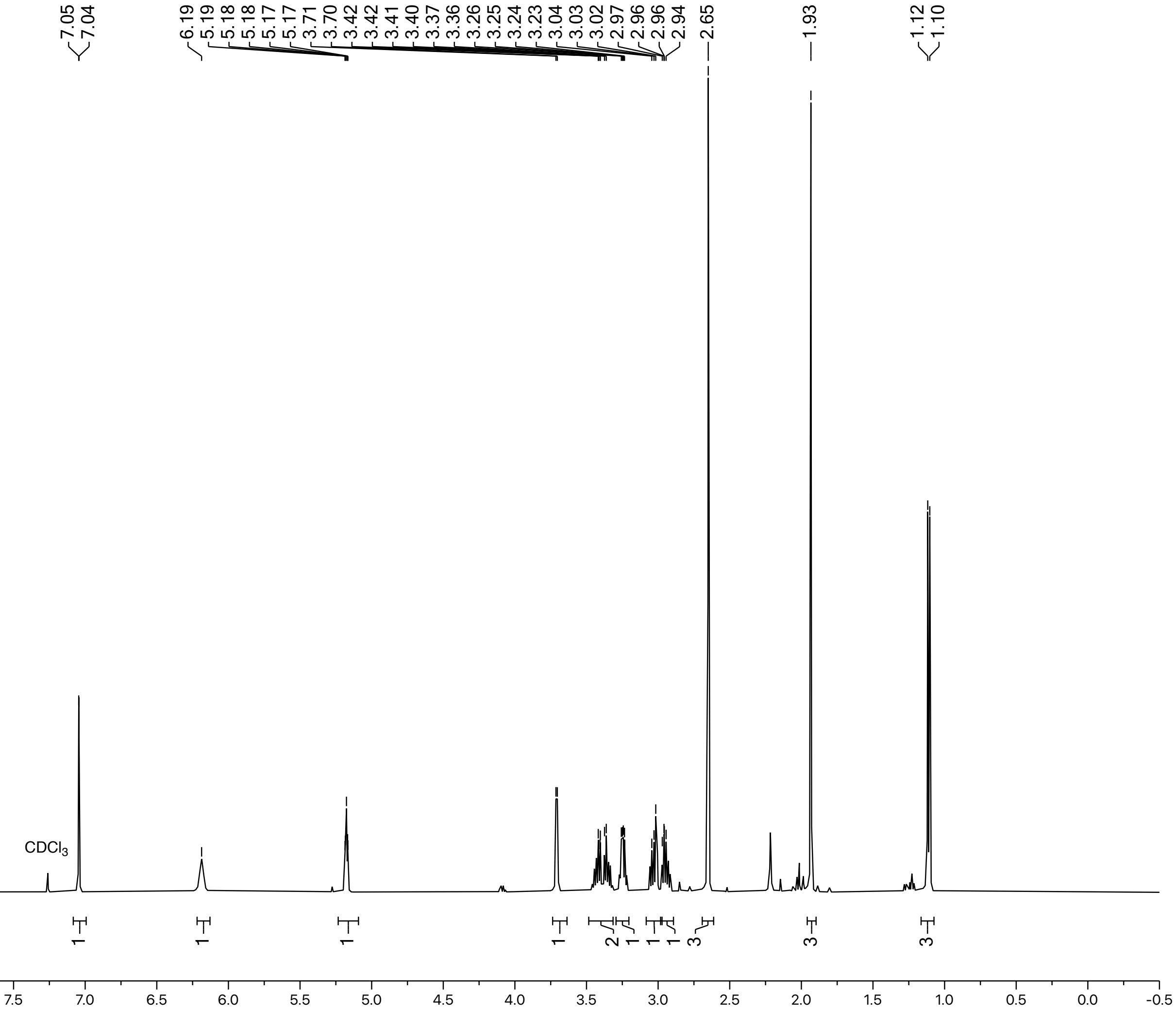

$^{13}C\{^1H\}$ NMR, 126 MHz, $CDCl_3$, **12**

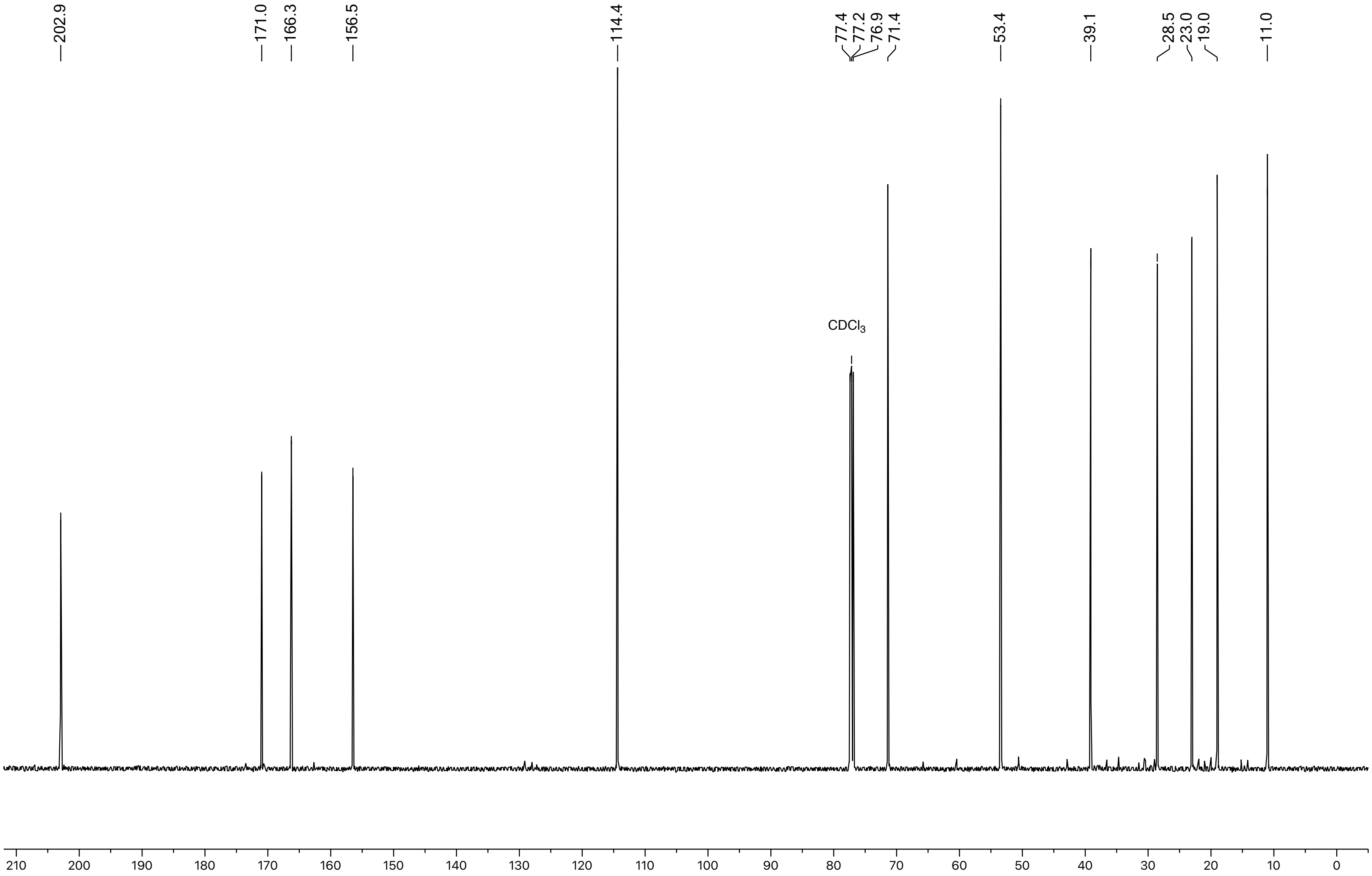

^{1}H NMR, 500 MHz, $CDCl_3$, **6x**

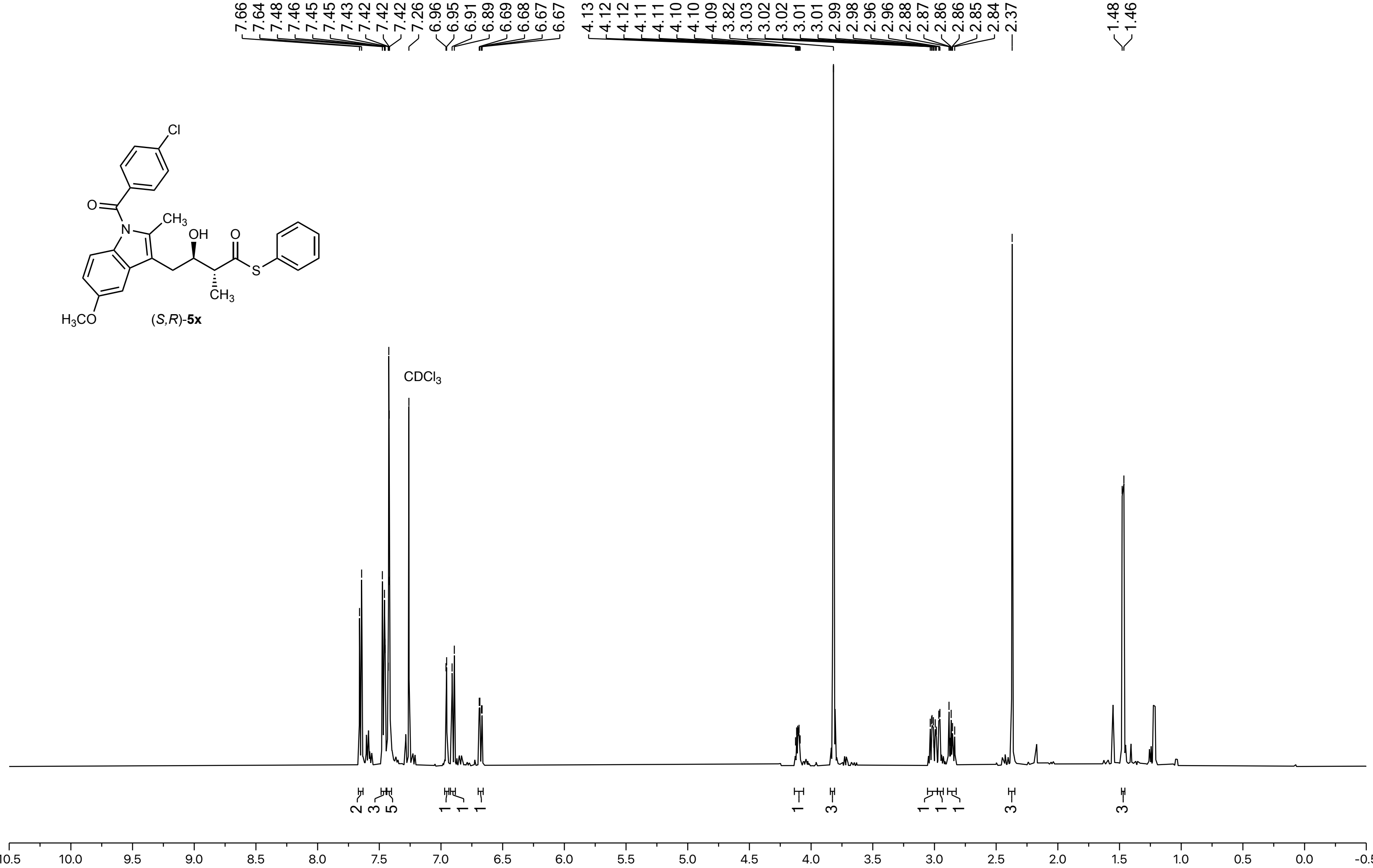

$^{13}C\{^{1}H\}$ NMR, 126 MHz, $CDCl_3$, **6x**

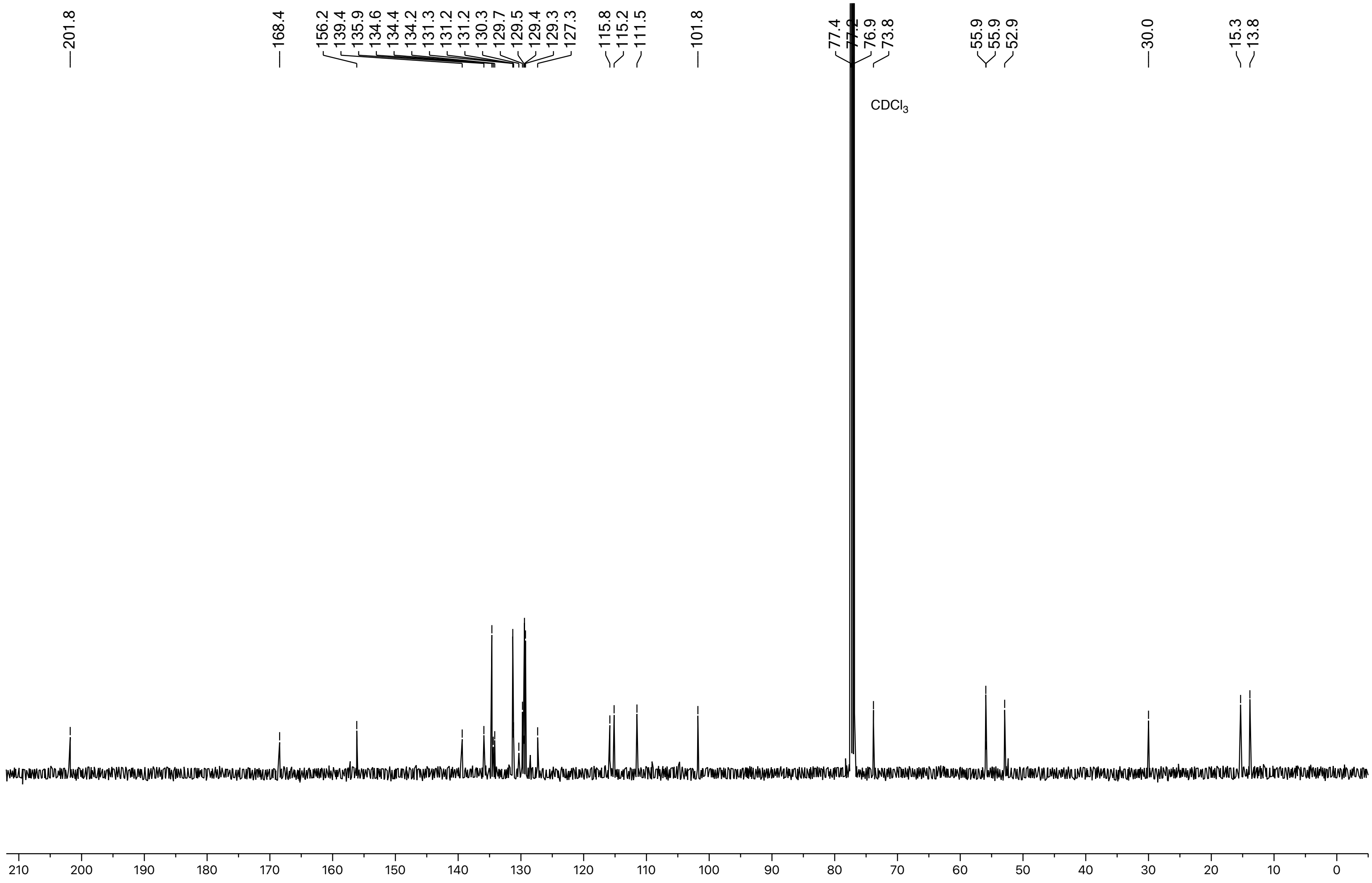

¹H NMR, 500 MHz, $CDCl_3$, **S11**

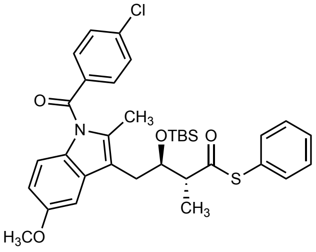

S11

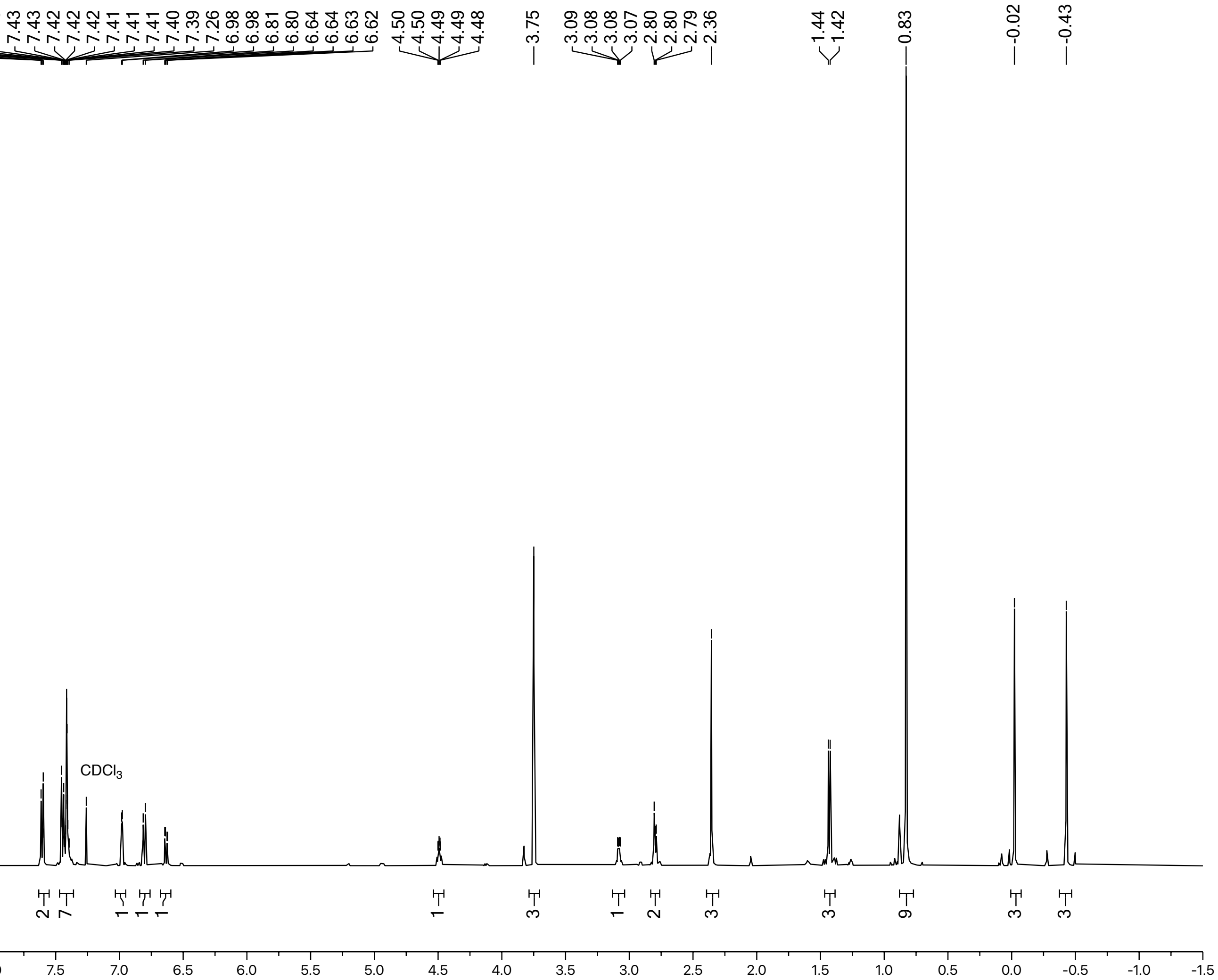

$^{13}C\{^{1}H\}$ NMR, 126 MHz, $CDCl_3$, **S11**

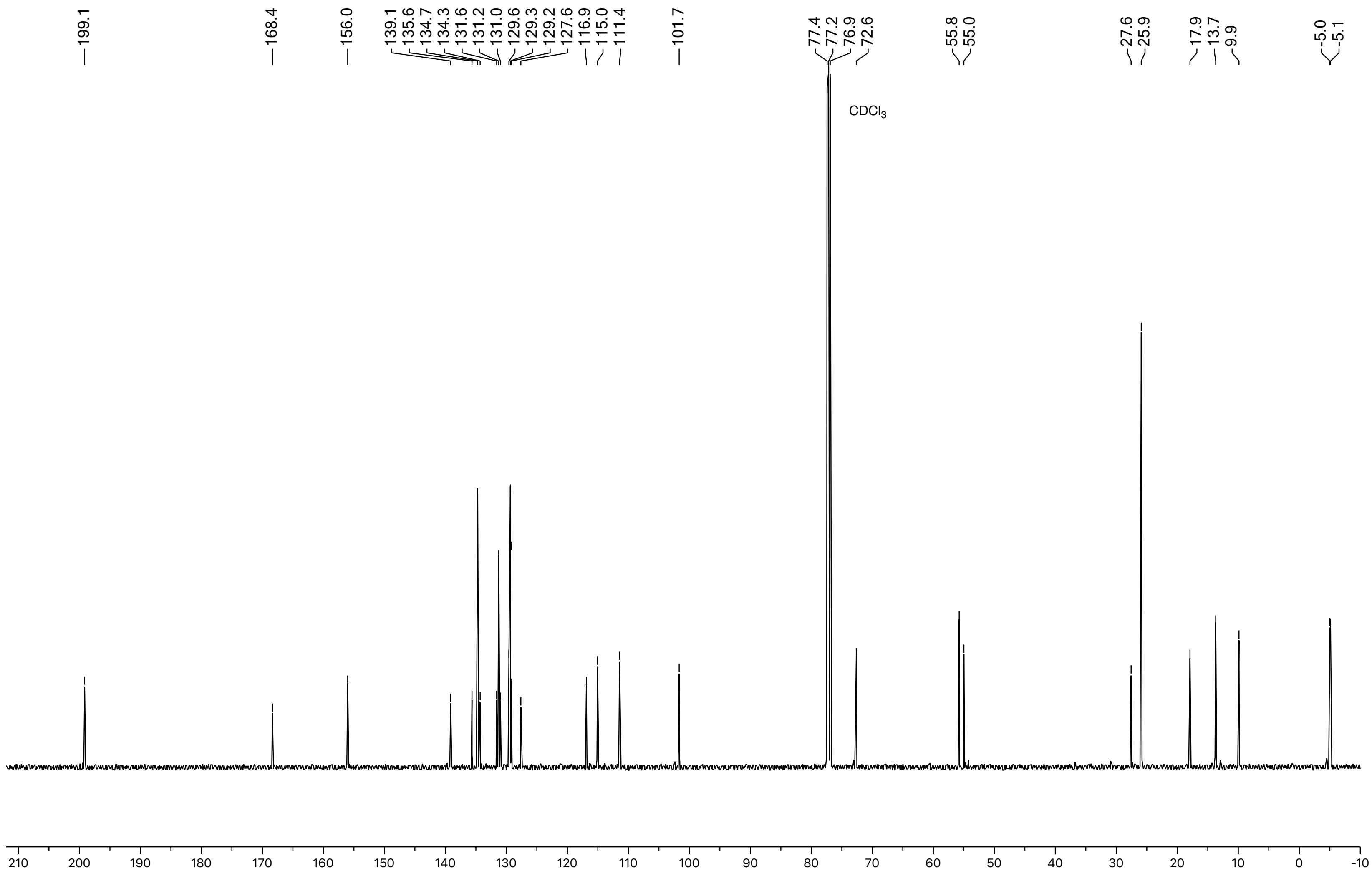

^{1}H NMR, 500 MHz, $CDCl_3$, **16**

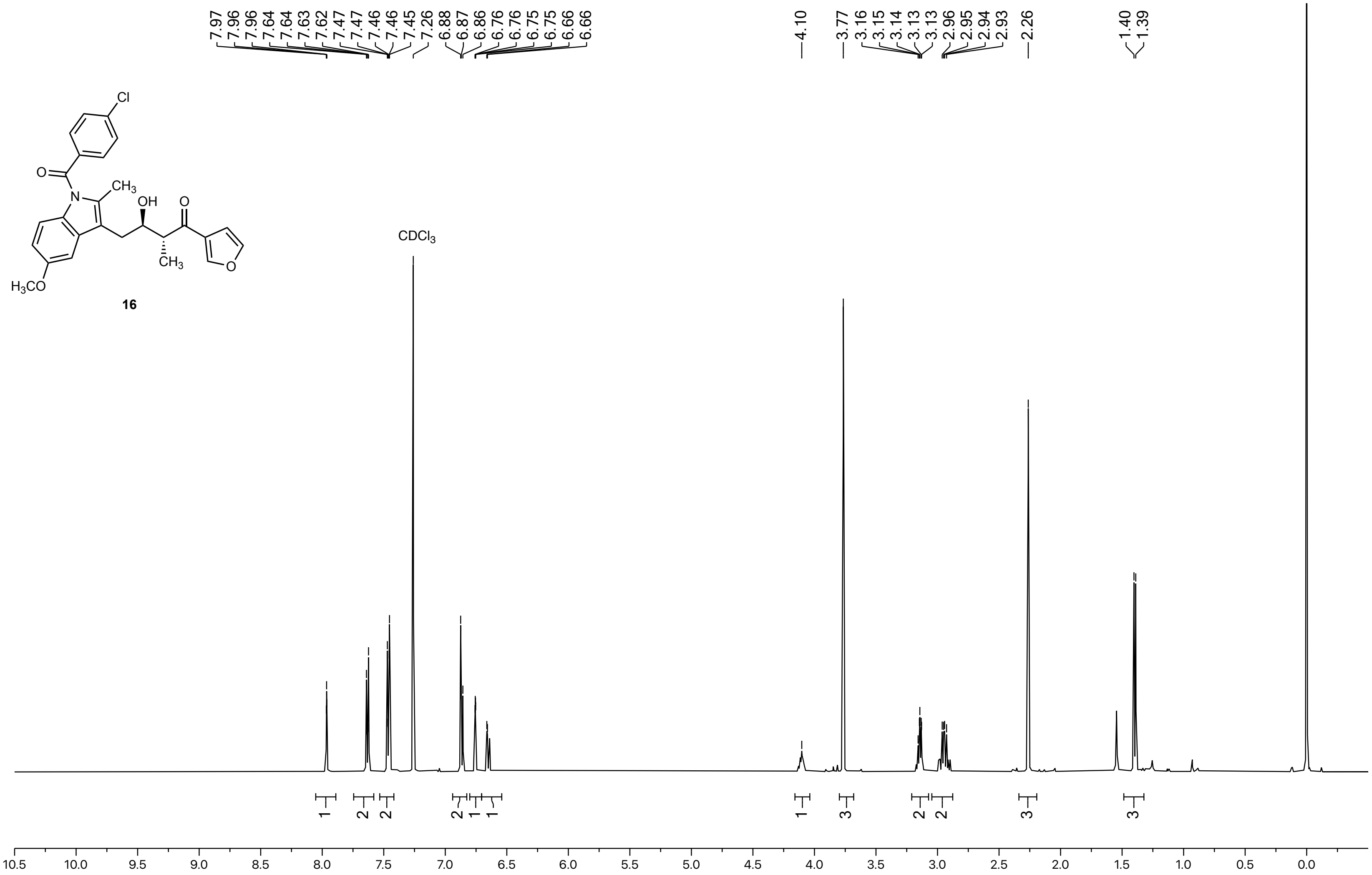

$^{13}C\{^{1}H\}$ NMR, 126 MHz, $CDCl_3$, **16**

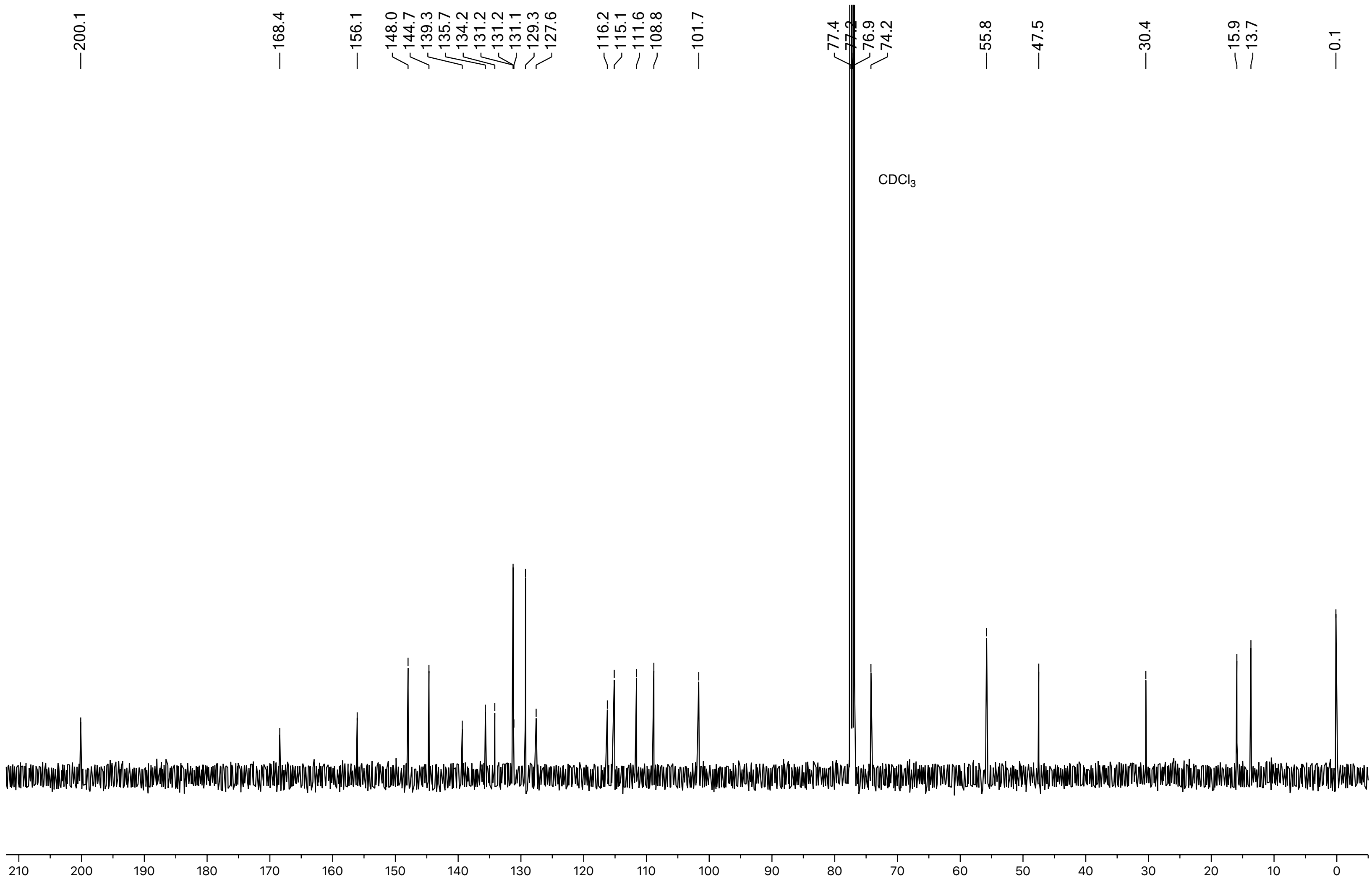

^{1}H NMR, 500 MHz, $CDCl_3$, **17**

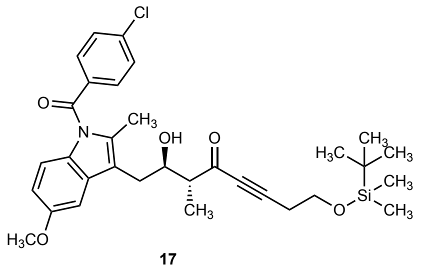

17

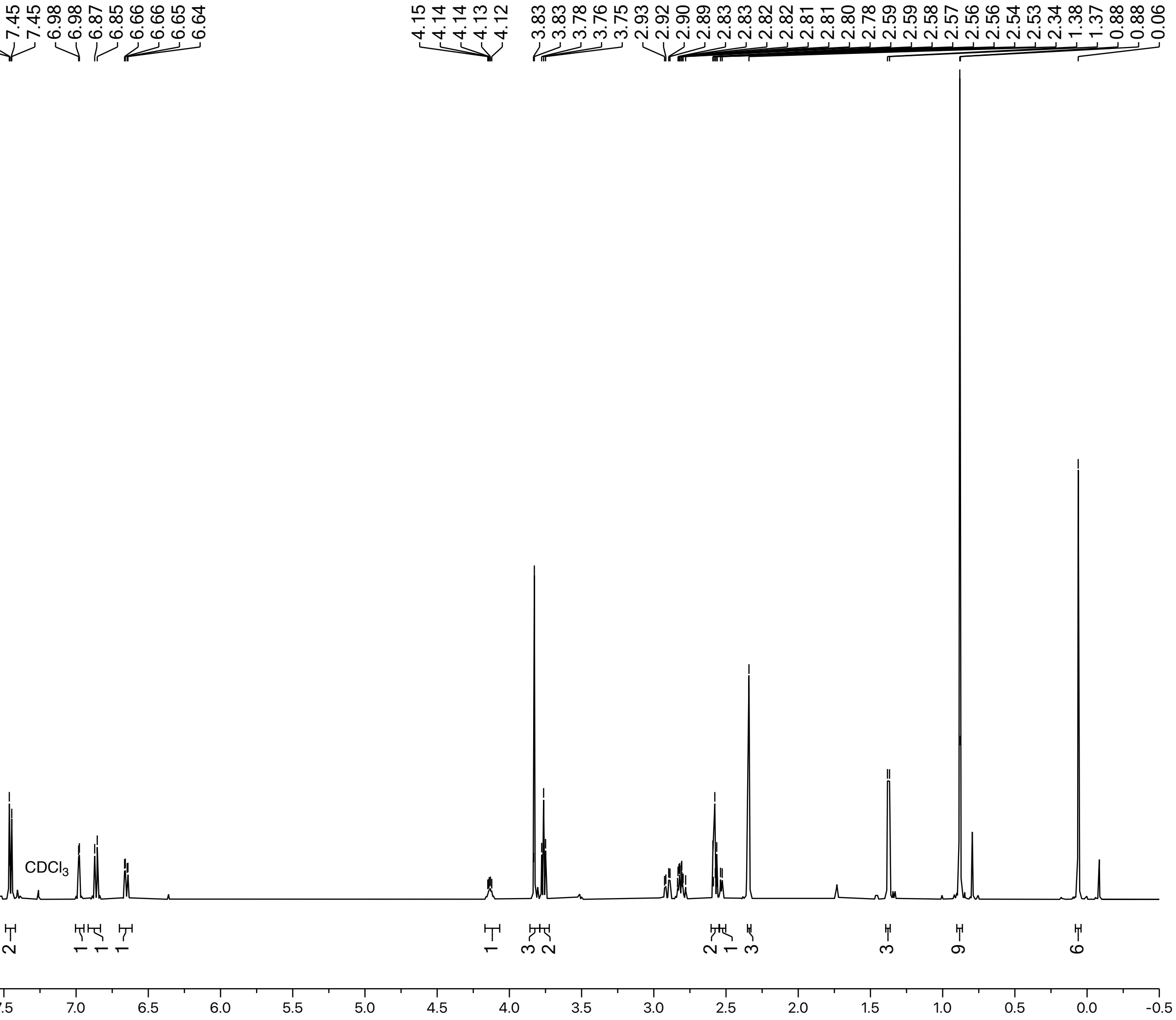

$^{13}C\{^{1}H\}$ NMR, 126 MHz, $CDCl_3$, **17**

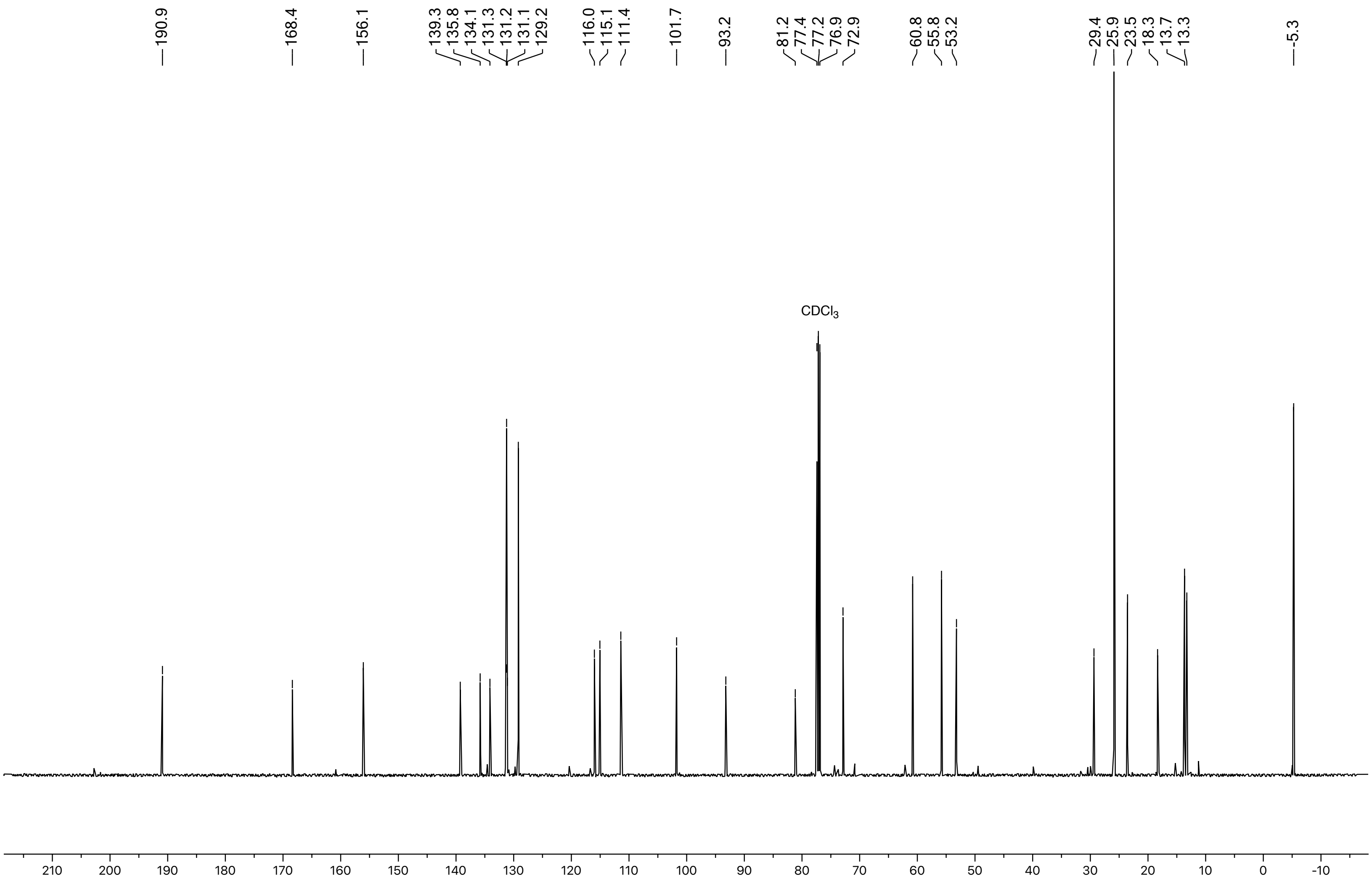

^1H NMR, 500 MHz, $CDCl_3$, **3g**

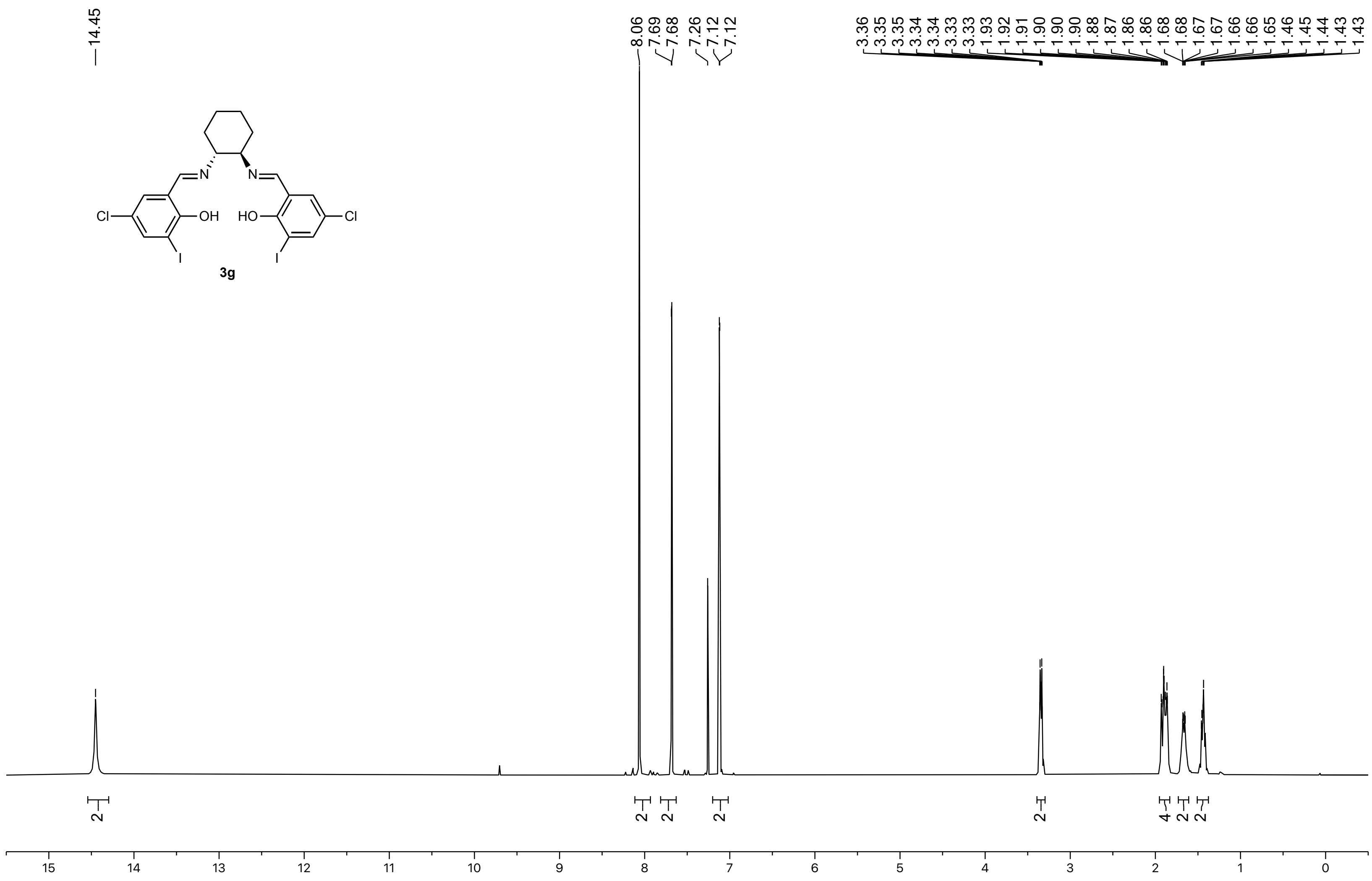

$^{13}C\{^{1}H\}$ NMR, 126 MHz, $CDCl_3$, **3g**

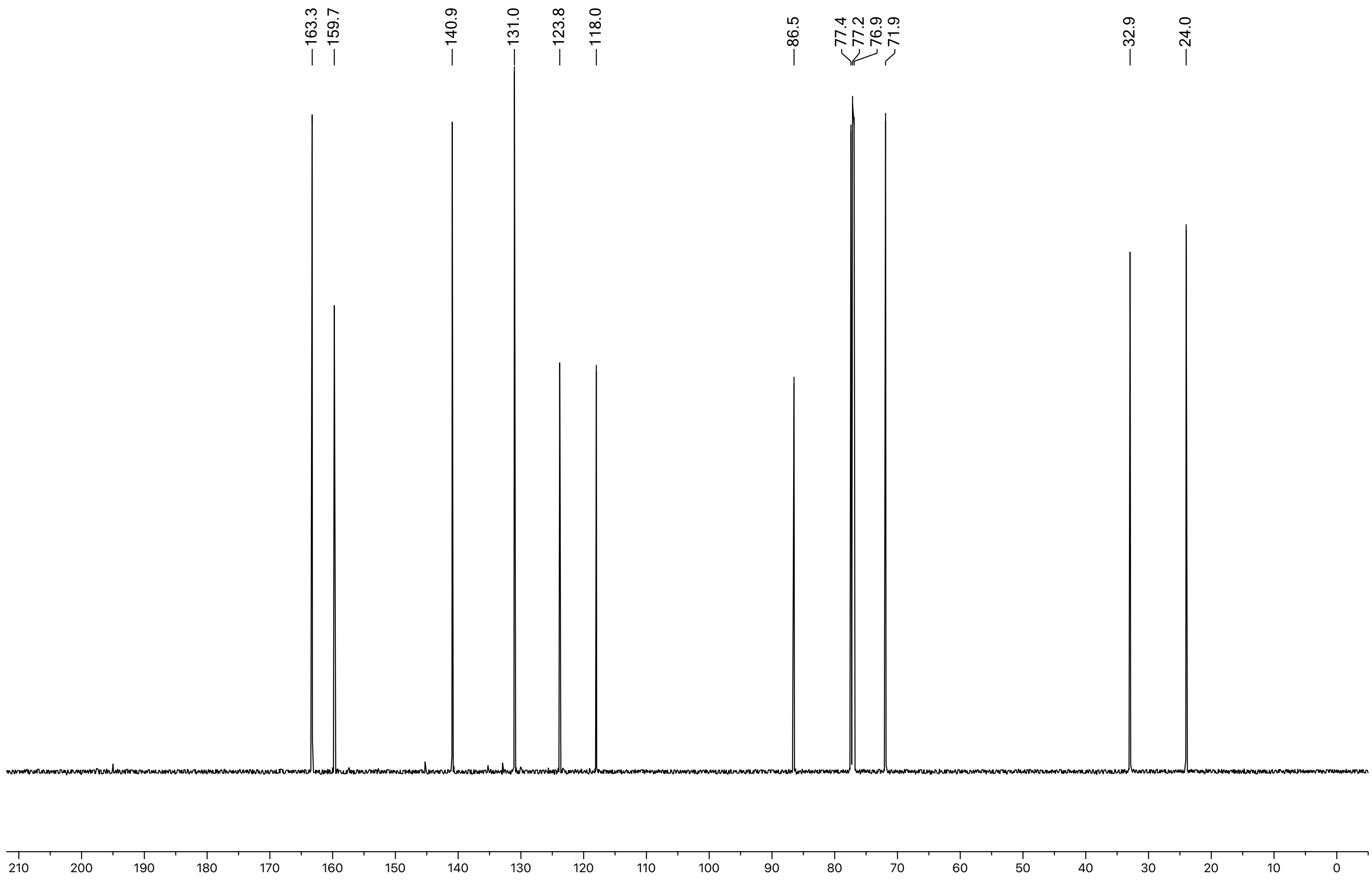

^1H NMR, 500 MHz, $CDCl_3$, **3n**

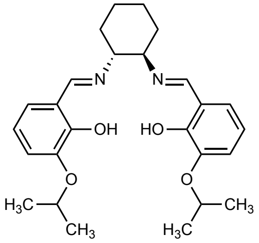

3n

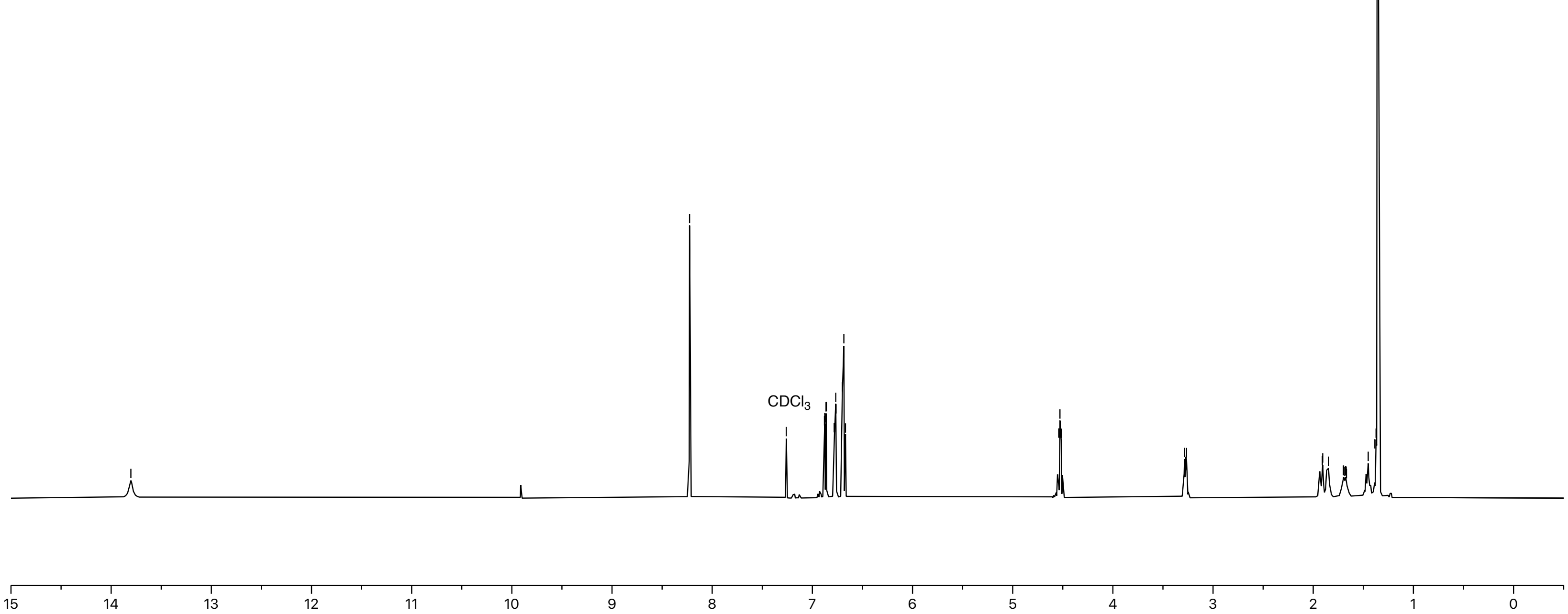

$^{13}C\{^{1}H\}$ NMR, 126 MHz, $CDCl_3$, **3n**

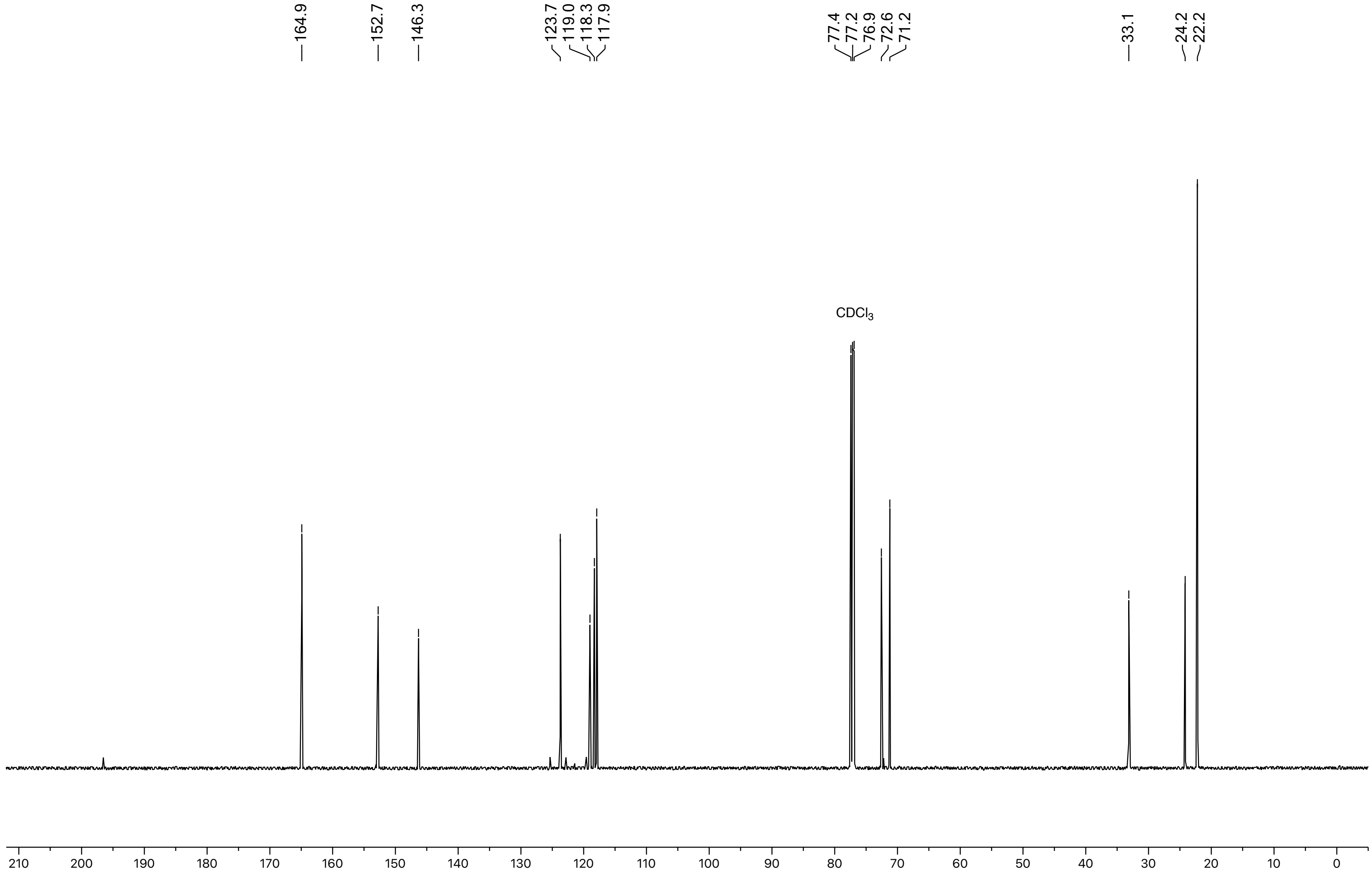

^1H NMR, 500 MHz, $CDCl_3$, **3r**

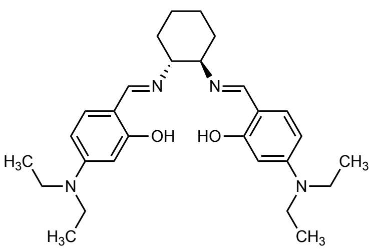

3r

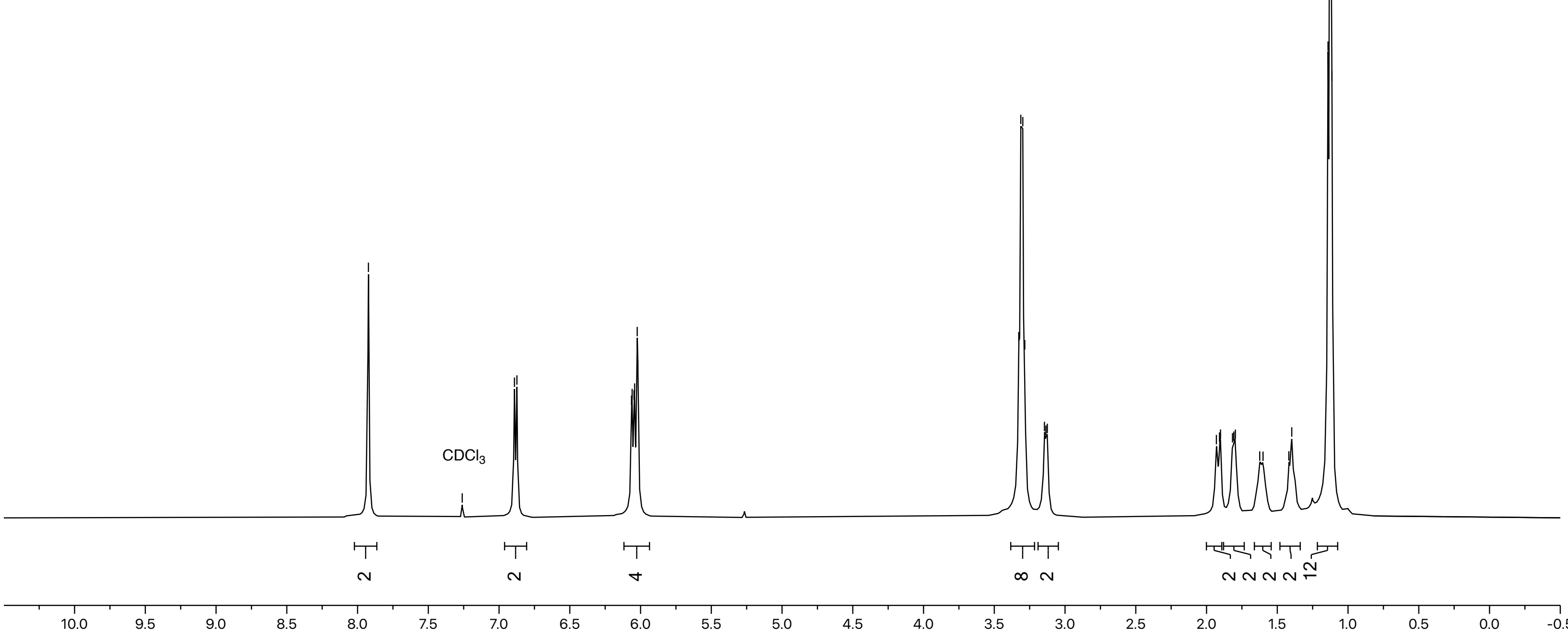

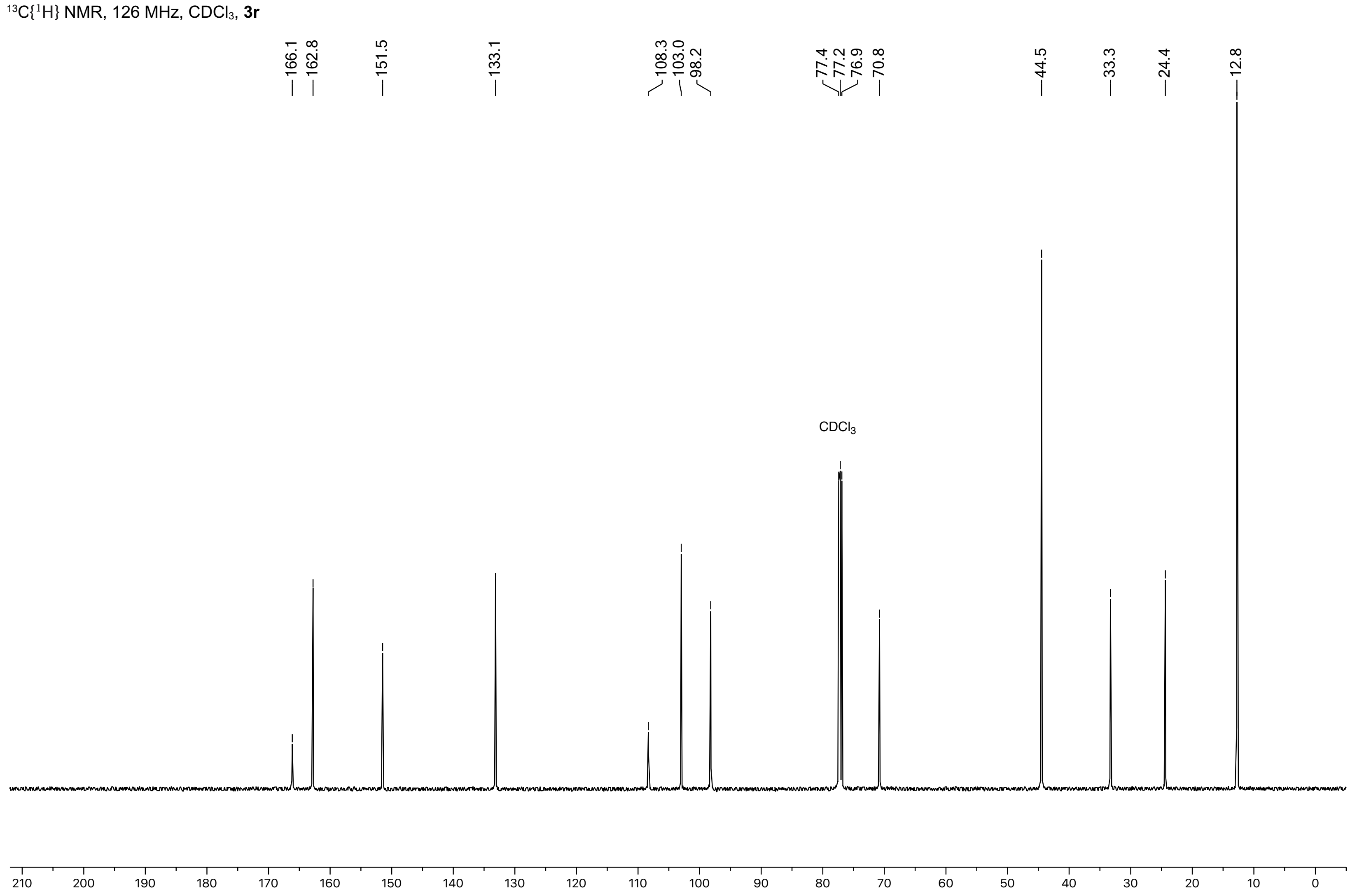

13C{1H} NMR, 126 MHz, CDCl3, 3r
166.1
162.8
151.5
133.1
108.3
103.0
98.2
77.4
77.2
76.9
70.8
44.5
33.3
24.4
12.8
CDCl3
210 200 190 180 170 160 150 140 130 120 110 100 90 80 70 60 50 40 30 20 10 0

^1H NMR, 500 MHz, $CDCl_3$, **4a**

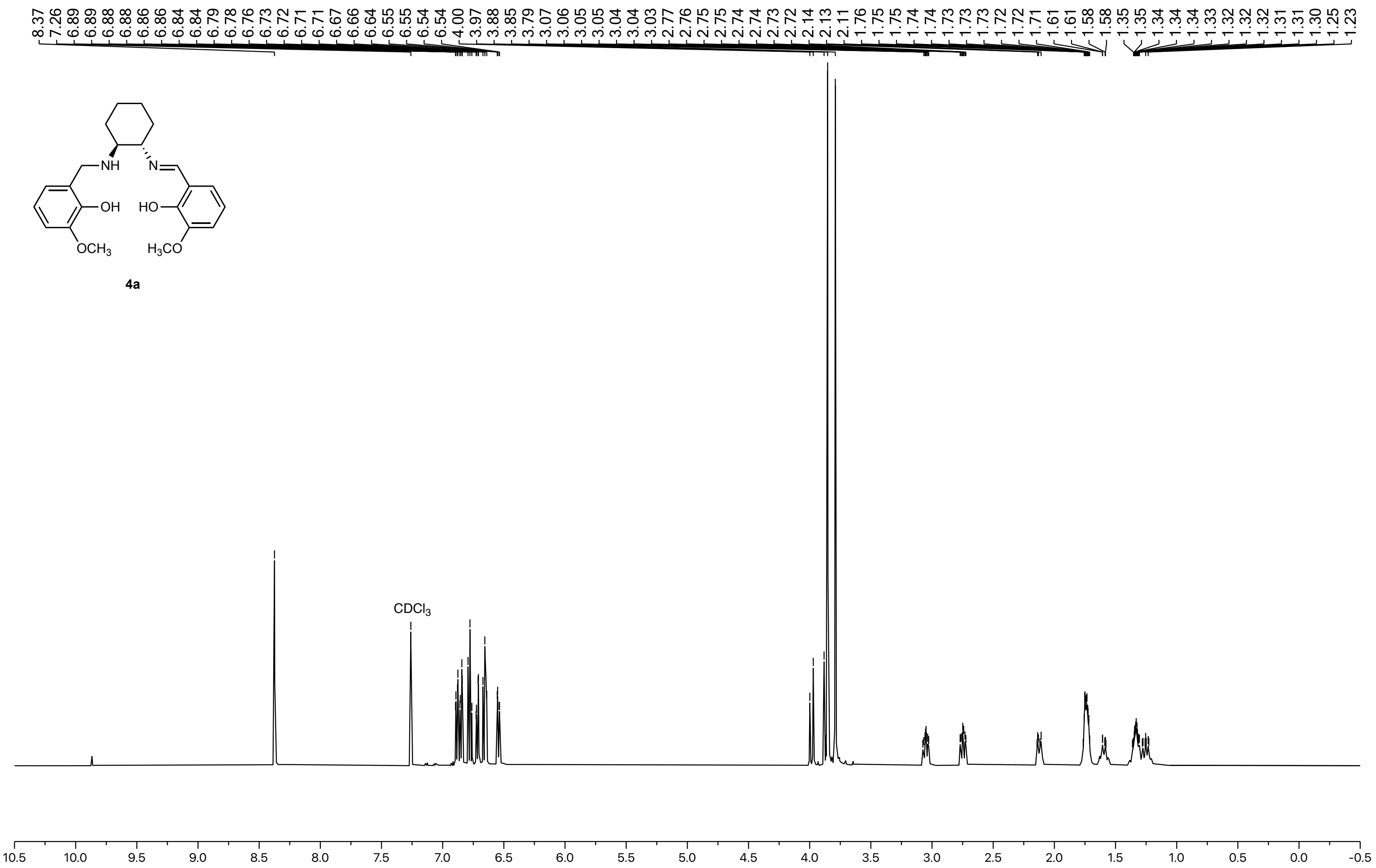

4a

$^{13}C\{^{1}H\}$ NMR, 126 MHz, $CDCl_3$, **4a**

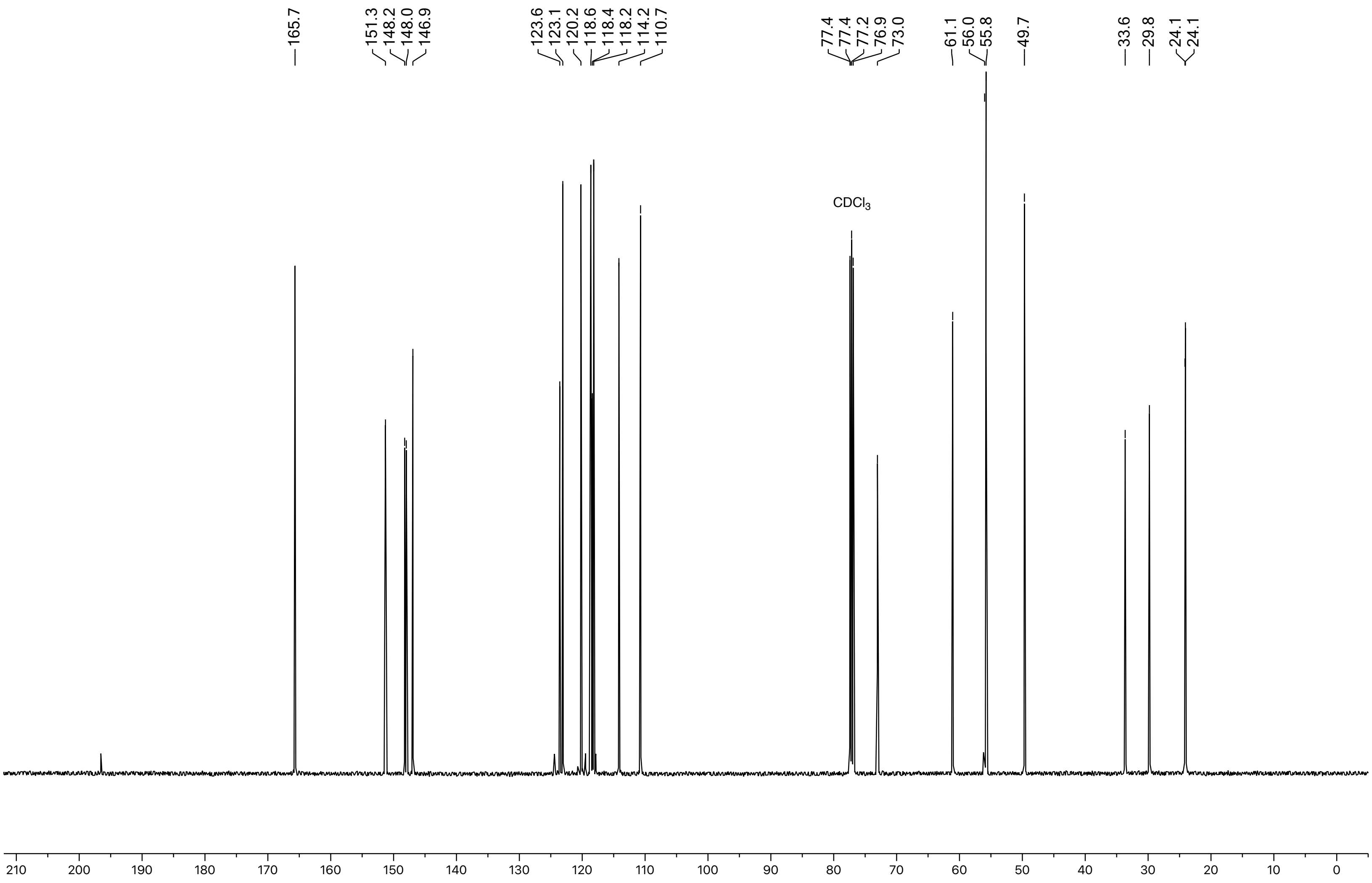

^{1}H NMR, 500 MHz, $CDCl_3$, **S12b**

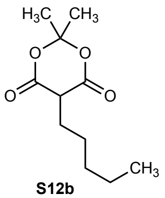

S12b

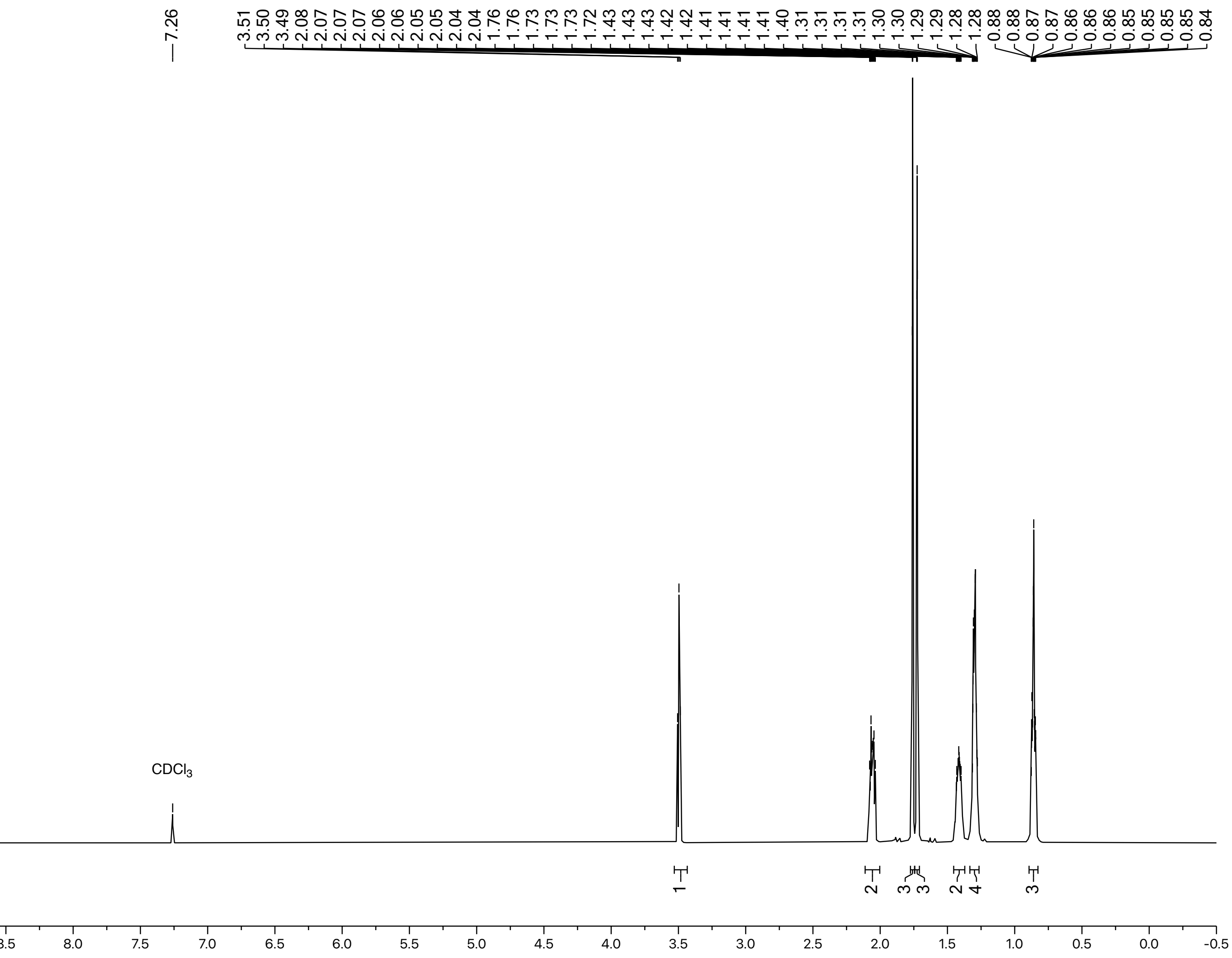

$^{13}C\{^1H\}$ NMR, 126 MHz, $CDCl_3$, **S12b**

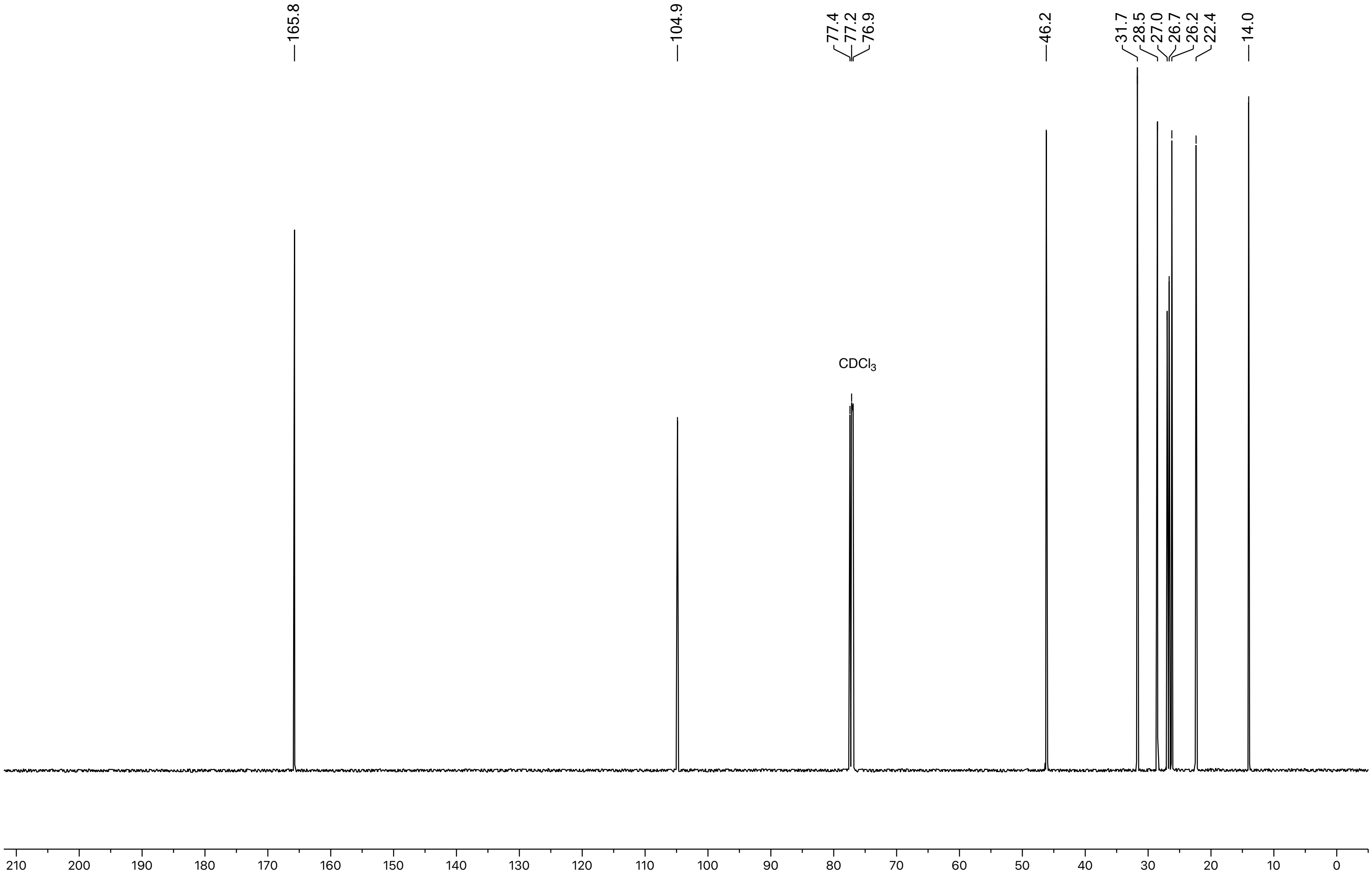

^{1}H NMR, 500 MHz, $CDCl_3$, **S2b**

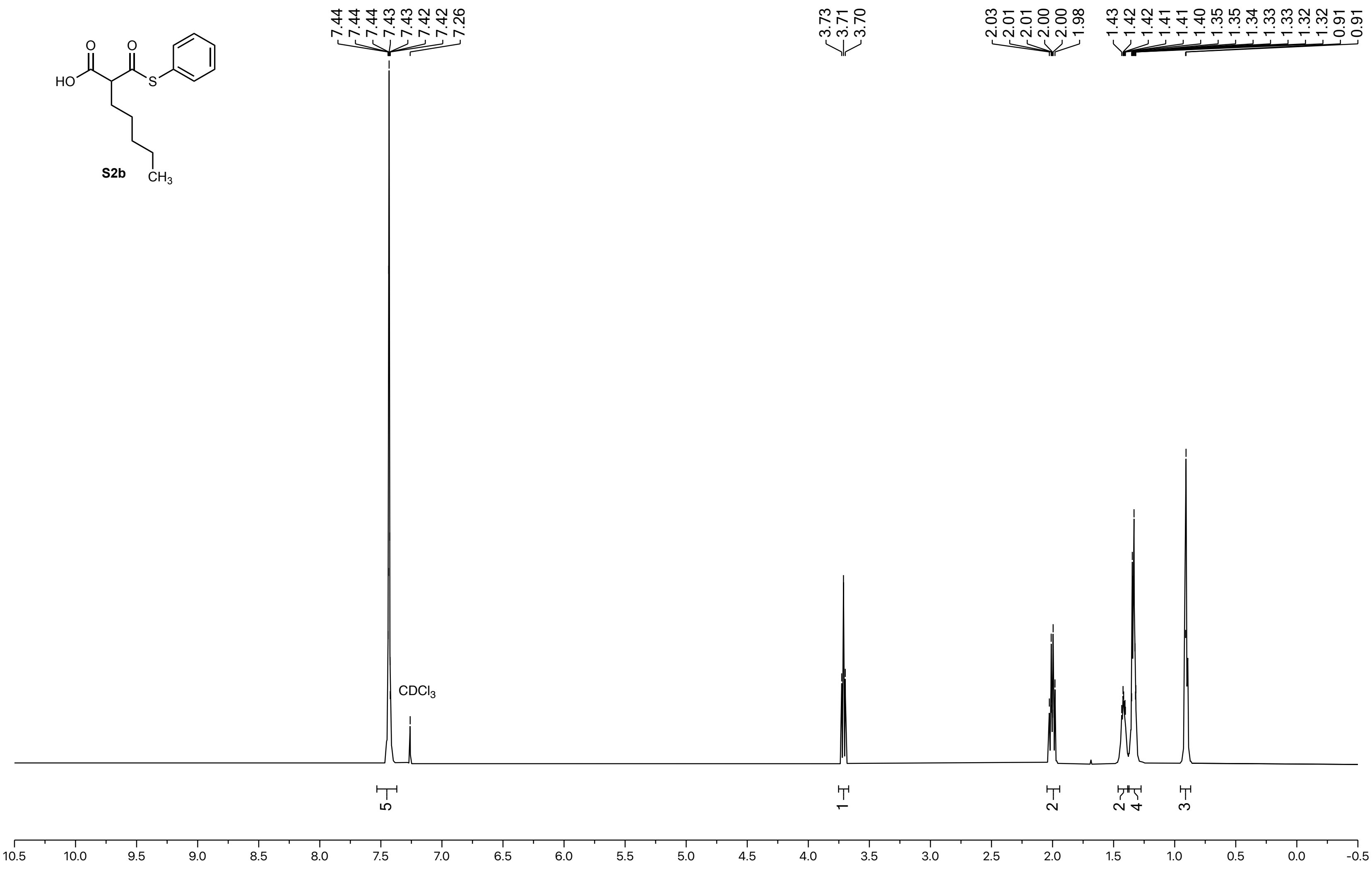

$^{13}C\{^{1}H\}$ NMR, 126 MHz, $CDCl_3$, **S2b**

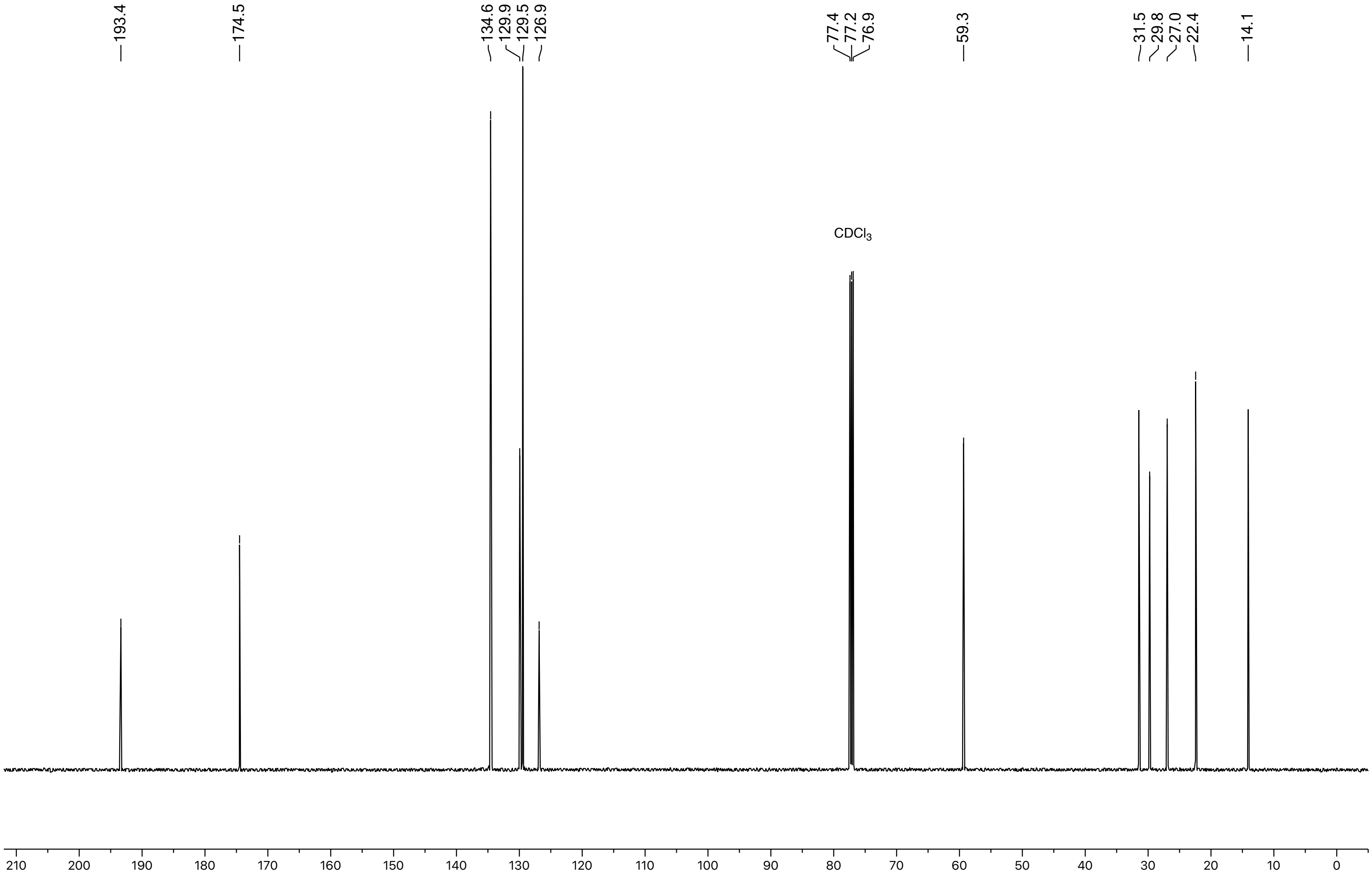

^{1}H NMR, 500 MHz, $CDCl_3$, **S2c**

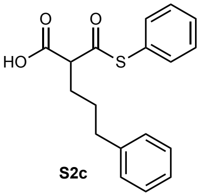

S2c

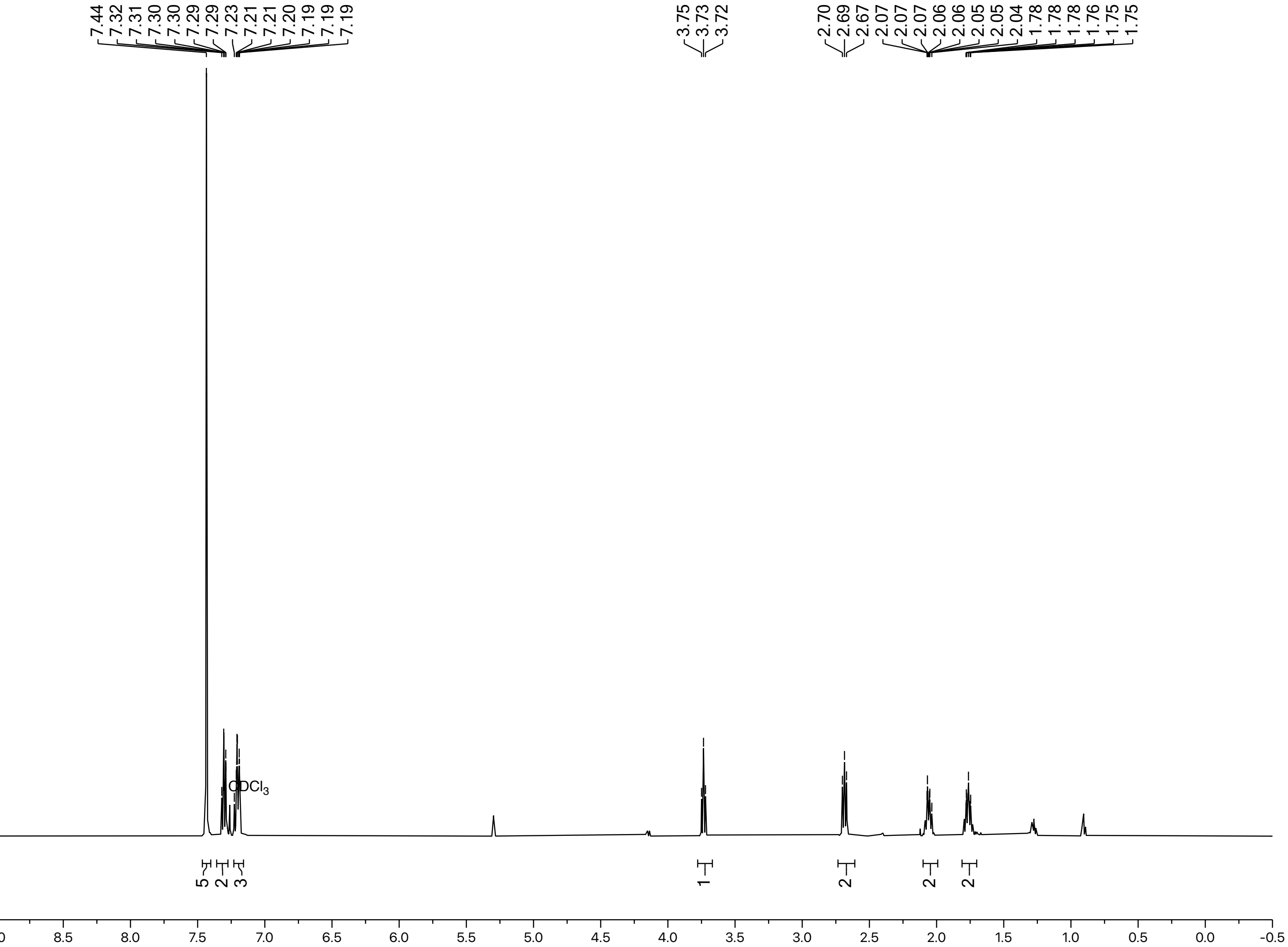

$^{13}C\{^{1}H\}$ NMR, 126 MHz, $CDCl_3$, **S2c**

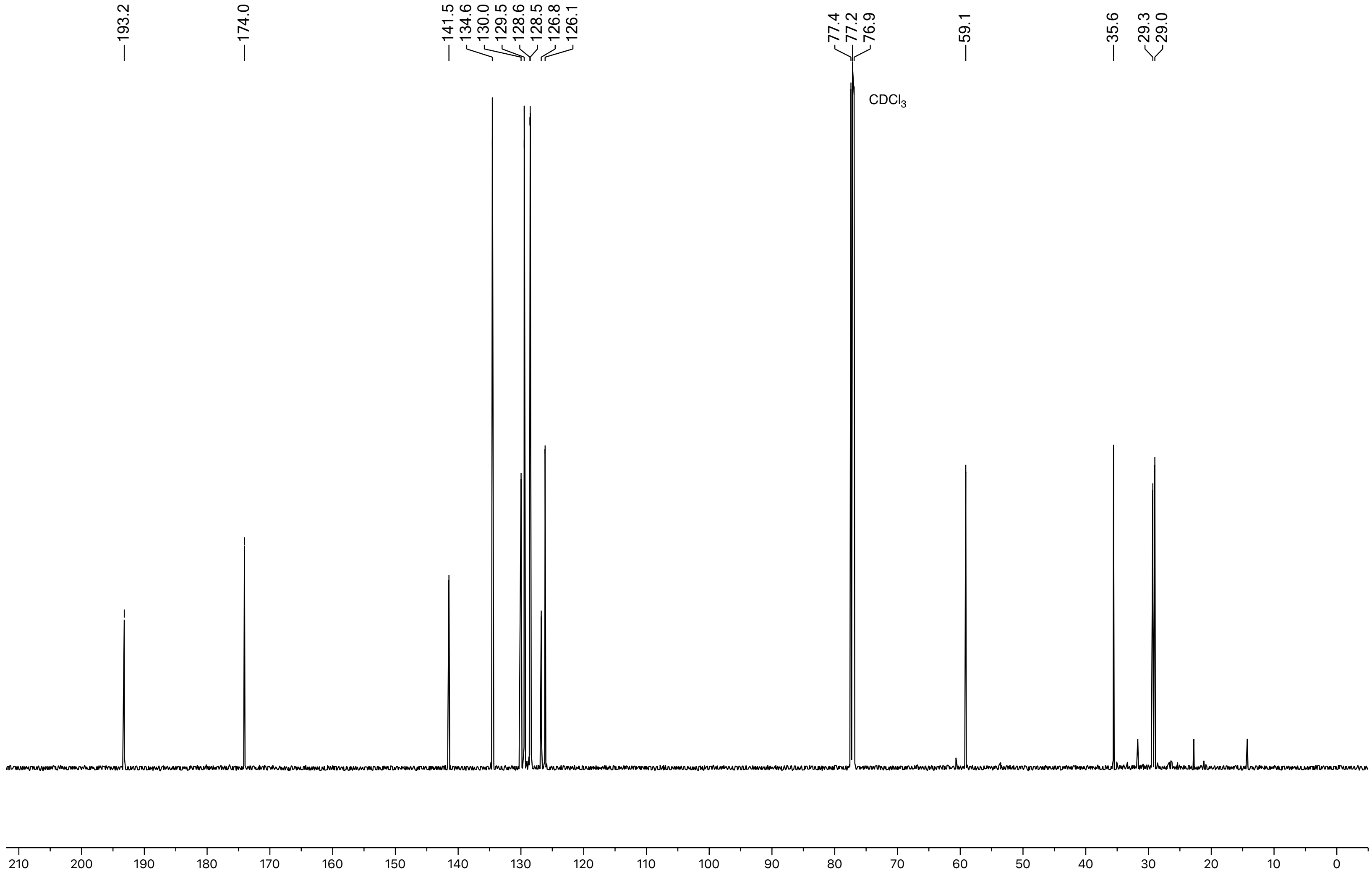

^{1}H NMR, 500 MHz, $CDCl_3$, **S12d**

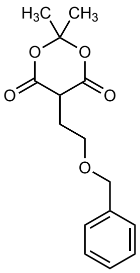

S12d

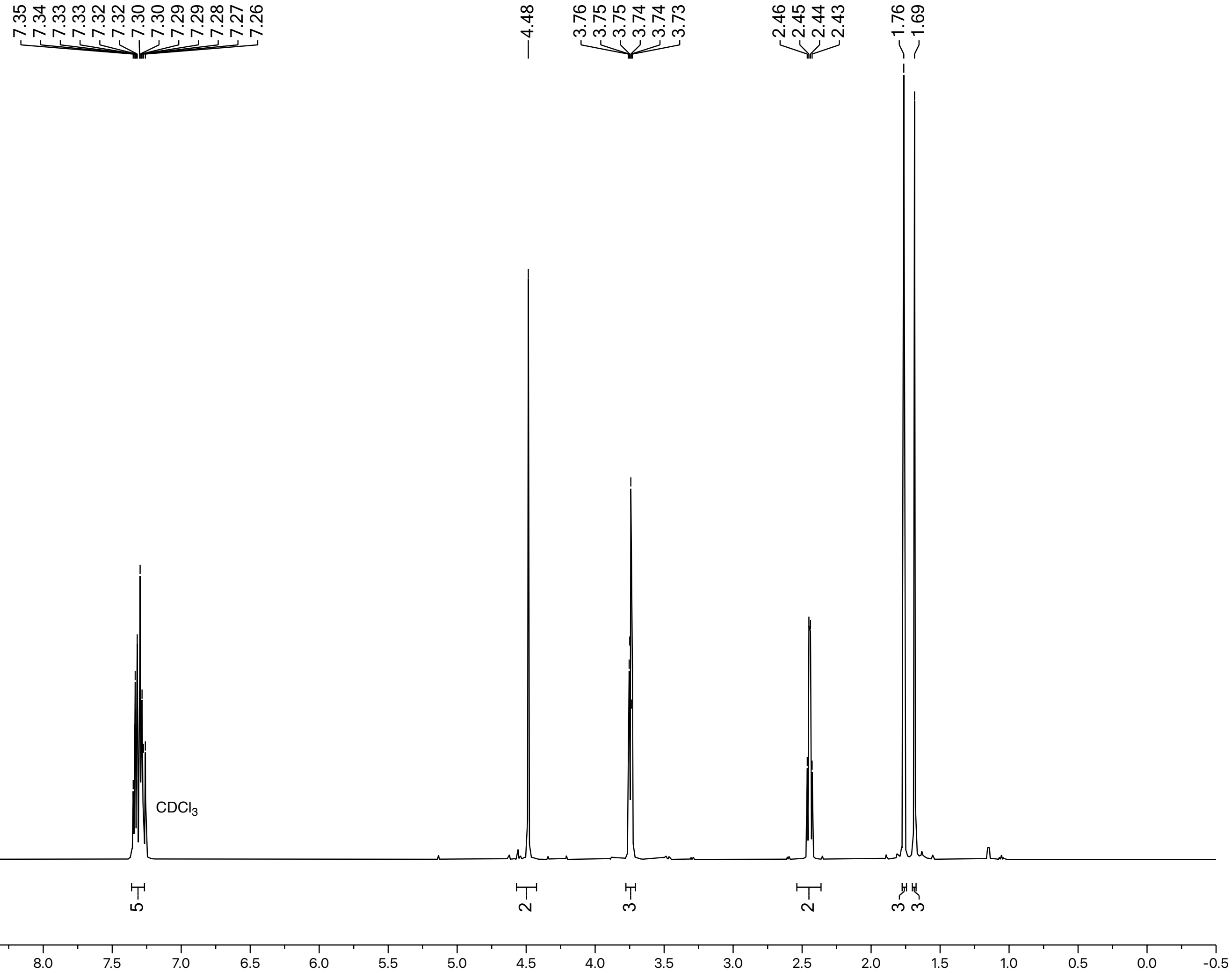

$^{13}C\{^{1}H\}$ NMR, 126 MHz, $CDCl_3$, **S12d**

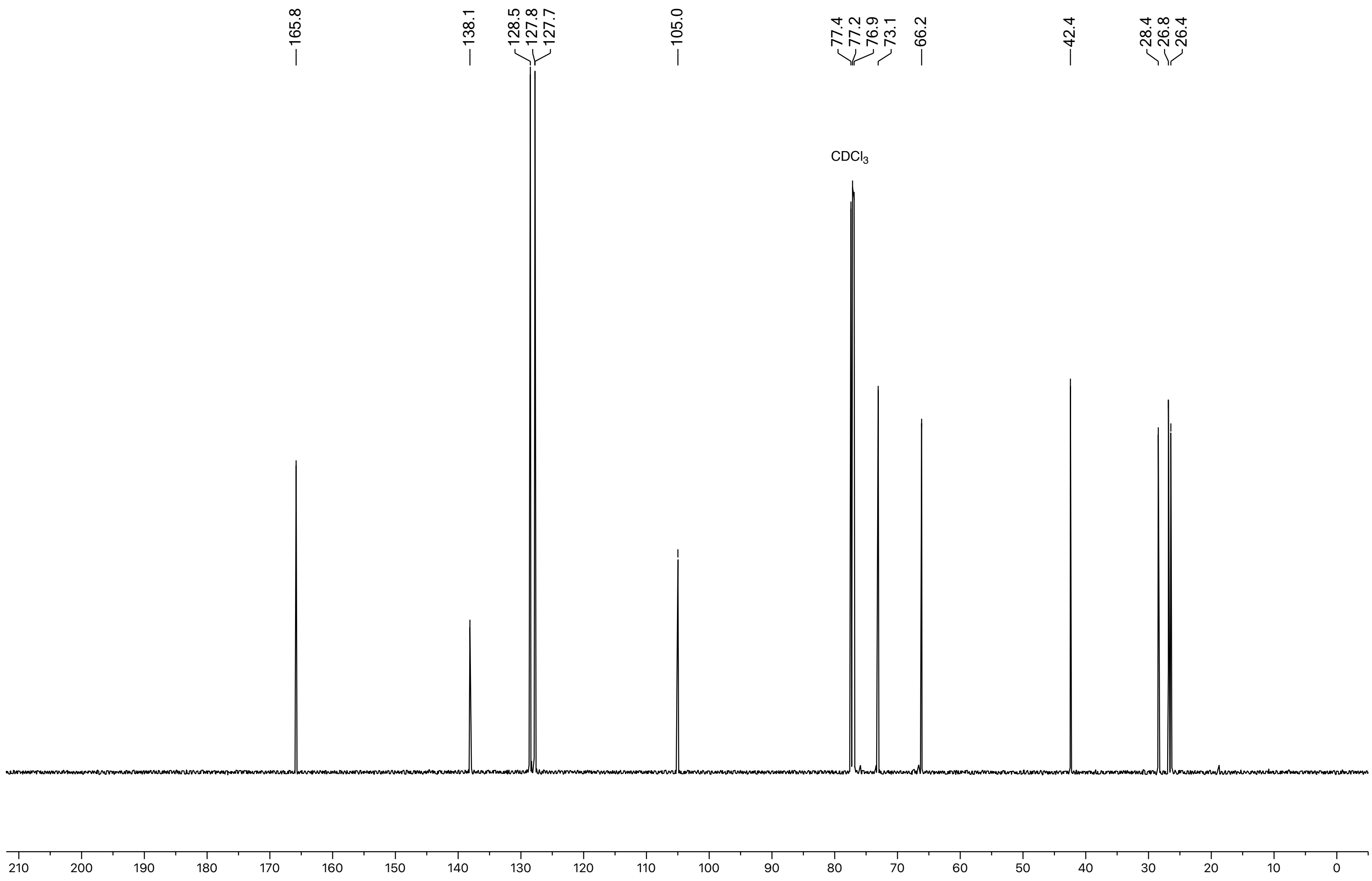

^{1}H NMR, 500 MHz, $CDCl_3$, **S2d**

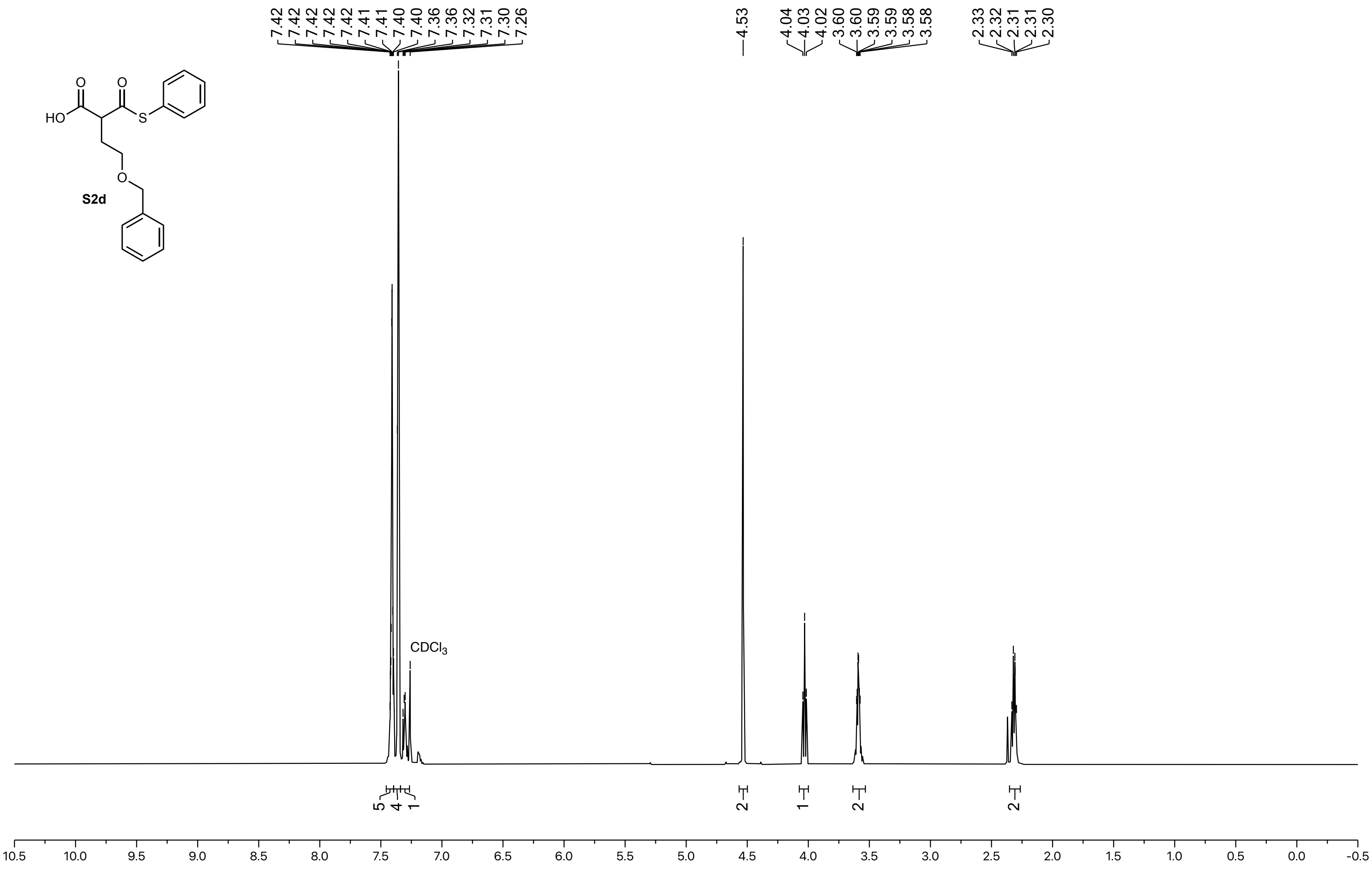

$^{13}C\{^{1}H\}$ NMR, 126 MHz, $CDCl_3$, **S2d**

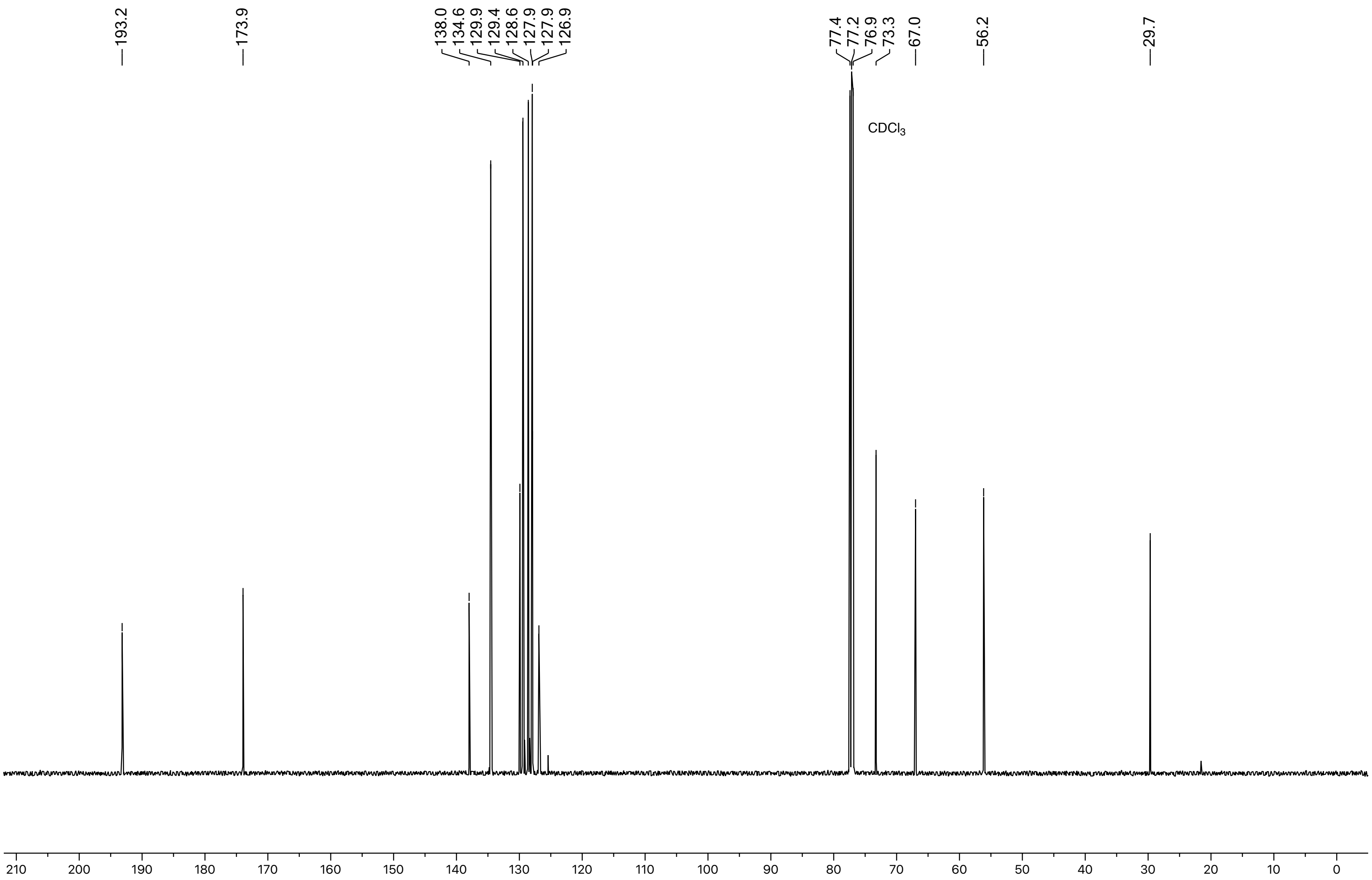

^{1}H NMR, 500 MHz, $CDCl_3$, **S2e**

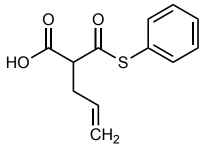

S2e

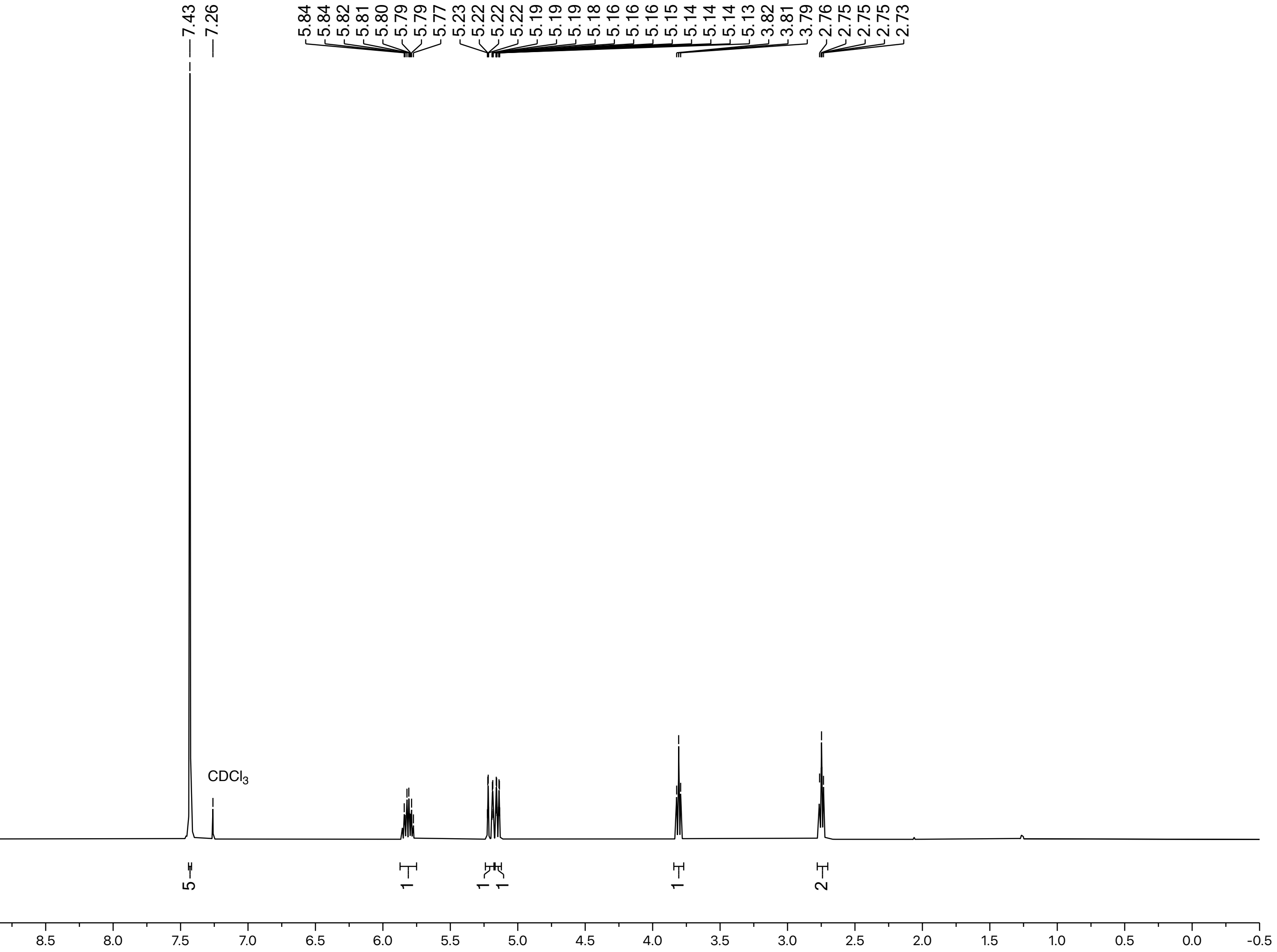

$^{13}C\{^{1}H\}$ NMR, 126 MHz, $CDCl_3$, **S2e**

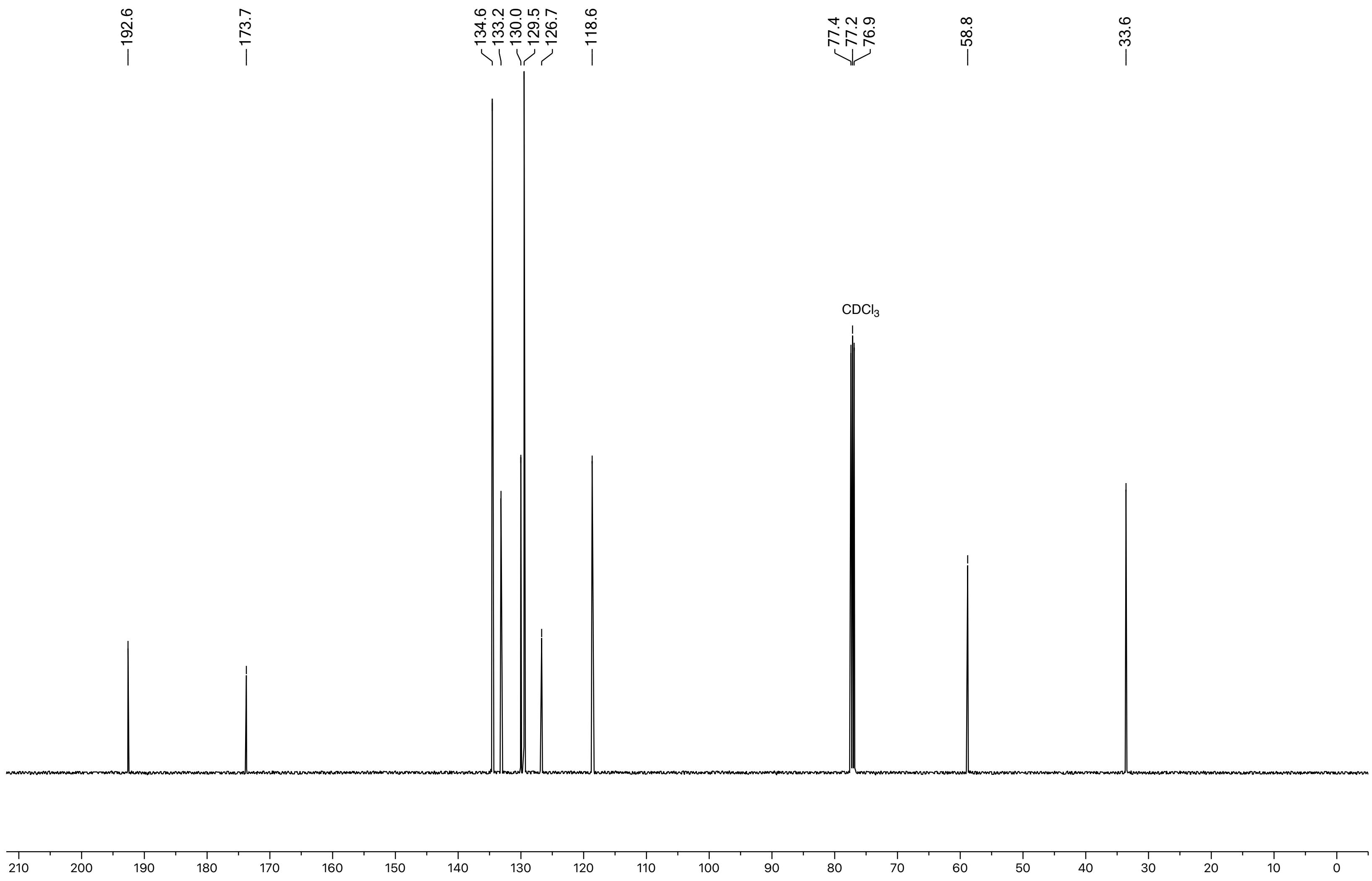

^{1}H NMR, 500 MHz, $CDCl_3$, **S13**

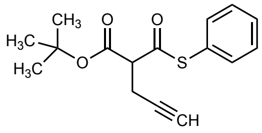

S13

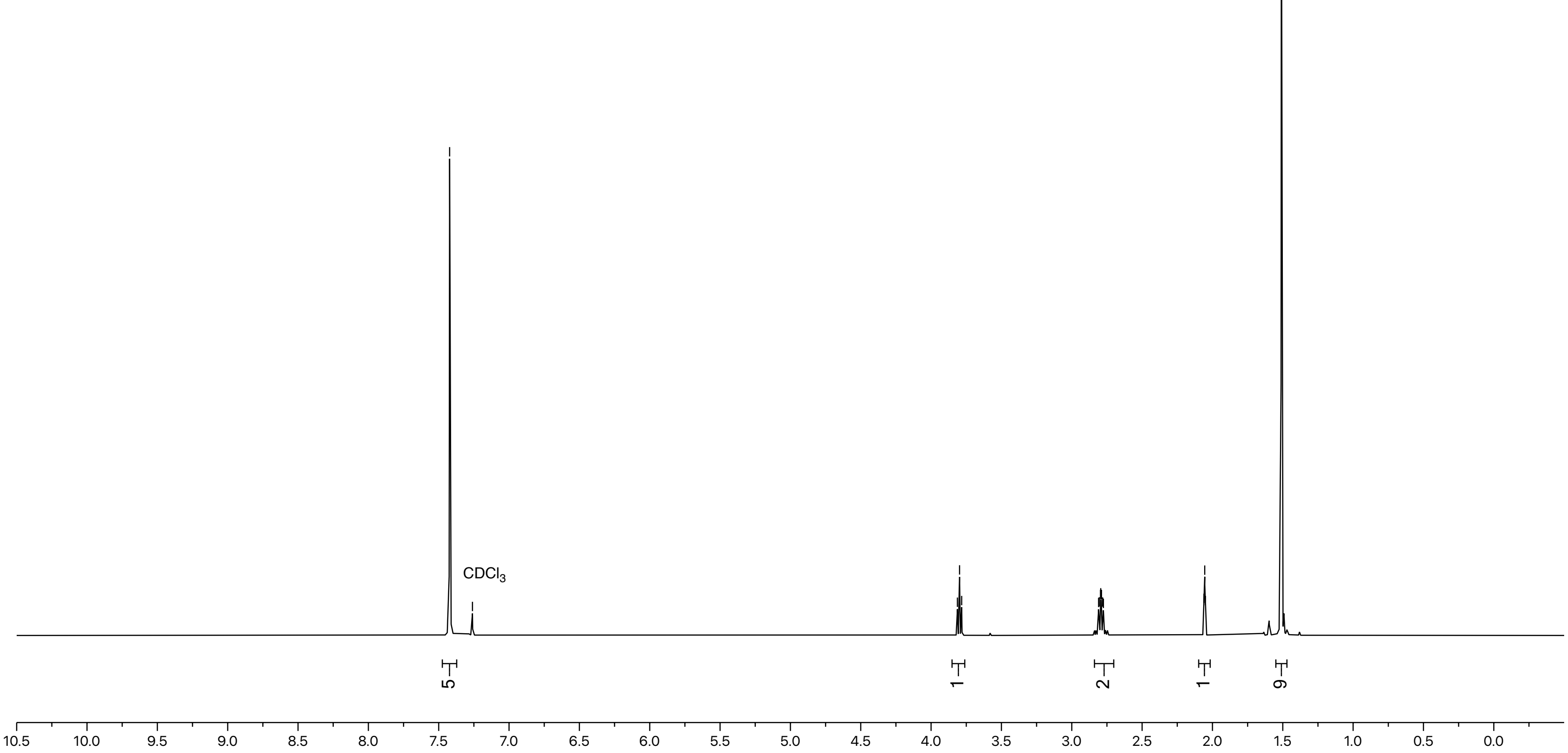

$^{13}C\{^{1}H\}$ NMR, 126 MHz, $CDCl_3$, **S13**

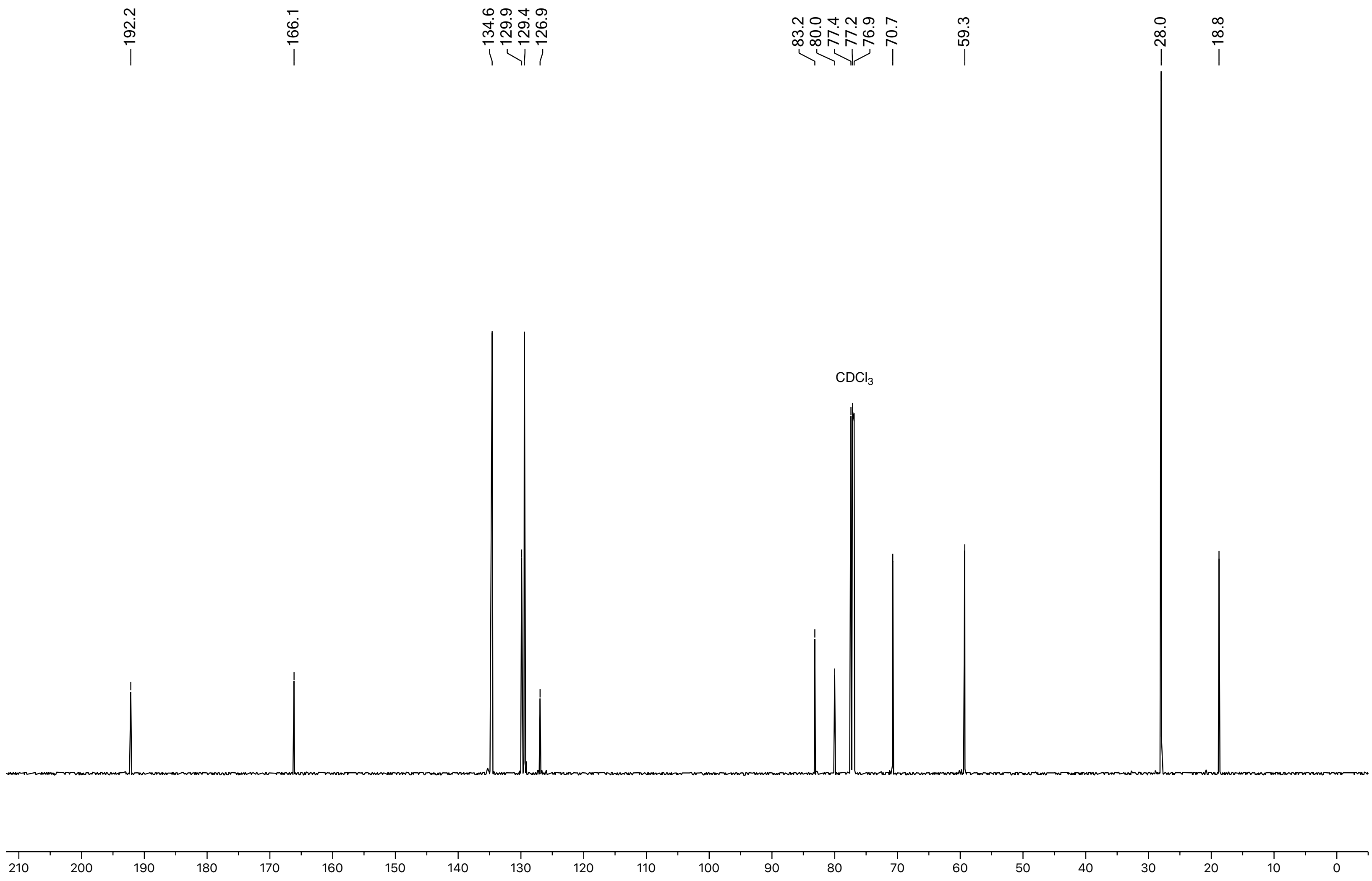

^{1}H NMR, 500 MHz, $CDCl_3$, **S2f**

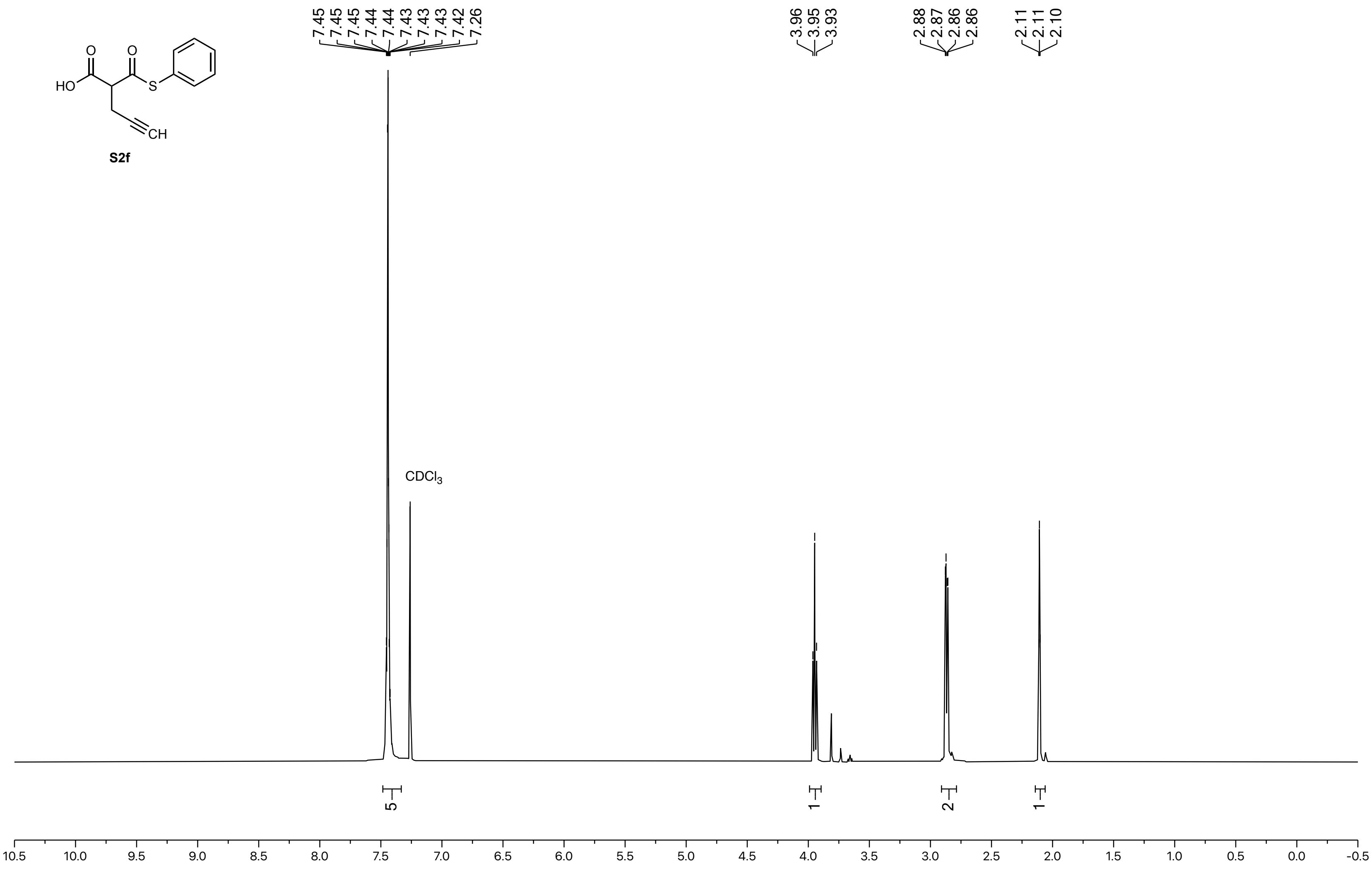

$^{13}C\{^1H\}$ NMR, 126 MHz, $CDCl_3$, **S2f**

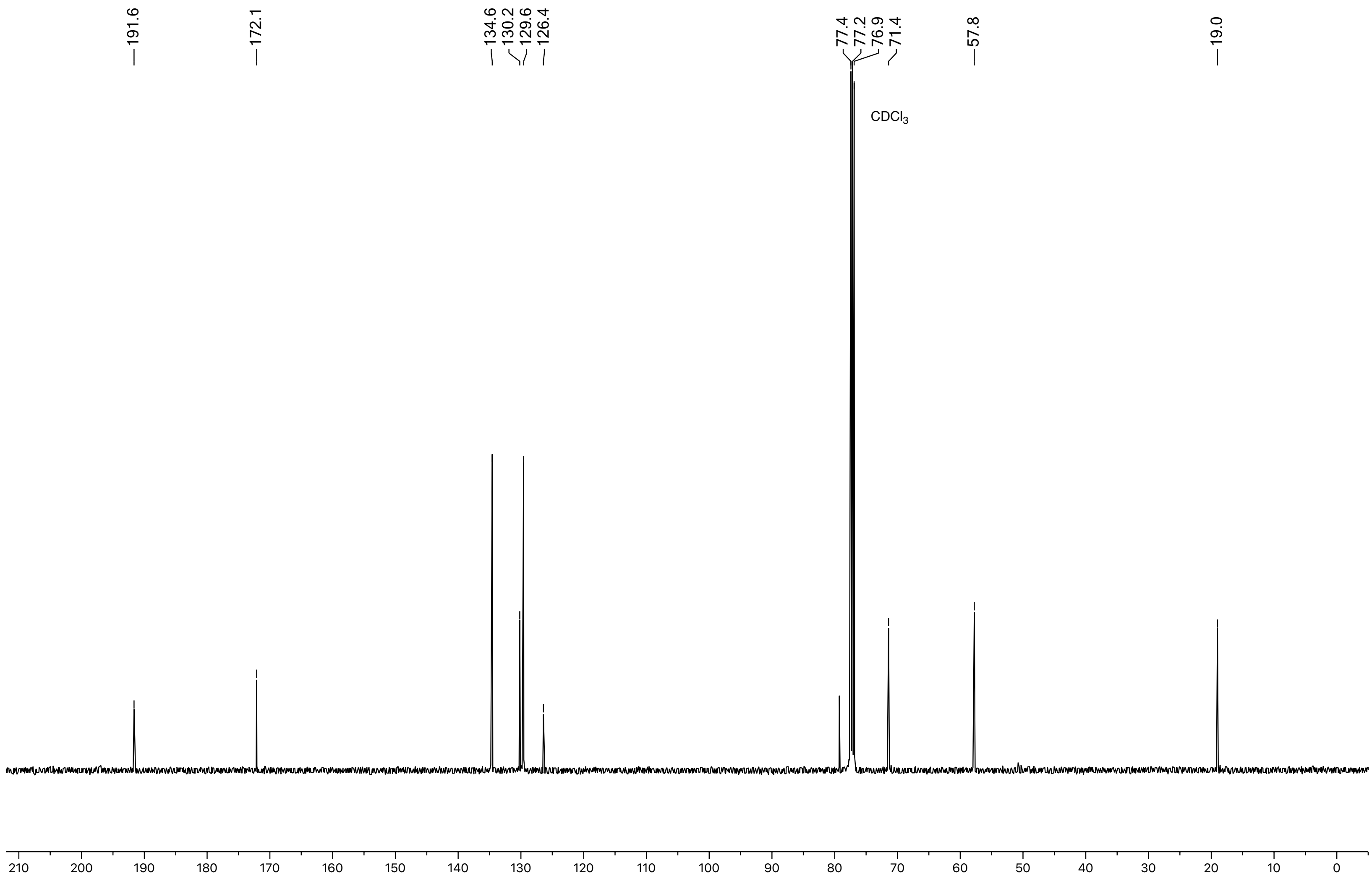

^{1}H NMR, 500 MHz, $CDCl_3$, **S2g**

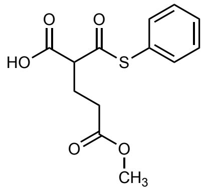

S2g

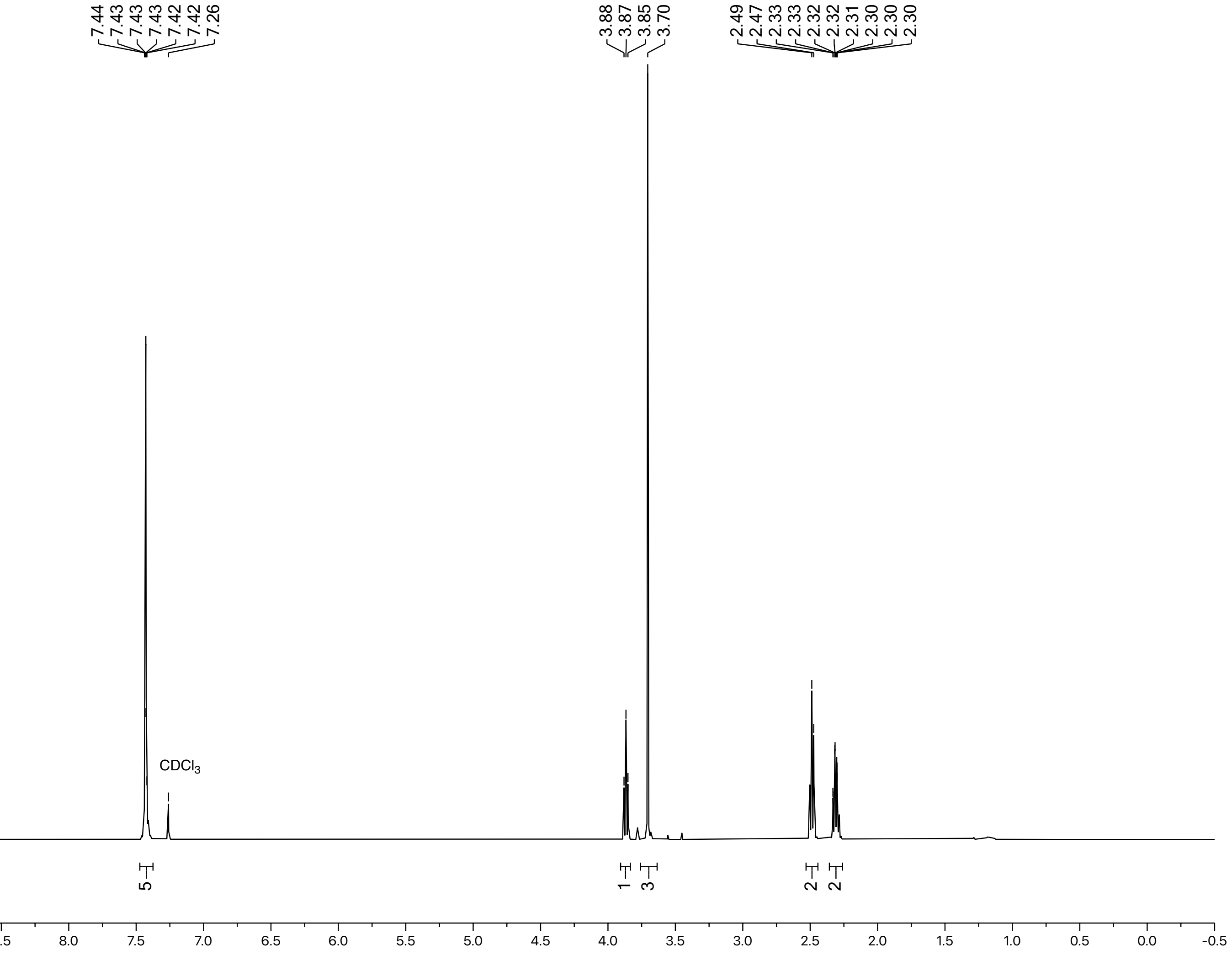

$^{13}C\{^{1}H\}$ NMR, 126 MHz, $CDCl_3$, **S2g**

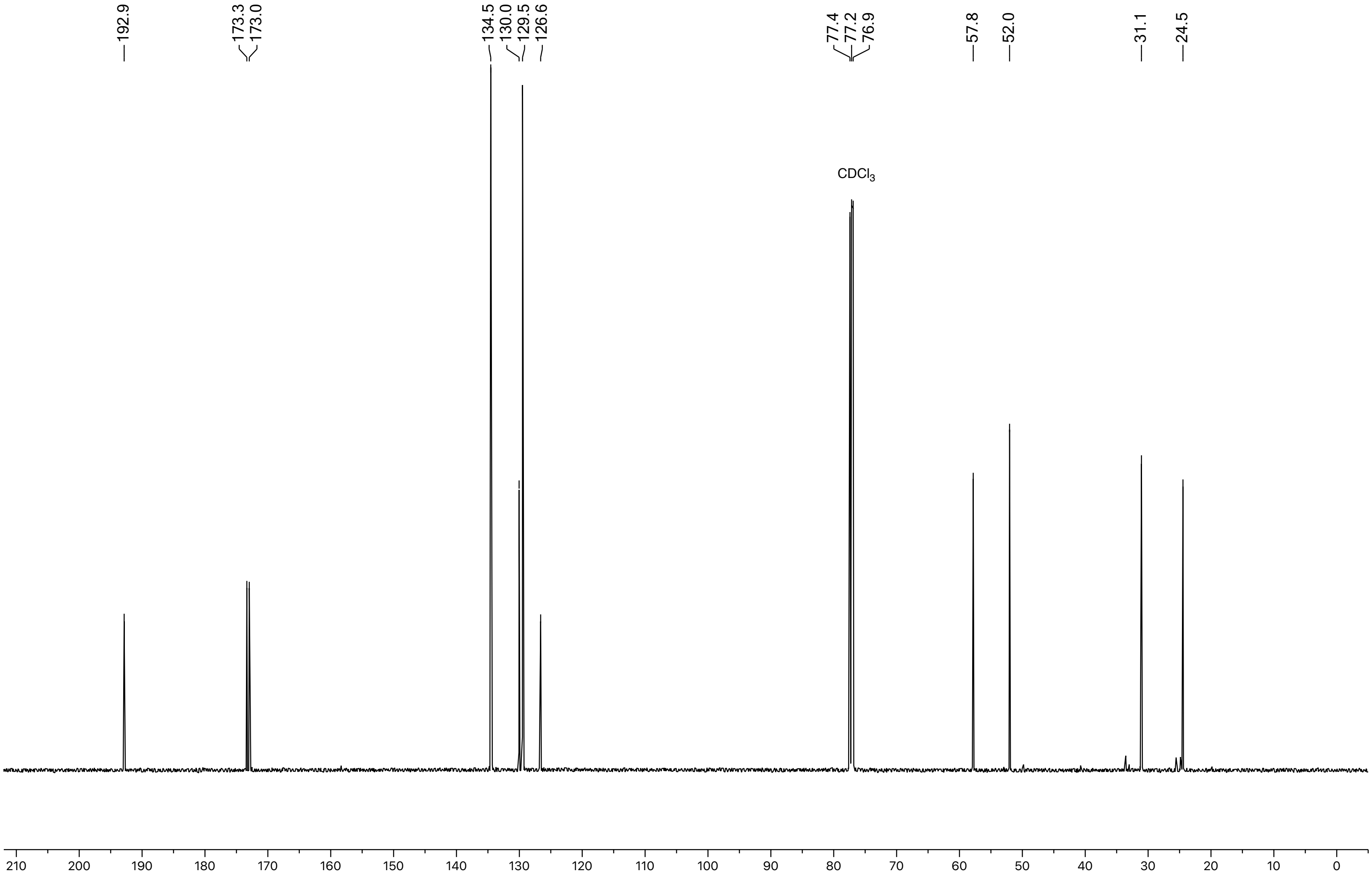

Catalog of X-ray data:

a) Crystal data of (*S*,*S*)-**5a**

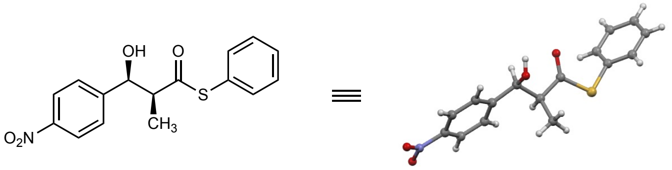

Table 1 Crystal data and structure refinement for (*S*,*S*)-**5a**.

Identification code	(*S*,*S*)-**5a**
Empirical formula	$C_{16}H_{15}NO_4S$
Formula weight	317.35
Temperature/K	296.15
Crystal system	orthorhombic
Space group	$P2_12_12_1$
a/Å	5.3405(2)
b/Å	16.2822(6)
c/Å	17.2211(6)
α/°	90
β/°	90
γ/°	90
Volume/Å^3	1497.46(9)
Z	4
ρ_{calc}g/cm^3	1.408
μ/mm^{-1}	2.085
F(000)	664.0
Crystal size/mm^3	0.3 × 0.25 × 0.2
Radiation	CuKα (λ = 1.54178)
2Θ range for data collection/°	7.472 to 124.958
Index ranges	$-6 \le h \le 6$, $-18 \le k \le 18$, $-19 \le l \le 19$
Reflections collected	24126
Independent reflections	2377 [R_{int} = 0.0450, R_{sigma} = 0.0281]
Data/restraints/parameters	2377/0/201
Goodness-of-fit on F^2	0.919
Final R indexes [I>=2σ (I)]	R_1 = 0.0284, wR_2 = 0.0809
Final R indexes [all data]	R_1 = 0.0287, wR_2 = 0.0811
Largest diff. peak/hole / e Å^{-3}	0.15/-0.12
Flack parameter	0.081(5)

Table 2 Fractional Atomic Coordinates (×10^4) and Equivalent Isotropic Displacement Parameters (Å^2×10^3) for (*S*,*S*)-**5a**. U_{eq} is defined as 1/3 of of the trace of the orthogonalised U_{IJ} tensor.

**Atom**	***x***	***y***	***z***	**U(eq)**
S1	1478.0(13)	2077.9(4)	7192.6(3)	50.8(2)
O1	2220(4)	2372.5(10)	5704.4(9)	49.4(4)
O3	3400(6)	7812.1(12)	6109.2(13)	77.3(7)
C2	5030(5)	6519.6(14)	5879.7(13)	46.7(6)
O2	6856(3)	3541.4(11)	5713.0(11)	53.3(5)
N1	5187(6)	7423.8(14)	5866.8(13)	59.9(7)
O4	7067(6)	7741.0(14)	5618.0(17)	93.6(9)
C14	-3224(6)	589.3(19)	6039.6(17)	60.9(7)
C9	3229(5)	3514.3(13)	6538.4(12)	38.0(5)
C7	4694(4)	4832.5(14)	5883.4(12)	38.8(5)
C8	4452(5)	3899.7(14)	5824.9(13)	39.6(5)
C11	2370(4)	2645.3(14)	6349.4(13)	37.9(5)
C12	68(5)	1199.2(15)	6771.6(14)	42.8(5)
C17	1076(6)	431.9(16)	6914.1(18)	57.0(7)
C13	-2098(5)	1278.4(16)	6338.0(15)	49.0(6)
C4	6828(8)	6070.5(18)	5533(2)	81.8(11)
C16	-62(7)	-253.2(17)	6614(2)	69.7(9)
C5	6624(7)	5223.2(17)	5541(2)	75.5(10)
C3	3105(8)	6164.6(19)	6241(3)	87.9(12)
C15	-2207(7)	-177.8(19)	6178.7(18)	69.9(9)
C6	2951(7)	5311.6(18)	6247(3)	87.2(13)
C10	4882(6)	3561.8(15)	7254.8(15)	53.8(6)

CCDC 2220578 contains the supplementary crystallographic data for this paper, including structure factors and refinement instructions. These data can be obtained free of charge from The Cambridge Crystallographic Data Centre, 12 Union Road.

b) Crystal data of (*R*,*R*)-**5a**

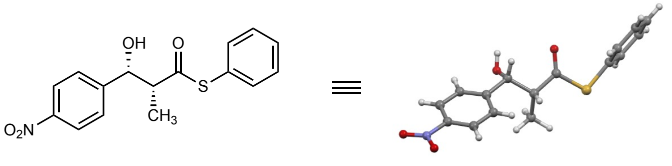

Table 1 Crystal data and structure refinement for (*R*,*R*)-**5a**.

Identification code	(*R*,*R*)-**5a**
Empirical formula	$C_{16}H_{15}NO_4S$
Formula weight	317.35
Temperature/K	296.15
Crystal system	orthorhombic
Space group	$P2_12_12_1$
a/Å	5.3835(6)
b/Å	16.2616(18)
c/Å	17.354(2)
α/°	90
β/°	90
γ/°	90
Volume/Å^3	1519.3(3)
Z	4
ρ_{calc}g/cm^3	1.387
μ/mm^{-1}	2.056
F(000)	664.0
Crystal size/mm^3	? × ? × ?
Radiation	CuKα (λ = 1.54178)
2Θ range for data collection/°	7.45 to 133.29
Index ranges	$-6 \le h \le 6$, $-19 \le k \le 19$, $-20 \le l \le 20$
Reflections collected	27359
Independent reflections	2656 [R_{int} = 0.0505, R_{sigma} = 0.0289]
Data/restraints/parameters	2656/0/215
Goodness-of-fit on F^2	1.069
Final R indexes [I>=2σ (I)]	R_1 = 0.0379, wR_2 = 0.0964
Final R indexes [all data]	R_1 = 0.0391, wR_2 = 0.0973
Largest diff. peak/hole / e Å^{-3}	0.15/-0.16
Flack parameter	0.073(6)

Table 2 Fractional Atomic Coordinates (×10^4) and Equivalent Isotropic Displacement Parameters (Å^2×10^3) for (*R*,*R*)-**5a**. U_{eq} is defined as 1/3 of of the trace of the orthogonalised U_{IJ} tensor.

**Atom**	***x***	***y***	***z***	**U(eq)**
S1	1444.1(19)	7913.7(5)	7183.5(5)	68.1(3)
O2	6773(5)	6463.6(14)	5702.9(16)	71.3(7)
O4	3506(9)	2182.4(16)	6112.0(18)	105.1(11)
C4	61(6)	8797.5(18)	6768.2(18)	56.7(7)
O1	2198(5)	7625.8(13)	5709.7(12)	67.0(6)
C10	4404(6)	6098.8(17)	5822.4(17)	52.0(7)
C7	2344(6)	7349.6(17)	6347.5(16)	49.1(7)
C15	3139(10)	3829(2)	6236(3)	97.7(15)
C11	4672(6)	5167.6(17)	5879.1(15)	49.3(6)
C14	5065(7)	3481.3(18)	5880.7(17)	61.6(8)
C1	-2140(11)	10182(3)	6190(3)	94.4(15)
O3	7133(9)	2268.1(19)	5624(2)	127.7(15)
C5	-2078(6)	8728(2)	6343(2)	66.0(9)
C6	-3176(8)	9420(3)	6051(2)	83.0(11)
C8	3214(6)	6483.4(16)	6531.2(15)	48.7(6)
C9	4869(10)	6435(2)	7240(2)	72.2(10)
N1	5262(9)	2575.9(19)	5868.8(17)	80.0(10)
C3	1096(8)	9557(2)	6911(2)	73.4(10)
C13	6819(10)	3935(2)	5535(3)	95.9(14)
C16	2950(9)	4681(2)	6237(3)	96.9(15)
C12	6595(9)	4784(2)	5541(3)	86.2(12)
C2	-13(11)	10248(2)	6617(3)	93.6(14)

CCDC 2220581 contains the supplementary crystallographic data for this paper, including structure factors and refinement instructions. These data can be obtained free of charge from The Cambridge Crystallographic Data Centre, 12 Union Road.

c) Crystal data of (*S*,*R*)-**6a**

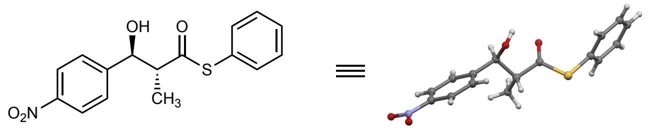

Table 1 Crystal data and structure refinement for (*S*,*R*)-**6a**.

Identification code	(*S*,*R*)-**6a**
Empirical formula	$C_{16}H_{15}NO_4S$
Formula weight	317.35
Temperature/K	100
Crystal system	orthorhombic
Space group	$P2_12_12_1$
a/Å	6.0689(5)
b/Å	15.5995(13)
c/Å	16.0150(13)
α/°	90
β/°	90
γ/°	90
Volume/Å^3	1516.2(2)
Z	4
ρ_{calc}g/cm^3	1.390
μ/mm^{-1}	2.060
F(000)	664.0
Crystal size/mm^3	0.3 × 0.05 × 0.05
Radiation	CuKα (λ = 1.54178)
2Θ range for data collection/°	7.912 to 132.348
Index ranges	$-6 \leq h \leq 6$, $-18 \leq k \leq 18$, $-18 \leq l \leq 18$
Reflections collected	37852
Independent reflections	2619 [R_{int} = 0.0863, R_{sigma} = 0.0395]
Data/restraints/parameters	2619/0/201
Goodness-of-fit on F^2	1.191
Final R indexes [I>=2σ (I)]	R_1 = 0.0402, wR_2 = 0.1269
Final R indexes [all data]	R_1 = 0.0403, wR_2 = 0.1270
Largest diff. peak/hole / e Å^{-3}	0.30/-0.27
Flack parameter	0.053(8)

Table 2 Fractional Atomic Coordinates (×10^4) and Equivalent Isotropic Displacement Parameters (Å^2×10^3) for (*S*,*R*)-**6a**. U_{eq} is defined as 1/3 of of the trace of the orthogonalised U_{IJ} tensor.

**Atom**	***x***	***y***	***z***	**U(eq)**
S001	-1068.1(11)	-7037.8(4)	-2230.5(4)	46.2(3)
C6	1719(5)	-5793.6(19)	-1652(2)	47.9(6)
N1	-5893(5)	-12265.9(17)	-876.2(16)	52.7(6)
C8	-3443(5)	-8274.8(18)	-1409.0(18)	44.2(6)
C4	-1857(6)	-5313(2)	-2080(2)	52.6(7)
C15	-3099(5)	-11155.6(19)	-1139(2)	49.7(7)
O3	-4741(5)	-12781.4(16)	-1234.0(18)	71.4(7)
O2	-579(4)	-8794.9(19)	-506.9(19)	65.6(7)
C9	-5950(6)	-8180(2)	-1548(2)	57.1(8)
C7	-2500(5)	-7376.7(18)	-1321.9(18)	43.5(6)
C11	-3703(4)	-9696.5(16)	-688.4(15)	37.3(6)
O4	-7701(6)	-12437.8(18)	-585(2)	78.5(8)
C13	-6494(5)	-10770.3(18)	-432.6(18)	44.1(6)
C12	-5765(4)	-9935.4(17)	-373.7(16)	40.6(6)
C5	-350(5)	-5967.7(17)	-1963.5(17)	40.1(6)
O1	-2755(6)	-6937.2(18)	-717.5(17)	80.1(10)
C10	-2902(4)	-8783.2(18)	-612.4(18)	40.2(6)
C16	-2398(5)	-10318(2)	-1072.0(19)	50.2(7)
C1	797(8)	-4303(2)	-1560(2)	65.2(10)
C2	2279(6)	-4957(3)	-1456(2)	61.9(9)
C14	-5145(5)	-11369.3(18)	-814.2(16)	41.3(6)
C3	-1276(9)	-4479(2)	-1864(2)	69.9(10)

CCDC 2220579 contains the supplementary crystallographic data for this paper, including structure factors and refinement instructions. These data can be obtained free of charge from The Cambridge Crystallographic Data Centre, 12 Union Road.

d) Crystal data of (*R*,*S*)-**6a**

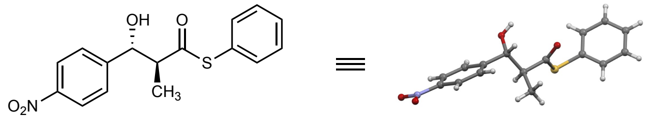

Table 1 Crystal data and structure refinement for (*R*,*S*)-**6a**.

Identification code	(*R*,*S*)-**6a**
Empirical formula	$C_{16}H_{15}NO_4S$
Formula weight	317.35
Temperature/K	100
Crystal system	orthorhombic
Space group	$P2_12_12_1$
a/Å	6.0254(7)
b/Å	15.6161(18)
c/Å	15.7720(18)
α/°	90
β/°	90
γ/°	90
Volume/Å^3	1484.0(3)
Z	4
ρ_{calc}g/cm^3	1.420
μ/mm^{-1}	0.236
F(000)	664.0
Crystal size/mm^3	0.12 × 0.12 × 0.1
Radiation	MoKα (λ = 0.71073)
2Θ range for data collection/°	3.67 to 53.086
Index ranges	$-7 \leq h \leq 7, -19 \leq k \leq 19, -19 \leq l \leq 19$
Reflections collected	50285
Independent reflections	3055 [R_{int} = 0.0581, R_{sigma} = 0.0169]
Data/restraints/parameters	3055/0/201
Goodness-of-fit on F^2	0.841
Final R indexes [I>=2σ (I)]	R_1 = 0.0264, wR_2 = 0.0904
Final R indexes [all data]	R_1 = 0.0271, wR_2 = 0.0910
Largest diff. peak/hole / e Å^{-3}	0.26/-0.30
Flack parameter	0.029(13)

Table 2 Fractional Atomic Coordinates ($\times 10^4$) and Equivalent Isotropic Displacement Parameters ($Å^2 \times 10^3$) for (*R*,*S*)-**6a**. U_{eq} is defined as 1/3 of of the trace of the orthogonalised U_{IJ} tensor.

**Atom**	***x***	***y***	***z***	**U(eq)**
S1	1098.3(8)	2069.4(3)	7240.3(3)	18.71(16)
O1	4830(3)	7819.3(10)	6237.6(11)	28.2(4)
O3	608(2)	3853.8(12)	5484.0(13)	30.0(4)
O4	2686(3)	1977.5(11)	5685.1(11)	36.4(5)
O2	7834(3)	7467.4(12)	5582.1(12)	33.5(4)
N1	6002(3)	7295.6(11)	5883.1(11)	21.4(4)
C11	394(3)	998.9(12)	6973.9(13)	16.2(4)
C12	-1707(3)	814.0(14)	6660.6(14)	19.8(4)
C1	5258(3)	6401.3(12)	5822.0(12)	16.3(4)
C6	6626(3)	5804.6(14)	5433.0(13)	18.1(4)
C8	3481(3)	3303.3(14)	6399.4(13)	19.0(4)
C16	1940(3)	351.7(14)	7091.3(14)	22.5(4)
C4	3778(3)	4736.6(13)	5683.1(12)	16.4(4)
C2	3184(4)	6191.9(14)	6144.7(15)	22.2(4)
C7	2945(3)	3821.5(14)	5600.2(14)	17.3(4)
C5	5872(3)	4964.6(13)	5368.8(13)	17.7(4)
C10	2484(3)	2414.1(14)	6310.4(14)	19.1(4)
C9	6012(3)	3184.6(15)	6533.5(14)	23.2(4)
C3	2455(3)	5353.7(14)	6079.4(14)	20.6(4)
C14	-713(4)	-674.5(15)	6570.1(14)	28.3(5)
C13	-2247(4)	-32.4(17)	6461.2(15)	28.0(5)
C15	1386(5)	-487.9(14)	6878.4(15)	28.5(5)

CCDC 2220583 contains the supplementary crystallographic data for this paper, including structure factors and refinement instructions. These data can be obtained free of charge from The Cambridge Crystallographic Data Centre, 12 Union Road.

e) Crystal data of **6c**

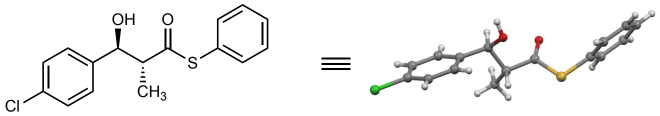

Table 1 Crystal data and structure refinement for **6c**.

Identification code	**6c**
Empirical formula	$C_{16}H_{15}O_2SCl$
Formula weight	306.79
Temperature/K	100(1)
Crystal system	orthorhombic
Space group	$P2_12_12_1$
a/Å	6.0841(16)
b/Å	14.870(4)
c/Å	16.588(5)
α/°	90
β/°	90
γ/°	90
Volume/Å^3	1500.8(7)
Z	4
ρ_{calc}g/cm^3	1.358
μ/mm^{-1}	0.391
F(000)	640.0
Crystal size/mm^3	0.15 × 0.1 × 0.1
Radiation	MoKα (λ = 0.71073)
2Θ range for data collection/°	3.678 to 56.818
Index ranges	$-8 \leq h \leq 8$, $-19 \leq k \leq 19$, $-22 \leq l \leq 22$
Reflections collected	38956
Independent reflections	3762 [R_{int} = 0.1622, R_{sigma} = 0.0928]
Data/restraints/parameters	3762/0/183
Goodness-of-fit on F^2	1.044
Final R indexes [I>=2σ (I)]	R_1 = 0.0558, wR_2 = 0.0909
Final R indexes [all data]	R_1 = 0.0898, wR_2 = 0.0984
Largest diff. peak/hole / e Å^{-3}	0.30/-0.36
Flack parameter	-0.01(5)

Table 2 Fractional Atomic Coordinates (×10^4) and Equivalent Isotropic Displacement Parameters (Å^2×10^3) for **6c**. U_{eq} is defined as 1/3 of of the trace of the orthogonalised U_{IJ} tensor.

**Atom**	***x***	***y***	***z***	**U(eq)**
S1	5124.8(17)	3274.3(6)	2168.1(6)	21.6(2)
Cl1	9305.7(18)	-2406.9(6)	686.1(7)	32.2(3)
O1	4999(4)	1746.6(17)	501.1(16)	21.3(6)
C13	6659(7)	-972(3)	893(2)	20.9(10)
C14	8699(7)	-1262(2)	673(3)	20.1(9)
C11	7754(6)	560(2)	639(2)	15.9(8)
C16	9821(7)	246(2)	404(2)	18.7(9)
C10	7266(6)	1561(2)	650(2)	17.0(8)
C1	4621(7)	4417(2)	1911(2)	17.9(9)
O2	7951(4)	3492.5(17)	983.4(17)	19.8(7)
C8	7715(7)	1974(2)	1477(2)	16.1(9)
C2	6226(7)	5067(3)	2031(3)	20.6(10)
C6	2596(7)	4639(3)	1605(2)	21.3(10)
C15	10298(7)	-662(2)	414(2)	21.4(9)
C9	10100(7)	1885(3)	1760(3)	27.0(10)
C7	7100(6)	2963(2)	1444(2)	14.1(8)
C12	6184(7)	-58(3)	887(2)	18.3(9)
C3	5785(7)	5950(2)	1811(2)	21.2(10)
C5	2154(7)	5527(3)	1391(3)	23.8(10)
C4	3751(7)	6177(3)	1494(3)	22.3(10)

CCDC 2220582 contains the supplementary crystallographic data for this paper, including structure factors and refinement instructions. These data can be obtained free of charge from The Cambridge Crystallographic Data Centre, 12 Union Road.

f) Crystal data of **6f**

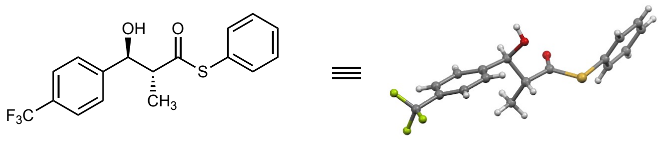

Table 1 Crystal data and structure refinement for **6f**.

Identification code	**6f**
Empirical formula	$C_{17}H_{15}F_3O_2S$
Formula weight	340.35
Temperature/K	100
Crystal system	orthorhombic
Space group	$P2_12_12_1$
a/Å	6.0149(7)
b/Å	15.6930(18)
c/Å	16.9152(19)
α/°	90
β/°	90
γ/°	90
Volume/Å^3	1596.7(3)
Z	4
ρ_{calc}g/cm^3	1.416
μ/mm^{-1}	0.240
F(000)	704.0
Crystal size/mm^3	0.1 × 0.02 × 0.01
Radiation	MoKα (λ = 0.71073)
2Θ range for data collection/°	3.54 to 52.74
Index ranges	$-7 \leq h \leq 7$, $-19 \leq k \leq 19$, $-21 \leq l \leq 21$
Reflections collected	57544
Independent reflections	3262 [R_{int} = 0.1311, R_{sigma} = 0.0378]
Data/restraints/parameters	3262/0/210
Goodness-of-fit on F^2	0.922
Final R indexes [I>=2σ (I)]	R_1 = 0.0390, wR_2 = 0.1116
Final R indexes [all data]	R_1 = 0.0422, wR_2 = 0.1139
Largest diff. peak/hole / e Å^{-3}	0.49/-0.48
Flack parameter	0.06(4)

Table 2 Fractional Atomic Coordinates ($\times10^4$) and Equivalent Isotropic Displacement Parameters ($Å^2\times10^3$) for **6f**. U_{eq} is defined as 1/3 of of the trace of the orthogonalised U_{IJ} tensor.

**Atom**	***x***	***y***	***z***	**U(eq)**
S1	5115.0(12)	8227.5(5)	2892.5(4)	21.2(2)
O2	5189(3)	6772.7(13)	4517.9(13)	19.8(5)
O1	2089(4)	8431.6(13)	4004.2(13)	18.7(4)
C11	2377(5)	5658.1(18)	4367.0(17)	15.1(6)
C10	2900(5)	6605.5(18)	4361.7(17)	14.8(6)
C3	3769(5)	9907.3(19)	2937.3(18)	19.3(6)
C16	1279(5)	3938.6(19)	4267.2(18)	17.1(6)
C14	3894(5)	5073(2)	4070.4(19)	18.0(6)
C4	5453(5)	9326(2)	3099.9(17)	18.6(6)
C13	-242(5)	4516.0(19)	4579.7(17)	19.3(6)
C15	3359(5)	4217.7(19)	4024.5(19)	19.1(6)
C12	312(5)	5372.3(19)	4631.1(18)	17.8(6)
C2	4085(5)	10767(2)	3108.4(19)	21.9(7)
C17	608(5)	3027(2)	4187.1(19)	21.6(6)
C1	6089(6)	11042(2)	3430.6(19)	24.8(7)
C7	3033(5)	7932.4(18)	3573.0(16)	14.3(6)
C5	7454(5)	9599(2)	3418(2)	24.5(7)
C9	2493(5)	6984.7(18)	3537.3(17)	15.1(6)
C6	7776(6)	10460(2)	3577(2)	26.9(7)
C8	103(6)	6872(2)	3244.5(19)	25.6(7)
F1	282(4)	2641.3(12)	4881.1(12)	32.9(5)
F2	2088(4)	2557.4(12)	3795.3(13)	32.3(5)
F3	-1322(4)	2952.2(14)	3791.7(16)	40.3(6)

CCDC 2220580 contains the supplementary crystallographic data for this paper, including structure factors and refinement instructions. These data can be obtained free of charge from The Cambridge Crystallographic Data Centre, 12 Union Road.

g) Crystal data of **5j**

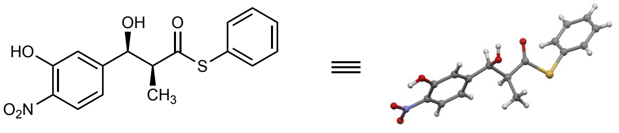

Table 1 Crystal data and structure refinement for **5j**.

Identification code	**5j**
Empirical formula	$C_{16}H_{15}NO_5S$
Formula weight	333.35
Temperature/K	210
Crystal system	orthorhombic
Space group	$P2_12_12_1$
a/Å	5.3714(3)
b/Å	16.5047(10)
c/Å	17.2413(11)
α/°	90
β/°	90
γ/°	90
Volume/Å^3	1528.50(16)
Z	4
ρ_{calc}g/cm^3	1.449
μ/mm^{-1}	2.123
F(000)	696.0
Crystal size/mm^3	0.2 × 0.2 × 0.05
Radiation	CuKα (λ = 1.54178)
2Θ range for data collection/°	7.414 to 133.028
Index ranges	$-6 \leq h \leq 6, -19 \leq k \leq 19, -19 \leq l \leq 19$
Reflections collected	77491
Independent reflections	2646 [R_{int} = 0.0647, R_{sigma} = 0.0179]
Data/restraints/parameters	2646/0/211
Goodness-of-fit on F^2	1.020
Final R indexes [I>=2σ (I)]	R_1 = 0.0311, wR_2 = 0.0897
Final R indexes [all data]	R_1 = 0.0311, wR_2 = 0.0898
Largest diff. peak/hole / e Å^{-3}	0.21/-0.23
Flack parameter	0.047(7)

Table 2 Fractional Atomic Coordinates (×10[4]) and Equivalent Isotropic Displacement Parameters (Å[2]×10[3]) for **5j**. U_{eq} is defined as 1/3 of the trace of the orthogonalised U_{IJ} tensor.

Atom	*x*	*y*	*z*	U(eq)
S001	8401.8(13)	1708.1(3)	2774.1(3)	52.0(2)
O002	7585(4)	2007.0(10)	4245.5(9)	54.8(5)
O003	3033(3)	3193.7(10)	4148.7(10)	46.9(4)
O004	3625(5)	7378.4(11)	4467.4(14)	76.3(7)
O005	1497(5)	6023.3(12)	4885.3(17)	82.9(7)
N006	5331(5)	7014.9(12)	4145.3(12)	54.7(6)
O007	7104(5)	7371.5(11)	3865.4(12)	71.4(6)
C008	5290(6)	3221.2(15)	2658.4(13)	51.9(6)
C009	5465(4)	3543.2(13)	4095.3(13)	38.7(5)
C00A	5229(5)	6139.1(13)	4103.1(13)	44.5(5)
C00B	6786(4)	3148.8(12)	3407.1(11)	37.7(4)
C00C	7088(6)	5748.7(16)	3699.6(16)	60.8(7)
C00D	7506(4)	2282.1(13)	3603.9(13)	38.7(5)
C00E	8599(5)	89.1(14)	3082.4(16)	51.5(6)
C00F	7158(6)	4918.2(15)	3669.7(17)	57.9(7)
C00G	11843(5)	891.2(16)	3656.4(16)	53.9(6)
C00H	5339(4)	4461.2(13)	4044.8(12)	38.5(5)
C00I	11768(6)	-549.1(17)	3847.0(16)	60.5(7)
C00J	3459(5)	4857.9(14)	4423.8(15)	48.4(5)
C00K	9654(6)	-604.2(16)	3402.0(18)	62.0(7)
C00L	3362(5)	5704.1(14)	4470.9(15)	48.9(5)
C00M	9689(4)	832.7(13)	3215.8(14)	43.5(5)
C00N	12861(5)	203.1(18)	3972.8(17)	60.7(7)

CCDC 2218965 contains the supplementary crystallographic data for this paper, including structure factors and refinement instructions. These data can be obtained free of charge from The Cambridge Crystallographic Data Centre, 12 Union Road.

h) Crystal data of **6j**

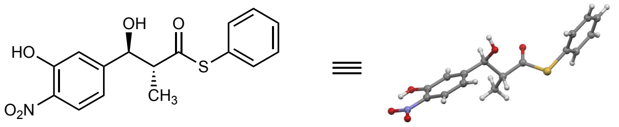

Table 1 Crystal data and structure refinement for **6j**.

Identification code	**6j**
Empirical formula	$C_{16}H_{15}NO_5S$
Formula weight	333.35
Temperature/K	200
Crystal system	orthorhombic
Space group	$P2_12_12_1$
a/Å	6.2035(5)
b/Å	15.5987(14)
c/Å	15.8803(15)
α/°	90
β/°	90
γ/°	90
Volume/Å^3	1536.7(2)
Z	4
ρ_{calc}g/cm^3	1.441
μ/mm^{-1}	0.236
F(000)	696.0
Crystal size/mm^3	0.2 × 0.03 × 0.03
Radiation	MoKα (λ = 0.71073)
2Θ range for data collection/°	3.66 to 60.092
Index ranges	$-8 \le h \le 8$, $-21 \le k \le 21$, $-22 \le l \le 20$
Reflections collected	21505
Independent reflections	4488 [R_{int} = 0.0675, R_{sigma} = 0.0713]
Data/restraints/parameters	4488/0/211
Goodness-of-fit on F^2	1.041
Final R indexes [I>=2σ (I)]	R_1 = 0.0648, wR_2 = 0.1604
Final R indexes [all data]	R_1 = 0.0928, wR_2 = 0.1730
Largest diff. peak/hole / e Å^{-3}	1.16/-0.39
Flack parameter	0.08(5)

Table 2 Fractional Atomic Coordinates (×10[4]) and Equivalent Isotropic Displacement Parameters (Å[2]×10[3]) for **6j**. U_{eq} is defined as 1/3 of the trace of the orthogonalised U_{IJ} tensor.

Atom	*x*	*y*	*z*	U(eq)
S001	1225.8(18)	8113.1(6)	7269.0(6)	30.5(3)
O002	866(5)	6507(2)	5638(2)	41.7(8)
O003	3433(7)	8313(2)	5870(2)	46.7(10)
O004	7508(9)	2691(2)	5487(3)	70.4(14)
N005	5863(8)	2914(2)	5830(2)	43.9(11)
O006	4706(8)	2399(2)	6207(3)	62.7(12)
O007	8358(7)	4229(2)	5016(3)	59.8(11)
C008	3144(6)	6426(3)	5687(3)	29.0(9)
C009	3806(7)	6918(2)	6490(2)	28.9(8)
C00A	3865(7)	5502(2)	5717(2)	27.1(8)
C00B	5819(7)	5266(2)	5375(2)	28.8(9)
C00C	409(7)	9162(2)	6968(2)	27.2(8)
C00D	6527(7)	4416(3)	5404(3)	29.8(9)
C00E	2937(7)	7821(3)	6425(3)	28.6(9)
C00F	5216(8)	3807(3)	5795(2)	31.0(9)
C00G	2559(8)	4893(3)	6112(3)	38.1(11)
C00H	-1659(8)	9270(3)	6645(3)	37.8(10)
C00I	6222(8)	6939(3)	6615(3)	41.6(11)
C00J	1747(8)	9852(3)	7063(3)	38.7(11)
C00K	3231(8)	4046(3)	6148(3)	39.9(11)
C00L	-2332(10)	10077(4)	6410(3)	49.2(14)
C00M	1038(13)	10662(3)	6825(3)	57.5(16)
C00N	-989(12)	10779(3)	6502(3)	58.0(18)

CCDC 2219075 contains the supplementary crystallographic data for this paper, including structure factors and refinement instructions. These data can be obtained free of charge from The Cambridge Crystallographic Data Centre, 12 Union Road.

i) Crystal data of **5p**

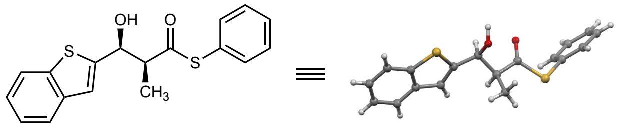

Table 1 Crystal data and structure refinement for **5p**.

Identification code	**5p**
Empirical formula	$C_{18}H_{16}O_2S_2$
Formula weight	328.43
Temperature/K	200
Crystal system	orthorhombic
Space group	$P2_12_12_1$
a/Å	5.7964(2)
b/Å	14.0425(5)
c/Å	19.3052(6)
α/°	90
β/°	90
γ/°	90
Volume/Å^3	1571.36(9)
Z	4
ρ_{calc}g/cm^3	1.388
μ/mm^{-1}	0.343
F(000)	688.0
Crystal size/mm^3	0.2 × 0.06 × 0.06
Radiation	MoKα (λ = 0.71073)
2Θ range for data collection/°	3.586 to 69.674
Index ranges	$-9 \leq h \leq 9$, $-20 \leq k \leq 22$, $-24 \leq l \leq 31$
Reflections collected	35340
Independent reflections	5986 [R_{int} = 0.0318, R_{sigma} = 0.0204]
Data/restraints/parameters	5986/0/201
Goodness-of-fit on F^2	1.082
Final R indexes [I>=2σ (I)]	R_1 = 0.0299, wR_2 = 0.0789
Final R indexes [all data]	R_1 = 0.0348, wR_2 = 0.0814
Largest diff. peak/hole / e Å^{-3}	0.27/-0.19
Flack parameter	0.007(11)

Table 2 Fractional Atomic Coordinates (×10⁴) and Equivalent Isotropic Displacement Parameters (Å²×10³) for **5p**. U_{eq} is defined as 1/3 of the trace of the orthogonalised U_{IJ} tensor.

**Atom**	***x***	***y***	***z***	**U(eq)**
S001	1695.6(6)	4608.7(3)	4407.9(2)	30.95(9)
S002	7862.6(8)	6849.5(3)	6897.3(2)	35.55(10)
O003	2435(2)	6164.5(8)	5347.0(7)	38.0(3)
O004	7246(3)	7277.4(9)	5587.2(6)	45.9(3)
C005	9338(3)	7947.3(10)	6827.1(7)	27.4(3)
C006	5650(2)	3989.0(11)	4839.7(7)	27.2(3)
C007	4759(2)	3259.7(10)	4388.8(7)	25.2(2)
C008	8432(3)	8761.2(12)	7127.8(8)	32.5(3)
C009	4208(2)	4755.9(10)	4885.1(7)	25.1(2)
C00A	2617(2)	3516.2(11)	4101.6(7)	27.0(3)
C00B	6996(3)	6670.9(10)	6021.3(7)	28.0(3)
C00C	5739(3)	2378.7(12)	4207.6(8)	32.0(3)
C00D	9654(3)	9611.1(13)	7095.8(9)	38.9(3)
C00E	4598(3)	5724.6(10)	5199.9(7)	28.2(3)
C00F	6046(3)	5681.2(10)	5865.9(7)	27.6(3)
C00G	11763(3)	9639.7(12)	6763.8(9)	39.2(4)
C00H	11466(3)	7979.1(12)	6497.9(8)	32.9(3)
C00I	12663(3)	8826.4(14)	6461.6(9)	37.6(3)
C00J	4608(3)	1805.7(13)	3735.6(8)	37.1(3)
C00K	1497(3)	2930.0(13)	3621.0(8)	36.1(3)
C00L	2527(3)	2086.9(13)	3439.2(8)	39.1(4)
C00M	4711(4)	5239.0(12)	6465.3(8)	37.8(4)

CCDC 2219021 contains the supplementary crystallographic data for this paper, including structure factors and refinement instructions. These data can be obtained free of charge from The Cambridge Crystallographic Data Centre, 12 Union Road,

j) Crystal data of **5w**

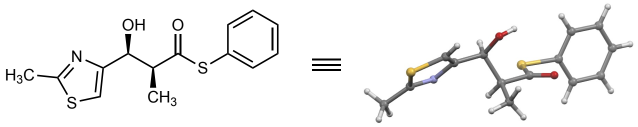

Table 1 Crystal data and structure refinement for **5w**.

Identification code	**5w**
Empirical formula	$C_{84}H_{90}N_6O_{12}S_{12}$
Formula weight	1760.33
Temperature/K	210
Crystal system	monoclinic
Space group	$P2_1$
a/Å	14.2812(8)
b/Å	17.8051(9)
c/Å	17.7182(9)
α/°	90
β/°	90.709(3)
γ/°	90
Volume/Å^3	4505.0(4)
Z	2
ρ_{calc}g/cm^3	1.298
μ/mm^{-1}	3.194
F(000)	1848.0
Crystal size/mm^3	0.1 × 0.03 × 0.03
Radiation	CuKα (λ = 1.54178)
2Θ range for data collection/°	4.988 to 132.712
Index ranges	$-15 \leq h \leq 15$, $-21 \leq k \leq 21$, $-20 \leq l \leq 21$
Reflections collected	150609
Independent reflections	15423 [R_{int} = 0.0948, R_{sigma} = 0.0481]
Data/restraints/parameters	15423/1/998
Goodness-of-fit on F^2	1.041
Final R indexes [I>=2σ (I)]	R_1 = 0.0681, wR_2 = 0.1838
Final R indexes [all data]	R_1 = 0.0751, wR_2 = 0.1918
Largest diff. peak/hole / e Å^{-3}	0.75/-0.51
Flack parameter	0.145(19)

Table 2 Fractional Atomic Coordinates (×10⁴) and Equivalent Isotropic Displacement Parameters (Å²×10³) for **5w**. U_{eq} is defined as 1/3 of the trace of the orthogonalised U_{IJ} tensor.

Atom	*x*	*y*	*z*	U(eq)
S1	8015.3(11)	5070.4(9)	5926.2(13)	72.3(5)
S2	6548.3(14)	8702.6(10)	4927.0(17)	89.9(7)
S3	1822.6(12)	3355.9(11)	8783.9(19)	101.9(9)
S4	3317.8(12)	6846.5(9)	10113.7(14)	76.2(6)
S5	-1555.9(12)	5576.5(16)	9253.4(12)	88.2(7)
S6	31.8(15)	2660.6(13)	11615.4(14)	89.2(7)
S7	5075.9(13)	4357.3(18)	11025.9(11)	96.7(8)
S8	6666.8(14)	3405.5(15)	7399.3(9)	83.2(6)
S9	1290.0(12)	7195.9(13)	6005.2(11)	75.1(5)
S10	-301.8(12)	4578.7(12)	3287.6(11)	74.3(5)
S11	4700.2(13)	6411.0(15)	4021.6(11)	83.8(6)
S12	3197.2(13)	4969.5(19)	7435.6(11)	99.9(9)
O1	8786(3)	6969(2)	4888(3)	60.4(11)
O2	9470(3)	5861(3)	6332(4)	93(2)
O3	217(3)	4055(3)	8825(3)	73.8(14)
O4	1041(3)	5147(2)	10249(2)	53.1(9)
O5	-3006(4)	5403(4)	10120(3)	95(2)
O6	-2313(3)	3586(3)	10245(3)	67.8(13)
N7	6764(3)	3600(3)	8816(3)	48.7(10)
O8	3576(3)	3758(3)	10424(3)	70.6(12)
O9	4436(3)	4442(3)	8768(2)	55.8(10)
O10	2086(3)	5403(3)	4675(2)	57.6(10)
O11	2753(3)	7158(3)	5121(3)	76.3(14)
O12	5317(3)	6300(3)	6180(2)	59.6(11)
O13	6168(4)	5611(5)	4407(4)	101(2)
N1	6391(3)	7387(3)	5463(3)	56.8(12)
N2	3389(3)	5528(3)	9558(3)	48.0(10)
N3	103(3)	3879(3)	10869(3)	51.6(11)
N4	-357(3)	5730(3)	4139(3)	49.3(11)
N5	3024(3)	5410(3)	6078(3)	50.0(11)
C1	4931(6)	8119(5)	5628(7)	95(3)
C2	5922(5)	8003(4)	5362(5)	73(2)
C3	7282(4)	7446(3)	5169(4)	52.7(13)
C4	7930(4)	6784(3)	5248(3)	47.4(12)

Table 2 Fractional Atomic Coordinates (×10^4) and Equivalent Isotropic Displacement Parameters (Å^2×10^3) for **5w**. U_{eq} is defined as 1/3 of the trace of the orthogonalised U_{IJ} tensor.

**Atom**	***x***	***y***	***z***	**U(eq)**
C5	8087(4)	6592(3)	6086(4)	51.2(13)
C6	8680(4)	5896(3)	6158(4)	52.5(13)
C7	8875(4)	4355(3)	6043(4)	56.1(14)
C8	9236(5)	4005(4)	5415(4)	61.3(15)
C9	9868(5)	3433(4)	5501(4)	58.6(14)
C10	10141(4)	3202(4)	6216(4)	57.1(14)
C11	9149(4)	4135(4)	6755(4)	55.8(14)
C12	9783(4)	3553(4)	6844(4)	61.0(15)
C13	8529(6)	7242(5)	6543(5)	77(2)
C14	7485(5)	8109(4)	4870(5)	72(2)
C15	-57(3)	1310(2)	8587(3)	69.0(18)
C16	355(3)	1613(3)	7949(2)	75(2)
C17	918(3)	2247(2)	8012(2)	64.2(16)
C18	1070(3)	2578(2)	8714(3)	56.9(15)
C26	658(3)	2275(3)	9352(2)	71.2(18)
C25	95(3)	1641(3)	9289(2)	70.3(19)
C19	1039(4)	4126(3)	8867(3)	47.9(12)
C20	1562(4)	4854(3)	8977(4)	51.6(13)
C21	1834(4)	4968(3)	9806(3)	45.5(11)
C22	2514(4)	5611(3)	9878(3)	47.6(12)
C23	3883(4)	6141(3)	9648(4)	55.7(14)
C24	4876(5)	6234(4)	9379(5)	75(2)
C27	1014(5)	5512(4)	8641(4)	64.0(16)
C28	2356(4)	6272(4)	10200(5)	67.8(18)
C29	-3687(4)	6764(4)	7773(4)	63.9(17)
C30	-3323(5)	7141(5)	8388(4)	70.0(17)
C31	-2673(5)	6772(5)	8869(4)	68.1(17)
C32	-2422(4)	6048(4)	8712(4)	58.3(15)
C33	-2206(4)	5265(4)	10041(4)	55.4(14)
C34	-1595(4)	4790(4)	10575(3)	56.8(14)
C35	-1463(4)	3998(4)	10259(3)	50.2(13)
C36	-774(4)	3577(3)	10735(3)	50.9(13)
C37	598(4)	3467(4)	11334(4)	58.1(14)
C38	1556(5)	3660(5)	11612(5)	78(2)

Table 2 Fractional Atomic Coordinates (×10⁴) and Equivalent Isotropic Displacement Parameters (Å^2×10^3) for **5w**. U_{eq} is defined as 1/3 of the trace of the orthogonalised U_{IJ} tensor.

Atom	*x*	*y*	*z*	U(eq)
C39	-927(5)	2922(5)	11084(5)	78(2)
C40	-2006(6)	4793(6)	11366(4)	78(2)
C41	-2806(5)	5685(4)	8090(4)	62.5(16)
C42	-3433(5)	6057(5)	7629(4)	65.3(17)
C43	8193(5)	2988(5)	8339(5)	71.5(19)
C44	7228(4)	3330(4)	8259(3)	54.3(13)
C45	5901(4)	3878(3)	8585(3)	48.7(12)
C46	5237(4)	4167(3)	9161(3)	45.1(11)
C47	4977(4)	3533(3)	9721(3)	49.0(12)
C48	4396(4)	3833(4)	10358(3)	52.1(13)
C49	4225(4)	4586(4)	11704(3)	82(3)
C50	4127(4)	4143(3)	12345(3)	96(3)
C51	3530(5)	4367(4)	12916(3)	112(4)
C52	3029(4)	5034(4)	12846(3)	103(3)
C53	3127(4)	5477(4)	12205(4)	117(4)
C54	3725(4)	5252(4)	11634(3)	96(3)
C55	5728(5)	3819(5)	7841(4)	70.7(19)
C56	4470(5)	2884(4)	9335(4)	64.6(16)
C57	-1872(5)	5500(6)	3479(4)	77(2)
C58	-868(4)	5339(4)	3677(3)	57.8(15)
C59	535(4)	5434(4)	4218(3)	50.2(13)
C60	1224(4)	5803(3)	4737(3)	48.9(12)
C61	1349(4)	6649(4)	4554(3)	54.2(14)
C62	1948(4)	7014(3)	5171(4)	56.0(14)
C63	2145(4)	7506(4)	6651(3)	81(2)
C64	2538(5)	8219(4)	6609(5)	122(4)
C65	3175(5)	8456(5)	7159(6)	159(8)
C66	3419(5)	7980(7)	7751(5)	180(10)
C70	3026(6)	7267(7)	7794(3)	162(7)
C69	2389(5)	7030(4)	7244(4)	116(4)
C67	1770(5)	6783(5)	3781(4)	75(2)
C68	690(4)	4814(5)	3803(4)	66.4(17)
C71	1608(5)	4771(5)	6535(4)	75(2)
C72	2573(4)	5064(5)	6605(4)	64.0(17)

Table 2 Fractional Atomic Coordinates (×10^4) and Equivalent Isotropic Displacement Parameters (Å^2×10^3) for **5w**. U_{eq} is defined as 1/3 of the trace of the orthogonalised U_{IJ} tensor.

**Atom**	***x***	***y***	***z***	**U(eq)**
C73	3906(4)	5641(4)	6321(3)	54.3(14)
C74	4542(4)	6015(4)	5770(3)	49.2(12)
C75	4862(4)	5404(4)	5207(4)	56.6(14)
C76	5383(4)	5746(5)	4565(4)	63.0(17)
C77	5476(3)	6569(3)	3269(2)	77(2)
C78	5554(4)	6036(3)	2700(3)	90(3)
C79	6129(4)	6173(4)	2087(3)	95(3)
C80	6625(4)	6843(4)	2044(3)	88(3)
C81	6547(4)	7376(3)	2614(4)	105(3)
C82	5972(4)	7239(3)	3226(3)	96(3)
C83	5445(5)	4790(5)	5579(5)	73.7(19)
C84	4116(5)	5459(6)	7038(4)	84(3)

CCDC 2218809 contains the supplementary crystallographic data for this paper, including structure factors and refinement instructions. These data can be obtained free of charge from The Cambridge Crystallographic Data Centre, 12 Union Road.

k) Crystal data of **S5c**

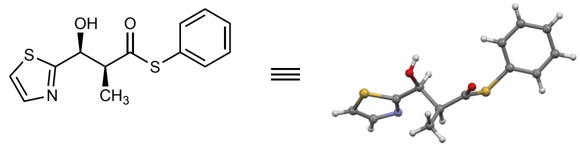

Table 1 Crystal data and structure refinement for **S5c**.

Identification code	**S5c**
Empirical formula	$C_{13}H_{13}NO_2S_2$
Formula weight	279.36
Temperature/K	296.15
Crystal system	monoclinic
Space group	$P2_1$
a/Å	9.8218(5)
b/Å	16.5687(8)
c/Å	13.0127(6)
α/°	90
β/°	90.799(2)
γ/°	90
Volume/Å^3	2117.41(18)
Z	6
ρ_{calc}g/cm^3	1.315
μ/mm^{-1}	0.370
F(000)	876.0
Crystal size/mm^3	0.2 × 0.1 × 0.1
Radiation	MoKα (λ = 0.71073)
2Θ range for data collection/°	3.13 to 59.014
Index ranges	-13 ≤ h ≤ 13, -22 ≤ k ≤ 22, -18 ≤ l ≤ 18
Reflections collected	82809
Independent reflections	11795 [R_{int} = 0.0334, R_{sigma} = 0.0232]
Data/restraints/parameters	11795/759/603
Goodness-of-fit on F^2	1.013
Final R indexes [I>=2σ (I)]	R_1 = 0.0358, wR_2 = 0.0860
Final R indexes [all data]	R_1 = 0.0511, wR_2 = 0.0936
Largest diff. peak/hole / e Å^{-3}	0.28/-0.20
Flack parameter	0.025(11)

Table 2 Fractional Atomic Coordinates (×10^4) and Equivalent Isotropic Displacement Parameters (Å^2×10^3) for **S5c**. U_{eq} is defined as 1/3 of the trace of the orthogonalised U_{IJ} tensor.

Atom	*x*	*y*	*z*	U(eq)
S001	6458.6(8)	4783.4(5)	-307.1(5)	55.02(18)
S002	3378.2(9)	7321.2(5)	2271.0(7)	62.2(2)
S003	470.1(7)	4058.8(6)	-1280.3(6)	63.5(2)
S004	7271.3(9)	3953.8(6)	5535.6(6)	68.1(2)
O00A	5192(2)	4381.2(14)	1404.1(14)	58.2(5)
O3	3999(2)	6050.0(13)	3714.2(16)	57.2(5)
O4	5312(2)	4351.8(13)	7061.5(15)	55.3(5)
O5	2143(2)	4603.9(13)	366.2(15)	55.1(5)
N00B	5923(2)	4951.1(14)	4450.7(16)	46.8(5)
N00C	2819(2)	6136.5(14)	1096.6(16)	43.6(5)
N00E	2695(2)	3977.3(16)	-2255.1(16)	52.4(5)
C00F	3334(2)	5688.2(15)	2872.2(18)	40.7(5)
C00G	7242(2)	5487.2(17)	539.9(18)	41.9(5)
C00H	2206(3)	4152.4(16)	-1361.8(19)	44.2(5)
C00I	5997(2)	4653.3(16)	5370.9(18)	42.7(5)
C00J	5089(3)	4890.2(17)	6239.4(19)	46.3(5)
C00K	2701(3)	6826(2)	512(2)	57.4(7)
C00L	8636(3)	5478(2)	678(2)	55.7(7)
C00M	6481(3)	6085.1(19)	996(2)	53.4(7)
C00N	1923(3)	5348.6(19)	3164(2)	50.1(6)
C00O	3037(3)	4455.9(17)	-459.9(19)	45.6(6)
C00Q	2108(3)	4613.4(19)	3831(2)	52.2(6)
C00R	6910(3)	4637(2)	3833(2)	58.7(7)
C00S	5214(3)	4316.8(17)	488(2)	45.8(5)
C00T	3165(2)	6308.5(15)	2041.2(19)	39.0(5)
C00U	4171(3)	3854.3(17)	-154(2)	46.9(6)
C00W	2949(3)	7514(2)	1015(3)	64.4(8)
C00Y	7105(3)	6665.2(19)	1591(2)	57.1(7)
C00Z	4275(4)	6101(2)	7220(2)	61.3(7)
C010	9243(3)	6078(2)	1261(3)	65.0(8)
C011	5404(3)	5774.9(18)	6554(2)	52.8(6)
C012	8485(3)	6668(2)	1719(2)	57.9(7)
C013	1662(4)	3764(2)	-2931(2)	61.2(8)
C014	1053(3)	5961(2)	3715(3)	72.9(10)

Table 2 Fractional Atomic Coordinates (×10^4) and Equivalent Isotropic Displacement Parameters (Å^2×10^3) for **S5c**. U_{eq} is defined as 1/3 of the trace of the orthogonalised U_{IJ} tensor.

**Atom**	***x***	***y***	***z***	**U(eq)**
C015	3649(3)	3118.7(19)	417(2)	58.5(7)
C016	416(4)	3771(2)	-2545(2)	67.0(8)
C017	7735(4)	4101(2)	4281(3)	71.6(9)
C01C	6769(4)	5865(2)	7085(3)	65.9(8)
S1	2816(8)	3751(2)	3122(3)	80.2(15)
C1	2656(10)	3022(6)	4072(7)	53(2)
C2	1453(8)	2583(6)	4102(6)	57(2)
C3	1297(7)	1976(5)	4829(6)	60(2)
C4	2346(10)	1809(4)	5525(5)	55(2)
C5	3550(8)	2249(5)	5495(6)	56(2)
C6	3705(8)	2856(6)	4768(8)	57(2)
O2	1778(16)	4536(10)	4696(11)	80(4)
S1A	2056(3)	3726.6(11)	3121.9(15)	62.6(6)
C1A	2472(5)	3021(3)	4081(4)	52.7(14)
C2A	1503(4)	2491(3)	4460(4)	69.1(15)
C3A	1851(5)	1957(3)	5245(4)	77.4(19)
C4A	3167(6)	1952(2)	5650(3)	66.8(16)
C5A	4137(5)	2482(3)	5271(3)	65.8(14)
C6A	3789(4)	3016(3)	4486(4)	57.0(13)
O2A	2311(7)	4652(5)	4755(6)	61.5(16)
S2	2824(5)	6384(5)	6534(3)	103.7(16)
C7	1872(9)	6893(5)	7488(8)	70(2)
C8	2255(8)	7667(5)	7787(8)	73(2)
C9	1431(8)	8110(4)	8438(6)	71(2)
C10	222(7)	7780(5)	8789(5)	66(2)
C11	-161(6)	7006(5)	8490(6)	70(2)
C12	664(9)	6563(4)	7840(7)	76(2)
O1	4389(13)	6224(6)	8153(7)	85(3)
S2A	3155(5)	6761(3)	6515(3)	74.1(11)
C7A	1893(12)	6988(7)	7420(10)	70(2)
C8A	1902(13)	7747(7)	7883(10)	77(2)
C9A	901(14)	7942(7)	8585(9)	81(2)
C10A	-70(11)	7368(9)	8786(8)	83(3)
C11A	-45(11)	6605(9)	8368(9)	89(3)

Table 2 Fractional Atomic Coordinates (×10^4) and Equivalent Isotropic Displacement Parameters (Å^2×10^3) for **S5c**. U_{eq} is defined as 1/3 of the trace of the orthogonalised U_{IJ} tensor.

**Atom**	***x***	***y***	***z***	**U(eq)**
C12A	951(12)	6411(8)	7661(10)	84(2)
O1A	4072(14)	5896(7)	8048(7)	92(4)

CCDC 2218969 contains the supplementary crystallographic data for this paper, including structure factors and refinement instructions. These data can be obtained free of charge from The Cambridge Crystallographic Data Centre, 12 Union Road.

l) Crystal data of **7c**

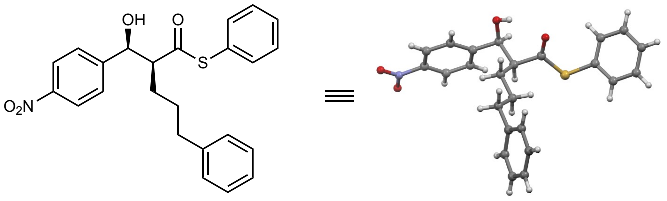

Table 1 Crystal data and structure refinement for **7c**.

Identification code	**7c**
Empirical formula	$C_{24}H_{23}NO_4S$
Formula weight	421.49
Temperature/K	200
Crystal system	monoclinic
Space group	$P2_1$
a/Å	12.2655(19)
b/Å	5.7696(9)
c/Å	16.091(2)
α/°	90
β/°	108.416(5)
γ/°	90
Volume/Å^3	1080.4(3)
Z	2
ρ_{calc}g/cm^3	1.296
μ/mm^{-1}	0.180
F(000)	444.0
Crystal size/mm^3	0.2 × 0.04 × 0.04
Radiation	MoKα (λ = 0.71073)
2Θ range for data collection/°	3.67 to 62.242
Index ranges	$-17 \le h \le 16$, $-8 \le k \le 8$, $-19 \le l \le 23$
Reflections collected	22707
Independent reflections	6130 [R_{int} = 0.0425, R_{sigma} = 0.0476]
Data/restraints/parameters	6130/1/272
Goodness-of-fit on F^2	1.037
Final R indexes [I>=2σ (I)]	R_1 = 0.0503, wR_2 = 0.1309
Final R indexes [all data]	R_1 = 0.0769, wR_2 = 0.1438
Largest diff. peak/hole / e Å^{-3}	0.71/-0.26
Flack parameter	0.05(3)

Table 2 Fractional Atomic Coordinates (×10^4) and Equivalent Isotropic Displacement Parameters (Å^2×10^3) for **7c**. U_{eq} is defined as 1/3 of the trace of the orthogonalised U_{IJ} tensor.

**Atom**	***x***	***y***	***z***	**U(eq)**
S001	6274.2(7)	2834.8(12)	6990.9(6)	52.9(2)
O002	4335(2)	9363(4)	5747.3(16)	56.2(6)
O003	6109(2)	7348(4)	7119.6(19)	64.3(7)
O004	-1001(3)	4495(6)	3094(2)	87.6(10)
C005	2824(2)	6862(5)	5008.3(18)	37.9(6)
N006	-689(3)	6202(7)	3543(2)	64.4(8)
C007	3247(3)	4061(5)	9154.7(19)	45.0(7)
C008	531(3)	6460(6)	4048.9(19)	47.1(7)
C009	3826(3)	4623(5)	7782.4(18)	40.4(6)
C00A	3843(3)	4791(6)	9991(2)	53.2(8)
C00B	3678(3)	6236(5)	6998.3(18)	40.0(6)
C00C	4063(3)	7028(5)	5549(2)	40.9(6)
C00D	5617(2)	5561(4)	6856.6(19)	38.6(6)
C00E	7716(3)	3318(5)	7651(2)	46.8(7)
C00F	4336(2)	5473(4)	6379.4(17)	34.9(5)
O00G	-1349(3)	7712(7)	3623(2)	92.4(10)
C00H	2052(3)	8615(5)	5020(2)	46.6(7)
C00I	3263(3)	1410(7)	10603(3)	65.9(11)
C00J	1276(3)	4683(6)	4024(2)	50.5(7)
C00K	904(3)	8425(6)	4537(2)	50.7(7)
C00L	2422(3)	4894(6)	4507(2)	47.9(7)
C00M	2682(3)	618(6)	9768(3)	61.7(9)
C00N	2685(3)	1935(6)	9049(2)	54.1(8)
C00O	3844(4)	3459(7)	10711(2)	63.2(10)
C00P	3170(3)	5558(6)	8367(2)	51.9(8)
C00Q	8368(3)	5090(9)	7503(3)	73.9(12)
C00R	9949(4)	3953(10)	8705(3)	80.2(13)
C00S	8189(4)	1800(8)	8309(3)	77.6(13)
C00T	9473(4)	5376(10)	8039(4)	89.7(16)
C00U	9311(4)	2102(11)	8840(4)	99.1(18)

CCDC 2218963 contains the supplementary crystallographic data for this paper, including structure factors and refinement instructions. These data can be obtained free of charge from The Cambridge Crystallographic Data Centre, 12 Union Road.

REFERENCES AND NOTES

1. Y. Yamashita, T. Yasukawa, W.-J. Yoo, T. Kitanosono, S. Kobayashi, Catalytic enantioselective aldol reactions. *Chem. Soc. Rev.* **47**, 4388–4480 (2018).

2. E. M. Carreira, R. A. Singer, W. Lee, Catalytic, enantioselective aldol additions with methyl and ethyl Acetate O-Silyl Enolates: A chiral tridentate chelate as a ligand for titanium(IV). *J. Am. Chem. Soc.* **116**, 8837–8838 (1994).

3. H. Ishitani, Y. Yamashita, H. Shimizu, S. Kobayashi, Highly *anti*-selective catalytic asymmetric aldol reactions. *J. Am. Chem. Soc.* **122**, 5403–5404 (2000).

4. G. L. Beutner, S. E. Denmark, Lewis base catalysis of the Mukaiyama directed aldol reaction: 40 years of inspiration and advances. *Angew. Chem. Int. Ed. Engl.* **52**, 9086–9096 (2013).

5. L. Lin, K. Yamamoto, H. Mitsunuma, Y. Kanzaki, S. Matsunaga, M. Kanai, Catalytic asymmetric iterative/domino aldehyde cross-aldol reactions for the rapid and flexible synthesis of 1,3-polyols. *J. Am. Chem. Soc.* **137**, 15418–15421 (2015).

6. T. Amatov, N. Tsuji, R. Maji, L. Schreyer, H. Zhou, M. Leutzsch, B. List, Confinement-controlled, either syn- or anti-selective catalytic asymmetric mukaiyama aldolizations of propionaldehyde enolsilanes. *J. Am. Chem. Soc.* **143**, 14475–14481 (2021).

7. B. M. Trost, C. S. Brindle, The direct catalytic asymmetric aldol reaction. *Chem. Soc. Rev.* **39**, 1600–1632 (2010).

8. B. List, Amine-catalyzed aldol reactions, in *Modern Aldol Reactions*, R. Mahrwald, Ed. (John Wiley & Sons, Ltd, 2004), pp. 161–200.

9. B. List, R. A. Lerner, C. F. Barbas, Proline-catalyzed direct asymmetric aldol reactions. *J. Am. Chem. Soc.* **122**, 2395–2396 (2000).

10. A. B. Northrup, D. W. C. MacMillan, Two-step synthesis of carbohydrates by selective aldol reactions. *Science* **305**, 1752–1755 (2004).

11. J. Wagner, R. A. Lerner, C. F. Barabas III, Efficient aldolase catalytic antibodies that use the enamine mechanism of natural enzymes. *Science* **270**, 1797–1800 (1995).

12. F. Tanaka, C. F. Barbas III, Antibody-catalyzed aldol reactions, in *Modern Aldol Reactions*, R. Mahrwald, Ed. (John Wiley & Sons, Ltd, 2004), pp. 273–310.

13. W.-D. Fessner, Enzyme-catalyzed aldol additions, in *Modern Aldol Reactions*, R. Mahrwald, Ed. (John Wiley & Sons, Ltd, 2004), pp. 201–272.

14. Y. M. A. Yamada, N. Yoshikawa, H. Sasai, M. Shibasaki, Direct catalytic asymmetric aldol reactions of aldehydes with unmodified ketones. *Angew. Chem. Int. Ed. Engl.* **36**, 1871–1873 (1997).

15. N. Yoshikawa, Y. M. A. Yamada, J. Das, H. Sasai, M. Shibasaki, Direct catalytic asymmetric aldol reaction. *J. Am. Chem. Soc.* **121**, 4168–4178 (1999).

16. B. M. Trost, H. Ito, A direct catalytic enantioselective aldol reaction via a novel catalyst design. *J. Am. Chem. Soc.* **122**, 12003–12004 (2000).

17. D. A. Evans, C. W. Downey, J. L. Hubbs, Ni(II) bis(oxazoline)-catalyzed enantioselective syn aldol reactions of N-propionylthiazolidinethiones in the presence of silyl triflates. *J. Am. Chem. Soc.* **125**, 8706–8707 (2003).

18. S. Saito, S. Kobayashi, Highly *anti*-selective catalytic aldol reactions of amides with aldehydes. *J. Am. Chem. Soc.* **128**, 8704–8705 (2006).

19. M. Iwata, R. Yazaki, Y. Suzuki, N. Kumagai, M. Shibasaki, Direct catalytic asymmetric aldol reactions of thioamides: Toward a stereocontrolled synthesis of 1,3-polyols. *J. Am. Chem. Soc.* **131**, 18244–18245 (2009).

20. Z. Liu, T. Takeuchi, R. Pluta, F. A. Arteaga, N. Kumagai, M. Shibasaki, Direct catalytic asymmetric aldol reaction of α-alkylamides. *Org. Lett.* **19**, 710–713 (2017).

21. S. C. D. Kennington, S. F. Teloxa, M. Mellado-Hidalgo, O. Galeote, S. Puddu, M. Bellido, P. Romea, F. Urpí, G. Aullón, M. Font-Bardia, Direct and enantioselective aldol reactions catalyzed by chiral Nickel(II) complexes. *Angew. Chem. Int. Ed.* **60**, 15307–15312 (2021).

22. T. Fujita, M. Yamane, W. M. C. Sameera, H. Mitsunuma, M. Kanai, Siloxy esters as traceless activators of carboxylic acids: Boron-catalyzed chemoselective asymmetric aldol reaction. *Angew. Chem. Int. Ed.* **60**, 24598–24604 (2021).

23. G. Lalic, A. D. Aloise, M. D. Shair, An exceptionally mild catalytic thioester aldol reaction inspired by polyketide biosynthesis. *J. Am. Chem. Soc.* **125**, 2852–2853 (2003).

24. D. Magdziak, G. Lalic, H. M. Lee, K. C. Fortner, A. D. Aloise, M. D. Shair, Catalytic enantioselective thioester aldol reactions that are compatible with protic functional groups. *J. Am. Chem. Soc.* **127**, 7284–7285 (2005).

25. J. Saadi, H. Wennemers, Enantioselective aldol reactions with masked fluoroacetates. *Nat. Chem.* **8**, 276–280 (2016).

26. H. Y. Bae, J. H. Sim, J.-W. Lee, B. List, C. E. Song, Organocatalytic enantioselective decarboxylative aldol reaction of malonic acid half thioesters with aldehydes. *Angew. Chem. Int. Ed.* **52**, 12143–12147 (2013).

27. S. Krautwald, E. M. Carreira, Stereodivergence in asymmetric catalysis. *J. Am. Chem. Soc.* **139**, 5627–5639 (2017).

28. J. F. Larrow, E. N. Jacobsen, in *Organometallics in Process Chemistry*, T. J. Colacot, V. Sivakumar, Eds. (Topics in Organometallic Chemistry, Springer, 2004).

29. T. P. Yoon, E. N. Jacobsen, Privileged chiral catalysts. *Science* **299**, 1691–1693 (2003).

30. H. Tokuyama, S. Yokoshima, S. C. Lin, L. P. Li, T. Fukuyama, Reduction of ethanethiol esters to aldehydes. *Synthesis* 10.1055/s-2002-31969, 1121–1123 (2002).

31. P. E. Dawson, T. W. Muir, I. Clark-Lewis, S. B. Kent, Synthesis of proteins by native chemical ligation. *Science* **266**, 776–779 (1994).

32. J. Franke, C. Hertweck, Biomimetic thioesters as probes for enzymatic assembly lines: Synthesis, applications, and challenges. *Cell Chem. Biol.* **23**, 1179–1192 (2016).

33. J. Lou, Q. Wang, P. Wu, H. Wang, Y.-G. Zhou, Z. Yu, Transition-metal mediated carbon–sulfur bond activation and transformations: an update. *Chem. Soc. Rev.* **49**, 4307–4359 (2020).

34. T. Cellnik, A. R. Healy, Sulfonyl chlorides as thiol surrogates for carbon–sulfur bond formation: One-pot synthesis of thioethers and thioesters. *J. Org. Chem.* **87**, 6454–6458 (2022).

35. Y. Shimada, Y. Matsuoka, R. Irie, T. Katsuki, Highly enantioselective cr(salen)-catalyzed mukaiyama aldol reaction: Construction of δ-hydroxy-β-keto ester derivatives. *Synlett.* 10.1055/s-2003-43351, 57–60 (2004).

36. S. Chen, D. Peng, H. Zhou, L. Wang, F. Chen, X. Feng, Highly enantioselective cyanoformylation of aldehydes catalyzed by a mononuclear Salen-Ti(O*i*Pr)$_4$ complex produced in situ. *Eur. J. Org. Chem.* **2007**, 639–644 (2007).

37. K. C. Fortner, M. D. Shair, Stereoelectronic effects dictate mechanistic dichotomy between Cu(II)-catalyzed and enzyme-catalyzed reactions of malonic acid half thioesters. *J. Am. Chem. Soc.* **129**, 1032–1033 (2007).

38. O. D. Engl, J. Saadi, E. Cosimi, H. Wennemers, Synthesis of monothiomalonates – versatile thioester enolate equivalents for C–C bond formations. *Helv. Chim. Acta* **100**, e1700196 (2017).

39. J. Le Nôtre, D. van Mele, C. G. Frost, A new method for constructing quaternary carbon centres: Tandem rhodium-catalysed 1,4-addition/intramolecular cyclisation. *Adv. Synth. Catal.* **349**, 432–440 (2007).

40. E. Szłyk, M. Barwiołek, R. Kruszynski, T. J. Bartczak, Synthesis and spectroscopic studies of the optically active copper(II), cobalt(II) and nickel(II) complexes with Schiff bases *N*,*N′*-(*1R*,*2R*)(−)-1,2-cyclohexylenebis(3-methoxybenzylideneiminato), *N*,*N′*-(*1R*,*2R*)(−)-1,2-cyclohexylenebis(5-

methoxybenzylideneiminato) and X-ray diffraction structure of the [Cu(II)(1*R*,2*R*)(−)chxnbis(5-methylbenzylideneiminato)$_2$]. *Inorganica Chim. Acta.* **358**, 3642–3652 (2005).

41. S.-B. Mou, W. Xiao, H.-Q. Wang, K.-Y. Chen, Z. Xiang, Syntheses of the carotane-type terpenoids (+)-schisanwilsonene A and (+)-tormesol via a two-stage approach. *Org. Lett.* **23**, 400–404 (2021).

42. T. Achard, Y. N. Belokon', J. A. Fuentes, M. North, T. Parsons, Influence of aromatic substituents on metal(II)salen catalysed, asymmetric synthesis of α-methyl α-amino acids. *Tetrahedron* **60**, 5919–5930 (2004).

43. A. M. Daly, T. Cormac Dalton, M. F. Renehan, D. G. Gilheany, Unsymmetrical salen ligands: Synthesis and use in chromium mediated asymmetric epoxidation. *Tetrahedron Lett.* **40**, 3617–3620 (1999).

44. Y. N. Belokon', S. Caveda-Cepas, B. Green, N. S. Ikonnikov, V. N. Khrustalev, V. S. Larichev, M. A. Moscalenko, M. North, C. Orizu, V. I. Tararov, M. Tasinazzo, G. I. Timofeeva, L. V. Yashkina, The asymmetric addition of trimethylsilyl cyanide to aldehydes catalyzed by chiral (Salen) titanium complexes. *J. Am. Chem. Soc.* **121**, 3968–3973 (1999).

45. B. Gao, D. Li, Y. Li, Q. Duan, R. Duan, X. Pang, Ring-opening polymerization of lactide using chiral salen aluminum complexes as initiators: High productivity and stereoselectivity†. *New J. Chem.* **39**, 4670–4675 (2015).

46. X. Yao, M. Qiu, W. Lü, H. Chen, Z. Zheng, Substituted salen–Ru (II) complexes as catalysts in the asymmetric cyclopropanation of styrene by ethyl diazoacetate: The influence of substituents and achiral additives on activity and enantioselectivity. *Tetrahedron Asymmetry* **12**, 197–204 (2001).

47. S. J. Chawner, M. J. Cases-Thomas, J. A. Bull, Divergent synthesis of cyclopropane-containing lead-like compounds, fragments and building blocks through a cobalt catalyzed cyclopropanation of phenyl vinyl sulfide. *Eur. J. Org. Chem.* **2017**, 5015–5024 (2017).

48. B. Legouin, M. Gayral, P. Uriac, J.-F. Cupif, N. Levoin, L. Toupet, P. van de Weghe, Molecular tweezers: Synthesis and formation of host–guest complexes. *Eur. J. Org. Chem.* **2010**, 5503–5508 (2010).

49. K. Nishioka, H. Goto, H. Sugimoto, Dual catalyst system for asymmetric alternating copolymerization of carbon dioxide and cyclohexene oxide with chiral aluminum complexes: Lewis base as catalyst activator and lewis acid as monomer activator. *Macromolecules* **45**, 8172–8192 (2012).

50. M. Barwiolek, E. Szlyk, A. Surdykowski, A. Wojtczak, New nickel(II) and copper(II) complexes with unsymmetrical Schiff bases derived from (1*R*,2*R*)(−)cyclohexanediamine and the application of Cu(II) complexes for hybrid thin layers deposition†. *Dalton Trans.* **42**, 11476–11487 (2013).

51. J. Lopez, S. Liang, X. R. Bu, Unsymmetric chiral salen Schiff bases: A new chiral ligand pool from bis-schiff bases containing two different salicylaldehyde units. *Tetrahedron Lett.* **39**, 4199–4202 (1998).

52. M. Christlieb, J. E. Davies, J. Eames, R. Hooley, S. Warren, The stereoselective synthesis of oxetanes; exploration of a new, Mitsunobu-style procedure for the cyclisation of 1,3-diols. *J. Chem. Soc. Perkin Trans. 1* **10.1039/B106851B**, 2983–2996 (2001).

53. C. Zhao, T. A. Mitchell, R. Vallakati, L. M. Pérez, D. Romo, Mechanistic investigations of the ZnCl2-mediated tandem mukaiyama aldol lactonization: Evidence for asynchronous, concerted transition states and discovery of 2-oxopyridyl ketene acetal variants. *J. Am. Chem. Soc.* **134**, 3084–3094 (2012).

54. R. Nagase, N. Matsumoto, K. Hosomi, T. Higashi, S. Funakoshi, T. Misaki, Y. Tanabe, Ti-direct, powerful, stereoselective aldol-type additions of esters and thioesters to carbonyl compounds: application to the synthesis and evaluation of lactone analogs of jasmone perfumes†. *Org. Biomol. Chem.* **5**, 151–159 (2007).

55. H.-Q. Cao, H.-N. Liu, Z.-Y. Liu, B. Qiao, F.-G. Zhang, J.-A. Ma, Silver-promoted direct phosphorylation of bulky C(sp^2)–H bond to build fully substituted β-phosphonodehydroamino acids. *Org. Lett.* **22**, 6414–6419 (2020).

56. M. Kannan, T. Punniyamurthy, Effect of ligand N,N-substituents on the reactivity of chiral copper(II) salalen, salan, and salalan complexes toward asymmetric nitroaldol reactions. *Tetrahedron Asymmetry* **25**, 1331–1339 (2014).

57. A. Berkessel, M. Brandenburg, E. Leitterstorf, J. Frey, J. Lex, M. Schäfer, A practical and versatile access to dihydrosalen (Salalen) ligands: Highly enantioselective titanium in situ catalysts for asymmetric epoxidation with aqueous hydrogen peroxide. *Adv. Synth. Catal.* **349**, 2385–2391 (2007).

58. R. Akué-Gédu, H. El-Hafidi, B. Rigo, D. Couturier, On the synthesis of 5-ethyl meldrum's acid. *J. Heterocycl. Chem.* **43**, 365–369 (2006).

59. N. N. Biswas, T. T. Yu, Ö. Kimyon, S. Nizalapur, C. R. Gardner, M. Manefield, R. Griffith, D. StC. Black, N. Kumar, Synthesis of antimicrobial glucosamides as bacterial quorum sensing mechanism inhibitors. *Bioorg. Med. Chem.* **25**, 1183–1194 (2017).

60. B. Hin, P. Majer, T. Tsukamoto, Facile synthesis of α-substituted acrylate esters. *J. Org. Chem.* **67**, 7365–7368 (2002).

61. B. J. Dunn, K. R. Watts, T. Robbins, D. E. Cane, C. Khosla, Comparative analysis of the substrate specificity of *trans*- versus *cis*-acyltransferases of assembly line polyketide synthases. *Biochemistry* **53**, 3796–3806 (2014).

62. S. J. Mo, D. H. Kim, J. H. Lee, J. W. Park, D. B. Basnet, Y. H. Ban, Y. J. Yoo, S.-w. Chen, S. R. Park, E. A. Choi, E. Kim, Y.-Y. Jin, S.-K. Lee, J. Y. Park, Y. Liu, M. O. Lee, K. S. Lee, S. J. Kim, D. Kim, B. C. Park, S.-g. Lee, H. J. Kwon, J.-W. Suh, B. S. Moore, S.-K. Lim, Y. J. Yoon, Biosynthesis of the allylmalonyl-CoA extender unit for the FK506 polyketide synthase proceeds through a dedicated polyketide synthase and facilitates the mutasynthesis of analogues. *J. Am. Chem. Soc.* **133**, 976–985 (2011).
